# Rice *SST* Variation Shapes the Rhizosphere Bacterial Community, Conferring Tolerance to Salt Stress through Regulating Soil Metabolites

**DOI:** 10.1128/mSystems.00721-20

**Published:** 2020-11-24

**Authors:** Tengxiang Lian, Yingyong Huang, Xianan Xie, Xing Huo, Muhammad Qasim Shahid, Lei Tian, Tao Lan, Jing Jin

**Affiliations:** a Guangdong Provincial Key Laboratory of Plant Molecular Breeding, College of Agriculture, South China Agricultural University, Guangzhou, China; b Key Laboratory of Ministry of Education for Genetics, Breeding and Multiple Utilization of Crops, Fujian Agriculture and Forestry University, Fuzhou, Fujian, China; c Key Laboratory of Applied Genetics of Universities in Fujian Province, Fujian Agriculture and Forestry University, Fuzhou, Fujian, China; d Fujian Provincial Key Laboratory of Crop Breeding by Design, Fujian Agriculture and Forestry University, Fuzhou, Fujian, China; e State Key Laboratory of Conservation and Utilization of Subtropical Agro-Bioresources, College of Forestry and Landscape Architecture, South China Agricultural University, Guangzhou, China; f Lingnan Guangdong Laboratory of Modern Agriculture, College of Forestry and Landscape Architecture, South China Agricultural University, Guangzhou, China; g Guangdong Key Laboratory for Innovative Development and Utilization of Forest Plant Germplasm, College of Forestry and Landscape Architecture, South China Agricultural University, Guangzhou, China; h Guangdong Provincial Key Laboratory of New Technology in Rice Breeding, Rice Research Institute, Guangdong Academy of Agricultural Sciences, Guangzhou, China; i College of Agriculture, Ningxia University, Ningxia Yinchuan, China; University of California, Berkeley

**Keywords:** *Oryza sativa*, *SST* variation, rhizosphere bacterial community, soil metabolites, salt tolerant

## Abstract

Some plant-specific resistance genes could affect rhizosphere microorganisms by regulating the release of root exudates. In a previous study, the *SST* (seedling salt tolerant) gene in rice (Oryza sativa) was identified, and loss of *SST* function resulted in better plant adaptation to salt stress. However, whether the rice *SST* variation could alleviate salt stress via regulating soil metabolites and microbiota in the rhizosphere is still unknown. Here, we used transgenic plants with *SST* edited in the Huanghuazhan (HHZ) and Zhonghua 11 (ZH11) cultivars by the CRISPR/Cas9 system and found that loss of *SST* function increased the accumulation of potassium and reduced the accumulation of sodium ions in rice plants. Using 16S rRNA gene amplicon high-throughput sequencing, we found that the mutant material shifted the rhizobacterial assembly under salt-free stress. Importantly, under salt stress, the *sst*, HHZ*cas*, and ZH11*cas* plants significantly changed the assembly of the rhizobacteria. Furthermore, the rice *SST* gene also affected the soil metabolites, which were closely related to the dynamics of rhizosphere microbial communities, and we further determined the relationship between the rhizosphere microbiota and soil metabolites. Overall, our results show the effects of the rice *SST* gene on the response to salt stress associated with the soil microbiota and metabolites in the rhizosphere. This study reveals a helpful linkage among the rice *SST* gene, soil metabolites, and rhizobacterial community assembly and also provides a theoretical basis for improving crop adaptation through soil microbial management practices.

**IMPORTANCE** Soil salinization is one of the major environmental stresses limiting crop productivity. Crops in agricultural ecosystems have developed various strategies to adapt to salt stress. We used rice mutant and CRISPR-edited lines to investigate the relationships among the Squamosa promoter Binding Protein box (SBP box) family gene (*SST*/*OsSPL10*), soil metabolites, and the rhizosphere bacterial community. We found that during salt stress, there are significant differences in the rhizosphere bacterial community and soil metabolites between the plants with the *SST* gene and those without it. Our findings provide a useful paradigm for revealing the roles of key genes of plants in shaping rhizosphere microbiomes and their relationships with soil metabolites and offer new insights into strategies to enhance rice tolerance to high salt levels from microbial and ecological perspectives.

## Abstract (precis)

Soil salinization is one of the major environmental stresses limiting crop productivity. Crops in agricultural ecosystems have developed various strategies to adapt to salt stress. We used rice mutant and CRISPR-edited lines to investigate the relationships among the Squamosa promoter Binding Protein box (SBP box) family gene (*SST*/*OsSPL10*), soil metabolites, and the rhizosphere bacterial community. We found that during salt stress, there are significant differences in the rhizosphere bacterial community and soil metabolites between the plants with the *SST* gene and those without it. Our findings provide a useful paradigm for revealing the roles of key genes of plants in shaping rhizosphere microbiomes and their relationships with soil metabolites and offer new insights into strategies to enhance rice tolerance to high salt levels from microbial and ecological perspectives.

## INTRODUCTION

Soil salinization has already been a serious threat to agricultural ecosystems. About 15 to 50% of irrigated land in the world has suffered from salinity (1). The adverse effects of soil salinization on plant growth and fitness have severely restricted crop productivity (1). As the global population increases, the productivity of crops needs to be increased. Therefore, it is worthwhile to explore how to reduce the effects of salt stress in saline soils, enhance plant tolerance to salinity, and ultimately improve the yield of crops. As one of the most important cereal crops for human consumption in the world, rice (Oryza sativa) is very sensitive to salt stress, which retards rice growth and development and subsequently reduces yield and quality (2). In the past several years, researchers have attempted to improve the salt tolerance of rice by breeding salt-tolerant plant varieties, through transgenic technology, and by application of beneficial microbes (3, 4).

Rhizosphere microorganisms play an important role in the process of plant adaptation to salt stress (5, 6). These rhizobacteria are mainly composed of plant-growth-promoting rhizosphere bacteria (PGPR) and endophytic bacteria (1). It is worth mentioning that PGPR have been widely used as a microbial fertilizer for several years because of significant positive effects on crop yield and fitness (7, 8). For example, Sinorhizobium meliloti (*Alphaproteobacteria*), Bacillus megaterium, *Novosphingobium* sp., and *Rhodococcus* sp. are able to produce 1-aminocyclopropane-1-carboxylic acid (ACC)-deaminase, abscisic acid (ABA), and indole-3-acetic acid (IAA), which can overcome salt-induced growth inhibition, via reducing ethylene levels, stimulating root proliferation, and mitigating plant adaptation to water deficiency (9–14). Besides, PGPR can enhance the uptake of nutrient elements (including nitrogen, phosphorus, and potassium) and efficiency of water use in plant tissues and regulate Na^+^ homeostasis, which help the plant to endure salinity (15–17). However, whether the rhizobacteria could help rice cope with external salt stress is still largely unknown.

Genetically modified plants can improve the efficiency of crops to absorb nutrients and the ability to resist external stresses (18, 19). Plant-specific resistance genes could regulate the release of root exudates and consequently affect the rhizosphere microorganisms (20, 21). In turn, the dynamics of soil microorganisms can induce plant systemic tolerance by releasing metabolites, affecting hormones, and altering host gene expression (19, 21). For example, Azospirillum brasilense and *Enterobacter* sp. could trigger the transcription of salt stress-responsive genes in barley and *Arabidopsis*, respectively (22, 23). A rice nitrate transporter gene, *NRT1.1B*, is associated with the recruitment of *O. sativa* subsp. *indica* rice-enriched bacteria, which could improve rice growth under organic nitrogen conditions using the synthetic communities (18). Another study determined the important role of coumarins in the assembly of the rhizosphere microbiome and proved that plants and probiotics together trigger the production and excretion of scopolin, which depend on MYB72/BGLU42, thereby improving the microbial community structure and making it conducive to the growth and immunity of host plants (19). Recent studies illustrated that the root exudates and rhizosphere microorganisms were controlled by certain plant-specific genes and were closely related to the efficiency of use of plant nutrients and the ability of plants to resist stress (24, 25).

The root exudates secreted from root tissues have multiple functions to affect the abiotic and biotic processes in soils, including changing the physicochemical properties and recruiting beneficial microorganisms to resist external stresses (20, 26). The composition of root exudates is dependent on the expression levels of plant-specific genes (21, 27). When crops are subjected to various biotic and abiotic stresses, the secretion of root exudates regulated by the overexpression or mutation of stress-related genes will have a special signaling effect on the rhizospheres. These special signals can enrich and maintain the specific beneficial microorganisms (26, 28, 29). Many studies have demonstrated the effect of signals from root exudates on the interactions between plants and microorganisms in the rhizospheres, such as the small signaling molecules (including nonproteinogenic amino acids and acyl-homoserine lactones) (30, 31), polymers (32), and antimicrobials (33), or plant hormones, such as salicylic acid (27).

The SPL (Squamosa promoter binding Protein-Like) family genes are plant-specific transcription factors (TFs) with a highly conserved DNA binding domain SBP box, which consists of two zinc finger structures (34). SPLs play vital roles in plant growth and development, including lateral root development, shoot and leaf morphogenesis, floral organ development, flowering, and fruit ripening (35–38). In rice, there are some members of the SPL family that have been identified and characterized. For example, *OsSPL13/GLW7*, *OsSPL16*, and *OsSPL18* control grain size (39–41), *OsSPL8*/*OsLG1* controls inflorescence architecture (42, 43), and *OsSPL14*/*IPA1*/*WFP* controls tiller number and panicle branching (44, 45). Recently, accumulating evidence showed that SPLs are crucial regulators of plant tolerance to abiotic stresses (46–50). In our previous study, we found a single-gene recessive mutant (*sst* [seedling salt tolerant]), which showed seedling salt tolerance compared with the wild type (WT; R401) (2). Furthermore, using a map-based cloning method, we identified an SBP box gene (*SST*/*OsSPL10*, *Os06g0659100*) as the candidate for the *SST* gene and subsequently characterized it through gene knockout and overexpression approaches. *SST* knockout mutants are better adapted to salt stress (2). However, whether the rice *SST* gene can alleviate external salt stress via regulating metabolites and microbiota in the rhizospheres remains elusive.

In this study, we used transgenic plants of the Huanghuazhan (HHZ) and Zhonghua 11 (HHZ) cultivars that were *SST* edited by the CRISPR/Cas9 system. The target sites were located in the first exon of *SST*, and the edited transgenic plants showed loss of function of *SST* (see Fig. S1 in the supplemental material). Two pairs of positive transgenic plants (HHZ*cas* and ZH11*cas*), HHZ and ZH11 plants, and one pair of mutant (*sst*) and WT plants were planted in soils under salt and nonsalt stresses. We examined the rhizosphere bacterial community by 16S rRNA amplicon high-throughput sequencing and further determined the soil metabolites affected by the variation of *SST* gene, and the relationship between rhizosphere microorganisms and soil metabolites. This study provides a theoretical basis for improving crop fitness through rhizosphere microbial management practices.

10.1128/mSystems.00721-20.1 FIG S1 The gene framework of *SST* (*OsSPL10*). The coding region is indicated with blue boxes, the 5′ and 3′ untranslated regions (UTR) are indicated with white boxes, and the lines represent the introns. CRISPR/Cas9 editing of *SST* (*OsSPL10*) in ZH11 and HHZ was performed. TS1, target site 1; TS2, target site 2. Black triangles represent inserted nucleotides. Dashed lines represent deletion of nucleotides. Download FIG S1, PDF file, 0.01 MB. Copyright © 2020 Lian et al. 2020 Lian et al. This content is distributed under the terms of the Creative Commons Attribution 4.0 International license.

## RESULTS

### Loss of *SST* function alleviates salt stress responses and enhances rice growth.

**FIG 1:**
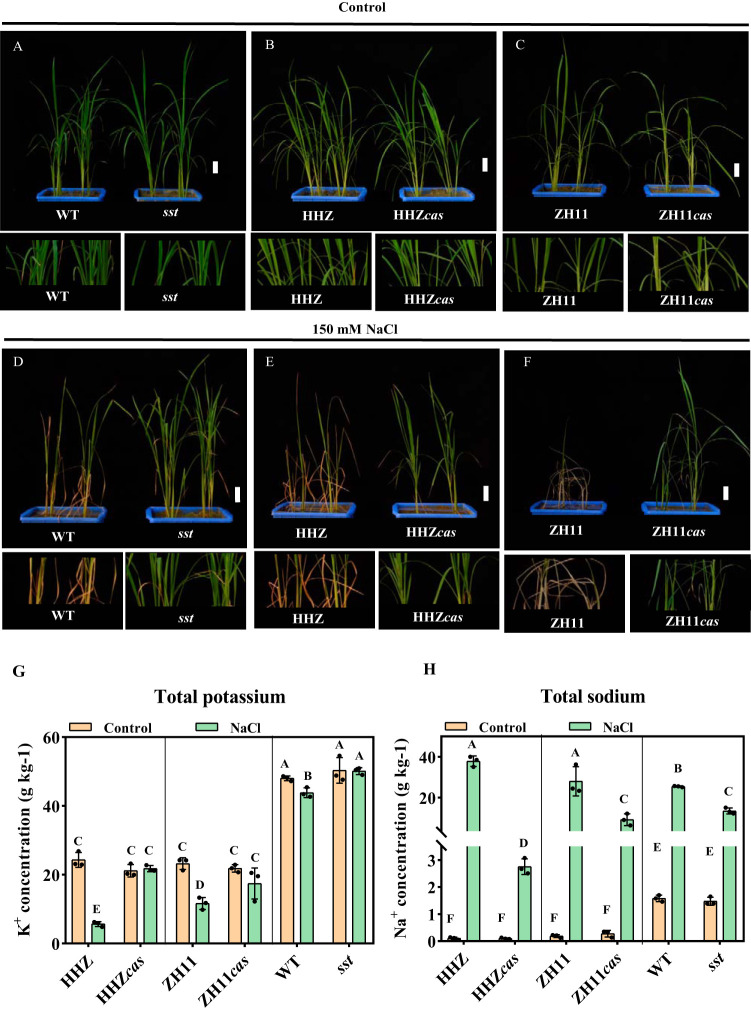
Phenotypes of WT, mutant (*sst*), HHZ, HHZ*cas*, ZH11, and ZH11*cas* rice plants after 20 days of control treatment (A to C) or 150 mM NaCl salt treatment (D to F). Size bars = 50 mm. The bottom of the image is divided into the corresponding local enlarged image. The control treatment was pure water. (G) K^+^ concentrations among the HHZ, HHZ*cas*, ZH11, ZH11*cas*, WT, and *sst* plants. (H) Na^+^ concentrations among the HHZ, HHZ*cas*, ZH11, ZH11*cas*, WT, and *sst* plants. One-way ANOVA, *n* = 3, *P* < 0.05.

In order to investigate the effect of *SST* mutation on salt tolerance, 150 mM NaCl was added to the soils of the mutant (*sst*), WT, and transgenic (T_2_) plants (HHZ*cas* and ZH11*cas*). Under standard growth conditions, all the rice seedlings were indistinguishable (Fig. 1A to C; see Fig. S2A to C in the supplemental material). Under salt treatment for 7 days, no visible phenotypic differences were detected in *sst*, HHZ*cas*, and ZH11*cas* plants. However, WT, HHZ, and ZH11 plants showed slight yellowing and stunted leaf tips (Fig. S2D to F). Moreover, after 20 days of treatment, *sst*, HHZ*cas*, and ZH11*cas* plants showed much more tolerance to salt stress than their corresponding wild types. WT, HHZ, and ZH11 seedlings almost died under the 20 days of salt treatment, but the *sst*, HHZ*cas*, and ZH11*cas* seedlings stayed alive (Fig. 1D to F).

10.1128/mSystems.00721-20.2 FIG S2 Phenotypes of WT, mutant (*sst*), HHZ, HHZ*cas*, ZH11, and ZH11*cas* plants after 10 days of control treatment (A to C) or 150 mM NaCl salt treatment (D to F). Size bars = 50 mm. The bottom of the image is divided into the corresponding local enlarged image. The control treatment was pure water. Download FIG S2, PDF file, 0.1 MB. Copyright © 2020 Lian et al. 2020 Lian et al. This content is distributed under the terms of the Creative Commons Attribution 4.0 International license.

In order to explore the underlying mechanism by which the *SST* gene regulated rice seedling salt tolerance, we further investigated the concentrations of Na^+^ and K^+^ in the plants under salt and control conditions. For these two ions, no significant differences in accumulation were observed between the plants with or without *SST* under the control condition. However, under salt stress, the concentration of K^+^ in the plants with the *SST* gene (HHZ, ZH11, and WT) was lower than in the plants without the *SST* gene (HHZ*cas*, ZH11*cas*, and *sst*), while Na^+^ concentration showed an opposite trend (Fig. 1G and H). These results indicated that the *SST* gene may alleviate the damage caused by salt stress through reducing the uptake of salt ions. Taken together, these results indicated that the mutation of the *SST* gene did not have a significant impact on rice growth in the absence of the salt stress condition, while gene deletion significantly improved rice growth under salt stress.

### Effects of salt stress on diversity of the bacterial community in the rhizospheres of various rice genotypes.

**FIG 2:**
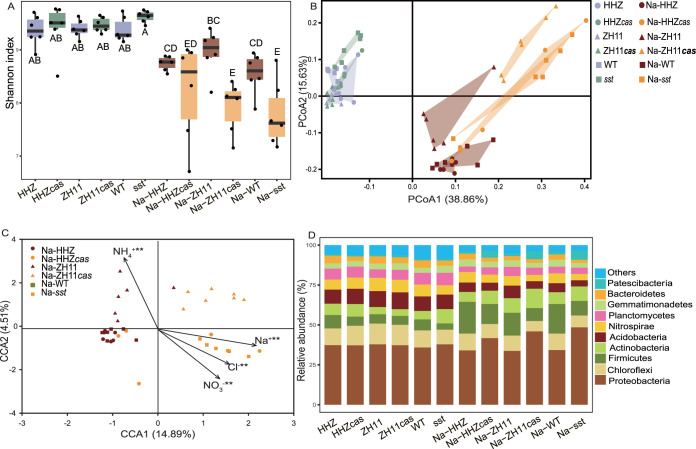
(A) Effects of NaCl and the *sst* gene on rice rhizosphere soil bacterial Shannon diversity index (one-way ANOVA, *n* = 6, *P* < 0.05). (B) Principal-coordinate analysis (PCoA) based on Bray-Curtis dissimilarities showing differences in rhizosphere bacterial community structure under the control and 150 mM NaCl salt conditions (PERMANOVA, *n* = 6, *P* < 0.05). (C) Canonical correspondence analysis (CCA) based on the bacterial community compositions of samples under the NaCl condition (Mantel test, *n* = 6, *P* < 0.05). (D) The relative abundances of the bacterial phyla.

To explore the genotype-mediated rhizosphere microbial community differences, the α-diversity (Shannon diversity) of the microbial community in each sample was estimated (Fig. 2A). The results showed that Shannon index values in nonsalinity soil were significantly higher than those in the salinity soil, and the Shannon index values for the ZH11*cas* and *sst* rhizosphere bacteria were significantly lower than those for the ZH11 and WT genotypes under salt treatment (one-way analysis of variance [ANOVA], *n* = 6, *P* < 0.05). For the β-diversity analysis, the principal-coordinate analysis (PCoA) showed that the soil bacterial community structures under salt stress were clearly distinguishable from those of the nonsalt conditions (Fig. 2B), indicating a significant effect of the salinity on soil microbiome assembly. Significant differences were observed between the microbial structures of the *sst* (Na-*sst*) and WT (Na-WT), HHZ (Na-HHZ) and HHZ*cas* (Na-HHZ*cas*), and ZH11 (Na-ZH11) and ZH11*cas* (Na-ZH11*cas*) genotypes, respectively, under the salt conditions (permutational multivariate ANOVA [PERMANOVA], *n* = 6, *P* < 0.05) (Table 1 and Fig. 2B to D). Moreover, the microbial structures of the WT and *sst* plants were also different under the nonsalt conditions. The rhizosphere microbial structures of the plants with the *SST* gene clearly are separate from those of the plants without *SST* genotypes, suggesting that the *SST* activity affects rice rhizosphere microbiome assembly.

**TABLE 1:** Effects of rice genotypes on bacterial community structure assessed by PERMANOVA

Pairwise comparison	*F* value	*R*^2^	*P* value^*a*^
HHZ vs HHZ*cas*	0.8544	0.0787	0.524
ZH11 vs ZH11*cas*	1.1143	0.1003	0.318
WT vs *sst*	5.5789	0.3581	0.003**
Na-HHZ vs Na-HHZ*cas*	2.5136	0.2009	0.015*
Na-ZH11 vs Na-ZH11*cas*	5.6461	0.3609	0.003**
Na-WT vs Na-*sst*	7.4013	0.4253	0.006**

a Statistically significant values are shown in boldface: *, *P* < 0.05; **, *P* < 0.01.

### Factors affecting bacterial community structure.

To compare the differences in soil physiochemical properties, we measured the pH, organic C, total N (TN), total P (TP), available P, available K, NH_4_^+^, NO_3_^−^, Na^+^, and Cl^−^ in the rhizosphere soils of the different rice genotypes (see Table S1 in the supplemental material). The application of salt decreased the soil pH of all the plant materials, while there were nonsignificant differences among the plant genotypes (two-way ANOVA, *n* = 6, *P* < 0.05). Under salt stress, the concentrations of NO_3_^−^, Na^+^, and Cl^−^ were increased, and the available K^+^ and NH_4_^+^ were decreased in the ZH11*cas*, HHZ*cas*, and *sst* genotypes compared with the ZH11, HHZ, and WT genotypes, respectively. Combined with the Na^+^ and K^+^ concentrations in the soil and plants, this finding showed that gene *SST* could affect sodium and potassium ion absorption (Fig. 1G and H). Canonical correspondence analysis (CCA) was performed to determine the relationships between the soil environmental factors and bacterial communities (Fig. 2C; see Fig. S3 in the supplemental material). The results of the Mantel test revealed that the bacterial community structures in the soil samples under salt stress correlated with the soil parameters of NH_4_^+^-N, NO_3_^−^-N, Na^+^, and Cl^−^ (Fig. 2C), and the bacterial community structures in all soil samples correlated with the soil parameters of NH_4_^+^-N, Na^+^, and Cl^−^ (see Fig. S3 in the supplemental material).

10.1128/mSystems.00721-20.3 FIG S3 Canonical correspondence analysis (CCA) based on the bacterial community compositions of all the samples (Mantel test, *n* = 6, *P* < 0.05). Download FIG S3, PDF file, 0.10 MB. Copyright © 2020 Lian et al. 2020 Lian et al. This content is distributed under the terms of the Creative Commons Attribution 4.0 International license.

10.1128/mSystems.00721-20.5 TABLE S1 Effects of NaCl treatment and different rice genotypes on soil physicochemical properties. Download Table S1, PDF file, 0.02 MB. Copyright © 2020 Lian et al. 2020 Lian et al. This content is distributed under the terms of the Creative Commons Attribution 4.0 International license.

### Rhizosphere soil bacterial composition response to salt stress.

A total of 4,359,024 high-quality bacterial sequences were obtained. The numbers of reads per sample ranged from 60,542 to 85,257. The high-quality reads were clustered into 10,444 operational taxonomic units (OTUs), with a mean of 3,802 OTUs per sample. Across all soil samples, the dominant bacterial phyla were *Proteobacteria*, *Firmicutes*, *Chloroflexi*, *Nitrospirae*, *Actinobacteria*, and *Acidobacteria*. Their relative abundances varied from 33.72 to 48.53%, 3.95 to 19.88%, 7.37 to 12.95%, 3.94 to 8.34%, 4.24 to 12.17%, and 3.88 to 10.52%, respectively, across all samples (Fig. 2D). The phyla with less relative abundances, such as *Gemmatimonadetes*, *Bacteroidetes*, *Planctomycetes*, and *Patescibacteria* were still detected in all soil samples (Fig. 2D). In detail, the relative abundances of *Proteobacteria* and *Actinobacteria* were significant higher in the rhizosphere soil of the plants with loss of function of the *SST* gene, while the relative abundances of *Chloroflexi*, *Firmicutes*, *Acidobacteria*, and *Nitrospirae* showed the opposite trend.

**FIG 3:**
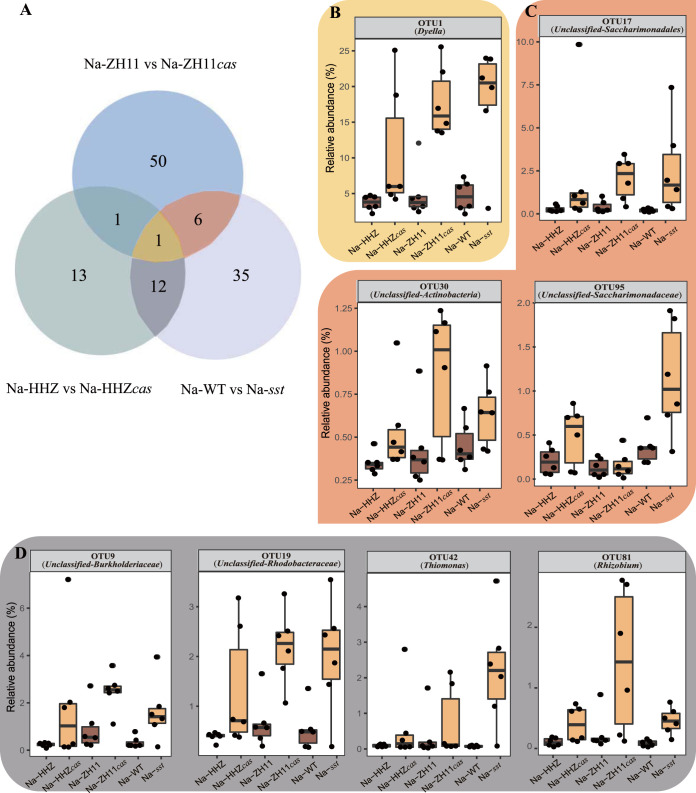
(A) Venn analysis of the OTUs that significantly differed in relative abundance between comparisons of Na-HHZ and Na-HHZ*cas*, Na-ZH11 and Na-ZH11*cas*, and Na-WT and Na-*sst* plants. (B to D) The relative abundances of the OTUs that were coenriched in the roots of plants with loss of function of *SST* under salt stress and coexisted in soils of three pairs of plant materials (B), in soils of Na-ZH11 and Na-ZH11*cas* and Na-WT and Na-*sst* plant materials (C), and in soils of Na-HHZ and Na-HHZ*cas* and Na-WT and Na-*sst* plant materials (D) (DESeq2, *n* = 6, *P* < 0.05). In panels B to D, different background colors correspond to different components in the Venn diagram.

Furthermore, under the saline conditions, we detected 58, 27, and 54 OTUs with significant differences between ZH11 and ZH11*cas*, HHZ and HHZ*cas*, and WT and *sst*, respectively (DESeq2, *n* = 6, *P* < 0.05). Among them, 20 OTUs coexisted in soils of at least two pairs of plant materials. OTU1 (*Dyella*), OTU17 (Unclassified-*Saccharimonadales*), OTU19 (Unclassified-*Rhodobacteraceae*), OTU9 (Unclassified-*Burkholderiaceae*), OTU81 (*Rhizobium*), OTU42 (*Thiomonas*), OTU30 (Unclassified-*Actinobacteria*), and OTU95 (Unclassified-*Saccharimonadaceae*) were increased in the ZH11*cas*, HHZ*cas*, and *sst* genotypes compared with the ZH11, HHZ and WT genotypes, respectively (Fig. 3). However, these OTUs were showed no significant differences between the plants with and those without the *SST* gene under the control condition (see Table S2 in the supplemental material).

10.1128/mSystems.00721-20.6 TABLE S2 Differentially expressed OTUs in the three pairs of plants under the no-salt and salt conditions (DESeq2, *n* = 6, *P* < 0.05). Download Table S2, PDF file, 0.2 MB. Copyright © 2020 Lian et al. 2020 Lian et al. This content is distributed under the terms of the Creative Commons Attribution 4.0 International license.

### Soil metabolites under salt stress.

**FIG 4:**
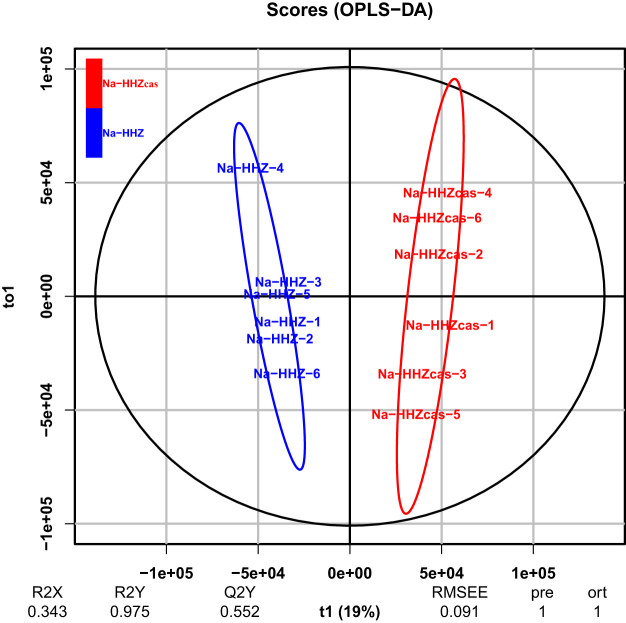
Orthogonal partial least-squares discrimination analysis (OPLS-DA) (*n* = 6, *P* < 0.05) showing differences in soil metabolites between HHZ and HHZ*cas* plants under the 150 mM NaCl condition.

**FIG 5:**
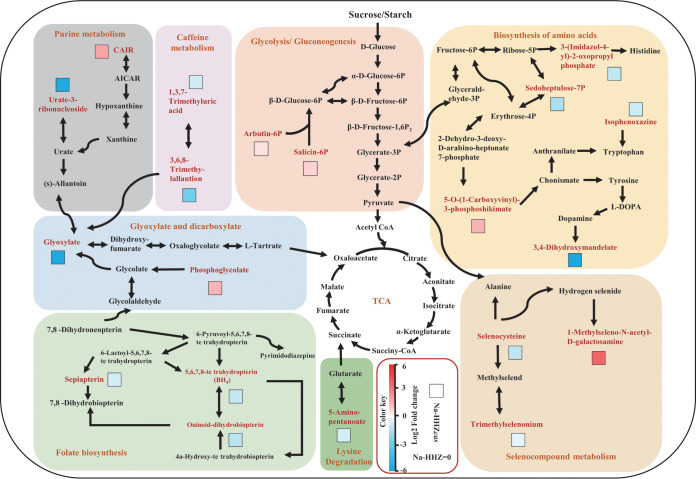
Screening for maps of metabolic pathways involved in key differentially expressed metabolites. The log_2_ fold change (HHZ versus HHZ*cas*) of each metabolite is displayed in the form of a heat map from low (blue) to high (red) as presented in the color scale. The box indicates the HHZ*cas* plants treated with 150 mM NaCl.

In order to investigate the underlying mechanism of the *SST* gene in altering the rice rhizosphere microbiome, we analyzed the soil metabolites of HHZ and transgenic (T_2_) plants (HHZ*cas*) under salt stress by liquid chromatography-tandem mass spectrometry (LC-MS/MS). A total of 4,397 peaks with names were detected in these two materials (see Table S3 in the supplemental material). The orthogonal partial least-squares discrimination analysis (OPLS-DA) demonstrated a clear separation between HHZ and HHZ*cas* plants under salt stress (Fig. 4). We obtained 135 differentially expressed soil metabolites between two rice materials (variable importance in projection [VIP] > 1.0, *P* < 0.05) by the combining and filtering procedures. Among them, 31 were upregulated, whereas 105 were downregulated under salt conditions (see Table S4 in the supplemental material). A major category of the differentially expressed metabolites enriched in the biosynthesis of secondary metabolites was identified by KEGG pathway enrichment analysis, including glycolysis/gluconeogenesis, purine metabolism, glyoxylate and dicarboxylate, folate biosynthesis, biosynthesis of amino acids, caffeine metabolism, and seleno compound metabolism (Fig. 5). Compared with HHZ plants, salicin, arbutin 6-phosphate, CAIR, phosphoglycolate, 5-O-3-phosphoshikimate, and 1-methlseleno-*N*-acetyl-d-galactosamine were upregulated in HHZ*cas* plants (Fig. 5). It is noteworthy that salicin and arbutin 6-phosphate involvement in the glycolysis/gluconeogenesis metabolite pathway showed upregulation in the HHZ*cas* lines.

10.1128/mSystems.00721-20.7 TABLE S3 Peaks with names were detected in Na-HHZ and Na-HHZ*cas* plants. Download Table S3, PDF file, 0.9 MB. Copyright © 2020 Lian et al. 2020 Lian et al. This content is distributed under the terms of the Creative Commons Attribution 4.0 International license.

10.1128/mSystems.00721-20.8 TABLE S4 Differentially expressed metabolites between Na-HHZ and Na-HHZ*cas* plants. Download Table S4, PDF file, 0.1 MB. Copyright © 2020 Lian et al. 2020 Lian et al. This content is distributed under the terms of the Creative Commons Attribution 4.0 International license.

### Correlation between microbial communities and soil metabolites.

**FIG 6:**
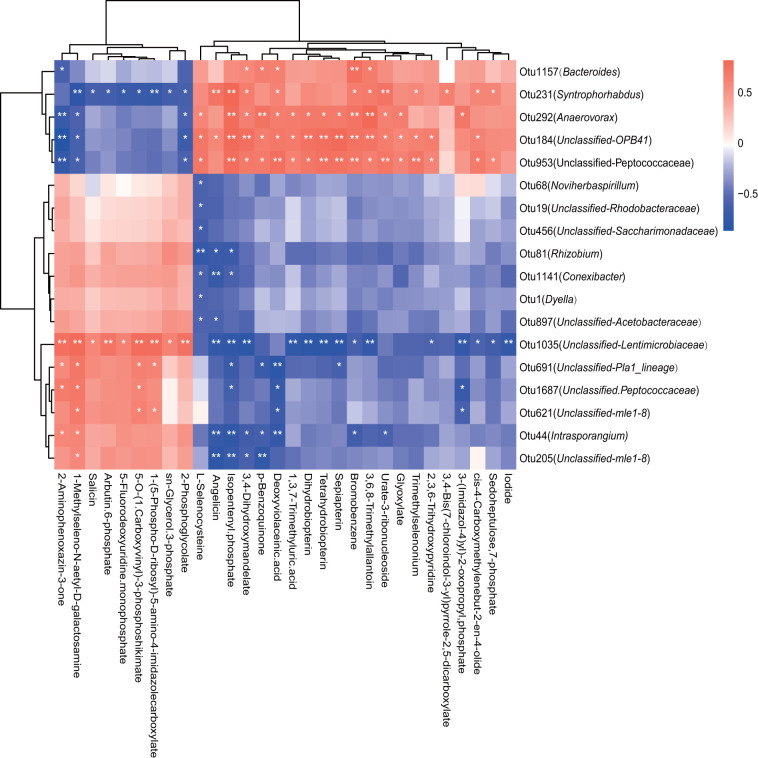
Correlation analysis between microbes and soil metabolites with significant differences between HHZ and HHZ*cas* plants under the salinity condition. Red boxes represent positive correlations, while blue boxes represent negative correlations (Pearson's correlation, *n* = 6, *P* < 0.05). White asterisks indicate statistical significance: *, *P* < 0.05; **, *P* < 0.01.

Correlations between rhizosphere soil microbiota and metabolites with significant differences between Na-HHZ and Na-HHZ*cas* were obtained via Pearson’s correlation analysis (Fig. 6). OTU1157 (*Bacteroides*), OTU231 (*Sytrophorhabdus*), and OTU292 (*Anaerovarax*) were positively correlated with sedoheptulose, 7-phosphate, glyoxylate, and bromobenzene and negatively correlated with arbutin, 6-phosphate, and salicin, while other OTUs, such as OTU1 (*Dyella*) and OTU81 (*Rhizobium*), showed the opposite trend (Fig. 6). Furthermore, we found that the relative abundances of OTU1 (*Dyella*), OTU19, (Unclassified-*Rhodobacteraceae*), and OTU81 (*Rhizobium*) had a significantly negative correlation with l-selenocysteine, while OTU19 (Unclassified-*Rhodobacteraceae*) was also correlated with angelicin and isopentenyl phosphate (*P* < 0.05) (Fig. 6).

## DISCUSSION

Using three pairs of plants with *SST* genes and loss of function of the *SST* gene in rice plants, we revealed the key role of the *SST* gene in shaping soil metabolites, resulting in shifts in bacterial communities in the rhizosphere. In comparison with the pronounced differences between salt and nonsalt rhizosphere bacteria, the plant genotype-mediated dynamics in soil bacterial community composition were more subtle (Fig. 2B), indicating that salt addition affects the composition of the soil bacterial communities. Specifically, under the absence of salt, knockout of the *SST* gene via the CRISPR/Cas9 approach did not change the bacterial community structure, but the *sst* mutants changed (Fig. 2A and Table 1). This may be attributed to the fact that the CRISPR/Cas9 system is an accurate tool to edit the *SST* gene in rice plants, while the mutant (*sst*) obtained from R401 by radiation mutagenesis could change other genes in the rice genome. The microbiomes assembled in the rhizospheres of HHZ, ZH11, and WT plants displayed great differences compared with the HHZ*cas*, ZH11*cas*, and *sst* plants, respectively, indicating that *SST* gene variation affects rhizosphere bacterial community composition under salt stress conditions (Fig. 2B and Table 1).

When excess salt ions accumulate in plant cells, they adversely impact multiple physiological metabolisms within plant cells, as well as the accumulation of mineral elements, and further affect the growth and development of plants. If plants suffer from high salt stress, potassium uptake is inhibited (1, 51). The rhizosphere soil associated with loss of *SST* function in rice contains higher NO_3_^+^, Na^+^, and Cl^−^ concentrations and lower K^+^ concentrations compared with rice containing the *SST* gene, whereas the rice plants with loss of *SST* function contain higher K^+^ and lower Na^+^ than the rice containing the *SST* gene under salt stress (Fig. 1G and H; Table S1). This indicated that the *sst* gene reduces the accumulation of sodium and increases the accumulation of potassium in plants, which directly shows that *SST* negatively regulates rice tolerance to salt stress. Our results showed that, under salt stress, the concentrations of K^+^ and Na^+^ in *sst* plants were higher and lower, respectively, than those in WT, respectively. It has been reported that genes in the SPL family can affect the expression of sodium ion transporters and increase the expression of potassium transporters (52). Thus, we speculate that the *SST* gene has a potential role in regulating the transport of the Na/K ion. Moreover, the changes in these soil chemical factors played an important role in affecting rhizosphere microbiota (Fig. 2C).

OTU-level analysis showed that the relative abundances of OTUs such as OTU1 (*Dyella*), OTU17 (Unclassified-*Saccharimonadales*), OTU19 (Unclassified-*Rhodobacteraceae*), OTU9 (Unclassified-*Burkholderiaceae*), OTU81 (*Rhizobium*), OTU42 (*Thiomonas*), and OTU95 (Unclassified-*Saccharimonadaceae*) were coenhanced on the rhizosphere soils of the plants that had loss of function of the *SST* gene under salt stress (Fig. 3). Therefore, we speculate that these 8 OTUs were the specific microbial species that were greatly affected by the *SST* gene in rice plants. In other words, these microbial species might be recruited in the rhizosphere after the loss of function of the *SST* gene in rice and were likely to help rice resistant to high salt stress. Among them, OTU1 (*Dyella*) has the highest relative abundance, and a previous study has reported that the genus *Dyella* was enriched in the rhizosphere of metal ion-tolerant soybeans, while there are few reports on the function of this bacterium (53). The most strongly promoted species of the plants without the *SST* gene are species that promote plant growth and facilitate phosphorus uptake in plants (*Burkholderiaceae*) or have a role in nitrogen cycling in soil (“*Candidatus* Rhizobium”) (54–56). Also, two genes (i.e., *amoA* and *hao*), that can encode key enzymes (ammonia monooxygenase and hydroxylamine oxidoreductase) for nitrification were detected in the *Rhodobacteraceae* (57). This is in line with our results that there was more NO_3_^−^ in the *sst*-expressed rhizospheres. Moreover, some species affiliated with *Rhodobacteraceae* could produce IAA, which may improve rice resistance against salt stress.

Plant genotypes can influence root exudation, which subsequently has a great effect on the rhizosphere microbiota (58–60). These root exudates could affect the growth of different bacteria presented in the rhizosphere (61). The OPLS-DA loading plot of soil metabolites showed that under the salt conditions, the HHZ groups were clearly differentiated from the control group in the *sst* group along principal coordinate 1 (PC1) (Fig. 4). This indicates that the *sst* gene plays an essential role in regulating the soil metabolite profile. Several metabolites, including benzoxazines, salicin, arbutin 6-phosphate, *sn*-glycerol 3-phosphate, 2-phosphoglycolate, phosphoshikimate, galactosamine, and 5-fluorodeoxyuridine monophosphate, were found to be significantly (*P* < 0.05) increased in the *sst* group (Fig. 5; Table S4). These significantly regulated metabolites belong to phenolics and fatty acids, aromatic acids, amino acids, and amides, all of which could regulate the growth of both microbiota and plants (62). For example, benzoxazinoids have been proven to improve plant resistance to microbial threats (63). Moreover, it was observed that rhizosphere microorganisms showed an affinity preference for the aromatic organic acids of nicotinic acid, salicylic acid, and cinnamic acid, which are secreted by the root (64). Because these metabolites can be used as sources of carbon and energy for the microbial community, the altered soil metabolism is likely to affect the soil’s microbial community composition (65). Selecting the specific compounds may be part of a strategy of the *sst* genotype rice to cope with salt stress. However, as there is a lack of data on HHZ or HHZ*cas* metabolite profiles in the absence of salt stress, whether the *SST* gene regulates the soil metabolite-mediated response to salinity needs to be further explored.

The relative abundances of rhizosphere bacteria at the OTU level were found to be closely associated with the concentrations of the specific rhizosphere soil metabolites. The members of OTU1 (*Dyella*), OTU81 (*Rhizobium*), and OTU19 (Unclassified-*Rhodobacteraceae*) were negatively correlated with l-selenocysteine, and OTU19 members were also correlated with angelicin and isopentenyl phosphate. l-Selenocysteine is one of the biological function forms of selenium in the plants and is volatile (66). Selenium is a vital micronutrient that is required to maintain homeostasis of several tissues, and there is a complex interaction mechanism between selenium and microbiota (66). Some bacterial species take advantage of the existence of selenium in their surrounding environments, and in some microbiomes, bacteria and host immune cells may compete for an inadequate supply of selenium. A previous study revealed that the volatilization of plant selenium is related to the plant rhizosphere microorganisms (67), but its mechanism is still unclear, so the influence of the volatilization of plant selenium on the interaction between plants and soil microorganisms is one of the topics to which should be paid special attention and which should be developed by in-depth study in the future. Angelicin is a specific group of secondary metabolites that is commonly present in higher plants, and previous studies in Heracleum sosnowskyi showed that angelicin is the principal allelochemical in fruits. Interestingly, angelicin displayed the highest antibacterial activity and might have ecological significance for the interaction between plants and other living organisms (68). Plant genomes encode isopentenyl phosphate kinases (IPKs) that reactivate isopentenyl phosphate (IP) via ATP-dependent phosphorylation, forming the primary metabolite isopentenyl diphosphate (IPP), used generally for isoprenoid/terpenoid biosynthesis (69). Terpenoid metabolites play a variety of basic functions in plant growth and development. In addition, the ecological importance of terpenoids has received increasing attention for developing strategies for plants to resist biotic and abiotic stresses (70).

We must point out that the changes in soil metabolites observed in this study are not equivalent to the results for the metabolites of root exudates; the contribution of native soil microbial communities to metabolites cannot be ignored. The root exudates regulated by the *SST* gene are likely to affect the metabolic activity of soil microorganisms and thereby trigger the up- or downregulation of extracellular metabolites (64). Many root exudate metabolites have been reported to affect the composition of soil microbial communities (64). Therefore, the altered soil metabolite profile may be partially due to passive extracellular compounds released by microorganisms. However, the contribution of root exudate metabolites to the altered rhizosphere bacterial structures still needs to be further explored in the future.

**FIG 7:**
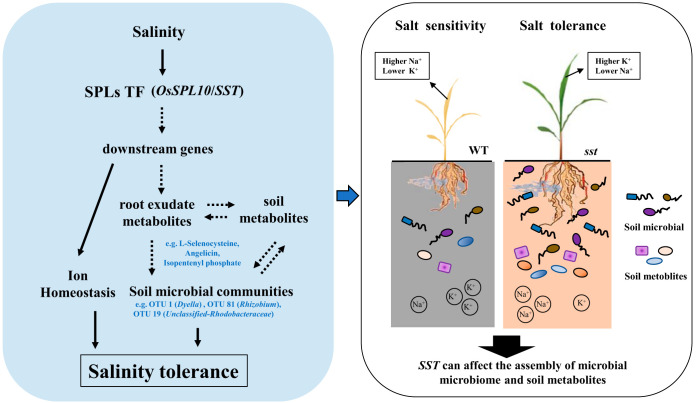
Schematic representation of how the *SST* gene shapes the rhizosphere bacterial community by conferring tolerance to salt stress through regulation of soil metabolites.

In conclusion, we find that the loss of function of *SST* can affect the assembly of the soil microbiome and soil metabolites. In more detail, some microbial species, such as OTU1 (*Dyella*), OTU81 (*Rhizobium*), and OTU42 (*Thiomonas*), were enriched in the rhizospheres of the rice plants that contain the *SST* gene. The mutation of the *SST* gene increases the accumulation of nitrate nitrogen and reduces the accumulation of sodium and chloride ions in rice. This not only can alleviate salt stress in rice, but also can change the rhizosphere environment and subsequently affects the rhizosphere microbiome. In addition, some soil metabolites, such as l-selenocysteine and angelicin, were related to the change of rhizosphere microbial communities. This research focused on how the *SST* gene related to salt tolerance regulates soil metabolites and rhizosphere microorganisms (Fig. 7). However, to what extent these enriched bacterial genera have an impact on rice resistance to salt stress is not yet known and is the subject of future work. Moreover, more soil types should be considered to explore general mechanisms by which the *SST* gene regulates the rice rhizobacteria. Overall, our findings not only provide a useful paradigm for revealing the roles of key genes of plants in shaping rhizosphere microbiomes and the relationship with soil metabolites, but also offer new insights into the strategies to enhance rice tolerance to high salt levels from microbial and ecological perspectives.

## MATERIALS AND METHODS

### Plant materials and soil sampling.

Two *O. sativa* subsp. *indica* rice cultivars, Huanghuazhan (HHZ) and R401 (WT), and an *O. sativa* subsp. *japonica* rice cultivar, Zhonghua 11 (ZH11), were used in this study. The salt-tolerant mutant (*sst*) was developed by mutagenesis through radiation from R401 (71–73), and *SST* (*LOC_Os06g44860*, *Os06g0659100*) knockout mutant plants were generated from HHZ and ZH11 (2) (Fig. S1). Positive transgenic (T_2_) plants (HHZ*cas* and ZH11*cas*) were also used in the study. The soil was collected from a field in Suixi County (110°25′N, 21°32′E), Guangdong Province, China, in July 2018 and classified as Ali-Udic Argosol. The chemical properties of the soil are listed in Table S1 in the supplemental material.

### Experimental design.

Before the experiment, the soil was sieved with a 4-mm-pore mesh size. The pot experiment was carried out under controlled conditions at South China Agricultural University in 2019. Rice seeds were grown on plastic trays (150-mm height by 200-mm width by 250-mm length). Twenty seeds of equal size were germinated and subsequently removed to obtain six plants per pot. There were three pots for each treatment, and two plants and rhizosphere soil replicates were collected from one pot. The type of soil management complied with conventional agronomic management practices for rice. Sodium chloride (NaCl) was used as the salt source. NaCl solution (150 mM) or pure water was used in each pot. The treatment procedure for rice seedlings was 7 days of treatment with NaCl, 3 days of treatment with water, and then 7 days of treatment with NaCl (see Fig. S4 in the supplemental material). A total of 72 samples (6 rice genotypes × 6 replicates × 2 NaCl treatments) were collected in this study. The rhizosphere soils of each treatment were sampled at the seedling stage on 20 June, 20 days after seeding. For the rhizosphere soil sample collection, the attached soil was removed by gentle shaking, and then the soil attached to the root was transported to a big beaker filled with 50 ml of phosphate-buffered saline. Fifteen grams of rhizosphere soils was collected by centrifugation, removed from phosphate-buffered saline from each sample, and then stored at −80°C for total DNA extraction and LC-MS analysis. The remaining rhizosphere soil samples were stored at 4°C for measurement of chemical properties.

10.1128/mSystems.00721-20.4 FIG S4 Schematic diagram of rice seedling treatment. (A) The treatment procedure for rice seedlings. (B) The experimental design for each rice material under salt or control treatment. Download FIG S4, PDF file, 0.06 MB. Copyright © 2020 Lian et al. 2020 Lian et al. This content is distributed under the terms of the Creative Commons Attribution 4.0 International license.

### Rhizosphere soil and plant properties.

The soil total carbon (TC) and total nitrogen (TN) were measured in an Elemental Analyser (Vario EL, Hanau, Germany). Soil total potassium (TK) was determined on an ICPS-7500 (Shimadzu, Kyoto, Japan). Soil total phosphorus (TP), nitrate (NO_3_^−^-N), and ammonium (NH_4_^+^-N) were assayed by a continuous-flow analytical system (Skalar, Breda, Netherlands) as previously described (74). The Na^+^ concentration in the soil and Na^+^ and K^+^ concentrations in the plants were measured by atomic absorption spectrometry (PerkinElmer Analyst 700; PerkinElmer, Norwalk, CT, USA). Concentrations of the Cl^−^ anions in the soil were measured by an ion chromatography apparatus (ICS-3000; Dionex, Sunnyvale, CA, USA). The molybdenum-antimony colorimetric method was used to analyze the soil available phosphorus (AP), and a pH meter was used to measure the soil pH.

### Metabolite measurement.

Fifty milligrams of samples was added into the extracted solvent (acetonitrile-methanol-water at 2:2:1, containing the internal standard), and then the samples were vortexed, homogenized, and sonicated in an ice-water bath. The homogenate and sonicate circle were repeated for 3 times, followed by incubation and centrifugation. The resulting supernatants were transferred to LC-MS vials and stored at −80°C until ultrahigh-performance liquid chromatography (UHPLC)-Q Exactive (QE) Orbitrap MS analysis. The quality control (QC) sample was prepared by mixing equal aliquots of the supernatants from all of the samples. Liquid chromatography-tandem mass spectroscopy (LC-MS/MS) was used to detect the soil metabolites. (Guangzhou Genedenovo Biotechnology Co., Ltd., assisted with MS analysis.)

### DNA extraction, gene amplification, and Illumina sequencing.

Based on the manufacturer’s instructions, a Fast DNA Spin kit for Soil (MP Biomedicals, Santa Ana, CA, USA) was used to extract the DNA of rhizosphere soil. Primers 515F (5′-GTGCCAGCMGCCGCGGTAA-3′) and 907R (5′-CCGTCAATTCMTTTRAGTTT-3′) with 8-nucleotide (nt) unique barcodes at the 5′ end were used to amplify the V4 hypervariable region of the 16S ribosomal DNA (rDNA) gene (75). Then an equal amount of product from PCR amplification was pooled and paired-end sequenced on an Illumina Hiseq2500 PE250 platform according to standard protocols.

### Bioinformatics.

The raw sequence data were processed in QIIME1.19.1. Briefly, sequences with low quality, which were identified as a length of <200 bp and an average base quality score of <20, were removed. The UCHIME algorithm was used to detect and remove the potentially chimeric sequences (76). Operational taxonomic units (OTUs) were clustered at 97% similarity in the CD-HIT program. The OTUs were phylogenetically assigned using the RDP naive Bayesian classifier, against the SILVA database (77). Moreover, the Shannon’s diversity indexes were also calculated in QIIME1.19.1.

### Statistical analyses.

Principal-coordinate analysis (PCoA) based on Bray-Curtis dissimilarities was conducted in R (version 3.5) using the “Ape” package (78). Nonparametric permutational multivariate analysis of variance (PERMANOVA), canonical correspondence analysis (CCA), and the Mantel test were conducted in R using the “vegan” package (79). Different relative abundant OTUs of the three pairs of plant materials were determined in R, respectively, using the DESeq2 package based on a *P* value of <0.05 (with a false-discovery rat [FDR] of <5% under the Benjamini-Hochberg correction) (DESeq2, *n* = 6, *P* < 0.05). Additionally, a Pearson bivariate correlation analysis was performed to access the correlations between microbes and soil metabolites with significant differences between HHZ and HHZ*cas* plants under the salinity condition. To rank the best-distinguished metabolites between two groups, a variable importance in projection (VIP) score of the OPLS model was used, and the threshold of VIP was set to ≥1 (OPLS, *n* = 6, VIP ≥ 1). Moreover, the *t* test was applied to screen metabolites, and those with a *P* value of ≤0.05 were considered as differentially expressed metabolites between two groups (*t* test, *n* = 6, *P* < 0.05). In addition, the differences between Na^+^ and K^+^ concentrations in plants under different treatments were assessed using the *t* test (*n* = 3, *P* < 0.05). Differences in soil chemical properties were evaluated using Genstat (version 13.0) with two-way analysis of variance (two-way ANOVA, *n* = 6, *P* < 0.05). Determination of the least significant difference (LSD) based on a *P* value of <0.05 was performed in GenStat 13 (VSN International, Hemel Hempstead, United Kingdom) to assess difference in soil chemical properties (LSD, *n* = 6, *P* < 0.05) (80).

### Data availability.

All the raw sequence data for the rhizosphere bacterial community have been deposited in the National Center for Biotechnology Information (NCBI) Sequence Read Archive under accession no. PRJNA642350. The raw metabolite data for the soil metabolites can be found in Table S3 in the supplemental material.
