# Supplementary figures and images for: Rice SST Variation Shapes the Rhizosphere Bacterial Community, Conferring Tolerance to Salt Stress through Regulating Soil Metabolites
Source: mSystems. 2020 Nov 24;5(6):e00721-20. doi: 10.1128/mSystems.00721-20 (PMC7687028; doi:10.1128/mSystems.00721-20)

Figure S1

**SST OsSPL10-SBP-box gene family member**      *LOC\_Os06g44860 (Os06g0659100)*

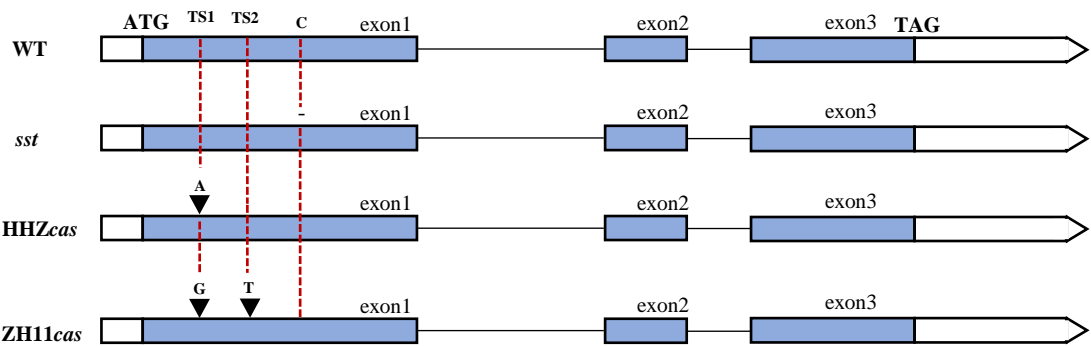

Supplement: FIG S1 [file mSystems.00721-20-sf001.pdf]

Figure S2.

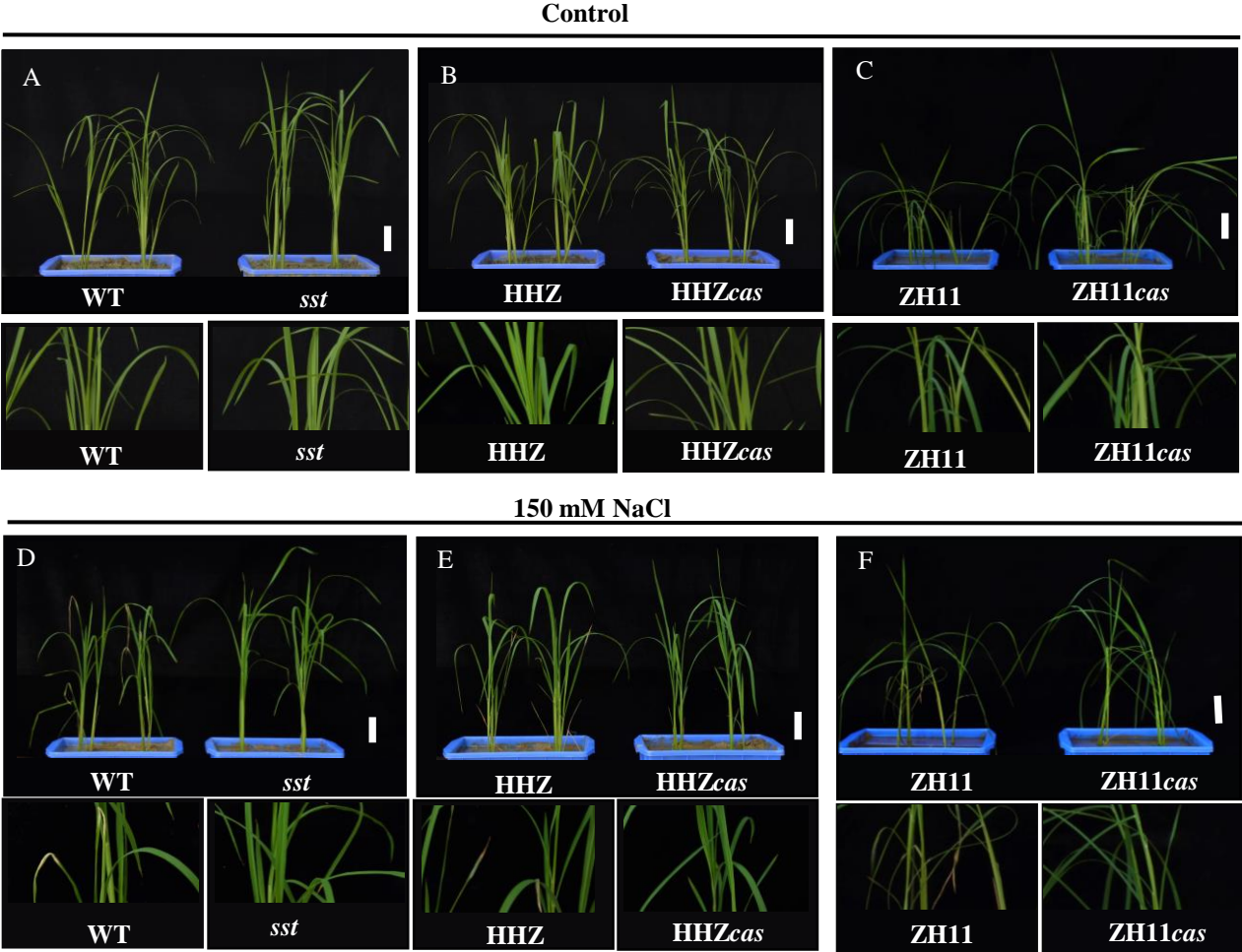

Supplement: FIG S2 [file mSystems.00721-20-sf002.pdf]

Figure S3.

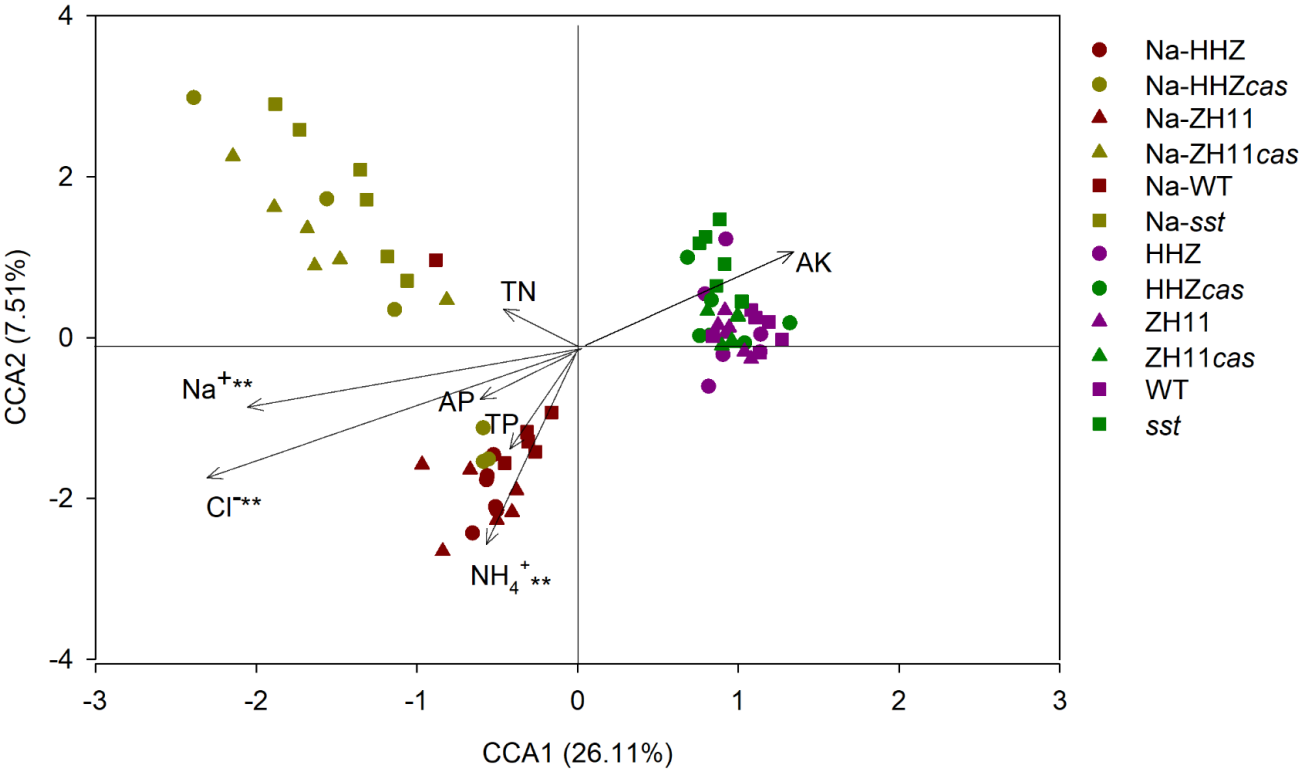

Supplement: FIG S3 [file mSystems.00721-20-sf003.pdf]

Figure S4.

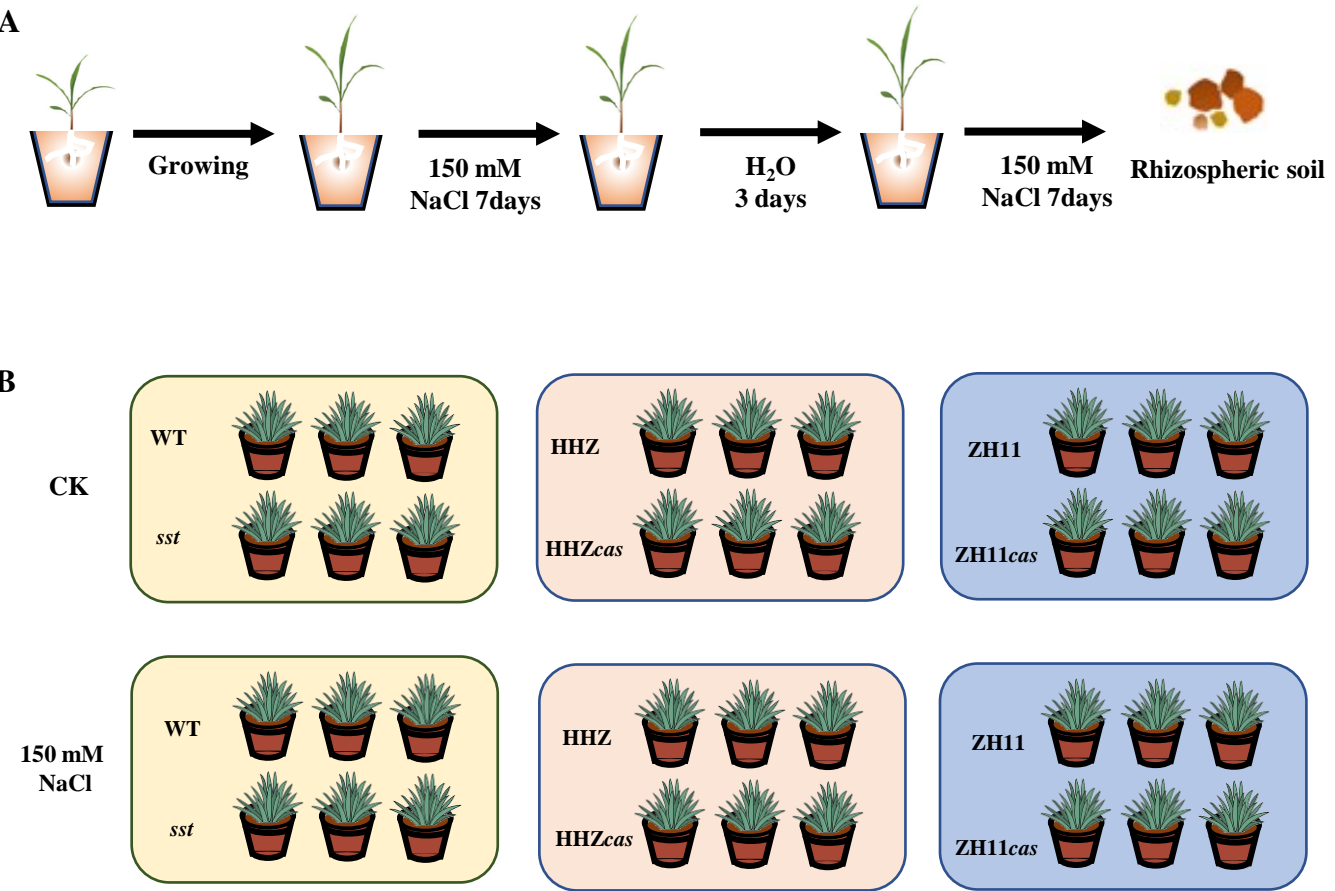

Supplement: FIG S4 [file mSystems.00721-20-sf004.pdf]
