# Supplementary material for: Rice SST Variation Shapes the Rhizosphere Bacterial Community, Conferring Tolerance to Salt Stress through Regulating Soil Metabolites
Source: mSystems. 2020 Nov 24;5(6):e00721-20. doi: 10.1128/mSystems.00721-20 (PMC7687028; doi:10.1128/mSystems.00721-20)
Supplement: TABLE S1 [file mSystems.00721-20-st001.pdf]

Table S1 Effects of NaCl treatment and different rice genotypes on soil physicochemical properties.

Treatments	pH	SOC (g/kg)	Total Nitrogen (g/kg)	Total phosphorus (g/kg)	Available phosphorus (mg/kg)	Available potassium (mg/kg)	NH_4^+ (mg/kg)	NO_3^- (mg/kg)	Na^+ (mg/kg)	Cl^- (mg/kg)
WT	5.92 ± 0.08	29.53 ± 0.1[illegible]	1.49 ± 0.01	0.59 ± 0.001	12.92 ± 0.11	35.32 ± 0.14	8.8 ± 0.52	1.21 ± 0.3	16.12 ± 0.08	14.89 ± 0.88
sst	6.03 ± 0.05	29.53 ± 0.1	1.45 ± 0.02	0.58 ± 0.002	11.72 ± 0.09	34.62 ± 0.21	6.21 ± 0.52	5.35 ± 0.3	12.53 ± 0.23	12.26 ± 0.88
HHZ	5.97 ± 0.06	28.82 ± 0.07	1.45 ± 0.01	0.58 ± 0.001	11.31 ± 0.14	33.39 ± 0.32	7.77 ± 0.52	4.49 ± 0.3	17.4 ± 0.27	11.68 ± 0.51
HHZ*cas*	6.07 ± 0.06	29.7 ± 0.11	1.36 ± 0.01	0.57 ± 0.01	10.49 ± 0.19	48.4 ± 0.37	6.04 ± 0.3	1.55 ± 0	15.39 ± 0.19	20.73 ± 1.01
ZH11	6.05 ± 0.02	30.19 ± 0.25	1.55 ± 0.02	0.59 ± 0	8.59 ± 0.19	56.77 ± 0.19	8.8 ± 0.52	1.04 ± 0	13.32 ± 0.14	17.52 ± 0
ZH11*cas*	5.97 ± 0.05	29.16 ± 0.18	1.5 ± 0	0.55 ± 0	11.28 ± 0.14	46.28 ± 0.21	6.39 ± 0.3	2 ± 0.12	14.37 ± 0.08	14.89 ± 0.88
Na-WT	5.69 ± 0.03	29.25 ± 0.23	1.5 ± 0.01	0.55 ± 0.01	15.16 ± 0.24	74.47 ± 0.68	19.85 ± 0.3	0.64 ± 0.11	1509.34 ± 3.9[illegible]	2744.8 ± 13.38
Na-*sst*	5.5 ± 0.03	29.37 ± 0.11	1.41 ± 0.01	0.58 ± 0	15.29 ± 0.14	55.76 ± 0.35	8.8 ± 0.52	1.43 ± 0.16	[illegible]846.74 ± 17.0[illegible]	3369.68 ± 13.38
Na-HHZ	5.69 ± 0.04	28.37 ± 0.35	1.56 ± 0.01	0.57 ± 0	11.75 ± 0.14	84.79 ± 0.47	23.82 ± 0.52	0.62 ± 0.1	1581.24 ± 3.2[illegible]	2838.24 ± 17.52
Na-HHZ*cas*	5.63 ± 0.02	30.4 ± 0.08	1.62 ± 0.02	0.58 ± 0	11.43 ± 0.09	73.9 ± 0.15	13.46 ± 0	3.83 ± 0.1	1703.77 ± 4.8[illegible]	3089.36 ± 26.76
Na-ZH11	5.56 ± 0.01	29.91 ± 0.09	1.53 ± 0.01	0.55 ± 0.01	13.77 ± 0.14	80.58 ± 0.38	25.03 ± 0.3	2.17 ± 0.1	1481.44 ± 9.4[illegible]	2709.76 ± 10.12
Na-ZH11*cas*	5.63 ± 0.04	30.29 ± 0.19	1.55 ± 0.04	0.57 ± 0	15.19 ± 0.14	74.55 ± 0.3	16.22 ± 0.3	2.49 ± 0.63	1809.67 ± 1.9[illegible]	3206.16 ± 23.18
NaCl	<0.001	0.548	0.006	0.002	<0.001	<0.001	<0.001	0.124	<0.001	<0.001
sst	0.671	0.036	0.124	0.655	0.54	0.063	<0.001	0.028	<0.001	<0.001
NaCl**sst*	0.029	0.019	0.212	<0.001	0.85	0.023	<0.001	0.453	<0.001	<0.001
LSD	0.0671	0.5235	0.0614	0.00865	1.502	7.94	1.972	1.441	37.89	65.7
