# Supplementary material for: Rice SST Variation Shapes the Rhizosphere Bacterial Community, Conferring Tolerance to Salt Stress through Regulating Soil Metabolites
Source: mSystems. 2020 Nov 24;5(6):e00721-20. doi: 10.1128/mSystems.00721-20 (PMC7687028; doi:10.1128/mSystems.00721-20)
Supplement: TABLE S2 [file mSystems.00721-20-st002.pdf]

TableS2 Differentially OTUs in the three pairs plants under the no-salt and salt conditions. (DESeq2; n=6; p < 0.05)

HHZ vs HHZ*cas*

OTU ID	baseMean	log2FoldChange	pvalue	padj
Otu000228	86.34682329	4.489564001	1.41E-06	0.0109568

ZH11 vs ZH11*cas*

OTU ID	baseMean	log2FoldChange	pvalue	padj
Otu000228	168.2888572	4.480607396	2.50E-13	4.93E-10
Otu000001	28.08666715	2.632580949	5.68E-08	5.59E-05
Otu000011	682.8784707	2.934622275	9.72E-07	0.00063814
Otu000113	5.333197263	4.918387925	0.00011294	0.04449701
Otu000711	15.05168491	-1.562823339	0.00010839	0.04449701

Na-HHZ vs Na-HHZ*cas*

OTU ID	baseMean	log2FoldChange	pvalue	padj
Otu000019	655.2612942	-2.421000782	9.86E-05	0.02073179
Otu000009	850.3921576	-3.707261133	1.24E-05	0.02073179
Otu000081	187.9311816	-2.550427224	0.00010292	0.02073179
Otu000044	166.7250734	-1.485538532	0.00011054	0.02073179
Otu000742	42.91215511	-3.893371487	3.81E-05	0.02073179
Otu001033	15.62816396	-4.875959533	8.72E-05	0.02073179
Otu000704	15.96149223	-2.773737192	7.56E-05	0.02073179
Otu000184	53.2856109	1.375600022	6.67E-05	0.02073179
Otu001687	5.225814134	-3.203644297	9.93E-05	0.02073179
Otu000042	318.8501674	-3.584738342	0.00013627	0.02300182
Otu000001	5457.201155	-2.181464686	0.00017232	0.02553723
Otu001035	11.50590782	-1.435002516	0.00018154	0.02553723
Otu001278	10.04591946	-3.336888379	0.00019796	0.02570439
Otu001970	7.334076777	-3.951452173	0.00021507	0.02593145
Otu000897	9.190385821	-3.068991804	0.00034875	0.03679333
Otu000953	17.12956933	2.11740117	0.00033771	0.03679333
Otu000068	215.6522464	-3.132662051	0.00044412	0.03928174
Otu000456	31.91440812	-2.693683358	0.00046542	0.03928174
Otu000205	51.29043897	-1.217412857	0.00039988	0.03928174
Otu000621	13.48896495	-1.309376903	0.00044642	0.03928174
Otu000829	27.13099229	-4.876862037	0.0005682	0.04248727
Otu000638	15.9715787	-2.658115547	0.0005355	0.04248727
Otu001141	7.418351366	-2.585478971	0.00062926	0.04248727
Otu000292	7.836004247	2.482542639	0.00061987	0.04248727
Otu001157	11.76524492	4.179251126	0.00062601	0.04248727
Otu000231	43.95380215	0.787343292	0.00076442	0.04837016
Otu000691	9.681938962	-1.542423181	0.00077369	0.04837016

Na-ZH11 vs Na-ZH11*cas*

OTU ID	baseMean	log2FoldChange	pvalue	padj
Otu000006	639.1118817	-3.616899779	6.20E-14	1.51E-10
Otu000096	39.13862752	-4.463984136	4.37E-09	5.31E-06
Otu000105	27.05310063	-4.220137629	4.37E-08	3.54E-05
Otu000618	7.204868783	-3.211784111	9.87E-08	6.00E-05
Otu000028	163.6485008	-3.235456073	2.77E-07	0.00013485
Otu000499	70.34454558	-2.781868865	6.87E-07	0.00023847
Otu003493	9.438131493	-6.448590634	6.77E-07	0.00023847

WT vs *sst*

OTU ID	baseMean	log2FoldChange	pvalue	padj
Otu000013	479.7065101	-0.88291506	9.04E-12	2.51E-08
Otu000351	70.87509494	-2.93471227	1.25E-10	1.73E-07
Otu000107	87.25843832	-1.49057002	5.76E-10	5.32E-07
Otu000084	240.6798361	-2.29404607	2.76E-09	1.91E-06
Otu000580	47.23932788	-3.77228844	3.79E-09	2.10E-06
Otu000092	133.5850767	-1.48023413	8.33E-09	3.85E-06
Otu000017	32.67571666	-4.10061275	1.06E-08	4.20E-06
Otu000129	198.0331765	-2.66083962	2.45E-08	8.48E-06
Otu000003	1702.47851	3.35112384	3.12E-08	8.64E-06
Otu000247	128.1366257	-4.34267159	3.43E-08	8.64E-06
Otu000586	36.07610361	-3.38424683	3.43E-08	8.64E-06
Otu000138	110.3683749	-1.3247038	8.59E-08	1.83E-05
Otu000647	34.1936344	-3.25594818	8.50E-08	1.83E-05
Otu000027	314.1971444	-1.16667385	1.04E-07	2.06E-05
Otu001704	17.70355759	-6.15684185	1.27E-07	2.35E-05
Otu000674	33.59773919	-3.75036953	1.42E-07	2.46E-05
Otu000009	345.2919074	-2.59906191	1.92E-07	3.14E-05
Otu000035	152.9411055	1.26438169	2.09E-07	3.22E-05
Otu000683	66.81777938	-8.08283254	2.44E-07	3.57E-05
Otu000028	250.5884526	-1.34599859	2.78E-07	3.67E-05
Otu000573	28.04927177	-1.8705041	2.77E-07	3.67E-05
Otu001387	13.61986918	-5.7957076	3.01E-07	3.79E-05
Otu000007	142.0857029	1.51293604	3.23E-07	3.90E-05
Otu000697	35.27466149	-2.81567427	4.47E-07	5.16E-05
Otu001643	10.01006067	-4.27484756	7.58E-07	8.40E-05
Otu000194	32.94051518	1.22914239	1.04E-06	0.000106366
Otu000564	46.57700987	-3.4089754	1.02E-06	0.000106366
Otu000663	25.76623448	4.69236084	1.14E-06	0.000113199
Otu000258	40.14641892	-1.30421665	2.01E-06	0.000183306
Otu000421	38.18264027	-1.93588451	2.02E-06	0.000183306
Otu001094	20.795256	-5.17625226	2.05E-06	0.000183306
Otu000184	39.52505453	1.7805452	2.79E-06	0.000240664
Otu000188	117.1563399	-1.73879936	2.87E-06	0.000240664
Otu002198	5.450127368	-3.36514247	3.09E-06	0.000251552
Otu000748	29.4535924	-2.44060332	3.18E-06	0.000252239
Otu000846	38.18923813	-3.31228342	4.62E-06	0.000355878
Otu000037	320.6011808	-1.65809572	5.18E-06	0.000387713
Otu000304	40.08915681	-1.09313702	6.70E-06	0.000471964
Otu000461	41.54987639	-0.96273524	6.63E-06	0.000471964
Otu001832	8.59675771	-3.29317041	6.81E-06	0.000471964
Otu000303	55.52730849	-1.24530721	7.08E-06	0.000478353
Otu000030	213.6925239	-1.37249019	7.38E-06	0.000487203
Otu000187	86.35564771	-1.5231254	8.30E-06	0.000535343
Otu000305	38.169461	-1.23555389	9.70E-06	0.000611046
Otu000269	62.70560707	-0.81529802	1.02E-05	0.000629366
Otu000490	50.32655238	-2.5229762	1.12E-05	0.00067572
Otu000610	10.3755791	2.72703522	1.32E-05	0.000778264
Otu001105	12.90632178	-2.9333088	1.48E-05	0.000854803
Otu000199	28.03673706	1.34427144	1.53E-05	0.000866782

Otu000122	17.65640325	-4.340476724	8.20E-07	0.00024908
Otu000293	12.40518404	-2.863370446	1.13E-06	0.00028042
Otu003513	9.319831855	-6.436832605	1.15E-06	0.00028042
Otu000063	22.95093836	-4.96141971	1.30E-06	0.00028775
Otu000074	28.70886952	-5.015391085	3.15E-06	0.00058837
Otu000344	39.08974397	-3.426993474	2.97E-06	0.00058837
Otu000351	21.41862484	-3.46171414	3.44E-06	0.00059639
Otu000030	16.92005026	-3.285792444	4.58E-06	0.00074148
Otu000001	7631.519702	-2.211626107	5.90E-06	0.0008515
Otu000020	188.5525363	-0.722320768	6.19E-06	0.0008515
Otu000183	65.07770005	-1.395885606	6.31E-06	0.0008515
Otu000151	31.52763443	-4.760700173	6.85E-06	0.00087578
Otu000352	45.90579814	-1.631418525	7.75E-06	0.0009417
Otu000139	97.52697742	-2.528626956	1.79E-05	0.00207421
Otu000135	19.30837317	-3.278153025	2.39E-05	0.00252231
Otu003260	8.329272259	-3.203226339	2.38E-05	0.00252231
Otu004337	3.819960455	-5.622414357	2.89E-05	0.00292997
Otu000023	111.5703288	2.740300777	3.83E-05	0.00372629
Otu000002	853.3931207	-2.741434382	4.01E-05	0.00374629
Otu000225	23.39465409	-2.294870229	4.73E-05	0.00426099
Otu000807	27.63263184	2.230865578	6.44E-05	0.00559025
Otu000095	29.85537546	-1.932425431	6.80E-05	0.00569467
Otu000005	948.5407083	-2.051569487	9.09E-05	0.00735914
Otu000186	44.14794208	-2.741410998	0.0001064	0.00834043
Otu000003	804.4743859	-1.74630854	0.00013795	0.01046652
Otu000080	108.4165405	-1.087824044	0.00014214	0.01046652
Otu000929	8.485333391	-2.668944244	0.00014657	0.01047549
Otu000046	87.83347092	-1.431027958	0.00019093	0.01325564
Otu000029	569.4857636	-2.880335359	0.00020358	0.01351209
Otu000043	8.536668947	-3.387220745	0.00020574	0.01351209
Otu000606	14.43276784	-2.294193823	0.00022257	0.01423283
Otu000205	55.23593471	2.874652535	0.00023971	0.01479531
Otu003253	9.700646002	-4.151225942	0.00024354	0.01479531
Otu000261	25.91597068	-1.203392068	0.00025146	0.0149036
Otu000051	190.7166311	-1.565730928	0.00025939	0.01500746
Otu000219	2.898583482	-4.225239514	0.00029324	0.01657142
Otu000985	19.97436263	-5.545191021	0.0004131	0.02281433
Otu000014	605.1586876	-2.825538323	0.00044985	0.024292
Otu000059	118.6507464	-0.610617744	0.0005787	0.03057039
Otu001587	9.021877518	-3.638949086	0.00063065	0.03260594
Otu000017	646.1049715	-1.443513604	0.00066645	0.03371475
Otu000792	8.09463097	-2.57739733	0.00067985	0.03371475
Otu004483	1.329858921	-4.108012048	0.00073402	0.03567343
Otu001525	4.118802095	-3.091731181	0.00076272	0.03634114
Otu000415	14.66987281	-1.620765417	0.00085041	0.03974034
Otu000264	9.418649817	-2.706474604	0.00104621	0.04539816
Otu000353	46.26881069	-1.205487097	0.00099148	0.04539816
Otu000407	19.43133369	-2.78067509	0.00102987	0.04539816
Otu001648	2.854097531	-4.164474278	0.00101273	0.04539816
Otu000045	122.2776757	-1.969868437	0.00108413	0.04542121
Otu000050	124.5059643	-1.897232161	0.00107831	0.04542121

Na-WT vs Na-*sst*

	baseMean	log2FoldChange	pvalue	padj
Otu000042	856.0732353	-5.510504892	5.72E-19	1.49E-15
Otu000356	40.55516085	-1.63122906	1.83E-05	0.000975995
Otu001302	19.96535533	-7.32038008	1.80E-05	0.000975995
Otu001842	8.94207278	-4.74803698	1.83E-05	0.000975995
Otu000207	14.51052777	2.35949812	1.97E-05	0.001024528
Otu001187	13.11236997	-2.3906681	2.00E-05	0.001024528
Otu000773	27.28676206	-2.63057355	2.09E-05	0.001054978
Otu003657	3.719313429	-4.86525782	2.15E-05	0.001065955
Otu000357	44.88843611	-1.94639714	2.33E-05	0.001130974
Otu000238	27.3209612	1.42585853	2.63E-05	0.001235428
Otu000262	52.0797948	-1.02720808	2.63E-05	0.001235428
Otu001553	8.811998307	-3.30407849	2.96E-05	0.001369632
Otu000022	454.9708655	-0.88703583	3.61E-05	0.001625009
Otu000135	328.3666323	4.37177288	3.69E-05	0.001625009
Otu000358	30.53911879	-1.25029352	3.65E-05	0.001625009
Otu003570	3.693501261	-5.36772129	3.80E-05	0.001647564
Otu000502	29.1696498	-1.81290386	3.89E-05	0.001657942
Otu000192	73.72840453	-1.9917839	4.22E-05	0.001773827
Otu003270	2.402289371	-4.22312316	4.40E-05	0.00182012
Otu000300	44.5379735	-1.29601641	4.73E-05	0.001908424
Otu000954	14.00178968	2.16566597	4.75E-05	0.001908424
Otu000698	20.3867005	-2.08521867	6.14E-05	0.002431403
Otu000887	7.248904647	-1.98837921	6.52E-05	0.00254413
Otu000006	888.2672827	0.59067197	6.93E-05	0.002630675
Otu001027	5.230034189	3.035437	6.85E-05	0.002630675
Otu001040	5.225190105	2.63894007	7.30E-05	0.002717442
Otu001607	5.289273015	-2.66003421	7.35E-05	0.002717442
Otu000482	37.79934336	-1.87068157	7.65E-05	0.00278989
Otu000420	35.30007093	-1.74877963	8.05E-05	0.002897474
Otu000699	21.42754773	-1.75109007	9.91E-05	0.00352257
Otu000390	41.89632374	-1.23892244	0.00010631	0.003683762
Otu001364	5.545793795	-2.3869039	0.00010586	0.003683762
Otu000128	63.89451251	1.12944229	0.00011295	0.003865456
Otu000116	67.68447805	1.29278961	0.00012173	0.004002685
Otu000253	54.31042273	-0.91808427	0.00012389	0.004002685
Otu000263	11.7096935	1.6743988	0.000119	0.004002685
Otu000267	58.92333824	-1.58933914	0.00012314	0.004002685
Otu000746	22.35872472	-1.73754214	0.00012418	0.004002685
Otu000589	57.98566163	-3.00103669	0.00012634	0.004025403
Otu001772	3.330256348	-4.19300196	0.00012938	0.004075377
Otu001971	11.35858359	6.50640864	0.00013198	0.00411076
Otu000484	12.35226579	1.50103569	0.00014342	0.004417204
Otu000229	83.65247817	-1.91619057	0.00014766	0.004484891
Otu000884	5.581898204	2.40486468	0.00014943	0.004484891
Otu001339	10.173453	-3.12583378	0.00015047	0.004484891
Otu000204	96.96777377	-1.00232015	0.00015592	0.004597943
Otu000324	27.13035544	0.88945625	0.00015916	0.004644254
Otu001189	16.0287214	-3.50232773	0.00017288	0.004991789
Otu000361	36.55487603	-0.95680414	0.00018997	0.005428772
Otu000871	8.806065783	1.70299061	0.00019692	0.005513808
Otu002649	3.322179107	-4.69911029	0.00019511	0.005513808
Otu000033	280.8118362	-0.9219466	0.00020716	0.005693584
Otu000657	13.47075536	1.4955286	0.00020745	0.005693584
Otu000060	39.96853596	1.60639749	0.00021389	0.005791358
Otu000593	25.25540066	-1.11833963	0.00021519	0.005791358
Otu001291	10.50809845	-2.5457199	0.0002244	0.005981015

Otu000558	83.05740775	-5.438734535	2.28E-12	2.96E-09
Otu000017	1053.687066	-4.275562331	3.02E-10	2.62E-07
Otu000223	213.4582161	-5.401560572	3.23E-09	2.10E-06
Otu000103	176.9183508	1.18378193	6.68E-09	3.48E-06
Otu000081	187.6920236	-2.97628389	1.06E-08	4.61E-06
Otu000444	34.94980735	-3.581427232	3.71E-08	1.38E-05
Otu000456	40.43845494	-3.330571812	5.53E-08	1.79E-05
Otu002172	11.33256134	-6.236338408	6.19E-08	1.79E-05
Otu000010	998.9109328	-2.590828618	1.40E-07	3.64E-05
Otu000992	20.41361835	-5.569496319	1.76E-07	4.17E-05
Otu000052	304.223973	-3.035116903	1.93E-07	4.18E-05
Otu000415	12.40041088	-2.472597268	2.93E-07	5.86E-05
Otu000001	7719.891351	-2.429481414	4.83E-07	8.99E-05
Otu000742	39.57138657	-4.180829915	5.72E-07	9.93E-05
Otu000955	10.90608493	-2.139605071	1.33E-06	0.00021682
Otu000045	118.3211302	0.731125137	3.37E-06	0.00051556
Otu000009	661.550546	-2.766118487	3.64E-06	0.00052601
Otu000007	1723.90967	0.922159935	8.24E-06	0.00112868
Otu000704	18.30145242	-2.192681134	8.81E-06	0.00114727
Otu000095	516.0361868	-2.17659591	1.20E-05	0.00148268
Otu001399	9.485192864	-3.957996116	1.51E-05	0.00178662
Otu001141	9.249016458	-2.662018911	2.54E-05	0.00276065
Otu002736	4.254723365	-5.284142397	2.53E-05	0.00276065
Otu000829	11.64733598	-3.400548146	3.95E-05	0.003812
Otu000914	14.04782863	-4.548524224	3.92E-05	0.003812
Otu001106	24.45647666	-3.048763938	3.70E-05	0.003812
Otu001915	5.242127999	-3.249286119	7.33E-05	0.00681819
Otu000039	190.6280753	-1.057285498	9.71E-05	0.00822898
Otu001050	5.080758543	-3.506730833	9.80E-05	0.00822898
Otu002258	7.115286919	-4.639803079	9.48E-05	0.00822898
Otu000493	15.68041012	3.634615478	0.00010607	0.00863167
Otu000019	889.9878696	-2.403302959	0.00011184	0.00882496
Otu000424	61.56780963	-1.94009952	0.0001411	0.01080642
Otu000227	66.79317544	-1.689332352	0.00015801	0.01175574
Otu000295	40.42403816	1.255838857	0.00016737	0.01210632
Otu000026	480.2604183	1.219374316	0.00017897	0.01259545
Otu000150	95.60471508	-1.804188525	0.00024196	0.01658081
Otu000953	15.50958421	1.911068794	0.00026435	0.01765031
Otu003631	2.452719511	-4.995383105	0.00031464	0.02048315
Otu000184	64.97104797	1.715063163	0.0003316	0.02106092
Otu000393	28.13736066	-2.063015792	0.00035875	0.02224279
Otu002349	3.098724315	3.309661712	0.00043686	0.02645525
Otu003277	2.236934978	-4.325607983	0.0005167	0.03057899
Otu001865	6.069528786	4.356313277	0.0005684	0.03289158
Otu000080	269.5414468	2.312317911	0.0006412	0.03552511
Otu001278	10.53663745	-2.870670773	0.0006341	0.03552511
Otu001233	4.381786022	-2.084817062	0.00068988	0.03742603
Otu000030	335.5461892	-0.942915414	0.00072583	0.03783163
Otu001270	8.62582692	-1.541643037	0.00072641	0.03783163
Otu000174	59.41911938	-1.011850841	0.00082066	0.04190209
Otu000308	58.39758452	1.203544452	0.00098947	0.04954974
Otu000963	7.07311573	4.141102387	0.00101426	0.04967152
Otu001135	11.02020919	-1.35139324	0.00103005	0.04967152

Otu000029	262.8272812	0.79519591	0.00022783	0.006014634
Otu000590	9.546481998	-1.80476374	0.00023109	0.006043351
Otu000928	23.76093425	-2.49787496	0.00024313	0.006298611
Otu001516	6.584273284	1.83309414	0.00024949	0.006403507
Otu000282	72.88411326	-2.46023487	0.00026006	0.006613735
Otu000306	51.88811553	-1.08769601	0.00026505	0.006679196
Otu003854	2.392627339	-4.74219078	0.0002709	0.006765219
Otu000457	29.79077021	-5.04084387	0.00029653	0.007339002
Otu000998	14.8133395	-1.40838636	0.0003018	0.007403422
Otu000317	37.79344976	-0.91747471	0.00031241	0.007596462
Otu001260	9.093591951	-1.86370643	0.00032524	0.007839707
Otu001160	10.6128388	-2.265035	0.00034407	0.008151789
Otu002232	4.633682928	-3.08246122	0.00034179	0.008151789
Otu000393	55.80194446	-2.2674304	0.00035182	0.008264781
Otu002294	4.837683585	-3.79848663	0.00036817	0.008576088
Otu000504	8.971370728	1.43490696	0.00038409	0.008872492
Otu000513	32.17427099	-1.34585424	0.00039778	0.009112841
Otu001885	6.824532847	-3.11896494	0.00045039	0.010233356
Otu000227	26.89974355	-1.38229426	0.00046405	0.010458192
Otu000426	29.87501288	-1.02979423	0.00047307	0.010575446
Otu000943	23.14494702	-1.26948132	0.00048535	0.010760499
Otu001573	5.01703127	-2.07526974	0.00048911	0.010760499
Otu000431	19.1438667	-1.36716139	0.00050051	0.010924587
Otu000275	51.24548638	-0.88603481	0.00051341	0.011118518
Otu000050	156.9484712	0.86216259	0.00053642	0.011420128
Otu000066	196.004497	-0.77522275	0.0005397	0.011420128
Otu000424	7.016237941	1.84283238	0.00053941	0.011420128
Otu000132	17.40174632	1.8646272	0.00054899	0.011481828
Otu000134	83.75729037	1.00290628	0.00055918	0.011481828
Otu000808	22.86997599	-1.16970662	0.00055531	0.011481828
Otu002279	7.094289287	-4.79725498	0.00055575	0.011481828
Otu000226	64.99063748	-1.51760331	0.0005827	0.011715678
Otu000543	19.58383779	-1.14559365	0.00058157	0.011715678
Otu001092	14.6186844	-2.86284426	0.00058325	0.011715678
Otu000339	52.8619675	-1.06948463	0.00060152	0.011742376
Otu000880	7.555709274	-1.69931557	0.00059883	0.011742376
Otu001407	2.997980529	4.03440326	0.00059496	0.011742376
Otu002396	4.447540903	-3.30760401	0.00060137	0.011742376
Otu000266	39.82033394	-0.93839995	0.00065208	0.012442391
Otu000287	58.66409557	-1.45979325	0.00066621	0.012442391
Otu000689	20.22835073	-2.30997763	0.0006688	0.012442391
Otu000669	17.13793901	1.85726474	0.000662	0.012442391
Otu000726	12.31023229	2.73669234	0.00066132	0.012442391
Otu001570	8.984173293	-2.48609	0.00066683	0.012442391
Otu001866	7.09612313	2.06793298	0.00065044	0.012442391
Otu003274	2.102899265	4.04125001	0.00068028	0.012571574
Otu001179	12.13669913	-2.62779842	0.00069684	0.012792233
Otu001666	3.614650529	-3.78997498	0.00070489	0.01285496
Otu000264	27.14355549	1.41225819	0.00071112	0.012883773
Otu000071	183.7633151	1.63238657	0.00073699	0.013228366
Otu001228	5.155197169	2.79657678	0.00073981	0.013228366
Otu003843	1.971025975	-3.92269501	0.00074445	0.013228366
Otu000347	23.0924898	1.06705491	0.00075919	0.013319506
Otu002960	2.888537724	-3.96939868	0.00075594	0.013319506
Otu000381	43.09663634	-1.57097904	0.0008596	0.01480186
Otu000785	20.19492029	-1.45619638	0.0008597	0.01480186

Otu001120	14.40318501	-1.3491982	0.00085952	0.01480186
Otu001753	6.736866918	-2.71020989	0.0008688	0.014866187
Otu000062	129.7188599	0.59395814	0.00089035	0.015141477
Otu003122	2.138831154	-4.04283327	0.00090193	0.015244737
Otu001621	11.89244913	3.50443125	0.00092902	0.015607487
Otu000464	37.32575467	-1.99086455	0.00094339	0.015659153
Otu001394	11.28514981	-2.19502993	0.00094294	0.015659153
Otu000341	16.86587461	1.33717538	0.00097034	0.015915826
Otu000638	5.709872398	-2.96043571	0.00096523	0.015915826
Otu000648	10.07112715	1.31541552	0.0009781	0.015948761
Otu000403	29.30367297	-0.8732433	0.00098623	0.015987263
Otu001786	4.154069673	-2.44768108	0.00100602	0.016213337
Otu000183	90.901723	-0.86547716	0.00105852	0.016884543
Otu001128	7.806658335	-2.442444	0.00106447	0.016884543
Otu001461	9.019048577	-3.33535932	0.00106594	0.016884543
Otu001742	6.824906776	-2.10784563	0.0010768	0.016959548
Otu003722	2.660761737	-3.83474621	0.00109484	0.01714637
Otu000826	17.09691162	-1.52824881	0.00110897	0.017270054
Otu001610	8.20160504	-2.19589949	0.00111816	0.017315929
Otu000280	35.47350613	-1.41657095	0.0011306	0.017411238
Otu000413	27.22229707	0.90703192	0.00116895	0.017902438
Otu001323	3.157389973	3.5780419	0.00118095	0.017986789
Otu000213	70.21402393	-0.95261418	0.00120025	0.018081958
Otu000576	17.60579739	-1.22093622	0.00119527	0.018081958
Otu000888	18.77560402	2.06370495	0.00121534	0.018210318
Otu000235	47.95777444	0.65281103	0.00129054	0.019028595
Otu000311	49.17698301	-1.6152207	0.00128662	0.019028595
Otu001456	3.983742584	-2.15209811	0.00128063	0.019028595
Otu000782	17.54251482	-1.51279291	0.00130288	0.019108873
Otu000878	11.56971685	-1.42738435	0.00131384	0.019168259
Otu000077	173.9131308	-1.24314655	0.0013872	0.020132547
Otu000112	150.8511661	0.71988445	0.00139826	0.020187354
Otu000121	145.4477521	0.8336211	0.00140917	0.020239466
Otu000425	21.43709896	0.92822025	0.0014209	0.02030272
Otu000712	37.07653551	-2.06321831	0.0014395	0.020463057
Otu000702	16.04392453	-1.03756413	0.00152479	0.021564908
Otu000046	337.7827187	1.19310073	0.00154172	0.021693592
Otu000127	76.35294968	0.68483405	0.00159198	0.022287685
Otu000131	93.92405553	-0.95627625	0.00161884	0.022549808
Otu000682	14.66033947	-1.68198047	0.00163553	0.022668422
Otu002067	7.395632802	-2.91721039	0.00164774	0.022724075
Otu000045	179.2215028	1.04581815	0.0016803	0.023032481
Otu003081	3.515455213	-2.65013628	0.00168672	0.023032481
Otu000075	208.2276736	-0.81021527	0.00173102	0.023521479
Otu000429	20.85258062	-1.01906557	0.00179961	0.024216106
Otu002648	1.558784123	3.56807227	0.00179796	0.024216106
Otu000025	229.025819	-0.44898857	0.00185763	0.024404546
Otu000026	39.40121588	2.12784623	0.00183308	0.024404546
Otu000114	71.1688104	0.83955262	0.00185359	0.024404546
Otu000175	67.89190803	-0.86394461	0.00185752	0.024404546
Otu001072	6.408663858	1.56338732	0.00183758	0.024404546
Otu002019	7.162767508	-3.11450648	0.00194103	0.025379947
Otu001881	5.993349229	1.87168468	0.00196167	0.02552928
Otu000136	101.5953696	-0.99907094	0.00199143	0.025795585
Otu002424	4.961965353	-1.77587242	0.00202715	0.026136079
Otu001700	9.932240254	-1.58438334	0.00210034	0.026954381

Otu000070	54.9366377	1.72887939	0.00211746	0.027048833
Otu000044	149.2192947	-0.96982978	0.00214614	0.027289428
Otu000422	5.861631327	1.98396232	0.00217168	0.027488165
Otu001811	4.850602502	-2.92002698	0.00221971	0.027968318
Otu000102	176.4878804	-0.79494791	0.00227917	0.028587546
Otu001341	5.119702132	-2.45539697	0.00231476	0.028903245
Otu000148	102.4704234	-0.58029279	0.00236819	0.029437711
Otu000415	23.42497267	-1.87562629	0.00257227	0.031679687
Otu000414	37.67477592	-0.88678689	0.00258283	0.031679687
Otu002246	2.069270536	-3.44356623	0.00256234	0.031679687
Otu002115	1.97631016	3.38332186	0.00263074	0.032125153
Otu001076	9.422777699	-1.21322891	0.00264653	0.032176278
Otu000471	16.68842885	0.95670605	0.00267785	0.032414907
Otu000231	37.74343903	0.81459092	0.00273325	0.032941639
Otu001687	2.679266603	-2.87200326	0.00276229	0.033147508
Otu001230	10.8896663	1.33572305	0.00283145	0.033830983
Otu001987	6.868428725	-2.74288261	0.00285246	0.033935688
Otu000103	32.76135172	1.61819898	0.00295113	0.034868734
Otu000364	41.43305303	-1.01724164	0.00295604	0.034868734
Otu001138	7.608722449	-1.62448632	0.00297944	0.034995776
Otu000441	17.63401333	0.98307072	0.00299246	0.035000435
Otu000201	90.7414699	-1.28024396	0.00302283	0.035207107
Otu001342	5.169754277	-1.70569759	0.00304799	0.035351529
Otu000140	36.6684968	0.84521204	0.00313857	0.036192441
Otu002574	5.152451854	-3.3041999	0.0031466	0.036192441
Otu000479	20.43740475	-0.8915804	0.00321688	0.036847907
Otu002629	3.524788027	-2.93012502	0.00323745	0.036930929
Otu000962	18.71030879	1.12054357	0.00332317	0.037753394
Otu000090	216.883354	2.24073349	0.00342262	0.038273546
Otu000108	132.2204884	-0.6460683	0.0034179	0.038273546
Otu000334	13.68799249	1.21079077	0.00342418	0.038273546
Otu004333	1.560226381	-3.56703414	0.00339255	0.038273546
Otu002301	5.064416472	-2.55288948	0.00346429	0.038566338
Otu001796	5.739260199	-1.56200488	0.00349065	0.038704376
Otu000222	26.04695219	1.22587636	0.00355116	0.03921834
Otu000711	21.0849203	-1.45607709	0.00360429	0.039551136
Otu003660	1.990007486	-3.93174341	0.00360983	0.039551136
Otu000365	28.95561105	0.71719075	0.00375569	0.040987259
Otu000736	18.52465027	-1.42795018	0.00380306	0.041341465
Otu000167	80.33903991	-0.63630499	0.00384309	0.04161347
Otu001803	6.704644231	-1.47953995	0.00397962	0.042924158
Otu000118	99.50103654	0.72814679	0.0040826	0.043734734
Otu000618	18.43142562	1.4272538	0.00409486	0.043734734
Otu001490	6.422142718	1.58992264	0.0041021	0.043734734
Otu000181	57.34728709	-0.67320024	0.00415548	0.043803639
Otu000277	85.92743291	0.98384292	0.00418758	0.043803639
Otu001505	6.959766099	-2.03123603	0.0041806	0.043803639
Otu001737	8.495258915	2.0301132	0.00417051	0.043803639
Otu002137	3.334164544	3.23648824	0.0041383	0.043803639
Otu002400	3.18722153	-2.20599127	0.00422902	0.04406573
Otu002491	2.083130622	2.94145114	0.00426032	0.04406573
Otu003246	3.130348143	-2.73945548	0.0042468	0.04406573
Otu000903	21.27546628	-1.93106558	0.004282	0.044125245
Otu001931	4.162832232	-1.87309777	0.00435168	0.044677274
Otu000172	83.47916351	-0.45295321	0.00438963	0.044900554
Otu003780	1.962299004	4.46256512	0.00463895	0.047276308

Otu000270	17.90933022	-1.57588129	0.00489226	0.049355644
Otu001360	8.301319676	-1.55899058	0.00489639	0.049355644
Otu002811	1.419876398	3.41772125	0.00489145	0.049355644
Otu000437	17.41186161	1.61387201	0.00493341	0.049486367
Otu001054	10.51791213	-1.7496681	0.0049557	0.049486367
Otu001115	7.054783986	1.68561222	0.00496292	0.049486367
Otu000212	58.28856839	-0.51242747	0.00507838	0.049919315
Otu000451	26.5161681	-1.13442067	0.00504581	0.049919315
Otu000655	17.04771088	5.07681636	0.00505103	0.049919315
Otu001598	3.987138503	1.9810824	0.00507068	0.049919315
Otu001912	7.088744429	-1.77729158	0.00509638	0.049919315
