# Supplementary material for: Rice SST Variation Shapes the Rhizosphere Bacterial Community, Conferring Tolerance to Salt Stress through Regulating Soil Metabolites
Source: mSystems. 2020 Nov 24;5(6):e00721-20. doi: 10.1128/mSystems.00721-20 (PMC7687028; doi:10.1128/mSystems.00721-20)
Supplement: TABLE S3 [file mSystems.00721-20-st003.pdf]

TableS3 Peaks with names were detected in Na-HHZ and Na-HHZ*cas*

index	Na-HHZ_mean	Na-HHZcas_mean	log2_FC	Pvalue	fdr	vip	MS2 name	MS2 score	MS1 name	MS1 ppm	SuperClass	formula	CAS	molecular weight	rt	mz
NEG00001	182912.1374	140060.4869	-0.3851	0.445119	0.7774	0.15926	Stearic acid	0.9999835			Lipids and lipid-like molecules	-	-	-	35.0365	284.059318
NEG00002	479905.6533	495649.1635	0.046568	0.864306	0.9547	0.02459	16-Methylheptadecanoic acid	0.9999723			Lipids and lipid-like molecules	-	-	-	57.50755	283.072365
NEG00003	14169963.33	14819907.14	0.0647	0.703395	0.8994	0.14175	methyl acetate	0.9999657			Organic acids and derivatives	-	-	-	117.15	73.0294714
NEG00004	26157444.27	24913406.91	-0.0703	0.838897	0.9471	0.62125	propanoic acid	0.9999578			Organic acids and derivatives	-	-	-	5.41692	73.0294614
NEG00005	83566688	81838528.67	-0.03015	0.83447	0.9453	0.02627	cis-Vaccenic acid	0.9999536			Lipids and lipid-like molecules	C18H34O2	506-17-2	282.2559	43.25095	281.248263
NEG00006	767403.1236	720177.3821	-0.09163	0.632789	0.8642	0.05427	15-Methylpalmitate	0.99995			Organic compounds	-	-	-	36.2849	269.045448
NEG00007	1230933.378	761714.7195	-0.69243	0.188081	0.6466	0.65294	(9Z,12Z)-octadeca-9,12-dienoic acid	0.9999045			Lipids and lipid-like molecules	-	-	-	37.3507	278.248727
NEG00008	121714.4482	97929.56435	-0.31368	0.252675	0.6865	0.16306	(Z)-hexadec-9-enoic acid	0.9998888			Lipids and lipid-like molecules	-	-	-	40.4453	252.969757
NEG00009	24462.80476	49916.50338	1.028927	0.173157	0.6325	0.15379	Palmitic acid	0.99985			Lipids and lipid-like molecules	-	-	-	48.5006	254.815736
NEG00010	46216082.27	76811757.68	0.732932	0.653254	0.8741	2.26193	nonanoic acid	0.999759			Lipids and lipid-like molecules	-	-	-	56.6136	157.050776
NEG00011	2905627.002	3282089.143	0.175765	0.715204	0.9033	0.18443	2-hydroxybenzaldehyde	0.9997175			Benzenoids	-	-	-	20.7452	121.014665
NEG00012	25185027.61	25812867.08	0.035524	0.89146	0.9637	0.18219	ethyl dodecanoate	0.9996655			Lipids and lipid-like molecules	-	-	-	50.0906	227.201462
NEG00013	84007.52366	87710.72601	0.062235	0.881823	0.9595	0.00849	dodecanoic acid	0.9996395			Lipids and lipid-like molecules	-	-	-	34.78085	198.912959
NEG00014	94194.01375	100550.859	0.094218	0.688377	0.8909	0.01797	4-dodecylbenzenesulfonic acid	0.9993612			Benzenoids	-	-	-	25.5928	324.209364
NEG00015	364416.2507	291979.2098	-0.31972	0.572873	0.8369	0.14011	Saccharin	0.9988709			Organoheterocyclic compounds	-	-	-	5.24534	180.991569
NEG00016	30393216.95	22888215.35	-0.40914	0.037998	0.4277	3.01951	heptadecanoic acid	0.9988167			Lipids and lipid-like molecules	C17H34O2	506-12-7	270.2559	43.5668	269.248337
NEG00017	365409.7452	336488.1214	-0.11896	0.576487	0.839	0.13493	methanesulfonic acid	0.998628			Organic compounds	-	-	-	32.51325	94.9653641
NEG00018	7748543.946	6873229.283	-0.17294	0.047974	0.4632	1.25397	phthalic acid	0.9983465			Benzenoids	C8H6O4	88-99-3	166.0266	350.811	165.019073
NEG00019	7748543.946	6873229.283	-0.17294	0.047974	0.4632	1.25397	phthalic acid	1.9983465			Benzenoids	C8H6O5	88-99-4	166.0266	350.811	165.019073
NEG00020	7748543.946	6873229.283	-0.17294	0.047974	0.4632	1.25397	phthalic acid	2.9983465			Benzenoids	C8H6O6	88-99-5	166.0266	350.811	165.019073
NEG00021	7748543.946	6873229.283	-0.17294	0.047974	0.4632	1.25397	phthalic acid	3.9983465			Benzenoids	C8H6O7	88-99-6	166.0266	350.811	165.019073

NEG00022	7748543.946	6873229.283	-0.17294	0.047974	0.4632	1.25397	phthalic acid	4.9983465	Benzenoids	C8H6O8	88-99-7	166.0266	350.811	165.019073
NEG00023	7748543.946	6873229.283	-0.17294	0.047974	0.4632	1.25397	phthalic acid	5.9983465	Benzenoids	C8H6O9	88-99-8	166.0266	350.811	165.019073
NEG00024	7748543.946	6873229.283	-0.17294	0.047974	0.4632	1.25397	phthalic acid	6.9983465	Benzenoids	C8H6O10	88-99-9	166.0266	350.811	165.019073
NEG00025	7748543.946	6873229.283	-0.17294	0.047974	0.4632	1.25397	phthalic acid	7.9983465	Benzenoids	C8H6O11	88-99-10	166.0266	350.811	165.019073
NEG00026	7748543.946	6873229.283	-0.17294	0.047974	0.4632	1.25397	phthalic acid	8.9983465	Benzenoids	C8H6O12	88-99-11	166.0266	350.811	165.019073
NEG00027	7748543.946	6873229.283	-0.17294	0.047974	0.4632	1.25397	phthalic acid	9.9983465	Benzenoids	C8H6O13	88-99-12	166.0266	350.811	165.019073
NEG00028	7748543.946	6873229.283	-0.17294	0.047974	0.4632	1.25397	phthalic acid	10.998346	Benzenoids	C8H6O14	88-99-13	166.0266	350.811	165.019073
NEG00029	7748543.946	6873229.283	-0.17294	0.047974	0.4632	1.25397	phthalic acid	11.998346	Benzenoids	C8H6O15	88-99-14	166.0266	350.811	165.019073
NEG00030	7748543.946	6873229.283	-0.17294	0.047974	0.4632	1.25397	phthalic acid	12.998346	Benzenoids	C8H6O16	88-99-15	166.0266	350.811	165.019073
NEG00031	7748543.946	6873229.283	-0.17294	0.047974	0.4632	1.25397	phthalic acid	13.998346	Benzenoids	C8H6O17	88-99-16	166.0266	350.811	165.019073
NEG00032	7748543.946	6873229.283	-0.17294	0.047974	0.4632	1.25397	phthalic acid	14.998346	Benzenoids	C8H6O18	88-99-17	166.0266	350.811	165.019073
NEG00033	7748543.946	6873229.283	-0.17294	0.047974	0.4632	1.25397	phthalic acid	15.998346	Benzenoids	C8H6O19	88-99-18	166.0266	350.811	165.019073
NEG00034	7748543.946	6873229.283	-0.17294	0.047974	0.4632	1.25397	phthalic acid	16.998346	Benzenoids	C8H6O20	88-99-19	166.0266	350.811	165.019073
NEG00035	7748543.946	6873229.283	-0.17294	0.047974	0.4632	1.25397	phthalic acid	17.998346	Benzenoids	C8H6O21	88-99-20	166.0266	350.811	165.019073
NEG00036	7748543.946	6873229.283	-0.17294	0.047974	0.4632	1.25397	phthalic acid	18.998346	Benzenoids	C8H6O22	88-99-21	166.0266	350.811	165.019073
NEG00037	7748543.946	6873229.283	-0.17294	0.047974	0.4632	1.25397	phthalic acid	19.998346	Benzenoids	C8H6O23	88-99-22	166.0266	350.811	165.019073
NEG00038	7748543.946	6873229.283	-0.17294	0.047974	0.4632	1.25397	phthalic acid	20.998346	Benzenoids	C8H6O24	88-99-23	166.0266	350.811	165.019073
NEG00039	7748543.946	6873229.283	-0.17294	0.047974	0.4632	1.25397	phthalic acid	21.998346	Benzenoids	C8H6O25	88-99-24	166.0266	350.811	165.019073
NEG00040	7748543.946	6873229.283	-0.17294	0.047974	0.4632	1.25397	phthalic acid	22.998346	Benzenoids	C8H6O26	88-99-25	166.0266	350.811	165.019073
NEG00041	7748543.946	6873229.283	-0.17294	0.047974	0.4632	1.25397	phthalic acid	23.998346	Benzenoids	C8H6O27	88-99-26	166.0266	350.811	165.019073
NEG00042	7748543.946	6873229.283	-0.17294	0.047974	0.4632	1.25397	phthalic acid	24.998346	Benzenoids	C8H6O28	88-99-27	166.0266	350.811	165.019073
NEG00043	7748543.946	6873229.283	-0.17294	0.047974	0.4632	1.25397	phthalic acid	25.998346	Benzenoids	C8H6O29	88-99-28	166.0266	350.811	165.019073
NEG00044	7748543.946	6873229.283	-0.17294	0.047974	0.4632	1.25397	phthalic acid	26.998346	Benzenoids	C8H6O30	88-99-29	166.0266	350.811	165.019073

NEG00045	7748543.946	6873229.283	-0.17294	0.047974	0.4632	1.25397	phthalic acid	27.998346	Benzenoids	C8H6O31	88-99-30	166.0266	350.811	165.019073
NEG00046	7748543.946	6873229.283	-0.17294	0.047974	0.4632	1.25397	phthalic acid	28.998346	Benzenoids	C8H6O32	88-99-31	166.0266	350.811	165.019073
NEG00047	7748543.946	6873229.283	-0.17294	0.047974	0.4632	1.25397	phthalic acid	29.998346	Benzenoids	C8H6O33	88-99-32	166.0266	350.811	165.019073
NEG00048	7748543.946	6873229.283	-0.17294	0.047974	0.4632	1.25397	phthalic acid	30.998346	Benzenoids	C8H6O34	88-99-33	166.0266	350.811	165.019073
NEG00049	7748543.946	6873229.283	-0.17294	0.047974	0.4632	1.25397	phthalic acid	31.998346	Benzenoids	C8H6O35	88-99-34	166.0266	350.811	165.019073
NEG00050	7748543.946	6873229.283	-0.17294	0.047974	0.4632	1.25397	phthalic acid	32.998346	Benzenoids	C8H6O36	88-99-35	166.0266	350.811	165.019073
NEG00051	7748543.946	6873229.283	-0.17294	0.047974	0.4632	1.25397	phthalic acid	33.998346	Benzenoids	C8H6O37	88-99-36	166.0266	350.811	165.019073
NEG00052	7748543.946	6873229.283	-0.17294	0.047974	0.4632	1.25397	phthalic acid	34.998346	Benzenoids	C8H6O38	88-99-37	166.0266	350.811	165.019073
NEG00053	7748543.946	6873229.283	-0.17294	0.047974	0.4632	1.25397	phthalic acid	35.998346	Benzenoids	C8H6O39	88-99-38	166.0266	350.811	165.019073
NEG00054	7748543.946	6873229.283	-0.17294	0.047974	0.4632	1.25397	phthalic acid	36.998346	Benzenoids	C8H6O40	88-99-39	166.0266	350.811	165.019073
NEG00055	7748543.946	6873229.283	-0.17294	0.047974	0.4632	1.25397	phthalic acid	37.998346	Benzenoids	C8H6O41	88-99-40	166.0266	350.811	165.019073
NEG00056	720671.278	908741.2682	0.334528	0.255226	0.6873	0.30122	diethyl benzene-1,2-dicarboxylate	0.9359209	Benzenoids	-	-	-	39.3605	220.097648
NEG00057	1497896.218	1174787.456	-0.35054	0.549491	0.8288	0.43826	(Z)-2-methylbut-2-enedioic acid	0.9339745	Lipids and lipid-like molecules	-	-	-	58.3179	128.023152
NEG00058	2293995.08	2830701.541	0.303297	0.300693	0.7148	0.7369	hexadecanoic acid	0.9338981	Lipids and lipid-like molecules	-	-	-	592.307	254.96077
NEG00059	901788.4555	1071325.07	0.248535	0.374854	0.7432	0.23182	1H-pyrimidine-2,4-dione	0.9313932	Organoheterocyclic compounds	-	-	-	82.3989	110.035946
NEG00060	516584.2709	537109.8793	0.056214	0.753123	0.9161	0.06162	2,3-dihydroxypropanal	0.9296528	Organic oxygen compounds	-	-	-	335.947	89.0243588
NEG00061	2031173.636	1773266.207	-0.1959	0.271873	0.6972	0.50852	butanedioic acid	0.9121644	Organic acids and derivatives	-	-	-	399.955	116.972427
NEG00062	789919.2402	715585.9006	-0.14258	0.422715	0.7668	0.23441	2,4,6-trihydroxy-5-[1-(4-hydroxy-1,1,4,7-tetramethyl-1a,2,3,4a,5,6,7a,7b-	0.911528	Lipids and lipid-like molecules	-	-	-	49.88235	470.407647
NEG00063	858596.9976	1027008.072	0.258394	0.555663	0.83	0.31686	Maslinic acid	0.9067102	Lipids and lipid-like molecules	-	-	-	55.1021	471.347357
NEG00064	55627.12252	8725.64687	-2.67245	0.069501	0.5218	0.22806	Ursolic acid	0.8973356	Lipids and lipid-like molecules	C30H48O3	77-52-1	456.3604	24.2086	455.269882
NEG00065	1232643.332	842363.6076	-0.54924	0.005279	0.2039	0.77594	Uracil	0.8929346	Organoheterocyclic compounds	C4H4N2O2	66-22-8	112.0273	156.5575	110.024693
NEG00066	454406.1541	492011.7983	0.114711	0.599711	0.8475	0.16849	Eicosapentaenoic acid	0.8897453	Lipids and lipid-like molecules	C20H30O2	10417-94-4	302.2246	56.3392	300.261974

NEG00067	5352784.434	4079433.36	-0.39192	0.047831	0.4632	1.21813	16-Hydroxy hexadecanoic acid	0.8835173			Lipids and lipid-like molecules	-	-	-	43.7192	270.251603
NEG00068	7291453.234	10186764.93	0.482418	0.118313	0.5808	1.9636	2-methylidenebutanedioic acid	0.8647198			Lipids and lipid-like molecules	-	-	-	72.3208	129.019358
NEG00069	559789.6423	571470.629	0.029795	0.826322	0.9418	0.01613	(10E,12Z)-9-hydroxyoctadeca-10,12-dienoic acid	0.8594035			Lipids and lipid-like molecules	-	-	-	79.81415	294.887877
NEG00070	8280630.534	7884068.014	-0.0708	0.406741	0.7584	0.66498	phosphoric acid	0.8446242			Homogeneous non-metal compounds	H3O4P	7664-38-2	97.9769	420.873	96.9600399
NEG00071	238818.0789	213212.6962	-0.16362	0.311922	0.7182	0.13107	3alpha-Acetoxy-11-keto-beta-boswellic acid	0.8439695			Lipids and lipid-like molecules	-	-	-	35.37055	510.488616
NEG00072	1767379.73	1664964.759	-0.08612	0.242865	0.6822	0.37323	3-(3,4-Dihydroxy-5-methoxy)-2-propenoic acid	0.8396842			Phenylpropanoids and polyketides	-	-	-	121.417	208.061442
NEG00073	4004750.693	3564538.118	-0.168	0.38553	0.7481	0.55513	4-methyl-2-oxopentanoic acid	0.8374843			Organic acids and derivatives	-	-	-	39.6575	129.055711
NEG00074	561900.2752	406302.726	-0.46776	0.303121	0.7152	0.46349	5-oxohexanoic acid	0.8303547			Lipids and lipid-like molecules	-	-	-	228.714	129.975674
NEG00075	269968.5034	272262.3827	0.012207	0.932055	0.9771	0.01913	Ethyl stearate	0.8295794			Lipids and lipid-like molecules	-	-	-	22.14015	310.602357
NEG00076	2007325.331	1495846.877	-0.42431	0.023393	0.3593	0.94771	2-hydroxy-3-methylbutanoic acid	0.8281741			Lipids and lipid-like molecules	-	-	-	182.771	116.972365
NEG00077	448574.6216	392955.9445	-0.19098	0.218361	0.6668	0.25575	octadecanoic acid	0.8204428			Lipids and lipid-like molecules	-	-	-	161.233	282.777322
NEG00078	51008.96212	72597.71537	0.509173	0.091802	0.5568	0.15531	3-Methyl-2-oxovaleric acid	0.7979585			Organic acids and derivatives	-	-	-	346.282	129.97538
NEG00079	575009.3479	613807.6007	0.094201	0.579904	0.8394	0.08268	2-methylpentanedioic acid	0.7743043			Lipids and lipid-like molecules	-	-	-	692.5805	145.014083
NEG00080	5309637.116	5031713.384	-0.07756	0.898939	0.967	0.01116	Cholesterol sulfate	0.6602492			Lipids and lipid-like molecules	-	-	-	26.49365	465.303663
NEG00081	5594781.6	6464977.005	0.208563	0.327838	0.721	0.7674	12-Oxo-2,3-dinor-10,15-phytodienoic acid	0.5969691			Lipids and lipid-like molecules	-	-	-	29.31965	262.988664
NEG00082	3245138.765	2630448.321	-0.30297	0.342031	0.7298	0.64089	Monomethyl glutaric acid	0.5241198			Lipids and lipid-like molecules	-	-	-	149.899	144.066463
NEG00083	381032.4079	388744.5409	0.028909	0.909238	0.9701	0.03091	Sulfaphenazole	0.4721622			Organoheterocyclic compounds	C15H14N4O2	526-08-9	314.0838	16.65775	312.898456
NEG00084	462575.6708	1133539.888	1.293074	0.247149	0.6846	0.75059	(1R,2S,3S,4R,5R)-6,8-dioxabicyclo[3.2.1]octane-2,3,4-triol	0.4543707			Organoheterocyclic compounds	-	-	-	270.707	160.841868
NEG00124	2400674.483	1	-21.195	0.014054	0.3053	2.01666			Glyoxylate	16.670319		-	-	-	720.388	73.0294576
NEG00133	600927.2892	662231.78	0.140146	0.449887	0.7801	0.13527			5-Hydroxyisourate	0.4049876		-	-	-	719.1115	183.102498
NEG00135	156203.1226	1	-17.2531	0.051641	0.4801	0.42723			Stipitatote	-16.517275		-	-	-	718.9965	207.113786
NEG00147	235285.7855	206169.3746	-0.19058	0.136932	0.6016	0.20526			Sodium arsenite	-10.434116		-	-	-	717.437	128.901568

NEG00157	799466.2813	936098.7166	0.227623	0.634689	0.8653	0.15623	Fosetyl	-8.6539349	-	-	-	716.746	109.040671
NEG00163	2437016.097	1631687.982	-0.57875	0.285311	0.7027	0.88252	sn-Glycerol 3-phosphate	-0.2502303	-	-	-	715.68	171.06638
NEG00164	1633627.727	1	-20.6396	0.080196	0.5369	1.57943	Cacodylate	7.3931115	-	-	-	715.515	136.991144
NEG00178	208182.9736	144417.0424	-0.52761	0.172159	0.631	0.25376	3-Phospho-D-glycerate	-24.576299	-	-	-	714.2955	185.045351
NEG00185	924689.5834	696688.2751	-0.40846	0.299118	0.7148	0.48	Glyphosate	3.7884079	-	-	-	713.381	168.066464
NEG00190	1368646.007	1267225.173	-0.11108	0.496571	0.8016	0.3366	2,4-Dioxotetrahydropyrimidine D-ribonucleotide	-18.427112	-	-	-	712.731	325.183913
NEG00217	879944.1852	826942.5829	-0.08962	0.301657	0.7148	0.2367	Arsenobetaine	9.5748048	-	-	-	709.603	177.055628
NEG00224	833838.4385	788989.7147	-0.07976	0.703671	0.8994	0.1759	5-Nitrofurfural	-19.068012	-	-	-	708.906	140.071633
NEG00247	842928.6209	664139.5216	-0.34392	0.61481	0.8559	0.43304	Haloxydine	12.152192	-	-	-	705.6405	198.965453
NEG00253	1915168.187	2111702.972	0.140936	0.588548	0.8421	0.1428	(1S,3R)-3-(2,2-Dichloroethenyl)-2,2-dimethylcyclopropanecarb	-5.5036196	-	-	-	704.8905	208.061373
NEG00254	3172536.658	3317050.107	0.064264	0.89028	0.9634	0.01339	Dihydrogen phosphate	2.2622158	-	-	-	704.8295	95.9801428
NEG00269	125458.7442	127409.7676	0.022263	0.948507	0.983	0.02402	3-{[(2E)-4-Amino-4-oxobut-2-enoyl]amino}-L-alanine	3.9279841	-	-	-	702.6175	200.173414
NEG00281	1067587.808	1374928.189	0.365002	0.313881	0.7189	0.46878	Tetranitromethane	21.419463	-	-	-	701.237	195.029622
NEG00290	3137546.279	1830269.134	-0.77758	0.207726	0.6619	1.08452	L-Selenocysteine	0.6443561	-	-	-	699.836	167.046032
NEG00311	269703.4638	228282.3351	-0.24055	0.531215	0.8206	0.1339	3-Phospho-D-glycerate	-24.553806	-	-	-	697.794	185.045355
NEG00317	277396.1813	203754.4519	-0.44512	0.130542	0.5935	0.25415	1,1-Dichloroethylene epoxide	18.166084	-	-	-	696.9135	111.937475
NEG00324	1914141.165	1883908.259	-0.02297	0.965923	0.9898	0.04646	sn-Glycerol 3-phosphate	-0.419123	-	-	-	696.098	171.066351
NEG00343	1282997.556	886446.6881	-0.53341	0.309959	0.7168	0.65628	Fosetyl	-8.649131	-	-	-	693.608	109.040672
NEG00352	209386.7543	392975.1103	0.908268	0.06934	0.5213	0.49443	Sodium arsenite	-10.502073	-	-	-	692.225	128.901559
NEG00375	1061890.82	896641.6749	-0.24403	0.301069	0.7148	0.37016	2,4-Dioxotetrahydropyrimidine D-ribonucleotide	-18.447314	-	-	-	689.429	325.183906
NEG00390	1476005.965	1296366.43	-0.18722	0.409009	0.7599	0.32021	Glyphosate	3.7765277	-	-	-	687.332	168.066462
NEG00397	303550.0618	295244.7482	-0.04002	0.790971	0.9281	0.07806	Etobenzanid	16.195722	-	-	-	686.627	339.199433
NEG00406	388691.8474	361201.2828	-0.10582	0.252217	0.6865	0.1752	O-Phospho-L-serine	-20.639101	-	-	-	685.38	184.061404

NEG00409	2731969.134	2244145.628	-0.28377	0.522556	0.8155	0.69182	(1S,3R)-3-(2,2-Dichloroethenyl)-2,2-dimethylcyclopropanecarb	-5.497291	-	-	-	684.187	208.061374
NEG00412	4737148.178	3571462.202	-0.4075	0.226842	0.671	1.22299	Dihydrogen phosphate	2.2636058	-	-	-	683.984	95.9801429
NEG00417	860519.2916	634075.0794	-0.44055	0.099163	0.5601	0.50012	3-(Phosphoacetylamido)-L-alanine	10.854127	-	-	-	683.2795	241.119151
NEG00434	904759.1976	761265.0333	-0.24914	0.227123	0.671	0.37171	5-Nitrofurfural	-19.110834	-	-	-	680.1235	140.071627
NEG00441	215849.1073	200999.2368	-0.10283	0.463336	0.7842	0.12151	3-Phospho-D-glycerate	-24.539576	-	-	-	677.6825	185.045358
NEG00450	1084507.521	973697.5744	-0.15549	0.619706	0.858	0.25763	Haloxydine	12.080322	-	-	-	675.7	198.965439
NEG00480	2431441.601	2225444.407	-0.12772	0.594494	0.8453	0.33	L-Selenocysteine	0.5673143	-	-	-	670.935	167.046019
NEG00496	322952.1774	322938.7872	-5.98E-05	0.999776	1	0.05855	Sodium arsenite	-10.428644	-	-	-	668.009	128.901569
NEG00504	889510.0049	882764.5008	-0.01098	0.939162	0.9796	0.06848	Tetranitromethane	21.506426	-	-	-	666.335	195.029639
NEG00507	677685.3059	631007.1534	-0.10296	0.68427	0.8892	0.11308	O-Phosphorylhydroxylamine	12.970634	-	-	-	665.7775	112.003989
NEG00517	6088487.41	4998384.904	-0.28462	0.278634	0.6999	0.84529	Dihydrogen phosphate	2.1702359	-	-	-	665.0145	95.9801339
NEG00531	1307975.175	1436446.006	0.135169	0.55321	0.8299	0.18947	2,4-Dioxotetrahydropyrimidine D-ribonucleotide	-18.567494	-	-	-	663.714	325.183867
NEG00540	1150537.862	1075400.211	-0.09743	0.235104	0.6796	0.28351	Glyphosate	3.7851323	-	-	-	662.47	168.066463
NEG00550	3210551.791	1455239.305	-1.14156	0.004116	0.1825	1.79229	(1S,3R)-3-(2,2-Dichloroethenyl)-2,2-dimethylcyclopropanecarboxylate	-5.5912467	-	-	-	660.571	208.061354
NEG00554	2294911.327	2180825.285	-0.07356	0.876771	0.9578	0.30754	Fosetyl	-8.8124446	-	-	-	659.971	109.040654
NEG00578	382598.4561	382913.2405	0.001186	0.995141	0.9986	0.00386	Etobenzanid	16.074886	-	-	-	652.328	339.199392
NEG00597	333198.3538	387145.4777	0.216495	0.316982	0.7189	0.15461	Sodium arsenite	-10.689198	-	-	-	649.395	128.901535
NEG00598	866738.3683	1421080.85	0.71332	0.261253	0.6905	0.83532	Haloxydine	12.010502	-	-	-	649.218	198.965425
NEG00605	809769.5041	738467.8534	-0.13298	0.383421	0.7477	0.23676	Tetranitromethane	21.418638	-	-	-	647.9765	195.029622
NEG00606	2024649.156	1846547.295	-0.13284	0.804996	0.9327	0.16053	Iodide	21.051651	-	-	-	647.823	125.899895
NEG00615	4174359.632	4108370.552	-0.02299	0.873549	0.9572	0.12533	Tetrafluoroethylene	-14.069229	-	-	-	646.121	99.0063163

NEG00661	6162200.135	11414431.09	0.889342	0.205201	0.6619	2.01769		Dihydrogen phosphate	1.9903731	-	-	-	640.839	95.9801164
NEG00689	3391986.855	1920076.807	-0.82097	0.314865	0.7189	1.17091		3-(Phosphoacetylamido)-L-alanine	10.691272	-	-	-	631.4275	241.119112
NEG00698	1041336.383	454592.1864	-1.19579	0.269889	0.696	0.77451		Glyphosate	3.4828444	-	-	-	628.739	168.066412
NEG00708	685761.9279	708602.2852	0.047268	0.788383	0.9278	0.01827		Tetranitromethane	21.277885	-	-	-	627.658	195.029595
NEG00719	900275.28	1057557.555	0.232298	0.464761	0.7856	0.3465		(1S,3R)-3-(2,2-Dichloroethenyl)-2,2-dimethylcyclopropanecarboxylate	-5.589067	-	-	-	626.948	208.061355
NEG00729	4520929.45	4777004.837	0.079487	0.789865	0.928	0.31914		sn-Glycerol 3-phosphate	-1.3430395	-	-	-	626.433	171.066192
NEG00737	1077101.613	1218026.389	0.177391	0.627957	0.8612	0.1573		Haloxydine	11.873139	-	-	-	626.1115	198.965398
NEG00746	5883588.64	6223109.135	0.080939	0.726858	0.907	0.38475		Cacodylate	6.8020745	-	-	-	625.605	136.991062
NEG00764	564366.7126	607575.5545	0.106431	0.575387	0.8382	0.18626		2,6-Dichloroindophenol	7.2840265	-	-	-	624.68	267.090176
NEG00769	166648.5219	156284.1844	-0.09264	0.703834	0.8994	0.05941		Chrysoeriol	10.082617	-	-	-	624.572	299.258651
NEG00775	2460391.479	1998793.276	-0.29976	0.076669	0.5283	0.84782		Selenium Sulfide	17.530747	-	-	-	624.2405	110.02467
NEG00784	673870.5756	668952.9237	-0.01057	0.980777	0.9946	0.11112		Sodium arsenite	-10.750336	-	-	-	624.148	128.901527
NEG00787	1408044.629	1647445.595	0.226538	0.414477	0.7624	0.49075		Bromobenzene	3.8992417	-	-	-	624.0635	156.001236
NEG00788	166095.9689	187573.3917	0.175438	0.566393	0.8339	0.10035		2,4-DB	-13.909504	-	-	-	624.057	248.079859
NEG00790	7443710.226	6687903.546	-0.15447	0.254359	0.6868	0.94301		Tetrafluoroethylene	-14.257097	-	-	-	623.981	99.0062975
NEG00793	40205697.83	16563554.6	-1.27939	0.001186	0.0993	6.20035		Dihydrogen phosphate	1.8512686	-	-	-	623.884	95.9801029
NEG00796	828796.4498	1063455.253	0.35967	0.32369	0.7192	0.33423		O-Phosphorylhydroxylamine	12.719196	-	-	-	623.803	112.003961
NEG00806	1357460.75	1480874.867	0.125539	0.551753	0.8296	0.15572		1,1-Dichloroethylene epoxide	17.765504	-	-	-	623.2015	111.93743
NEG00825	824047.9692	574180.1332	-0.52122	0.227525	0.6711	0.48351		Potassium dichromate	-7.6226812	-	-	-	622.021	293.175081
NEG00850	4981252.675	4258815.63	-0.22606	0.168177	0.6293	0.88902		2,4-Dioxotetrahydropyrimidine D-ribonucleotide	-18.706105	-	-	-	620.949	325.183822
NEG00852	716258.7567	635508.0177	-0.17257	0.877342	0.9578	0.02516		Radon-222	13.747398	-	-	-	620.862	220.995775
NEG00865	3377368.38	3061792.698	-0.14152	0.218409	0.6668	0.60174		3-(Dichloromethylene)-2,5-pyrrolidinedione	10.296895	-	-	-	619.819	178.983577

NEG00873	1814349.931	1865482.01	0.040096	0.864062	0.9547	0.03901	Etobenzanid	15.8106	-	-	-	619.64	339.199302
NEG00901	581597.1001	608925.0834	0.066245	0.814104	0.9354	0.06817	Ferrous lactate	4.7788572	-	-	-	618.496	232.978842
NEG00938	415109.577	377948.3462	-0.1353	0.268836	0.6957	0.2095	Eschscholtzxanthin	-21.34966	-	-	-	616.047	565.836221
NEG00955	155078.02	205821.3927	0.408399	0.011902	0.2843	0.30609	Flumiclorac pentyl	20.840156	-	-	-	614.967	422.863957
NEG00957	83924.51959	117938.7774	0.490874	0.042363	0.4426	0.21177	4-Hydroxy-16,18-tritriacontanedione	-22.335434	-	-	-	614.964	507.840858
NEG00959	3117946.297	3073028.476	-0.02093	0.86613	0.9555	0.18599	Pancuronium	-2.6411956	-	-	-	614.9545	571.85311
NEG00964	913840.4078	857503.7415	-0.0918	0.252552	0.6865	0.2729	Butter acids	19.719592	-	-	-	614.624	502.849659
NEG00965	343012.3919	280611.9048	-0.28968	0.081472	0.5377	0.28487	Dihydro-alpha-ergocryptine mesylate	-11.850417	-	-	-	614.622	672.804738
NEG00966	720850.5772	646163.8212	-0.1578	0.352506	0.7326	0.26429	Neoarctin A	11.160761	-	-	-	614.622	741.808414
NEG00967	789695.0183	906719.2124	0.19936	0.593921	0.8453	0.22084	Terodiline hydrochloride	2.5462387	-	-	-	614.613	316.889533
NEG00990	388839.3724	390134.2404	0.004796	0.978131	0.994	0.06157	N-(4-Chloro-3-methyl-5-isothiazolyl)-N-methyl-2-[p-[(alpha,alpha,alpha-trifluoro-p-tolyl)oxy]phenyl]acetamid[illegible]	24.767868	-	-	-	613.959	439.870143
NEG01011	825301.1384	734845.4602	-0.16748	0.580713	0.8399	0.06279	cis-Chlorobenzene dihydrodiol	-22.717441	-	-	-	613.1495	248.893846
NEG01013	341049.9821	338534.9194	-0.01068	0.970251	0.9915	0.03566	2,4-Dibromophenol	-18.456007	-	-	-	612.988	250.891474
NEG01016	2245419.984	904517.2426	-1.31177	0.011685	0.2819	1.44079	Iodide	21.961282	-	-	-	612.432	125.90001
NEG01029	729255.6607	2288559.628	1.649943	0.13534	0.5997	1.3007	Nornitrogen mustard	4.4785009	-	-	-	611.643	141.020359
NEG01034	107480.9134	82237.69368	-0.38621	0.160974	0.6224	0.18454	Oleanoic acid 3-O-glucuronide	-12.360672	-	-	-	611.27	631.809301
NEG01044	468654.7449	367081.9401	-0.35242	0.14939	0.6145	0.38158	2,3,23-Triacetylsericic acid	16.547727	-	-	-	610.7085	629.811762
NEG01045	1074052.432	1214293.688	0.177053	0.492101	0.8004	0.19032	2-Bromoacetaldehyde	21.465752	-	-	-	610.5095	121.943963
NEG01051	1204809.301	1028021.337	-0.22893	0.280124	0.7002	0.46775	Anhydrochlortetracycline	-0.4630491	-	-	-	610.156	459.85681
NEG01053	479190.7142	419525.4582	-0.19184	0.524117	0.8163	0.23438	Reduced haloperidol	8.1335199	-	-	-	609.765	376.875797
NEG01063	88424.18899	71674.74281	-0.30298	0.483088	0.7977	0.12108	Janthitrem E	20.583074	-	-	-	608.984	602.793251
NEG01075	117574.8297	108939.279	-0.11006	0.769327	0.9221	0.07305	2-Hexaprenyl-6-hydroxyphenol	19.187263	-	-	-	608.216	517.815478

NEG01089	93559.083	75158.8744	-0.31593	0.062855	0.5039	0.17132	Eupachlorin acetate	-9.4949443	-	-	-	607.2295	453.886404
NEG01093	65539.94998	68538.9829	0.06455	0.771659	0.9221	0.00244	4-Hydroxy-16,18-tritriacontanedione	19.26402	-	-	-	606.207	507.862026
NEG01094	141812.8258	151856.143	0.098717	0.698187	0.8974	0.05036	Muricadienin	-10.331921	-	-	-	606.1465	513.853004
NEG01096	766921.4241	706679.0228	-0.11802	0.263632	0.6922	0.28778	beta-2,3,5,6-Tetrachloro-1,4-cyclohexanediol	-4.8610502	-	-	-	605.077	252.929989
NEG01099	113299.0523	111983.0901	-0.01685	0.930129	0.9764	0.03226	Pyridate	-23.825701	-	-	-	604.855	377.899795
NEG01131	1665000.973	1638811.62	-0.02287	0.806083	0.9332	0.0697	Dihydrogen phosphate	2.3022756	-	-	-	601.526	95.9801467
NEG01145	450651.6893	586176.1968	0.379322	0.585618	0.8418	0.17681	Sodium molybdate	16.368012	-	-	-	600.2435	204.913194
NEG01148	796590.5692	763098.5323	-0.06197	0.475956	0.7932	0.19257	Haloxydine	12.260672	-	-	-	599.996	198.965475
NEG01164	99218.11582	92801.34527	-0.09646	0.737426	0.9119	0.07688	Pyrimidifen	14.809852	-	-	-	598.4085	376.90652
NEG01174	307168.5461	313196.044	0.028035	0.867284	0.9556	0.009	Fenvalerate	18.823309	-	-	-	597.8245	418.900727
NEG01209	2912636.379	2230112.304	-0.38521	0.266058	0.6938	0.70055	2-Bromoacetaldehyde	21.870623	-	-	-	593.749	121.944012
NEG01238	192938.889	157968.4926	-0.28851	0.15307	0.6159	0.21497	2,3,7,8-Tetrachlorodibenzodioxin	21.788445	-	-	-	591.906	320.970739
NEG01252	3565124.066	3254745.009	-0.13141	0.457403	0.7822	0.49034	Cryolite	1.9298855	-	-	-	591.215	208.934429
NEG01258	4731712.356	4560794.669	-0.05308	0.747593	0.9146	0.39777	Tetrafluoroethylene	-13.877738	-	-	-	590.9275	99.0063354
NEG01290	583059.7461	559528.4102	-0.05943	0.607448	0.8516	0.13064	3-(Dichloromethylene)-2,5-pyrrolidinedione	10.291819	-	-	-	587.95	178.983576
NEG01294	300379.2505	285313.7431	-0.07424	0.714604	0.9033	0.10269	Glyburide	-12.856588	-	-	-	587.437	492.989872
NEG01298	595245.8048	479981.9087	-0.31051	0.301238	0.7148	0.34076	2,5-Dichloro-4-oxohex-2-enedioate	10.895926	-	-	-	587.198	225.994197
NEG01300	859939.2303	517932.9469	-0.73147	0.084135	0.5411	0.62742	Ferrous lactate	4.9180503	-	-	-	587.127	232.978874
NEG01302	687757.6103	638218.047	-0.10785	0.566388	0.8339	0.20895	YC 170	5.9296932	-	-	-	587.102	486.972917
NEG01313	715135.1579	739265.3925	0.047876	0.875891	0.9578	0.04419	Butefine hydrochloride	21.730054	-	-	-	586.5465	352.928514
NEG01316	175662.7804	157113.1886	-0.161	0.50146	0.8029	0.12574	Novclobiocin 105	15.196213	-	-	-	586.413	574.992976
NEG01322	297378.6571	269958.4277	-0.13956	0.704638	0.8995	0.15571	Tetrachlorvinphos	16.016854	-	-	-	585.93	364.960385
NEG01328	159455.7055	152258.6437	-0.06663	0.75726	0.918	0.07967	3,5-Dichloro-2-methylmucote	19.335646	-	-	-	585.644	224.023174

NEG01329	74872.02848	75719.703	0.016242	0.935645	0.9788	0.02037	7-Epizucchini factor A	7.3963963	-	-	-	585.452	680.940767
NEG01359	7046820.698	6786042.318	-0.0544	0.755283	0.9171	0.276	6-Oxo-2-hydroxy-7-(4'-chlorophenyl)-3,8,8-trichloroocta-2E,4E,7E-trienoate	-18.178126	-	-	-	582.711	381.008679
NEG01375	2741495.588	4007928.487	0.547894	0.257704	0.6898	0.91763	Iodide	21.71685	-	-	-	580.535	125.899979
NEG01410	1275757.205	1621809.711	0.346251	0.270456	0.6967	0.60736	Hydroxyzine	-0.0024147	-	-	-	576.0275	373.897022
NEG01411	1112128.141	1244662.753	0.162432	0.626169	0.8602	0.1912	Mazaticol hydrochloride	-23.958757	-	-	-	575.96	441.017033
NEG01419	9637155.664	8912766.479	-0.11273	0.247404	0.6846	1.01046	Radon-222	12.629076	-	-	-	575.252	220.995527
NEG01420	1003409.691	845768.1195	-0.24658	0.491201	0.7998	0.38013	PE-NMe(14:1(9Z)/20:5(5Z,8Z,11Z,14Z,17Z))	-2.0348798	-	-	-	575.094	720.948254
NEG01441	1645298.471	1795517.203	0.12605	0.470114	0.7898	0.26448	Iodide	21.465982	-	-	-	558.5725	125.899948
NEG01449	2708692.783	3193771.785	0.237664	0.503412	0.8042	0.48321	6-Oxo-2-hydroxy-7-(4'-chlorophenyl)-3,8,8-trichloroocta	-18.255393	-	-	-	550.3575	381.008649
NEG01471	3063396.267	2656163.031	-0.20579	0.406685	0.7584	0.55518	6-Oxo-2-hydroxy-7-(4'-chlorophenyl)-3,8,8-trichloroocta	-18.243327	-	-	-	524.481	381.008654
NEG01473	1884323.937	2418338.081	0.359969	0.371476	0.7415	0.64718	Iodide	21.582365	-	-	-	522.883	125.899962
NEG01490	2562032.422	3591827.081	0.487429	0.20153	0.6597	0.78234	6-Oxo-2-hydroxy-7-(4'-chlorophenyl)-3,8,8-trichloroocta	-18.212735	-	-	-	503.83	381.008666
NEG01501	65044.30221	52231.37651	-0.31651	0.030675	0.3949	0.14452	Tetrachlorobisphenol A	-16.26664	-	-	-	495.907	365.053369
NEG01516	3164614.817	2224818.001	-0.50834	0.350967	0.7323	0.81144	Iodide	21.701384	-	-	-	481.6905	125.899977
NEG01520	5564523.272	5091610.655	-0.12814	0.131876	0.5958	0.86911	Radon-222	12.537136	-	-	-	480.286	220.995507
NEG01535	3211022.181	2900158.261	-0.1469	0.775756	0.9235	0.47132	6-Oxo-2-hydroxy-7-(4'-chlorophenyl)-3,8,8-trichloroocta	-18.148196	-	-	-	474.521	381.00869
NEG01537	508030.7501	455762.3836	-0.15663	0.656211	0.8753	0.10006	2-Bromoacetaldehyde	21.784963	-	-	-	471.098	121.944002
NEG01545	1285861.487	1272229.962	-0.01538	0.843738	0.9483	0.10609	Iodide	21.998634	-	-	-	452.8215	125.900015
NEG01560	7561678.637	6553232.107	-0.2065	0.685389	0.8899	0.79423	6-Oxo-2-hydroxy-7-(4'-chlorophenyl)-3,8,8-trichloroocta	-18.114583	-	-	-	442.025	381.008703
NEG01578	1352946.568	1272863.956	-0.08803	0.260012	0.6904	0.34128	Iodide	21.978828	-	-	-	434.882	125.900013
NEG01591	209030.8158	169814.6665	-0.29975	0.165432	0.6274	0.20968	PE(16:1(9Z)/16:1(9Z))	19.064389	-	-	-	422.535	686.945839

NEG01594	282363.2043	249211.8564	-0.18018	0.128255	0.5925	0.21556	7-Epizucchini factor A	-10.134657	-	-	-	421.9285	680.928812
NEG01602	272239.1802	148887.8	-0.87065	0.160976	0.6224	0.38377	3,5-Dibromo-4-hydroxyphenylpyruvate	-7.8847952	-	-	-	421.26	336.939559
NEG01624	288558.3293	263898.8658	-0.12888	0.218921	0.6675	0.17602	Butefine hydrochloride	-10.924926	-	-	-	421.0695	352.916957
NEG01629	640549.741	569186.3543	-0.17041	0.369188	0.7408	0.26857	Tetrachlorvinphos	-10.497768	-	-	-	421.032	364.950682
NEG01634	273442.8769	241001.9968	-0.18219	0.36446	0.7397	0.17869	Clemastine	-20.458246	-	-	-	421.0175	342.875888
NEG01651	67578.22596	62253.68454	-0.1184	0.368292	0.7406	0.06924	PE(14:1(9Z)/16:1(9Z))	-20.445738	-	-	-	420.834	658.853432
NEG01653	57383.29152	48178.07606	-0.25225	0.319413	0.7192	0.10085	Dimefline hydrochloride	2.4727141	-	-	-	420.828	358.840213
NEG01656	55017.69837	48068.85728	-0.19479	0.231207	0.6747	0.09469	Gravacridonolchlorine	24.380132	-	-	-	420.821	374.804886
NEG01659	71404.05884	67790.57374	-0.07492	0.614063	0.8559	0.05367	Chlorphonium chloride	10.039087	-	-	-	420.675	396.786817
NEG01661	58064.31704	49167.54486	-0.23995	0.356596	0.7347	0.09333	Ochratoxin A	-4.473098	-	-	-	420.644	402.803917
NEG01669	477672.8074	424044.2901	-0.17181	0.639852	0.8677	0.10816	2-Bromoacetaldehyde	21.794752	-	-	-	416.4255	121.944003
NEG01674	368109.4098	297199.8619	-0.3087	0.076022	0.5279	0.31695	PE-NMe(14:1(9Z)/20:5(5Z,8Z,11Z,14Z,17Z))	-1.7142648	-	-	-	414.1485	720.948486
NEG01675	540994.2877	619377.2824	0.195205	0.587193	0.8421	0.15521	Mazaticol hydrochloride	-23.831997	-	-	-	413.958	441.017089
NEG01686	1411401.07	1237213.384	-0.19003	0.146176	0.6127	0.49271	Iodide	21.895756	-	-	-	411.132	125.900002
NEG01690	3144324.255	3854638.615	0.293846	0.322474	0.7192	0.80857	Tetrafluoroethylene	-14.014999	-	-	-	410.76	99.0063217
NEG01709	2192367.468	2099088.497	-0.06273	0.907878	0.9697	0.31739	Novclobiocin 105	15.034358	-	-	-	409.258	574.992883
NEG01713	2444087.116	2319112.976	-0.07572	0.802127	0.9311	0.19933	YC 170	5.8110748	-	-	-	408.5985	486.972859
NEG01714	2069180.432	2923302.712	0.49854	0.117322	0.5786	1.08313	Glyburide	-12.889745	-	-	-	408.586	492.989856
NEG01721	588049.9285	489613.2731	-0.2643	0.486703	0.7989	0.2806	PS(DiMe(9,3)/DiMe(9,3))	15.925126	-	-	-	407.735	808.968222
NEG01730	1068058.186	973551.4165	-0.13366	0.798147	0.9306	0.0664	2,5-Dichloro-4-oxohex-2-enedioate	10.939029	-	-	-	405.8295	225.994207
NEG01737	4273971.996	7687700.675	0.846975	0.320094	0.7192	1.23812	6-Oxo-2-hydroxy-7-(4'-chlorophenyl)-3,8,8-trichloroocta	-18.155001	-	-	-	404.133	381.008688
NEG01748	222447.9174	195212.8795	-0.18842	0.190859	0.6485	0.18705	3-Phospho-D-glycerate	-24.393298	-	-	-	391.63	185.045385
NEG01764	690994.327	728819.2678	0.076887	0.528487	0.8188	0.0741	UTP	-2.560463	-	-	-	390.864	483.132584

NEG01766	297877.1907	306333.6268	0.040386	0.787123	0.927	0.00099	Prochlorperazine mesylate	-19.567108	-	-	-	390.864	565.135645
NEG01794	1806985.239	1380536.242	-0.38836	0.114892	0.5747	0.74315	Iodide	21.658431	-	-	-	385.951	125.899972
NEG01795	316331.6798	284224.1913	-0.15441	0.636127	0.8664	0.11981	2-Bromoacetaldehyde	21.728372	-	-	-	385.913	121.943995
NEG01810	996150.1248	883070.4074	-0.17383	0.888389	0.9626	0.22742	Tetrafluoroethylene	8.4444512	-	-	-	382.3935	99.008568
NEG01820	6155175.761	4163007.77	-0.56417	0.417093	0.7646	1.53485	6-Oxo-2-hydroxy-7-(4'-chlorophenyl)-3,8,8-trichloroocta-2E,4E,7E-trienoate	-18.259867	-	-	-	377.166	381.008648
NEG01825	148783.9187	161560.5611	0.118856	0.395718	0.7526	0.07888	Propanil	-5.8040325	-	-	-	374.936	217.071358
NEG01849	417665.4045	311345.0803	-0.42383	0.041473	0.4403	0.39583	2-Hydroxyethylphosphote	15.990023	-	-	-	362.0475	125.043039
NEG01855	4178844.346	1894788.817	-1.14107	0.075619	0.5279	1.61461	Iodide	21.774784	-	-	-	358.538	125.899987
NEG01861	58331.64441	73518.56234	0.33383	0.35064	0.7323	0.08926	Prochlorperazine mesylate	-20.018294	-	-	-	358.347	565.13539
NEG01869	272032.3425	359014.9204	0.400266	0.224502	0.6707	0.23605	UTP	-2.8377245	-	-	-	358.286	483.13245
NEG01883	32412.98608	42531.22242	0.39195	0.279971	0.7002	0.07247	PC(18:2(9Z,12Z)/P-18:1(11Z))	-3.0583579	-	-	-	358.244	767.088474
NEG01899	391701.64	370151.3161	-0.08164	0.586215	0.8419	0.14285	PE(14:0/18:0)	9.6411748	-	-	-	355.355	690.971395
NEG01902	256809.0539	207400.106	-0.30828	0.330257	0.7223	0.25385	PE(14:0/P-18:0)	-18.14106	-	-	-	354.614	674.940161
NEG01911	220363.232	183550.1617	-0.26371	0.243545	0.6825	0.21567	Monoammonium glycyrrhizite	-11.157094	-	-	-	353.919	838.945952
NEG01919	75068.60429	68835.34381	-0.12506	0.58664	0.8419	0.05955	Quadrigemine A	21.196948	-	-	-	352.696	689.928769
NEG01923	490425.8014	390961.6886	-0.32701	0.056832	0.4892	0.40996	YC 170	5.5430245	-	-	-	352.3535	486.972728
NEG01924	69441.9833	53982.13484	-0.36333	0.220498	0.6688	0.12903	PA(14:1(9Z)/22:2(13Z,16Z))	-15.876147	-	-	-	352.326	697.944627
NEG01928	81804.2481	65849.14284	-0.31301	0.15029	0.6145	0.14486	PA(14:0/20:2(11Z,14Z))	21.578255	-	-	-	352.262	671.932244
NEG01934	79975.74539	56330.24692	-0.50565	0.074241	0.5245	0.16621	Spinosyn A	-24.326358	-	-	-	351.634	730.930418
NEG01935	160077.8117	170068.4696	0.087342	0.709356	0.9015	0.04106	Phenolic phosphate	-12.124008	-	-	-	351.63	173.081713
NEG01939	162653.2909	129488.4342	-0.32898	0.098352	0.5586	0.21367	beta1-Chaconine	10.179289	-	-	-	351.2235	704.918109
NEG01942	823137.3229	491783.447	-0.74311	0.062384	0.5022	0.66092	Glycerone phosphate	-0.2586163	-	-	-	350.895	169.050479

NEG01957	45535.69473	40661.10524	-0.16335	0.499329	0.8016	0.06548	Formamidopyrimidine nucleoside triphosphate	15.926651	-	-	-	350.212	540.197043
NEG01960	87492.03819	77259.84552	-0.17943	0.490025	0.7998	0.06074	Spirolide B	-10.950939	-	-	-	350.117	692.937224
NEG01962	106943.0206	117747.1675	0.13885	0.640485	0.8681	0.04633	Sorafenib beta-D-Glucuronide	20.749698	-	-	-	349.985	639.955023
NEG01970	5889985.7	5345695.479	-0.13989	0.129013	0.5925	0.92921	Radon-222	12.29675	-	-	-	349.7045	220.995453
NEG01971	508907.378	270851.9918	-0.9099	0.318359	0.7192	0.54381	2-Bromoacetaldehyde	21.655067	-	-	-	349.693	121.943986
NEG01985	79601.26881	67602.31179	-0.23572	0.337057	0.7276	0.1055	Formyl phosphate	-11.650321	-	-	-	348.836	124.996555
NEG02004	120871.6509	101161.463	-0.25682	0.316664	0.7189	0.13607	beta-Hederin	-13.542988	-	-	-	348.206	733.93887
NEG02007	119028.7687	95576.10548	-0.31659	0.251157	0.6861	0.152	PC(14:1(9Z)/16:1(9Z))	9.3296615	-	-	-	348.143	700.953172
NEG02013	201533.8965	173177.5342	-0.21877	0.431008	0.77	0.1591	CDP-DG(a-13:0/a-13:0)	8.3792776	-	-	-	347.7455	868.924013
NEG02016	63306.92574	56820.36355	-0.15596	0.56706	0.8344	0.06667	Digitoxigenin 3-[glucosyl-(1->6)-glucosyl-(1->4)-2,6	-8.4808166	-	-	-	347.417	827.922393
NEG02022	81318.26143	70359.79412	-0.20883	0.404481	0.7582	0.08293	Patellamide A	13.296816	-	-	-	347.113	741.954102
NEG02027	141064.6366	126656.312	-0.15544	0.503713	0.8042	0.07261	PS(18:4(6Z,9Z,12Z,15Z)/18:4(6Z,9Z,12Z,15Z))	-17.245698	-	-	-	346.859	774.940341
NEG02029	66950.12269	69248.3486	0.048693	0.850899	0.9515	0.00392	Beauvericin	18.374999	-	-	-	346.736	782.955928
NEG02038	109600.3094	95116.40696	-0.20449	0.198158	0.6561	0.11105	PC(14:0/P-16:0)	-9.4630946	-	-	-	346.359	688.972494
NEG02044	226138.6257	268596.139	0.248231	0.436967	0.7726	0.13718	PG(16:0/16:0)	-22.976148	-	-	-	346.159	721.958112
NEG02048	35890.64096	44749.31735	0.318258	0.169443	0.6304	0.1008	Bis(4-nitrophenyl)phosphate	-24.183579	-	-	-	345.992	339.166697
NEG02054	53446.43754	56157.08894	0.071374	0.801389	0.9311	0.02621	2-Aminoethyl diphenylborite	24.615762	-	-	-	345.7745	224.092164
NEG02057	141905.8948	136898.0649	-0.05183	0.780514	0.9255	0.0511	Glyphosate	3.2272867	-	-	-	345.738	168.066369
NEG02060	119693.5444	115225.8353	-0.05488	0.81236	0.935	0.01536	3-(Phosphoacetylamido)-L-alanine	9.9263479	-	-	-	345.588	241.118927
NEG02082	346125.6048	336845.9459	-0.03921	0.750671	0.9147	0.10124	DG(15:0/0:0/22:5n3)	4.7527687	-	-	-	344.931	627.974713
NEG02089	1008353.74	990959.1627	-0.0251	0.928909	0.9762	0.07998	14-hydroxyclarithromycin	18.382449	-	-	-	344.48	762.959567
NEG02096	2542754.035	2498342.489	-0.02542	0.92924	0.9763	0.08677	2,5-Dichloro-4-oxohex-2-enedioate	9.6393091	-	-	-	343.558	225.993912
NEG02098	610256.3673	545087.8314	-0.16293	0.379667	0.7473	0.1817	3(S),10(R)-OH-octadeca-6-trans-4,12-cis-trienoate	-17.298769	-	-	-	343.532	636.008704

NEG02102	42889396.94	49752428.75	0.214146	0.217713	0.6665	2.20201	Tetrafluoroethylene	-14.957536	-	-	-	343.2175	99.0062274
NEG02104	486430.4589	429823.3479	-0.17849	0.534168	0.8219	0.17664	PIP(16:1(9Z)/16:1(9Z))	-10.077291	-	-	-	342.904	885.963885
NEG02107	132721.2754	145560.1146	0.133215	0.650686	0.8732	0.0538	PIP2(16:0/16:1(9Z))	-2.4157219	-	-	-	342.892	967.966183
NEG02115	1642680.754	1442760.936	-0.18722	0.071234	0.5226	0.51524	PA(15:0/22:4(7Z,10Z,13Z,16Z))	15.681241	-	-	-	342.151	709.977872
NEG02116	1092307.078	658728.3996	-0.72962	0.018242	0.3332	0.88245	PS(14:1(9Z)/22:5(4Z,7Z,10Z,13Z,16Z))	6.8308416	-	-	-	342.022	778.991051
NEG02117	279837.6494	285829.1974	0.030563	0.870477	0.9567	0.05233	ADP-ribose 1'',2''phosphate	1.0552111	-	-	-	342.005	620.020779
NEG02128	204240.9869	212271.5782	0.055639	0.787938	0.9276	0.03056	TDE	13.215772	-	-	-	341.5705	319.038153
NEG02131	292867.5695	328772.3798	0.166841	0.259568	0.6904	0.13558	CDP-DG(18:2(9Z,11Z)/a-15:0)	-22.879634	-	-	-	341.523	963.051666
NEG02143	423801.577	487299.617	0.20142	0.646087	0.8704	0.07544	PS(14:0/22:4(7Z,10Z,13Z,16Z))	9.2315882	-	-	-	341.336	783.024961
NEG02144	581667.5386	624935.7369	0.103513	0.651805	0.8735	0.13448	PIP2(16:0/22:5(4Z,7Z,10Z,13Z,16Z))	-11.429464	-	-	-	341.336	1044.05258
NEG02147	509273.7112	639906.8762	0.329421	0.150278	0.6145	0.32029	Dichloran	-12.469842	-	-	-	341.327	206.004342
NEG02149	244415.6774	272711.961	0.158041	0.310136	0.7168	0.11852	Podorhizol beta-D-glucoside	-0.5663903	-	-	-	341.325	577.554196
NEG02150	518912.2551	590654.6719	0.186824	0.083573	0.5411	0.26194	Maytansine	6.315319	-	-	-	341.3245	691.193195
NEG02159	628180.7255	642492.8847	0.032501	0.813387	0.9353	0.04663	PA(15:0/22:0)	20.248601	-	-	-	341.25	718.045283
NEG02160	1252217.845	1422479.625	0.183922	0.046495	0.4568	0.41872	23-Hydroxyphysalolactone	-19.250874	-	-	-	341.2405	554.039038
NEG02161	1976917.232	2143076.274	0.116431	0.115251	0.575	0.38099	PC(18:4(6Z,9Z,12Z,15Z)/20:5(5Z,8Z,11Z,14Z,17Z))	-3.4522884	-	-	-	341.23	799.045361
NEG02169	144240.9458	172181.7069	0.255451	0.484221	0.798	0.10343	N-Demethyl-desepoxymaytansinol	-10.86483	-	-	-	341.213	534.01591
NEG02170	843441.5403	824128.7839	-0.03342	0.83615	0.9465	0.1045	Iodofenphos	0.6016322	-	-	-	341.206	411.989572
NEG02176	1677820.463	1712567.27	0.029572	0.801424	0.9311	0.04069	Sanguisorbin E	-5.4459978	-	-	-	341.128	791.980605
NEG02177	6951851.397	7825468.84	0.17078	0.052793	0.4813	0.92308	PG(16:0/22:4(7Z,10Z,13Z,16Z))	-18.404179	-	-	-	341.1265	798.043817
NEG02179	324945.5917	340409.4555	0.067073	0.708999	0.9015	0.06018	Chivosazole E	7.3250497	-	-	-	341.111	823.006559
NEG02184	254606.4582	198403.7846	-0.35983	0.281672	0.7016	0.26107	Ferrichrome	-17.212906	-	-	-	341.0675	739.500477
NEG02186	31865792.7	35477901.15	0.154912	0.055828	0.4884	1.86516	PS(15:0/22:4(7Z,10Z,13Z,16Z))	-5.3714617	-	-	-	341.0565	797.040437

NEG02188	1974368.697	2039216.195	0.046623	0.66433	0.8795	0.00795		CerP(d18:1/22:0)	-15.663608	-	-	-	341.055	701.021827
NEG02189	3671525.828	4028039.157	0.133698	0.11286	0.5707	0.51832		PGP(18:0/20:3(5Z,8Z,11Z))	-8.84023	-	-	-	341.055	880.046535
NEG02190	798762.4314	1000822.069	0.325347	0.024608	0.3595	0.49522		DG(15:0/0:0/22:1n9)	10.242231	-	-	-	341.0535	636.042248
NEG02195	5044540.152	5573683.154	0.143908	0.137991	0.6035	0.62257		PC(14:0/P-18:0)	14.489036	-	-	-	341.0355	717.042627
NEG02196	1985834.72	1888548.481	-0.07247	0.717098	0.904	0.25118		Alloxan	0.2346861	-	-	-	341.034	141.062457
NEG02197	18797456.84	21019641.18	0.161201	0.064616	0.509	1.40718		1-O-(1Z-Tetradecenyl)-2-(9Z-octadecenoyl)-sn-glycero-3-phosphocholine	23.244902	-	-	-	341.03	716.040891
NEG02200	151759.192	259979.2171	0.776612	0.017622	0.3285	0.38164		Scopoloside I	-9.1688171	-	-	-	341.028	932.025369
NEG02202	95748734.89	105626633.9	0.141648	0.085911	0.5433	2.83857		SM C16:1	-2.9453506	-	-	-	341.026	715.037414
NEG02203	922883.9066	1000543.874	0.116563	0.427973	0.7691	0.19075		31-Hydroxy rifabutin	6.1532225	-	-	-	341.026	862.002134
NEG02209	14863401.88	16347657.94	0.13732	0.113195	0.5714	1.06324		1-Diphosinositol pentakisphosphate	-12.485425	-	-	-	341.0235	738.998684
NEG02217	208785.1992	200284.6597	-0.05997	0.751509	0.9148	0.05426		TG(15:0/20:0/o-18:0)	24.397876	-	-	-	341.022	862.504791
NEG02228	15573371.99	16352990.65	0.070473	0.480431	0.7955	0.42726		Novclobiocin 105	14.477077	-	-	-	341.017	574.992562
NEG02229	10038902.09	10861351.15	0.113602	0.220008	0.6687	0.68726		Glyburide	-13.237815	-	-	-	341.0165	492.989684
NEG02230	1492426.676	1766552.88	0.243277	0.012084	0.2865	0.60264		Bryostatin 1	-19.068366	-	-	-	341.0165	904.008066
NEG02234	28300237.95	31270719.22	0.143998	0.079606	0.5356	1.58863		CE(5D5)	23.645743	-	-	-	341.0155	634.037739
NEG02242	524762.4828	533292.0888	0.023261	0.889954	0.9633	0.05064		PC(15:0/18:3(6Z,9Z,12Z))	-9.5600274	-	-	-	341.01	741.00343
NEG02246	42606828.32	47031189.97	0.142533	0.07845	0.5337	1.99537		3,5-Dichloro-2-methylmucote	17.943958	-	-	-	340.999	224.022861
NEG02260	4457645754	4838871613	0.118389	0.136191	0.6016	17.2216		Nornitrogen mustard	-20.914126	-	-	-	340.777	141.016753
NEG02264	1034632.52	1306515.539	0.336606	0.410073	0.7604	0.4124		Cycloheptanecarboxylic acid	-8.098718	-	-	-	340.719	141.187172
NEG02269	16026636.84	11020990.4	-0.54022	0.030831	0.3953	3.00836		6-Oxo-2-hydroxy-7-(4'-chlorophenyl)-3,8,8-trichloroocta-2E,4E,7E-trienoate	-18.459727	-	-	-	340.31	381.008571
NEG02270	185039.8521	200024.5481	0.112341	0.781631	0.9257	0.02945		CDP-DG(a-13:0/a-15:0)	18.774784	-	-	-	340.31	896.987583
NEG02273	158088.6746	170773.8628	0.111353	0.524765	0.8163	0.05013		Perfluorohexane sulfonic acid	14.922268	-	-	-	338.124	399.113694

NEG02301	119202.3232	114043.3359	-0.06383	0.711918	0.9019	0.05569	Monoammonium glycyrrhizite	-11.205501	-	-	-	334.561	838.945911
NEG02304	193075.7846	185507.9872	-0.05769	0.776989	0.9242	0.06245	DG(18:2n6/0:0/20:5n3)	-16.938398	-	-	-	334.215	651.982663
NEG02307	259941.1591	222606.343	-0.22369	0.364258	0.7397	0.18838	PE(14:0/18:0)	9.4357972	-	-	-	334.047	690.971253
NEG02313	188873.681	163241.099	-0.21042	0.264254	0.6923	0.17681	PE(14:0/P-18:0)	-17.815601	-	-	-	333.751	674.940381
NEG02316	501519.5147	657548.6932	0.390792	0.567165	0.8345	0.11307	L-Selenocysteine	1.1788023	-	-	-	333.3775	167.046122
NEG02317	468006.8167	459299.6638	-0.02709	0.945373	0.9818	0.02857	12,13-epoxy-9-alkoxy-10E-octadecenoate	0.3525251	-	-	-	333.36	156.990779
NEG02324	352455.5653	371663.9768	0.076557	0.770365	0.9221	0.0062	Clocapramine dihydrochloride	20.012277	-	-	-	332.734	552.99831
NEG02330	116323.7197	88618.29042	-0.39247	0.262381	0.6915	0.1814	1-Hexadecanoyl-2-(9Z-octadecenoyl)-sn-glycero-3-phosphoethanolamine	21.458627	-	-	-	332.315	717.004531
NEG02342	894170.6056	718976.6075	-0.31461	0.089584	0.5511	0.53688	Tetranitromethane	21.24353	-	-	-	330.892	195.029588
NEG02348	2361381.543	1760459.171	-0.42368	0.060527	0.4986	0.9488	YC 170	5.6713296	-	-	-	330.1155	486.972791
NEG02357	655295.5195	617460.5457	-0.0858	0.59177	0.8437	0.13399	sn-Glycerol 3-phosphate	-1.8881903	-	-	-	329.6485	171.066098
NEG02358	350254.6425	287191.8271	-0.28639	0.525997	0.8168	0.13147	PS(DiMe(9,3)/DiMe(9,3))	15.705221	-	-	-	329.618	808.968044
NEG02360	456676.8743	505476.1918	0.146469	0.529551	0.8192	0.15386	Barbiturate	-19.858021	-	-	-	329.486	127.07638
NEG02363	213238.4472	154466.9659	-0.46517	0.274471	0.6994	0.24951	FAHFA(22:6(4Z,7Z,10Z,13Z,16Z,19Z)/14-O-22:6(4Z,7Z,10Z,13Z,16Z,19Z))	-21.155109	-	-	-	329.1	653.954867
NEG02364	282851.6455	211158.6041	-0.42172	0.110926	0.5694	0.28511	DG(15:0/0:0/22:4n6)	3.3267249	-	-	-	329.071	629.989823
NEG02368	3467950.409	4635720.265	0.41871	0.412022	0.7613	0.73884	Iodide	21.504174	-	-	-	328.125	125.899952
NEG02370	12476536.77	11393999.45	-0.13094	0.127422	0.5923	1.30965	Radon-222	12.509505	-	-	-	327.9575	220.995501
NEG02374	551017.0975	503608.6506	-0.12979	0.284524	0.7021	0.23879	Perflutren	-13.637367	-	-	-	327.618	187.009459
NEG02376	953717.4009	1084211.235	0.185012	0.577621	0.8394	0.22591	2-Bromoacetaldehyde	21.584666	-	-	-	327.434	121.943977
NEG02379	394976.112	529723.3137	0.423474	0.255033	0.6871	0.31578	Glycerone phosphate	-0.0334322	-	-	-	327.1135	169.050518
NEG02387	823805.6319	957223.3207	0.216552	0.461485	0.7838	0.24383	Radium-224	0.0670983	-	-	-	326.661	224.992739
NEG02389	3335006.681	3376357.705	0.017778	0.937324	0.9792	0.12295	2-Iodophenol	-8.1159512	-	-	-	326.46	218.998738

NEG02392	1030949.023	1210715.93	0.231887	0.728954	0.9081	0.27481	Ferrous lactate	-12.427513	-	-	-	326.397	232.974816
NEG02401	132990.0555	114584.9656	-0.2149	0.369819	0.7408	0.14606	AS 1-1	-15.025236	-	-	-	325.545	684.954017
NEG02416	123532.8216	114254.4541	-0.11264	0.375945	0.7441	0.08295	(1E,3E)-4-Hydroxybuta-1,3-diene-1,2,4-tricarboxylate	8.799483	-	-	-	323.273	201.112802
NEG02421	6616358.588	6324545.449	-0.06508	0.72556	0.9063	0.49312	6-Oxo-2-hydroxy-7-(4'-chlorophenyl)-3,8,8-trichloroocta-2E,4E,7E-trienoate	-18.297218	-	-	-	321.572	381.008633
NEG02427	101790.8802	90140.34099	-0.17536	0.612489	0.8552	0.0976	Uvaricin	-14.487833	-	-	-	318.8125	647.936321
NEG02430	238077.0179	207256.8376	-0.20001	0.118701	0.5808	0.22007	PC(14:0/16:0)	-10.551379	-	-	-	317.6785	704.970974
NEG02434	808065.9338	761793.1288	-0.08507	0.629257	0.8619	0.17561	PS(15:0/16:0)	1.9874183	-	-	-	315.648	720.948158
NEG02440	991352.4549	821216.1933	-0.27164	0.52056	0.8142	0.19545	Mazaticol hydrochloride	-23.878217	-	-	-	314.848	441.017068
NEG02459	813189.7798	742263.0019	-0.13166	0.052347	0.4813	0.34204	Tetranitromethane	21.371504	-	-	-	309.5145	195.029613
NEG02467	150586.9675	132689.54	-0.18254	0.579805	0.8394	0.13014	Pseudoargiopinin I	-22.344642	-	-	-	306.908	742.9389
NEG02471	352835.8589	275009.0879	-0.35952	0.127783	0.5924	0.32142	lysoPC(28:1(5Z))	14.99177	-	-	-	306.603	660.935747
NEG02476	44392.40567	40583.53062	-0.12942	0.608688	0.8525	0.05111	PC(14:0/14:0)	-23.518975	-	-	-	306.538	676.909279
NEG02477	57273.84363	54992.9258	-0.05863	0.748959	0.9146	0.03528	Zuclopenthixol	-1.8407444	-	-	-	306.526	399.956985
NEG02484	1879680.492	1790608.882	-0.07004	0.57939	0.8394	0.21615	12,13-epoxy-9-alkoxy-10E-octadecenoate	-0.5097589	-	-	-	306.371	156.990643
NEG02504	805039.7979	671713.0215	-0.26122	0.197111	0.6557	0.42697	Mazaticol hydrochloride	-23.958884	-	-	-	299.574	441.017033
NEG02507	1631610.729	1881941.523	0.205925	0.510673	0.8083	0.40391	2-Bromoacetaldehyde	21.42	-	-	-	299.1735	121.943957
NEG02511	191760.3227	215785.3426	0.170293	0.464882	0.7856	0.07263	alpha-D-Ribose 1,2-cyclic phosphate 5-phosphate	16.282937	-	-	-	297.6925	291.071879
NEG02516	5330941.452	2582734.647	-1.04549	0.020898	0.3487	1.88503	6-Oxo-2-hydroxy-7-(4'-chlorophenyl)-3,8,8-trichloroocta	-18.282784	-	-	-	296.9255	381.008639
NEG02524	2312960.257	2872254.046	0.312443	0.342164	0.7298	0.71025	Iodide	21.107946	-	-	-	294.98	125.899902
NEG02528	483180.972	559384.3984	0.211276	0.30791	0.7158	0.26784	Se-Methyl-L-selenocysteine	-5.1592021	-	-	-	293.8955	181.071584
NEG02537	855297.2089	900841.2996	0.074847	0.81038	0.9347	0.01957	12,13-epoxy-9-alkoxy-10E-octadecenoate	-0.7610185	-	-	-	288.5425	156.990603
NEG02540	75130.14335	72594.62855	-0.04953	0.813679	0.9353	0.02805	Perfluorohexane sulfonic acid	14.743217	-	-	-	287.339	399.113622

NEG02554	169055.7794	136048.8632	-0.31337	0.077084	0.5296	0.21381	Lyciumin A	23.154563	-	-	-	279.6495	872.920358
NEG02556	105779.5613	116116.3333	0.13451	0.701713	0.8994	0.00828	Sporidesmin	-19.936833	-	-	-	279.523	472.934174
NEG02559	668972.7009	721590.4934	0.109233	0.60855	0.8524	0.06143	3-Vinylbacteriochlorophyllide a	8.490181	-	-	-	279.31	615.987062
NEG02576	1636108.022	1261509.388	-0.37512	0.524903	0.8163	0.41771	Mazaticol hydrochloride	-24.084531	-	-	-	277.527	441.016977
NEG02579	37719788.52	34947824.91	-0.11012	0.176455	0.6362	2.06588	Radon-222	12.265456	-	-	-	277.281	220.995446
NEG02580	468288.254	400516.6776	-0.22553	0.509363	0.8082	0.21705	PE(14:0/18:0)	7.5945125	-	-	-	277.193	690.969979
NEG02581	2843161.782	2038121.873	-0.48026	0.571811	0.8362	0.4679	Ferrous lactate	-12.387586	-	-	-	277.019	232.974825
NEG02587	7085538.483	7256426.749	0.034382	0.913674	0.9715	0.23239	2-Iodophenol	-8.2730327	-	-	-	276.5315	218.998703
NEG02599	900091.3657	1243381.869	0.466126	0.357836	0.7353	0.50296	Radium-224	0.1643007	-	-	-	275.832	224.992761
NEG02603	254343.8984	260863.8461	0.036517	0.904339	0.9691	0.02412	3-(3,5-Diiodo-4-hydroxyphenyl)lactate	-20.271151	-	-	-	275.1865	432.950326
NEG02611	543574.11	655280.1596	0.269635	0.556696	0.8301	0.21426	Cryolite	0.2604434	-	-	-	272.377	208.934078
NEG02618	2210930.833	2280051.192	0.044412	0.91628	0.9721	0.17783	2-Bromoacetaldehyde	21.381759	-	-	-	271.3485	121.943952
NEG02630	3204773.728	3889023.673	0.279186	0.320331	0.7192	0.72388	Iodide	21.147486	-	-	-	270.5375	125.899907
NEG02646	1359234.094	1150332.813	-0.24074	0.390435	0.751	0.45348	12,13-epoxy-9-alkoxy-10E-octadecenoate	-0.6297369	-	-	-	266.061	156.990624
NEG02660	8660531.585	8046908.377	-0.10602	0.20428	0.6615	0.93047	Radon-222	12.294318	-	-	-	262.74	220.995453
NEG02663	4657733.142	1973494.939	-1.23888	0.021006	0.3489	1.89736	6-Oxo-2-hydroxy-7-(4'-chlorophenyl)-3,8,8-trichloroocta	-18.191888	-	-	-	260.055	381.008674
NEG02677	73545.68999	51620.28622	-0.5107	0.212614	0.6634	0.14677	YC 170	6.0524987	-	-	-	255.396	486.972977
NEG02691	3479230.273	2988393.433	-0.2194	0.371292	0.7415	0.57192	Iodide	21.267955	-	-	-	254.493	125.899922
NEG02701	2325017.142	2326116.266	0.000682	0.997585	0.999	0.09196	2-Bromoacetaldehyde	21.456504	-	-	-	249.909	121.943961
NEG02705	517854.3283	410207.1399	-0.33619	0.068286	0.5185	0.38796	Mazaticol hydrochloride	-24.112555	-	-	-	248.597	441.016965
NEG02716	2822551.765	3637621.479	0.365995	0.464434	0.7854	0.50446	2-Iodophenol	-8.1613131	-	-	-	247.127	218.998728
NEG02721	160385.4627	128767.2127	-0.31678	0.214681	0.6643	0.16451	Dehydroascorbide(1-)	22.803998	-	-	-	246.3115	172.097671
NEG02728	808431.7523	799166.0198	-0.01663	0.978012	0.994	0.01985	Ferrous lactate	-12.408296	-	-	-	244.675	232.97482

NEG02730	11595827	10546370.15	-0.13686	0.12343	0.5888	1.30785	Radon-222	12.31396	-	-	-	243.885	220.995457
NEG02735	1154291.982	1317812.442	0.191137	0.487356	0.799	0.15914	Radium-224	0.2915155	-	-	-	242.866	224.992789
NEG02745	574613.9562	553796.3108	-0.05324	0.79414	0.9291	0.06919	12,13-epoxy-9-alkoxy-10E-octadecenoate	-0.5339474	-	-	-	241.95	156.990639
NEG02756	1803873.717	2030732.228	0.170902	0.52934	0.8192	0.22308	6-Oxo-2-hydroxy-7-(4'-chlorophenyl)-3,8,8-trichloroocta	-18.171335	-	-	-	237.767	381.008682
NEG02774	2142910.242	1894195.433	-0.17799	0.200112	0.658	0.52376	Iodide	21.485477	-	-	-	229.745	125.89995
NEG02785	991377.3684	897130.5015	-0.14412	0.161564	0.6225	0.35041	Perflutren	-13.089783	-	-	-	228.875	187.009562
NEG02803	3744758.906	3803590.736	0.022489	0.944999	0.9818	0.09917	2-Bromoacetaldehyde	21.547197	-	-	-	226.3605	121.943973
NEG02823	2099045.144	1089199.494	-0.94646	0.021576	0.3502	1.24923	6-Oxo-2-hydroxy-7-(4'-chlorophenyl)-3,8,8-trichloroocta	-18.091571	-	-	-	221.683	381.008712
NEG02838	6622870.839	5955747.733	-0.15317	0.11797	0.5807	1.04847	Radon-222	12.507217	-	-	-	217.068	220.9955
NEG02846	450983.4292	425700.0168	-0.08324	0.712041	0.9019	0.12205	Tantalum	11.477225	-	-	-	211.115	179.9427
NEG02863	5675676.481	3364004.795	-0.75461	0.116883	0.5777	1.37217	6-Oxo-2-hydroxy-7-(4'-chlorophenyl)-3,8,8-trichloroocta	-18.093033	-	-	-	202.982	381.008711
NEG02874	1099175.985	907526.7133	-0.27641	0.421708	0.7663	0.32949	Mazaticol hydrochloride	-24.00898	-	-	-	199.424	441.017011
NEG02897	40339.64561	14866.19611	-1.44016	0.006903	0.2336	0.21354	Pyrazolate	-15.240568	-	-	-	196.33	438.298328
NEG02919	1599699.436	1178345.584	-0.44104	0.066793	0.5151	0.69614	Perflutren	-13.247736	-	-	-	193.783	187.009533
NEG02926	8420106.169	8477318.847	0.00977	0.960305	0.9879	0.01145	2-Bromoacetaldehyde	21.672424	-	-	-	193.1495	121.943988
NEG02939	1752454.162	1581926.281	-0.14769	0.627085	0.8608	0.27884	Radium-224	0.3883733	-	-	-	192.374	224.992811
NEG02940	1072997.354	1037197.193	-0.04896	0.801352	0.9311	0.01601	12,13-epoxy-9-alkoxy-10E-octadecenoate	-0.3395361	-	-	-	192.1945	156.99067
NEG02941	741946.2116	743617.6035	0.003246	0.981014	0.9946	0.04724	Violacene	8.1190758	-	-	-	192.158	353.921805
NEG02965	27072440.93	23394670.24	-0.21065	0.040957	0.44	2.5774	Radon-222	12.590537	-	-	-	190.423	220.995518
NEG02966	6893049.263	4663483.92	-0.56373	0.098905	0.5601	1.56519	2-Iodophenol	-7.9491283	-	-	-	190.4025	218.998775
NEG02968	1058843.926	1015220.062	-0.0607	0.629654	0.8619	0.18551	3-(3,5-Diiodo-4-hydroxyphenyl)lactate	-19.694969	-	-	-	190.341	432.950576
NEG02970	183308.9855	223600.0071	0.286643	0.565187	0.8334	0.15228	Chlorcyclizine	1.6021273	-	-	-	190.2625	299.819205
NEG02973	51270.02426	52846.98767	0.043706	0.959719	0.9877	0.01842	Triphenyltetrazolium chloride	11.918287	-	-	-	190.234	333.798914

NEG02994	2443224.902	3459764.504	0.501887	0.582682	0.8406	0.65741	Selenophosphate	-24.116948	-	-	-	188.988	158.938866
NEG03003	298784.4215	238904.4019	-0.32267	0.041089	0.44	0.33036	N-Carbamoyl glucuronide lorcaserin	22.719966	-	-	-	188.3025	408.906036
NEG03007	1857144.471	1724358.007	-0.10703	0.872855	0.9572	0.01457	Phosphoramide mustard	-19.797421	-	-	-	188.1925	220.010448
NEG03018	193205.8057	184364.2925	-0.06758	0.551261	0.8296	0.08163	2,3-Dibromo-1-propanol	2.6774156	-	-	-	187.8305	216.880407
NEG03055	445901.6972	382914.6681	-0.2197	0.037546	0.4241	0.33768	Clobenpropit	24.585954	-	-	-	186.6105	307.829916
NEG03065	1333628.987	1045769.332	-0.35079	0.650097	0.873	0.33308	Ferrous lactate	-12.331319	-	-	-	186.482	232.974838
NEG03076	168168.0885	134991.2975	-0.31704	0.033456	0.405	0.2311	1,1-Dibromo-3-iodo-2-propanone	16.683993	-	-	-	186.0075	340.766425
NEG03083	97058.53815	69265.83008	-0.48671	0.010526	0.2728	0.22308	Triadimenol	15.534792	-	-	-	185.8285	294.761918
NEG03101	541545.2252	454919.1929	-0.25147	0.231458	0.6747	0.31138	Dedimethylchlorpromazine	-4.2627724	-	-	-	185.346	289.802484
NEG03117	1249454.161	1016605.099	-0.29754	0.034447	0.4108	0.63411	Sodium arsenite	-19.467972	-	-	-	185.032	128.900394
NEG03118	114357.0708	100794.7443	-0.18213	0.152198	0.6145	0.13633	Chlordiazepoxide	0.1236229	-	-	-	184.974	298.74776
NEG03127	404153.422	375970.4238	-0.10428	0.403476	0.7578	0.1784	Quizalofop	-2.3302942	-	-	-	184.591	343.74102
NEG03137	76860.30735	52913.58462	-0.5386	0.01315	0.2953	0.19189	6-Hydroxy-8-hexacosanone	-0.1805652	-	-	-	184.294	395.682552
NEG03140	99615.67166	69413.75285	-0.52115	0.008266	0.2511	0.2365	17Z-hexacosenoic acid	22.270898	-	-	-	184.224	393.685513
NEG03148	728270.2003	659186.6988	-0.14379	0.109584	0.5692	0.33042	Gold	-4.0130045	-	-	-	184.075	195.958533
NEG03149	3345676.16	2887848.371	-0.2123	0.087977	0.5472	0.7965	Hydroiodic acid	-13.134351	-	-	-	184.062	126.903443
NEG03152	46579.06933	83874.82632	0.848556	0.163392	0.625	0.20709	Quinethazone	21.735472	-	-	-	183.956	288.737621
NEG03162	107432.7609	93595.76131	-0.19892	0.297081	0.7143	0.11728	Methoxyflurane	-20.045082	-	-	-	183.605	163.955517
NEG03207	2392429.32	2162078.719	-0.14606	0.052832	0.4813	0.60341	3-(Dichloromethylene)-2,5-pyrrolidinedione	-20.930973	-	-	-	182.677	178.977956
NEG03218	86618.632	79193.42909	-0.1293	0.604464	0.8498	0.08194	Vadium pentoxide	-10.360507	-	-	-	181.892	180.870839
NEG03229	53343.83054	59025.37541	0.146014	0.60751	0.8516	0.05647	Verruculogen	-15.904703	-	-	-	181.553	510.551387
NEG03232	71311.61452	76922.1518	0.109262	0.702193	0.8994	0.03002	3'-O-(4-Benzoyl)benzoyl ATP	8.1532173	-	-	-	181.4805	714.391656
NEG03242	88955.95314	84813.10053	-0.0688	0.710824	0.9019	0.05464	Nomilin	5.8985679	-	-	-	181.333	513.559959

NEG03244	1317584.219	1161617.788	-0.18176	0.171144	0.631	0.44663	2-Bromoacetaldehyde	21.201554	-	-	-	181.281	121.94393
NEG03249	3873661.321	3530547.111	-0.13381	0.18035	0.6397	0.5161	Iodide	21.82054	-	-	-	181.272	125.899993
NEG03252	133094.8858	113578.8026	-0.22876	0.453551	0.7814	0.1361	Sophoranone	24.466973	-	-	-	181.271	459.608393
NEG03254	174528.8038	112779.7417	-0.62996	0.022277	0.354	0.32643	Deoxytubulosine	-12.612918	-	-	-	181.266	458.609926
NEG03256	57608.10249	57824.90647	0.005419	0.994008	0.9985	0.00146	4,4'-Diapolycopene	20.760875	-	-	-	181.238	399.639641
NEG03257	388737.2503	355156.4453	-0.13034	0.259589	0.6904	0.1983	Linoelaidyl carnitine	14.801288	-	-	-	181.238	422.628094
NEG03261	152832.3448	127327.6627	-0.2634	0.267387	0.6945	0.15227	ID14326	-22.426262	-	-	-	181.216	507.656316
NEG03263	385758.981	367786.2201	-0.06883	0.936133	0.9789	0.1287	(25R)-3beta,4beta-dihydroxycholest-5-en-26-oate(1-)	-4.0202868	-	-	-	181.096	430.628988
NEG03264	75742.81769	75254.77931	-0.00933	0.97504	0.9931	0.02977	Vardenafil	-13.105352	-	-	-	181.0945	487.58932
NEG03268	167948.6873	152711.6492	-0.13721	0.601098	0.8483	0.09193	6-(4-{5,7-dihydroxy-3-[(3,4,5-trihydroxyoxan-2-yl)oxy]-5H-chromen-2	3.1330815	-	-	-	181.076	594.480589
NEG03271	125965.8352	105575.7784	-0.25475	0.306292	0.7152	0.1358	3-Sulfodeoxycholic acid	21.701098	-	-	-	181.066	457.611676
NEG03272	68034.8321	58257.9214	-0.22382	0.460461	0.7836	0.06955	Iriomoteolide 1a	-9.1231196	-	-	-	181.051	505.659401
NEG03280	274583.0421	456871.2835	0.734545	0.4305	0.77	0.35319	Imperialine	9.2150667	-	-	-	180.951	428.631883
NEG03281	181183.9797	149735.6326	-0.27504	0.031979	0.4009	0.22173	7alpha-Hydroxy-3-oxo-4-cholestenoate	19.990014	-	-	-	180.951	429.630332
NEG03282	436352.3923	415724.8569	-0.06986	0.429006	0.7695	0.12204	Bensultap	24.031888	-	-	-	180.951	430.615996
NEG03283	89393.47239	83925.59514	-0.09106	0.712435	0.9019	0.06692	Phylloquinone	21.027724	-	-	-	180.951	449.697901
NEG03285	204862.5059	171842.0484	-0.25357	0.139237	0.6058	0.20347	LysoPC(15:0)	-19.365998	-	-	-	180.9495	480.586897
NEG03288	106222.9514	104728.0408	-0.02045	0.971259	0.9918	0.00416	Cellulose triacetate	11.338471	-	-	-	180.9405	536.521818
NEG03289	73054.04968	75628.40447	0.049964	0.925544	0.9755	0.01286	Myxochelin C	-15.243859	-	-	-	180.938	538.518399
NEG03291	83863.42501	73288.51142	-0.19445	0.748914	0.9146	0.04701	Ergosterol	11.162614	-	-	-	180.934	395.645551
NEG03293	309326.431	231714.7314	-0.41678	0.04797	0.4632	0.35205	N-Oleoyl phenylalanine	16.706538	-	-	-	180.933	428.644901
NEG03294	187651.8195	157084.514	-0.25652	0.489785	0.7998	0.10818	Delphinidin 3-O-beta-D-sambubioside	-23.454176	-	-	-	180.933	596.47761
NEG03298	624588.7949	545588.5922	-0.19509	0.786496	0.927	0.18359	Glycolithocholate	-8.7969433	-	-	-	180.92	432.612809

NEG03300	154406.2111	148421.1682	-0.05703	0.767111	0.9214	0.05373	D1927	20.613273	-	-	-	180.9175	455.601435
NEG03302	86119.66133	70455.99052	-0.28962	0.418689	0.7657	0.11947	Glycodeoxycholate	-7.170971	-	-	-	180.917	448.612799
NEG03303	626712.9021	434584.9062	-0.52817	0.022643	0.3561	0.59885	Isomigrastatin	14.695894	-	-	-	180.917	488.601019
NEG03306	1214319.494	994918.2005	-0.2875	0.108611	0.5681	0.6046	Peimine	-4.6765766	-	-	-	180.8335	430.641805
NEG03309	729795.7996	657280.4792	-0.15098	0.159847	0.6215	0.30658	Mirex	0.8338834	-	-	-	180.8115	544.536178
NEG03310	585957.4085	548810.6452	-0.09449	0.262674	0.6915	0.20342	OA-6129 E	-2.9111829	-	-	-	180.808	488.574198
NEG03313	1085566.574	1019987.983	-0.0899	0.32028	0.7192	0.25119	Imperialine	-20.805562	-	-	-	180.79	428.618985
NEG03314	790253.9394	670949.6909	-0.23611	0.093392	0.5568	0.42251	2-amino-4-({1-[(carboxymethyl)-C-hydroxycarbonimidoyl]-2	13.108703	-	-	-	180.79	542.539849
NEG03316	150467.2391	132417.4085	-0.18436	0.25556	0.6877	0.14151	1-(7Z,10Z,13Z,16Z-docosatetraenoyl)-glycero-3-phosphate	0.5409878	-	-	-	180.7785	485.578987
NEG03320	136013.1319	124717.6306	-0.12508	0.796414	0.9301	0.01939	(3beta,5alpha,6alpha,7alpha,22E,24R)-5,6-Epoxyergosta-8,14,22	-1.0216787	-	-	-	180.755	425.623588
NEG03327	735936.5363	564514.3608	-0.38257	0.183036	0.6419	0.45168	Syringolin A	11.39833	-	-	-	180.6375	492.59475
NEG03329	262610.241	108685.3436	-1.27277	0.260944	0.6904	0.45153	N2'-Acetylgentamicin C1a	-1.1525437	-	-	-	180.634	490.571057
NEG03332	126380.9479	120864.7804	-0.06439	0.820696	0.9386	0.02499	20-Hydroxyleukotriene E4	5.30041	-	-	-	180.628	454.603138
NEG03338	170641.1752	142698.4561	-0.258	0.507765	0.8067	0.12215	Sipatrigine	-2.3929662	-	-	-	180.624	371.671832
NEG03341	304644.9861	232174.357	-0.39192	0.021648	0.3502	0.37289	Colchicoside	-19.627857	-	-	-	180.614	546.532976
NEG03346	101030.8654	109498.007	0.116109	0.788487	0.9278	0.03236	3-Aza-A-homocholest-4a-en-4-one	-9.8262067	-	-	-	180.584	398.641096
NEG03352	69897.57581	59079.54008	-0.24258	0.367027	0.7403	0.11779	Gitogenin	-3.4664414	-	-	-	180.327	431.627124
NEG03353	4877286.255	4898286.982	0.006199	0.949351	0.9832	0.0456	Isazofos	-18.82664	-	-	-	180.316	312.728117
NEG03354	79743.83553	76402.54207	-0.06175	0.730096	0.9083	0.02463	2-amino-4-({1-[(carboxymethyl)-C-hydroxycarbonimidoyl]-2	23.557715	-	-	-	180.309	500.554539
NEG03355	1040736.606	807116.4786	-0.36676	0.189167	0.6466	0.56323	Benzamil	7.5831045	-	-	-	180.308	318.744748
NEG03356	1295915.592	1108426.388	-0.22546	0.045315	0.4507	0.54831	Flamprop-M	-23.455286	-	-	-	180.307	320.715877
NEG03359	8643382.189	8396190.719	-0.04186	0.654916	0.8746	0.24173	Pyrithione zinc	23.901296	-	-	-	180.3	316.722017
NEG03361	24270858.81	21779030.33	-0.15629	0.406015	0.7584	1.33848	Clobenzorex	-12.366128	-	-	-	180.287	258.763511

NEG03372	8597841.087	8159504.3	-0.07549	0.634158	0.865	0.4086	Dehydrogenated ticlopidine	-8.7481791	-	-	-	179.9945	260.760433
NEG03373	36999765.31	35335191.7	-0.06641	0.712513	0.9019	0.60681	Orbencarb	-21.654941	-	-	-	179.992	256.766641
NEG03384	16270718.21	13419328.38	-0.27797	0.161855	0.6225	1.98674	Ticlopidine	19.169123	-	-	-	179.901	262.78348
NEG03421	1220943.338	1412774.523	0.210535	0.496544	0.8016	0.21175	(1S,3R)-3-(2,2-Dichloroethenyl)-2,2-dimethylcyclopropanecarb	-6.7902133	-	-	-	179.0355	208.061104
NEG03459	2031096.533	910042.7936	-1.15825	0.041895	0.4405	1.20778	L-Selenocysteine	0.0178123	-	-	-	178.343	167.045926
NEG03462	59660.75672	35574.84748	-0.74592	0.057139	0.4899	0.20009	Melleolide K	-16.936785	-	-	-	178.321	433.895357
NEG03469	483507.7902	452462.0179	-0.09574	0.454768	0.7814	0.19584	Dieldrin	16.361899	-	-	-	178.078	379.908256
NEG03472	49333.39138	45608.30246	-0.11327	0.62403	0.8594	0.06492	Moxifloxacin hydrochloride	8.4551183	-	-	-	178.075	436.888726
NEG03483	327891.9687	262400.6597	-0.32145	0.137319	0.6023	0.27714	Tetrafluoroethylene	9.5112135	-	-	-	177.751	99.0086747
NEG03500	350181.0496	185754.3018	-0.91471	0.041081	0.44	0.50387	Cacodylate	5.4728362	-	-	-	176.796	136.990879
NEG03514	35604.17649	20981.22344	-0.76295	0.094867	0.5568	0.15542	3'-(2'',3''	12.344043	-	-	-	175.875	633.496356
NEG03533	265665.4176	261487.1452	-0.02287	0.922866	0.9741	0.05865	Pulvomycin	-8.7784346	-	-	-	174.837	838.004458
NEG03535	443166.8577	424504.8409	-0.06207	0.775322	0.9235	0.12727	Potassium dichromate	-6.8302594	-	-	-	174.608	293.175314
NEG03546	139816.4218	149287.7171	0.094562	0.72273	0.9063	0.09721	Lucyoside M	15.527262	-	-	-	173.7705	836.00742
NEG03563	323056.6911	339961.2559	0.073583	0.730777	0.9083	0.00249	2,4-Dioxotetrahydropyrimidine D-ribonucleotide	-19.042432	-	-	-	172.866	325.183712
NEG03570	19808.99744	14931.9455	-0.40775	0.632221	0.8636	0.07411	4-Pyridoxate	3.2501825	-	-	-	172.568	182.154719
NEG03595	2040217.509	2197975.512	0.107452	0.732348	0.9091	0.23569	Selenophosphate	-24.330836	-	-	-	170.61	158.938832
NEG03600	312400.8489	1417704.288	2.182086	0.118655	0.5808	1.06149	Nornitrogen mustard	6.0253707	-	-	-	170.519	141.020579
NEG03643	8816677.161	8200751.28	-0.10448	0.602996	0.8493	0.72832	Cryolite	1.0978003	-	-	-	168.631	208.934254
NEG03646	79308.73014	64808.61625	-0.29129	0.219049	0.6675	0.13627	Aluminoparaaminosalicylate calcium	-20.33824	-	-	-	168.185	403.3057
NEG03648	602983.5579	558828.5327	-0.10971	0.637265	0.8668	0.22667	Epoxicozole	22.434341	-	-	-	168.101	328.756121
NEG03649	471579.9251	389990.5173	-0.27406	0.192268	0.65	0.26226	Dichlobenil	-0.4214968	-	-	-	168.013	171.004051
NEG03673	541623.1184	541021.0762	-0.0016	0.986819	0.997	0.0651	Dieldrin	16.777146	-	-	-	166.005	379.908414

NEG03690	250200.8135	212928.0537	-0.23272	0.097652	0.5586	0.22736	Quinclorac	-20.07698	-	-	-	164.7585	241.046064
NEG03714	128627.3601	102988.5539	-0.32071	0.121588	0.5846	0.18793	Bromobenzene-2,3-dihydrodiol	-8.2853511	-	-	-	164.0775	190.013741
NEG03718	184682.3664	168495.226	-0.13234	0.337062	0.7276	0.13698	dCTP	-18.860661	-	-	-	163.907	466.140813
NEG03748	161825.4427	145040.1334	-0.15799	0.59084	0.8433	0.11106	8-Bromoadenosine	8.4554696	-	-	-	162.921	345.13305
NEG03755	4512352.65	3918797.911	-0.20347	0.206166	0.6619	0.95157	Iodide	22.207631	-	-	-	162.78	125.900042
NEG03757	9568.645966	139907.0081	3.87001	0.326658	0.7209	0.3065	UDP-glucurote	9.4230002	-	-	-	162.722	579.283491
NEG03760	312668.4438	252667.3366	-0.30739	0.279936	0.7002	0.24479	Perfluorohexane sulfonic acid	-2.9480032	-	-	-	162.6405	399.106544
NEG03762	1272936.712	1385901.335	0.122664	0.467574	0.7887	0.13287	Deoxyribose triphosphate	-24.723772	-	-	-	162.602	373.053675
NEG03770	1022762.672	1503920.09	0.556257	0.428215	0.7691	0.56605	Dichlormid	-2.9784538	-	-	-	162.4685	207.077104
NEG03778	174710.5451	185411.1954	0.085762	0.807423	0.934	0.07175	6-(2,3-dihydroxybenzoyloxy)-3,4,5-trihydroxyoxane-2	-14.768776	-	-	-	162.355	329.232846
NEG03785	9761883.089	8113609.789	-0.26682	0.003042	0.1506	1.81247	Ticlopidine	19.390184	-	-	-	162.2045	262.783538
NEG03799	129588.7667	117073.8445	-0.14652	0.434299	0.77	0.09265	(6-carboxy-3,4,5-trihydroxyoxan-2-yl)({4-[7-(3,4-dihydroxy-5	7.0017508	-	-	-	162.021	608.511991
NEG03806	1092361.455	952236.3337	-0.19806	0.024184	0.3593	0.50285	Benzamil	8.1689287	-	-	-	161.879	318.744935
NEG03811	188431.6589	156610.102	-0.26686	0.03453	0.4108	0.20586	Jadomycin B	-8.3736285	-	-	-	161.822	548.556522
NEG03812	468522.1925	384154.3671	-0.28643	0.093365	0.5568	0.35524	Lead arsete	-8.3337007	-	-	-	161.816	346.116931
NEG03819	133667.9448	28998.23511	-2.20462	0.273365	0.6991	0.37618	7-Methylguanosine 5'-phosphate	24.113797	-	-	-	161.8065	377.256945
NEG03823	104942.8881	85155.74166	-0.30143	0.201851	0.6603	0.15239	({6-[5,7-dihydroxy-4-oxo-8-(3,4,5-trihydroxyoxan-2-yl)-2-(2,4,5	1.3334603	-	-	-	161.7665	675.543626
NEG03825	758449.9952	785430.5476	0.05043	0.912005	0.9709	0.05168	Isazofos	-18.405612	-	-	-	161.716	312.728249
NEG03827	151442.8067	123385.6972	-0.2956	0.152449	0.6145	0.18519	2-amino-4-({1-[(carboxymethyl)-C-hydroxycarbonimidoyl]-2	-16.902685	-	-	-	161.68	550.5534
NEG03835	309404.2812	250210.8183	-0.30635	0.041181	0.44	0.28274	Peimine	-4.276257	-	-	-	161.574	430.641978
NEG03838	1543279.916	695378.4165	-1.15013	0.019868	0.3429	1.18116	Dehydrogenated ticlopidine	-8.2194156	-	-	-	161.504	260.760572
NEG03839	2350692.218	3856119.428	0.714064	0.157004	0.6188	1.33475	Clobenzorex	-11.90136	-	-	-	161.496	258.763632
NEG03841	134796.4852	106638.7465	-0.33805	0.037077	0.4235	0.20462	Antimony trisulfide	15.57025	-	-	-	161.491	338.713013

NEG03846	775129.1637	671669.3544	-0.20669	0.310663	0.7175	0.35345	Midazolam	6.025994	-	-	-	161.466	324.761986
NEG03847	182451.112	161849.6696	-0.17286	0.218016	0.6665	0.12616	Heterocladol	0.5768356	-	-	-	161.4265	336.716218
NEG03852	1462510.108	961269.2586	-0.60543	0.144514	0.6122	0.58031	Pyrithione zinc	24.281918	-	-	-	161.409	316.722138
NEG03853	58514.48745	47511.20536	-0.30053	0.417229	0.7646	0.09572	({6-[2-(3,4- dihydroxyphenyl)-5,7- dihydroxy-4-oxo-3-[3,4,5	-19.913342	-	-	-	161.396	689.558972
NEG03855	230541.8967	212079.9477	-0.12042	0.404303	0.7582	0.104	Isomigrastatin	14.268602	-	-	-	161.372	488.600809
NEG03866	3572769.013	5284208.219	0.564645	0.147968	0.6145	1.414	Orbencarb	-21.246178	-	-	-	161.169	256.766747
NEG03867	65657.66508	46695.41624	-0.49168	0.05723	0.4899	0.16849	DAMGO	19.269826	-	-	-	161.169	512.58862
NEG03888	128636.2321	113695.8818	-0.17812	0.492585	0.8005	0.10874	Neoacrimarine K	20.231441	-	-	-	161.003	558.567344
NEG03890	56632.419	43757.34635	-0.3721	0.282821	0.7021	0.08131	1-O-Galloyl-beta-D-glucose	-12.918208	-	-	-	160.952	331.248531
NEG03893	674863.73	592483.1012	-0.18782	0.133381	0.5988	0.36002	Octachlorostyrene	-4.5900545	-	-	-	160.936	378.700581
NEG03899	330099.4214	265125.753	-0.31622	0.147352	0.6145	0.27876	Acrimarine N	-23.225445	-	-	-	160.8425	556.570173
NEG03907	255788.7002	234615.2285	-0.12466	0.520818	0.8144	0.13639	Dimethemid	15.039606	-	-	-	160.823	274.791671
NEG03909	295669.015	266069.9902	-0.15218	0.404524	0.7582	0.17108	Mibefradil	-6.885662	-	-	-	160.813	494.618011
NEG03918	132556.0767	90585.07577	-0.54926	0.04398	0.4477	0.25779	Cyanidin 3-(6-p-caffeoyl)glucoside	24.61606	-	-	-	160.759	610.534777
NEG03919	42421.34102	36711.7219	-0.20855	0.602979	0.8493	0.04792	TG(16:1(9Z)/22:2(13Z,16Z)/o-18:0)	22.400711	-	-	-	160.748	898.536873
NEG03921	111855.0648	130993.0322	0.227859	0.581321	0.8399	0.05512	Zygadenine	-9.3020869	-	-	-	160.74	492.620932
NEG03923	115524.0489	97622.06264	-0.24291	0.182561	0.6419	0.15287	12-Hydroxy-12-octadecanoylcarnitine	-5.9096475	-	-	-	160.73	442.650402
NEG03929	43408.54381	29310.48238	-0.56656	0.116123	0.5757	0.14732	Mycalamide B	12.149031	-	-	-	160.6985	516.608612
NEG03949	30190.91226	19656.29343	-0.61912	0.017526	0.3285	0.1333	Protopaxadiol	22.680095	-	-	-	160.504	459.735273
NEG03955	247636.5498	208024.5079	-0.25147	0.421054	0.7661	0.20264	Amprevir	18.540537	-	-	-	160.443	504.628998
NEG03963	210752.2159	224316.4738	0.089988	0.806348	0.9333	0.01495	TG(18:1(11Z)/22:2(13Z,16Z)/o-18:0)	-1.7914797	-	-	-	160.294	926.569062
NEG03965	99858.84332	71779.45496	-0.47632	0.10845	0.5681	0.18941	Lupeol acetate	4.3125962	-	-	-	160.283	467.748845
NEG03968	44170.95381	28445.18243	-0.63491	0.015137	0.3098	0.16876	C-13(2)-Carboxypyropheophorbide a	-1.7820673	-	-	-	160.1985	577.649292

NEG03971	131882.6175	109835.9319	-0.2639	0.436251	0.7718	0.15422	(24E)-15alpha-Acetoxy-3alpha-hydroxy-23-oxo-7,9(11),24-lanostatrien	20.533114	-	-	-	160.181	525.707538
NEG03977	109222.5472	177496.7149	0.700522	0.117178	0.5782	0.25463	Zn-Bacteriochlorophyll a	-24.809474	-	-	-	160.1455	951.57739
NEG03980	50005.18003	52413.99347	0.067874	0.791693	0.9284	0.01078	7-Chloro-6-demethylcepharadione B	-5.4981916	-	-	-	160.127	340.735844
NEG03985	92726.99032	80089.09278	-0.21138	0.531592	0.8207	0.10822	11-[(2R,3S)-3-[2-amino-3-methyl-4-(2-methyl-1,3-thiazol-4-yl)but-3-en-1	-3.9947998	-	-	-	160.11	523.710627
NEG03986	165773.2211	132011.613	-0.32855	0.301981	0.7148	0.19188	2-amino-4-({1-[(carboxymethyl)-C-hydroxycarbonimidoyl]-2	-22.16921	-	-	-	160.11	560.590273
NEG03989	142712.0792	165239.729	0.211453	0.58106	0.8399	0.0553	TG(18:0/22:4(7Z,10Z,13Z,16Z)/o-18:0)	-2.097152	-	-	-	160.0855	924.552782
NEG03991	72439.29349	75020.28284	0.050508	0.856447	0.9531	0.00031	(22S)-Acetoxy-3alpha,15alpha-dihydroxylanosta	-15.206137	-	-	-	160.061	527.704584
NEG04000	80591.47714	29670.02655	-1.44162	0.010537	0.2728	0.27621	Sanguisorbic acid dilactone	8.0726873	-	-	-	160.003	469.29282
NEG04001	79684.60457	65428.32996	-0.28439	0.339319	0.7277	0.12139	Glucoarabin	-12.969143	-	-	-	159.9605	506.62574
NEG04002	2845637.044	2530715.147	-0.16921	0.008388	0.2511	0.79037	Hydroiodic acid	-12.759396	-	-	-	159.9545	126.903491
NEG04004	43403.88064	55183.91176	0.346424	0.480034	0.7955	0.04938	TG(20:1(11Z)/o-18:0/20:1(11Z))	3.1589146	-	-	-	159.901	928.56636
NEG04006	1578957.243	1427615.905	-0.14536	0.028592	0.378	0.50183	Sodium arsenite	-18.310485	-	-	-	159.865	128.900545
NEG04010	39008.11157	26945.23032	-0.53374	0.197028	0.6557	0.12597	Pancuronium	7.9352869	-	-	-	159.854	571.859169
NEG04027	31368.70584	22030.66025	-0.50981	0.204839	0.6619	0.1128	Ziram	15.798616	-	-	-	159.539	304.839455
NEG04035	133256.1926	101919.9475	-0.38677	0.36456	0.7397	0.16562	TG(15:0/20:4(5Z,8Z,11Z,14Z)/24:1(15Z))	19.325261	-	-	-	159.522	950.567112
NEG04037	65496.37814	105127.3412	0.682651	0.150591	0.6145	0.17993	TG(15:0/20:3(5Z,8Z,11Z)/24:1(15Z))	18.200753	-	-	-	159.52	952.582079
NEG04040	204233.6962	156083.7492	-0.3879	0.186615	0.6464	0.19506	Gold	-2.998272	-	-	-	159.428	195.958733
NEG04054	3430979.636	2786720.148	-0.30005	0.084209	0.5411	0.91872	2-Bromoacetaldehyde	21.724904	-	-	-	159.1215	121.943994
NEG04058	454011.4666	394448.021	-0.20289	0.022392	0.354	0.33775	Dedimethylchlorpromazine	-3.6858693	-	-	-	159.1215	289.802652
NEG04065	215887.2258	184745.2865	-0.22474	0.133343	0.5988	0.21383	Selenophosphoric acid	-1.6465344	-	-	-	159.06	159.948258
NEG04081	33557.33581	41395.25113	0.302837	0.324497	0.7202	0.05276	Castasterone	16.647054	-	-	-	158.869	463.678159
NEG04087	46335.71055	36039.2022	-0.36256	0.226765	0.671	0.10492	Gorgostane	24.039629	-	-	-	158.751	411.736545
NEG04088	200398.8181	178750.5767	-0.16493	0.012854	0.2932	0.20363	Tetrachlorohydroquinone	-9.5511001	-	-	-	158.717	246.881256

NEG04090	37439.36116	32345.20926	-0.211	0.458201	0.7822	0.06566	6alpha-Hydroxy-castasterone	-23.882123	-	-	-	158.713	465.675178
NEG04102	144172.4499	128049.373	-0.1711	0.035507	0.4191	0.15589	Dichloroacetonitrile	24.239935	-	-	-	158.5455	108.937388
NEG04104	454409.6242	403835.7044	-0.17022	0.339027	0.7277	0.23677	Panaxydol chlorohydrin	-17.687099	-	-	-	158.5455	295.819473
NEG04106	54249.31491	53225.69383	-0.02748	0.902373	0.968	0.03517	Tiron	-6.6522061	-	-	-	158.5425	313.191333
NEG04114	152475.8881	81389.09356	-0.90567	0.524213	0.8163	0.14936	Calcium iodide	1.9530136	-	-	-	158.515	292.880197
NEG04138	490442.3836	492125.9139	0.004944	0.979865	0.9946	0.03685	3-(Dichloromethylene)-2,5-pyrrolidinedione	-20.32721	-	-	-	157.924	178.978065
NEG04140	115142.2898	103678.1451	-0.15131	0.510726	0.8083	0.10685	Thenylchlor	24.785887	-	-	-	157.888	322.83835
NEG04151	37551.30197	58247.5315	0.633334	0.25257	0.6865	0.1068	TG(20:0/22:6(4Z,7Z,10Z,13Z,16Z,19Z)/o-18:0)	21.595012	-	-	-	157.5995	948.59723
NEG04158	131990.8176	139486.5529	0.079688	0.815168	0.9361	0.00439	TG(18:4(6Z,9Z,12Z,15Z)/24:1(15Z)/o-18:0)	20.898416	-	-	-	157.33	950.61261
NEG04181	5588307.856	5092588.692	-0.13401	0.339399	0.7277	0.59302	Radon-222	13.069695	-	-	-	156.7485	220.995625
NEG04183	8851981.532	6431807.786	-0.46078	0.024246	0.3595	1.99641	N-(3,5-Dichlorophenyl)succinimide	-20.061	-	-	-	156.68	243.061927
NEG04186	1723023.545	1531237.026	-0.17024	0.360714	0.7368	0.406	2-Iodophenol	-7.5060993	-	-	-	156.6335	218.998872
NEG04205	483155.0988	439042.0588	-0.13813	0.482621	0.7977	0.16177	Radium-224	0.7635647	-	-	-	156.4045	224.992896
NEG04216	151988.6277	96236.97641	-0.6593	0.035806	0.4191	0.28672	Stearyltrimethylammonium chloride	13.11971	-	-	-	156.34	347.04689
NEG04224	4245447.066	1589835.462	-1.41704	0.060339	0.4986	1.91284	Phosphoramide mustard	-19.557617	-	-	-	156.273	220.010501
NEG04228	184776.2812	172452.73	-0.09958	0.794868	0.9292	0.04563	Perflutren	-12.276806	-	-	-	156.238	187.009715
NEG04237	916634.8447	835896.5716	-0.13302	0.283543	0.7021	0.23532	6-Oxo-2-hydroxy-7-(4'-chlorophenyl)-3,8,8-trichloroocta[illegible]	-18.130264	-	-	-	156.137	381.008697
NEG04240	685345.6793	493160.3324	-0.47478	0.045492	0.4515	0.47866	3,3',4,4'-Tetrachloroazobenzene	3.1715748	-	-	-	156.101	318.994738
NEG04257	3070608.702	2596922.622	-0.24172	0.071914	0.5226	0.79627	Butefine hydrochloride	22.106879	-	-	-	155.924	352.928648
NEG04275	238906.0745	203041.3069	-0.23467	0.488936	0.7996	0.13108	Triphosphate	-7.7935161	-	-	-	155.806	256.945713
NEG04276	421609.5626	311631.9697	-0.43606	0.005146	0.202	0.46743	Diphosphate	18.809035	-	-	-	155.804	176.971171
NEG04281	82694.17199	143874.8642	0.798957	0.286682	0.7033	0.19892	TG(22:1(13Z)/22:6(4Z,7Z,10Z,13Z,16Z,19Z)/o-18:0)	-2.5187903	-	-	-	155.7375	974.612266
NEG04285	241559.9417	173801.2182	-0.47494	0.012867	0.2932	0.34593	3,5-Dichloro-2-methylmucote	19.919782	-	-	-	155.7015	224.023306

NEG04287	36686.72998	43073.55076	0.231544	0.60904	0.8527	0.04027	TG(20:4(5Z,8Z,11Z,14Z)/24:1(15Z)/o-18:0)	-3.7220749	-	-	-	155.669	978.643077
NEG04298	154258.2635	163883.729	0.087325	0.787179	0.927	0.03455	Tetramethylrosamine	19.113606	-	-	-	155.592	390.913414
NEG04308	262672.6052	327554.4953	0.318469	0.602229	0.8489	0.19399	Cloxacillin	7.136936	-	-	-	155.318	434.877134
NEG04319	143740.3573	180689.1598	0.330045	0.375671	0.7438	0.12397	alpha-Amyrin tetratriacontanoate	10.871774	-	-	-	155.197	916.607299
NEG04320	162546.5446	198157.7617	0.285797	0.389134	0.7506	0.12437	TG(20:5(5Z,8Z,11Z,14Z,17Z)/24:1(15Z)/o-18:0)	-2.5174767	-	-	-	155.197	976.628262
NEG04333	447381.9479	489954.604	0.131141	0.406165	0.7584	0.09947	Ochratoxin C	-18.331527	-	-	-	154.982	430.850807
NEG04341	310586.5034	275118.8531	-0.17494	0.040663	0.4381	0.25031	2,6-Dichloroindophenol	7.1766234	-	-	-	154.86	267.090147
NEG04362	883252.0877	522096.8231	-0.75851	0.317487	0.7192	0.62524	11-cis-Retinyl-palmitate	-10.039136	-	-	-	154.445	157.977127
NEG04372	38845.01904	1	-15.2454	0.059398	0.4966	0.24829	UDP-2-deoxyglucose	10.028719	-	-	-	154.183	549.300642
NEG04375	1060110.302	831903.599	-0.34973	0.422483	0.7668	0.38848	12,13-epoxy-9-alkoxy-10E-octadecenoate	0.18879	-	-	-	154.062	156.990753
NEG04387	1134787.159	952954.8174	-0.25194	0.415663	0.7631	0.30022	3-(Dichloromethylene)-2,5-pyrrolidinedione	14.490237	-	-	-	153.772	178.984331
NEG04396	3053605.597	5672379.442	0.89344	0.228962	0.6731	1.54365	Phenolic phosphate	-11.42257	-	-	-	153.497	173.081835
NEG04418	1143869.265	1471509.469	0.363375	0.377871	0.745	0.35406	Glycerone phosphate	2.2345245	-	-	-	152.937	169.050903
NEG04448	1326341.859	1824542.053	0.460082	0.029381	0.3837	0.88608	5-Nitrofurfural	-18.64225	-	-	-	152.277	140.071693
NEG04458	1234173.452	1089125.784	-0.18037	0.661594	0.8779	0.15854	Cyperaquinone	-9.7923232	-	-	-	152.1055	241.217151
NEG04462	853849.4917	730510.0654	-0.22508	0.282916	0.7021	0.38121	Methyl 2-propenyl selenide	-12.193834	-	-	-	152.066	134.061076
NEG04465	15998792.65	18755999.54	0.229389	0.507192	0.8067	0.89292	Tetrafluoroethylene	-13.738868	-	-	-	152.0345	99.0063493
NEG04487	359961.0414	311418.3887	-0.20899	0.497732	0.8016	0.20829	beta-2,3,5,6-Tetrachloro-1,4-cyclohexanediol	-4.6464584	-	-	-	151.6715	252.930043
NEG04490	5487244.705	5306556.178	-0.04831	0.898173	0.9668	0.19986	Haloxydine	12.296797	-	-	-	151.6355	198.965482
NEG04497	476845.5251	448803.4485	-0.08744	0.735443	0.9109	0.14619	Dihydroxyfumarate	16.679343	-	-	-	151.5555	147.066193
NEG04498	2036064.198	1923058.209	-0.08238	0.874811	0.9576	0.31408	3,4-Dihydroxyphthalate	-21.016683	-	-	-	151.493	197.118159
NEG04501	2211051.6	2115373.134	-0.06382	0.906745	0.9697	0.29771	Barbiturate	-19.542132	-	-	-	151.398	127.07642
NEG04511	1847325.189	1498742.54	-0.30169	0.297326	0.7146	0.57602	3-hydroxy-2-methyl-2-[(sulfooxy)methyl]propanoic acid	14.485396	-	-	-	151.2595	213.185826

NEG04534	1287102.835	1198139.831	-0.10333	0.718916	0.9041	0.26788	5-Hydroxyisourate	0.6303617	-	-	-	150.952	183.102539
NEG04539	2683922.992	2862906.71	0.093137	0.704351	0.8994	0.10945	Glyphosate	4.0591743	-	-	-	150.881	168.06651
NEG04542	1237914.019	1260146.946	0.025681	0.927283	0.9759	0.05997	4-Maleylacetoacetate	-22.261574	-	-	-	150.858	199.133768
NEG04552	1942350.305	1939491.882	-0.00212	0.988684	0.9976	0.10876	Chlorthiamid	8.9745222	-	-	-	150.7485	205.086873
NEG04557	2775504.08	2664749.444	-0.05875	0.870944	0.9571	0.21242	2-Aminoadenosine	-1.5632319	-	-	-	150.573	281.248282
NEG04563	2913340.014	2488742.876	-0.22726	0.469708	0.7897	0.67248	2-Hydroxyethylphosphote	17.361915	-	-	-	150.452	125.043212
NEG04581	4919819.484	4707960.228	-0.0635	0.67651	0.8854	0.46453	Angelicin	-8.9417459	-	-	-	150.1975	185.154559
NEG04584	1051378.459	890957.3806	-0.23885	0.486222	0.7985	0.33683	2,3-Diketo-5-methylthiopentyl-1-phosphate	5.6186591	-	-	-	150.1515	241.180784
NEG04590	1271574.893	1074509.484	-0.24294	0.110491	0.5692	0.53527	2,4-Dioxotetrahydropyrimidine D-ribonucleotide	-18.43555	-	-	-	150.102	325.18391
NEG04599	6973095.595	7115000.937	0.029065	0.687556	0.8902	0.01755	Trimethylselenonium	0.4615224	-	-	-	150.057	123.056381
NEG04603	11627161.99	14526793.24	0.321217	0.553212	0.8299	0.56433	2,5-Dioxopentanoate	5.2094544	-	-	-	150	129.092101
NEG04606	3278888.446	3117606.633	-0.07277	0.793569	0.929	0.26517	Cacodylate	7.4316846	-	-	-	149.873	136.991149
NEG04613	1973880.481	4131060.98	1.065478	0.038821	0.4324	1.75701	sn-Glycerol 3-phosphate	-1.2217795	-	-	-	149.744	171.066213
NEG04624	11334842.83	10649216.55	-0.09002	0.623839	0.8594	0.79492	N-Benzoyl-4-hydroxyanthranilate	7.6044265	-	-	-	149.517	256.23608
NEG04633	5443542.051	4348503.504	-0.32403	0.075716	0.5279	1.37784	Diamidafos	13.511671	-	-	-	149.2395	199.170228
NEG04636	2681641.29	2359542.816	-0.18461	0.423601	0.7672	0.58152	Pemirolast	-5.9569222	-	-	-	149.207	227.201464
NEG04644	1759671.696	1512255.181	-0.2186	0.181542	0.6415	0.5502	3-(Phosphoacetylamido)-L-alanine	10.221078	-	-	-	149.086	241.118998
NEG04646	1495725.839	1526671.006	0.029543	0.925842	0.9755	0.03846	Meconic acid	11.174645	-	-	-	149.056	199.097459
NEG04653	500231.7228	509075.8256	0.025284	0.94094	0.9803	0.00709	Propyzamide	3.0691971	-	-	-	148.9735	255.12131
NEG04655	4269509.363	3185848.548	-0.42239	0.827726	0.9421	0.73316	Disodium malate	-19.250224	-	-	-	148.964	177.040396
NEG04660	6303493.169	5917015.684	-0.09128	0.763332	0.9201	0.66287	3-Fluorocyclohexadiene-cis,cis-1,2-diol-1-carboxylate	-6.1466709	-	-	-	148.788	173.118153
NEG04663	2598858.13	2544203.634	-0.03066	0.911934	0.9709	0.12121	(1S,3R)-3-(2,2-Dichloroethenyl)-2,2-dimethylcyclopropanecarb	-5.357074	-	-	-	148.692	208.061403
NEG04664	1594370.031	1443133.483	-0.14378	0.507373	0.8067	0.33688	Arbutin 6-phosphate	-9.5255468	-	-	-	148.677	351.220468

NEG04673	1160833.485	1567066.946	0.432906	0.107571	0.5681	0.53391	Bromobenzene	4.4548187	-	-	-	148.194	156.001323
NEG04678	1253649.298	1645951.176	0.392788	0.281032	0.7009	0.65078	O-Phosphorylhydroxylamine	13.126599	-	-	-	148.027	112.004007
NEG04693	13971294.67	13757375.23	-0.02226	0.873855	0.9573	0.2394	Glyoxylate	16.754525	-	-	-	146.955	73.0294638
NEG04703	147242.536	150901.116	0.035409	0.917447	0.9728	0.01256	Streptomycin 6-phosphate	-21.458635	-	-	-	146.09	660.532527
NEG04716	1963916.192	2784737.457	0.503808	0.368674	0.7408	0.86384	Ferrous lactate	-12.028175	-	-	-	144.719	232.974909
NEG04724	1568132.372	5269196.865	1.748536	0.049375	0.4711	1.88575	Dihydrogen phosphate	2.5261622	-	-	-	144.5895	95.9801684
NEG04736	399672.1041	387558.1416	-0.0444	0.85579	0.9527	0.10778	Sodium L-ascorbic acid 2-phosphate	21.370955	-	-	-	144.434	324.072971
NEG04750	74842.50711	54098.64873	-0.46827	0.016732	0.3228	0.1918	Norbadione A	-9.3228314	-	-	-	143.7205	677.494798
NEG04766	1117314.642	1008610.768	-0.14767	0.161236	0.6225	0.35388	2-({2-[4-(1,2-dihydroxyethyl)-5,11,12,13-tetrahydroxy-8	11.896253	-	-	-	142.4475	688.491926
NEG04775	61182.00186	55659.32915	-0.13648	0.543481	0.8268	0.04883	Agrocin 84	4.0101848	-	-	-	141.958	701.494941
NEG04781	144835.4891	90748.9522	-0.67446	0.06746	0.5163	0.24871	1-O-Galloyl-beta-D-glucose	-12.649365	-	-	-	141.6725	331.248621
NEG04784	385004.3229	331760.0186	-0.21473	0.093959	0.5568	0.25201	N(omega)-(ADP-D-ribosyl)-L-arginine	18.849753	-	-	-	140.975	714.50761
NEG04785	131219.1915	109830.7249	-0.2567	0.077332	0.5298	0.16146	[2,6-dihydroxy-4-({[3-hydroxy-6-(hydroxymethyl)-2,5	-16.624978	-	-	-	140.904	715.510811
NEG04786	118603.1871	81834.13445	-0.53537	0.010652	0.2735	0.25678	Dihydrostreptomycin 3'alpha,6-bisphosphate	-5.3748293	-	-	-	140.094	742.538427
NEG04796	11432188.03	11203913.34	-0.0291	0.855022	0.9527	0.24143	Nitrogen mustard	-1.6024249	-	-	-	137.662	155.045973
NEG04799	98963.2224	86040.61307	-0.20187	0.525314	0.8163	0.12707	Cyclohexa-1,5-diene-1-carbonyl-CoA	-12.934879	-	-	-	137.568	872.637523
NEG04805	1512500.843	1611776.871	0.091716	0.766723	0.9214	0.00106	2,4-DB	-14.014622	-	-	-	136.6185	248.079832
NEG04807	964040.0008	883376.2352	-0.12606	0.148675	0.6145	0.26591	(1E,3E)-4-Hydroxybuta-1,3-diene-1,2,4-tricarboxylate	10.416871	-	-	-	136.4295	201.113129
NEG04816	596861.3072	567101.249	-0.07379	0.479729	0.7954	0.15119	Disodium malate	-19.447941	-	-	-	135.717	177.040361
NEG04819	228482.2781	179438.2377	-0.34859	0.284508	0.7021	0.22579	5-Phosphoribosyl-4-carboxy-5-aminoimidazole	-8.3338094	-	-	-	134.462	335.163922
NEG04825	7396334.58	6372494.138	-0.21495	0.438107	0.7731	0.74434	Tetrafluoroethylene	-13.648064	-	-	-	133.715	99.0063584
NEG04827	2120160.974	2602253.006	0.295587	0.382874	0.7477	0.51044	3-Fluorocyclohexadiene-cis,cis-1,2-diol-1-carboxylate	-6.115696	-	-	-	133.451	173.118158
NEG04832	168397.6421	142291.0678	-0.24303	0.235981	0.6796	0.14626	(R)-5-Diphosphomevalote	24.199938	-	-	-	132.272	307.11708

NEG04836	380516.8391	259682.2785	-0.55121	0.015305	0.31	0.46125	2,4-Dihydroxypteridine	-9.2434786	-	-	-	131.691	163.112806
NEG04837	1842857.241	1472243.245	-0.32393	0.108567	0.5681	0.66014	Haloxydine	12.35717	-	-	-	131.567	198.965494
NEG04861	1222421.624	1164725.253	-0.06975	0.701962	0.8994	0.25226	2,4-Dioxotetrahydropyrimidine D-ribonucleotide	-18.473252	-	-	-	128.6225	325.183897
NEG04869	232481.1773	316446.7616	0.444849	0.081551	0.5377	0.31554	Chlorobenzilate	-0.9931497	-	-	-	127.732	324.179
NEG04881	292822.6868	287253.1595	-0.0277	0.955611	0.986	0.01533	Phosphocreatine	-11.012209	-	-	-	127.0325	210.103499
NEG04891	135978.0453	40351.71189	-1.75267	0.094079	0.5568	0.2893	Tetracozole	-16.666326	-	-	-	126.062	371.132121
NEG04896	548650.9855	565842.6055	0.044512	0.872424	0.9572	0.01952	thymidine 3'-monophosphate	10.612733	-	-	-	125.653	319.191122
NEG04901	1259733.399	1588270.423	0.334338	0.134328	0.5997	0.62094	Pydanon	16.114359	-	-	-	124.9415	187.133855
NEG04908	5534632.155	3833656.554	-0.52977	0.342961	0.7298	1.43226	2,4-DB	-13.975843	-	-	-	124.236	248.079842
NEG04912	552926.867	565714.3079	0.032985	0.903497	0.9686	0.03339	Arbutin 6-phosphate	-9.3073093	-	-	-	123.803	351.220545
NEG04918	442243.7948	282799.9262	-0.64506	0.12796	0.5924	0.49586	Dichlofenthion	5.7958302	-	-	-	123.006	314.14775
NEG04921	942628.3988	800185.6889	-0.23635	0.457702	0.7822	0.4095	Lamotrigine	15.281293	-	-	-	122.5735	255.087637
NEG04928	68365.13132	57943.06536	-0.23862	0.316923	0.7189	0.07097	2,3,4,7,8,9,15,22,23,28-decahydroxy-14-(hydroxymethyl)	19.59663	-	-	-	122.135	755.541549
NEG04942	3720260.363	3588699.364	-0.05194	0.911984	0.9709	0.18658	Dihydrogen phosphate	2.6200492	-	-	-	120.823	95.9801775
NEG04951	1115807.326	1273139.453	0.190303	0.111652	0.5702	0.41314	O-Phosphorylhydroxylamine	13.267964	-	-	-	120.219	112.004023
NEG04958	1500427.393	1666453.833	0.151408	0.222512	0.6692	0.47199	Cacodylate	7.6976542	-	-	-	119.803	136.991186
NEG04965	743365.7915	476130.315	-0.64272	0.229677	0.6734	0.45014	Butefine hydrochloride	22.113011	-	-	-	119.655	352.92865
NEG04975	849531.4596	869740.5049	0.033918	0.736751	0.9115	0.00233	Bromobenzene	4.4725728	-	-	-	119.2495	156.001326
NEG05000	457504.8253	371357.4459	-0.30098	0.474428	0.7921	0.24091	Selenomethionine Se-oxide	-6.5140875	-	-	-	117.591	211.097042
NEG05005	1274001.356	1332905.039	0.065207	0.418873	0.7657	0.18306	(1E,3E)-4-Hydroxybuta-1,3-diene-1,2,4-tricarboxylate	10.574468	-	-	-	117.486	201.113161
NEG05028	387589.0319	434567.3801	0.165052	0.392402	0.7513	0.16211	Shikimate 3-phosphate	-1.7516882	-	-	-	113.997	253.123478
NEG05032	1852346.347	1922231.498	0.053428	0.563104	0.8325	0.17858	3-Fluorocyclohexadiene-cis,cis-1,2-diol-1-carboxylate	-6.1153823	-	-	-	113.038	173.118159
NEG05040	1836048.2	1399987.333	-0.39119	0.00626	0.2196	0.90683	Pydanon	16.029015	-	-	-	112.6245	187.133839

NEG05044	1318679.327	1278948.064	-0.04414	0.686563	0.89	0.14437	5-Hydroxyisourate	0.4707611	-	-	-	112.5735	183.10251
NEG05045	876532.5527	119288.7911	-2.87735	0.011783	0.2831	1.14366	3',5'-Cyclic IMP	-23.8464	-	-	-	112.324	329.17555
NEG05046	260811.6494	139528.1126	-0.90245	0.323717	0.7192	0.24739	5,6-Dihydrouracil	16.388536	-	-	-	112.2975	113.097193
NEG05048	4259787.166	5443023.917	0.353627	0.231272	0.6747	0.87991	Tetrafluoroethylene	-13.533417	-	-	-	112.045	99.0063699
NEG05059	54571.63975	90080.03431	0.723056	0.352059	0.7325	0.18212	3'-Deoxydihydrostreptomycin 6,3''-bis	11.859471	-	-	-	110.874	726.551652
NEG05067	785408.895	643430.53	-0.28766	0.247723	0.6846	0.38385	Meconic acid	11.142537	-	-	-	109.85	199.097453
NEG05072	325836.7635	185288.2962	-0.81438	0.15315	0.6159	0.44917	3-Phospho-D-glycerate	-23.700788	-	-	-	109.499	185.045514
NEG05073	6265699.256	5981571.178	-0.06695	0.941182	0.9804	0.70704	Disodium malate	-18.495725	-	-	-	109.484	177.04053
NEG05074	77742.30779	57407.82735	-0.43745	0.252989	0.6865	0.12322	Epigallocatechin-(4beta->8)-epicatechin-3-O-gallate ester	-19.628576	-	-	-	109.3415	745.601968
NEG05082	149041.3652	122537.5829	-0.28249	0.114345	0.5743	0.18778	Delphinidin 5-O-beta-D-glucoside 3-O-beta-D-sambubioside	-24.281627	-	-	-	107.755	758.613778
NEG05092	1372572.659	963503.0486	-0.51052	0.242477	0.6822	0.71951	2-Amino-5-phosphopentanoic acid	6.7246557	-	-	-	106.715	196.120249
NEG05094	563412.2111	559483.0037	-0.0101	0.965772	0.9897	#######	2-Methacryloyloxyethyl phenyl phosphate	-12.274015	-	-	-	106.351	285.20691
NEG05095	390965.6528	349657.5255	-0.1611	0.451952	0.7814	0.19889	S-methyl-5-thio-D-ribulose 1-phosphate(2-)	11.669493	-	-	-	106.0215	257.175736
NEG05099	122370.4571	109660.5757	-0.15821	0.513864	0.811	0.10843	{[6-[(2-{3-[(6-carboxy-3,4,5-trihydroxyoxan-2-yl)oxyl-4-hydroxyphenyl}	-3.3044433	-	-	-	105.371	786.645121
NEG05100	534210.1368	161049.1521	-1.72991	0.013088	0.2953	0.83352	Purine mononucleotide	-24.447796	-	-	-	105.37	331.191202
NEG05102	8881521.248	7652732.087	-0.21483	0.159919	0.6215	1.3189	Trimethylselenonium	0.5274113	-	-	-	105.342	123.056389
NEG05103	845466.0571	662509.2614	-0.35181	0.217164	0.6662	0.49371	Formylphosphote	-19.558177	-	-	-	105.298	108.996472
NEG05104	1179340.913	1087135.652	-0.11745	0.658705	0.8769	0.29458	cis-(Homo)2-aconitate	-23.107891	-	-	-	105.1675	201.149452
NEG05105	240623.5872	175802.6255	-0.45282	0.129032	0.5925	0.24957	2,6-Dichlorophenolindophenol sodium salt	6.0234164	-	-	-	105.012	289.071771
NEG05107	1407097.279	1142745.753	-0.30022	0.395813	0.7526	0.46284	Diethylphosphoric acid	-14.626545	-	-	-	104.9965	153.091969
NEG05114	584492.788	569041.2459	-0.03865	0.899026	0.967	0.03509	Wedelolactone	-2.8989706	-	-	-	104.975	313.238212
NEG05117	214613.678	144754.4228	-0.56813	0.106103	0.5677	0.32273	Sevoflurane	18.45388	-	-	-	104.622	199.051215
NEG05118	86339.26526	79256.80457	-0.12348	0.523978	0.8163	0.0854	Delphinidin 3-lathyroside 5-(6-acetylglucoside)	-9.9156276	-	-	-	104.6215	800.660874

NEG05124	1731019.959	1420883.165	-0.28483	0.362016	0.7382	0.55291	4-Maleylacetoacetate	-21.796044	-	-	-	104.289	199.133861
NEG05125	1607398.516	1588814.091	-0.01678	0.949466	0.9832	0.06457	Dihydrogen phosphate	2.6252062	-	-	-	104.283	95.980178
NEG05128	523739.0535	879478.2185	0.7478	0.276882	0.6995	0.60091	4-nitrophenolate	10.021502	-	-	-	104.263	137.097107
NEG05150	191920.9148	186042.6098	-0.04488	0.959061	0.9873	0.10187	O-Phospho-L-serine	9.975715	-	-	-	102.882	184.06707
NEG05153	858329.4625	843740.6243	-0.02473	0.675334	0.8854	0.09804	Pydanon	16.121192	-	-	-	102.1725	187.133856
NEG05154	2339391.214	1459323.613	-0.68083	0.134714	0.5997	1.00939	Arbutin 6-phosphate	-9.4794733	-	-	-	101.8445	351.220484
NEG05169	271172.5396	114230.0439	-1.24727	0.000169	0.0947	0.56673	3-Deoxy-D-manno-octulosote 8-phosphate	-14.261213	-	-	-	100.477	317.160086
NEG05170	1358976.333	1380836.012	0.023022	0.859657	0.9535	0.01703	Fosetyl	-7.7684377	-	-	-	100.475	109.040768
NEG05174	1399701.756	108351.8176	-3.69132	0.014383	0.3082	1.53914	3',5'-Cyclic IMP	-23.804419	-	-	-	100.434	329.175563
NEG05187	146360.737	139184.2087	-0.07253	0.644051	0.8698	0.08297	Erythrityl Tetranitrate	3.7286932	-	-	-	98.7149	301.10385
NEG05188	678197.9596	635646.6435	-0.09348	0.446216	0.7781	0.20895	Bromobenzene	4.3779961	-	-	-	98.4107	156.001311
NEG05197	742996.3404	984640.5489	0.406242	0.039861	0.438	0.60104	3-O-Methylquercetin	-4.5895794	-	-	-	97.3345	315.253572
NEG05203	62888.05856	95258.46567	0.599061	0.254011	0.6867	0.14442	2-(3,4-dihydroxy-5-methoxyphenyl)-3,5,7-trihydroxy-1chromen-1	-18.515998	-	-	-	96.627	316.257049
NEG05210	1096958.959	1191186.63	0.11889	0.541498	0.8268	0.15739	2,4-Dioxotetrahydropyrimidine D-ribonucleotide	-18.217289	-	-	-	96.249	325.183981
NEG05225	698305.926	869973.7612	0.317113	0.345331	0.7309	0.29826	dTDP-4-dehydro-beta-L-rhamnose	-18.819733	-	-	-	93.85005	545.296142
NEG05227	157550.5668	225487.9236	0.517235	0.215449	0.6643	0.21911	CDP-4-dehydro-6-deoxy-D-glucose	9.3110913	-	-	-	93.68095	546.299519
NEG05228	114550.6777	135419.7343	0.241452	0.460197	0.7836	0.09205	Diflunisal	-19.012102	-	-	-	93.66315	249.185567
NEG05234	334097.5007	515589.501	0.625954	0.068343	0.5185	0.45784	Se-Methylselenomethionine	10.177104	-	-	-	91.768	210.135772
NEG05236	276941.6417	343377.9668	0.310215	0.367532	0.7403	0.16848	dTDP-6-deoxy-beta-L-talose	-18.430988	-	-	-	91.761	547.312217
NEG05238	465008.7571	442338.0711	-0.07211	0.343851	0.7301	0.1731	Fenpiclonil	6.6697484	-	-	-	91.4155	236.079005
NEG05241	1185721.178	301131.7908	-1.9773	0.03515	0.4158	1.18466	2,2-Bis(4-hydroxyphenyl)hexafluoropropane	1.9667176	-	-	-	91.0705	335.222485
NEG05246	371690.4583	420740.4576	0.178829	0.322794	0.7192	0.19659	Flupropate	23.675169	-	-	-	90.7169	145.036581
NEG05250	898606.0444	251436.4643	-1.83749	0.028115	0.3761	1.08056	Dichlofenthion	6.0000443	-	-	-	90.3733	314.147814

NEG05256	148950.3882	116229.9592	-0.35785	0.154684	0.6173	0.16464	2-(Hydroxymethyl)-3-(acetamidomethylene)succ[illegible]	-1.5283621	-	-	-	90.0367	216.168391
NEG05261	171218.2604	116954.8824	-0.54988	0.094256	0.5568	0.28299	Ellagic acid	-15.911076	-	-	-	89.8436	301.180515
NEG05273	3488295.292	5343876.112	0.615364	0.608823	0.8525	0.86958	5-Hydroxyisourate	0.3944486	-	-	-	88.9836	183.102496
NEG05274	378196.4376	458364.24	0.277359	0.616161	0.8567	0.13669	L-Threonylcarbamoyladenyl[illegible]	-10.536777	-	-	-	88.8089	491.322136
NEG05298	285972.8916	274668.5781	-0.05819	0.858118	0.9533	0.06431	CGH 2466	4.9756933	-	-	-	85.6852	321.206727
NEG05307	4445366.606	3413728.344	-0.38095	0.33194	0.7237	0.88425	Barbiturate	-19.507648	-	-	-	84.23065	127.076425
NEG05313	92678.52087	74315.13293	-0.31858	0.179114	0.6387	0.13251	Propane-1,2-diol 1-phosphate	10.852826	-	-	-	84.13205	155.068717
NEG05314	208489.9392	265563.3821	0.349078	0.264414	0.6923	0.16491	3-Phospho-D-glycerate	-23.802896	-	-	-	84.1247	185.045495
NEG05316	4586967.248	4086685.582	-0.16661	0.619239	0.8576	0.58311	sn-Glycerol 3-phosphate	0.4448725	-	-	-	84.1234	171.0665
NEG05320	684694.384	571897.5286	-0.2597	0.021305	0.3495	0.41985	Monobasic calcium phosphate	2.4044959	-	-	-	83.7991	233.045786
NEG05321	124141.5873	122760.0799	-0.01615	0.872707	0.9572	0.00418	Succite	-0.3296922	-	-	-	83.7925	117.080684
NEG05323	839860.3346	704952.9858	-0.25262	0.299665	0.7148	0.34728	2-Methacryloyloxyethyl phenyl phosphate	-12.262628	-	-	-	83.7709	285.206914
NEG05330	1789455.024	1484206.028	-0.26983	0.53866	0.8251	0.25026	Dihydrogen phosphate	2.5076671	-	-	-	83.0787	95.9801666
NEG05332	467358.5898	493375.0215	0.078155	0.548405	0.8287	0.11783	(R)-5-Phosphomevalote	-4.0258999	-	-	-	82.6095	227.128805
NEG05337	3284856.443	1375091.905	-1.2563	0.395298	0.7526	1.3907	Disodium malate	-13.163136	-	-	-	82.2852	177.04148
NEG05338	5406550.917	4170125.657	-0.37462	0.224558	0.6707	1.21877	Tetrafluoroethylene	-13.559129	-	-	-	82.2381	99.0063673
NEG05348	699301.2127	633324.2283	-0.14297	0.441504	0.7752	0.21222	3-hydroxy-2-(hydroxymethyl)-2-[(sulfooxy)methyl]propan[illegible]	-8.3868836	-	-	-	81.7067	229.180793
NEG05349	831226.7078	738018.5436	-0.17158	0.156301	0.6183	0.37447	Haloxydine	12.064445	-	-	-	81.7007	198.965436
NEG05356	731796.4132	576112.7083	-0.34509	0.149651	0.6145	0.35888	Arbutin 6-phosphate	-9.1053258	-	-	-	80.6771	351.220616
NEG05358	1412773.194	1973012.072	0.48187	0.217583	0.6665	0.61251	(Z)-4-(2-Hydroxy-5-sulfotophenyl)-2-oxo-3-butenoate	12.928646	-	-	-	80.6646	271.227543
NEG05366	1074438.574	1054807.317	-0.0266	0.929361	0.9763	0.12862	3,4-Dihydroxymandelate	0.0477654	-	-	-	79.9101	183.138832
NEG05369	356739.2016	355814.8065	-0.00374	0.976758	0.9933	0.03108	Bromobenzene	4.371434	-	-	-	79.617	156.00131
NEG05374	133182.3362	108105.0822	-0.30097	0.183902	0.6427	0.16672	Reduced coenzyme F420	9.9451539	-	-	-	78.41835	774.608937

NEG05375	408363.5683	433482.1312	0.086118	0.505399	0.8056	0.11031	Tricalcium phosphate	2.6262922	-	-	-	78.2228	309.170238
NEG05379	1762097.21	621008.9816	-1.50461	0.023266	0.3593	1.43183	sn-Glycero-3-phospho-1-inositol	-0.7404892	-	-	-	77.7544	333.206776
NEG05384	1656945.072	1417860.755	-0.22481	0.557179	0.8301	0.36917	Diethylphosphoric acid	-14.752985	-	-	-	77.5293	153.09195
NEG05386	8551232.383	10510847.94	0.297675	0.569971	0.8352	0.25441	Trimethylselenonium	0.4984485	-	-	-	77.1757	123.056385
NEG05387	4376049.205	3199222.588	-0.45191	0.145663	0.6127	0.99037	4-Maleylacetoacetate	-21.93197	-	-	-	77.1755	199.133834
NEG05388	1035833.187	1134117.158	0.130778	0.54118	0.8268	0.20095	3,4-Dihydroxyphthalate	-20.496705	-	-	-	77.1755	197.118262
NEG05397	1134792.694	1340082.318	0.239893	0.202684	0.6612	0.45796	Chrysoeriol	10.369296	-	-	-	76.4529	299.258737
NEG05398	127326.2348	118884.896	-0.09896	0.530894	0.8204	0.06982	5'-Deoxy-5-fluorocytidine	-3.73114	-	-	-	76.2998	244.199508
NEG05399	862858.935	876015.7546	0.021832	0.859787	0.9535	0.02933	Thiadiazolidinone	-13.129232	-	-	-	76.1519	325.20064
NEG05404	1432247.269	1197984.741	-0.25767	0.040424	0.438	0.59448	5-Azacytidine	-4.9455308	-	-	-	75.8179	243.196216
NEG05412	308771.3157	402674.1211	0.383074	0.247397	0.6846	0.25479	UDP-N-acetyl-D-galactosamine 4,6-bissulfate	16.936879	-	-	-	75.4472	766.485822
NEG05415	969252.358	827450.9264	-0.2282	0.530672	0.8203	0.34365	(1E,3E)-4-Hydroxybuta-1,3-diene-1,2,4-tricarboxylate	10.669948	-	-	-	75.3982	201.11318
NEG05422	153733.8033	242587.9429	0.658073	0.024733	0.3595	0.40808	N-Acetylglucosamine 4-sulfate	-5.5727634	-	-	-	75.10925	300.262044
NEG05428	23981132.25	12456435.44	-0.94501	0.296089	0.7138	3.27961	(2R,3S)-Piscidic acid	-19.183062	-	-	-	74.57315	255.196609
NEG05444	455501.296	347333.5645	-0.39113	0.435956	0.7716	0.27625	beta-Citryl-L-glutamate	1.9606774	-	-	-	73.88775	320.230853
NEG05452	1396435.27	153703.0363	-3.18353	0.014113	0.3053	1.48615	3',5'-Cyclic IMP	-23.860977	-	-	-	73.68555	329.175545
NEG05462	156935.2503	40384.94996	-1.95828	0.026714	0.367	0.45051	AminoDAHP	-4.4468456	-	-	-	73.3743	286.152546
NEG05466	4131583.91	1991891.035	-1.05256	0.157618	0.6192	1.70477	Inosine-5'-carboxylate	-18.896546	-	-	-	73.369	311.222423
NEG05468	1345993.196	1080931.446	-0.3164	0.082556	0.5402	0.63101	Fosetyl	-8.2089613	-	-	-	73.19335	109.04072
NEG05471	4791791.846	4480200.523	-0.097	0.197641	0.6557	0.66079	Angelicin	-8.9660489	-	-	-	73.0325	185.154554
NEG05478	267401.2382	376808.8563	0.494827	0.235748	0.6796	0.29464	Formyl-CoA	-24.999163	-	-	-	72.85465	794.517035
NEG05481	3019015.448	2789681.02	-0.11398	0.344468	0.7302	0.46909	Selenomethionine Se-oxide	-16.955213	-	-	-	72.7003	211.094827
NEG05493	305874.4352	506600.2593	0.727908	0.084418	0.5416	0.51446	Oxaloglutarate	8.8303974	-	-	-	72.0606	203.128726

NEG05495	88986.12304	62898.76773	-0.50055	0.156207	0.6183	0.14677	MC-5127	19.308994	-	-	-	71.9966	355.1571
NEG05503	2171183.394	2100320.961	-0.04787	0.786978	0.927	0.03529	Diallat	0.8612248	-	-	-	71.4788	269.212056
NEG05505	269064.8364	275272.154	0.032905	0.929644	0.9763	0.05235	2,3,4,7,8,9,15,22,23,28-decahydroxy-14-(hydroxymethyl)	-3.8649957	-	-	-	71.3077	753.507807
NEG05510	853038.766	563192.1849	-0.59898	0.053206	0.483	0.62002	2,4-Dinitroaniline	22.509176	-	-	-	70.96635	182.118445
NEG05512	4675907.65	4576357.394	-0.03105	0.638495	0.8672	0.34366	Diamidafos	13.22532	-	-	-	70.9637	199.170171
NEG05515	205865.1273	191039.5222	-0.10783	0.517957	0.8123	0.10914	Luteolin 7-O-[beta-D-glucuronosyl-(1->2)-beta-D-glucuronide]	-17.245534	-	-	-	70.6266	637.466212
NEG05517	2723064.05	690034.2238	-1.98049	0.32233	0.7192	1.20582	Wedelolactone	-2.9706891	-	-	-	70.2661	313.23819
NEG05520	282011.1598	225126.7412	-0.32501	0.355008	0.7334	0.19837	5-Tetrazolyl-glycine	-10.539308	-	-	-	70.25365	142.095315
NEG05522	1991382.568	1833248.665	-0.11937	0.177946	0.6369	0.43633	3-hydroxy-2-methyl-2-[(sulfooxy)methyl]propanoic acid	14.74565	-	-	-	69.92945	213.185882
NEG05530	4189238.998	3899280.99	-0.10348	0.292484	0.7095	0.54496	Pemirolast	-6.298332	-	-	-	69.4314	227.201386
NEG05536	1984754.725	2340452.406	0.237827	0.110519	0.5692	0.56812	Arbutin 6-phosphate	-8.9058242	-	-	-	69.2213	351.220686
NEG05542	82669.1838	105381.5639	0.350201	0.199111	0.6569	0.16848	Tannin	2.5268129	-	-	-	68.7263	619.420491
NEG05543	1320041.932	1346699.268	0.028844	0.841405	0.9478	0.06827	3-Phosphonooxypyruvate	-23.646341	-	-	-	68.71335	183.029771
NEG05545	10374054.21	11896367.78	0.197541	0.095136	0.5568	1.37187	N-Benzoyl-4-hydroxyanthranilate	7.5779606	-	-	-	68.5524	256.236073
NEG05547	491852.6628	413092.7172	-0.25176	0.321141	0.7192	0.23766	Delphinidin 3-O-3'',6''-O	-21.372653	-	-	-	68.5411	636.455699
NEG05551	870052.8491	826838.4994	-0.0735	0.764178	0.9204	0.07523	Corilagin	-13.959016	-	-	-	68.3648	633.436667
NEG05553	1254031.477	1042685.605	-0.26627	0.269295	0.6959	0.41021	1-O,2-O,6-O-Trigalloyl-beta-D-glucose	-14.076688	-	-	-	68.1925	635.452464
NEG05575	1020318.322	570548.6999	-0.8386	0.014991	0.3098	0.90393	1-Deoxy-D-altro-heptulose 7-phosphate	-21.220047	-	-	-	64.052	273.149306
NEG05588	655957.9584	1154942.248	0.816145	0.179907	0.6395	0.56909	2-Oxo-3-hydroxy-4-phosphobutanoate	-20.568218	-	-	-	62.9286	213.05562
NEG05599	236079.5343	189054.6415	-0.32047	0.617139	0.8569	0.21891	Disodium malate	-19.372854	-	-	-	61.9905	177.040374
NEG05610	107323.2366	97158.18208	-0.14356	0.334448	0.7265	0.10177	(R)-5-Diphosphomevalote	12.530011	-	-	-	61.44935	307.113484
NEG05635	421945.7407	447234.9366	0.083975	0.652657	0.874	0.06829	Tolcapone	-10.11358	-	-	-	60.2334	272.23076
NEG05651	733421.9058	744465.1516	0.021561	0.942256	0.9805	0.04686	Schradan	6.4316541	-	-	-	59.6065	285.243264

NEG05657	2629740.388	3107426.988	0.2408	0.152266	0.6145	0.65546	(Z)-4-(2-Hydroxy-5-sulfotophenyl)-2-oxo-3-butenoate	13.03817	-	-	-	59.512	271.227573
NEG05664	827978.8411	999232.9009	0.271227	0.302168	0.7148	0.36851	L-Histidinol phosphate	11.940966	-	-	-	59.2509	220.146264
NEG05665	440953.5138	493351.1684	0.161988	0.574242	0.8374	0.14603	Selfotel	6.3802403	-	-	-	59.2165	222.157647
NEG05677	6258924.277	7242966.259	0.210666	0.421954	0.7663	0.78073	Aminoimidazole ribotide	-1.3671935	-	-	-	58.9414	294.17872
NEG05681	36937913.68	40623156.34	0.1372	0.589707	0.8425	1.30726	Potassium dichromate	-5.8740068	-	-	-	58.8773	293.175595
NEG05682	4106078.885	4124890.863	0.006595	0.966453	0.9899	0.18015	Tetrafluoroethylene	-13.549971	-	-	-	58.7742	99.0063682
NEG05687	581168.0858	689180.1409	0.245926	0.369565	0.7408	0.27144	(R)-4'-phosphonatopantothenate(3-)	-14.401497	-	-	-	58.61765	295.181458
NEG05703	443072.0029	977137.5647	1.141021	0.067894	0.5177	0.87164	Sevoflurane	18.661069	-	-	-	58.3007	199.051257
NEG05709	18517464.29	19296815.43	0.059476	0.694034	0.894	0.37035	Glyoxylate	16.562075	-	-	-	58.1994	73.0294496
NEG05713	582190.6758	669903.3033	0.202461	0.272014	0.6974	0.19537	Bromobenzene	5.0178819	-	-	-	58.12525	156.001411
NEG05717	365513.1688	328693.4027	-0.15318	0.428119	0.7691	0.17886	Potassium bromate	20.282268	-	-	-	58.0373	165.996611
NEG05721	2934937.917	2628303.006	-0.1592	0.393909	0.7523	0.51483	Dihydrogen phosphate	2.4104652	-	-	-	57.9206	95.9801572
NEG05733	3295176.651	4404047.349	0.418474	0.38343	0.7477	0.8005	Diatretin 2	23.037861	-	-	-	57.6527	144.110967
NEG05736	278649.6762	1854.675118	-7.23114	0.019424	0.3424	0.68396	Ethiprole	-21.999952	-	-	-	57.60075	396.186985
NEG05744	1314105.215	42729.18584	-4.94272	0.012847	0.2932	1.49101	Furcozole-cis	-3.4077477	-	-	-	57.43025	395.183173
NEG05757	587785.5705	537756.9923	-0.12834	0.691361	0.8929	0.15991	5-Phosphoribosyl-4-carboxy-5-aminoimidazole	-12.465629	-	-	-	57.1576	335.162533
NEG05758	21813999.43	697326.6584	-4.96728	0.014697	0.3087	6.03373	5'-Phosphoribosyl-N-formylglycimide	4.7266256	-	-	-	57.0151	313.180708
NEG05762	1488196.352	1459628.44	-0.02796	0.884984	0.9609	0.17099	Barbiturate	-19.699228	-	-	-	56.9273	127.0764
NEG05764	754144.1961	312073.9044	-1.27295	0.026687	0.367	0.89062	2-(3,5-Dichlorophenylcarbamoyl)-1,2	-5.6160767	-	-	-	56.924	301.144226
NEG05776	721065.7641	6095903.834	3.079637	0.153169	0.6159	2.22479	Benzoyl meso-tartaric acid	-20.342115	-	-	-	56.59415	253.180453
NEG05778	187260.2135	175758.931	-0.09145	0.31522	0.7189	0.10578	Genistein 4',7-O-diglucuronide	-7.6793184	-	-	-	56.5907	621.473043
NEG05786	1164561.103	1042406.786	-0.15987	0.492637	0.8005	0.21614	5-Hydroxyisourate	1.8339778	-	-	-	56.5123	183.102761
NEG05797	141029.1285	126993.1588	-0.15124	0.261147	0.6904	0.12292	3'-demethyletoposide	-23.626125	-	-	-	56.3099	573.509149

NEG05801	268699.3311	441216.2991	0.715493	0.453539	0.7814	0.20808	[5-(Aminomethyl)furan-3-yl]methyl phosphate	10.883122	-	-	-	56.25735	206.116078
NEG05804	2456465.143	2602053.812	0.083067	0.676423	0.8854	0.30991	Chrysoeriol	10.27855	-	-	-	56.2363	299.25871
NEG05805	23023.64562	23631.71441	0.037608	0.918809	0.9735	0.00312	Cartormin	3.6762273	-	-	-	56.2265	574.512939
NEG05807	7750884.525	7415242.046	-0.06387	0.430015	0.7698	0.60824	2,5-Dioxopentanoate	5.1029798	-	-	-	56.2136	129.092087
NEG05810	209066.8034	159897.755	-0.38681	0.22396	0.6705	0.21201	Azacozole	-14.307884	-	-	-	56.188	299.129029
NEG05816	136301.5576	127852.9326	-0.09232	0.518381	0.8129	0.0589	3,4,5-trihydroxy-6-({15-hydroxy-11,17,18-trimethoxy-13-oxo-6,8,20 [illegible]	21.006681	-	-	-	56.15615	575.467833
NEG05817	60037.10469	66676.27103	0.151319	0.615527	0.8564	0.04176	N-[(7,8-Dihydropterin-6-yl)methyl]-4-(beta-D-ribofuranosyl)aniline	-19.110284	-	-	-	56.15325	481.367905
NEG05827	6769737.559	519500.6689	-3.7039	0.337985	0.7277	2.08946	Wedelolactone	-3.0770773	-	-	-	55.98115	313.238156
NEG05829	910719.3441	1063658.057	0.223956	0.542104	0.8268	0.10379	S-(1,2-Dichlorovinyl)-L-cysteine	17.955529	-	-	-	55.9439	215.082203
NEG05830	195915.048	152074.7109	-0.36545	0.365854	0.7399	0.2221	L-Ascorbate 6-phosphate	14.917696	-	-	-	55.9431	255.100544
NEG05831	166620.7326	155385.8919	-0.10071	0.445466	0.7776	0.04836	S-methyl-5-thio-D-ribulose 1-phosphate(2-)	11.337938	-	-	-	55.93815	257.175651
NEG05833	2102277.082	1809928.024	-0.21602	0.321299	0.7192	0.5013	4-Maleylacetoacetate	-21.886118	-	-	-	55.9187	199.133843
NEG05837	715653.22	740230.0714	0.048713	0.84887	0.9515	0.04425	3-Fluorocyclohexadiene-cis,cis-1,2-diol-1-carboxylate	-6.4026262	-	-	-	55.8908	173.118109
NEG05845	91436.44727	97746.31715	0.096273	0.722716	0.9063	0.05171	Cibarian	-22.158848	-	-	-	55.8369	381.261753
NEG05848	1024194.563	601634.48	-0.76753	0.073222	0.5226	0.82364	3,4,5-trihydroxy-6-[(2-hydroxyacetyl)oxy]oxane-2-carboxylic acid	-11.020602	-	-	-	55.7112	251.164944
NEG05849	792481.0965	898670.5791	0.181416	0.349654	0.7323	0.34164	O-Phosphorylhydroxylamine	12.900128	-	-	-	55.7107	112.003981
NEG05852	642883.0578	1068183.696	0.732532	0.421207	0.7661	0.35177	5-(methylsulfanyl)-2,3-dioxopentyl Phosphate(2-)	8.5059311	-	-	-	55.692	239.164766
NEG05856	657110.6471	641474.2789	-0.03474	0.840719	0.9478	0.02157	Inosine-5'-carboxylate	-19.212054	-	-	-	55.5931	311.222325
NEG05868	902197.94	418433.7349	-1.10844	0.065187	0.5103	0.78169	Ibandronate	-20.567088	-	-	-	55.3753	318.215058
NEG05870	856040.8127	803319.2755	-0.09171	0.746468	0.914	0.21338	Pydanon	16.450885	-	-	-	55.3638	187.133918
NEG05875	384148.0294	187752.6666	-1.03283	0.021016	0.3489	0.59608	2-(alpha-D-Mannosyl)-3-phosphoglycerate	-13.352451	-	-	-	55.3154	347.185874
NEG05878	205263.482	209675.437	0.030681	0.857397	0.9533	0.01418	3-hydroxy-2-(hydroxymethyl)-2-[(sulfooxy)methyl]propan [illegible]	-8.2880271	-	-	-	55.2774	229.180816
NEG05879	91172.97588	91633.36296	0.007267	0.979131	0.9946	0.00734	3,4,5-trihydroxy-6-({11-methoxy-13-oxo-6,8,20-trioxapentacyclo[10.8.0.0i [illegible]	-10.201384	-	-	-	55.2774	499.399619

NEG05880	1190800.137	1043370.633	-0.19068	0.203719	0.6615	0.4283	2,3-Diketo-5-methylthiopentyl-1-phosphate	4.8968268	-	-	-	55.27	241.180609
NEG05882	247535.8499	124417.6984	-0.99245	0.006212	0.2196	0.48611	Cycloguanil hydrochloride	-14.115774	-	-	-	55.2594	287.164956
NEG05884	511276.5514	449668.1722	-0.18524	0.42787	0.7691	0.21031	5-Azacytidine	-5.2926863	-	-	-	55.2562	243.196131
NEG05887	9708038.58	12528024.1	0.367907	0.715447	0.9033	1.29304	sn-Glycerol 3-phosphate	-0.0631609	-	-	-	55.2302	171.066413
NEG05888	1992106.473	2921031.508	0.552183	0.455108	0.7817	0.84707	Fenpiclonil	3.7762152	-	-	-	55.2208	236.078319
NEG05891	966249.5153	1164606.333	0.269375	0.343762	0.7301	0.25487	Diethylphosphoric acid	-15.065509	-	-	-	55.1857	153.091902
NEG05893	190138.4651	207355.5554	0.125056	0.760812	0.919	0.08423	2-{[hydroxy(6-hydroxy-7-methoxy-2H-1,3-benzodioxol-5	-8.6442519	-	-	-	55.17765	268.199396
NEG05894	1588822.321	1545184.204	-0.04018	0.882901	0.9601	0.03953	DHAP(6:0)	10.536186	-	-	-	55.1742	267.196349
NEG05895	317284.494	387600.2283	0.288792	0.125476	0.5916	0.24285	Urate-3-ribonucleoside	15.659691	-	-	-	55.1733	299.222325
NEG05897	228127.0388	296471.9349	0.378058	0.518763	0.8132	0.20707	Compound WIN VIII	23.462245	-	-	-	55.135	472.350729
NEG05909	1003369.269	782108.3611	-0.35941	0.498392	0.8016	0.34721	sn-Glycero-3-phospho-1-inositol	-0.9189013	-	-	-	54.9517	333.206716
NEG05911	489978.8377	410033.0878	-0.25698	0.199299	0.6569	0.29439	Stipitatate	2.6219422	-	-	-	54.9374	181.123401
NEG05915	788864.8768	716606.7648	-0.1386	0.579478	0.8394	0.23291	Phenolic phosphate	-11.618488	-	-	-	54.9288	173.081801
NEG05918	183121.3252	156476.7476	-0.22685	0.442195	0.7757	0.06837	Glucovanillin	-19.639097	-	-	-	54.9246	313.274451
NEG05922	22954.23994	30113.35428	0.391643	0.274168	0.6994	0.09107	(2S,3R)-2-[(1R)-1-[3,5-bis(trifluoromethyl)phenyl]ethoxy]-3-(4	9.7515849	-	-	-	54.9142	436.348188
NEG05925	726684.6435	657890.372	-0.14348	0.248008	0.6846	0.26119	Anthragallol	-24.914384	-	-	-	54.8789	255.19664
NEG05927	2784702.289	3250052.2	0.22294	0.69315	0.8938	0.40883	L-Selenocysteine	0.5392667	-	-	-	54.8721	167.046014
NEG05937	1268768.825	1101367.037	-0.20413	0.14866	0.6145	0.48985	L-Selenomethionine	18.392738	-	-	-	54.5981	195.10263
NEG05940	4939127.273	5408159.563	0.130882	0.355516	0.7339	0.6305	Bowdichione	11.404844	-	-	-	54.5256	297.243125
NEG05943	442409.1752	373572.4079	-0.24399	0.332684	0.7243	0.2106	norsertraline	0.8691088	-	-	-	54.514	291.195977
NEG05947	41038.63706	72572.30556	0.822436	0.055858	0.4884	0.22509	11-O-Demethylpradinone I	12.746886	-	-	-	54.4423	463.376443
NEG05950	201567.8188	218190.9251	0.114326	0.586238	0.8419	0.09027	Isochamaejasmin	1.2479812	-	-	-	54.3838	541.4831
NEG05957	208098.8379	148021.9184	-0.49146	0.408782	0.7599	0.24595	Juglone	1.7008404	-	-	-	54.2743	173.14582

NEG05961	349230.4367	366630.723	0.070148	0.875458	0.9578	0.04805	Reduced 2,6-Dichlorophenolindophenol	-4.5792607	-	-	-	54.2568	269.102886
NEG05963	1496464.014	1239313.338	-0.27202	0.026764	0.367	0.66119	3,4-Dihydroxymandelate	-0.0304505	-	-	-	54.22265	183.138818
NEG05964	42274.52112	55837.71649	0.401452	0.200037	0.658	0.1166	Phosphoramidon	-19.095806	-	-	-	54.21525	542.486245
NEG05965	2819212.529	2393895.408	-0.23593	0.105509	0.5677	0.75398	Tris(1-aziridinyl)phosphine oxide	-18.161083	-	-	-	54.21205	172.142279
NEG05967	176837.9103	141085.6076	-0.32586	0.400195	0.7562	0.15625	Nicergoline	10.660399	-	-	-	54.18985	483.383887
NEG05971	1149383.007	1208367.278	0.072199	0.671878	0.883	0.10772	Chloroneb	-4.0886203	-	-	-	54.15375	206.045777
NEG05973	341099.3264	248567.1117	-0.45656	0.015036	0.3098	0.3869	Niflumic acid	4.7893152	-	-	-	54.1201	281.212075
NEG05977	3980256.918	4495450.842	0.175604	0.339316	0.7277	0.50278	Diallat	0.6184116	-	-	-	54.0516	269.211991
NEG05984	1141025.649	1043992.021	-0.12822	0.6336	0.8648	0.11845	Tricrozarin A	18.828316	-	-	-	53.8224	293.211963
NEG05988	1159682.481	1044071.884	-0.15151	0.221005	0.6692	0.3179	Meconic acid	11.165608	-	-	-	53.7113	199.097458
NEG05989	2349797.205	2578338.868	0.133906	0.558044	0.8305	0.3545	Glycerone phosphate	0.4884455	-	-	-	53.6861	169.050606
NEG05992	175242.6613	188918.8931	0.108413	0.570063	0.8352	0.07901	Hexaflurate	6.443226	-	-	-	53.57785	227.004493
NEG05993	473006.2267	401890.662	-0.23506	0.66779	0.8807	0.19609	2,4-DB	-12.54089	-	-	-	53.5774	248.0802
NEG05994	82120.29663	79654.49115	-0.04398	0.849979	0.9515	0.01228	7-Dehydrologanin tetraacetate	-13.133444	-	-	-	53.57475	555.498614
NEG05999	3594446.503	3186006.875	-0.17402	0.383651	0.7477	0.61501	(1S,3R)-3-(2,2-Dichloroethenyl)-2,2-dimethylcyclopropanecarb	-5.3898544	-	-	-	53.5078	208.061397
NEG06009	1447744.023	124045.0011	-3.54487	0.018205	0.3332	1.47046	3',5'-Cyclic IMP	-23.970384	-	-	-	53.28005	329.175509
NEG06016	2850161.766	2541173.1	-0.16555	0.476921	0.7932	0.16647	Phaseolic acid	16.220883	-	-	-	53.2108	295.227129
NEG06019	1486386.76	1956656.904	0.396581	0.245283	0.6839	0.62141	Tricalcium phosphate	2.8514621	-	-	-	53.17375	309.170308
NEG06022	281091.6486	675042.8542	1.263939	0.321984	0.7192	0.63079	Nitrofen	-19.878714	-	-	-	53.0811	283.081976
NEG06023	94425.93	88861.34105	-0.08763	0.856964	0.9533	0.00607	Mangiferin	-6.3386328	-	-	-	53.04385	421.329646
NEG06031	9458616.874	7619404.963	-0.31195	0.001154	0.0993	1.82724	Angelicin	-9.0999696	-	-	-	52.81095	185.154529
NEG06032	124064.3583	80035.58941	-0.63238	0.036838	0.4235	0.26383	3-(Uracil-1-yl)-L-alanine	5.2165146	-	-	-	52.7707	198.157862
NEG06036	355754.7694	228925.8148	-0.636	0.007767	0.2439	0.44671	TEPP	-0.1241581	-	-	-	52.71645	289.180387

NEG06040	132146.2491	131314.6422	-0.00911	0.974269	0.9925	0.00586	Pteroyltriglutamic acid	16.281502	-	-	-	52.45115	698.629514
NEG06043	136231.0646	92698.9673	-0.55543	0.050713	0.4758	0.22445	Melarsoprol	0.7055479	-	-	-	52.4022	397.331704
NEG06044	413151.7105	741473.4595	0.843723	0.359912	0.7364	0.56141	2-<br>Protocatechoylphlorogluci<br>nolcarboxylate	-18.01687	-	-	-	52.3984	305.211606
NEG06050	124612.7414	104979.1114	-0.24735	0.458154	0.7822	0.12505	(-)-Epiafzelechin 3-gallate	-5.5790354	-	-	-	52.1739	425.363245
NEG06051	207912.8799	165660.0168	-0.32775	0.207587	0.6619	0.19985	Diclosulam	23.945835	-	-	-	52.1527	405.222051
NEG06053	3048578.994	6792561.744	1.155819	0.162937	0.6247	2.0828	2-Phosphoglycolate	-19.656968	-	-	-	52.13435	155.020956
NEG06059	166706.143	134794.2945	-0.30655	0.047466	0.4627	0.19356	Thiadiazolidinethione	-3.6296324	-	-	-	51.9166	341.269281
NEG06060	590053.5885	531211.6435	-0.15156	0.383155	0.7477	0.14052	Tetracenomycin M	7.2389391	-	-	-	51.91475	355.321403
NEG06064	3838146.142	93614.39791	-5.35754	0.333684	0.7254	1.43199	3-Deoxy-D-manno-<br>octulosote	4.0660397	-	-	-	51.8228	237.185692
NEG06067	247986.9527	195505.0891	-0.34306	0.016269	0.3186	0.29578	Nifuradene	16.644909	-	-	-	51.6319	223.169955
NEG06069	18494847.18	16789106.3	-0.1396	0.653815	0.8741	0.27532	Diamidafos	13.333673	-	-	-	51.57955	199.170192
NEG06071	1724088.818	1843659.847	0.096738	0.756052	0.9174	0.08898	3-{[(2E)-4-Amino-4-<br>oxobut-2-enoyl]amino}-L-<br>alanine	4.2850969	-	-	-	51.571	200.173485
NEG06074	207602.5296	194256.5697	-0.09586	0.686748	0.89	0.04925	Cidofovir	13.686886	-	-	-	51.4853	278.183545
NEG06076	29494.62028	34189.33078	0.213094	0.388818	0.7506	0.05482	1,3,5,8-Tetrahydroxy-6-<br>methoxy-2-<br>methylanthraquinone 8-O	-7.1579662	-	-	-	51.4668	477.392199
NEG06077	244031.2684	144542.9324	-0.75557	0.074535	0.5247	0.33636	CGH 2466	0.7197233	-	-	-	51.4622	321.205355
NEG06078	232185.9728	190256.6557	-0.28733	0.146764	0.6136	0.20069	(R)-4'-<br>phosphonatopantothenate(<br>3-)	11.170682	-	-	-	51.4605	295.189032
NEG06080	531434.8836	874688.9697	0.718877	0.42842	0.7691	0.53571	Vicianose	-14.852227	-	-	-	51.311	311.258585
NEG06081	515144.485	541469.3045	0.071902	0.683906	0.8892	0.09224	Triphenyl phosphate	-4.0119811	-	-	-	51.28825	325.274514
NEG06082	282329.6581	254169.0066	-0.15159	0.547982	0.8287	0.1484	Styraxin	-23.142383	-	-	-	51.25605	369.336853
NEG06084	326635.985	451192.3382	0.466059	0.007309	0.24	0.47913	beta-Citryl-L-glutamate	0.4375818	-	-	-	51.23865	320.230364
NEG06087	339101.7439	305835.412	-0.14896	0.314681	0.7189	0.1581	Chlorogete	11.702964	-	-	-	51.1869	353.30557
NEG06090	426319.9879	608284.6987	0.51281	0.586969	0.8421	0.19833	{[4-(7-methoxy-2-oxo-2H-<br>chromen-6-yl)-2-oxobut-3-<br>en-1-yl]oxy}sulfonic acid	-8.6048251	-	-	-	51.0871	339.289795
NEG06095	95094.05618	75527.93233	-0.33234	0.040261	0.438	0.15985	Methyl 2,3,4,6-tetra-O-<br>galloyl-beta-D-<br>glucopyranoside	-24.570079	-	-	-	50.9165	801.572503

NEG06096	597450.5918	553568.4975	-0.11006	0.484639	0.7985	0.12838	4-(Methylnitrosamino)-1-(3-pyridyl)-1-butanol glucuronide	-15.630674	-	-	-	50.91255	384.3558
NEG06101	234401.3977	173259.8822	-0.43604	0.05851	0.4925	0.27653	Decarbamoylgonyautoxin 1	12.66865	-	-	-	50.8748	367.32109
NEG06102	3091547.521	2327421.182	-0.4096	0.087138	0.5472	0.9612	[2-methoxy-4-(3,5,7-trihydroxy-3,4-dihydro-2H-1-benzopyran-2	-0.4589116	-	-	-	50.80705	383.352547
NEG06104	674683.363	497722.6368	-0.43887	0.05626	0.4892	0.45354	Pyridoxamine phosphate	17.795963	-	-	-	50.7785	247.17014
NEG06107	263482.0012	271805.6332	0.044871	0.813762	0.9353	0.02552	1,2,3,6-Tetrakis-O-galloyl-beta-D-glucose	-11.560392	-	-	-	50.7346	787.556507
NEG06108	49288.51064	76549.20949	0.635136	0.194411	0.6521	0.15082	FADH2	-17.634127	-	-	-	50.71235	786.544435
NEG06109	5311670.658	4314993.658	-0.29981	0.01412	0.3053	1.23364	3-hydroxy-2-methyl-2-[(sulfooxy)methyl]propanoic acid	14.508503	-	-	-	50.6756	213.185831
NEG06110	231951.9763	253321.8527	0.127145	0.680442	0.8871	0.03858	6''-(4-Carboxy-3-hydroxy-3-methylbutanoyl)hyperin	-16.311663	-	-	-	50.6756	607.484398
NEG06111	160924.4067	237673.5357	0.562598	0.20829	0.6619	0.25718	Tellimagrandin I	-10.977348	-	-	-	50.6756	785.541089
NEG06116	3166431.075	2899110.172	-0.12725	0.280511	0.7005	0.55347	6-{[6-(3,4-dihydroxy-6-methyl-5-oxooxan-2-yl)-2-(3,4-dihydroxyphenyl)-5	-5.931803	-	-	-	50.6254	605.478126
NEG06118	472775.7455	468665.1544	-0.0126	0.938201	0.9792	0.0009	hesperetin 3'-O-sulfate	5.2850797	-	-	-	50.5885	381.336744
NEG06119	936344.5595	811076.2577	-0.2072	0.173789	0.6333	0.40195	alpha-1,5-L-Arabinotetraose	-18.019558	-	-	-	50.5864	545.456676
NEG06123	64732.83828	44820.84083	-0.53033	0.324707	0.7202	0.16665	(-)-Epigallocatechin 3,3'-di-gallate	-21.78867	-	-	-	50.5788	609.455422
NEG06124	211073.8342	185364.8668	-0.18738	0.263545	0.6922	0.17584	Epicatechin 3-O-(3-O-methylgallate)	-6.0118776	-	-	-	50.5776	455.38888
NEG06125	116951.7955	97167.04977	-0.26737	0.335606	0.7266	0.14372	Manniflavanone	-24.444479	-	-	-	50.57345	589.466189
NEG06126	63539.11274	53815.638	-0.23962	0.433835	0.77	0.10069	Myricatin	-4.9101638	-	-	-	50.55845	551.408011
NEG06130	296680.7726	230656.4316	-0.36317	0.022741	0.3565	0.33498	Phosphotyrosine	13.728005	-	-	-	50.45305	260.164709
NEG06131	1278692.073	1128851.732	-0.17981	0.174292	0.6335	0.43837	7''-O-Phosphohygromycin	-18.264703	-	-	-	50.45285	606.481628
NEG06134	737367.2968	659559.8314	-0.16088	0.321396	0.7192	0.30195	6-{[6-(3,4-dihydroxy-6-methyl-5-oxooxan-2-yl)-5-hydroxy-2-(4-hydroxy-3	-23.841995	-	-	-	50.36565	619.493929
NEG06141	62384.97713	73261.85934	0.231864	0.786802	0.927	0.05073	Amaranth	-5.510927	-	-	-	50.2587	603.462392
NEG06146	608443.8379	598068.4067	-0.02481	0.947267	0.9827	0.07903	4-Glutathionyl cyclophosphamide	10.208019	-	-	-	50.2465	565.392105
NEG06147	381433.7871	274671.723	-0.47372	0.155051	0.6173	0.2908	4-Amino-anhydrotetracycline	23.08514	-	-	-	50.2438	397.36802
NEG06149	2585092.623	2303493.626	-0.16639	0.622401	0.8589	0.46569	5-Acetylamino-6-formylamino-3-methyluracil	16.022643	-	-	-	50.23535	225.185748

NEG06155	217472.099	151445.3771	-0.52203	0.0614	0.501	0.28373	2,4,6-Trihydroxybenzophenone	-3.3250191	-	-	-	50.1834	229.208058
NEG06159	13893.34631	20266.72552	0.544719	0.197964	0.656	0.07865	2-[(2-{[5,7-dihydroxy-2-(4-hydroxyphenyl)-4-oxo-4H-chromen-3-yl]oxy}	2.4578922	-	-	-	50.14575	815.712731
NEG06161	3590822.276	3652760.23	0.024673	0.922882	0.9741	0.03842	Endalin	15.462471	-	-	-	50.0906	228.204568
NEG06165	2139356.783	2068880.673	-0.04833	0.775684	0.9235	0.12513	Podophyllotoxone	4.088055	-	-	-	49.9926	411.383809
NEG06166	13516.14727	27475.97509	1.023487	0.133534	0.5988	0.12916	Uroporphyrin III	-13.895637	-	-	-	49.9926	829.72808
NEG06167	4877525.396	5343512.709	0.131639	0.622979	0.859	0.22654	Phosphoenolpyruvate	0.1852154	-	-	-	49.9398	167.034755
NEG06168	178333.3174	294129.5075	0.721875	0.391192	0.7513	0.29621	Digallate	8.5123645	-	-	-	49.93775	321.219266
NEG06169	597130.8468	650301.7497	0.123062	0.515857	0.8114	0.19179	6-Deoxy-6-sulfo-D-fructose 1-phosphate	-10.697555	-	-	-	49.9357	323.188855
NEG06178	114522.8064	141791.2664	0.308134	0.473537	0.792	0.12138	Cyanidin 3-O-3'',6''-O-	-22.678963	-	-	-	49.872	620.455829
NEG06180	34453.37102	28237.6979	-0.28702	0.506541	0.8064	0.05699	Pyrazosulfuron-ethyl	9.1019205	-	-	-	49.8363	413.390195
NEG06185	1052990.952	1286663.067	0.289141	0.483728	0.798	0.25942	Chloropropylate	4.9483122	-	-	-	49.6143	338.207502
NEG06187	633626.6767	608114.8409	-0.05929	0.858707	0.9535	0.03126	6-Amino-6-deoxyfutalosine	24.818204	-	-	-	49.6061	412.387083
NEG06188	82489.66179	85639.54016	0.054064	0.883647	0.9605	0.01368	6-(2-Carboxyethyl)-7-hydroxy-2,2-dimethyl-4-chromanone glucoside	-17.390851	-	-	-	49.5922	425.399708
NEG06189	2983165.436	2643727.16	-0.17427	0.500798	0.8022	0.41018	Diflunisal	-18.590786	-	-	-	49.59145	249.185672
NEG06190	4237248.411	2488526.624	-0.76784	0.100248	0.5617	1.29586	(1R,6R)-6-Hydroxy-2-succinylcyclohexa-2,4-diene-1-carboxylate	-2.5854457	-	-	-	49.5783	239.201502
NEG06199	2459631.935	2689209.428	0.12874	0.700468	0.8986	0.25835	Atoxin a(s)	2.3869582	-	-	-	49.27615	251.201325
NEG06200	204141.4442	161860.3159	-0.33482	0.274151	0.6994	0.16696	Pseudohypericin	6.8055828	-	-	-	49.2661	519.438865
NEG06204	1309359.463	2841608.645	1.117847	0.457913	0.7822	0.95428	1-(5'-Phosphoribosyl)-5-amino-4-imidazolecarboxamide	3.8303021	-	-	-	49.22745	337.205219
NEG06207	10278157.08	12513554.23	0.28391	0.597007	0.8463	1.4657	Cobaltous sulfate	6.3304606	-	-	-	49.1981	153.989505
NEG06214	1443549.691	1464203.394	0.020495	0.970722	0.9916	0.08418	Porphobilinogen	2.4366368	-	-	-	49.0265	225.222475
NEG06215	162203.5798	832801.5309	2.360167	0.174586	0.6336	0.93389	1-(5-Phospho-D-ribosyl)-5-amino-4-imidazolecarboxylate	17.5593	-	-	-	49.01385	338.194579
NEG06216	111057.24	115936.8846	0.062036	0.80161	0.9311	0.00285	Propicozole	-0.0804797	-	-	-	48.9727	341.213096
NEG06218	312094.9566	192427.5241	-0.69767	0.049312	0.4711	0.36797	Mandelonitrile rutinoside	-8.0177786	-	-	-	48.9505	440.418284

NEG06219	66305.12392	83259.15657	0.328489	0.381287	0.7477	0.11457	Clitocine	-24.409458	-	-	-	48.9326	286.215112
NEG06220	1030799.698	987586.2767	-0.06179	0.844282	0.9487	0.03163	(2-{9-hydroxy-2-oxo- 2H,8H,9H-furo[2,3- h]chromen-8-yl}-2-[(3	5.5879046	-	-	-	48.9008	439.415184
NEG06226	472185.6826	499312.6258	0.080589	0.810434	0.9347	0.08404	Neopterin	-10.76754	-	-	-	48.7921	252.204697
NEG06228	61950.39456	57784.42776	-0.10043	0.74623	0.9139	0.01638	cyclo-Dopa- glucuronylglucoside	24.078598	-	-	-	48.7623	532.442368
NEG06229	40907.24597	43294.32905	0.081822	0.726513	0.9068	0.01558	Methotrexate	-2.36935	-	-	-	48.75445	453.430947
NEG06230	7257.844309	31751.71138	2.129221	0.026134	0.3665	0.20712	3,4,5-trihydroxy-6-({3- hydroxy-8,8-dimethyl-4- oxo-2-phenyl-4H,8H	24.969705	-	-	-	48.7491	511.47252
NEG06231	281674.6679	292759.2951	0.055685	0.758928	0.918	0.06122	Biochanin A 7-O-(6-O- malonyl-beta-D-glucoside)	-7.8450447	-	-	-	48.7491	531.438846
NEG06232	46262256.09	38644325.95	-0.25958	0.50946	0.8082	2.10341	Cyperaquinone	-9.7409507	-	-	-	48.7339	241.217164
NEG06234	258449.4763	361311.1603	0.48336	0.072221	0.5226	0.37306	1-Methyl 2- galloylgalactarate	-15.389978	-	-	-	48.65375	375.256533
NEG06237	210206.4043	485563.7439	1.207854	0.021214	0.3495	0.67282	11-O- Demethylpradimicinone I	17.553038	-	-	-	48.6275	534.457822
NEG06239	678117.8831	1458935.249	1.105308	0.004745	0.1927	1.23333	5''- Phosphoribostamycin	16.98883	-	-	-	48.5758	533.454403
NEG06240	491177.3318	414476.5017	-0.24495	0.245941	0.6842	0.27721	2,4-Dichloro-cis,cis- mucote	-11.068617	-	-	-	48.5714	209.989988
NEG06242	252931.1046	543860.2384	1.104491	0.00016	0.0947	0.76004	Tiron	-6.0753354	-	-	-	48.5557	313.191515
NEG06245	379790.2089	220416.3019	-0.78497	0.01635	0.3189	0.51382	5- Hydroxymethyldeoxycytid ylate	22.689275	-	-	-	48.5284	336.223475
NEG06248	157735.695	189245.8889	0.262753	0.347038	0.7313	0.17939	Vitisin A	22.83016	-	-	-	48.4496	560.473842
NEG06249	421165.9685	570323.1737	0.437391	0.205744	0.6619	0.41658	1-(4-Hydroxy-2- methylpyrimid-5- ylmethyl)-3-(beta	20.297027	-	-	-	48.4277	339.215229
NEG06250	496858.9792	816626.5889	0.71684	0.019446	0.3424	0.7321	[2,6-dihydroxy-3-(7- hydroxy-4-oxo-4H- chromen-2	-4.2805312	-	-	-	48.4172	365.291155
NEG06253	3685355.249	1829700.18	-1.0102	0.018507	0.3344	1.85253	Fosetyl	-8.8410739	-	-	-	48.3702	109.04065
NEG06259	539569.6626	440930.2875	-0.29126	0.257048	0.6894	0.30256	104-1	2.0579562	-	-	-	48.2506	467.446387
NEG06260	130538.0563	145664.3126	0.158177	0.653683	0.8741	0.0836	({3,4,5-trihydroxy-6- [3,5,7-trihydroxy-2-(2,4,5- trihydroxyphenyl)-3,4	12.394622	-	-	-	48.2506	547.469521
NEG06262	2489261.482	1575376.787	-0.66002	0.059976	0.4982	1.14134	Clitidine 5'- phosphate	-19.506652	-	-	-	48.2416	349.204492
NEG06263	2076767.192	2643168.088	0.347929	0.350982	0.7323	0.73217	2,5-Diamino-6-(5- phospho-D- ribosylamino)pyrimidin	14.970149	-	-	-	48.2416	352.223811
NEG06264	253411.7464	425751.4663	0.748528	0.238621	0.6811	0.45511	({3,4,5-trihydroxy-6-[5- hydroxy-7-methoxy-4- oxo-2-(2,4,5	2.9505504	-	-	-	48.2416	557.454371

NEG06265	308364.6762	421855.6806	0.452112	0.029319	0.3836	0.43198	5'-Butyrylphosphouridine	8.7388496	-	-	-	48.23975	393.267269
NEG06273	373480.269	358624.668	-0.05856	0.831943	0.9445	0.05027	3,4,5-trihydroxy-6-{[3-hydroxy-2-(hydroxymethyl)-2-	-20.973205	-	-	-	48.16835	309.241216
NEG06275	62565109.73	48830516.05	-0.35758	0.024893	0.3596	4.29581	Nifurthiazole	7.2535822	-	-	-	48.1008	253.217167
NEG06278	409513.2726	939346.5767	1.197747	0.028055	0.3758	0.91959	3,4,5-trihydroxy-6-{[5-hydroxy-4-oxo-2-phenyl-8-(3,4,5-trihydroxyoxan-2-	23.100265	-	-	-	47.99	561.485717
NEG06280	4143341.378	3232677.552	-0.35807	0.150727	0.6145	1.15901	Cacodylate	8.0152163	-	-	-	47.95475	136.991229
NEG06282	1137230.713	860478.9179	-0.40231	0.027882	0.3745	0.59057	Benzoylprop-ethyl	14.31275	-	-	-	47.93955	365.236465
NEG06293	1388838.28	1222675.883	-0.18384	0.603307	0.8497	0.22644	4-[2-(5-Carboxy-2-hydroxy-3-methoxyphenyl)-2-	23.733971	-	-	-	47.122	351.250884
NEG06296	154363.135	103818.2594	-0.57227	0.192805	0.6509	0.21571	Nifursol	14.519352	-	-	-	47.02495	364.210126
NEG06301	14795092.54	14292168.94	-0.04989	0.687133	0.89	0.16907	7-Methylinosine	-5.9623314	-	-	-	46.8492	282.251635
NEG06302	35005.04879	31125.74416	-0.16945	0.543443	0.8268	0.0475	Proanthocyanidin A2	7.4403826	-	-	-	46.8135	575.501413
NEG06303	138793.9231	401665.476	1.53305	0.016864	0.3231	0.62976	Prostalidin A	-2.0236553	-	-	-	46.7814	393.323025
NEG06308	86391.70833	221366.9362	1.357475	0.045493	0.4515	0.47029	Maysin 3'-methyl ether	-8.6627047	-	-	-	46.5396	589.517008
NEG06312	93045.51014	161232.7059	0.793136	0.141786	0.6087	0.27222	Prunin 6''-O-gallate	-7.6600019	-	-	-	46.36535	585.485831
NEG06317	1143629.755	2163964.231	0.920057	0.072534	0.5226	1.07626	N7-Methylguanosine	9.2087118	-	-	-	46.23395	297.27067
NEG06318	3941869.705	6278609.35	0.671565	0.053607	0.4853	1.73914	2'-Carboxy-4-[bis(2-chloroethyl)amino]-2-methylazobenzene	-24.863283	-	-	-	46.2149	379.251669
NEG06324	46865.64428	111898.7343	1.255591	0.005758	0.2144	0.34514	N7-Methylguanosine 5'-diphosphate	2.7439989	-	-	-	45.9422	457.228981
NEG06333	2054327.15	1487066.339	-0.4662	0.387257	0.7495	0.43553	3,4,5-trihydroxy-6-(2,3,4,5-tetrahydroxybenzoyloxy)o	-0.5532508	-	-	-	45.6256	361.235523
NEG06344	6054527.772	4717382.888	-0.36003	0.217611	0.6665	1.08964	3,4,5-trihydroxy-6-(1-oxo-1H-isochromene-3-carbonyloxy)oxane-2	-10.882464	-	-	-	44.59825	365.266737
NEG06346	37405.50026	124459.2221	1.734351	0.001175	0.0993	0.41378	Ethalfluralin	3.0401138	-	-	-	44.4764	332.256937
NEG06349	2692893.484	1384122.146	-0.96019	0.013633	0.3012	1.54239	2,2-Bis(4-hydroxyphenyl)hexafluoropropane	-5.2619206	-	-	-	44.24655	335.220054
NEG06354	17861662.67	18123891.75	0.021026	0.912676	0.971	0.20679	2-hydroxy-3-[4-hydroxy-3-(sulfooxy)phenyl]propanoic acid	-20.654243	-	-	-	43.87675	277.216977
NEG06356	8700100.695	7189440.835	-0.27515	0.143554	0.6122	1.11776	2-Amino-4-hydroxy-6-(D-erythro-1,2,3-trihydroxypropyl)-7,8	-11.137566	-	-	-	43.87675	254.220481
NEG06357	57133240.49	65888867.28	0.205704	0.461592	0.7839	1.70556	N-Benzoyl-4-hydroxyanthranilate	7.6656705	-	-	-	43.87675	256.236095

NEG06360	181029.584	158092.9117	-0.19545	0.517182	0.8117	0.10545	3,4,5-trihydroxy-6-({3-[3-methoxy-4-(sulfooxy)phenyl]prop-2	-4.0445683	-	-	-	43.8132	449.360902
NEG06363	551209.0359	573080.2839	0.056138	0.819942	0.938	0.00711	Bromobenzene	4.5993548	-	-	-	43.7002	156.001346
NEG06370	49489845.7	52215017.8	0.077332	0.865063	0.9549	1.37042	Endothion	18.559723	-	-	-	43.4319	279.232624
NEG06372	110621844.8	191365108.1	0.790691	0.007651	0.2427	12.1141	Coformycin	9.2888819	-	-	-	43.40905	283.263864
NEG06373	2032693.227	1603659.219	-0.34202	0.098115	0.5586	0.63495	Psicofuranine	24.064889	-	-	-	43.40905	296.267177
NEG06374	21041892.86	36858955.2	0.808751	0.009666	0.2615	5.32282	3,5,7-trihydroxy-2-(3-methoxyphenyl)-5H-chromen-5-yl	-1.7292804	-	-	-	43.40905	284.26723
NEG06375	1850143.155	3326628.343	0.846424	0.009718	0.2615	1.62577	Salicin	-0.1227049	-	-	-	43.40905	285.270488
NEG06385	4246928.118	4237619.552	-0.00317	0.990889	0.9983	0.04516	Picein	-6.3952318	-	-	-	42.9325	297.279316
NEG06389	5637931.164	5697081.176	0.015057	0.957185	0.9865	0.02141	[7-hydroxy-3-(2-hydroxy-4-methoxyphenyl)-3,4-dihydro-2H-1-benzopyran	13.280862	-	-	-	42.79975	367.357616
NEG06390	494166.5229	65155.49998	-2.92304	0.020201	0.3444	0.88163	Ethiprole	-8.766487	-	-	-	42.7839	396.192241
NEG06391	1753830.004	1699701.569	-0.04523	0.838339	0.9471	0.05843	{[1-(7-hydroxy-2-oxo-2H-chromen-8-yl)-3-methylbut-3-en-2	-6.4398935	-	-	-	42.77315	325.310622
NEG06393	3811737.113	3941692.475	0.048367	0.719204	0.9041	0.31364	Pyridafenthion	-2.7851167	-	-	-	42.6142	339.326276
NEG06395	1082986.371	925222.3159	-0.22714	0.261617	0.6909	0.28421	Saxitoxin	10.678965	-	-	-	42.5274	298.282419
NEG06397	1137276.325	1193975.725	0.070191	0.653858	0.8741	0.22417	Melicopine	3.7001017	-	-	-	42.45925	312.298583
NEG06405	4835974.207	3170369.173	-0.60916	0.115023	0.5747	1.36483	5-FU	-23.016699	-	-	-	41.71655	129.066929
NEG06409	1347613.947	962204.2016	-0.48599	0.604999	0.85	0.43927	Adenylylselete	-19.321995	-	-	-	41.64625	473.162961
NEG06413	6426354.204	4737376.006	-0.43991	0.412102	0.7613	0.92619	2-Maleylacetate	-21.592437	-	-	-	41.4791	157.098109
NEG06414	476506.8776	384567.476	-0.30926	0.129952	0.5933	0.38489	Haloxydine	12.140166	-	-	-	41.47865	198.965451
NEG06415	664326.5269	561706.7075	-0.24208	0.703916	0.8994	0.03429	3-(Dichloromethylene)-2,5-pyrrolidinedione	-8.0587493	-	-	-	41.4702	178.980273
NEG06429	361537.3098	343798.7963	-0.07258	0.585992	0.8419	0.13564	3-(Dichloromethylene)-2,5-pyrrolidinedione	11.281115	-	-	-	41.2175	178.983754
NEG06443	11859484.75	11668433	-0.02343	0.777785	0.9242	0.40816	Formylphosphote	-22.014344	-	-	-	41.0581	108.996202
NEG06464	932308.1601	874481.856	-0.09238	0.866798	0.9556	0.15175	L-3,4-Dihydroxybutan-2-one 4-phosphate	0.7918253	-	-	-	40.8125	183.077269
NEG06469	1350697.933	1298701.793	-0.05663	0.39502	0.7526	0.21012	2,5-Furandicarboxylate	-20.388582	-	-	-	40.7736	155.082541

NEG06470	833208.5417	858601.4227	0.043311	0.927161	0.9759	0.09467	3-Oxalomalate	-8.3795705	-	-	-	40.76875	205.097996
NEG06471	3962159.096	3557297.214	-0.15551	0.800731	0.9311	0.52902	Trimethylselenonium	0.3231813	-	-	-	40.7636	123.056363
NEG06481	59600.52498	89216.86115	0.581991	0.012603	0.2923	0.19766	Phosacetim	8.08511	-	-	-	40.59955	374.205757
NEG06483	307296.1154	207421.2687	-0.56707	0.20783	0.6619	0.32772	Dihydroxyfumarate	16.951894	-	-	-	40.5718	147.066233
NEG06484	15312003.66	15466353.51	0.01447	0.914638	0.9719	0.09924	Glyoxylate	16.545204	-	-	-	40.5364	73.0294483
NEG06494	2223373.382	2190431.965	-0.02153	0.872974	0.9572	0.11821	Dihydrogen phosphate	2.3152467	-	-	-	40.4453	95.9801479
NEG06506	184674.588	89282.22636	-1.04854	0.103599	0.5647	0.33554	Radon-222	14.589399	-	-	-	40.3461	220.995962
NEG06509	290584.0255	247909.9382	-0.22914	0.653904	0.8741	0.08568	Ferrous lactate	-10.318088	-	-	-	40.32935	232.975309
NEG06525	1117015.824	2069564.662	0.889678	0.067818	0.5176	0.97447	N-Acetyldemethylphosphinothricin	21.028054	-	-	-	40.1642	208.134021
NEG06536	1643172.513	140253.1534	-3.55038	0.028575	0.378	1.54175	Furcozole-cis	8.1925746	-	-	-	40.05885	395.187769
NEG06547	323374.4286	332305.2943	0.039304	0.75769	0.918	0.02742	Coumermic acid	-3.4459546	-	-	-	40.00775	546.460537
NEG06550	98816.04813	133339.869	0.432291	0.499606	0.8016	0.10111	19-Bromoaplysiatoxin	24.676262	-	-	-	39.98465	749.520843
NEG06576	545388.1176	546207.5736	0.002166	0.995458	0.9987	0.05053	3-Fluorocyclohexadiene-cis,cis-1,2-diol-1-carboxylate	-6.0159017	-	-	-	39.7368	173.118176
NEG06580	925478.4587	914915.4307	-0.01656	0.88424	0.9606	0.06899	alpha-1,5-L-Arabinotetraose	-16.219487	-	-	-	39.7257	545.45766
NEG06582	1063468.634	1125493.548	0.08178	0.532357	0.8211	0.17989	Glyphosate	4.0020795	-	-	-	39.687	168.0665
NEG06585	52772.46543	105650.2406	1.001439	0.224095	0.6707	0.20799	UDP-N-acetyl-D-galactosamine 4,6-bissulfate	15.378965	-	-	-	39.6738	766.484626
NEG06587	265071.7381	210237.3232	-0.33436	0.638416	0.8672	0.17904	Selenocysteine seleninic acid	-3.9136411	-	-	-	39.673	199.04394
NEG06591	5792459.938	8385816.807	0.533775	0.012539	0.2923	2.18086	Arbutin 6-phosphate	-9.704334	-	-	-	39.6527	351.220405
NEG06592	10892453.18	9308390.005	-0.22673	0.186224	0.6457	1.04197	Dehypoxanthine futalosine	-5.117645	-	-	-	39.6527	295.263807
NEG06596	306115.5127	489531.6486	0.677326	0.420009	0.7661	0.36921	Hexaflurate	6.1082793	-	-	-	39.642	227.004416
NEG06600	387821.9436	316511.6067	-0.29314	0.102012	0.563	0.32877	Pydanon	16.211601	-	-	-	39.6101	187.133873
NEG06601	241101.122	183206.1006	-0.39617	0.468912	0.7893	0.16713	Clavulanic acid	-18.351826	-	-	-	39.60645	198.149868
NEG06605	513027.4499	533987.8146	0.057771	0.665155	0.8796	0.04461	Methyl 2-propenyl selenide	-12.015452	-	-	-	39.56745	134.0611

NEG06607	1337837.054	1521410.836	0.185507	0.763516	0.9202	0.35666	Flutamide	-12.017375	-	-	-	39.5594	275.201304
NEG06615	189469.4661	242887.9733	0.358326	0.484238	0.798	0.20333	cis-(Homo)2-aconitate	-23.201935	-	-	-	39.4843	201.149433
NEG06629	1648961.391	1996039.363	0.275583	0.013758	0.3016	0.78693	Phaseolic acid	14.400749	-	-	-	39.40105	295.226589
NEG06635	185441.6868	221221.4319	0.254526	0.343661	0.73	0.20624	N-Acetylglucosamine 4-sulfate	-6.333409	-	-	-	39.37195	300.261815
NEG06637	793352.6201	727526.6516	-0.12496	0.407427	0.7587	0.18796	Tetrafluoroethylene	-13.674057	-	-	-	39.3605	99.0063558
NEG06639	458261.0783	488750.8801	0.09293	0.767094	0.9214	0.06347	(1E,3E)-4-Hydroxybuta-1,3-diene-1,2,4-tricarboxylate	10.662146	-	-	-	39.3605	201.113178
NEG06646	348826.8706	299735.7711	-0.21882	0.078444	0.5337	0.24268	Phosphotyrosine	14.525609	-	-	-	39.35645	260.164917
NEG06656	413199.9332	298985.9491	-0.46676	0.079797	0.536	0.33572	1,2-Bis(4-nitrophenyl)ethane	23.926973	-	-	-	39.3335	271.255338
NEG06658	3223889.273	3686052.995	0.193275	0.365585	0.7399	0.73809	Mefluidide	-18.81871	-	-	-	39.3322	309.279584
NEG06659	1234776.275	1768714.115	0.518451	0.054002	0.4853	0.81594	Convicine	6.3438384	-	-	-	39.3322	304.23606
NEG06660	1402077.067	1488059.281	0.085866	0.730123	0.9083	0.12804	O-Methylsterigmatocystin	21.628967	-	-	-	39.3322	337.310941
NEG06663	1349010.144	1541115.398	0.192074	0.250836	0.6861	0.4824	Chrysoeriol	10.045838	-	-	-	39.32485	299.25864
NEG06666	140641.8656	214590.2416	0.609558	0.081587	0.5377	0.29592	Selfotel	7.0924307	-	-	-	39.3065	222.157806
NEG06667	73811.96809	83645.00329	0.180425	0.617434	0.8569	0.06082	Catalposide	-3.449305	-	-	-	39.3065	481.425659
NEG06675	761076.9556	1012217.69	0.411405	0.174653	0.6336	0.50853	5-Nitrofurfural	-18.91695	-	-	-	39.2685	140.071655
NEG06677	833694.2292	592237.9334	-0.49334	0.02089	0.3487	0.57919	O-Phospho-L-serine	-20.686097	-	-	-	39.26815	184.061395
NEG06678	491329.5842	471637.7313	-0.05901	0.861994	0.9541	0.00613	S-(1,2-Dichlorovinyl)-L-cysteine	19.565488	-	-	-	39.2605	215.082551
NEG06683	694966.4531	670375.5346	-0.05197	0.453681	0.7814	0.12815	3-Hydroxy-2-methylpyridine-4,5-dicarboxylate	-18.061028	-	-	-	39.2511	196.134063
NEG06685	2553142.833	2870956.641	0.169257	0.113234	0.5714	0.60448	Diallat	1.4809255	-	-	-	39.2508	269.212224
NEG06686	782202.6427	923169.4005	0.239053	0.39042	0.751	0.25008	4-Nitrophenyl phosphate	2.3189465	-	-	-	39.2508	218.081931
NEG06688	433103.3794	388056.3135	-0.15845	0.410762	0.761	0.11182	Schradan	6.9474617	-	-	-	39.23605	285.243412
NEG06689	377448.9883	416899.1338	0.143417	0.392552	0.7513	0.10949	Methyl aklanote	4.5733004	-	-	-	39.2285	409.3681
NEG06694	242596.1163	297893.5576	0.29624	0.316735	0.7189	0.17446	Nicergoline	10.780886	-	-	-	39.21475	483.383946

NEG06695	621244.1563	724677.9713	0.22218	0.348233	0.7313	0.3277	Icilin	-5.5926683	-	-	-	39.2062	310.283082
NEG06698	390277.6358	356196.4886	-0.13183	0.477417	0.7932	0.08739	D-Erythroascorbic acid 1'-a-D-glucoside	-12.760755	-	-	-	39.2062	307.22749
NEG06700	902793.0623	1083539.762	0.263285	0.044942	0.45	0.53544	(Z)-4-(2-Hydroxy-5-sulfotophenyl)-2-oxo-3-butenoate	12.343856	-	-	-	39.20425	271.227384
NEG06703	46013.27881	40844.33012	-0.17191	0.603598	0.8497	0.06073	[(5,6,12,13,14-pentahydroxy-9-oxo-11-{6,7,13,14-tetrahydroxy[illegible]	-2.8193681	-	-	-	39.19095	693.470765
NEG06707	1040583.723	1007654.243	-0.04639	0.858272	0.9533	0.06395	DHAP(6:0)	11.762302	-	-	-	39.1736	267.196678
NEG06709	715789.3965	818533.0091	0.193505	0.506569	0.8064	0.26665	Apraclonidine	-14.258409	-	-	-	39.1736	244.097729
NEG06713	149657.1304	137092.7031	-0.12651	0.666948	0.8805	0.03917	Glucovanillin	-19.478108	-	-	-	39.1644	313.274502
NEG06714	38347747.97	1090104.252	-5.1366	0.019905	0.3429	7.93682	1D-1-Guanidino-3-amino-1,3-dideoxy-scyllo-inositol 4-phosphate	8.4481297	-	-	-	39.159	299.20146
NEG06717	558284.1664	526837.5774	-0.08364	0.701966	0.8994	0.10195	Ibandronate	-20.723237	-	-	-	39.15645	318.215008
NEG06721	358719.1577	398526.9673	0.151823	0.696216	0.8956	0.0832	Bortezomib	-24.452183	-	-	-	39.1499	383.220328
NEG06722	450795.6552	582878.9054	0.370723	0.005036	0.2001	0.50565	7-Methylxanthosine	-19.669147	-	-	-	39.1492	298.246837
NEG06723	12885541.04	16449330.28	0.352276	0.104987	0.5673	2.13443	Potassium dichromate	-6.2308636	-	-	-	39.1492	293.17549
NEG06727	574860.8152	556799.889	-0.04605	0.645804	0.8704	0.02891	S-(4-Bromophenyl)-mercaptopyruvate	-11.58811	-	-	-	39.1432	274.108635
NEG06728	137410.9486	94525.56808	-0.53972	0.226402	0.671	0.16825	(-)-Epiafzelechin 3-gallate	-5.5403245	-	-	-	39.1432	425.363261
NEG06729	2662778.072	3233136.181	0.280002	0.009143	0.2589	1.03566	Bowdichione	11.681006	-	-	-	39.1404	297.243207
NEG06730	2256123.849	2910378.259	0.36736	0.079635	0.5356	0.97653	Aminoimidazole ribotide	-1.8336319	-	-	-	39.1404	294.178582
NEG06732	669057.0008	684005.1385	0.031878	0.885324	0.961	0.09355	Tricrozarin A	19.216596	-	-	-	39.1404	293.212077
NEG06735	143605.475	101577.7608	-0.49953	0.024041	0.3593	0.26017	3-hydroxy-2-(hydroxymethyl)-2-[(sulfooxy)methyl]propan[illegible]	-7.4974218	-	-	-	39.1344	229.180998
NEG06736	314283.2116	328610.5532	0.064314	0.571755	0.8362	0.06997	5-Azacytidine	-4.9711508	-	-	-	39.1344	243.196209
NEG06738	1549994.19	1527377.964	-0.02121	0.880098	0.9586	0.1161	5-Hydroxyisourate	0.5653259	-	-	-	39.1256	183.102527
NEG06745	359439.2037	519431.3305	0.531185	0.693658	0.8938	0.17953	Trifluridine	-9.5804848	-	-	-	39.1114	295.189786
NEG06746	1231807.209	1172466.658	-0.07123	0.511251	0.8087	0.15495	3-{[(2E)-4-Amino-4-oxobut-2-enoyl]amino}-L-alanine	4.3639021	-	-	-	39.1114	200.173501
NEG06747	1626114.538	1681296.546	0.048145	0.777361	0.9242	0.11289	Podophyllotoxone	4.3983945	-	-	-	39.1114	411.383937

NEG06749	525289.4441	560770.5188	0.094298	0.557052	0.8301	0.14379	O-Phospho-4-hydroxy-L-threonine	-18.236954	-	-	-	39.1093	214.087301
NEG06752	450499.8556	418347.8634	-0.10682	0.573648	0.8369	0.08806	4-(Methylnitrosamino)-1-(3-pyridyl)-1-butanol glucuronide	-15.452662	-	-	-	39.0932	384.355868
NEG06753	2658066.484	2435281.599	-0.12629	0.361244	0.7374	0.39281	Endalin	15.636418	-	-	-	39.0932	228.204607
NEG06758	390644.8789	433352.3661	0.149683	0.581015	0.8399	0.0503	Dichlozoline	-18.123786	-	-	-	39.0866	273.087856
NEG06759	9944958.307	9562323.721	-0.0566	0.531323	0.8206	0.41252	Diamidafos	13.386167	-	-	-	39.0834	199.170203
NEG06760	1174882.547	1644923.62	0.485504	0.143344	0.6122	0.72997	Tricalcium phosphate	2.3964888	-	-	-	39.0834	309.170167
NEG06761	2444754.443	2320715.811	-0.07512	0.76602	0.9214	0.16092	Diflunisal	-18.299217	-	-	-	39.0834	249.185745
NEG06763	427437.5624	400612.5593	-0.09351	0.598543	0.8467	0.05897	Tetracenomycin M	7.5863298	-	-	-	39.08145	355.321527
NEG06764	18238665.46	16956421.89	-0.10517	0.404578	0.7582	0.91739	Pemirolast	-5.9427363	-	-	-	39.0802	227.201467
NEG06765	1886213.355	1831613.191	-0.04238	0.770728	0.9221	0.05685	[2-methoxy-4-(3,5,7-trihydroxy-3,4-dihydro-2H-1-benzopyran-2-	-0.2523198	-	-	-	39.0802	383.352626
NEG06766	3935160.323	4189198.701	0.090252	0.580008	0.8394	0.32305	2,5-Dioxopentanoate	5.1253318	-	-	-	39.0802	129.09209
NEG06774	1716751.204	1410377.939	-0.2836	0.100176	0.5617	0.56343	5-Acetylamino-6-formylamino-3-methyluracil	16.210307	-	-	-	39.07585	225.18579
NEG06779	137162.4644	79528.81788	-0.78634	0.034193	0.4099	0.27104	Melarsoprol	0.6100028	-	-	-	39.05825	397.331666
NEG06780	1094064.631	1016968.039	-0.10542	0.644265	0.8699	0.16709	L-Selenomethionine	18.100906	-	-	-	39.0559	195.102573
NEG06782	1407630.363	96209.14166	-3.87095	0.332566	0.7243	0.85187	3-Deoxy-D-manno-octulosote	4.497475	-	-	-	39.0531	237.185795
NEG06783	2499097.754	1998751.711	-0.32231	0.137442	0.6023	0.68208	(1R,6R)-6-Hydroxy-2-succinylcyclohexa-2,4-diene-1-carboxylate	-2.3929971	-	-	-	39.0527	239.201549
NEG06791	1280146.404	1088926.727	-0.2334	0.457017	0.7822	0.30071	Barbiturate	-19.553174	-	-	-	39.0303	127.076419
NEG06793	158542.8197	163552.0254	0.044877	0.878153	0.9584	0.02502	(2-{[2-(9-{[(2E)-2-methylbut-2-enoyl]oxy}-2-oxo-2H,8H,9H-furo[2,3	19.628302	-	-	-	39.03	481.462193
NEG06794	129814.1526	124382.851	-0.06166	0.840662	0.9478	0.03651	[3-(3,5,7-trihydroxy-6-methoxy-4-oxo-4H-chromen-2	8.578133	-	-	-	39.02995	395.316123
NEG06795	281007.8127	431114.045	0.617459	0.468482	0.7893	0.3753	rciclasine	10.056985	-	-	-	39.02995	306.251313
NEG06798	826461.888	843576.8804	0.029571	0.836808	0.9465	0.03433	2,3-Diketo-5-methylthiopentyl-1-phosphate	4.4665835	-	-	-	39.0266	241.180505
NEG06799	4649717.504	4034589.053	-0.20472	0.065777	0.5118	0.82261	3-hydroxy-2-methyl-2-[(sulfooxy)methyl]propanoic acid	14.663843	-	-	-	39.02655	213.185864
NEG06800	388141.7904	295444.1461	-0.3937	0.217669	0.6665	0.28183	Chlorogete	11.898549	-	-	-	39.0232	353.305639

NEG06801	627360.5098	581853.5976	-0.10864	0.622265	0.8589	0.18007	Alendronic acid	15.207743	-	-	-	39.0232	248.092512
NEG06803	41805.41311	50052.93693	0.259765	0.795702	0.9298	0.00832	Remoxipride	-6.1657998	-	-	-	39.0232	370.259434
NEG06806	2097105.638	2404060.996	0.197074	0.564539	0.8333	0.37195	Atoxin a(s)	2.7238295	-	-	-	39.0185	251.20141
NEG06807	1226174.915	977536.6029	-0.32694	0.116079	0.5757	0.56516	Meconic acid	11.400664	-	-	-	39.0138	199.097505
NEG06811	57477.17503	62170.52314	0.113242	0.824279	0.9407	0.0215	1-Nitro-7-hydroxy-8-glutathionyl-7,8-dihydronaphthalene	-11.435328	-	-	-	39.0017	495.478046
NEG06812	1246070.162	16439048.05	3.72167	0.000137	0.0947	5.81073	1-Methylseleno-N-acetyl-D-galactosamine	-7.2977981	-	-	-	38.987	297.185547
NEG06813	2511748.534	2271390.675	-0.14512	0.695382	0.8947	0.233	3,4,5-trihydroxy-6-[(2,3,5-trihydroxy-6-{[(3-methoxy-3	15.256192	-	-	-	38.9833	455.352686
NEG06815	1600735.283	1617912.04	0.015398	0.921499	0.9739	0.04431	6-Hydroxyprotopine	0.9384981	-	-	-	38.9833	368.36107
NEG06816	243796.5744	279076.8568	0.194985	0.761304	0.9195	0.08253	Quercetin 3-sulfate	23.323667	-	-	-	38.9833	381.30054
NEG06819	365093.2223	515055.7231	0.496464	0.211727	0.6624	0.31008	2-(2,4-dihydroxy-5-methoxyphenyl)-3,5,7-trihydroxy-4H-chromen-4	22.588508	-	-	-	38.96705	331.264229
NEG06828	929231.3188	1077622.508	0.213742	0.089268	0.5506	0.39739	Selenomethionine Se-oxide	-3.6313321	-	-	-	38.9264	211.097653
NEG06829	264741.2918	165858.3674	-0.67463	0.108106	0.5681	0.37341	2,2-Bis(4'-chlorophenyl)ethanol	-13.360391	-	-	-	38.9203	266.139654
NEG06831	7941628.166	6971706.913	-0.18792	0.041424	0.4403	1.08282	Angelicin	-9.0060645	-	-	-	38.90385	185.154547
NEG06836	137553.8072	225826.1593	0.715217	0.548349	0.8287	0.15483	2-(2,5-dihydroxy-4-methoxyphenyl)-3,5,6,7-tetrahydroxy-3,4-dihydro	6.879352	-	-	-	38.8874	349.274133
NEG06837	256421.9352	237503.3071	-0.11057	0.571916	0.8362	0.09383	Furamizole	-24.160992	-	-	-	38.8871	287.20146
NEG06839	643031.7941	501826.5322	-0.3577	0.187851	0.6466	0.31404	Triphenyl phosphate	-3.7293044	-	-	-	38.88515	325.274607
NEG06841	1591542.297	492447.7389	-1.69238	0.347953	0.7313	0.73972	Vicianose	-14.713116	-	-	-	38.87845	311.258629
NEG06846	906571.0002	1081129.623	0.254048	0.098098	0.5586	0.46771	3,4-Dihydroxymandelate	-0.1869307	-	-	-	38.85425	183.138789
NEG06847	1909859.855	21091459.74	3.46512	0.183323	0.642	4.67915	Inosine-5'-carboxylate	-19.505715	-	-	-	38.8529	311.222233
NEG06848	1108069.995	846074.56	-0.38919	0.019859	0.3429	0.61653	Nicotite D-ribonucleotide	-16.618713	-	-	-	38.8529	335.199036
NEG06850	493326.7505	507397.6808	0.040573	0.862193	0.9541	0.09566	hesperetin 3'-O-sulfate	5.6352368	-	-	-	38.8435	381.336878
NEG06851	785692.3143	696930.7568	-0.17295	0.549229	0.8287	0.16867	sn-Glycero-3-phospho-1-inositol	-0.9137557	-	-	-	38.8419	333.206718
NEG06853	2160417.76	1636310.861	-0.40086	0.03859	0.4318	0.9021	Tris(1-aziridinyl)phosphine oxide	-18.003746	-	-	-	38.839	172.142306

NEG06854	283199.1561	292276.2506	0.045516	0.890228	0.9634	0.04845	Silandrin	2.4749804	-	-	-	38.8365	465.430678
NEG06859	45093071.34	791868.872	-5.8315	0.021103	0.3495	8.61352	Urate-3-ribonucleoside	15.748273	-	-	-	38.827	299.222351
NEG06860	1126633.663	1211441.122	0.104706	0.561111	0.8325	0.20754	sn-Glycerol 3-phosphate	-0.478079	-	-	-	38.8175	171.066341
NEG06865	6159406.997	8717847.42	0.50118	0.004312	0.186	2.1569	2,3,4-trihydroxy-5-(3,4,5-trihydroxybenzoyloxy)benzoic acid	-7.1958083	-	-	-	38.8143	337.21429
NEG06869	1019072.567	672638.2196	-0.59935	0.1508	0.6145	0.69182	Isocil	-18.798958	-	-	-	38.8071	246.077178
NEG06874	221078.2889	157400.2757	-0.49012	0.562278	0.8325	0.16004	beta-D-3-[5-Deoxy-5-(dimethylarsinyl)ribofuranosyloxy]-2-hydroxy-1	9.7219851	-	-	-	38.79125	391.251537
NEG06883	111485.7966	130680.6996	0.229186	0.549509	0.8288	0.09607	({4-[4,6-dihydroxy-3-(6-hydroxy-3-oxo-2,3-dihydro-1-benzofuran-2	-16.261707	-	-	-	38.76865	451.415366
NEG06886	202361.1297	251853.6483	0.315653	0.448834	0.7796	0.16155	Tuliposide B	1.9822927	-	-	-	38.7661	293.248507
NEG06888	153057.9835	122779.7114	-0.31801	0.118292	0.5808	0.15066	Thiadiazolidinethione	-3.0553836	-	-	-	38.7661	341.269478
NEG06889	84511.17421	73114.21153	-0.20899	0.474953	0.7927	0.07016	Cyflufemid	3.3135265	-	-	-	38.7661	411.34729
NEG06891	216712.7593	155728.4001	-0.47675	0.033923	0.4077	0.31111	Nifuradene	17.00523	-	-	-	38.7604	223.170036
NEG06893	193778.6828	187107.5262	-0.05054	0.866094	0.9555	0.02143	Decarbamoylgonyautoxin 1	12.84212	-	-	-	38.7553	367.321153
NEG06895	248952.8816	227409.5853	-0.13058	0.874248	0.9574	0.1659	5-Hydroxydantrolene	9.760262	-	-	-	38.7547	329.248347
NEG06896	282654.6375	294557.2014	0.059507	0.859931	0.9535	0.0365	Cefacetrile	-7.8811296	-	-	-	38.7518	338.314049
NEG06902	141666.5866	331835.0474	1.227967	0.154446	0.6173	0.46465	Diclobutrazol	8.5467775	-	-	-	38.72945	327.232429
NEG06905	936727.1322	2123157.648	1.180511	0.321411	0.7192	1.04366	{[4-(7-methoxy-2-oxo-2H-chromen-6-yl)-2-oxobut-3-en-1-yl]oxy}sulfonic acid	-8.3336659	-	-	-	38.7142	339.289887
NEG06910	190878.3038	165238.1262	-0.20811	0.413023	0.7619	0.15653	Carboxyphosphamide	16.285446	-	-	-	38.7121	292.082296
NEG06914	988727.2041	1180504.485	0.255759	0.314606	0.7189	0.29735	Diethylphosphoric acid	-14.39965	-	-	-	38.71055	153.092004
NEG06923	3338057.776	3310379.182	-0.01201	0.925934	0.9755	0.00944	Fusarenone X	-6.7511817	-	-	-	38.69945	353.342131
NEG06924	164882.8125	117582.3398	-0.48777	0.098181	0.5586	0.25848	3,4,5-trihydroxy-6-{[3-(4-methoxy-1-benzofuran-5-yl)-3	19.731563	-	-	-	38.67165	409.33182
NEG06926	1225005.297	1388510.533	0.18075	0.868849	0.9556	0.04429	Wedelolactone	-2.4179017	-	-	-	38.64	313.238364
NEG06928	137573.4213	228883.3928	0.734411	0.246321	0.6842	0.34283	N-(6-Oxo-6H-dibenzo[b,d]pyran-3-yl)maleamic acid	5.9707318	-	-	-	38.6237	308.26747
NEG06930	18875109.22	27139209.24	0.523894	0.587449	0.8421	2.61585	Benzoyl meso-tartaric acid	-19.796407	-	-	-	38.6102	253.180591

NEG06932	877218.5882	1110369.916	0.340032	0.11204	0.5703	0.51874	Phenolic phosphate	-11.513015	-	-	-	38.57275	173.081819
NEG06933	3262718.34	3397366.802	0.058343	0.855916	0.9527	0.28849	(1S,3R)-3-(2,2-Dichloroethenyl)-2,2-dimethylcyclopropanecarb	-5.1790919	-	-	-	38.5671	208.061441
NEG06937	3660635.358	3774680.363	0.04426	0.724734	0.9063	0.12275	Phosphoenolpyruvate	0.2669894	-	-	-	38.53085	167.034768
NEG06943	78300.33932	165189.9104	1.077035	0.383932	0.7477	0.26749	UDP	-23.56302	-	-	-	38.5074	403.1444
NEG06949	179511.5073	150863.4903	-0.25083	0.490322	0.7998	0.09064	Azimsulfuron	-9.2165748	-	-	-	38.4687	423.383912
NEG06952	366136.1753	344725.8336	-0.08693	0.573213	0.8369	0.08046	Stipitatate	2.5859539	-	-	-	38.4662	181.123394
NEG06955	292552.7945	371178.7502	0.343417	0.139041	0.6058	0.29853	N-debutylhalofantrine	14.074485	-	-	-	38.4572	443.315977
NEG06962	441216.8957	387238.1323	-0.18827	0.230421	0.6743	0.20887	(4-{7-[3,5-dimethoxy-4-(sulfooxy)phenyl]-6,11-dihydroxy-2,8	20.673926	-	-	-	38.4153	526.483628
NEG06966	715467.4306	763225.8271	0.093224	0.700274	0.8985	0.1449	{2,6-dihydroxy-4-[(E)-2-(4-hydroxyphenyl)ethenyl]ph	7.2506683	-	-	-	38.3948	323.295075
NEG06970	428739.9923	383442.3674	-0.16109	0.396892	0.7532	0.17214	N-(3,5-Dichlorophenyl)succinimide	-2.2165996	-	-	-	38.3934	243.066282
NEG06976	164426.7613	269522.1924	0.712959	0.400407	0.7562	0.19955	3-Epihydroxymugineic acid	21.143714	-	-	-	38.3788	335.295034
NEG06977	429493.6564	210195.0232	-1.03091	0.384973	0.7478	0.51187	4-nitrophenolate	10.132248	-	-	-	38.35865	137.097123
NEG06978	629184.175	557437.8893	-0.17467	0.316062	0.7189	0.1967	(R)-5-Phosphomevalote	-3.5542177	-	-	-	38.3419	227.128913
NEG06980	573747.5237	331682.8264	-0.79061	0.410612	0.761	0.50921	EDTA	-9.7137093	-	-	-	38.33655	291.232485
NEG06986	1490752.35	1285164.067	-0.21409	0.837973	0.9468	0.10551	D-myo-Inositol 1,2-cyclic phosphate	-20.214626	-	-	-	38.304	241.108329
NEG07004	107863.7784	109025.926	0.015461	0.96809	0.9907	0.01477	{[1-(3-methoxyphenyl)-3-(2,4,6-trihydroxyphenyl)propan	3.860041	-	-	-	38.20405	369.364153
NEG07006	1398068.222	648902.2744	-1.10736	0.05589	0.4884	0.97683	2-Carboxy-2-hydroxy-8-carboxychromene	-0.2870146	-	-	-	38.1907	235.170256
NEG07008	880832.4078	559634.0909	-0.65438	0.008817	0.2551	0.78261	Purine mononucleotide	-24.689642	-	-	-	38.18805	331.191121
NEG07012	1684465.004	1679037.557	-0.00466	0.983575	0.9956	0.02297	4-Maleylacetoacetate	-21.685783	-	-	-	38.1625	199.133883
NEG07017	290263.0768	235745.8445	-0.30013	0.209367	0.6619	0.22503	Epigallocatechin 3-gallate	9.5534686	-	-	-	38.1495	457.368802
NEG07021	637407.8253	585262.9993	-0.12313	0.789341	0.9279	0.16595	D-Ribose 5-phosphate	22.156356	-	-	-	38.1392	229.107622
NEG07022	1338588.544	1396972.571	0.061591	0.819007	0.9375	0.10804	Chlorthiamid	8.5365251	-	-	-	38.1385	205.086783
NEG07023	604381.7434	542333.1854	-0.15628	0.439017	0.7739	0.2604	(-)-Epicatechin 3-O-gallate	10.315659	-	-	-	38.13655	441.373287

NEG07024	330686.8701	248310.1096	-0.41332	0.32635	0.7206	0.20348	3-[(2,6-Dichlorobenzylidene)amino]-6H-dibenzo[b,d]pyran	18.145836	-	-	-	38.1306	367.212205
NEG07027	746514.2704	219439.8677	-1.76634	0.006035	0.2182	0.99857	Ellagic acid	-15.632297	-	-	-	38.1064	301.180599
NEG07030	189307.6115	165419.7361	-0.1946	0.47674	0.7932	0.11554	Celecoxib glucuronide	-15.868763	-	-	-	38.0944	558.452845
NEG07031	690791.047	563221.1645	-0.29455	0.166834	0.6281	0.3797	[5-(Aminomethyl)furan-3-yl]methyl phosphate	22.173633	-	-	-	38.0863	206.118416
NEG07034	105029.7873	108007.8189	0.040337	0.889858	0.9633	0.04083	2-O-alpha-D-Galactopyranosyl-1-deoxynojirimycin	-19.856696	-	-	-	38.07865	324.298564
NEG07035	1994473.659	1889058.961	-0.07834	0.573477	0.8369	0.29063	Zwittermicin A	-1.6199238	-	-	-	38.0782	395.388881
NEG07039	244947.878	216312.5371	-0.17936	0.527687	0.8181	0.118	Aurasperone D	16.808066	-	-	-	38.07185	555.518377
NEG07040	470716.0622	406003.2131	-0.21337	0.648902	0.8721	0.11415	Savinin	-10.520774	-	-	-	38.07075	351.326417
NEG07043	405835.973	870938.9796	1.101675	0.015854	0.3144	0.79657	Tazobactam	7.7430881	-	-	-	38.0659	299.286149
NEG07044	937599.4349	1305502.173	0.477561	0.247062	0.6846	0.64642	Glycerone phosphate	0.8808707	-	-	-	38.0659	169.050673
NEG07045	1205400.607	1629769.909	0.435156	0.458239	0.7822	0.32342	Methylmercury chloride	6.9287373	-	-	-	38.0659	250.071963
NEG07048	84487.59302	80259.83373	-0.07406	0.762377	0.9199	0.0155	3,4-dihydroxy-5-[2,4,5-trihydroxy-3-(3,4,5-trihydroxybenzoyloxy)ben	1.489605	-	-	-	38.0633	489.322454
NEG07055	1159311.343	1345868.912	0.21527	0.790832	0.928	0.28853	3,4,5-trihydroxy-6-{[3-(6-hydroxy-7-methoxy-2H-1,3-benzodioxol-5	-15.657625	-	-	-	38.0045	415.321205
NEG07063	57009.53428	52892.98656	-0.10813	0.792003	0.9284	0.04426	6,11-dihydroxy-3-(hydroxymethyl)-7-(3,4,5-trihydroxyphenyl)-28	-15.73848	-	-	-	37.94995	356.2801
NEG07067	347218.2817	174680.9017	-0.99112	0.002365	0.1309	0.56056	Quinclorac	-2.0680014	-	-	-	37.9355	241.050423
NEG07071	153614.755	86364.92727	-0.8308	0.017015	0.3231	0.28678	2,3,7,8-Tetrachlorodibenzodioxin	-0.8700496	-	-	-	37.9276	320.963443
NEG07076	108473.0154	213222.7698	0.975025	0.01707	0.3231	0.38133	Graticin	18.346264	-	-	-	37.8978	443.389076
NEG07077	130009.2749	194721.4556	0.582797	0.083019	0.5411	0.24988	Morfamquat	-6.3332163	-	-	-	37.88795	538.483707
NEG07079	354411.9535	345858.7389	-0.03524	0.858051	0.9533	0.03124	D-Lombricine	9.5511488	-	-	-	37.8742	269.175604
NEG07091	605438.1378	406867.6722	-0.57342	0.255117	0.6872	0.41962	Lamotrigine	13.968472	-	-	-	37.8309	255.087301
NEG07095	373518.5912	314161.7828	-0.24967	0.012791	0.2932	0.29588	Brimonidine	-12.494119	-	-	-	37.79655	291.123673
NEG07096	619994.1456	346919.6443	-0.83765	0.006822	0.2329	0.73532	2-(alpha-D-Mannosyl)-3-phosphoglycerate	-12.254704	-	-	-	37.79605	347.186256
NEG07110	96334.29662	213092.0168	1.145355	0.440935	0.7749	0.21071	6-[2,3-Dihydroxy-1-(hydroxymethyl)propyl]-1,2-dihydro-7-hydroxy-9	-5.3921943	-	-	-	37.7377	349.310834

NEG07111	469059.135	340277.6853	-0.46306	0.050717	0.4758	0.42911	3,5-Dinitro-4-hydroxyphenylpyruvate	-24.123555	-	-	-	37.7262	269.138706
NEG07114	337641.6793	310008.4236	-0.12319	0.591736	0.8437	0.0835	Decuroside III	2.275189	-	-	-	37.69815	569.533821
NEG07115	1175344.794	899875.3856	-0.38529	0.459397	0.7829	0.32576	3,5-Dichloro-L-tyrosine	21.051467	-	-	-	37.68965	249.076688
NEG07127	594934.9943	529485.7864	-0.16814	0.375706	0.7438	0.25549	Isopentenyl diphosphate	-13.456007	-	-	-	37.6338	245.081512
NEG07134	314475.5666	199575.6984	-0.65601	0.23613	0.6796	0.37341	3'-Sialyllactose	-21.19277	-	-	-	37.60455	632.530397
NEG07135	472500.7669	309079.2571	-0.61234	0.037275	0.4235	0.53147	6-Phospho-D-glucote	2.1401234	-	-	-	37.6037	275.128514
NEG07138	494435.5096	334516.6069	-0.5637	0.037227	0.4235	0.53297	6-Phospho-2-dehydro-D-glucote	4.5482474	-	-	-	37.57805	273.11327
NEG07141	674069.9894	657514.5647	-0.03588	0.869391	0.956	0.03816	2,2',4,4',5,5'-Hexabromodiphenyl ether	-13.372393	-	-	-	37.54755	642.567717
NEG07143	243246.1294	538814.9933	1.147373	0.150045	0.6145	0.58924	Digallate	9.5051516	-	-	-	37.5457	321.219586
NEG07147	982367.6919	700746.598	-0.48737	0.258372	0.6898	0.58367	beta-D-4-Deoxy-delta4-GlcA-(1->4)-beta-D-Glc-(1->4)-alpha-L [illegible]	4.2839201	-	-	-	37.535	645.541993
NEG07150	555201.6648	422851.5776	-0.39286	0.292824	0.7099	0.39093	Prebetanin	-21.271286	-	-	-	37.517	629.511311
NEG07151	148337.2218	311516.1801	1.070426	0.308146	0.7158	0.36656	Bicuculline	2.0264809	-	-	-	37.5166	366.345568
NEG07153	212732.307	219325.4172	0.044034	0.859712	0.9535	0.02832	Pteroyltriglutamic acid	16.511755	-	-	-	37.5071	698.629675
NEG07156	276141.3094	173266.7301	-0.67241	0.153314	0.6159	0.37378	Heme	24.591808	-	-	-	37.50245	615.495184
NEG07160	320402.2634	279090.8647	-0.19915	0.648889	0.8721	0.12419	5-(methylsulfanyl)-2,3-dioxopentyl Phosphate(2-)	9.0203861	-	-	-	37.4937	239.16489
NEG07161	297614.1021	223917.9902	-0.41047	0.035162	0.4158	0.33748	Isoimide	-24.419547	-	-	-	37.4937	305.139847
NEG07164	334824.7785	398785.6179	0.252207	0.437462	0.7726	0.19717	S-(N-Hydroxy-N-methylcarbamoyl)glutathione	-23.952921	-	-	-	37.4899	379.357812
NEG07166	219505.275	261454.4584	0.252304	0.326035	0.7202	0.18	Petunidin 3-gentiobioside	12.334487	-	-	-	37.48385	640.552037
NEG07167	1320158.455	273073.4362	-2.27335	0.023729	0.3593	1.29761	3',5'-Cyclic IMP	-23.12891	-	-	-	37.4744	329.175786
NEG07169	843657.0692	529418.1029	-0.67225	0.268141	0.6952	0.51195	Acetyl phosphate	-11.341221	-	-	-	37.4677	139.023035
NEG07171	564846.8022	379545.7248	-0.57359	0.19448	0.6521	0.48662	3,4,5-trihydroxy-6-(2-hydroxy-5-{3,5,6,7-tetrahydroxy-8-[3,4,5 [illegible]	-1.5068762	-	-	-	37.447	643.526752
NEG07175	229244.3065	160866.457	-0.51102	0.322936	0.7192	0.26682	Aldotetraouronic acid	-10.014627	-	-	-	37.4437	603.496469
NEG07176	89865.85426	62274.19036	-0.52914	0.203565	0.6615	0.16344	N-Glycoloyl-neuramite	-15.666435	-	-	-	37.4421	324.256928

NEG07178	2600285.883	15305188.88	2.557279	0.000952	0.0947	5.05582	Bis(4-nitrophenyl)phosphate	18.703959	-	-	-	37.43795	339.181286
NEG07179	558313.9973	383681.2265	-0.54117	0.061648	0.5018	0.44899	(2R)-O-Phospho-3-sulfolactate	-3.1599389	-	-	-	37.4376	249.112933
NEG07185	2390338.297	2367612.472	-0.01378	0.929741	0.9763	0.01893	[2-ethyl-5-(3,5,7-trihydroxy-3,4-dihydro-2H-1-benzopyran-2	1.593486	-	-	-	37.3713	381.373333
NEG07189	562343.8133	504341.2044	-0.15705	0.517761	0.8122	0.25241	Chloranocryl	14.918837	-	-	-	37.3573	229.086756
NEG07193	760809.1453	547346.5932	-0.47508	0.107078	0.5681	0.49666	Pyridoxamine phosphate	17.881929	-	-	-	37.3489	247.170161
NEG07194	53695.89923	49827.36453	-0.10787	0.783988	0.9268	0.02206	Cefuroxime axetil	-20.130449	-	-	-	37.3489	509.456947
NEG07196	424349.3929	363141.5975	-0.22472	0.299386	0.7148	0.21624	Pelargonidin 3-O-rutinoside	-7.4109799	-	-	-	37.33335	578.515029
NEG07198	455933.2271	346149.9115	-0.39743	0.44525	0.7774	0.32904	D-Erythritol 4-phosphate	-1.6743189	-	-	-	37.32925	201.092085
NEG07204	590889.6149	621701.9088	0.073334	0.661743	0.8779	0.09668	Niazimin A	-23.336609	-	-	-	37.30825	382.376776
NEG07205	202387.0659	131617.9585	-0.62076	0.222824	0.6692	0.30293	Myricetin 3,3'-digalactoside	3.457135	-	-	-	37.30065	641.511245
NEG07208	190751.1657	547772.8276	1.521886	0.003385	0.1597	0.8003	Streptidine 6-phosphate	14.694342	-	-	-	37.28585	341.243952
NEG07209	166054.4714	95530.57678	-0.79762	0.209109	0.6619	0.32502	3,5-Dihydroxyphenyl 1-O-(6-O-galloyl-beta-D-glucopyranoside)	22.596941	-	-	-	37.2841	439.357574
NEG07211	169156.6172	1047335.854	2.630293	0.141016	0.6076	0.96805	Chlomethoxyfen	-16.518853	-	-	-	37.2823	313.108434
NEG07214	76464.38773	154670.5283	1.016338	0.39936	0.7555	0.19091	2-(4-benzoyl-3,5-dihydroxyphenoxy)-6-(hydroxymethyl)oxane	13.343759	-	-	-	37.2805	391.357959
NEG07216	372453.4347	393147.1544	0.078009	0.732277	0.9091	0.10142	Justicidin A	16.726875	-	-	-	37.2397	393.37342
NEG07218	112436.5193	102391.0018	-0.13502	0.621589	0.8589	0.05512	Lippioside II	22.33004	-	-	-	37.2202	553.502405
NEG07220	106197.74	80890.54218	-0.39271	0.515825	0.8114	0.09192	Lamivudine-monophosphate	-19.172898	-	-	-	37.1953	308.222794
NEG07224	39679.78481	93403.84293	1.235078	0.424636	0.7672	0.14716	Ipolamiide	-2.208059	-	-	-	37.18775	405.373526
NEG07229	814336.9569	776666.5559	-0.06833	0.737006	0.9117	0.1079	5'-Hydroxystreptomycin	-6.8173589	-	-	-	37.1858	596.56215
NEG07232	136817.8593	147077.6373	0.104321	0.676464	0.8854	0.0606	Pelargonidin 3-O-beta-D-sambubioside	11.27512	-	-	-	37.1686	564.499199
NEG07237	605665.1073	584713.4932	-0.05079	0.832795	0.9447	0.13963	3-Phospho-D-erythrote	-21.418448	-	-	-	37.16585	215.071295
NEG07244	542701.1549	391064.2755	-0.47275	0.32784	0.721	0.42309	({5-[(2-{[5,7-dihydroxy-2-(4-hydroxyphenyl)-4-oxo-4H-chromen-3-yl]oxy}	23.111536	-	-	-	37.1501	659.55799
NEG07246	37096.3757	49238.41254	0.408506	0.510631	0.8083	0.08088	Celecoxib	-9.4346944	-	-	-	37.1449	380.361325

NEG07247	142219.7443	117198.5724	-0.27917	0.480547	0.7955	0.1455	Isosorbide dinitrate	-22.570973	-	-	-	37.13115	235.123694
NEG07252	83802.41631	71377.43842	-0.23152	0.470612	0.7902	0.06891	11-[(6-carboxy-3,4,5-trihydroxyoxan-2-yl)oxy]-3-(3,4-dihydroxyphenyl)-	3.6035582	-	-	-	37.1007	638.536028
NEG07256	493981.0164	475725.0137	-0.05433	0.820893	0.9386	0.06074	[(6-{[5,7-dihydroxy-2-(4-oxocyclohexa-2,5-dien-1-ylidene)-2H-chromen-3	17.249372	-	-	-	37.0895	608.562238
NEG07265	168628.5665	182565.3085	0.114564	0.727628	0.9075	0.05614	Aplysiatoxin	-10.777587	-	-	-	37.02265	670.599085
NEG07270	295679.3288	167255.7249	-0.82198	0.008067	0.25	0.47419	M1	16.27569	-	-	-	36.98735	303.123673
NEG07275	141587.6205	124042.7301	-0.19086	0.618797	0.8572	0.07167	Flupyrsulfuron-methyl sodium	-2.2286738	-	-	-	36.9654	487.342535
NEG07276	90116.98067	103839.115	0.204479	0.562036	0.8325	0.07293	ringin	-15.693318	-	-	-	36.9654	579.518213
NEG07280	302897.6101	208349.1244	-0.53983	0.373635	0.7428	0.32324	({6-[5,7-dihydroxy-2-(3-hydroxyphenyl)-4-oxo-3-[3,4,5-trihydroxy-6	0.3314735	-	-	-	36.9622	673.572947
NEG07284	193756.5232	190075.6639	-0.02767	0.950522	0.9834	0.0514	Caloxetate trisodium	-11.189474	-	-	-	36.95795	630.514857
NEG07285	212628.0991	209513.0212	-0.02129	0.925852	0.9755	0.00389	Malvin	-4.1359822	-	-	-	36.95795	654.568012
NEG07288	533757.751	334276.8882	-0.67514	0.365514	0.7399	0.39202	2-Oxo-3-hydroxy-4-phosphobutanoate	-20.787751	-	-	-	36.95115	213.055573
NEG07289	185387.3342	118048.7485	-0.65116	0.230387	0.6743	0.30136	3''-Adenylylspectinomycin	20.212195	-	-	-	36.95115	660.561595
NEG07295	2705062.103	2234901.061	-0.27545	0.002344	0.1309	0.97696	Arsenobetaine	9.7247124	-	-	-	36.9343	177.055655
NEG07296	220067.1499	139492.4086	-0.65776	0.213037	0.6634	0.32167	Patuletin 3-gentiobioside	-12.685554	-	-	-	36.90965	655.527295
NEG07298	132636.3402	140125.8897	0.079247	0.743364	0.9124	0.06106	Phosacetim	-7.7973804	-	-	-	36.8958	374.199798
NEG07302	179624.2096	113124.5071	-0.66707	0.125475	0.5916	0.32708	1-alkyl-2-acylglycerophosphoethanolamine	13.849831	-	-	-	36.8589	242.148091
NEG07304	349451.8082	136516.5745	-1.35602	0.006564	0.2295	0.64545	D-Glucono-1,5-lactone 6-phosphate	22.350978	-	-	-	36.8589	257.118393
NEG07306	41837.41661	87645.26143	1.066882	0.369791	0.7408	0.14748	[1-(2H-1,3-benzodioxol-5-yl)-3-(4-methoxy-1-benzofuran-5-yl)-3	15.871299	-	-	-	36.8579	419.389396
NEG07310	74695.83558	92085.11485	0.30194	0.53754	0.8243	0.10441	(3,5-dihydroxy-2-{5-hydroxy-7-methoxy-4-oxo-2-phenyl-6-[3,4,5	-2.4636097	-	-	-	36.85105	671.601066
NEG07313	132986.9585	137849.8537	0.051813	0.835893	0.9465	0.02024	Neohesperidin dihydrochalcone	18.972993	-	-	-	36.8438	611.580746
NEG07317	43475.83848	57171.38857	0.395079	0.450416	0.7801	0.08833	Tribenuron methyl	-15.874784	-	-	-	36.8419	394.376847
NEG07319	300321.0375	283212.5309	-0.08462	0.742012	0.9121	0.05031	Apigenin 7-(6''-malonylglucoside)	-12.206268	-	-	-	36.8419	517.410095
NEG07322	2013932.786	1034493.321	-0.96109	0.155557	0.6182	1.13634	3,4,5-trihydroxy-6-[(2-hydroxyacetyl)oxy]oxane-2-carboxylic acid	-10.714893	-	-	-	36.8365	251.165021

NEG07326	215572.9949	141015.7653	-0.61232	0.331663	0.7236	0.29606	({3,4,5-trihydroxy-6-[5- hydroxy-2-(4- hydroxyphenyl)-7	-20.283115	-	-	-	36.8261	687.588756
NEG07327	68931.50922	55191.48225	-0.32072	0.199463	0.6571	0.11876	3,4,5-trihydroxy-6- ({11,17,18-trimethoxy-13- oxo-6,8,20	-0.7379098	-	-	-	36.8258	559.45631
NEG07329	225195.6943	144934.9238	-0.63577	0.282451	0.7021	0.32171	[2-({6- [(acetyloxy)methyl]-4,5- dihydroxy-3-{[5-hydroxy	-13.416096	-	-	-	36.8258	685.573512
NEG07330	175549.3203	151569.0671	-0.2119	0.421801	0.7663	0.11504	Hesperidin	19.075341	-	-	-	36.8255	609.56497
NEG07331	634426.3571	654126.6067	0.044117	0.96123	0.9883	0.03461	beta-Chloro-alpha- phenylbenzeneethamine hydrochloride	4.2421639	-	-	-	36.82495	267.175461
NEG07336	235514.5502	207136.9405	-0.18523	0.516411	0.8117	0.08948	Diosmin	19.97849	-	-	-	36.8238	607.549581
NEG07341	342489.3661	289621.7228	-0.24189	0.374144	0.7431	0.13823	SN38 glucuronide	-6.7045276	-	-	-	36.8028	567.517612
NEG07343	155444.1581	164523.3638	0.081896	0.839531	0.9472	0.02611	3,5-Dinitrosalicylic acid	-4.3899931	-	-	-	36.7897	227.107622
NEG07344	428053.9345	444841.7021	0.055499	0.953332	0.9849	0.13253	3,4,5-trihydroxy-6-({7- methoxy-4-oxo-2-phenyl- 8-[3,4,5-trihydroxy-6	12.078429	-	-	-	36.7861	605.533049
NEG07345	86238.27567	88833.60484	0.042777	0.811537	0.9348	0.01229	Kuwanon L	-3.8588553	-	-	-	36.7861	625.596405
NEG07347	267328.4864	241635.2258	-0.14578	0.431801	0.77	0.11896	Uracil	17.546114	-	-	-	36.7748	111.08149
NEG07350	308511.9596	220895.3946	-0.48196	0.045028	0.45	0.36542	6-({3,5-dihydroxy-2-[3- hydroxy-10-(3- methoxyphenyl)-2-oxo-4	-11.333559	-	-	-	36.7603	643.567418
NEG07351	886038.1839	899893.6267	0.022386	0.933341	0.9777	0.0229	Benzethonium chloride	-22.273066	-	-	-	36.7533	447.063643
NEG07353	235517.2123	435966.8765	0.888386	0.349402	0.7322	0.46507	1-Carboxyvinyl carboxyphosphote	1.1611374	-	-	-	36.7345	195.045051
NEG07354	62454.01656	39965.25997	-0.64405	0.143508	0.6122	0.16923	3-Methylellagic acid 2-(4- galactosylglucoside)	-13.534594	-	-	-	36.7345	653.510865
NEG07361	127023.7995	130500.3121	0.038954	0.899764	0.9672	0.01377	rcobarbital	-4.622193	-	-	-	36.6962	302.143722
NEG07362	99229.28209	197633.8123	0.993992	0.396221	0.7528	0.19386	Ritipenem acoxil hydrate	-14.971783	-	-	-	36.6861	377.342059
NEG07363	713585.1763	895008.387	0.326816	0.627076	0.8608	0.3769	alpha-1,5-L-Arabinotriose	-15.079285	-	-	-	36.6828	413.345575
NEG07365	96147.8323	97622.67499	0.021962	0.866523	0.9556	0.01474	Hellicoside	-14.671612	-	-	-	36.6814	655.56899
NEG07366	150660.2331	94113.2692	-0.67883	0.253249	0.6865	0.26855	6-({3-[5,7-dihydroxy-2-(3- hydroxyphenyl)-4-oxo- 3,4-dihydro-2H-1	-13.281703	-	-	-	36.6814	701.604391
NEG07369	416896.4497	457936.3795	0.135458	0.708378	0.9015	0.14901	Propyzamide	13.440355	-	-	-	36.65995	255.123966
NEG07370	70974.38883	72735.27817	0.035357	0.907191	0.9697	0.03494	Pelargonidin 3-O-(6-O- malonyl-beta-D-glucoside)	-20.402793	-	-	-	36.65425	518.413726
NEG07376	120789.3178	94563.53286	-0.35314	0.037234	0.4235	0.21014	(2-{5,7-dihydroxy-4-oxo- 2-phenyl-3-[3,4,5- trihydroxy-6	18.010346	-	-	-	36.6321	657.584585

NEG07379	171204.8243	185877.7317	0.118631	0.765785	0.9213	0.04564	Ginkgolide A	-7.2405252	-	-	-	36.6215	407.388966
NEG07381	97962.24836	112688.5307	0.202043	0.402612	0.7574	0.10043	Forsythiaside	1.0306635	-	-	-	36.6215	623.580467
NEG07384	169447.5333	183420.9496	0.11432	0.663258	0.8787	0.0687	2-amino-4-({1-[(carboxymethyl)-C-hydroxycarbonimidoyl]-2	-1.3871114	-	-	-	36.621	620.561861
NEG07391	63595.81607	65278.62074	0.037679	0.917756	0.9729	0.01807	Tris(2,3-dibromopropyl) phosphate	15.461737	-	-	-	36.6062	696.61431
NEG07394	332599.2429	245506.8105	-0.43802	0.006259	0.2196	0.38207	D-Erythrose 4-phosphate	-0.6393549	-	-	-	36.5788	199.076395
NEG07398	502349.2916	505738.4877	0.009701	0.982354	0.9949	0.063	L-Fuculose 1-phosphate	-22.337743	-	-	-	36.55255	243.12367
NEG07399	122414.5792	74383.22655	-0.71873	0.242637	0.6822	0.25004	(S)-Skyrin 2-glucoside	-3.381193	-	-	-	36.5361	699.588855
NEG07405	61174.07977	50144.27329	-0.28684	0.390318	0.751	0.07448	5-[(6-{[(2-carboxyacetyl)oxy]methyl}-3,4,5-trihydroxyoxan-2	22.609685	-	-	-	36.5222	680.583134
NEG07407	67181.19175	58174.50658	-0.20767	0.462505	0.7842	0.08096	3-Methylquercetin 7-[galactosyl-(1->4)-glucoside]	-2.1787822	-	-	-	36.5083	639.534828
NEG07410	512017.2057	481721.5441	-0.08799	0.711155	0.9019	0.05662	({2,3,4-trihydroxy-6-oxo-3-[3,4,5-trihydroxy-6-(hydroxymethyl)oxan-2	-2.0889521	-	-	-	36.5082	545.441582
NEG07411	47300.28491	62976.09897	0.412956	0.428648	0.7691	0.0801	2-O-beta-D-Glucopyranuronosyl-D-mannose	-23.766182	-	-	-	36.4987	355.264256
NEG07415	83823.22454	79588.38566	-0.07479	0.782057	0.9257	0.01132	Aflatoxin B1exo-8,9-epoxide-GSH	-18.372082	-	-	-	36.4891	634.577546
NEG07416	330457.1982	247597.4036	-0.41647	0.229698	0.6734	0.23994	10-Acetoxyligustroside	-18.822869	-	-	-	36.4861	581.532258
NEG07418	406663.9715	407811.8042	0.004066	0.993041	0.9985	0.09456	Endothal-disodium	20.341421	-	-	-	36.4831	229.123104
NEG07419	296317.4743	261571.8162	-0.17994	0.704927	0.8996	0.15543	Oxapyrazon	4.9149321	-	-	-	36.4831	337.107985
NEG07420	63442.90473	51812.69326	-0.29215	0.316739	0.7189	0.07076	Orobanchoside	1.1178878	-	-	-	36.4831	621.564719
NEG07426	50515.19812	51736.16004	0.034455	0.905223	0.9695	0.01387	Kanokoside C	-13.326652	-	-	-	36.4795	637.596413
NEG07427	301040.3132	337814.3455	0.166274	0.861933	0.9541	0.05293	Phosphoguanidinoacetate	-12.789738	-	-	-	36.4787	196.076703
NEG07431	271315.3915	206881.5382	-0.39117	0.072333	0.5226	0.2765	D-Mannitol 1-phosphate	19.802534	-	-	-	36.47745	261.149615
NEG07434	82371.17063	138559.151	0.750291	0.432994	0.77	0.14463	Bicalutamide	16.699325	-	-	-	36.45445	429.37331
NEG07437	66167.50123	67857.62943	0.036388	0.856751	0.9533	0.00603	Cefoperazone sodium	-2.9961049	-	-	-	36.451	666.639923
NEG07444	279413.7033	190903.3469	-0.54956	0.313529	0.7189	0.20818	Erythrityl Tetranitrate	17.437843	-	-	-	36.4258	301.107992
NEG07447	154619.0209	150605.7011	-0.03794	0.863793	0.9547	0.00513	(S)-2,3-Dihydro-3,5-dihydroxy-2-oxo-3-indoleacetic acid 5	-19.820968	-	-	-	36.41225	546.445272

NEG07450	215470.1723	190422.0218	-0.17829	0.539364	0.8256	0.10832	Se-Adenosylselenomethionine	-2.5182298	-	-	-	36.3987	445.331899
NEG07452	685503.319	464018.3663	-0.56298	0.678292	0.8861	0.12109	Caffeoyl aspartic acid	20.5724	-	-	-	36.3906	294.243597
NEG07456	263092.0703	229785.0414	-0.19528	0.343603	0.73	0.18266	Luteoskyrin	-16.503189	-	-	-	36.3751	573.471743
NEG07457	1362449.929	1330459.29	-0.03428	0.860183	0.9535	0.06263	Halobetasol Propionate	10.471414	-	-	-	36.3716	483.957802
NEG07462	347272.9589	318328.7138	-0.12555	0.772054	0.9222	0.16199	2-epi-5-epi-Valiolone 7-phosphate	-19.622914	-	-	-	36.346	271.133883
NEG07464	106218.0069	58770.7245	-0.85386	0.024337	0.3595	0.28785	Panfuran S	0.4070962	-	-	-	36.3384	292.228343
NEG07471	217057.224	194697.1341	-0.15684	0.597533	0.8464	0.0791	Ammeline	24.372317	-	-	-	36.29	126.100521
NEG07473	111594.0395	186584.5538	0.74157	0.512554	0.8101	0.1808	trans-1,2-Dichloroethene	-15.525365	-	-	-	36.2849	95.9345183
NEG07480	506262.9866	300481.7753	-0.75261	0.167897	0.6287	0.42523	2,4-DB	-12.414382	-	-	-	36.257	248.080231
NEG07481	321355.5302	332562.9827	0.049457	0.841295	0.9478	0.03802	Glucosinalbin	11.093695	-	-	-	36.257	423.420432
NEG07483	112187.3841	66940.6352	-0.74496	0.023206	0.3593	0.26921	Limocitrin 3-rutinoside	-20.820563	-	-	-	36.25335	653.549195
NEG07488	366233.4299	363570.7627	-0.01053	0.971744	0.9919	0.0189	Molybdate	21.278755	-	-	-	36.19605	164.986255
NEG07493	249756.0195	225924.6093	-0.14468	0.335962	0.7266	0.17205	1,2-Dichloropropane	6.1542433	-	-	-	36.16025	111.979119
NEG07498	691552.8062	752181.9859	0.121242	0.537502	0.8243	0.16627	Bruceine D	-7.2081655	-	-	-	36.1522	409.404765
NEG07512	96094.67058	104171.6795	0.116435	0.764908	0.9208	0.04797	Fosphenytoin	-20.012691	-	-	-	36.12715	361.259373
NEG07514	56272.45631	64267.88515	0.191669	0.782588	0.926	0.02594	Boc-Asn-OPhNO2	4.4801931	-	-	-	36.11615	352.321606
NEG07518	385713.952	485955.6338	0.333293	0.345835	0.7309	0.31027	Corilagin	-14.092174	-	-	-	36.0817	633.436583
NEG07523	373558.8002	277925.2301	-0.42664	0.353136	0.7326	0.34558	Reduced 2,6-Dichlorophenolindophenol	-0.8657329	-	-	-	35.9866	269.10389
NEG07525	145098.7535	184017.8129	0.34281	0.063852	0.5055	0.22679	UDP-2,4-bis(acetamido)-2,4,6-trideoxy-beta-L-altropyranose	1.5732649	-	-	-	35.9866	631.399918
NEG07529	340556.4102	324752.5653	-0.06855	0.809271	0.9347	0.08805	Kolaflavanone	-11.298099	-	-	-	35.97005	587.501174
NEG07533	121403.764	99112.68034	-0.29267	0.237526	0.6801	0.11405	Oxaziclomefone	15.210457	-	-	-	35.9536	375.274847
NEG07534	173977.0761	140968.8118	-0.30352	0.462583	0.7842	0.16405	L-Threonylcarbamoyladenylate	20.031139	-	-	-	35.9482	491.337185
NEG07541	547711.9827	597521.8953	0.125574	0.809823	0.9347	0.11559	Dimethyl selenide	-0.0135414	-	-	-	35.92125	108.021722

NEG07542	70326.81373	77421.76394	0.138664	0.609201	0.8528	0.04996	{4,11-dihydroxy-17-methoxy-10-oxo-2-oxatricyclo[13.2.2.1icosa	-15.402508	-	-	-	35.9191	437.43597
NEG07546	321729.0127	347492.42	0.111135	0.424902	0.7672	0.14252	Monobromobisphenol A	-14.282771	-	-	-	35.8563	306.170736
NEG07548	79602.75766	83783.66578	0.073851	0.775723	0.9235	0.01091	Felodipine	-8.2712302	-	-	-	35.8506	383.243545
NEG07558	1085362.761	533705.6321	-1.02406	0.142739	0.6112	0.84181	Glyphosine	-0.6597413	-	-	-	35.8166	262.07215
NEG07559	867342.7935	1115280.164	0.362732	0.58417	0.8415	0.37564	2-Oxo-4-phosphonobutanoate	2.7350234	-	-	-	35.8158	181.061721
NEG07563	482321.0458	497471.7714	0.044621	0.899591	0.9672	0.06466	3,4,5-trihydroxy-6-(6-hydroxy-7-methoxy-2H-1,3-benzodioxole-5	3.0661963	-	-	-	35.8154	387.274914
NEG07564	225399.1078	298130.0672	0.40346	0.363279	0.7392	0.17475	Methylgallic acid-O-sulphate	-1.0360219	-	-	-	35.8147	263.20145
NEG07566	231678.2198	254565.0228	0.135912	0.841592	0.9478	0.03425	Phosphoenol-4-deoxy-3-tetrulosote	0.888986	-	-	-	35.8136	197.060799
NEG07568	86625.63129	81737.14287	-0.0838	0.622282	0.8589	0.05207	Perfluorohexane sulfonic acid	14.00977	-	-	-	35.8068	399.113329
NEG07569	152062.7976	105594.2004	-0.52614	0.303667	0.7152	0.2053	Antrafenine	-19.029858	-	-	-	35.806	587.525024
NEG07570	108386.7316	105034.3386	-0.04533	0.775861	0.9235	0.02154	Arnamiol	-22.582368	-	-	-	35.8026	449.93454
NEG07573	356524.8416	90065.39312	-1.98496	0.240926	0.6822	0.41274	Bropirimine	1.3018654	-	-	-	35.79365	265.08707
NEG07578	351363.8916	217339.5162	-0.69302	0.073695	0.5234	0.40122	Sanguisorbic acid dilactone	14.804637	-	-	-	35.7814	469.295986
NEG07582	468811.588	391705.8574	-0.25924	0.420953	0.7661	0.21235	2,6-Dichloroindophenol	7.7363038	-	-	-	35.7522	267.090297
NEG07585	147483.6471	111754.335	-0.40022	0.227825	0.6714	0.18223	CDP-choline	-21.463372	-	-	-	35.74865	487.306242
NEG07589	892657.6847	952880.8459	0.094189	0.729733	0.9083	0.17138	Dimethyl 2-galloylgalactarate	4.3405913	-	-	-	35.7342	389.290618
NEG07590	80494.41216	60841.47914	-0.40383	0.330593	0.7228	0.15592	Quercetin 3-O-beta-D-glucosyl-(1->2)-beta-D-glucoside	10.00432	-	-	-	35.7243	625.515891
NEG07594	68513.35907	64964.93646	-0.07672	0.744962	0.9131	0.02446	Armillaridin	18.189528	-	-	-	35.6796	447.936889
NEG07599	287849.2638	243127.6908	-0.2436	0.490169	0.7998	0.17185	4-(4-Hydroxyphenyl)-2-butanone O-[2,6-digalloylglucoside]	9.7013974	-	-	-	35.6663	629.549041
NEG07600	396709.8376	195758.8864	-1.01901	0.012581	0.2923	0.6172	Cycloguanil hydrochloride	-13.828838	-	-	-	35.66395	287.165038
NEG07601	130391.1946	156707.1566	0.265225	0.475739	0.7932	0.12717	Isoscoparine	7.3500226	-	-	-	35.6467	461.399622
NEG07605	158296.9447	108682.0292	-0.54252	0.16941	0.6304	0.21469	dUMP	-1.9668213	-	-	-	35.6315	307.174017
NEG07614	90542.42814	88727.19254	-0.02922	0.941668	0.9804	0.00175	O-Carbamoyladenylate	-11.941854	-	-	-	35.5961	389.234063

NEG07615	307545.075	286748.5716	-0.10101	0.863362	0.9547	0.09053	Aurothioglucose	8.4500358	-	-	-	35.5961	391.176137
NEG07625	1035860.088	1386953.311	0.42109	0.570104	0.8352	0.35032	Arsenocholine	11.152742	-	-	-	35.55375	164.079665
NEG07626	434070.0756	387171.1884	-0.16496	0.57583	0.8384	0.11899	p-Aminobenzamidine dihydrochloride	2.243233	-	-	-	35.54995	207.08149
NEG07632	282787.1327	230562.1446	-0.29456	0.080827	0.5377	0.28066	6-{[6,12-bis(3,4-dihydroxyphenyl)-13-hydroxy-4-oxo-3,11-	3.6491175	-	-	-	35.52135	627.534017
NEG07635	1558609.345	1919505.006	0.300475	0.686662	0.89	0.4748	[2,6-dihydroxy-3-(3,5,6,7-tetrahydroxy-4-oxo-4H-chromen-2-	18.970484	-	-	-	35.5117	413.290583
NEG07637	46437.65491	33677.01661	-0.46353	0.650297	0.8731	0.04434	({7,8,9,12,13,14,17,18,19,25,29-undecahydroxy-4,22,27-trioxo-3,23,26-	3.2433218	-	-	-	35.5116	711.495034
NEG07640	93305.48385	83098.36361	-0.16714	0.387773	0.7498	0.07542	Aluminoparaaminosalicylate calcium	-18.944097	-	-	-	35.5112	403.306264
NEG07641	5990.42095	8018.04205	0.420593	0.723705	0.9063	0.00731	4-Pyridoxate	4.5367959	-	-	-	35.50875	182.154954
NEG07644	318380.4864	427951.9106	0.426697	0.561207	0.8325	0.22498	Acetyl citrate	8.6398634	-	-	-	35.49155	231.139029
NEG07646	343484.5911	228291.3547	-0.58937	0.371879	0.7415	0.30992	D-Fructose 6-phosphate	18.626547	-	-	-	35.486	259.133369
NEG07648	172105.5768	268933.5784	0.643956	0.056361	0.4892	0.3719	1-epi-Valienol 7-phosphate	-2.5790326	-	-	-	35.481	255.139163
NEG07649	118342.3065	32008.1035	-1.88646	0.058194	0.4907	0.34455	Diclofop	-12.443247	-	-	-	35.481	326.148052
NEG07653	128455.2097	220778.8895	0.781337	0.519801	0.8139	0.25559	Shikimate 3-phosphate	-2.7299747	-	-	-	35.4732	253.12323
NEG07658	326197.2764	263069.3239	-0.3103	0.058095	0.4907	0.28815	dUDP	-11.829919	-	-	-	35.4635	387.149931
NEG07662	214061.3736	164533.0042	-0.37965	0.026816	0.367	0.25112	3-[5,7-dihydroxy-2-(4-hydroxy-3-methoxyphenyl)-4-oxo-	-2.8155838	-	-	-	35.45285	571.513111
NEG07663	1607729.004	1664664.931	0.050208	0.906381	0.9697	0.19971	[2,6-dihydroxy-3-(3,5,6,7-tetrahydroxy-4-oxo-3,4-dihydro-2H-1-benzopyran-	8.7964176	-	-	-	35.4519	415.306385
NEG07665	174072.2496	143641.8586	-0.27721	0.24125	0.6822	0.17002	Erythro-4-hydroxy-L-glutamate(1-)	11.079332	-	-	-	35.44555	161.11652
NEG07667	51632153.03	48175647.92	-0.09997	0.817276	0.9369	1.20109	2-Iodo-6-methoxyphenol	21.90451	-	-	-	35.4373	249.032
NEG07672	1104304.694	968888.708	-0.18874	0.588221	0.8421	0.18328	3,4-Dihydroxyphthalate	-20.591315	-	-	-	35.3937	197.118244
NEG07687	1161391.838	958833.5393	-0.2765	0.151075	0.6145	0.44752	4-Amino-2,6-dinitrotoluene	6.954761	-	-	-	35.33625	196.142295
NEG07707	193029.7828	154159.2835	-0.3244	0.063198	0.5039	0.20746	Geniposide pentaacetate	-24.031884	-	-	-	35.2889	597.528239
NEG07708	35781.19994	26659.06452	-0.42458	0.251704	0.6861	0.08895	2'',6''-Di-O-acetylononin	0.5551046	-	-	-	35.278	513.471009
NEG07717	304650.1532	177633.7537	-0.77825	0.03226	0.4009	0.42179	1-Deoxy-D-altro-heptulose 7-phosphate	-21.265061	-	-	-	35.2192	273.149293

NEG07718	305300.9835	98943.03085	-1.62556	0.281778	0.7016	0.35862	Alginic acid	18.52075	-	-	-	35.213	417.227969
NEG07720	132419.1959	104729.4004	-0.33845	0.277106	0.6995	0.15519	5,7-dihydroxy-2-(4-hydroxy-3-methoxyphenyl)-3,6	3.2345233	-	-	-	35.2065	623.542744
NEG07721	51909.19781	36993.26383	-0.48873	0.3833	0.7477	0.08086	Aripiprazole	11.202959	-	-	-	35.20585	447.383147
NEG07723	186404.7001	184273.2802	-0.01659	0.971003	0.9918	0.07165	Luteoskyrin	7.8634099	-	-	-	35.2043	573.485741
NEG07724	110821.5636	73344.65212	-0.59547	0.055392	0.4884	0.22125	Aluminoparaaminosalicylate calcium	18.175949	-	-	-	35.20065	403.321272
NEG07725	71828.25181	57269.47879	-0.32678	0.166146	0.6281	0.11255	5-O-(Indol-3-ylacetyl-myo-inositol) D-galactoside	-10.515104	-	-	-	35.19725	498.452671
NEG07728	707906.9882	350721.613	-1.01323	0.001378	0.1079	0.84787	2-(3,5-Dichlorophenylcarbamoyl)-1,2	-3.9072361	-	-	-	35.1905	301.144743
NEG07729	236619.1822	191615.9806	-0.30435	0.064143	0.5065	0.22726	Amarogentin	-7.6833191	-	-	-	35.1891	585.528917
NEG07734	71558.98671	46210.04329	-0.63093	0.029268	0.3835	0.1905	Cefamandole fate	8.7972704	-	-	-	35.17335	511.491832
NEG07739	133472.9509	122782.6903	-0.12044	0.807413	0.934	0.04818	6,11-dihydroxy-3-methyl-7-(3,4,5-trihydroxyphenyl)-28	-4.0729986	-	-	-	35.1582	340.285333
NEG07743	735497.0331	659977.1809	-0.1563	0.201491	0.6597	0.25764	SN38 glucuronide carboxylate form	-5.2110127	-	-	-	35.15365	584.525772
NEG07745	227906.057	225353.0286	-0.01625	0.962436	0.9888	0.04198	L 735821	15.956695	-	-	-	35.1451	463.343433
NEG07746	168431.248	162698.7253	-0.04996	0.76713	0.9214	0.03405	3-Demethylsimmondsin 2'-(Z)-ferulate	-2.7933424	-	-	-	35.1383	536.504322
NEG07748	220041.9214	239341.7843	0.121294	0.871372	0.9572	0.03193	Kaempferol 3-O-beta-D-xyloside	-15.501793	-	-	-	35.1367	417.337138
NEG07750	355910.1425	328780.4176	-0.11439	0.430978	0.77	0.11944	Loganin pentaacetate	-22.865661	-	-	-	35.13505	599.544691
NEG07752	229066.6295	110275.1362	-1.05466	0.024486	0.3595	0.45634	Pencozole	-24.847808	-	-	-	35.1334	283.169962
NEG07757	178855.1924	153318.2976	-0.22226	0.519417	0.8137	0.1387	2-Phosphoglycolate	-0.5001616	-	-	-	35.12835	155.023945
NEG07761	99514.43412	110779.3893	0.154712	0.753386	0.9162	0.06217	O-Carbamoyladenylate	22.289798	-	-	-	35.11645	389.247422
NEG07762	312058.2481	263081.8315	-0.2463	0.188958	0.6466	0.21098	N-Trimethyl-2-aminoethylphosphote	0.4955664	-	-	-	35.1115	167.144107
NEG07765	247526.4771	217225.3355	-0.18839	0.391085	0.7513	0.1404	5-Methyleriodictyol 7-[glucosyl-(1->4)-galactoside]	10.270904	-	-	-	35.1021	625.559159
NEG07766	269980.1988	231217.5074	-0.2236	0.511969	0.8097	0.19322	TEPP	1.5877882	-	-	-	35.0946	289.180884
NEG07769	374020.7293	346363.0727	-0.11083	0.470256	0.7899	0.12368	Myxochelin C	-12.37087	-	-	-	35.0861	538.519949
NEG07781	219489.4685	195000.4998	-0.17067	0.307882	0.7158	0.10349	3,4,5-trihydroxy-6-(3-{3,5,7-trihydroxy-6-[3,4,5-trihydroxy-6	23.097149	-	-	-	35.0384	611.543871

NEG07783	363882.8221	283300.1978	-0.36114	0.202986	0.6615	0.1901	Flucarbazone-sodium	-19.781286	-	-	-	35.0365	417.265449
NEG07784	653844.2228	638197.9742	-0.03494	0.907006	0.9697	0.01452	O-Phosphorylhydroxylamine	13.417581	-	-	-	35.022	112.00404
NEG07793	979844.2039	832854.0306	-0.23449	0.070243	0.5226	0.44076	Nitrofurantoin	-0.711943	-	-	-	34.95575	237.149554
NEG07794	228052.6102	211588.5106	-0.10811	0.413684	0.762	0.09235	3,4,5-trihydroxy-2-[hydroxy(3,4,5-trihydroxyoxan-2	24.091037	-	-	-	34.95575	613.560529
NEG07796	124919.1194	122771.4584	-0.02502	0.922622	0.9741	0.03049	Orotidine 5'-phosphate	-12.745897	-	-	-	34.9547	367.17883
NEG07798	104740.3888	118945.3633	0.183481	0.830338	0.9436	0.05516	Cyanidin 3-O-rutinoside	-15.21745	-	-	-	34.9547	594.509661
NEG07799	118895.0541	86697.38334	-0.45563	0.111224	0.5699	0.2129	2-Amino-1,2-bis(p-chlorophenyl)ethanol	-11.240861	-	-	-	34.93705	281.154652
NEG07800	740389.3912	849390.1298	0.198143	0.738908	0.912	0.26698	Benzoylprop-ethyl	14.123687	-	-	-	34.9337	365.236396
NEG07803	218760.225	292943.1975	0.42127	0.633229	0.8645	0.11819	Glyphosate-monoammonium	4.5178031	-	-	-	34.9092	185.097164
NEG07804	347243.4914	366579.2884	0.078178	0.885437	0.961	0.00507	Imazalil nitrate	3.5088187	-	-	-	34.907	359.186587
NEG07806	35278.18145	30384.85076	-0.21542	0.590463	0.843	0.05729	Cefixime	10.01815	-	-	-	34.8922	452.446866
NEG07812	64686.16356	308005.7431	2.251428	0.020373	0.3448	0.55041	Primisulfuron	-5.3143671	-	-	-	34.8832	453.300709
NEG07816	73188.44147	66806.4136	-0.13163	0.649454	0.8724	0.03848	6-[3-(6,7-dimethoxy-2H-1,3-benzodioxol-5-yl)oxirane-2	-0.2049125	-	-	-	34.87545	443.337632
NEG07817	4540.506635	10620.92828	1.225985	0.417243	0.7646	0.04819	Leucodelphinidin	-17.42553	-	-	-	34.8622	321.254008
NEG07819	204368.6519	173177.3431	-0.23892	0.101487	0.5624	0.19129	Lipoyl-GMP	-7.0532437	-	-	-	34.8599	550.519833
NEG07827	42695.02108	37972.76769	-0.1691	0.545691	0.8273	0.062	Amaranth	-24.202396	-	-	-	34.8482	537.50769
NEG07830	204784.3692	206598.2706	0.012723	0.94199	0.9805	0.01299	12-(3,4-dihydroxyphenyl)-13-hydroxy-6-(4-hydroxy-3-methoxyphenyl)-8	0.6601517	-	-	-	34.84	627.576138
NEG07832	596915.4581	563069.0783	-0.08421	0.581595	0.8399	0.10565	Tri-N-acetylchitotriose	-20.340245	-	-	-	34.8276	626.572758
NEG07834	267216.1623	241962.9931	-0.14322	0.349061	0.7321	0.15095	Spirolaurenone	-2.4827504	-	-	-	34.8159	298.23848
NEG07837	123601.9399	106635.1803	-0.21302	0.356226	0.7345	0.07974	3',4',5'-Trimethoxytricetin 7-[rhamnosyl-(1->6)	-22.308133	-	-	-	34.8042	651.575365
NEG07838	76836.04252	111768.497	0.540658	0.124244	0.5898	0.17077	Cyclic di-3',5'-adenylate	16.579785	-	-	-	34.8042	657.41554
NEG07840	196070.0689	180683.2661	-0.11791	0.519792	0.8139	0.10722	2-amino-4-({1-[(carboxymethyl)-C-hydroxycarbonimidoyl]-2	-20.7873	-	-	-	34.79915	566.550925
NEG07845	60738.41787	50652.00845	-0.26199	0.315099	0.7189	0.07942	3,4,5-trihydroxy-6-{[3-hydroxy-10-(3-hydroxybutanoyl)-2,2	-17.312609	-	-	-	34.7941	565.538915

NEG07846	98217.88002	64411.39592	-0.60867	0.120607	0.5836	0.16549	Baicalin	-1.13317	-	-	-	34.79055	445.353218
NEG07847	628252.2018	521454.5611	-0.2688	0.096596	0.5573	0.3228	Chlorhexidine acetate	22.176511	-	-	-	34.7857	624.557096
NEG07854	295789.4166	276820.5302	-0.09562	0.591274	0.8436	0.08356	(6-carboxy-3,4,5-trihydroxyoxan-2-yl)({4-[6,11-dihydroxy-7-(4-	10.360338	-	-	-	34.76755	622.541184
NEG07859	157675.4098	156443.2416	-0.01132	0.946992	0.9827	0.01402	Robecoxib	10.160006	-	-	-	34.75145	326.269549
NEG07861	453417.9835	615540.4195	0.441012	0.339074	0.7277	0.26991	4-(N-Maleimido)phenyltrimethylammonium iodide	14.886029	-	-	-	34.7451	357.172955
NEG07862	311671.3139	262607.9509	-0.24711	0.183213	0.642	0.20592	Malvidin 3-(6-coumaroylglucoside)	-0.0366504	-	-	-	34.74505	638.5728
NEG07863	129438.3188	91285.94976	-0.5038	0.053606	0.4853	0.21281	Plantamajoside	-6.116974	-	-	-	34.74505	639.575305
NEG07864	45512.4731	54404.86717	0.257474	0.593216	0.8447	0.05084	Secogalioside	-13.267089	-	-	-	34.745	419.352446
NEG07866	130332.0012	541715.0684	2.055343	0.285866	0.7027	0.64405	3,4-Hexahydroxydiphenoylarabinose	20.637078	-	-	-	34.71915	451.324558
NEG07867	56909.42723	44380.98748	-0.35873	0.166007	0.6281	0.11104	(Z)-Resveratrol 3,4'-diglucoside	11.042928	-	-	-	34.7191	551.523325
NEG07876	106502.4251	71140.16454	-0.58215	0.100932	0.5624	0.2039	2-{[hydroxy({3,4,8,9,10-pentahydroxy-6-oxo-6H-benzo[c]chromen-1-	-8.1812321	-	-	-	34.69055	376.250637
NEG07879	71715.68197	65657.56248	-0.12733	0.363662	0.7394	0.07219	Osthenol-7-O-beta-D-gentiobioside	10.529685	-	-	-	34.6525	553.538963
NEG07882	89523.97425	82840.41345	-0.11194	0.752687	0.9157	0.04056	Deoxyribose triphosphate	-0.7750401	-	-	-	34.644	373.062633
NEG07888	126267.9537	122730.4356	-0.041	0.754207	0.9166	0.03586	Riccionidin A	7.2840835	-	-	-	34.6226	284.223201
NEG07889	300791.4072	281583.6289	-0.0952	0.827711	0.9421	0.13555	Atheroline	24.124483	-	-	-	34.6226	336.326961
NEG07891	134511.4795	137774.9104	0.034584	0.972944	0.9921	0.05985	Cartormin	-24.586632	-	-	-	34.61805	574.496673
NEG07896	96837.88899	84792.30266	-0.19164	0.367312	0.7403	0.10469	Streptomycin	-0.1693363	-	-	-	34.59125	580.566725
NEG07897	55063.34225	50272.55942	-0.13132	0.473602	0.792	0.04361	Rhamnazin 3-rutinoside	-7.7880973	-	-	-	34.58475	637.55845
NEG07905	58863.56621	54696.3131	-0.10593	0.823731	0.9405	0.06125	[2,6-dihydroxy-3-(3-phenylpropanoyl)phenyl]oxidanesulfonic acid	22.228779	-	-	-	34.5616	337.330244
NEG07910	95228.74809	92432.35645	-0.043	0.8515	0.9515	0.03249	Haemocorin	-7.5773933	-	-	-	34.54485	641.591854
NEG07911	62570.23836	55858.36269	-0.1637	0.532941	0.8215	0.06718	Citrusin B	-9.0106332	-	-	-	34.5385	567.5545
NEG07914	58970.63114	152188.4433	1.36779	0.415295	0.7629	0.19192	Tomentin	-4.0988537	-	-	-	34.5337	345.279604
NEG07918	518770.4312	238700.3194	-1.1199	0.323371	0.7192	0.40185	4-(N-Maleimido)benzyltrimethylammonium iodide	-20.930331	-	-	-	34.5154	371.186433

NEG07923	52225.93305	53150.31172	0.025312	0.941559	0.9804	0.00517	Diosmin	8.3753184	-	-	-	34.5119	607.54252
NEG07926	201645.5061	217243.9245	0.107495	0.618571	0.8572	0.07769	{4-[(2,4-dihydroxy-3-oxo-<br>6-{[3,4,5-trihydroxy-6-<br>(hydroxymethyl)oxan-2	7.2294668	-	-	-	34.50275	529.446558
NEG07927	121236.2948	82379.68867	-0.55746	0.313117	0.7189	0.18756	Procymidone	-11.863822	-	-	-	34.4901	283.127252
NEG07929	171812.5283	156679.1194	-0.13302	0.314266	0.7189	0.12045	4-Hydroxylamino-2,6-<br>dinitrotoluene	-14.537491	-	-	-	34.4678	212.137225
NEG07931	155467.9431	170896.5633	0.136506	0.624265	0.8594	0.08894	Malonyldaidzin	-2.8632811	-	-	-	34.4678	501.415585
NEG07932	655489.552	580060.3314	-0.17637	0.124071	0.5898	0.25461	Pelargonidin 3-O-<br>3'',6'&<br>apos;-O	-14.396322	-	-	-	34.4678	604.461807
NEG07937	62436.46625	50737.7404	-0.29933	0.370349	0.7409	0.0901	Bromocriptine	6.1885544	-	-	-	34.4548	653.591274
NEG07940	3068534.917	4149212.168	0.435287	0.51063	0.8083	0.76776	Niflumic acid	5.4939878	-	-	-	34.4523	281.212274
NEG07945	322267.5455	251094.435	-0.36003	0.458694	0.7822	0.1886	5-Methyl-THF	-0.5202307	-	-	-	34.44675	502.419462
NEG07949	332772.7801	2480409.745	2.897969	0.353561	0.7327	0.89351	Anthragallol	-24.692474	-	-	-	34.4413	255.196697
NEG07954	157094.581	167690.6814	0.094169	0.828398	0.9427	0.0034	Cyanidin 3-O-<br>3'',6'&<br>apos;-O	15.682648	-	-	-	34.43585	620.47967
NEG07955	331654.4651	352911.9645	0.089627	0.732313	0.9091	0.06136	dTDP-4-dimethylamino-<br>4,6-dideoxy-5-C-methyl-<br>L-mannose	22.860297	-	-	-	34.4335	588.430698
NEG07958	463817.6268	256174.3611	-0.85643	0.099801	0.5613	0.53387	Azacozole	-6.326812	-	-	-	34.4257	299.131424
NEG07965	144697.9697	123974.9963	-0.223	0.463195	0.7842	0.10794	Molybdopterin guanine<br>dinucleotide	11.557314	-	-	-	34.4257	737.542559
NEG07967	655948.5084	861341.6175	0.393003	0.326077	0.7202	0.42418	TG(22:5(4Z,7Z,10Z,13Z,1<br>6Z)/22:6(4Z,7Z,10Z,13Z,1<br>6Z,19Z)/o-18:0)	-20.854291	-	-	-	34.40775	966.530546
NEG07969	102131.5467	207374.295	1.021809	0.415255	0.7629	0.20566	1,2,3,4-Tetrahydro-<br>alpha,7-dihydroxy-beta-<br>(hydroxymethyl)-9	-4.9406929	-	-	-	34.4039	347.295103
NEG07970	1133612.552	56050.80129	-4.33805	0.322632	0.7192	0.76788	Norbadione A	-7.484108	-	-	-	34.4039	677.496045
NEG07972	46755.49112	39610.2862	-0.23926	0.602675	0.8493	0.04694	2-amino-4-({1-<br>[(carboxymethyl)-C-<br>hydroxycarbonimidoyl]-2	24.064011	-	-	-	34.39445	592.567007
NEG07974	97014.95451	285119.7122	1.555289	0.347396	0.7313	0.42475	2,6-Dichlorobenzamide	16.70234	-	-	-	34.38355	189.022597
NEG07977	103295.297	115888.1814	0.165959	0.550517	0.8293	0.06217	4,4',6,6'-<br>Tetranitro-2,2'-<br>azoxytoluene	19.403675	-	-	-	34.3796	405.264606
NEG07978	100578.6055	66901.63534	-0.58821	0.126301	0.5923	0.20719	Oleuropein	-4.5358832	-	-	-	34.3796	539.504072
NEG07982	51551.03621	50658.19015	-0.02521	0.90767	0.9697	0.01834	Diflufenican	-20.798623	-	-	-	34.3632	393.279423
NEG07988	206790.5976	224973.8261	0.121587	0.709964	0.9015	0.0311	GDP-4-acetamido-4,6-<br>dideoxy-alpha-D-mannose	-2.8614434	-	-	-	34.33655	629.38452

NEG07990	748547.0427	925149.6252	0.305594	0.298895	0.7148	0.35646	Selenium Sulfide	17.797918	-	-	-	34.3325	110.0247
NEG07996	630850.3443	844219.8526	0.420321	0.431695	0.77	0.37725	2,4-Dihydroxypteridine	-8.7389712	-	-	-	34.3095	163.112889
NEG08002	252886.5482	125930.481	-1.00586	0.108274	0.5681	0.36129	MC-5127	21.511958	-	-	-	34.2926	355.157885
NEG08005	102671.5819	162699.9133	0.664177	0.180866	0.6407	0.24336	TG(22:5(4Z,7Z,10Z,13Z,16Z)/o-18:0/22:5(4Z,7Z,10Z,13Z [illegible]	-5.8983318	-	-	-	34.2887	968.537005
NEG08006	113186.8486	113625.4892	0.00558	0.976978	0.9934	0.00298	3,4,5-trihydroxy-6-({12-hydroxy-13,14-dimethoxy-9-oxo-8,17 [illegible]	6.9029501	-	-	-	34.28865	503.396205
NEG08014	616726.3264	37510166.4	5.926507	0.013389	0.2989	8.3516	5-Fluorodeoxyuridine monophosphate	10.851988	-	-	-	34.25405	325.168563
NEG08016	123406.3467	91973.70175	-0.42412	0.091522	0.5568	0.19734	Aluminum acetoacetate	-13.446244	-	-	-	34.2338	329.211883
NEG08025	79014.29894	91731.17276	0.215298	0.544033	0.827	0.06071	11-O-Demethylpradinone I	17.234573	-	-	-	34.213	463.378527
NEG08027	7374.933072	5504.505464	-0.42202	0.413484	0.762	0.04104	Dihydro-ME	-10.585354	-	-	-	34.186	397.351307
NEG08029	2724697.294	2744894.056	0.010655	0.959922	0.9877	0.05613	6-{[6-(3,4-dihydroxy-6-methyl-5-oxooxan-2-yl)-2-(3,4-dihydroxyphenyl)-5 [illegible]	-0.1590171	-	-	-	34.18175	605.481627
NEG08031	38264.74641	46673.27877	0.286581	0.61805	0.8569	0.06151	TG(15:0/20:5(5Z,8Z,11Z,14Z,17Z)/24:1(15Z))	-21.281236	-	-	-	34.1715	948.512516
NEG08033	155515.8869	125976.8827	-0.3039	0.159756	0.6215	0.18102	p-N,N-(Dimethylamino)phenyldiazonium fluoroborate	24.375244	-	-	-	34.1682	233.988151
NEG08040	251297.1318	106722.0026	-1.23554	0.260481	0.6904	0.31394	1'-hydroxytriazolam	24.44842	-	-	-	34.13995	358.210505
NEG08041	78975.27736	65899.01005	-0.26114	0.189395	0.6466	0.12413	Hesperidin	7.3390628	-	-	-	34.13995	609.557804
NEG08045	456062.5262	473723.9557	0.054815	0.794834	0.9292	0.05467	m-Trigallic acid	12.520016	-	-	-	34.1317	473.326762
NEG08049	674782.674	606199.8217	-0.15463	0.482984	0.7977	0.24038	Cisplatin	-0.0213619	-	-	-	34.1253	299.043717
NEG08055	49097.29259	50739.97941	0.047479	0.861728	0.9541	0.00509	alpha,alpha'-Trehalose 6-phosphate	13.030838	-	-	-	34.1192	421.274626
NEG08057	103819.8247	82631.29516	-0.32932	0.577211	0.8394	0.1522	Sedoheptulose 7-phosphate	18.080355	-	-	-	34.1132	289.15977
NEG08060	200419.95	239044.2302	0.254251	0.055074	0.4884	0.20856	Zoledronate	4.8999786	-	-	-	34.09725	271.083657
NEG08061	444567.3456	531716.4969	0.258255	0.498174	0.8016	0.17147	Gardenoside	-2.5918636	-	-	-	34.09725	403.357575
NEG08069	929828.07	919172.1955	-0.01663	0.940583	0.9803	0.08156	[Nitrilotris(methylene)]trisphosphonic acid	11.469168	-	-	-	34.0758	298.046153
NEG08071	180747.5043	213086.2038	0.237461	0.586154	0.8419	0.08597	7a-Hydroxy-O-carbamoyl-deacetylcephalosporin C	-21.887211	-	-	-	34.07145	431.388959
NEG08077	127248.0843	81353.32105	-0.64537	0.062091	0.5022	0.21675	6-Mercaptopurine ribonucleoside 5'-diphosphate	2.5991131	-	-	-	34.0573	443.245378

NEG08082	1007373.137	192893.203	-2.38472	0.331399	0.7232	1.00383	Hydroxyflutamide	-21.471769	-	-	-	34.0039	291.197749
NEG08085	89907.68971	90884.74018	0.015594	0.971243	0.9918	0.03782	2-O-Galloyl-1,4-galactarolactone	20.684789	-	-	-	33.981	343.227644
NEG08091	207323.425	199261.464	-0.05722	0.690628	0.8924	0.06206	Pyriprole	-9.9896632	-	-	-	33.9455	493.256286
NEG08093	73358.82566	96199.77174	0.391063	0.145429	0.6127	0.15205	1-O,6-O-Digalloyl-beta-D-glucose	-19.711065	-	-	-	33.9386	483.347576
NEG08096	172659.0809	98328.30614	-0.81225	0.101043	0.5624	0.30701	3,4,5-trihydroxy-6-(2,4,6-trihydroxybenzoyloxy)oxane-2-carboxylic acid	20.89038	-	-	-	33.91495	345.243957
NEG08098	93177.75423	421599.2084	2.177815	0.000645	0.0947	0.82635	Dichlofenthion	17.447602	-	-	-	33.90575	314.151422
NEG08102	43480.19518	34980.73813	-0.3138	0.11485	0.5747	0.09934	Forsythiaside	-11.358735	-	-	-	33.8749	623.572729
NEG08105	190358.1347	217602.4105	0.192978	0.283235	0.7021	0.12091	Chondroitin sulfate	-9.343933	-	-	-	33.86595	462.357394
NEG08109	33025.59582	32753.6618	-0.01193	0.955136	0.9856	0.02313	Enilcozole	-24.499014	-	-	-	33.84715	296.165243
NEG08119	37465.12028	42753.33732	0.190489	0.598332	0.8466	0.03034	Prosulfuron	12.38524	-	-	-	33.7601	418.376818
NEG08123	58961.47906	57868.5731	-0.02699	0.896501	0.9654	0.02189	Quercetin 3-O-glucoside	-16.156512	-	-	-	33.7442	463.361521
NEG08127	238348.8082	139571.5798	-0.77207	0.221685	0.6692	0.30545	(3E)-4-(2-Carboxyphenyl)-2-oxobut-3-enoate	20.312551	-	-	-	33.71925	219.175396
NEG08129	926205.5003	398666.102	-1.21615	0.26043	0.6904	0.60015	2-Galloyl-1,4-galactarolactone methyl ester	-10.955506	-	-	-	33.716	357.243199
NEG08130	198466.7653	193982.7779	-0.03297	0.924739	0.9754	0.05129	Biochanin A-beta-D-glucoside	17.171903	-	-	-	33.716	445.404489
NEG08131	89439.41488	84970.02251	-0.07396	0.772394	0.9224	0.02729	7-Epiloganin tetraacetate	-12.745381	-	-	-	33.6845	557.514705
NEG08141	100602.6558	82974.23423	-0.27793	0.137468	0.6023	0.14823	2-Iodophenol methyl ether	1.18475	-	-	-	33.5369	233.027401
NEG08142	492420.3006	271036.56	-0.8614	0.088291	0.5472	0.5712	thymidine 3'-monophosphate	18.494584	-	-	-	33.5369	319.193645
NEG08144	749293.3908	255905.7608	-1.54992	0.41366	0.762	0.75512	2-Protocatechoylphloroglucinolcarboxylate	-8.3658178	-	-	-	33.531	305.214562
NEG08146	207611.6265	346502.1675	0.738977	0.174024	0.6335	0.39367	TG(15:0/20:2n6/24:1(15Z))	-24.779154	-	-	-	33.49585	954.557045
NEG08152	1388149.383	2184649.454	0.654239	0.189093	0.6466	0.92906	TG(22:2(13Z,16Z)/15:0/22:2(13Z,16Z))	10.212098	-	-	-	33.45865	952.551561
NEG08156	255282.4585	248440.4645	-0.03919	0.819166	0.9375	0.05295	Cefuroxime sodium	19.076129	-	-	-	33.45485	445.368338
NEG08159	200828.7858	200146.4238	-0.00491	0.992375	0.9983	0.00167	2-Iodophenol	-16.934549	-	-	-	33.4377	218.996798
NEG08161	56302.48989	44865.32409	-0.3276	0.084116	0.5411	0.13116	5'-Deoxy-5'-fluoroadenosine	10.3208	-	-	-	33.4265	268.227902

NEG08162	162853.3359	128820.8815	-0.33821	0.245685	0.6842	0.18088	6''-(4-Carboxy-3-hydroxy-3-methylbutanoyl)hyperin	-9.7063967	-	-	-	33.4156	607.488417
NEG08171	980054.3732	858706.6626	-0.1907	0.094234	0.5568	0.42967	7''-O-Phosphohygromycin	-12.252694	-	-	-	33.3749	606.48528
NEG08174	169410.4655	161664.867	-0.06752	0.735465	0.9109	0.04079	Myricatin	-0.7621566	-	-	-	33.36535	551.410302
NEG08177	198281.8892	192164.102	-0.04521	0.816012	0.9364	0.00929	Nitrosylhaem	15.26976	-	-	-	33.3567	645.495595
NEG08179	372108.7359	339991.6937	-0.13022	0.636659	0.8666	0.09526	Isosorbide dinitrate	19.161764	-	-	-	33.3163	235.133548
NEG08182	1911361.804	1837387.317	-0.05695	0.731261	0.9083	0.22956	6-{[6-(3,4-dihydroxy-6-methyl-5-oxooxan-2-yl)-5-hydroxy-2-(4-hydroxy-3-	-17.154674	-	-	-	33.3113	619.498079
NEG08185	445014.0704	512365.0832	0.203321	0.623601	0.8594	0.13883	L-Histidinol phosphate	13.25333	-	-	-	33.2851	220.146554
NEG08186	46584.03675	29771.33355	-0.64591	0.066684	0.5151	0.13784	Taxiphyllin	-15.994255	-	-	-	33.2828	310.275045
NEG08191	290271.5274	568626.3387	0.970078	0.146134	0.6127	0.46288	Dopaxanthin	13.431026	-	-	-	33.2658	389.342066
NEG08192	49510.58944	86552.69414	0.805842	0.15894	0.6214	0.20371	3,28-Dihexadecanoyl 20(29)-lupene-2a,3b,28-triol	7.6064951	-	-	-	33.2658	934.53364
NEG08206	36100.1376	63639.81464	0.817925	0.283371	0.7021	0.15924	TG(14:0/20:3(5Z,8Z,11Z)/24:1(15Z))	-4.6753799	-	-	-	33.15765	938.533331
NEG08210	192443.5132	163515.208	-0.23501	0.128939	0.5925	0.18144	D-Glucosamine 6-phosphate	-4.5966957	-	-	-	33.1235	258.142532
NEG08216	85713.93606	78559.36924	-0.12575	0.536247	0.8236	0.07366	3,4,5-trihydroxy-6-{[2-oxo-3-(2,3,4,5-tetrahydroxyphenyl)propa	-22.163898	-	-	-	33.1061	403.263763
NEG08223	168595.2208	108063.0079	-0.64169	0.017454	0.3284	0.28642	8-C-Ascorbylepigallocatechin 3-gallate	9.4726956	-	-	-	33.08995	631.478715
NEG08224	87205.64	99709.79023	0.193314	0.616093	0.8567	0.09886	Chondroitin 4-sulfate	-12.927055	-	-	-	33.08745	474.365578
NEG08232	260057.4962	247474.0575	-0.07155	0.919302	0.9738	0.03704	4-(4-Deoxy-beta-D-gluc-4-enuronosyl)-D-galacturote	-24.236422	-	-	-	33.0684	351.232386
NEG08239	368431.9315	400192.071	0.119294	0.743388	0.9124	0.14846	Chicoric acid	-1.2030289	-	-	-	33.0185	473.363253
NEG08245	400170.0154	454589.9532	0.183953	0.7804	0.9255	0.10973	Fosaprepitant	-16.069612	-	-	-	32.9843	613.38945
NEG08248	1547099.185	1371500.805	-0.17381	0.564835	0.8334	0.34127	L-Selenocysteine	0.6226419	-	-	-	32.97105	167.046028
NEG08249	55982.29283	46750.33403	-0.25999	0.257271	0.6897	0.11084	2'',4'',6''-Triacetylglycitin	-21.53096	-	-	-	32.9707	571.494497
NEG08253	102850.3856	83441.34832	-0.30171	0.452152	0.7814	0.13673	3,4,5-trihydroxy-6-[(3,4,5-trihydroxy-6-{[2-(2-hydroxypropan-2-yl)-7-	9.6910334	-	-	-	32.9262	599.524543
NEG08256	575588.0567	572790.0019	-0.00703	0.951846	0.9842	0.0288	6-({13,14-dihydroxy-9-oxo-8,17-dioxatetracyclo[8.7.0.0	16.89517	-	-	-	32.89195	459.347501
NEG08266	166190.2639	157595.4775	-0.07661	0.7289	0.9081	0.04217	3,4,5-trihydroxy-6-[3-(3,5,7-trihydroxy-5H-chromen-2-	23.796405	-	-	-	32.7885	446.375369

NEG08273	58417.10805	40317.12819	-0.535	0.005576	0.2114	0.17677	Cefonicid sodium	-21.801417	-	-	-	32.7829	585.509536
NEG08275	380583.2252	346967.5828	-0.13341	0.468608	0.7893	0.12446	Quercetin 3,3'-bissulfate	19.266626	-	-	-	32.7712	461.363732
NEG08279	370605.5875	375872.2708	0.020358	0.888641	0.9626	0.03168	{4-[2,3-dioxo-3-(2,4,6-trihydroxy-3-methoxyphenyl)propyl]-2	-0.6320806	-	-	-	32.7394	443.352443
NEG08282	75019.66262	49535.92743	-0.59879	0.065494	0.5111	0.17741	O-Hexanoyl-adnosine monophosphate	-1.9341627	-	-	-	32.7001	444.356062
NEG08289	400945.8468	86163.25582	-2.21826	0.311362	0.7182	0.62666	Phenyl 5-phospho-alpha-D-ribofuranoside	-9.2229866	-	-	-	32.5875	305.195699
NEG08297	306052.9218	336374.5867	0.136288	0.617983	0.8569	0.13483	3,4,5-trihydroxy-6-({15-hydroxy-11,17,18-trimethoxy-13-oxo-6,8,20	-4.0020978	-	-	-	32.56145	575.453416
NEG08309	66830.82784	45334.00392	-0.55992	0.041202	0.44	0.15769	Leiocarposide	-2.8084358	-	-	-	32.449	613.540297
NEG08311	837703.9436	500890.5129	-0.74195	0.554376	0.83	0.32524	S-(1,2-Dichlorovinyl)glutathione	11.479833	-	-	-	32.4422	401.248241
NEG08314	33231.79249	45831.60172	0.463779	0.3215	0.7192	0.10178	TG(14:0/24:1(15Z)/o-18:0)	2.0795839	-	-	-	32.4249	902.550602
NEG08317	69600.03238	83663.2287	0.265506	0.425157	0.7674	0.08331	N-Desmethyl vandetanib	20.692168	-	-	-	32.3552	460.329269
NEG08318	694003.9661	584613.1656	-0.24746	0.047586	0.4627	0.37525	Manniflavanone	-19.696646	-	-	-	32.3518	589.468993
NEG08321	115227.7133	85988.48599	-0.42227	0.345699	0.7309	0.18196	Nitrosylhaem	-20.495322	-	-	-	32.3037	645.472473
NEG08322	436349.2348	374746.7272	-0.21957	0.40729	0.7587	0.25781	2-[(2R,5Z)-2-Carboxy-4-methylthiazol-5(2H)-ylidene]ethyl phosphate	-17.45104	-	-	-	32.3023	266.184261
NEG08335	2419380.728	1195155.358	-1.01744	0.082565	0.5402	1.2407	Iodide	21.594617	-	-	-	32.1486	125.899964
NEG08340	873663.4509	872056.4078	-0.00266	0.981976	0.9949	0.06079	p-Chloromercuribenzoate	11.357633	-	-	-	32.1142	356.15318
NEG08341	165763.5548	173272.6224	0.063917	0.834456	0.9453	0.03916	Luteolin 4'-glucoside 7-galacturonide	-8.3420769	-	-	-	32.10285	623.488514
NEG08349	549755.676	315615.4019	-0.80062	0.306221	0.7152	0.43706	Chlorambucil	-18.073984	-	-	-	32.0485	303.199425
NEG08350	271623.7742	319807.6068	0.235594	0.544736	0.8272	0.16221	Quercetin 3-O-beta-D-glucosyl-(1->2)-beta-D-glucoside	-8.5882321	-	-	-	32.0485	625.504243
NEG08355	50557.18948	50498.13795	-0.00169	0.997205	0.999	0.00924	Cyanidin 3-O-(2-O-beta-D-glucuronosyl)-beta-D-glucoside	-15.357723	-	-	-	32.00175	624.492117
NEG08356	625514.2509	647703.7217	0.050291	0.892681	0.9637	0.14648	Chrysoeriol 4',7-diglucuronide	24.857563	-	-	-	31.9972	651.520043
NEG08357	94292.456	105552.0462	0.16274	0.708967	0.9015	0.06258	Delphinidin 3-O-sophoroside	-14.977872	-	-	-	31.994	626.508124
NEG08358	115255.2586	128637.1104	0.158474	0.621208	0.8589	0.11313	Bisnorbadioquinone A	20.619052	-	-	-	31.994	649.504436
NEG08359	105935.5035	115816.1672	0.12865	0.702725	0.8994	0.07209	Luteolin	-23.678039	-	-	-	31.98785	285.222246

NEG08361	141905.4179	139695.4727	-0.02264	0.955945	0.9861	0.05209	dTDP-3-methyl-4-oxo-2,6-dideoxy-L-allose	-2.6577265	-	-	-	31.9366	543.332177
NEG08363	347416.9531	383180.7249	0.141357	0.789042	0.9279	0.13872	Amlaic acid	24.65091	-	-	-	31.9119	651.476907
NEG08364	245828.905	273973.3683	0.156381	0.693354	0.8938	0.15415	Ferrioxamine	7.2740083	-	-	-	31.9119	652.523477
NEG08381	359264.3478	394132.2725	0.133634	0.740018	0.912	0.12643	3,4,5-trihydroxy-6-({7-methoxy-4-oxo-6-[3,4,5-trihydroxy-6	19.632237	-	-	-	31.78685	653.535573
NEG08382	214776.0056	211110.3801	-0.02484	0.957091	0.9865	0.03949	5-[(6-{[(2-carboxyacetyl)oxy]methyl}-3,4,5-trihydroxyoxan-2	-19.127401	-	-	-	31.7851	680.554687
NEG08387	354704.2867	383611.4838	0.113029	0.878451	0.9584	0.13101	11-[(6-carboxy-3,4,5-trihydroxyoxan-2-yl)oxy]-3-methyl-6-{[3,4,5	-18.67252	-	-	-	31.7788	678.539034
NEG08389	554792.4142	518223.1262	-0.09837	0.826797	0.9418	0.00558	6-{4-[(1E)-3-[(6-{[3,4-dihydroxy-2,5-bis(hydroxymethyl)oxolan	-9.4690874	-	-	-	31.77825	679.551279
NEG08394	39440.23056	21658.59431	-0.86473	0.272953	0.6987	0.09267	8-Hydroxyhesperetin 7-[6-acetylglucosyl-(1->2)-glucoside]	-18.661716	-	-	-	31.7314	681.560185
NEG08396	72541.82549	49949.73316	-0.53834	0.062174	0.5022	0.18097	Reduced coenzyme F420	-13.515811	-	-	-	31.6943	774.59074
NEG08410	64486.28711	53567.87152	-0.26762	0.483282	0.7978	0.09696	alpha-Amyrin tetratriacontanoate	4.7521778	-	-	-	31.4555	916.601684
NEG08419	214253.396	171510.4381	-0.32102	0.259811	0.6904	0.21122	Uracil mustard	6.4565337	-	-	-	31.29475	251.092151
NEG08430	137305.6285	35672615.21	8.021282	0.000275	0.0947	8.79263	Diclofop	-3.7615781	-	-	-	31.10565	326.150893
NEG08435	460118.6182	390325.645	-0.23733	0.306001	0.7152	0.29877	Bromobenzene	4.5386415	-	-	-	31.0335	156.001336
NEG08443	39396.91251	26723.78582	-0.55996	0.046137	0.4551	0.13871	2'-C-Methylmyricetin 3-rhamnoside 5'-	1.5508282	-	-	-	30.8477	629.500801
NEG08447	106385.892	81717.3685	-0.38059	0.301773	0.7148	0.13614	2,6-Dichloro-4'-biphenylol	7.3484301	-	-	-	30.7824	238.09178
NEG08455	271848.7606	133483.8488	-1.02614	0.057284	0.4899	0.4385	2-C-Methyl-D-erythritol 2,4-cyclodiphosphate	23.210053	-	-	-	30.7345	277.090078
NEG08456	287403.4844	274651.3214	-0.06548	0.718692	0.9041	0.05906	1,7-diphospho-1-epi-valienol	-9.1579437	-	-	-	30.7009	335.116645
NEG08469	180964.5464	411987.0674	1.186892	0.010535	0.2728	0.6172	DTP	-18.379877	-	-	-	30.4355	284.113483
NEG08470	1088488.619	3431107.04	1.656348	0.00076	0.0947	2.17292	Diclofec	-15.017715	-	-	-	30.3857	295.136876
NEG08471	396628.8448	306306.1872	-0.37282	0.108401	0.5681	0.34813	Coenzyme F420-1	-20.666204	-	-	-	30.36655	643.458004
NEG08474	92203.93285	6292.961789	-3.87302	0.151821	0.6145	0.35501	dTDP-4-oxo-2,6-dideoxy-L-allose	-4.7969242	-	-	-	30.31375	529.30448
NEG08478	225103.1311	385160.2151	0.774873	0.002971	0.1506	0.53104	5-Phosphoribosyl-4-carboxy-5-aminoimidazole	4.5095426	-	-	-	30.2483	335.168239
NEG08482	209984.7169	468034.4569	1.15633	0.005202	0.2034	0.67162	2-(Formamido)-N1-(5-phospho-D-ribosyl)acetamidine	-3.0732258	-	-	-	30.1218	284.140347

NEG08487	25869596.4	2173450.52	-3.5732	0.105864	0.5677	5.87721	DCI	-18.196865	-	-	-	29.98505	247.137108
NEG08490	230726.1931	207878.0461	-0.15044	0.725481	0.9063	0.07466	Cyanidin 3-(6''-dioxalylglucoside)	24.601733	-	-	-	29.9735	592.432323
NEG08500	137836.31	143738.7877	0.060493	0.787478	0.9273	0.00203	Bisphenol A bis(chloroformate)	-5.6872326	-	-	-	29.7982	352.187415
NEG08504	132259.9531	82403.6382	-0.6826	0.320655	0.7192	0.15428	IDP	0.548069	-	-	-	29.7799	427.178858
NEG08505	67305.27714	46317.55352	-0.53916	0.095209	0.5568	0.16604	Saprisartan	-2.7213055	-	-	-	29.7755	610.42206
NEG08509	1409898.05	3431765.186	1.28336	0.003304	0.1574	1.93783	Procymidone	22.601561	-	-	-	29.61945	283.137045
NEG08510	497880.5767	1454441.65	1.546594	0.000643	0.0947	1.38435	Chlorobenzilate	-24.312763	-	-	-	29.61945	324.171417
NEG08513	80717.96644	52100.45578	-0.63159	0.070296	0.5226	0.19834	3-Oxopropionyl-CoA	-21.662398	-	-	-	29.50045	836.555479
NEG08516	2651835.704	7621567.264	1.523096	0.000684	0.0947	3.16787	5-O-(1-Carboxyvinyl)-3-phosphoshikimate	-7.9448952	-	-	-	29.4716	323.168148
NEG08518	65663.62103	233313.4205	1.829103	0.000887	0.0947	0.56107	N-Acetyl-D-glucosamine 1,6-bisphosphate	-3.3971422	-	-	-	29.4496	380.159029
NEG08521	1088938.456	1054508.498	-0.04635	0.663939	0.8791	0.12364	Kamycin A 3'-phosphate	-16.597102	-	-	-	29.4477	563.461855
NEG08523	223747.5995	300473.0525	0.425363	0.709721	0.9015	0.07261	Hypericin	20.533474	-	-	-	29.4423	503.446281
NEG08524	57890.99906	52164.54593	-0.15027	0.874685	0.9576	0.04101	Medicarpin 3-O-glucoside-6'-malote	4.5012982	-	-	-	29.4423	517.461757
NEG08526	603775.2078	547228.2583	-0.14187	0.239915	0.6822	0.23344	Cyanidin 3-(6''-succinyl-glucoside)	-13.941157	-	-	-	29.4386	548.442663
NEG08527	1934587.18	1746399.213	-0.14764	0.196338	0.6557	0.47215	Chrysoeriol 7-O-(6''-malonyl-glucoside)	-5.3099589	-	-	-	29.4282	547.439511
NEG08530	133618.9693	40339.96533	-1.72784	0.002876	0.1473	0.41455	Cyhexatin	-22.313022	-	-	-	29.35605	384.156129
NEG08533	46097.53491	76397.99297	0.728845	0.656116	0.8753	0.05709	Chlorhexidine	20.915809	-	-	-	29.312	504.449895
NEG08538	200035.5532	152815.6971	-0.38846	0.147434	0.6145	0.2353	N-Acetyl-N6,O-didemethylpuromycin-5'-phosphate	-18.657691	-	-	-	29.26915	564.455173
NEG08545	162852.4077	135722.3643	-0.26291	0.460432	0.7836	0.1722	3-oxobrimonidine	-17.397926	-	-	-	29.1512	309.137327
NEG08547	654245.374	424526.4134	-0.62398	0.241804	0.6822	0.47915	Methazole	8.4150785	-	-	-	29.1512	260.05642
NEG08549	29239.49846	88731.37677	1.601526	0.001159	0.0993	0.31723	Sedoheptulose 1,7-bisphosphate	-20.045552	-	-	-	29.1289	369.127004
NEG08550	99284.31204	112623.6139	0.181872	0.590324	0.8429	0.10936	Quinacridone	16.204936	-	-	-	29.1248	311.319285
NEG08557	86380.16151	47203.53253	-0.87181	0.015058	0.3098	0.26707	CMP-N-glycoloyl-beta-neuraminate(2-)	-17.108149	-	-	-	29.1145	627.419972

NEG08559	325408.723	1211059.246	1.895945	0.000572	0.0947	1.33906		1-(5-Phospho-D-ribosyl)-5-amino-4-imidazolecarboxylate	-3.0803021	-	-	-	29.08515	338.187579
NEG08560	1895793.333	6165263.165	1.701361	0.001082	0.0968	2.91136		Azafenidin	7.4656553	-	-	-	29.0596	337.183848
NEG08581	54712.46875	16407.15014	-1.73754	0.023717	0.3593	0.24935		3-Iodo-L-tyrosine	24.439747	-	-	-	28.8051	306.085328
NEG08584	2431169.497	10568999.76	2.120116	0.000606	0.0947	4.07203		Eudistomin G	5.0792024	-	-	-	28.8008	313.174219
NEG08610	167829.2663	192234.004	0.195869	0.703154	0.8994	0.00751		3'-(2'',3''	-19.836395	-	-	-	28.492	633.475937
NEG08613	175858.3844	838499.6539	2.253396	0.000754	0.0947	1.15062		3',5'-Cyclic AMP	-18.404981	-	-	-	28.4818	328.192564
NEG08617	56486.89932	386033.076	2.772736	0.000695	0.0947	0.81011		3-hydroxyguanfacine glucuronide	-6.0288984	-	-	-	28.4716	453.205985
NEG08618	2799449.052	11094864.78	1.986677	0.000875	0.0947	4.08933		Fluorodifen	-9.8272009	-	-	-	28.4705	327.189798
NEG08625	69312.97609	410086.8789	2.564732	0.000972	0.0947	0.83922		3',5'-Cyclic IMP	8.4013223	-	-	-	28.4694	329.186197
NEG08626	141986983.8	561944944.5	1.98467	0.000815	0.0947	29.0832		2,4-Dioxotetrahydropyrimidine D-ribonucleotide	-18.872593	-	-	-	28.4689	325.183767
NEG08628	5746092.945	23158626.25	2.010896	0.000888	0.0947	5.91478		Etacozole	-21.274559	-	-	-	28.4689	327.179541
NEG08629	1520319.774	1561040.559	0.038133	0.771011	0.9221	0.00965		Manniflavanone	9.4740562	-	-	-	28.4689	589.486218
NEG08631	666573.7794	560695.0776	-0.24955	0.54821	0.8287	0.21404		Carfentrazone-ethyl	-0.2252737	-	-	-	28.4684	411.183831
NEG08633	131276.8472	136262.9437	0.053781	0.885679	0.961	0.00793		3,4,5-trihydroxy-6-[2-hydroxy-4-(3,5,6,7-tetrahydroxy-4-oxo-4H	3.351991	-	-	-	28.4655	493.35538
NEG08639	487144.4792	408476.3593	-0.2541	0.624489	0.8594	0.27101		Alginic acid	-24.716765	-	-	-	28.4317	417.209886
NEG08645	234556.0015	516183.692	1.137952	0.348794	0.732	0.43874		3,5-Di-O-galloyl-1,4-galactarolactone	-21.763065	-	-	-	28.3633	495.313922
NEG08651	281093.5683	224447.0148	-0.32468	0.730121	0.9083	0.05365		Molybdopterin precursor Z	3.8206667	-	-	-	28.31595	343.191438
NEG08655	1417324.379	923208.9658	-0.61844	0.212378	0.6634	0.83403		Gamma-Glutamyl-Se-methylselenocysteine	-18.476379	-	-	-	28.2157	310.176974
NEG08657	175623.4843	450572.7023	1.359274	0.151107	0.6145	0.51954		1-(5'-Phosphoribosyl)-5-formamido-4	1.7450926	-	-	-	28.2023	365.214662
NEG08658	205149.6004	626164.7974	1.609866	0.008182	0.2511	0.8807		Triazolam	15.949716	-	-	-	28.1796	342.208198
NEG08659	27147.96128	92513.40781	1.768819	0.001354	0.1079	0.34217		Adenosine 5'-phosphate disodium	-14.782313	-	-	-	28.1796	390.171841
NEG08660	2105879.633	8334610.528	1.984692	0.001832	0.1169	3.48382		Propicozole	-22.349512	-	-	-	28.163	341.205475
NEG08662	101038275.6	391403817.7	1.953756	0.001588	0.1127	23.8497		Etobenzanid	15.661582	-	-	-	28.1585	339.199251

NEG08665	818604.6189	3254264.674	1.991093	0.001567	0.1127	2.18634	Boscalid	-2.9309088	-	-	-	28.1585	342.198417
NEG08673	174089.7902	125793.7889	-0.46877	0.237539	0.6801	0.24447	Bromobenzene-2,3-dihydrodiol	-4.5684148	-	-	-	28.14285	190.014451
NEG08677	9880919.571	6245473.985	-0.66183	0.180228	0.6397	2.30295	Tricalcium phosphate	14.21979	-	-	-	28.02935	309.173834
NEG08685	221334.2722	769605.2604	1.797892	0.075978	0.5279	0.85168	Urate D-ribonucleotide	-15.684557	-	-	-	27.93675	379.19156
NEG08687	305859.5273	34581.178	-3.14481	0.073689	0.5234	0.64421	Sanguisorbic acid dilactone	-12.608905	-	-	-	27.93055	469.283093
NEG08691	702484.9048	680561.951	-0.04574	0.77458	0.9234	0.11905	3,4,5-trihydroxy-6-{[5-hydroxy-2-(3-hydroxyphenyl)-4-oxo-8 [illegible]	9.1396777	-	-	-	27.88225	577.477011
NEG08703	145205.0815	96742.70878	-0.58587	0.012532	0.2923	0.28621	Bis(4'-chlorophenyl)acetate	11.983133	-	-	-	27.8181	280.130092
NEG08708	492649.4149	470818.0713	-0.06539	0.691622	0.8929	0.13388	Emblicanin A	-6.4620361	-	-	-	27.8041	781.512967
NEG08709	12387562.64	13950566.6	0.171431	0.349465	0.7322	0.69329	Hydroiodic acid	-1.6279761	-	-	-	27.801	126.904915
NEG08716	1750340.89	395232.9987	-2.14686	0.083688	0.5411	1.47571	8-Hydroxy-3,4-dichlorodibenzofuran	-2.2078196	-	-	-	27.6801	252.072965
NEG08717	112167.5608	65610.72067	-0.77365	0.02024	0.3444	0.27965	3,4,5-trihydroxy-6-{[5-hydroxy-2-(3-hydroxyphenyl)-3-[(3,4,5 [illegible]	-13.163826	-	-	-	27.64595	578.472095
NEG08724	1297298.631	1198554.34	-0.11422	0.25839	0.6898	0.26911	Amaranth	0.5141595	-	-	-	27.5376	603.466034
NEG08726	6520888.789	5718319.763	-0.18948	0.276872	0.6995	0.94099	1,1-Dichloro-2,2-diphenylethane	2.8067696	-	-	-	27.527	250.144528
NEG08732	166542.1009	57443.80806	-1.53566	0.000948	0.0947	0.45751	3,4,5-trihydroxy-6-({6,7,11,12,13,21,22,23-octahydroxy-3,16-dioxo [illegible]	11.132911	-	-	-	27.487	657.474054
NEG08735	66615.65762	525099.2494	2.978657	0.135368	0.5997	0.74235	2-Bromo-5-hydroxy-N-[2-(4-hydroxyphenyl)ethyl]-4-methoxy-N [illegible]	13.795649	-	-	-	27.4275	379.231069
NEG08736	4798474.434	2790088.332	-0.78227	0.146579	0.6131	1.64887	1-(5'-Phosphoribosyl)-5-amino-4-imidazolecarboxamide [illegible]	3.4811412	-	-	-	27.38525	337.205101
NEG08740	90629.1209	276104.8314	1.60717	0.327682	0.721	0.36692	UK-47265	-2.4676441	-	-	-	27.23875	338.171886
NEG08743	154094.102	116769.6455	-0.40015	0.203616	0.6615	0.18796	a-L-Arabinofuranosyl-(1->2)-[a-D-mannopyranosyl-(1 [illegible]	-2.1605872	-	-	-	27.21655	473.402798
NEG08746	83027.26106	117658.8807	0.502953	0.16181	0.6225	0.2043	Thiamphenicol	-12.769255	-	-	-	27.1927	355.210375
NEG08749	2169110.668	1647956.774	-0.39643	0.287153	0.7037	0.73707	Cacodylate	7.2856028	-	-	-	27.18465	136.991129
NEG08753	3329753.045	1111641.145	-1.58272	0.147878	0.6145	1.80069	5-Amino-6-(5'-phosphoribosylamino)uracil	-9.9377782	-	-	-	27.163	353.199803
NEG08755	777244.6561	541598.3266	-0.52115	0.081737	0.5377	0.61212	3,4,5-trihydroxy-6-({11-hydroxy-17,18-dimethoxy-13-oxo-6,8,20 [illegible]	-9.9093811	-	-	-	27.163	545.424309
NEG08756	511357.6877	452196.7908	-0.17738	0.267289	0.6945	0.24586	Pelargonidin 3-O-3'',6''-O [illegible]	-4.2293309	-	-	-	27.1606	604.467963

NEG08759	988718.6439	1008569.221	0.028678	0.873088	0.9572	0.04321	2,8-bis-Trifluoromethyl-4-quinoline carboxylic acid	15.264375	-	-	-	27.0274	308.161443
NEG08760	153922.4524	87065.68869	-0.82203	0.331175	0.7231	0.27132	5-Amino-6-(5'-phospho-D-ribitylamino)uracil	6.0346371	-	-	-	27.0126	355.221273
NEG08765	1712606.568	509763.5397	-1.74829	0.10607	0.5677	1.30403	dehydrofelodipine	1.0147657	-	-	-	26.9132	381.231111
NEG08767	722863.4299	635123.8485	-0.18669	0.16719	0.6281	0.34592	6-[4-({[2-(3,4-dihydroxyphenyl)-5,7-dihydroxy-3,4-dihydro	-17.85902	-	-	-	26.9	617.481678
NEG08770	866170.7311	705858.9327	-0.29527	0.178592	0.638	0.43975	Cycloprothrin	-24.64241	-	-	-	26.8855	481.336037
NEG08777	168480.2644	102411.6557	-0.7182	0.031957	0.4009	0.30283	Eriodictyol 7-(6-galloylglucoside)	-5.55798	-	-	-	26.8532	601.486375
NEG08782	562190.6244	116605.575	-2.26942	0.088266	0.5472	0.83833	WIN56291	-15.051758	-	-	-	26.8361	354.203177
NEG08786	186291.41	83246.85724	-1.16209	0.000299	0.0947	0.47507	Transfluthrin	-9.0602185	-	-	-	26.8264	370.143561
NEG08787	1012927.706	692638.1086	-0.54836	0.46136	0.7838	0.51451	Isopentenyladenosine-5'-diphosphate	-17.78832	-	-	-	26.8092	494.302013
NEG08788	224269.237	164185.5278	-0.4499	0.063079	0.5039	0.27319	Kaempferol 7-(6''-galloylglucoside)	-4.7413368	-	-	-	26.79975	599.471076
NEG08794	1083462.183	925866.3556	-0.22677	0.128539	0.5925	0.49837	3-Phosphonooxypyruvate	-24.076321	-	-	-	26.7078	183.029692
NEG08795	52425.17617	33926.27119	-0.62786	0.072533	0.5226	0.16912	1-Caffeoyl-5-feruloylquinic acid	-9.2544374	-	-	-	26.6856	529.465214
NEG08801	126670.4863	72201.22042	-0.81099	0.017017	0.3231	0.323	11-[(6-carboxy-3,4,5-trihydroxyoxan-2-yl)oxy]-6-hydroxy-3	3.5512408	-	-	-	26.5486	532.411618
NEG08809	9330148.971	10268763.04	0.13829	0.502496	0.8038	0.61619	3,4-Dihydro-3-hydroxy-4-S-glutathionyl bromobenzene	-8.4775364	-	-	-	26.52395	479.319451
NEG08812	388447.3111	372987.8143	-0.05859	0.767419	0.9216	0.10525	Phenobenzuron	-1.9843579	-	-	-	26.5181	336.192654
NEG08814	327816.4233	110165.4982	-1.57322	0.26967	0.6959	0.53362	Chloramben	-23.252533	-	-	-	26.5178	205.014033
NEG08815	1722554.258	1579581.117	-0.12501	0.39542	0.7526	0.32669	[2-hydroxy-5-(3,5,6-trihydroxy-4-oxo-7-{[3,4,5-trihydroxy-6	12.156714	-	-	-	26.5178	559.439537
NEG08816	2853434.567	3103169.063	0.121043	0.566771	0.8343	0.27188	T 0901317	-5.5019409	-	-	-	26.51195	480.322775
NEG08817	255446.9533	183646.8432	-0.47609	0.083837	0.5411	0.31514	Karakin	15.713089	-	-	-	26.5061	482.338618
NEG08823	191431.4051	101286.0562	-0.91839	0.007126	0.2376	0.41074	Carmustine	-11.125908	-	-	-	26.4731	213.040242
NEG08829	5047992.39	3800511.709	-0.40952	0.192195	0.65	1.36164	4,4'-Methylene-bis-(2-chloroaniline)	17.289223	-	-	-	26.4098	266.151142
NEG08832	4574827.296	4716169.846	0.043898	0.808262	0.9344	0.11414	L-Threonylcarbamoyladenylate	-15.959949	-	-	-	26.3801	491.319466
NEG08834	98704.35235	140756.338	0.512014	0.555714	0.83	0.13875	3,4,5-trihydroxy-6-{3,4,8,9,10-pentahydroxy-6-oxo-6H	12.808609	-	-	-	26.3471	495.332081

NEG08839	97139.35879	1030533.944	3.407192	0.339431	0.7277	0.82266	N-Adenylylanthranilate	10.018528	-	-	-	26.2665	465.339295
NEG08841	1284135.834	1179328.866	-0.12283	0.284511	0.7021	0.27166	Fucofuroeckol B	14.865245	-	-	-	26.2665	477.361234
NEG08842	734520.2374	573270.4006	-0.35759	0.06347	0.5039	0.50348	3,3',5,5'- Tetrahydroxy-6,7- methylenedioxy-4' [illegible]	-12.164699	-	-	-	26.2631	535.382098
NEG08854	52873.32488	71974.11136	0.444938	0.469411	0.7895	0.09196	Thiamine(1+) Diphosphate(1-)	-13.53316	-	-	-	26.2211	423.292981
NEG08856	43982.90405	17054.33107	-1.3668	0.004796	0.1938	0.21929	Cyclochlorotine	15.483304	-	-	-	26.20015	571.439787
NEG08861	128635.7696	115997.5936	-0.1492	0.789451	0.9279	0.05505	(2,3- Dihydroxybenzoyl)adenyl [illegible]	0.5565303	-	-	-	26.1751	482.319092
NEG08862	126783.2224	69543.60771	-0.86637	0.033607	0.405	0.27436	Cyanidin 3-O- 3'',6''-O- [illegible]	-20.889509	-	-	-	26.1694	620.456941
NEG08864	110400.4806	58079.82733	-0.92664	0.002464	0.1319	0.29369	Heme	-23.322302	-	-	-	26.1569	615.465645
NEG08865	157164.4185	93324.25999	-0.75195	0.086178	0.5433	0.32772	L-Selenocystathionine	-10.341811	-	-	-	26.14575	268.14704
NEG08870	58881865.32	56289858.5	-0.06495	0.703737	0.8994	1.72892	Potassium dichromate	5.6740763	-	-	-	26.142	293.178993
NEG08873	711155.4505	644169.9653	-0.14272	0.489515	0.7998	0.28771	(R)-4'- phosphonatopantothenate(3-)	-5.2340562	-	-	-	26.1285	295.184173
NEG08875	8602056.738	8241314.591	-0.06181	0.716058	0.9036	0.63751	Aminoimidazole ribotide	10.536901	-	-	-	26.124	294.182234
NEG08876	341899.2657	277228.8761	-0.30249	0.032155	0.4009	0.28656	(6-carboxy-3,4,5- trihydroxyoxan-2-yl)[2- (3,4-dihydroxyphenyl) [illegible]	6.1136946	-	-	-	26.0823	478.364654
NEG08878	338375.8405	276463.028	-0.29154	0.21931	0.6677	0.29475	Enilcozole	18.03123	-	-	-	26.04255	296.177882
NEG08879	2037407.67	1922095.835	-0.08405	0.506286	0.8064	0.30712	Metaflumizone	0.1478252	-	-	-	26.04255	505.392698
NEG08881	158014.511	112490.6662	-0.49025	0.052462	0.4813	0.24036	Gossypetin 8-glucuronide 3-sulfate	7.0682103	-	-	-	26.0289	573.418784
NEG08882	7545416.254	2187147.717	-1.78655	0.076248	0.5279	2.44799	Diclofenac acyl glucuronide	18.832519	-	-	-	26.0207	471.274617
NEG08884	105799.0812	138297.2007	0.386445	0.445971	0.7779	0.15226	3,3'-Biflaviolin	-6.6594692	-	-	-	25.95625	409.277391
NEG08890	201670.2921	67677.87216	-1.57524	0.018368	0.3337	0.44825	Isoimide	-22.147619	-	-	-	25.93085	305.140543
NEG08893	617681.1981	498766.7209	-0.3085	0.121284	0.5846	0.39063	Polyoxin B	-5.9747525	-	-	-	25.9067	506.395792
NEG08897	190368.0781	141827.3627	-0.42466	0.075463	0.5272	0.27688	1-O,2-O,6-O-Trigalloyl- beta-D-glucose	20.829553	-	-	-	25.88625	635.474681
NEG08900	131152.6171	135035.4548	0.042092	0.893171	0.9639	0.03188	L-2-Aminoadipate adenylate	13.552896	-	-	-	25.8745	489.361169
NEG08905	20197287.54	20208571.78	0.000806	0.995921	0.9988	0.42758	CGH 2466	15.528081	-	-	-	25.8516	321.210127

NEG08915	47302.96763	16996.07109	-1.47673	0.001801	0.1158	0.23277	5'-Phosphoribosylglycimide	-20.696615	-	-	-	25.7903	285.163201
NEG08918	1418103.021	1331861.55	-0.09052	0.513378	0.811	0.21372	Luteolin 7-O-(6''-malonylglucoside)	15.029565	-	-	-	25.75895	533.423856
NEG08919	885478.1014	669105.8791	-0.40422	0.012813	0.2932	0.60482	dCMP	-9.9629822	-	-	-	25.7416	306.186763
NEG08920	1157397.603	685434.8759	-0.75579	0.055841	0.4884	0.73669	Fosetyl	-8.5068836	-	-	-	25.7276	109.040687
NEG08922	452649.9429	342964.1291	-0.40034	0.018395	0.3337	0.39039	5''-Phosphoribosylparomamin	-5.3540515	-	-	-	25.70655	534.427157
NEG08923	115921.1336	100149.1847	-0.21099	0.264696	0.6928	0.11541	6-[3,4-dihydroxy-5-(3,4,5-trihydroxybenzoyloxy)benzoyloxy]-3,4,5-	-23.540599	-	-	-	25.6865	497.329992
NEG08929	664811.3123	457237.7302	-0.54	0.00952	0.2615	0.57062	Tiron	3.0425372	-	-	-	25.5907	313.194379
NEG08936	58360.93751	52680.74336	-0.14773	0.596641	0.8463	0.06028	Adenosine thiamine diphosphate	16.464265	-	-	-	25.5795	673.543829
NEG08938	235906.9199	217477.5031	-0.11735	0.562166	0.8325	0.09944	Dantrolene sodium anhydrous	-5.4397756	-	-	-	25.5772	335.225694
NEG08939	127241.5837	101560.6168	-0.32523	0.102458	0.5639	0.14457	O-(1->4)-alpha-L-Dihydrostreptosyl-streptidine 6-phosphate	3.719353	-	-	-	25.5602	487.38194
NEG08940	107624.9509	85659.27175	-0.32933	0.260646	0.6904	0.13485	Carumom sodium	11.807361	-	-	-	25.54515	509.366149
NEG08941	119846.0731	164460.7923	0.456561	0.560971	0.8325	0.16923	3-Iodothyronamine	-14.063272	-	-	-	25.5321	354.158629
NEG08945	283500.3824	216216.5734	-0.39087	0.136434	0.6016	0.26225	6-({5,7-dihydroxy-2-[4-hydroxy-3-(sulfooxy)phenyl]-4-oxo	20.364151	-	-	-	25.5056	557.424095
NEG08951	602757.9244	498094.4066	-0.27516	0.136187	0.6016	0.33646	6-Hydroxyluteolin 6-xyloside	-18.544895	-	-	-	25.4484	433.334968
NEG08952	284075.7789	227950.5501	-0.31755	0.301617	0.7148	0.19322	2-O-[2-O-(alpha-D-Mannopyranosyl)-alpha-D-glucopyranosyl]-3	-2.4339834	-	-	-	25.4484	509.329881
NEG08959	118333.5734	80217.43934	-0.56087	0.066803	0.5151	0.24529	Hexaflurate	-4.8754701	-	-	-	25.3656	227.001912
NEG08961	49969.90324	42673.02127	-0.22774	0.488169	0.7991	0.07801	({5-[(2-{[5,7-dihydroxy-2-(4-hydroxyphenyl)-4-oxo-4H-chromen-3-yl]oxy)	-21.432764	-	-	-	25.3656	659.528566
NEG08964	146519.5948	159087.7829	0.118729	0.885922	0.961	0.01624	Griseorhodin A	13.018267	-	-	-	25.34	507.386642
NEG08965	359211.5362	329406.7101	-0.12496	0.405694	0.7584	0.16366	N,N'-Diacetylchitobiose 6'-phosphate	8.2543983	-	-	-	25.31495	503.377087
NEG08986	623373.9536	449942.9806	-0.47036	0.182031	0.6415	0.41743	3,4,5-trihydroxy-6-({6-[3-(4-methoxy-1-benzofuran-5-yl)-3-oxopropanoyl]-2H	-3.2874775	-	-	-	25.17355	529.42898
NEG08991	827159.1112	703713.7213	-0.23318	0.194087	0.6519	0.33666	(2-{[2-(3,4-dihydroxyphenyl)-5,7-dihydroxy-4-oxo-3,4	-18.283793	-	-	-	25.09165	515.413281
NEG08992	229893.2224	196862.8614	-0.22377	0.244579	0.6836	0.15369	11-[(6-carboxy-3,4,5-trihydroxyoxan-2-yl)oxy]-6-hydroxy-3-methyl-7	11.417562	-	-	-	25.0674	516.416631
NEG08996	614864.6787	463371.7466	-0.4081	0.052185	0.4813	0.45028	Maclurin 3-C-(6''-p-hydroxybenzoyl	-16.425114	-	-	-	25.02935	543.444781

NEG08998	384277.6927	326818.5665	-0.23366	0.217107	0.6662	0.21641	Quercetin 3,3',7-trissulfate	20.300834	-	-	-	24.9785	541.429035
NEG09005	115498.9826	94008.81001	-0.29701	0.433899	0.77	0.08819	6-({13,14-dihydroxy-9-oxo-8,17-dioxatetracyclo[8.7.0.0	24.190794	-	-	-	24.9285	459.35086
NEG09008	242510.5253	182739.8829	-0.40826	0.485582	0.7985	0.13916	Plutonium	16.581391	-	-	-	24.92	242.996769
NEG09016	107727.6979	91278.52719	-0.23904	0.386214	0.7487	0.08072	3,4,5-trihydroxy-6-({11-hydroxy-13-oxo-6,8,20-trioxapentacyclo[10.8.0.0	-23.117727	-	-	-	24.8478	485.366479
NEG09022	163708.8139	129281.8247	-0.34061	0.322106	0.7192	0.15194	Ketoconazole	16.284716	-	-	-	24.80615	530.432378
NEG09024	212696.2413	162461.8628	-0.38869	0.680266	0.8871	0.07094	2-Caffeoylisocitrate	-19.281563	-	-	-	24.73775	353.251593
NEG09031	71408.44765	60292.94501	-0.24411	0.303019	0.7152	0.09361	(2-{[2-(3,4-dihydroxyphenyl)-5,7-dihydroxy-4-oxo-4H	-10.010511	-	-	-	24.678	513.397574
NEG09034	92040.33281	172074.4578	0.902695	0.279757	0.7002	0.25696	TG(10:0/10:0/20:0)	15.573055	-	-	-	24.647	694.126549
NEG09036	138070.2434	124740.5244	-0.14647	0.559803	0.8319	0.1005	Luteoskyrin	18.124084	-	-	-	24.6232	573.491635
NEG09037	48564.7244	44377.03538	-0.1301	0.555808	0.83	0.04578	dTDP-5-dimethyl-L-lyxose	-10.788705	-	-	-	24.6075	561.342756
NEG09040	57782.20332	65777.35221	0.186966	0.453766	0.7814	0.0527	3,4,5-trihydroxy-6-[2-hydroxy-3-(7-hydroxy-4-oxo-4H-chromen-2-yl)-6	-1.6866164	-	-	-	24.58255	475.38192
NEG09044	2051885.559	582227.1606	-1.8173	0.043314	0.4467	1.37447	3,4-Bis(7-chloroindol-3-yl)pyrrole-2,5-dicarboxylate	20.265196	-	-	-	24.5313	453.264229
NEG09046	1966804.161	2032813.489	0.047625	0.752305	0.9154	0.08726	alpha-1,5-L-Arabinotetraose	-11.331902	-	-	-	24.51135	545.460331
NEG09047	679401.4596	631918.9605	-0.10452	0.543871	0.827	0.16017	BMS 379224	-0.0894555	-	-	-	24.51135	546.463674
NEG09054	162429.6624	135661.7189	-0.2598	0.223829	0.6704	0.16537	2''-O-Acetylisoorientin	-17.327825	-	-	-	24.4914	489.397826
NEG09056	1218526.513	1129782.425	-0.10909	0.486943	0.799	0.25488	Apigenin 7-(6''-malonylglucoside)	24.328229	-	-	-	24.4634	517.429036
NEG09057	864078.2245	762313.7663	-0.18078	0.233803	0.6779	0.33318	Biochanin A 7-O-(6-O-malonyl-beta-D-glucoside)	3.0608897	-	-	-	24.4354	531.444653
NEG09059	365867.5635	326013.0961	-0.16639	0.27816	0.6995	0.18675	Pelargonidin 3-O-(6-O-malonyl-beta-D-glucoside)	15.463312	-	-	-	24.41205	518.432356
NEG09060	234744.5772	200640.626	-0.22648	0.211532	0.6624	0.18947	8-Hydroxyluteolin 4'-methyl ether 8-glucoside 3'-sulfate	2.3342592	-	-	-	24.41205	557.460027
NEG09064	151087.2034	354067.0375	1.228641	0.551308	0.8296	0.3245	Selenohomocystine	-11.490512	-	-	-	24.3887	361.132162
NEG09068	16023.39928	46351.89835	1.532448	0.266845	0.6945	0.16367	DG(22:0/0:0/18:2n6)	0.0774547	-	-	-	24.3565	690.127777
NEG09075	95937.87096	44821.05945	-1.09792	0.028882	0.38	0.27676	Ribose-1-arsenate	-23.582636	-	-	-	24.2917	273.04386
NEG09081	591015.8165	536931.0897	-0.13846	0.710358	0.9015	0.128	8-Bromoadenosine	19.93044	-	-	-	24.2048	345.137022

NEG09090	199604.5713	163697.7604	-0.28611	0.174562	0.6336	0.19113	Prunin 6''-O-gallate	2.2723894	-	-	-	23.95485	585.491656
NEG09091	63946.9545	56951.76027	-0.16714	0.47606	0.7932	0.06086	({3,4,5-trihydroxy-6-[3,5,7-trihydroxy-2-(2,4,5-trihydroxyphenyl)-3,4	6.246611	-	-	-	23.93225	547.466149
NEG09093	365977.7071	87344.59618	-2.06697	0.116378	0.5761	0.53152	Bis(1L-myo-inositol)-3,1'-phosphate 1-phosphate	0.1476464	-	-	-	23.9048	501.249098
NEG09098	54622.00714	36390.1343	-0.58593	0.013184	0.2953	0.17414	Celecoxib glucuronide	3.2975791	-	-	-	23.8882	558.463568
NEG09107	975659.0092	1037888.766	0.089203	0.693229	0.8938	0.0593	Phosphonoacetaldehyde	2.8721359	-	-	-	23.67505	123.02558
NEG09109	590380.8885	850171.5961	0.526108	0.074877	0.5262	0.56254	2,3-Diketo-5-methylthiopentyl-1-phosphate	4.8903426	-	-	-	23.634	241.180608
NEG09123	220858.9771	233019.6123	0.077326	0.800088	0.9311	0.02136	Lead arsete	1.6340307	-	-	-	23.45755	346.120391
NEG09126	381343.0117	333821.3389	-0.19201	0.391783	0.7513	0.14418	2,4,5-trihydroxy-3-[3-hydroxy-4-(sulfooxy)-5-(3,4,5	8.3669964	-	-	-	23.4435	569.387496
NEG09127	33639.14202	51625.86557	0.617953	0.403727	0.7578	0.09956	4'-O-Demethylrebeccamycin	-15.233912	-	-	-	23.3727	555.335248
NEG09131	711423.5522	105239.6348	-2.75703	0.134955	0.5997	0.73915	Adenylylselete	19.013352	-	-	-	23.3329	473.181139
NEG09132	132273.7318	192790.6073	0.543508	0.549834	0.8291	0.1647	2-(Formamido)-N1-(5'-phosphoribosyl)acetamidi	-24.121446	-	-	-	23.2919	312.186869
NEG09136	350742.0473	329371.6515	-0.09069	0.832678	0.9447	0.01426	IACI	-15.567973	-	-	-	23.21245	597.418807
NEG09138	411206.5877	418030.1034	0.023743	0.907158	0.9697	0.06292	Ubiquinone-8	1.3726581	-	-	-	23.19515	726.103221
NEG09139	93496.24524	90265.54919	-0.05073	0.905861	0.9697	0.01291	CMP-N-acetyl-beta-neuraminate(2-)	4.5200193	-	-	-	23.1938	611.434492
NEG09142	386742.3483	470691.7745	0.28341	0.187332	0.6466	0.24657	(3beta,17alpha,23S)-17,23-Epoxy-3,29-dihydroxy-27-norlanosta	24.711047	-	-	-	23.18495	469.645153
NEG09143	411799.0163	475221.0409	0.206658	0.318966	0.7192	0.1799	Decachlorobiphenyl	-5.0044436	-	-	-	23.1824	497.648628
NEG09144	183426.4048	223608.0932	0.285771	0.208743	0.6619	0.16276	Fexofedine	-10.414217	-	-	-	23.1824	500.643899
NEG09145	1537650.452	4720441.261	1.618194	0.221092	0.6692	1.81204	TG(10:0/10:0/18:0)	-3.7653726	-	-	-	23.1824	666.059212
NEG09146	180498.501	591458.9401	1.712291	0.234275	0.6785	0.64586	2-Octaprenyl-6-methoxyphenol	-3.9964336	-	-	-	23.1824	668.063449
NEG09149	708358.3088	846405.9515	0.25687	0.151523	0.6145	0.32766	Triamcinolone hexacetonide	7.894041	-	-	-	23.1773	531.637628
NEG09151	1	13216.56397	13.69006	0.327312	0.721	0.10977	2,3-Bis-O-(geranylgeranyl)-sn-glycero-1-phospho-L	-11.176062	-	-	-	23.17685	803.070937
NEG09152	180238.3582	29528.07831	-2.60975	0.166856	0.6281	0.35089	2',3'-Dideoxyadenosine-5-triphosphate	20.352005	-	-	-	23.1757	474.184594
NEG09156	176124.9449	255245.9467	0.535289	0.156741	0.6186	0.27389	3,4,5-trihydroxy-6-({3-hydroxy-2-methyl-6-[(3,4,5,6	-15.156921	-	-	-	23.17445	647.546893

NEG09159	5342742.989	6376807.715	0.255254	0.127062	0.5923	0.93572	Proscillaridin	-1.1387559	-	-	-	23.16185	529.641819
NEG09167	42127.12173	180289.9337	2.097498	0.198159	0.6561	0.38582	DG(15:0/24:1(15Z)/0:0)	-17.514316	-	-	-	23.13135	664.062675
NEG09169	44577.97867	69116.10151	0.632691	0.045102	0.45	0.17159	Pfaffic acid	-9.3349147	-	-	-	23.1018	439.64651
NEG09170	26951.03979	55299.285	1.03692	0.12553	0.5916	0.15698	N-(2R-Hydroxydocosanoyl)-2S-amino-1,3S,4R	10.287455	-	-	-	23.0913	655.074273
NEG09173	73470.84754	98487.71415	0.422772	0.09335	0.5568	0.14825	Scymnol	-19.533486	-	-	-	23.0099	467.649969
NEG09178	38759.51817	61232.6698	0.659751	0.126603	0.5923	0.14568	beta-D-4-Deoxy-delta4-GlcA-(1->4)-beta-D-Glc-(1->4)-alpha-L	19.040621	-	-	-	22.8913	645.551534
NEG09180	86013.04205	109202.421	0.344378	0.154666	0.6173	0.13081	LysoPE(0:0/22:4(7Z,10Z,13Z,16Z))	10.370541	-	-	-	22.8815	528.644516
NEG09188	26028.78448	40632.78673	0.642536	0.06811	0.5182	0.12388	(17alpha,23S)-Epoxy-28,29-dihydroxy-27-norlanost-8-ene-3,24	-17.007212	-	-	-	22.7884	471.641385
NEG09197	113980.3823	94320.8506	-0.27314	0.126554	0.5923	0.15052	3',5'-Cyclic CMP	-8.4743048	-	-	-	22.4901	304.171337
NEG09204	163710.0682	341675.6434	1.061484	0.033108	0.4037	0.48586	2,6-Dichlorophenolindophenol sodium salt	23.910076	-	-	-	22.3995	289.076959
NEG09259	12034.68425	88420.59441	2.877184	0.081023	0.5377	0.28461	Phytyl phosphate	20.193432	-	-	-	22.0262	375.511226
NEG09264	674311.4098	194531.0724	-1.79341	0.128758	0.5925	0.79607	1,1-Dichloro-2-(dihydroxy-4'-chlorophenyl)-2-(4' [illegible]	18.646149	-	-	-	21.95195	349.02335
NEG09265	35761.50772	65580.80684	0.874866	0.065105	0.5103	0.18718	1,3-Dibenzyltetramethyldisiloxane	16.173287	-	-	-	21.9248	313.567211
NEG09274	233105.3547	213863.4239	-0.12429	0.679793	0.8871	0.10808	Spirasine I	-18.628431	-	-	-	21.882	354.456702
NEG09277	170115.8921	121881.4327	-0.48104	0.181923	0.6415	0.24541	Cetiedil	8.8251004	-	-	-	21.8737	348.526408
NEG09284	823881.5355	1844821.498	1.162972	0.133847	0.5989	0.95838	Cinnarizine	18.138784	-	-	-	21.84765	367.513308
NEG09286	338808.9111	515449.305	0.605359	0.200371	0.6584	0.36781	3,1-1imethyl-1-1ropyl-2-1urantridecanoic acid	-3.4481833	-	-	-	21.84765	349.534515
NEG09291	38355.61083	107528.191	1.487205	0.077247	0.5298	0.26831	N-arachidonoylglycinate	-12.673672	-	-	-	21.83935	359.507154
NEG09297	152645.7661	117272.7391	-0.38032	0.025163	0.3609	0.22756	2,5,6-Tribromo-1-methylgramine	-12.279989	-	-	-	21.8309	423.944505
NEG09299	179819.7659	279959.4054	0.638666	0.186091	0.6456	0.27651	8,11,14-Eicosatrienoylethanolamide	-20.887889	-	-	-	21.8226	348.536022
NEG09301	3489400.003	7955282.864	1.188934	0.125599	0.5916	2.0158	Fentin hydroxide	-23.092944	-	-	-	21.82135	366.013248
NEG09302	44851.54179	33260.2841	-0.43136	0.043789	0.4467	0.12193	Sulfinpyrazone	-6.8834325	-	-	-	21.82135	403.471439
NEG09308	225927.7728	201391.8784	-0.16586	0.230751	0.6744	0.16497	Cortisone acetate	2.3816239	-	-	-	21.81355	401.474382

NEG09313	588988.0749	524032.2808	-0.16858	0.191802	0.6491	0.27944	Clocinizine	21.109144	-	-	-	21.8042	401.97123
NEG09315	225706.8588	200491.6476	-0.17091	0.212506	0.6634	0.14862	Perphezine	20.951257	-	-	-	21.7964	402.969787
NEG09317	81379.68608	208998.5882	1.360753	0.139133	0.6058	0.33905	3-Oxo-4,6-choladienoic acid	-19.982374	-	-	-	21.79365	369.510319
NEG09319	43271.50423	41599.18292	-0.05686	0.854792	0.9527	0.02275	Tangeraxanthin	4.9105611	-	-	-	21.792	483.707104
NEG09323	28432.82039	86785.89987	1.609903	0.081184	0.5377	0.24572	N-Arachidonoyl glycine	-14.610401	-	-	-	21.789	360.505641
NEG09327	54677.83821	41754.99479	-0.38901	0.035635	0.4191	0.13776	Morusin	15.096513	-	-	-	21.7839	419.453571
NEG09336	134420.078	128910.1585	-0.06038	0.72114	0.9054	0.0414	Fenchlorazole-ethyl	10.821539	-	-	-	21.77975	402.47289
NEG09348	871273.1375	1791335.521	1.039839	0.013573	0.3009	1.27795	Arbutin 6-phosphate	-9.4072237	-	-	-	21.6948	351.22051
NEG09352	1622983.329	1552321.936	-0.06422	0.866449	0.9556	0.12821	Dihydrogen phosphate	2.4214612	-	-	-	21.63105	95.9801583
NEG09354	48988.43379	36879.36349	-0.40963	0.124591	0.5902	0.11443	{[1-(4-hydroxyphenyl)-3-(5-methoxy-2,2-dimethyl-2H-chromen-6-yl)prop-2	9.2857681	-	-	-	21.6149	417.456609
NEG09362	48006.8221	41963.87286	-0.19409	0.367891	0.7404	0.07748	N-Desmethyldiltiazem	-15.7763	-	-	-	21.4334	399.477405
NEG09372	873523.737	813941.2468	-0.10192	0.284553	0.7021	0.29057	3-Phospho-D-erythrote	-21.742085	-	-	-	20.4103	215.071225
NEG09376	3189732.47	2766810.663	-0.20521	0.130211	0.5933	0.73335	Tetrafluoroethylene	-13.65956	-	-	-	20.1904	99.0063572
NEG09416	1956457.846	1846480.122	-0.08347	0.694843	0.8944	0.20591	Bowdichione	12.034196	-	-	-	18.08015	297.243313
NEG09418	566835.7538	421812.44	-0.42633	0.024845	0.3595	0.43926	2-Bromoacetaldehyde	22.018136	-	-	-	17.9127	121.94403
NEG09422	1024559.823	997483.1066	-0.03864	0.624754	0.8594	0.15273	Arsenobetaine	9.7080902	-	-	-	17.9086	177.055652
NEG09423	15028546.03	12632742.09	-0.25054	0.409677	0.7602	1.40595	Nitrogen mustard	-2.0878033	-	-	-	17.8799	155.045898
NEG09449	550414.0363	357922.9165	-0.62087	0.024851	0.3595	0.48963	(beta-1-O-[N-(2-hydroxymethyl-3-chlorophenyl)	-5.9407342	-	-	-	15.4705	452.817027
NEG09451	122056.0067	100657.212	-0.27809	0.15297	0.6159	0.16936	3b,8b-Dihydroxy-6b-(3-chloro-2-hydroxy-2-methylbutanoyloxy)	1.6312461	-	-	-	15.1025	415.886403
NEG09455	1358936.238	1299158.137	-0.0649	0.458378	0.7822	0.23094	1,3-Dichloro-2-propanol	2.7552853	-	-	-	14.23965	127.978179
NEG09456	594169.7832	692110.9307	0.220128	0.175229	0.6342	0.25109	Bromobenzene	4.2283295	-	-	-	13.833	156.001287
NEG09462	1896675.723	5260165.476	1.471635	0.008643	0.2551	2.37556	2-Phosphoglycolate	-19.884991	-	-	-	11.9767	155.020921
NEG09477	463076.2138	648337.073	0.485494	0.050935	0.4769	0.49959	2,3-Diketo-5-methylthiopentyl-1-phosphate	4.2048792	-	-	-	9.64508	241.180442

NEG09490	5578482.881	3643373.461	-0.6146	0.007169	0.2376	1.74809			(1S,3R)-3-(2,2-Dichloroethenyl)-2,2-dimethylcyclopropanecarb	-5.5070812		-	-	-	8.3116	208.061372
NEG09494	5289346.07	5659596.371	0.09761	0.729945	0.9083	0.2224			Tetrafluoroethylene	-13.68912		-	-	-	7.998145	99.0063543
NEG09501	3512918.333	3521448.678	0.003499	0.993547	0.9985	0.01806			Dihydrogen phosphate	2.3612566		-	-	-	7.65741	95.9801524
NEG09521	1952303.372	2082520.444	0.093153	0.526992	0.8177	0.19376			Cacodylate	7.5045444		-	-	-	5.644645	136.991159
NEG09522	564549.9418	586695.3407	0.05551	0.867903	0.9556	0.00499			3,4-Dihydroxymandelate	-0.057749		-	-	-	5.623635	183.138813
NEG09524	1182200.241	1126148.568	-0.07008	0.821332	0.939	0.08666			Bowdichione	11.823366		-	-	-	5.40977	297.24325
NEG09545	271991.4102	256074.105	-0.087	0.458736	0.7822	0.1175			Nifurthiazole	8.123015		-	-	-	4.2527	253.217388
NEG09552	503047.6055	590827.0555	0.232041	0.356837	0.7348	0.175			Arbutin 6-phosphate	-9.3379952		-	-	-	3.885485	351.220534
NEG09555	635279.7076	622972.4245	-0.02822	0.742465	0.9121	0.11346			Phenolic phosphate	-10.455537		-	-	-	3.770875	173.082003
NEG09566	1075713.473	1130364.747	0.071495	0.569097	0.8352	0.05849			Fosetyl	-8.4094342		-	-	-	1.827055	109.040698
NEG09571	2307015.021	2456523.882	0.090591	0.766086	0.9214	0.05788			sn-Glycerol 3-phosphate	2.7355407		-	-	-	1.78894	171.066894
POS00001	644908.3471	514592.7478	-0.32566	0.38399	0.6572	0.22385	N,N-dimethylformamide	0.9999947			Organic acids and derivatives	-	-	-	230.985	73.075899
POS00002	14338402.53	7253692.19	-0.9831	0.303586	0.6161	1.46325	2-methylpropan-1-amine	0.9999938			Organic nitrogen compounds	-	-	-	676.616	75.0933961
POS00003	7173846.365	3688727.582	-0.95962	0.567302	0.7784	0.68219	1-Butylamine	0.9999886			Organic nitrogen compounds	-	-	-	4.901865	75.0934029
POS00004	32970728.87	24533805.07	-0.42641	0.032433	0.2773	2.4741	(2R,3R,4S,5R)-2-(6-aminopurin-9-yl)-5-(hydroxymethyl)oxolane	0.9998521			Nucleosides, nucleotides, and analogues	-	-	-	166.103	268.103297
POS00005	106678304.6	46461498.73	-1.19916	0.169583	0.4962	6.13966	hexan-1-amine	0.9998039			Organonitrogen compounds	-	-	-	700.74	102.074182
POS00006	772808.7147	528314.1012	-0.54872	0.071374	0.3591	0.37549	2-(trimethylazaniumyl)acetate	0.9997555			Organic acids and derivatives	-	-	-	257.2395	117.10204
POS00007	3468671.722	2423027.843	-0.51757	0.011092	0.1994	0.91598	Nervonyl carnitine	0.9997231			Organic nitrogen compounds	-	-	-	50.04845	103.075217
POS00008	2225186.129	2354084.055	0.08124	0.748756	0.8819	0.1492	Diethanolamine	0.9996509			Organic nitrogen compounds	C4H11NO2	111-42-2	105.079	180.699	74.9307908
POS00009	132964.5933	115886.9623	-0.19832	0.593701	0.7927	0.05788	Choline	0.9995012			Organic nitrogen compounds	C5H13NO	62-49-7	103.0997	254.315	104.070371
POS00010	11415248.5	13207853.92	0.210434	0.652487	0.8291	0.5631	2-Pyrrolidinone	0.9992938			Organoheterocyclic compounds	-	-	-	628.843	100.075521
POS00011	87302.76893	79085.90128	-0.14261	0.80222	0.9099	0.02475	Palmitic amide	0.9989307			Lipids and lipid-like molecules	-	-	-	36.11655	256.169084
POS00012	62392004.79	62935802.74	0.01252	0.963809	0.983	0.40043	2-Methylpiperidine	0.9987425			Organoheterocyclic compounds	-	-	-	620.1775	100.111877

POS00013	102559831	55778693.51	-0.87868	0.021448	0.2438	5.97838	4-Hydroxybenzylamine	0.9978641	Benzenoids	-	-	-	87.2392	124.075609
POS00014	55409373.5	47753756.5	-0.21452	0.345531	0.6343	1.70815	2-Propionylpyrrole	0.9969503	Organooxygen compounds	-	-	-	117.879	124.075614
POS00015	4174068.255	3097210.979	-0.43048	0.335922	0.6326	0.63104	1-Pyrroline	0.9963931	Organoheterocyclic compounds	-	-	-	126.61	70.0399824
POS00016	7646750.157	5605337.239	-0.44805	0.095967	0.4006	1.10856	2-Ethoxy-5-methylpyrazine	0.9958981	Organooxygen compounds	-	-	-	693.138	138.091232
POS00017	791122.0282	657371.9363	-0.26719	0.049049	0.3174	0.30096	1-Pyrrolidinecarboxaldehyd	0.9953361	Organoheterocyclic compounds	-	-	-	36.47405	99.0804485
POS00018	3847744.576	5374942.544	0.482236	0.367426	0.6468	0.53452	(2S)-2-amino-5-(diaminomethylideneamino)pentanoic acid	0.9950648	Organic acids and derivatives	-	-	-	521.0755	175.118732
POS00019	5235913.216	4648655.773	-0.17163	0.279831	0.5957	0.42865	Fomepizole	0.9949376	Organoheterocyclic compounds	-	-	-	53.2078	84.0443047
POS00020	11748246.62	9808875.335	-0.26029	0.078398	0.3727	1.05115	Adenine	0.9942853	Organoheterocyclic compounds	C5H5N5	73-24-5	135.0545	156.2455	136.061713
POS00021	408579.5692	474758.4288	0.216577	0.656003	0.8308	0.03626	4-(ethylamino)-6-(propan-2-ylamino)-1H-1,3,5-triazin-2-one	0.9941215	Organoheterocyclic compounds	-	-	-	110.45	198.079287
POS00022	286610.4217	249196.102	-0.20181	0.050224	0.3192	0.15099	N,N-bis(2-hydroxyethyl)dodecanamide	0.9939455	Lipids and lipid-like molecules	-	-	-	128.4925	289.247672
POS00023	802638.4615	454391.9015	-0.82081	0.203486	0.5277	0.3634	Lauroyl diethanolamide	0.9928565	Lipids and lipid-like molecules	-	-	-	33.1754	288.268028
POS00024	36427428.99	21561086.81	-0.7566	0.065513	0.3476	3.00908	1-Ethyl-1H-pyrrole-2-carboxaldehyde	0.9906681	Organic oxygen compounds	-	-	-	190.367	124.024219
POS00025	130971033.8	62590128.09	-1.06524	0.051863	0.3215	6.36168	Triethylamine	0.9904337	Organonitrogen compounds	C6H15N	121-44-8	101.1205	686.849	102.127574
POS00026	510086.5244	401279.3136	-0.34614	0.044364	0.3087	0.25102	Acetaminophen	0.9889717	Benzenoids	-	-	-	117.977	152.056564
POS00027	199645.4504	154723.0643	-0.36775	0.184956	0.5109	0.12693	octadecanamide	0.9888915	Lipids and lipid-like molecules	-	-	-	36.45775	283.921469
POS00028	11955671.34	13085272.53	0.130249	0.480049	0.7237	0.55317	2-Ethoxy-6-methylpyrazine	0.9880665	Organic oxygen compounds	-	-	-	20.0215	139.086514
POS00029	1903886.151	3341411.13	0.81151	0.143709	0.4682	0.89065	3-(diaminomethylidene)-1,1-dimethylguanidine	0.9867144	Organic nitrogen compounds	-	-	-	681.898	130.108645
POS00030	23518420.95	30527804.39	0.376333	0.392762	0.662	1.77181	(2R)-2-Hydroxy-2-methylbutanenitrile	0.9857872	Organic oxygen compounds	-	-	-	152.213	100.075574
POS00031	2063813.525	3092773.658	0.583589	0.477605	0.7223	0.47198	alpha-Methylstyrene	0.9849798	Benzenoids	-	-	-	36.7406	118.090205
POS00032	11173739.97	8547816.254	-0.38648	0.187705	0.5138	1.19824	Proline betaine	0.9839025	Organic acids and derivatives	-	-	-	245.005	143.994567
POS00033	1334822.543	4463774.318	1.741616	0.040329	0.2975	1.36541	Gentiatibetine	0.9825724	Organoheterocyclic compounds	-	-	-	16.0994	165.09084
POS00034	605753.3194	377312.0541	-0.68297	0.040611	0.2986	0.3706	7H-purin-6-amine	0.9823222	Organoheterocyclic compounds	-	-	-	184.034	135.055359
POS00035	8494305.775	7986657.411	-0.0889	0.889525	0.9496	0.28606	(腙卤)-2-(2-Furanyl)pyrrolidine	0.9822414	Organic nitrogen compounds	-	-	-	146.524	138.054915

POS00036	6909133.903	6697979.17	-0.04478	0.659639	0.8327	0.14901	2-Methylbutylamine	0.9821789	Organonitrogen compounds	-	-	-	177.351	88.1119193
POS00037	151393.9803	140464.7538	-0.1081	0.408315	0.6733	0.05613	2-aminooctadecane-1,3-diol	0.9784768	Organic nitrogen compounds	-	-	-	26.9983	302.148879
POS00038	6545182.508	5659510.166	-0.20976	0.59372	0.7927	0.31777	2-Acetyl-1-methylpyrrole	0.9776084	Organooxygen compounds	-	-	-	681.408	123.099679
POS00039	155748.61	142294.1522	-0.13034	0.791719	0.9039	0.02516	(2R,3S,4S,5R,6S)-2-(hydroxymethyl)-6-[4-[(E)-3-hydroxyprop-1	0.9767774	Organooxygen compounds	-	-	-	34.8453	364.232248
POS00040	8597230.395	7978880.638	-0.10769	0.769896	0.8937	0.2492	2-Diethylaminoethanol	0.9759037	Organic nitrogen compounds	-	-	-	619.54	118.122435
POS00041	26581852.04	20112101.19	-0.40238	0.568025	0.7785	0.79781	2-Piperidinone	0.9757187	Organoheterocyclic compounds	-	-	-	16.834	100.075553
POS00042	11313151.69	6507896.61	-0.79774	0.086622	0.3863	1.69669	2-phenylethanamine	0.9752192	Benzenoids	-	-	-	16.2023	122.0963
POS00043	17329522.54	12804247.53	-0.43661	0.591608	0.7918	1.16027	Metenamine	0.9750107	Organoheterocyclic compounds	-	-	-	288.801	141.113203
POS00044	5224392.954	5719942.905	0.130737	0.75789	0.8874	0.36656	Eicosapentaenoic acid	0.9736668	Lipids and lipid-like molecules	C20H30O2	10417-94-4	302.2246	99.91335	164.106836
POS00045	182688.2354	107803.7029	-0.76098	0.006557	0.1696	0.25282	3,7-dihydropurin-6-one	0.9731353	Organoheterocyclic compounds	-	-	-	180.658	136.883432
POS00046	1255797.845	1435205.868	0.192653	0.705614	0.8562	0.17478	PA(20:2(11Z,14Z)/22:6(4Z,7Z,10Z,13Z,16Z,19Z))	0.9724809	Lipids and lipid-like molecules	-	-	-	39.41	772.590338
POS00047	3323917.998	798357.3546	-2.05778	0.149954	0.4753	1.25516	2,3,4,5-Tetrahydro-6-(5-methyl-2-furanyl)pyridine	0.9719978	Organoheterocyclic compounds	-	-	-	90.4308	164.052644
POS00048	8941336.878	7879488.757	-0.18239	0.273915	0.5913	0.61308	2,3-Dihydro-5-propanoyl-1H-pyrrolizine	0.971416	Organoheterocyclic compounds	-	-	-	77.856	163.122746
POS00049	25710128.84	15441712.04	-0.7355	0.265861	0.5823	1.734	1,2,3,4,5,6-Hexahydro-7H-cyclopenta[b]pyridin-7-one	0.970571	Organoheterocyclic compounds	-	-	-	114.547	138.091244
POS00050	107483.8159	118986.9866	0.146684	0.625897	0.814	0.05103	6,10,14-Trimethyl-5,9,13-pentadecatrien-2-one	0.9704257	Lipids and lipid-like molecules	-	-	-	33.7384	262.252546
POS00051	2156093.347	1544930.906	-0.48088	0.030183	0.2685	0.6569	1-Methylpyrrolo[1,2-a]pyrazine	0.9676989	Organoheterocyclic compounds	-	-	-	165.014	132.889415
POS00052	2627324.274	834028.4366	-1.65543	0.005665	0.1571	1.21611	Oleamide	0.9675278	Lipids and lipid-like molecules	C18H35NO	301-02-0	281.2719	40.2267	281.283155
POS00053	1371580.479	912335.2162	-0.5882	0.039079	0.2955	0.5658	(R)-2-Hydroxysterculic acid	0.966913	Lipids and lipid-like molecules	-	-	-	36.7726	310.258041
POS00054	171897.7893	121000.7141	-0.50654	0.011969	0.2051	0.20073	2-amino-9-[(2R,4S,5R)-4-hydroxy-5-(hydroxymethyl)oxolan-2	0.9661225	Nucleosides, nucleotides, and analogues	-	-	-	182.142	267.838051
POS00055	326490.119	298206.9179	-0.13073	0.367866	0.6472	0.1002	2-(2-Furanyl)-3,4,5,6-tetrahydropyridine	0.9641355	Organoheterocyclic compounds	-	-	-	70.6313	150.037111
POS00056	1146685.865	608675.5789	-0.91372	0.051881	0.3215	0.60253	(2S)-2-amino-4-methylpentanoic acid	0.9635222	Organic acids and derivatives	-	-	-	97.8208	133.101091
POS00057	8409429.499	4955991.37	-0.76283	0.034257	0.282	1.41343	5-aminopentanoic acid	0.96028	Organic acids and derivatives	-	-	-	625.088	117.110128
POS00058	4664928.866	3460164.924	-0.43101	0.099714	0.4055	0.77143	3-Pentylpyridine	0.9590095	Organoheterocyclic compounds	-	-	-	34.7227	150.127757

POS00059	774578.2695	695271.294	-0.15584	0.361993	0.6439	0.16305	2-(2-hydroxyethylamino)ethanol	0.9557044	Organic nitrogen compounds	-	-	-	299.139	106.086089
POS00060	1322304.377	717830.1716	-0.88134	0.151221	0.4779	0.51388	4,6-Dihydroxy-2-quinolinecarboxylic acid	0.9532144	Organoheterocyclic compounds	-	-	-	163.671	205.07454
POS00061	443992.0402	322621.3943	-0.46069	0.049819	0.3188	0.28137	Fucoxanthin	0.952497	Lipids and lipid-like molecules	-	-	-	32.45055	658.459387
POS00062	1210484.621	1147554.901	-0.07702	0.761542	0.8887	0.05479	4,5-Diethyl-2-methyloxazole	0.9496039	Organoheterocyclic compounds	-	-	-	618.893	139.914511
POS00063	9777022.361	8445297.132	-0.21125	0.206763	0.5314	0.69198	2,5-Dimethyloxazole	0.946994	Organoheterocyclic compounds	-	-	-	39.5031	97.0759565
POS00064	405005.8631	260052.8702	-0.63914	0.069591	0.3558	0.29772	2-Acetylpyrrolidine	0.9458294	Organoheterocyclic compounds	-	-	-	259.3295	114.102369
POS00065	557134.1827	712790.8499	0.355454	0.279548	0.5955	0.25618	Epsilon-caprolactam	0.9457609	Organoheterocyclic compounds	-	-	-	184.032	114.054892
POS00066	439096.2029	311188.9455	-0.49675	0.024157	0.2512	0.30337	5-Pentyloxazole	0.9444758	Organoheterocyclic compounds	-	-	-	672.669	139.914539
POS00067	238898.9956	236801.4658	-0.01272	0.95849	0.9811	0.01693	Dimethyl dialkyl ammonium chloride	0.9442598	Organonitrogen compounds	-	-	-	121.7015	303.307735
POS00068	142046.3445	1691507.484	3.573876	0.157777	0.4863	0.75586	Conhydrinone	0.9438567	Organoheterocyclic compounds	-	-	-	36.1187	141.127375
POS00069	3019111.185	1342682.268	-1.16901	0.174996	0.501	0.83406	2,5-Dihydro-2,4,5-trimethyloxazole	0.9335861	Organoheterocyclic compounds	-	-	-	135.6825	115.05017
POS00070	541952.6832	357093.5044	-0.60187	0.106012	0.4144	0.30574	Methyprylon	0.9090608	Organoheterocyclic compounds	-	-	-	40.8207	184.002805
POS00071	64317.13456	61402.57653	-0.0669	0.83063	0.9228	0.00962	Lutein	0.9083562	Lipids and lipid-like molecules	-	-	-	31.9881	567.455902
POS00072	1710038.162	1150027.914	-0.57236	0.236736	0.557	0.56344	Pyrimidine	0.9057225	Organoheterocyclic compounds	-	-	-	247.131	80.9880442
POS00073	5971477.086	4216424.797	-0.50207	0.000635	0.0721	1.27486	Ketoconazole	0.9039576	Organoheterocyclic compounds	C26H28Cl2N4O4	65277-42-1	530.1488	216.826	87.0915192
POS00074	1554754.986	1419136.913	-0.13167	0.349062	0.636	0.21862	PE(16:0/16:1(9Z))	0.8980114	Lipids and lipid-like molecules	-	-	-	143.507	690.504252
POS00075	2759777.091	1857546.867	-0.57115	0.052683	0.3231	0.78534	Benzyl methyl sulfide	0.8873532	Benzenoids	-	-	-	158.287	127.122871
POS00076	215805.9642	169571.9284	-0.34784	0.127156	0.4454	0.15762	Armillaramide	0.8829603	Lipids and lipid-like molecules	-	-	-	27.7231	555.532722
POS00077	7205990.251	2263785.316	-1.67046	0.027012	0.2593	1.9952	2-Ethyl-5-methyloxazole	0.8788891	Organoheterocyclic compounds	-	-	-	128.858	112.063434
POS00078	1608729.964	1230429.013	-0.38676	0.048306	0.3148	0.49363	6-aminohexanoic acid	0.8756705	Lipids and lipid-like molecules	-	-	-	37.15805	131.106639
POS00079	148049.8408	117811.8101	-0.3296	0.373142	0.6502	0.09157	Oxprenolol	0.8713709	Benzenoids	-	-	-	144.8555	266.002104
POS00080	2354784.479	908463.7508	-1.37409	0.287339	0.6026	0.66264	5-Acetyl-3,4-dihydro-2H-pyrrole	0.8663978	Organoheterocyclic compounds	-	-	-	57.4628	112.050405
POS00081	10212433.5	7510166.441	-0.44341	0.326332	0.6319	1.02559	2-Isopropyl-1,4-benzenediol	0.8661172	Benzenoids	-	-	-	19.20635	152.106823

POS00082	818672.6616	596086.7444	-0.45776	0.118285	0.4332	0.34765	Aprindine	0.8659838	Benzenoids	-	-	-	36.1259	322.258081
POS00083	1454810.196	1074618.918	-0.43701	0.026184	0.2576	0.49355	6-Deoxyfagomine	0.8610939	Organoheterocyclic compounds	-	-	-	33.63935	132.080833
POS00084	560766.1348	335322.1597	-0.74185	0.040764	0.2986	0.38613	Cyclocalopin F	0.8608839	Organoheterocyclic compounds	-	-	-	43.78945	294.18036
POS00085	16639295.09	4810115.883	-1.79045	0.0677	0.3527	2.78048	Methylimidazole acetaldehyde	0.8592418	Organoheterocyclic compounds	-	-	-	621.18	125.115285
POS00086	1926027.293	1743660.901	-0.14351	0.093954	0.3971	0.33779	2,5-Dimethyl-1H-pyrrole	0.8541975	Organoheterocyclic compounds	-	-	-	580.126	96.0442449
POS00087	8727548.002	8040980.195	-0.11821	0.803936	0.9105	0.37565	Trimethylaminoacetone	0.8448173	Organic oxygen compounds	-	-	-	2.558175	116.106834
POS00088	1723600.798	1514112.616	-0.18695	0.267749	0.5849	0.27292	Ethylphosphate	0.8399951	Organic acids and derivatives	-	-	-	40.9505	182.080986
POS00089	18322880.79	16105275.13	-0.18611	0.097735	0.4036	1.12204	Trimethylpyrazine	0.8366887	Organoheterocyclic compounds	-	-	-	197.4195	123.091502
POS00090	86618.85699	121271.6449	0.485489	0.556976	0.7731	0.05435	Linoleamide	0.8344965	Lipids and lipid-like molecules	-	-	-	32.6924	281.050264
POS00091	586573.6672	510638.3936	-0.20001	0.122724	0.4403	0.19051	2,3-Dihydro-7-hydroxy-2-methyl-4-oxo-4H-1-benzopyran-5-acetic acid	0.8335908	Organoheterocyclic compounds	-	-	-	12.01499	236.102286
POS00092	4287703.268	3393773.557	-0.33731	0.217284	0.5384	0.66257	Acetone cyanohydrin	0.8251948	Organic oxygen compounds	-	-	-	67.8803	85.0759012
POS00093	14152109.2	7458545.147	-0.92405	0.005853	0.1609	2.41683	piperazine	0.8182894	Organoheterocyclic compounds	-	-	-	532.1595	87.0915506
POS00094	1401953.624	984889.6257	-0.5094	0.011449	0.2014	0.58533	Isopropylpyrazine	0.8158302	Organoheterocyclic compounds	-	-	-	156.6165	123.040183
POS00095	288193621.2	153600702.6	-0.90785	0.039838	0.2968	9.07638	Tranexamic Acid	0.8137318	Organic acids and derivatives	-	-	-	689.8185	157.133346
POS00096	1	740434.6798	19.49801	0.340893	0.6326	0.37624	alpha-Tocopherol succinate	0.8104298	Lipids and lipid-like molecules	-	-	-	37.8515	531.184042
POS00097	1143271.968	836558.0646	-0.45063	0.117113	0.432	0.41494	(2S,3S,4R)-2-aminooctadecane-1,3,4-triol	0.7898069	Organic nitrogen compounds	-	-	-	56.8296	317.323377
POS00098	3072483.874	2391688.182	-0.36138	0.763988	0.8905	0.48457	Kynuramine	0.7871388	Organic oxygen compounds	-	-	-	57.9245	165.050798
POS00099	87629.83816	79495.89815	-0.14054	0.743454	0.8788	0.04048	Lycopene	0.7839232	Lipids and lipid-like molecules	-	-	-	32.02745	535.470422
POS00100	248473.8085	203085.7977	-0.291	0.110809	0.4228	0.16666	PC(18:1(11Z)/14:0)	0.7813088		-	-	-	159.295	732.380215
POS00101	991994.2185	792154.6793	-0.32455	0.07249	0.3618	0.30419	1-[2-(dimethylamino)-1-(4-methoxyphenyl)ethyl]cycl	0.7775101	Benzenoids	-	-	-	37.22195	277.215471
POS00102	1498144.354	1111571.179	-0.43058	0.015677	0.2226	0.53967	2-amino-2-(hydroxymethyl)propane-1,3-diol	0.772983	Organic compounds	-	-	-	562.691	121.966047
POS00103	264115.1414	242483.1714	-0.12328	0.650697	0.829	0.07045	Phytosphingosine	0.7607878	Organic nitrogen compounds	C18H39NO3	554-62-1	317.293	120.327	317.323322
POS00104	214567.6617	113204.2288	-0.9225	0.024539	0.2524	0.2655	2-O-p-Coumaroylhydroxycitric acid	0.7579365	Phenylpropanoids and polyketides	-	-	-	36.72955	354.247875

POS00105	72522.7308	70910.25694	-0.03244	0.912054	0.9581	0.01144	PE(15:0/15:0)	0.7514854		Lipids and lipid-like molecules	-	-	-	143.357	663.511699
POS00106	596477.1171	635088.6564	0.090491	0.676505	0.8402	0.06125	PC(16:0/16:0)	0.7460592		Lipids and lipid-like molecules	-	-	-	140.633	734.565263
POS00107	529174.546	425674.7511	-0.31399	0.009224	0.1917	0.30082	Ribose-1-arsenate	0.7428154		Organic oxygen compounds	-	-	-	260.607	273.990087
POS00108	1537607.011	1939680.505	0.335132	0.378173	0.6527	0.40546	8-hydroxy-4-oxo-1H-quinoline-2-carboxylic acid	0.7420979		Organoheterocyclic compounds	-	-	-	117.269	205.096924
POS00109	1239462.188	1043226.363	-0.24866	0.501534	0.7379	0.19486	5,8,14-triazatetracyclo[10.3.1.02,11.04,9]hexadeca	0.7367313		Organoheterocyclic compounds	-	-	-	97.8361	211.132439
POS00110	3080516.935	2882313.835	-0.09595	0.387254	0.6585	0.22354	3-Methyl-1-butylamine	0.733444		Organic nitrogen compounds	-	-	-	212.285	88.1119076
POS00111	71014.20413	58872.53282	-0.27051	0.229643	0.5507	0.07476	Cer(d18:0/16:0)	0.7185925		Lipids and lipid-like molecules	-	-	-	33.0966	539.539228
POS00112	189062.4891	437921.3273	1.211809	0.050588	0.3192	0.38808	Chavicol	0.6813758		Benzenoids	-	-	-	54.2681	134.08913
POS00113	5613565.715	4683448.413	-0.26135	0.542476	0.7638	0.33457	2,4-Diamino-6-hydroxypyrimidine	0.6768798		Organoheterocyclic compounds	-	-	-	331.815	126.077288
POS00114	437034.9298	484926.0798	0.150016	0.517328	0.7487	0.06867	PC(22:4(7Z,10Z,13Z,16Z)/14:0)	0.6704215			-	-	-	136.1835	782.566426
POS00115	5554019.315	5077843.011	-0.12932	0.616574	0.8092	0.24831	1,2,3,4,5,6-Hexahydro-5-(1-hydroxyethylidene)-7H-cyclopenta[b]pyridin	0.6528562		Organoheterocyclic compounds	-	-	-	237.336	180.101785
POS00116	117976.7204	113500.1174	-0.05581	0.719598	0.8647	0.01544	Cer(d18:0/14:0)	0.6352478		Lipids and lipid-like molecules	-	-	-	33.13045	511.507479
POS00117	797158.778	838374.2373	0.072727	0.841413	0.9279	0.08051	Pipericine	0.610972		Lipids and lipid-like molecules	-	-	-	12.7431	335.976981
POS00118	55524.13814	70844.886	0.351549	0.268534	0.5855	0.08698	Idoxanthin	0.577698		Lipids and lipid-like molecules	-	-	-	37.9009	598.538411
POS00119	72819.09343	70464.79414	-0.04741	0.8661	0.9397	0.00336	Epomusenin A	0.5685018		Lipids and lipid-like molecules	-	-	-	38.1068	558.53455
POS00120	451734.7413	328039.6441	-0.46161	0.208491	0.5321	0.24175	N-Nitroso-pyrrolidine	0.5507708		Organoheterocyclic compounds	-	-	-	278.896	100.979355
POS00121	490245.0006	507969.789	0.05124	0.935288	0.9711	0.04202	5,6,7,8-Tetrahydro-2,4-dimethylquinoline	0.5474782		Organoheterocyclic compounds	-	-	-	38.5022	161.132433
POS00122	6600927.042	5727594.224	-0.20474	0.474407	0.7206	0.45486	Inositol 1,3,4-trisphosphate	0.5301872		Organic oxygen compounds	-	-	-	407.953	420.969157
POS00123	2308685.583	1306091.206	-0.82182	0.180951	0.5068	0.627	alpha-Methylphenylalanine	0.5231091		Phenylpropanoids and polyketides	-	-	-	257.0225	180.14923
POS00124	242488.8872	221303.4835	-0.13189	0.305709	0.6178	0.1025	PC(22:5(7Z,10Z,13Z,16Z,19Z)/16:0)	0.5176472		Lipids and lipid-like molecules	-	-	-	136.011	808.578938
POS00125	27047.06944	39907.90839	0.561202	0.21448	0.5366	0.07225	Chlorophyll b	0.5165205		Organoheterocyclic compounds	-	-	-	32.9071	906.57811
POS00126	54016.31141	42935.46575	-0.33123	0.511228	0.7452	0.04616	3,5-Dichloro-4-hydroxy-2-methoxy-6-methylbenzoic acid	0.5018735		Benzenoids	-	-	-	187.685	250.041105
POS00127	4202565.137	3458741.442	-0.28102	0.477537	0.7223	0.32294	Dimethylaniline-N-oxide	0.4863843		Benzenoids	-	-	-	719.2135	138.054901

POS00128	457098.2275	299500.6827	-0.60994	0.051248	0.3203	0.30847	1'-Acetoxyeugenol acetate	0.4854602			Benzenoids	-	-	-	38.7922	264.242389
POS00129	107039.7148	103172.9807	-0.05308	0.869127	0.9413	0.00278	Crassostrea Secocarotenoid	0.4081819			Lipids and lipid-like molecules	-	-	-	36.773	616.433864
POS00130	157763.2625	83644.25749	-0.91542	0.000835	0.0732	0.26739	PE(18:2(9Z,12Z)/20:3(5Z, 8Z,11Z))	0.3626898			Lipids and lipid-like molecules	-	-	-	31.44485	765.575457
POS00283	2917117.978	2852284.419	-0.03243	0.855876	0.9346	0.09558			2-Amino-3,4-dihydroxypentanedioic acid	14.740037		-	-	-	719.714	180.138017
POS00286	1003769.225	849697.0964	-0.24041	0.020944	0.2409	0.32295			N-Acetyldemethylphosphinothricin	20.515509		-	-	-	719.7115	210.148467
POS00287	33815904.46	35251715.85	0.059992	0.840705	0.9276	0.28866			Fosetyl	-8.0924409		-	-	-	719.711	111.055286
POS00290	3852575.057	3553746.446	-0.11648	0.749762	0.8827	0.1236			5-Nitrofurfural	-19.26487		-	-	-	719.381	142.086159
POS00291	585530.5159	490833.6709	-0.25451	0.031118	0.2714	0.24169			2-C-Methyl-D-erythritol 4-phosphate	-2.1072348		-	-	-	719.38	217.133121
POS00301	987499.9681	352094.6558	-1.48782	0.182579	0.5082	0.5114			2-Aminophenoxazin-3-one	16.751098		-	-	-	716.714	213.214931
POS00309	747436.7828	201055.8926	-1.89435	0.104307	0.4115	0.61749			Pelargonidin 3-O-(6-O-malonyl-beta-D-glucoside)	-8.9097642		-	-	-	715.725	520.434249
POS00316	2122331.616	1464767.384	-0.53498	0.125238	0.4435	0.60026			Quite	-22.214675		-	-	-	714.391	193.169608
POS00318	1773099.034	1705497.576	-0.05608	0.872256	0.9428	0.08277			5-O-Methyl-myo-inositol	-23.125603		-	-	-	714.38	195.185286
POS00326	793053.4776	676021.9135	-0.23035	0.033468	0.2804	0.27666			Boc-Asn-OPhNO2	2.1000262		-	-	-	713.725	354.335319
POS00335	1431357.489	1125149.242	-0.34727	0.388734	0.6595	0.34055			Glyphosate-isopropylammonium	-0.3140496		-	-	-	713.046	229.190505
POS00344	2639081.539	2334182.253	-0.17712	0.567046	0.7783	0.21549			N,N'-dinitrosopiperazine	16.158944		-	-	-	712.0475	145.141506
POS00348	3720783.747	3219653.646	-0.2087	0.242749	0.5618	0.38812			2,3,6-Trihydroxypyridine	11.681132		-	-	-	711.3855	128.106861
POS00349	14210540.54	27656555.18	0.96066	0.191458	0.5181	2.28642			(S)-Dihydroorotate	8.4508128		-	-	-	711.3855	159.120713
POS00352	1086279.462	1307284.372	0.267178	0.449864	0.704	0.25194			D-Ribose 5-phosphate	-20.771875		-	-	-	711.045	231.112297
POS00358	5082637.94	2944949.847	-0.78733	0.258369	0.5766	0.9272			1,3,7-Trimethyluric acid	9.7970251		-	-	-	710.395	211.199336
POS00360	442423.4783	691040.1087	0.643342	0.090103	0.3896	0.37153			Didecyldimethylammonium chloride	7.9335209		-	-	-	710.3795	363.086349
POS00363	2566907.365	2649918.819	0.045917	0.911942	0.9581	0.07951			Methyl (2-propenylthio) selenide	4.5989612		-	-	-	710.0475	168.138045
POS00365	1217805.772	1020502.199	-0.255	0.044062	0.3081	0.36157			S-(1,2-Dichlorovinyl)-L-cysteine	17.72278		-	-	-	709.883	217.096706
POS00369	1619025.086	1282839.823	-0.33578	0.233247	0.5541	0.3885			Stipitatote	-18.02368		-	-	-	708.88	209.128025

POS00370	2332612.96	1714871.158	-0.44385	0.150165	0.4755	0.52566	N-Dimethyl-2-aminoethylphosphote	-9.6785145	-	-	-	708.555	154.122495
POS00379	4030506.27	2960134.526	-0.4453	0.363664	0.6446	0.65507	3-Nitroacrylate	-21.093885	-	-	-	706.898	118.065007
POS00384	1469811.37	1163112.873	-0.33764	0.151581	0.4784	0.41524	Tetrahydroxypteridine	2.2087658	-	-	-	706.72	197.12811
POS00391	1885427.762	1167619.11	-0.69132	0.133519	0.4539	0.6347	2,4-Dinitroaniline	22.381763	-	-	-	706.058	184.132975
POS00399	2510196.594	2186240.277	-0.19935	0.519085	0.7499	0.26927	[5-(Aminomethyl)furan-3-yl]methyl phosphate	21.456975	-	-	-	704.8975	208.132821
POS00400	2337816.664	5733679.716	1.2943	0.382321	0.656	1.01278	cis-4-Carboxymethylenebut-2-en-4-olide	8.7055943	-	-	-	704.574	141.102096
POS00402	3300102.862	2743471.473	-0.26651	0.606009	0.802	0.23883	Isopentenyl phosphate	-10.571321	-	-	-	704.396	167.117721
POS00404	2859001.778	2970547.575	0.055217	0.908471	0.9578	0.02036	5-Nitrofurfural	-19.130445	-	-	-	703.401	142.086178
POS00418	14551661.57	25219688.05	0.793367	0.123525	0.441	2.59152	Fosetyl	-8.1513528	-	-	-	702.071	111.05528
POS00421	850546.6595	807407.5546	-0.07509	0.538724	0.7611	0.07181	Calcium glycerophosphate	2.7105971	-	-	-	701.2555	211.143646
POS00432	1093375.88	914227.4945	-0.25816	0.052501	0.3231	0.32479	O-Phospho-4-hydroxy-L-threonine	-19.872622	-	-	-	699.103	216.101502
POS00434	3078904.629	3440895.326	0.160367	0.481012	0.7243	0.25807	2-Amino-3,4-dihydroxypentanedioic acid	14.57505	-	-	-	698.932	180.137987
POS00438	685936.4104	668174.1651	-0.03785	0.887756	0.9486	0.00344	Se-Propenylselenocysteine Se-oxide	-3.8681939	-	-	-	698.757	225.12281
POS00440	1945294.641	2436642.499	0.324906	0.355356	0.6391	0.45531	3-Oxalomalate	-8.9700892	-	-	-	698.107	207.112428
POS00442	977432.1036	795569.1414	-0.29701	0.040183	0.2969	0.35455	Apraclonidine	-15.730637	-	-	-	697.926	246.111921
POS00447	1387096.139	1544551.521	0.15512	0.60196	0.7987	0.16065	3-(Imidazol-4-yl)-2-oxopropyl phosphate	4.0256796	-	-	-	697.766	221.127963
POS00453	1296061.867	918164.0441	-0.49731	0.07699	0.3707	0.46422	D-Ribitol 5-phosphate	-21.907453	-	-	-	697.2725	233.127891
POS00456	2487331.507	1993233.547	-0.31949	0.578398	0.7848	0.30522	Citrate	12.963365	-	-	-	696.775	193.133267
POS00467	3244704.778	2761366.835	-0.2327	0.576619	0.7836	0.3305	Methyl 2-propenyl selenide	-12.11644	-	-	-	694.46	136.07564
POS00471	1715785.323	1439399.096	-0.2534	0.405173	0.6714	0.25415	Oxalosuccite	13.955025	-	-	-	693.9635	191.11753
POS00472	21845352.98	22589445.35	0.048322	0.928675	0.9674	0.02102	N-Formylmaleamic acid	-20.908811	-	-	-	693.9575	144.101785
POS00486	1323339.113	1339213.145	0.017203	0.953345	0.9797	0.01309	4-Nitrophenyl phosphate	1.7340801	-	-	-	691.312	220.096357
POS00492	2511195.284	2830596.242	0.172732	0.708819	0.8579	0.08649	Cytosine	24.450973	-	-	-	690.485	112.111993

POS00497	528073.4947	445022.7483	-0.24686	0.062918	0.3422	0.23572	Dopaquinone	5.7263472	-	-	-	689.826	196.180494
POS00499	1391908.242	999516.4659	-0.47776	0.518355	0.7495	0.2215	5-O-Methyl-myo-inositol	-23.235005	-	-	-	689.495	195.185265
POS00500	1742570.914	1843393.822	0.081147	0.842054	0.9279	0.09002	2-Aminoethyl diphenylborite	24.949155	-	-	-	689.495	226.106793
POS00504	833296.4139	840415.6447	0.012273	0.909473	0.9581	0.00446	2-Amino-5-(5-nitro-2-furyl)-1,3,4-thiadiazole	11.322638	-	-	-	689.165	213.195679
POS00505	2719856.561	2105552.244	-0.36933	0.185822	0.5118	0.4917	Sepiapterin	19.218458	-	-	-	688.836	238.227136
POS00516	1865742.113	2571209.71	0.462698	0.332168	0.6326	0.48269	N-Dimethyl-2-aminoethylphosphote	-9.8127107	-	-	-	687.183	154.122474
POS00525	1888682.984	2170636.118	0.200737	0.675072	0.8396	0.10314	Methyl (2-propenylthio) selenide	4.5350926	-	-	-	686.3565	168.138035
POS00531	1991633.208	2447555.857	0.29739	0.513374	0.7463	0.26729	5-Amino-4-imidazolecarboxyamide	-8.611761	-	-	-	685.533	127.122791
POS00532	2896665.988	3762230.046	0.377195	0.477424	0.7223	0.4154	3-Nitroacrylate	-21.294767	-	-	-	685.3645	118.064984
POS00539	4471723.383	3589337.991	-0.31711	0.546153	0.7665	0.57111	5-Nitrofurfural	-19.376383	-	-	-	684.542	142.086143
POS00543	1304171.911	1289411.175	-0.01642	0.946981	0.9764	0.04336	Dichlormid	-2.7750685	-	-	-	684.044	209.091699
POS00545	1088077.319	1031583.97	-0.07692	0.798931	0.9083	0.08843	Didecyldimethylammonium chloride	7.7932245	-	-	-	683.892	363.086298
POS00546	2231512.693	1943858.525	-0.1991	0.591998	0.7918	0.17371	N,N'-dinitrosopiperazine	16.031981	-	-	-	683.8805	145.141487
POS00549	14022490.31	4515482.439	-1.63479	0.186234	0.5124	1.99466	(S)-Dihydroorotate	8.4227072	-	-	-	683.7215	159.120708
POS00555	1390596.306	1384887.55	-0.00593	0.983611	0.9919	0.04281	D-Ribose 5-phosphate	-20.861732	-	-	-	683.3905	231.112276
POS00571	3711282.308	2501412.001	-0.56918	0.253022	0.5714	0.77864	1,3,7-Trimethyluric acid	9.7599464	-	-	-	681.7455	211.199328
POS00579	1648228.83	1335975.761	-0.30302	0.32462	0.631	0.31817	2,4-Dinitroaniline	22.361992	-	-	-	680.9115	184.132972
POS00580	1090212.991	908065.4691	-0.26374	0.117546	0.432	0.30499	S-(1,2-Dichlorovinyl)-L-cysteine	17.766445	-	-	-	680.9095	217.096716
POS00585	2675386.232	2630781.602	-0.02426	0.953368	0.9797	0.03266	Isopentenyl phosphate	-10.620497	-	-	-	679.594	167.117712
POS00593	19461930.84	6942478.699	-1.48713	0.238867	0.5588	2.20219	cis-4-Carboxymethylenebut-2-en-4-olide	8.7188504	-	-	-	678.1075	141.102098
POS00595	1830691.574	1416320.147	-0.37024	0.455782	0.7088	0.29432	[5-(Aminomethyl)furan-3-yl]methyl phosphate	21.391738	-	-	-	675.966	208.132807
POS00599	34829330.37	21633600.62	-0.68703	0.262704	0.58	2.41614	Calcium hydroxide	-4.3791828	-	-	-	674.323	75.0996521
POS00600	940859.2929	856231.7114	-0.13598	0.349166	0.636	0.15849	N-Acetyldemethylphosphinothricin	20.229401	-	-	-	674.3195	210.148407

POS00605	1814543.334	1841857.514	0.021555	0.959557	0.9815	0.04411	2-Amino-3,4-dihydroxypentanedioic acid	14.482234	-	-	-	672.008	180.137971
POS00608	1385747.586	929587.4561	-0.576	0.073166	0.3629	0.49209	Glyphosate-isopropylammonium	-0.3911514	-	-	-	671.6725	229.190487
POS00615	1148775.787	887455.595	-0.37235	0.156319	0.4846	0.3469	Quite	-22.301345	-	-	-	667.389	193.169591
POS00617	957800.2506	883297.9225	-0.11682	0.599432	0.7972	0.09667	2-Aminophenoxazin-3-one	16.328212	-	-	-	666.898	213.214842
POS00622	1846866.348	1668337.16	-0.14667	0.320495	0.6284	0.2592	Methyl (2-propenylthio) selenide	4.4861906	-	-	-	665.746	168.138026
POS00625	2671448.818	2577239.484	-0.0518	0.907186	0.9573	0.08686	Cytosine	24.620403	-	-	-	665.408	112.112012
POS00628	2058222.246	1873618.341	-0.13557	0.699204	0.852	0.16437	5-Amino-4-imidazolecarboxyamide	-8.6628356	-	-	-	665.084	127.122784
POS00640	1452185.884	1296878.194	-0.16318	0.186654	0.5126	0.25693	3-Oxalomalate	-8.9869955	-	-	-	663.108	207.112424
POS00641	946738.6006	835976.4484	-0.1795	0.442154	0.6982	0.16263	2-Amino-5-(5-nitro-2-furyl)-1,3,4-thiadiazole	11.241793	-	-	-	663.106	213.195662
POS00644	1713391.21	1403848.307	-0.28747	0.043889	0.3077	0.4803	Citrate	12.840127	-	-	-	662.458	193.133243
POS00648	3818591.024	3096405.35	-0.30245	0.306281	0.6184	0.51053	Sepiapterin	19.26452	-	-	-	661.791	238.227146
POS00650	2308840.468	1927252.478	-0.26062	0.261001	0.5788	0.3624	Methyl 2-propenyl selenide	-12.319642	-	-	-	661.4645	136.075613
POS00660	2843066.603	2554224.198	-0.15456	0.653499	0.8295	0.21538	1,3,7-Trimethyluric acid	9.7061609	-	-	-	659.829	211.199317
POS00662	1646313.549	1472260.586	-0.16121	0.460453	0.7119	0.22297	N-Dimethyl-2-aminoethylphosphote	-9.7896438	-	-	-	659.827	154.122478
POS00667	1468313.393	1383183.451	-0.08617	0.779371	0.8987	0.12215	5-O-Methyl-myo-inositol	-23.244811	-	-	-	659.5035	195.185263
POS00669	768493.8751	1002790.507	0.383915	0.31499	0.6245	0.2401	2,4-Dinitroaniline	22.371467	-	-	-	659.5015	184.132973
POS00687	957128.1528	771766.5705	-0.31055	0.047547	0.3143	0.3603	3-(Imidazol-4-yl)-2-oxopropyl phosphate	4.1424046	-	-	-	658.172	221.127988
POS00694	314380.1339	243745.0348	-0.36714	0.003431	0.1265	0.25379	Dopaquinone	6.0874134	-	-	-	657.193	196.180565
POS00706	1397963.52	1301715.718	-0.10291	0.24408	0.5624	0.20012	3-(Phosphoacetylamido)-L-alanine	8.8440521	-	-	-	655.548	243.133218
POS00707	4440965.275	2657453.505	-0.74083	0.038852	0.2951	1.08583	3-Nitroacrylate	-21.36908	-	-	-	655.5475	118.064975
POS00709	440587.6131	400851.2402	-0.13636	0.60036	0.7976	0.1077	Violacene	-1.4185754	-	-	-	655.545	355.932973
POS00712	3084069.778	2426084.418	-0.34621	0.38655	0.6583	0.3638	Phosphoagmatine	6.3978363	-	-	-	655.22	211.180021
POS00713	1693732.039	1385658.102	-0.28963	0.364813	0.6452	0.30046	Didecyldimethylammonium chloride	8.0697732	-	-	-	655.0525	363.086398

POS00714	2723801.813	3350248.208	0.298646	0.41268	0.677	0.38683	N,N'-dinitrosopiperazine	15.977515	-	-	-	655.051	145.141479
POS00715	17565494.83	7562786.969	-1.21575	0.424846	0.685	1.70809	(S)-Dihydroorotate	8.3585401	-	-	-	655.0505	159.120698
POS00730	564775.4709	507323.4445	-0.15477	0.598338	0.7961	0.10682	Boc-Asn-OPhNO2	1.6694456	-	-	-	650.941	354.335166
POS00733	1063599.266	846535.2733	-0.32931	0.033658	0.2804	0.36585	Oxalosuccite	13.848895	-	-	-	649.789	191.117509
POS00738	1868490.503	1267768.302	-0.55958	0.021755	0.2452	0.65475	Quite	-22.269903	-	-	-	647.4955	193.169597
POS00739	2362672.095	1619418.698	-0.54494	0.108439	0.4185	0.60916	Calcium peroxide	0.1772543	-	-	-	646.8305	73.0840894
POS00741	1546025.628	1100651.855	-0.49021	0.041823	0.3006	0.55143	Methyl (2-propenylthio) selenide	4.4780458	-	-	-	646.023	168.138025
POS00760	924252.4637	908903.5476	-0.02416	0.8688	0.941	0.00741	2-Amino-5-(5-nitro-2-furyl)-1,3,4-thiadiazole	11.253688	-	-	-	642.086	213.195664
POS00761	1804841.421	2097528.735	0.216818	0.679271	0.8418	0.14726	1,7-diphospho-1-epi-valienol	20.80426	-	-	-	642.08	337.141269
POS00762	590781.3824	731718.8966	0.308665	0.448496	0.7027	0.20976	D-Ribitol 5-phosphate	-22.116715	-	-	-	641.966	233.127843
POS00767	2417741.199	1514578.908	-0.67474	0.030386	0.2685	0.75416	2-Aminoethyl diphenylborite	24.983034	-	-	-	640.757	226.1068
POS00774	462974.8178	334618.1956	-0.46842	0.003893	0.1351	0.33105	Nicotinurate	9.0343787	-	-	-	638.791	181.169604
POS00780	2577152.518	1971216.537	-0.38669	0.290706	0.6051	0.41816	3-(Phosphoacetylamido)-L-alanine	8.8658717	-	-	-	637.48	243.133223
POS00783	1014146.953	746087.6256	-0.44285	0.087594	0.3867	0.3897	Citrate	12.828976	-	-	-	637.156	193.133241
POS00786	637597.3548	682664.0132	0.09853	0.850022	0.9322	0.03337	[5-(Aminomethyl)furan-3-yl]methyl phosphate	21.449061	-	-	-	636.833	208.132819
POS00797	391466.7749	361803.0098	-0.11369	0.368207	0.6475	0.07967	Apraclonidine	-16.501757	-	-	-	631.7645	246.111732
POS00800	430513.8245	255858.1391	-0.75072	0.192409	0.519	0.29156	Aminopyrrolnitrin	16.675885	-	-	-	630.952	228.100964
POS00801	607803.0242	508138.3403	-0.25838	0.012743	0.21	0.2706	O-Phospho-4-hydroxy-L-threonine	-20.088834	-	-	-	630.812	216.101456
POS00821	200042569.9	53342516.46	-1.90695	0.17008	0.4966	8.36739	Trifluoromethyl-bismethyl ketone	16.490393	-	-	-	626.8975	141.113287
POS00825	21493761.72	47677811.27	1.1494	0.23222	0.553	3.50623	Calcium hydroxide	-4.6307956	-	-	-	626.674	75.0996335
POS00828	8872174.616	6600404.96	-0.42673	0.448417	0.7027	0.69675	Sepiapterin	19.10508	-	-	-	626.1615	238.227109
POS00835	1106763.422	784711.2604	-0.49611	0.004217	0.1383	0.52002	SQ 26180	19.994213	-	-	-	625.569	239.23044
POS00838	1894701.345	946283.8773	-1.00163	0.00356	0.1278	0.9219	Dimefox	-9.2571137	-	-	-	625.368	155.12885

POS00847	3268112.145	2638534.754	-0.30872	0.582128	0.7874	0.28113	(S)-Dihydroorotate	8.2992333	-	-	-	624.472	159.120689
POS00852	2080962.184	1718362.915	-0.27622	0.389957	0.6601	0.28768	Methyl (2-propenylthio) selenide	4.373977	-	-	-	624.155	168.138008
POS00855	5250672.24	8471742.491	0.690157	0.112024	0.4244	1.14366	1,3,7-Trimethyluric acid	9.5043021	-	-	-	624.129	211.199274
POS00867	1282127.05	1173377.167	-0.12787	0.790219	0.9034	0.13114	5-Amino-4-imidazolecarboxyamide	-9.1634938	-	-	-	623.454	127.122721
POS00869	4552425.59	2343208.961	-0.95815	0.036571	0.2867	1.23151	3-Nitroacrylate	-21.460847	-	-	-	623.3055	118.064964
POS00871	1533065.882	809844.7496	-0.9207	0.001866	0.0969	0.78057	Dopaquinone	5.7715595	-	-	-	623.172	196.180503
POS00876	349243.4834	327411.747	-0.09313	0.738616	0.8763	0.03165	Boc-Asn-OPhNO2	1.7999921	-	-	-	623.107	354.335213
POS00879	12477177.11	11558596.25	-0.11033	0.69717	0.851	0.4192	N,N'-dinitrosopiperazine	15.789293	-	-	-	622.834	145.141452
POS00888	3817599.429	2148136.762	-0.82958	0.223506	0.5454	0.84874	Glyphosate-isopropylammonium	-0.5550311	-	-	-	621.993	229.19045
POS00894	7416700.44	4355170.314	-0.76805	0.316618	0.6253	0.9851	Phosphoagmatine	6.2609087	-	-	-	621.833	211.179992
POS00897	2606100.955	1139811.522	-1.1931	0.025601	0.2569	1.07053	5-O-Methyl-myo-inositol	-23.60385	-	-	-	621.6745	195.185193
POS00899	1945877.258	1939920.077	-0.00442	0.988907	0.9942	0.03065	2-Amino-5-(5-nitro-2-furyl)-1,3,4-thiadiazole	11.076017	-	-	-	621.534	213.195627
POS00901	15292041.73	19224843.9	0.330191	0.629404	0.8159	1.20462	Maleimide	-1.5445643	-	-	-	621.5145	98.0792267
POS00907	848861.7927	1967737.335	1.212936	0.095098	0.399	0.80464	2-Aminophenoxazin-3-one	16.466744	-	-	-	621.5025	213.214871
POS00909	8236402.461	4178650.806	-0.97898	0.045535	0.3123	1.6415	Calcium peroxide	0.0516828	-	-	-	621.4805	73.0840803
POS00915	2211474.767	1151202.938	-0.94187	0.055438	0.3276	0.81818	Calcium formate	-18.471167	-	-	-	621.184	131.117773
POS00923	8210868.496	6053808.813	-0.43969	0.314558	0.6243	0.96118	Cytosine	24.276971	-	-	-	620.851	112.111974
POS00926	16970407.35	7201324.987	-1.23669	0.375248	0.6505	1.42985	5-Hydroxypyrazinamide	5.2234639	-	-	-	620.845	140.120103
POS00928	79471857.53	61112544.61	-0.37898	0.847886	0.9313	1.90598	6-Thiourate	1.2482792	-	-	-	620.675	185.183407
POS00934	681893.3667	558454.961	-0.28811	0.055714	0.3276	0.28385	Calcium L-aspartate	-16.550773	-	-	-	620.5265	172.169244
POS00936	4453728.637	1377172.948	-1.6933	0.083777	0.3808	1.37088	Quite	-22.457323	-	-	-	620.349	193.169561
POS00938	3220283.828	4023108.563	0.321123	0.801516	0.9096	0.19961	L-Serine O-sulfate	-9.1385044	-	-	-	620.214	186.161385
POS00939	1994468.934	2052140.616	0.041125	0.935516	0.9711	0.03579	Didecyldimethylammonium chloride	8.0852325	-	-	-	620.214	363.086404

POS00941	5663153.086	5326794.237	-0.08834	0.903224	0.9553	0.14483	Methamidophos	-5.583809	-	-	-	620.2045	142.135689
POS00959	5205296.624	2848271.763	-0.86989	0.045882	0.3134	1.1924	L-Hexahydro-3-imino-1,2,4-oxadiazepine-3-carboxylic acid	10.53518	-	-	-	619.394	160.152253
POS00967	727358.9648	1405801.989	0.950654	0.193991	0.5197	0.58401	Pelargonidin 3-O-(6-O-malonyl-beta-D-glucoside)	-9.2246075	-	-	-	618.252	520.434085
POS00980	28909.2634	49061.26132	0.763052	0.101121	0.4075	0.1146	Carboplatin	-16.499513	-	-	-	617.486	372.255151
POS00993	877337.9924	845703.0598	-0.05298	0.874159	0.9444	0.00976	Bromoxynil	3.5145176	-	-	-	615.634	277.92105
POS00994	1463422.557	1738018.199	0.248097	0.310067	0.6214	0.23527	3-(Phosphoacetylamido)-L-alanine	8.788612	-	-	-	615.6055	243.133205
POS01019	395252.1682	388514.5379	-0.0248	0.860811	0.9368	0.00797	3-oxobrimonidine	15.442924	-	-	-	613.316	311.162066
POS01033	763634.5552	937486.369	0.295915	0.294473	0.6086	0.23968	Violacene	-0.9876213	-	-	-	612.303	355.933126
POS01047	794909.3348	705783.5971	-0.17156	0.41605	0.679	0.1921	Adenylylselete	-0.9398356	-	-	-	609.511	475.186231
POS01089	3323836.846	3020411.429	-0.1381	0.661428	0.8332	0.21855	Bromoxynil	3.7684858	-	-	-	601.039	277.92112
POS01098	750187.5911	639235.5469	-0.2309	0.210988	0.5344	0.24358	4-Chloromethandienone	-23.589269	-	-	-	598.559	335.879377
POS01126	1558272.031	1046931.577	-0.57378	0.264057	0.5803	0.44363	9-Chloro-11beta,17beta-dihydroxy-17-methylandrost-4-en-3-one	11.229488	-	-	-	592.348	353.906739
POS01136	172381.4397	88107.95453	-0.96826	0.011537	0.2016	0.27235	Triphosphate	1.418502	-	-	-	591.0775	253.922935
POS01140	826698.1913	644773.9741	-0.35857	0.102938	0.4094	0.33147	Phosfolan	-11.112482	-	-	-	590.213	256.29914
POS01142	1708209.531	1369934.643	-0.31838	0.030023	0.2685	0.46921	3-Dehydroquite	-21.346857	-	-	-	590.098	191.153917
POS01146	2463671.041	2276143.572	-0.11422	0.245908	0.564	0.21445	Cryptocyanin	14.694545	-	-	-	589.6775	481.398236
POS01148	117704.6243	87321.48509	-0.43076	0.011843	0.2036	0.15386	Chlorquinox	-16.27029	-	-	-	589.2735	268.929817
POS01149	89100.35768	70337.10505	-0.34115	0.149837	0.4753	0.10675	3,5-Dibromo-4-hydroxybenzamide	-6.7136698	-	-	-	589.108	295.933397
POS01155	88460.90747	61522.6179	-0.52392	0.021188	0.2424	0.15088	Ioxynil	-5.8916115	-	-	-	588.8215	371.918891
POS01156	1240287.361	1045629.378	-0.2463	0.057977	0.3334	0.34244	N-(2,3-Dihydroxybenzoyl)-L-serine	6.4351752	-	-	-	588.7615	242.206229
POS01158	8446921.389	6935637.158	-0.2844	0.015506	0.2226	1.01823	Cyazine	19.811771	-	-	-	588.734	241.704745
POS01185	105022.5329	86079.35954	-0.28696	0.1395	0.4626	0.09578	5-Hydroxyxanthotoxin	19.398163	-	-	-	585.508	233.200681
POS01187	1106877.032	648787.9389	-0.77068	0.015938	0.2226	0.56218	4-Bromophenylacetate	18.65863	-	-	-	585.182	216.055289

POS01198	214111.4065	166389.5112	-0.3638	0.181507	0.5069	0.13638	Nicotinurate	8.9539284	-	-	-	583.44	181.16959
POS01200	1547437.218	1582861.589	0.032654	0.976442	0.9886	0.00874	4-Pyridoxate	4.1795161	-	-	-	583.091	184.169442
POS01210	1730403.225	1662259.181	-0.05796	0.685688	0.8454	0.14217	Violacene	-1.1228301	-	-	-	582.285	355.933078
POS01217	106975.6401	96317.90621	-0.15141	0.178507	0.5048	0.06851	Butefine hydrochloride	9.226062	-	-	-	581.503	354.938642
POS01240	1149760.168	787422.6851	-0.54612	0.091055	0.3913	0.47427	(1S,3R)-3-(2,2-Dichloroethenyl)-2,2-dimethylcyclopropanecarb	-18.300271	-	-	-	580.477	210.073251
POS01241	11622694.94	12326764.77	0.08485	0.756743	0.8869	0.34752	Cyanidin	3.559275	-	-	-	580.441	288.252499
POS01244	428034.5745	305353.7353	-0.48725	0.033587	0.2804	0.30476	Polixetonium chloride	14.502323	-	-	-	580.122	326.207993
POS01245	1870989.524	2006315.961	0.100747	0.668343	0.8368	0.19083	Dihydrokaempferol	-12.7142	-	-	-	580.106	289.255812
POS01263	8113329.713	4241031.518	-0.93588	0.386127	0.6583	1.13731	Calcium formate	-18.232702	-	-	-	578.2345	131.117804
POS01272	163648.9836	135487.2506	-0.27245	0.355776	0.6395	0.09405	Salvarsan	-16.632645	-	-	-	576.545	440.000975
POS01278	2975634.492	1973551.672	-0.5924	0.104626	0.4116	0.80864	Monobasic calcium phosphate	2.490699	-	-	-	576.216	235.06036
POS01283	56695669.8	52786993.88	-0.10306	0.591417	0.7918	0.84186	Bromobenzene	-18.227198	-	-	-	576.16	158.012315
POS01311	1128521.168	1118197.023	-0.01326	0.963613	0.983	0.00838	3-Methyleneoxindole	-1.4600626	-	-	-	574.448	146.165065
POS01313	805790.2272	755922.9407	-0.09217	0.535773	0.7591	0.09376	Sulfluramid	-16.565122	-	-	-	574.4015	528.196544
POS01321	3411992.939	2901676.772	-0.23373	0.49929	0.7362	0.38959	cis-Aconitate	18.979593	-	-	-	573.806	175.118781
POS01333	1379108.776	1249117.613	-0.14283	0.778322	0.8983	0.13102	Nornitrogen mustard	8.9385357	-	-	-	572.814	143.035546
POS01348	3954588.77	2087678.32	-0.92163	0.155803	0.4841	0.85056	Lamotrigine	2.2898965	-	-	-	569.9005	257.098863
POS01404	4848229.982	1726308.629	-1.48977	0.076758	0.3704	1.44308	Calcium formate	-18.202699	-	-	-	532.867	131.117808
POS01405	629144.7284	550113.0962	-0.19366	0.534606	0.7588	0.11117	Nornitrogen mustard	8.856679	-	-	-	532.004	143.035534
POS01419	1450442.663	1147078.723	-0.33853	0.033851	0.2807	0.46346	Monobasic calcium phosphate	2.4798022	-	-	-	526.802	235.060357
POS01427	553159.3454	607819.315	0.135947	0.502131	0.7383	0.11675	3-Methyleneoxindole	-1.3957891	-	-	-	523.752	146.165074
POS01435	1821759.026	1636177.868	-0.155	0.721008	0.8658	0.2121	Lamotrigine	2.2763897	-	-	-	522.577	257.09886
POS01451	5787828.529	5121622.465	-0.17642	0.799345	0.9083	0.11835	cis-Aconitate	19.061898	-	-	-	519.8195	175.118795

POS01485	757408.1313	403369.735	-0.90897	0.026804	0.2585	0.49325	Nornitrogen mustard	8.9664587	-	-	-	490.388	143.03555
POS01489	960720.3874	311181.3055	-1.62636	0.29368	0.6082	0.53166	N-Methylethanolamine phosphate	13.449634	-	-	-	489.405	156.098962
POS01503	2039702.243	988900.9411	-1.04446	0.000492	0.0721	1.00269	Calcium formate	-18.145313	-	-	-	486.532	131.117816
POS01530	519032.6346	745035.7312	0.521484	0.163109	0.4904	0.33852	3-Methyleneoxindole	-1.4201268	-	-	-	482.6175	146.16507
POS01537	952746.8364	691508.8622	-0.46235	0.15718	0.4856	0.36871	Lamotrigine	2.2296927	-	-	-	480.916	257.098848
POS01559	6001674.174	3709183.823	-0.69426	0.142931	0.4676	1.03496	Cyazine	20.044871	-	-	-	472.784	241.704801
POS01566	2718972.526	1166547.417	-1.22082	0.008578	0.1899	1.12408	Calcium formate	-18.274652	-	-	-	468.962	131.117799
POS01592	836140.3624	680578.2648	-0.29698	0.031292	0.2723	0.3107	Lamotrigine	2.0297416	-	-	-	457.693	257.098796
POS01596	1030322.791	745183.9531	-0.46743	0.113089	0.4262	0.37733	Violacene	-1.2241182	-	-	-	455.036	355.933042
POS01629	713103.051	796814.1112	0.160133	0.542731	0.7639	0.13656	3-Methyleneoxindole	-1.495943	-	-	-	440.267	146.165059
POS01636	2359168.62	1299740.218	-0.86006	0.032958	0.2781	0.90412	Calcium formate	-18.230362	-	-	-	438.427	131.117805
POS01639	748125.7201	685505.9736	-0.12611	0.647311	0.8267	0.15195	Lamotrigine	2.1109042	-	-	-	437.613	257.098817
POS01670	5800336.829	4252696.793	-0.44776	0.002769	0.1143	1.16347	Paramethadione	-8.8067604	-	-	-	424.2485	158.172992
POS01676	119271.5701	103817.4917	-0.2002	0.511121	0.7452	0.05628	erythro-6,8-Dotriacontanediol	-2.388974	-	-	-	423.5575	483.871323
POS01679	51289.34798	54426.57786	0.085652	0.774241	0.896	0.01929	Octachlorocamphene	20.664626	-	-	-	423.191	412.810286
POS01689	460976.3626	436437.8909	-0.07892	0.689709	0.8466	0.05084	Tiracizine	4.9760744	-	-	-	422.814	404.911286
POS01701	348364.7875	310512.6894	-0.16595	0.220669	0.5419	0.12352	1,2,3,5-Tetrachloro-4-methoxybenzene	-9.6749845	-	-	-	422.4755	246.922897
POS01703	63444.88154	58852.69607	-0.1084	0.574292	0.7821	0.03312	Tetrachloro-cis,cis-mucote	4.8567315	-	-	-	422.469	280.898336
POS01716	372696.7134	338016.3734	-0.14091	0.46598	0.7151	0.09104	Ziram	5.2123764	-	-	-	422.448	306.850771
POS01722	152081.5451	140584.7963	-0.1134	0.413853	0.6778	0.06161	Dimefline hydrochloride	2.1865628	-	-	-	422.4435	360.854663
POS01730	86266.21815	82859.56631	-0.05813	0.717364	0.8633	0.01285	Trisalicylate-choline	6.341946	-	-	-	422.439	540.8247
POS01736	180533.4111	153073.1992	-0.23804	0.081495	0.378	0.1164	Haloperidol	-5.2536118	-	-	-	422.4355	376.869502
POS01751	1170303.523	1443123.221	0.302312	0.321336	0.6288	0.30818	1,7-diphospho-1-epi-valienol	21.066155	-	-	-	422.269	337.141358

POS01757	588901.0607	518509.0721	-0.18366	0.137297	0.4604	0.19054	Chlorpromazine	21.733443	-	-	-	422.243	319.878407
POS01759	26971.76546	26162.38997	-0.04396	0.886923	0.9486	0.00649	N-Acetyl-leu-leu-leu-tyr-amide	-13.720481	-	-	-	422.183	562.71297
POS01760	65496.91329	70105.79464	0.098107	0.580207	0.7858	0.03245	3b,8b-Dihydroxy-6b-(3-chloro-2-hydroxy-2-methylbutanoyloxy)	-10.666105	-	-	-	422.155	417.89583
POS01774	191290.2008	177008.6567	-0.11194	0.445388	0.7005	0.06225	Chlorphonium chloride	7.4813503	-	-	-	422.1105	398.800353
POS01777	153360.0841	131653.0476	-0.22018	0.156945	0.4851	0.10306	Gravacridonolchlorine	23.054976	-	-	-	422.0995	376.818941
POS01779	94608.08937	88228.65638	-0.10072	0.685426	0.8454	0.03156	Ochratoxin A	-6.5917801	-	-	-	422.0915	404.817615
POS01780	56374.36435	58813.65555	0.061112	0.7498	0.8827	0.02328	Triacontan-1-ol	16.331233	-	-	-	422.0915	439.827143
POS01784	30236.1491	37116.37285	0.295781	0.173008	0.4988	0.06021	Costatone	-20.788558	-	-	-	422.081	395.914167
POS01789	432914.4885	376738.3093	-0.20052	0.323656	0.6304	0.13336	3-Methyleneoxindole	-1.4326198	-	-	-	420.7685	146.165069
POS01790	227867.4787	233554.9017	0.035567	0.816712	0.9165	0.02425	Azaserine	4.5352251	-	-	-	419.547	174.134862
POS01792	318463.8552	221168.7612	-0.52598	0.00083	0.0732	0.30709	Nornitrogen mustard	8.7932341	-	-	-	418.0935	143.035525
POS01805	534452.242	494879.9498	-0.11098	0.517041	0.7485	0.08369	Lamotrigine	2.1081404	-	-	-	414.48	257.098816
POS01816	363260.6064	236110.8564	-0.62154	0.000578	0.0721	0.34653	Glyphosate-isopropylammonium	0.0121391	-	-	-	411.6785	229.190579
POS01820	568270.0488	430560.605	-0.40036	0.250214	0.568	0.25189	N-(2R-Hydroxyhexadecanoyl)-2S-amino-9-methyl	12.557742	-	-	-	411.244	566.925183
POS01839	891418.0838	863819.5046	-0.04537	0.766953	0.8917	0.06157	Violacene	-1.193704	-	-	-	409.414	355.933053
POS01867	395898.4056	264748.9121	-0.58051	0.002943	0.117	0.34614	Nornitrogen mustard	8.8173408	-	-	-	405.411	143.035529
POS01878	851097.1698	733527.6072	-0.21447	0.059602	0.3364	0.26053	Calcium oxalate	-17.257966	-	-	-	401.1	129.102066
POS01879	2148688.519	1182266.153	-0.8619	0.002696	0.1131	0.92408	Calcium formate	-18.272055	-	-	-	400.359	131.117799
POS01890	910655.0819	969297.2013	0.090034	0.725722	0.8684	0.13517	1,7-diphospho-1-epi-valienol	21.015493	-	-	-	392.318	337.14134
POS01894	54026.31457	49325.70525	-0.13132	0.380705	0.6544	0.03686	PC(P-16:0/P-16:0)	0.1398289	-	-	-	392.067	703.062375
POS01899	1166207.768	589175.8491	-0.98505	0.172976	0.4988	0.47717	Lamotrigine	2.1794174	-	-	-	392.059	257.098835
POS01900	175461.2956	162244.4551	-0.11298	0.410399	0.6752	0.05734	Cer(d18:1/22:1(13Z))	14.080722	-	-	-	392.055	621.060307
POS01912	835454.0656	750199.0159	-0.15529	0.19261	0.519	0.181	Didecyldimethylammonium chloride	12.834907	-	-	-	392.0355	363.088124

POS01914	59435.25753	56358.57075	-0.07668	0.689783	0.8466	0.01302	alpha-D-Ribose 1-methylphosphote 5-triphosphate	-18.659673	-	-	-	392.035	469.075242
POS01916	262695.309	223448.9802	-0.23344	0.072143	0.3613	0.15024	Monobasic calcium phosphate	2.6945022	-	-	-	392.03	235.060407
POS01919	159091.6844	159684.7422	0.005368	0.9639	0.983	0.0015	8-Br-cGMP	8.4568321	-	-	-	392.0285	425.112263
POS01930	800554.1976	783011.6263	-0.03197	0.798078	0.908	0.03477	Dibromobisphenol A	0.3928625	-	-	-	392.0255	387.085928
POS01936	108248.4584	112200.5176	0.051733	0.909869	0.9581	0.02626	(1S,3R)-3-(2,2-Dichloroethenyl)-2,2-dimethylcyclopropanecarb	-17.318251	-	-	-	392.024	210.073456
POS01962	36914.68327	41962.87982	0.184919	0.435909	0.6938	0.04134	Sulfluramid	-16.511405	-	-	-	391.959	528.196572
POS01967	56876.08886	60722.69397	0.094414	0.539661	0.7617	0.02836	alpha-D-Ribose 1-methylphosphote 5-triphosphate	10.915038	-	-	-	391.782	469.089086
POS01972	859609.4632	967467.6474	0.170532	0.552617	0.7706	0.17905	Violacene	-1.1514776	-	-	-	391.0945	355.933068
POS01977	21465.60537	23353.61691	0.121619	0.591275	0.7918	0.02039	2-Hydroxy-6-oxono-2,4-diene-1,9-dioate	-19.55625	-	-	-	389.4885	215.175188
POS02011	123235.7328	118456.5539	-0.05706	0.806138	0.9116	0.03532	3-hydroxy-2-isobutyrate	14.974815	-	-	-	378.159	104.10682
POS02017	283978.0712	212381.8601	-0.41912	0.051506	0.3205	0.20763	D-Erythritol 4-phosphate	-22.592628	-	-	-	374.193	203.102411
POS02018	755168.8191	635985.9105	-0.2478	0.14149	0.4653	0.23951	Lamotrigine	2.1788241	-	-	-	374.0415	257.098835
POS02022	966413.5695	714098.2573	-0.43652	0.322076	0.629	0.30124	1,7-diphospho-1-epi-valienol	20.889698	-	-	-	370.264	337.141298
POS02029	66371.81881	61857.80573	-0.10162	0.554811	0.7716	0.02217	2-Oxoglutaramate	-22.616064	-	-	-	368.225	146.117395
POS02034	145931.682	140277.9713	-0.057	0.761942	0.8889	0.02531	5a,11a-Dehydrochlortetracycline	24.274233	-	-	-	367.324	477.882552
POS02039	1050321.201	950235.7372	-0.14447	0.838131	0.9265	0.04614	5-FU	-23.24485	-	-	-	365.659	131.081453
POS02045	579338.9402	653252.5476	0.173233	0.625427	0.8135	0.12236	N-(2R-Hydroxyhexadecanoyl)-2S-amino-9-methyl	12.649488	-	-	-	364.6975	566.925235
POS02055	122171.0467	69782.24995	-0.80797	0.031637	0.2738	0.19003	(1S,3R)-3-(2,2-Dichloroethenyl)-2,2-dimethylcyclopropanecarb	-7.2305712	-	-	-	364.058	210.075565
POS02060	2794762	2541661.956	-0.13695	0.620238	0.8112	0.21811	Violacene	-1.3816633	-	-	-	363.471	355.932986
POS02068	52006727.11	38613626.79	-0.42959	0.574326	0.7821	1.08562	Trifluoromethyl-bismethyl ketone	16.640107	-	-	-	362.352	141.113308
POS02070	1490444.769	1242771.376	-0.26218	0.237718	0.5574	0.27689	Dimefox	-8.8450701	-	-	-	362.337	155.128913
POS02076	14643988.51	9778792.614	-0.58258	0.022757	0.2473	1.94132	Cyazine	19.861931	-	-	-	361.67	241.704757
POS02079	6105992.842	3026476.041	-1.01259	0.004378	0.1402	1.65176	Calcium formate	-18.433761	-	-	-	361.28	131.117778

POS02083	859990.1973	697924.1898	-0.30125	0.441596	0.6978	0.19677	Nornitrogen mustard	8.8779523	-	-	-	360.229	143.035538
POS02103	125228.3111	120395.7975	-0.05678	0.876378	0.9452	0.03456	Monobasic calcium phosphate	2.710089	-	-	-	359.616	235.060411
POS02110	1411737.495	1491914.663	0.079693	0.842386	0.928	0.09409	Didecyldimethylammonium chloride	13.281277	-	-	-	359.557	363.088285
POS02121	129386.0623	134066.5576	0.051267	0.890109	0.9498	0.0158	Phosphoguanidinoacetate	15.967162	-	-	-	359.531	198.096924
POS02122	39588.49883	43543.39	0.137372	0.807986	0.9121	0.02025	dCDP	-12.177007	-	-	-	359.521	388.179562
POS02124	163944.8491	151744.8701	-0.11156	0.766649	0.8917	0.03489	Methylphosphote	-14.492606	-	-	-	359.519	97.028285
POS02131	408141.3802	426072.5276	0.06203	0.848004	0.9313	0.04329	Dibromobisphenol A	-0.0360916	-	-	-	359.497	387.085763
POS02133	289855.4645	288420.6882	-0.00716	0.981799	0.9913	0.00294	alpha-D-Ribose 1-methylphosphote 5-triphosphate	10.495839	-	-	-	359.4965	469.088889
POS02136	37643.03057	41639.52747	0.145571	0.778809	0.8984	0.02034	8-Br-cGMP	7.7054825	-	-	-	359.4935	425.111945
POS02162	783292.235	936875.3504	0.258306	0.463184	0.7133	0.14005	Lamotrigine	2.0523763	-	-	-	359.121	257.098802
POS02167	103197.6599	88179.01421	-0.2269	0.463432	0.7135	0.05177	4-Hydroxycyclophosphamid	22.638844	-	-	-	357.605	278.098949
POS02169	1819756.133	1828204.297	0.006682	0.985856	0.9927	0.05246	L-Hexahydro-3-imino-1,2,4-oxadiazepine-3-carboxylic acid	11.238388	-	-	-	357.306	160.152365
POS02172	1955394.542	1409448.323	-0.47233	0.087264	0.3863	0.56164	Calcium oxalate	-17.501533	-	-	-	356.982	129.102035
POS02176	1011233.413	789777.1813	-0.3566	0.064798	0.3459	0.37639	2-Nitrofuran	-12.979679	-	-	-	356.5825	114.077309
POS02191	313024.2148	387846.6887	0.309212	0.370949	0.6495	0.1642	Triphosphate	1.4845826	-	-	-	353.6685	253.922952
POS02197	96874.93957	68159.1465	-0.50722	0.122033	0.4395	0.11955	Demeclocycline	-21.77352	-	-	-	352.9935	465.850155
POS02200	680318.5206	839853.346	0.303927	0.507399	0.7434	0.19482	3-Methyleneoxindole	-1.4576709	-	-	-	352.927	146.165065
POS02208	185321.173	219895.9796	0.246794	0.515586	0.7477	0.10011	3b-Hydroxy-6b-(3-chloro-2-hydroxy-2-methylbutanoyloxy)	-11.214686	-	-	-	352.436	401.896781
POS02224	235844.9946	239978.3698	0.025065	0.832083	0.9235	0.01905	Lolicine A	-8.9514352	-	-	-	351.8685	604.832971
POS02247	2507391.775	2201352.287	-0.1878	0.114037	0.4272	0.36546	Radon-222	2.4251519	-	-	-	350.689	223.007815
POS02251	91955.58504	99272.79929	0.110461	0.476733	0.722	0.04799	Indivir	18.695641	-	-	-	350.536	614.808252
POS02252	206305.7303	181263.4806	-0.1867	0.163531	0.4913	0.10782	Leucomycin V	24.973432	-	-	-	350.532	702.866704
POS02254	140587.0388	113679.9062	-0.30649	0.206952	0.5314	0.11599	Oleandomycin 2'-O-phosphate	-10.314352	-	-	-	350.528	768.837557

POS02259	107039.2687	99240.55109	-0.10914	0.721653	0.8663	0.02245	Clethodim	11.2254	-	-	-	350.523	360.922517
POS02262	70837.95946	89935.30911	0.344365	0.046831	0.314	0.12197	Rifamycin	-18.408208	-	-	-	350.483	698.763032
POS02269	149710.2186	153123.2713	0.032521	0.833868	0.9241	0.01727	Glycylserylprolylmethionylphenylalanylvalinamide	12.271167	-	-	-	350.192	636.790078
POS02271	388052.224	382934.7598	-0.01915	0.841792	0.9279	0.01751	Flumiclorac pentyl	-16.543874	-	-	-	350.19	424.862664
POS02275	138223.8145	117916.356	-0.22924	0.225884	0.5473	0.08407	33-Deoxy-33-hydroperoxyfurohyperfori[illegible]	8.0027748	-	-	-	350.1795	569.795628
POS02282	315471.1354	277025.9339	-0.18749	0.224682	0.5464	0.12537	sn-Glycerol 3-phosphate	-16.91092	-	-	-	350.14	173.078067
POS02287	114188.8435	124381.844	0.123354	0.423951	0.6843	0.06258	Lolitrem K	3.0024506	-	-	-	350.0105	602.781283
POS02290	321761.1176	249815.2985	-0.36513	0.072389	0.3616	0.20888	3-(3,5-Diiodo-4-hydroxyphenyl)lactate	-9.08741	-	-	-	349.901	434.969733
POS02292	127511.729	150209.4676	0.236346	0.228697	0.5501	0.11036	Arnamiol	-19.762426	-	-	-	349.863	451.950365
POS02293	117971.436	121957.0224	0.047935	0.718236	0.864	0.02475	Aquifoliunine EIII	-14.816046	-	-	-	349.858	764.734961
POS02294	209391.8445	199586.8047	-0.06919	0.735223	0.8745	0.03467	Dichlormid	-14.291548	-	-	-	349.855	209.089303
POS02297	106779.418	114197.8274	0.096902	0.570653	0.7802	0.04805	Cholesteryl-beta-D-glucoside	-3.6038284	-	-	-	349.852	549.799399
POS02306	143938.8295	150731.8037	0.066528	0.76484	0.8911	0.0217	Veratridine	8.3128357	-	-	-	349.8125	674.803078
POS02311	508987.2598	409440.1815	-0.31398	0.050099	0.3189	0.25143	Pavoninin 1	2.4468385	-	-	-	349.789	662.874596
POS02325	112295.2634	109661.8503	-0.03424	0.886933	0.9486	0.01909	PC(14:1(9Z)/15:0)	-18.781586	-	-	-	349.507	690.937518
POS02330	475939.659	409255.1739	-0.21778	0.036408	0.2865	0.20776	Terodiline hydrochloride	-13.699615	-	-	-	349.502	318.898922
POS02331	123568.9676	91460.53525	-0.4341	0.196427	0.5222	0.11087	Sodium hexafluorosilicate	2.0002668	-	-	-	349.4965	189.063153
POS02332	62723.21433	63258.75515	0.012266	0.956252	0.9806	0.01099	Dihydrocaffeoyl-CoA	-0.7544598	-	-	-	349.4865	932.698674
POS02334	71436.82216	102562.0986	0.521758	0.035625	0.285	0.14866	Discodermolide	-0.3971864	-	-	-	349.469	594.798741
POS02340	64403.01186	55183.53448	-0.22289	0.339929	0.6326	0.05188	S-(1,2-Dichlorovinyl)-L-cysteine	7.2680399	-	-	-	349.461	217.094447
POS02342	73795.22224	88017.56266	0.254264	0.077048	0.3708	0.09922	Dihydromethanophezine	-22.704936	-	-	-	349.439	541.816597
POS02343	695754.2319	565174.5519	-0.29988	0.0032	0.1215	0.3433	Deamino-alpha-keto-demethylphosphinothricin	14.04576	-	-	-	349.432	167.078709
POS02344	54987.88282	59384.42447	0.110971	0.671414	0.8378	0.02533	Rifamycin B	-9.408335	-	-	-	349.431	756.804866

POS02346	94439.3947	103290.8243	0.129251	0.549122	0.769	0.05026	3-(Methylthio)propanoyl-CoA	2.5672264	-	-	-	349.409	870.698509
POS02361	66632.12717	66022.92661	-0.01325	0.945519	0.9757	0.00056	Dauricine	20.796705	-	-	-	349.1695	625.78607
POS02363	864201.7601	846943.2836	-0.0291	0.777327	0.8978	0.03452	Indoxacarb	10.529287	-	-	-	349.168	528.847334
POS02364	48396.0923	63179.30076	0.384561	0.099486	0.4055	0.10118	21,22-Diprenylpaxilline	12.747417	-	-	-	349.1645	572.803865
POS02365	143912.6344	143154.7808	-0.00762	0.978908	0.9898	0.00234	3,5-Dibromo-4-hydroxybenzamide	-6.6681859	-	-	-	349.161	295.93341
POS02368	289592.5971	246117.2434	-0.23468	0.563188	0.7763	0.08438	Tetramethylrosamine	14.041222	-	-	-	349.137	392.92598
POS02372	1691785.807	1508748.366	-0.16519	0.105075	0.4121	0.29306	Cryptocyanin	14.305859	-	-	-	349.1175	481.398049
POS02377	1064532.802	912593.9562	-0.22218	0.045464	0.3121	0.31748	Armillaramide	-22.117961	-	-	-	348.9795	556.910981
POS02381	334526.4179	328571.9044	-0.02591	0.781932	0.8995	0.01402	1,4-Dihydroxy-2-phthoyl-CoA	-24.576181	-	-	-	348.846	954.681538
POS02389	283910.9018	282061.7162	-0.00943	0.939543	0.9726	0.00386	11-[(2R)-3-[2-amino-3-methyl-4-(2-methyl-1,3-thiazol-4-yl)but-3-en-1	21.06018	-	-	-	348.805	605.790013
POS02398	325178.6157	280058.1273	-0.21551	0.210411	0.5339	0.12509	Demethyllactenocin	-11.825463	-	-	-	348.481	744.87698
POS02402	60799.70523	64233.35505	0.079258	0.724111	0.8676	0.02602	DPDPE	-9.5930819	-	-	-	348.323	646.791081
POS02415	45079.57556	48680.57635	0.110872	0.757994	0.8874	0.02942	Trisalicylate-choline	12.466844	-	-	-	347.986	540.828006
POS02418	122448.7907	164261.7754	0.423818	0.090004	0.3896	0.16151	Doronine	-5.4473248	-	-	-	347.868	460.922571
POS02421	68138.93462	47767.0588	-0.51246	0.231375	0.553	0.08881	Santiaguine	-19.064294	-	-	-	347.8605	593.809275
POS02434	64252.79409	70622.56363	0.13637	0.622607	0.8124	0.03473	Cimicifugoside	-20.30355	-	-	-	347.586	675.811675
POS02436	138955.4143	115708.8848	-0.26412	0.161388	0.4889	0.09289	DG(14:0/20:5(5Z,8Z,11Z,14Z,17Z)/0:0)	-1.061133	-	-	-	347.5465	587.891854
POS02442	42197.7279	41817.99745	-0.01304	0.978049	0.9896	0.00112	15,15'-Dihydroxy-beta-carotene	8.9646876	-	-	-	347.531	571.899694
POS02458	84602.68426	51963.05693	-0.70322	0.012768	0.21	0.15028	Glyburide	-2.884104	-	-	-	347.213	495.009352
POS02460	129563.2956	152383.3942	0.234049	0.315857	0.625	0.09492	1,1'-(1,4-Dihydro-4-nonyl-3,5-pyridinediyl)bis[1-decanone]	11.184046	-	-	-	347.2125	516.866646
POS02463	69640.79875	88160.69464	0.340203	0.316814	0.6253	0.08634	Acetrizoic acid	-3.2533394	-	-	-	347.21	557.867765
POS02465	202136.0426	207815.3829	0.039976	0.820032	0.9178	0.03317	23-trans-p-Coumaroyloxytormentic acid	3.0932988	-	-	-	347.204	651.85059
POS02477	85146.05852	60897.8578	-0.48355	0.159242	0.4871	0.11864	Epomusenin A	-11.341496	-	-	-	347.157	559.919038

POS02482	914204.8388	762436.0173	-0.2619	0.084174	0.3821	0.27041	Erythromycin C	11.813814	-	-	-	347.0255	720.915981
POS02486	795031.8592	711560.2645	-0.16003	0.13449	0.4553	0.19315	9,10-12,13-diepoxy-octadecanoate	-17.881158	-	-	-	346.8955	626.881085
POS02488	577081.0386	464701.773	-0.31247	0.216587	0.5382	0.19219	Iopodic acid	17.754112	-	-	-	346.893	598.975093
POS02489	272303.2598	265798.3551	-0.03488	0.813028	0.9151	0.00321	Ginsenoside F1	-13.94068	-	-	-	346.893	639.87047
POS02492	455961.9217	367314.2719	-0.3119	0.384521	0.6575	0.17993	DG(14:0/0:0/22:5n3)	-5.9134796	-	-	-	346.891	615.95564
POS02502	31583.1249	39204.02044	0.311848	0.623485	0.8127	0.03318	Aquifoliunine EI	-16.623215	-	-	-	346.8695	868.83795
POS02504	212656.2715	206449.1835	-0.04274	0.88997	0.9497	0.00348	Kad 1229	17.45308	-	-	-	346.868	669.894451
POS02506	56777.8917	59642.12164	0.071002	0.778312	0.8983	0.01512	6-(2-{6-[2,4-dihydroxy-3-(3-methylbut-2-en-1-yl)benzoyl]-5-(2,4-	-22.70512	-	-	-	346.868	827.836503
POS02508	314452.5372	395437.4413	0.330607	0.348214	0.6355	0.18914	PE(14:1(9Z)/P-16:0)	17.352066	-	-	-	346.8585	646.909184
POS02511	201397.5668	261340.0984	0.375882	0.168785	0.4949	0.1732	PS(14:0/14:0)	-12.391517	-	-	-	346.847	680.871852
POS02518	219615.1907	296466.3117	0.43289	0.239594	0.5591	0.18149	PS(14:1(9Z)/18:3(9Z,12Z,15Z))	-2.024124	-	-	-	346.572	728.910903
POS02522	172627.5286	151189.5142	-0.1913	0.366596	0.6461	0.06265	Cer(d18:1/18:1(11Z))	-23.337217	-	-	-	346.558	564.932116
POS02524	61472.39868	54008.96755	-0.18674	0.675358	0.8398	0.031	PS(14:0/15:0)	-5.3039293	-	-	-	346.558	694.903596
POS02527	136437.7372	100137.0231	-0.44627	0.070279	0.357	0.14186	Bis(glutathionyl)spermine disulfide	14.703323	-	-	-	346.55	779.95953
POS02534	81524.03822	82279.24335	0.013303	0.972631	0.9873	0.00325	(3-{3,5,7-trihydroxy-4-[6,9,17,19,21-pentahydroxy-5,13-bis(3-	23.063812	-	-	-	346.4095	897.857961
POS02538	231169.5522	209427.7935	-0.1425	0.474857	0.7207	0.07376	Dermorphin	-5.3649841	-	-	-	346.259	803.875569
POS02544	3691407.644	2894925.281	-0.35064	0.039288	0.2959	0.69226	Antimony potassium tartrate	17.213635	-	-	-	346.253	668.891373
POS02546	80189.1048	90412.96274	0.173123	0.662334	0.8336	0.03245	Prephytoene diphosphate	7.1743694	-	-	-	346.253	723.923663
POS02550	162046.39	170521.5679	0.073547	0.758552	0.8874	0.02467	{6,7,8,11,12,13,21,22-octahydroxy-3,16-dioxo-2,17,20-	21.039251	-	-	-	346.2525	563.429109
POS02554	112966.037	122779.3385	0.120179	0.685692	0.8454	0.05326	R.g.-Keto III	16.453605	-	-	-	346.247	629.941025
POS02557	116677.4274	99475.7443	-0.23011	0.518134	0.7495	0.06352	Mometasone furoate	-16.679149	-	-	-	346.2265	522.42808
POS02564	557568.4472	576810.6028	0.048949	0.834507	0.9243	0.04843	Hovenidulcioside B2	13.241402	-	-	-	345.943	709.892463
POS02567	222558.465	206117.488	-0.11072	0.834769	0.9243	0.04428	PA(14:0/22:4(7Z,10Z,13Z,16Z))	5.8195199	-	-	-	345.938	697.958332

POS02568	852810.1226	856469.3043	0.006177	0.969295	0.9861	0.00358	Cethromycin	-18.521501	-	-	-	345.938	766.92509
POS02572	985378.9071	866724.47	-0.18511	0.339229	0.6326	0.17857	Donhexocin	13.798676	-	-	-	345.929	615.909461
POS02573	3475302.471	3041585.524	-0.19231	0.366862	0.6463	0.3846	Erioglaucine A	-7.9586023	-	-	-	345.927	750.894308
POS02574	498510.997	437953.4869	-0.18685	0.376215	0.6512	0.15187	Nostocyclopeptide A2	-22.323442	-	-	-	345.9265	791.894621
POS02577	1008537.989	986499.707	-0.03187	0.867226	0.9403	0.00243	20,21-Diprenylterpendole C	-3.2310155	-	-	-	345.9165	656.910857
POS02580	285043.1061	287114.6834	0.010447	0.972992	0.9873	0.00031	CerP(d18:1/18:0)	-8.6999518	-	-	-	345.776	646.935357
POS02581	146058.1984	85846.23565	-0.76672	0.068126	0.3534	0.18584	Corchorusoside E	21.085429	-	-	-	345.773	861.96093
POS02582	2729379.117	2489977.216	-0.13244	0.56424	0.7766	0.1859	PE(18:4(6Z,9Z,12Z,15Z)/P-16:0)	-2.3058091	-	-	-	345.772	696.955072
POS02590	1318738.879	1221091.05	-0.11099	0.615343	0.8087	0.13387	Lasonolide A	-7.5294263	-	-	-	345.6175	697.911729
POS02599	1326755.741	951035.0771	-0.48033	0.02975	0.2674	0.49135	Homotrypanothione	23.418297	-	-	-	345.4515	738.913357
POS02601	4330767.851	3380817.333	-0.35725	0.138375	0.4613	0.64802	Cephalomannine	-13.72511	-	-	-	345.3075	832.896459
POS02603	906854.1103	1026683.78	0.17905	0.378652	0.6529	0.21669	Periandrin V	-20.07075	-	-	-	345.306	779.914243
POS02605	180019.4346	150406.1359	-0.25929	0.285491	0.6008	0.10873	Glycerol 1-(9Z-octadecenoate) 2-hexadecanoate 3	1.2122293	-	-	-	345.304	862.419021
POS02606	2051363.744	1810309.919	-0.18035	0.181706	0.5071	0.32788	DG(15:0/20:4(5Z,8Z,11Z,14Z)/0:0)	-10.233121	-	-	-	345.3025	603.928707
POS02607	228232.6237	197845.706	-0.20613	0.595021	0.7937	0.0549	PS(14:0/18:4(6Z,9Z,12Z,15Z))	20.91183	-	-	-	345.3005	728.939499
POS02608	2424874.117	1998885.533	-0.27871	0.294618	0.6086	0.40111	PS(14:1(9Z)/22:6(4Z,7Z,10Z,13Z,16Z,19Z))	-16.71958	-	-	-	345.3	778.958069
POS02610	153866.6131	110947.9102	-0.4718	0.124995	0.4435	0.1502	DG(14:0/22:4(7Z,10Z,13Z,16Z)/0:0)	-0.6590587	-	-	-	345.294	617.96107
POS02611	150047.725	140989.0823	-0.08984	0.759303	0.8876	0.02078	PS(14:1(9Z)/20:5(5Z,8Z,11Z,14Z,17Z))	-0.7392254	-	-	-	345.294	752.945721
POS02612	2091273.739	1760212.667	-0.24863	0.297483	0.6109	0.3595	DG(9D3/9D3/0:0)	-11.131413	-	-	-	345.2925	645.934098
POS02618	2280748.164	1979534.371	-0.20435	0.286022	0.6016	0.31872	PA(15:0/20:3(5Z,8Z,11Z))	-18.000669	-	-	-	344.995	685.930947
POS02632	108206.8614	97516.40878	-0.15008	0.720782	0.8657	0.03724	Gypsogenin 3-O-rhamnosylglucuronide	-13.25297	-	-	-	344.683	793.945968
POS02633	1831027.543	1612116.643	-0.1837	0.385862	0.6583	0.23833	PS(15:0/18:4(6Z,9Z,12Z,15Z))	16.698032	-	-	-	344.6825	742.963666
POS02634	14083695.51	14427584.69	0.034804	0.864334	0.9385	0.09748	Galactosylceramide (d18:1/12:0)	-18.487361	-	-	-	344.68	644.930372

POS02640	2716267.022	2139230.897	-0.34453	0.064975	0.3461	0.58184	(3b,5a,25R)-3-Hydroxyspirostan-6-one 3-[2-acetylarabinosyl-(1 [illegible]	18.955111	-	-	-	344.674	767.933714
POS02641	482806.9691	394101.0464	-0.29288	0.325366	0.6315	0.13944	TG(10:0/16:0/a-25:0)[rac]	13.639271	-	-	-	344.673	850.438862
POS02644	293311.4105	253544.9734	-0.21019	0.220585	0.5419	0.12134	PE(14:0/P-18:1(11Z))	3.3359743	-	-	-	344.668	674.953325
POS02647	317025.0013	278497.7749	-0.18693	0.486812	0.7289	0.09994	Bis(glutathionyl)spermine	1.4714283	-	-	-	344.649	781.965026
POS02649	150138.5657	101251.8883	-0.56835	0.26597	0.5823	0.11755	Leucokinin I	12.679399	-	-	-	344.5155	892.944586
POS02651	358658.9258	224035.4341	-0.67889	0.074412	0.3656	0.26735	PA(14:1(9Z)/22:2(13Z,16Z))	-11.154454	-	-	-	344.5125	699.96248
POS02652	4086431.851	3011260.694	-0.44047	0.05629	0.3284	0.81063	DG(14:0/0:0/20:3n6)	19.171971	-	-	-	344.507	591.948606
POS02655	984550.124	928981.7306	-0.08381	0.793966	0.9052	0.09141	2-[4-(1,2-dihydroxyethyl)-5,11,12,13-tetrahydroxy-8-oxo-3,7 [illegible]	7.5417702	-	-	-	344.365	633.451046
POS02656	2610637.658	1868244.474	-0.48272	0.059056	0.336	0.66497	Licoricesaponin B2	-20.75054	-	-	-	344.365	809.93909
POS02657	2491761.579	1938454.391	-0.36226	0.242323	0.5616	0.48051	20-O-Methyl-19-chloroproansamitocin	-0.8534432	-	-	-	344.3645	493.011157
POS02673	451879.6961	494174.4253	0.129082	0.461102	0.7119	0.1053	PE(16:1(9Z)/16:1(9Z))	12.312375	-	-	-	344.04	688.955747
POS02674	319568.8285	333943.813	0.063479	0.783392	0.9004	0.03603	Digitin	16.068409	-	-	-	344.038	797.957982
POS02681	369696.1238	706692.339	0.934742	0.263059	0.58	0.41141	Triforine	19.042442	-	-	-	343.737	435.977359
POS02685	730534.7566	580750.3699	-0.33103	0.141351	0.4651	0.29256	2,3,4,5-Tetrachloro-4'-biphenylol	-13.538359	-	-	-	343.73	308.990507
POS02692	331851.0992	330291.4594	-0.0068	0.979454	0.9899	0.02348	PS(14:0/20:4(5Z,8Z,11Z,14Z))	-11.086346	-	-	-	343.429	756.957196
POS02698	685557.9876	495038.5281	-0.46974	0.400076	0.6679	0.23018	Tetrabromobisphenol A	1.0991437	-	-	-	343.276	544.878474
POS02707	783765.1941	536737.2287	-0.54621	0.211653	0.5346	0.3127	3,4,5-trihydroxy-6-{2-[(E)-2-(3-hydroxy-5-{[3,4,5-trihydroxy-6 [illegible]	-15.833061	-	-	-	343.1145	745.647486
POS02710	1989471.038	3563426.934	0.84088	0.315066	0.6245	0.72107	DG(11D3/9D3/0:0)	2.6259802	-	-	-	343.113	673.997044
POS02714	1951368.058	1744387.517	-0.16177	0.466509	0.7151	0.24109	(E)-1-{3-[6-hydroxy-3-(3-hydroxyphenyl)-2-(4-hydroxyphenyl)-2,3 [illegible]	-8.054281	-	-	-	343.1035	891.941101
POS02719	151161.85	210264.822	0.476113	0.070961	0.3583	0.1946	PE(14:0/22:6(4Z,7Z,10Z,13Z,16Z,19Z))	-18.012967	-	-	-	342.9615	736.96422
POS02727	335354.0795	2545871.79	2.924403	0.059508	0.3364	1.2137	Chikusetsusaponin Ia	3.5513379	-	-	-	342.799	755.997258
POS02731	998550.2399	818904.8687	-0.28614	0.04707	0.314	0.29755	TG(15:0/20:1(11Z)/22:6(4Z,7Z,10Z,13Z,16Z,19Z))	-17.853273	-	-	-	342.798	920.460861
POS02745	8025227.074	8179404.337	0.027454	0.77286	0.8949	0.16609	DG(18:4(6Z,9Z,12Z,15Z)/22:6(4Z,7Z,10Z,13Z,16Z,19Z)/0:0)	-3.3699495	-	-	-	342.785	661.970349

POS02746	1322248.813	1185379.878	-0.15764	0.455132	0.7083	0.18939	Agathisflavone	6.3244776	-	-	-	342.6465	539.468582
POS02749	491055.8069	407031.8825	-0.27075	0.155188	0.4838	0.21088	Dimethylarsinous acid	-21.864022	-	-	-	342.494	123.002609
POS02750	528715.8869	715406.9577	0.436271	0.032838	0.2781	0.35719	Cer(d18:0/18:0)	23.342224	-	-	-	342.494	568.990234
POS02754	1006486.145	1042112.523	0.050184	0.681356	0.8431	0.07634	Fursultiamine hydrochloride	-1.4029783	-	-	-	342.489	436.010966
POS02757	8128093.057	8215431.854	0.015419	0.888399	0.949	0.10473	Patellamide A	19.408388	-	-	-	342.489	743.973196
POS02761	995015.695	939511.3745	-0.08281	0.496806	0.7349	0.10785	Pseudoargiopinin I	7.6490288	-	-	-	342.488	744.975767
POS02766	2522283.282	2258545.196	-0.15934	0.227905	0.5492	0.30938	alpha-Amanitin	-18.168376	-	-	-	342.487	919.96028
POS02771	13420630.25	12624235.33	-0.08826	0.339727	0.6326	0.46377	erythro-6,8-Hexatriacontanediol	-16.194346	-	-	-	342.486	539.970148
POS02774	2485248.886	2423764.507	-0.03614	0.736227	0.8747	0.06054	PC(14:0/16:1(9Z))	-0.7704536	-	-	-	342.4845	704.976534
POS02777	657830.0852	592040.64	-0.15202	0.4805	0.7238	0.12747	Adenosylhopane	8.4115179	-	-	-	342.483	662.972745
POS02782	884636.3526	1046551.17	0.242486	0.133583	0.454	0.29946	Spinosyn D	10.970311	-	-	-	342.4815	746.99756
POS02783	2320107.014	2280986.868	-0.02453	0.826366	0.9204	0.03454	Leucomycin A1	19.787999	-	-	-	342.4815	786.981129
POS02789	9855704.4	9489514.396	-0.05462	0.584531	0.7883	0.23145	Astragaloside III	0.6681251	-	-	-	342.481	785.978001
POS02790	432334.4077	701437.7272	0.698167	0.223286	0.545	0.285	PGP(16:0/16:1(9Z))	21.82054	-	-	-	342.481	801.969754
POS02793	5656137.809	5911440.582	0.063692	0.62019	0.8112	0.26288	Nodularin	7.1325338	-	-	-	342.4795	825.975861
POS02798	1466155.667	1492643.062	0.025831	0.860405	0.9366	0.05029	Chlorophyll b	0.6826003	-	-	-	342.479	908.480396
POS02802	508840.1511	578310.9308	0.184633	0.35965	0.6425	0.16536	Prenylated FMNH2	7.5192134	-	-	-	342.4785	527.487935
POS02806	376858.1652	550153.5106	0.545813	0.117527	0.432	0.31553	TG(20:4(5Z,8Z,11Z,14Z)/22:5(4Z,7Z,10Z,13Z,16Z)/20:4(5Z,8Z,11Z,14Z))	9.6074297	-	-	-	342.478	978.502568
POS02811	410094.3056	597526.488	0.543047	0.123309	0.441	0.33919	DG(14:0/0:0/20:1n9)	4.8582977	-	-	-	342.476	595.972167
POS02812	180769.9711	238265.9704	0.398418	0.2767	0.5932	0.14989	PA(10:0/i-24:0)	14.603773	-	-	-	342.476	677.974163
POS02816	386983.6641	418303.818	0.112278	0.677236	0.8404	0.10126	Oleoyl-CoA	0.3572573	-	-	-	342.4755	1032.98785
POS02817	2624333.466	2549203.897	-0.0419	0.62934	0.8159	0.09841	Azimilide	17.52379	-	-	-	342.475	458.968402
POS02819	53906157.27	52145426.32	-0.04791	0.645003	0.8256	0.43382	Beauvericin	23.747449	-	-	-	342.474	784.974693

POS02823	317906.1847	320622.2629	0.012274	0.940678	0.9732	0.01941	rasin B	7.2494635	-	-	-	342.472	764.022108
POS02826	66352914.48	63522691.9	-0.06289	0.515647	0.7477	0.68294	PE-NMe(14:0/18:2(9Z,12Z))	-3.2484713	-	-	-	342.4705	702.971996
POS02827	3736834.596	3984504.619	0.092584	0.505125	0.7415	0.27049	1-Hexadecanoyl-2-(9Z-octadecenoyl)-sn-glycero-3-phosphoserine	7.7561498	-	-	-	342.4705	763.019087
POS02830	2940889.499	3404539.753	0.211207	0.220802	0.5419	0.47895	Bayogenin 3-O-cellobioside	9.8995756	-	-	-	342.469	813.995625
POS02835	826584.2886	592092.5393	-0.48134	0.063638	0.3438	0.35781	Clocapramine dihydrochloride	-7.0784763	-	-	-	342.3325	554.997855
POS02843	29581818.29	30662496.61	0.051764	0.671478	0.8378	0.49484	CE(14:0)	-8.0188991	-	-	-	342.3275	598.011689
POS02847	71886892.76	75344694.13	0.067777	0.618011	0.8101	0.89826	PE-NMe2(18:4(6Z,9Z,12Z,15Z)/18:4(6Z,9Z,12Z,15Z))	0.4096078	-	-	-	342.3275	761.013588
POS02849	7968055.635	8081595.624	0.020412	0.857002	0.9351	0.12196	Pristimycin IA	17.614088	-	-	-	342.3275	867.980447
POS02853	10348549.65	10460793.93	0.015564	0.897217	0.9529	0.10648	Linoleoyl-CoA	11.50075	-	-	-	342.3275	1030.98342
POS02858	236911.6694	372036.3179	0.651094	0.049728	0.3188	0.30396	PA(10:0/a-25:0)	5.0462298	-	-	-	342.3225	691.994763
POS02862	826915.9106	1051704.626	0.346917	0.145412	0.4697	0.33724	TG(16:0/22:5(4Z,7Z,10Z,13Z,16Z)/o-18:0)	0.3241783	-	-	-	342.186	896.499567
POS02866	4502648.323	5098041.508	0.179169	0.171403	0.4973	0.55009	PC(14:0/P-16:0)	-3.346071	-	-	-	342.184	690.991268
POS02876	1727718.422	1826176.256	0.079958	0.614869	0.8087	0.14691	CDP-DG(a-13:0/a-17:0)	-16.564797	-	-	-	342.181	927.023937
POS02877	1069029.549	1335353.665	0.32092	0.177381	0.5035	0.36642	Crocin	21.366988	-	-	-	342.179	978.000152
POS02890	72391564.08	67929528.93	-0.09178	0.338556	0.6326	1.13702	4-Bromo-3,5-cyclohexadiene-1,2-dione	0.973743	-	-	-	342.176	187.998259
POS02892	962779.5178	1279325.493	0.410106	0.133046	0.4533	0.42501	Ginsenoside Rf	-6.6632423	-	-	-	342.176	802.014639
POS02894	537322.8183	726221.2634	0.43462	0.052669	0.3231	0.35251	Leucomycin A3	-1.9486262	-	-	-	342.176	829.000663
POS02895	223002.5701	230459.7776	0.047455	0.891959	0.9504	0.02715	Cyclopassifloside III	-6.8616862	-	-	-	342.176	846.023678
POS02898	410583.9449	382002.0423	-0.1041	0.648344	0.8276	0.05209	trans-2-Enoyl-OPC8-CoA	22.409291	-	-	-	342.176	1042.96263
POS02907	12052405.97	11495652.01	-0.06823	0.459482	0.7113	0.34403	Dimethyl diselenide	9.572858	-	-	-	342.175	188.999076
POS02909	219149.5107	235714.4029	0.105124	0.679272	0.8418	0.05565	(3-{4-[(E)-2-{3-[3-(3,5-dihydroxyphenyl)-6-hydroxy-2-(4 [illegible]	15.895376	-	-	-	342.175	938.00217
POS02910	5043894.798	4531088.751	-0.15468	0.11469	0.4286	0.49473	Galactosylceramide (d18:1/14:0)	-2.0380767	-	-	-	342.174	672.994007
POS02912	258441.7014	516482.4752	0.998881	0.005654	0.1571	0.46029	TG(22:6(4Z,7Z,10Z,13Z,16Z,19Z)/o-18:0/22:6(4Z,7Z,10Z,13Z [illegible]	-4.6949722	-	-	-	342.173	966.521044

POS02915	221421.8403	190585.666	-0.21636	0.43729	0.6948	0.0777	DG(15:0/0:0/20:1n9)	-8.5341552	-	-	-	342.171	609.991079
POS02918	287839.3975	380953.4836	0.404351	0.24367	0.5621	0.21151	Alamandine	-4.327718	-	-	-	342.1665	855.998876
POS02945	476489.6852	402972.8169	-0.24176	0.041288	0.2991	0.21908	Armillaramide	-22.220362	-	-	-	338.064	556.910924
POS02946	900799.5628	787320.3003	-0.19426	0.062338	0.3417	0.25717	Cryptocyanin	14.416657	-	-	-	338.054	481.398102
POS02955	190934.5697	234119.8966	0.294169	0.666346	0.8359	0.06894	3-(3,5-Diiodo-4-hydroxyphenyl)lactate	-9.0911297	-	-	-	337.717	434.969731
POS02958	184493.9658	150732.6563	-0.29158	0.022731	0.2473	0.15293	Pavoninin 1	2.3118453	-	-	-	337.707	662.874507
POS02964	1454118.584	1107154.498	-0.39329	0.097244	0.4032	0.44489	Radon-222	2.397794	-	-	-	337.416	223.007809
POS02983	84467.85981	80998.65172	-0.0605	0.687412	0.8457	0.02467	Lolicine A	-9.5606654	-	-	-	337.0215	604.832604
POS02987	72644.87622	66798.23139	-0.12105	0.474556	0.7207	0.03102	3b-Hydroxy-6b-(3-chloro-2-hydroxy-2-methylbutanoyloxy)	-10.650866	-	-	-	337.008	401.897007
POS02999	272546.7028	184556.6821	-0.56244	0.095014	0.3988	0.22272	Arsenobetaine	-20.101259	-	-	-	336.698	179.064897
POS03010	184528.1508	165883.6558	-0.15367	0.246103	0.564	0.07954	Triphosphate	1.3440786	-	-	-	336.305	253.922917
POS03016	247931.6568	209627.1767	-0.24212	0.034128	0.2814	0.1593	Lamotrigine	2.2041355	-	-	-	335.691	257.098841
POS03020	133939.4772	108058.4052	-0.30977	0.261497	0.5789	0.10565	O-phosphonato-L-homoserine(2-)	-13.548038	-	-	-	334.929	198.088606
POS03026	86272.46201	91023.12962	0.077333	0.704704	0.8557	0.02156	5a,11a-Dehydrochlortetracycline	24.17035	-	-	-	334.201	477.882503
POS03034	98109.3424	113135.9931	0.205596	0.340378	0.6326	0.06693	Clobetasol	-8.0089104	-	-	-	333.6915	411.910986
POS03035	102868.3188	71490.62136	-0.52497	0.01589	0.2226	0.14482	Pepsinostreptin	22.5561	-	-	-	333.553	672.887931
POS03038	283949.3462	264692.4783	-0.10132	0.49606	0.7344	0.05481	Melleolide M	-23.732939	-	-	-	333.451	453.921527
POS03043	175905.4692	125238.9358	-0.49012	0.007602	0.1804	0.20093	PE(14:0/P-16:0)	20.856053	-	-	-	333.125	648.927389
POS03076	616045.3321	456368.4561	-0.43284	0.114899	0.4289	0.25275	N-(2R-Hydroxyhexadecanoyl)-2S-amino-9-methyl	12.563681	-	-	-	332.0395	566.925187
POS03080	769784.5711	576018.0396	-0.41834	0.042825	0.3045	0.3607	2,4-Dinitroaniline	22.611606	-	-	-	331.8805	184.133017
POS03084	845199.0081	688755.8845	-0.2953	0.055913	0.3276	0.2967	Oxalosuccite	14.383357	-	-	-	331.4005	191.117611
POS03085	1552733.582	1241507.874	-0.32272	0.078135	0.3724	0.42773	Dimefox	-8.949497	-	-	-	331.3285	155.128897
POS03089	1047714.505	974101.1118	-0.1051	0.462438	0.7128	0.14151	Calcium oxalate	-17.639119	-	-	-	331.127	129.102017

POS03092	275400.6078	62886.9524	-2.1307	0.284249	0.6004	0.27035	Se-Methyl-L-selenocysteine	-16.099762	-	-	-	330.978	183.084145
POS03094	94723.37904	97832.98749	0.0466	0.85986	0.9363	0.01866	Ioxynil	-6.1383387	-	-	-	330.9055	371.9188
POS03098	1025019.805	680086.4216	-0.59186	0.066281	0.3498	0.43697	Didecyldimethylammonium chloride	9.6083213	-	-	-	330.667	363.086956
POS03102	3810370.393	2083245.134	-0.8711	0.029575	0.2674	1.10191	Calcium formate	-18.568118	-	-	-	330.228	131.117761
POS03106	1401085.465	1661726.234	0.246138	0.512516	0.7457	0.22148	N-(2,3-Dihydroxybenzoyl)-L-serine	6.5023157	-	-	-	329.904	242.206245
POS03109	203429.7607	191327.722	-0.08848	0.776603	0.8973	0.03681	2-Ketospirilloxanthin	-9.4567634	-	-	-	329.268	611.909599
POS03111	2624293.987	3109094.409	0.244565	0.38646	0.6583	0.40686	Threote	-24.056674	-	-	-	329.034	137.107302
POS03114	6097861.313	7065662.953	0.212522	0.677722	0.8406	0.34326	Pyrazinoic acid	19.563573	-	-	-	328.92	125.107204
POS03147	3757438.392	4179881.738	0.153713	0.668547	0.8369	0.2016	2,5-Dichloro-2,5-cyclohexadiene-1,4-diol	9.5827468	-	-	-	327.098	182.025611
POS03153	1104490.568	901161.3671	-0.29352	0.284379	0.6004	0.25289	Stipitatote	-18.097579	-	-	-	327.038	209.12801
POS03157	4380982.167	1900339.087	-1.205	0.048668	0.316	1.33109	Urate radical	19.988758	-	-	-	326.9425	168.112917
POS03158	1018913.256	794173.4376	-0.35951	0.352042	0.6364	0.25223	Nornitrogen mustard	8.6498788	-	-	-	326.9175	143.035505
POS03159	195520.2906	137406.4572	-0.50887	0.113796	0.427	0.17599	3',4'-Dihydrorhodovibrin	17.363623	-	-	-	326.88	587.947268
POS03167	1190124.753	1008317.462	-0.23916	0.020872	0.2404	0.35421	Citrate	12.853629	-	-	-	326.71	193.133246
POS03169	3674482.846	3576219.449	-0.03911	0.862151	0.9376	0.09888	Bronopol	5.4362173	-	-	-	326.693	200.996364
POS03182	66482492.49	56190402.83	-0.24265	0.053229	0.3235	2.46836	Bromobenzene	-18.355741	-	-	-	326.365	158.012295
POS03188	809184.4812	765040.7226	-0.08093	0.820833	0.9179	0.0927	3-Methyleneoxindole	-1.5709696	-	-	-	326.2675	146.165049
POS03199	4976223.983	6647941.096	0.417856	0.108102	0.4178	0.96862	Dinitrosopentamethylenetetramine	5.2958647	-	-	-	325.9715	187.180163
POS03200	1701417.393	2481182.104	0.544291	0.101978	0.4083	0.67594	Violacene	-1.4103767	-	-	-	325.967	355.932976
POS03202	2102831.153	2507360.258	0.253836	0.280618	0.5965	0.38899	3-Bromo-2Z-heptenoic acid	11.488579	-	-	-	325.827	208.074655
POS03210	141373.1997	118576.4768	-0.25369	0.046626	0.3138	0.11723	Apraclonidine	-17.62609	-	-	-	325.6235	246.111456
POS03219	15492817.46	10858114.36	-0.51283	0.259614	0.5782	1.26444	Cyazine	19.802494	-	-	-	324.929	241.704743
POS03222	13097967.79	8232708.833	-0.6699	0.014407	0.2182	1.86686	Trifluoromethyl-bismethyl ketone	16.435521	-	-	-	324.4415	141.113279

POS03223	12725051.46	11170323.54	-0.188	0.147725	0.4733	0.83246	Cyanidin	3.2044634	-	-	-	324.4045	288.252397
POS03230	713865.4894	626271.3539	-0.18886	0.26327	0.58	0.20228	Arsenobetaine	-19.641274	-	-	-	323.5175	179.064979
POS03257	325148.3992	279500.7568	-0.21825	0.06589	0.3485	0.1499	Semicarbazide	-21.368121	-	-	-	320.003	76.0755725
POS03264	878393.2822	808098.4543	-0.12034	0.220981	0.542	0.15875	1,7-diphospho-1-epi-valienol	20.774057	-	-	-	317.635	337.141259
POS03266	683703.0885	1035314.701	0.598628	0.429169	0.6879	0.3245	Ammelide	-4.3613903	-	-	-	317.534	129.096218
POS03284	687838.9955	613768.6396	-0.16438	0.55283	0.7707	0.11352	Salvarsan	-16.6448	-	-	-	315.584	440.00097
POS03310	1150177.863	1175581.586	0.031518	0.923808	0.9656	0.00255	5-Nitrofurfural	-19.902428	-	-	-	309.531	142.086069
POS03315	8159010.352	5643986.411	-0.53168	0.046495	0.3136	1.32923	Nitrosylsulfuric acid	-20.244735	-	-	-	307.08	128.081704
POS03324	72395.11792	64785.92141	-0.16021	0.435141	0.6932	0.05254	CerP(d18:1/16:0)	5.6901816	-	-	-	306.056	618.891392
POS03326	174594.4816	137781.4105	-0.34163	0.196296	0.5222	0.13772	N-[(4E,8E)-1,3-dihydroxyoctadeca-4,8-dien-2-yl]hexadecanamide	-4.7491367	-	-	-	306.051	536.889532
POS03330	131898.8599	135619.3256	0.040131	0.892912	0.9507	0.02022	Polixetonium chloride	14.094884	-	-	-	305.7085	326.20786
POS03339	902925.0005	849420.6606	-0.08813	0.485888	0.7284	0.1012	Butefine hydrochloride	13.250077	-	-	-	305.371	354.940066
POS03343	272944.6246	367316.8938	0.428417	0.204825	0.5284	0.20974	Nornitrogen mustard	8.4648092	-	-	-	303.619	143.035479
POS03346	3521504.535	3237013.764	-0.12153	0.819825	0.9178	0.0692	Dimefox	-8.9085411	-	-	-	302.594	155.128904
POS03396	583254.9844	599445.5324	0.039502	0.815805	0.9163	0.03631	L-3,4-Dihydroxybutan-2-one 4-phosphate	0.7120407	-	-	-	297.75	185.091808
POS03403	1049310.958	920642.5527	-0.18873	0.645337	0.826	0.17612	Didecyldimethylammonium chloride	8.0116921	-	-	-	296.2935	363.086377
POS03426	1534886.99	1775754.578	0.2103	0.51734	0.7487	0.2168	3-Bromo-2Z-heptenoic acid	11.209594	-	-	-	285.525	208.074598
POS03447	6649581.664	6246168.279	-0.09029	0.883433	0.9474	0.03036	Salvarsan	-16.69651	-	-	-	280.61	440.000947
POS03457	10334748.59	4745678.424	-1.12282	0.089861	0.3895	1.82199	2,5-Dichloro-2,5-cyclohexadiene-1,4-diol	9.0703901	-	-	-	279.941	182.025518
POS03470	3218864.029	2176943.671	-0.56425	0.068151	0.3534	0.73495	3,3',5,5'-Tetrachloro-4,4'-biphenyldiol	-24.353045	-	-	-	279.3295	324.986187
POS03472	10580786.84	8451163.006	-0.32423	0.547348	0.7675	0.71304	Bronopol	5.1538412	-	-	-	279.215	200.996307
POS03480	481523.2125	344453.1335	-0.4833	0.097737	0.4036	0.28666	Trichlormethine	-24.932775	-	-	-	278.948	241.987368
POS03481	109120.0306	185965.4701	0.769119	0.301812	0.6146	0.16566	Diazenedicarboxamide	-9.1637498	-	-	-	278.921	117.085013

POS03491	868737.3569	1021712.19	0.233997	0.488893	0.7301	0.21602	Costatone	24.490143	-	-	-	278.573	395.932048
POS03496	230296.6569	219148.0323	-0.07159	0.911446	0.9581	0.00636	erythro-3-Hydroxy-Ls-aspartate	19.471665	-	-	-	278.5375	150.11228
POS03507	439310.7656	302204.348	-0.53972	0.007222	0.1779	0.3426	Homotrypanothione	21.079664	-	-	-	278.2145	738.911631
POS03522	988835.986	844789.5487	-0.22714	0.134182	0.4547	0.26004	Helianyl octanoate	-20.628872	-	-	-	277.8995	555.925229
POS03531	1546038.31	962351.7197	-0.68394	0.088518	0.3878	0.60424	Triphosphate	-9.0097207	-	-	-	277.716	258.959952
POS03535	7722436.437	4537271.618	-0.76723	0.346509	0.6348	0.98763	Isometamidium chloride	-17.65161	-	-	-	277.6635	497.004421
POS03540	768832.7179	738207.191	-0.05864	0.892709	0.9507	0.0319	Nornitrogen mustard	8.1182372	-	-	-	277.541	143.03543
POS03545	3102432.816	1801626.705	-0.7841	0.058721	0.3352	0.89154	3,5-Diiodo-4-hydroxyphenylpyruvate	10.977291	-	-	-	277.416	432.962518
POS03547	1441871.245	1204900.342	-0.25903	0.407161	0.6725	0.23729	Pepsinostreptin	21.915253	-	-	-	277.376	672.887501
POS03550	163177720.6	55161160.62	-1.56472	0.019167	0.234	8.20889	Bromobenzene	-18.73238	-	-	-	277.373	158.012235
POS03561	493324.5117	423772.8953	-0.21925	0.453282	0.707	0.12792	Quicrine	19.3613	-	-	-	277.152	400.97182
POS03564	682534.3846	628499.104	-0.11899	0.347533	0.6351	0.12804	9-Hydroxytridecyl docosanoate	22.66906	-	-	-	277.1295	539.947994
POS03572	690621.0527	509895.6956	-0.43769	0.277552	0.5936	0.25795	20,21-Diprenylterpendole C	-6.0670527	-	-	-	276.943	656.908997
POS03602	342265.4242	361344.6184	0.07826	0.805545	0.9113	0.07001	Protochlorophyllide	3.2088833	-	-	-	276.387	613.966644
POS03605	4328876.736	3970450.156	-0.12469	0.764153	0.8906	0.21798	Cyazine	19.572925	-	-	-	276.3145	241.704688
POS03606	3962069.999	2622708.621	-0.5952	0.223748	0.5455	0.73456	3-Iodo-4-hydroxyphenylpyruvate	0.2869359	-	-	-	276.307	307.061364
POS03620	144632.6286	151331.6081	0.06532	0.888502	0.949	0.03223	PS(14:0/18:3(9Z,12Z,15Z))	0.0345878	-	-	-	275.2975	730.928302
POS03624	34968.37567	24837.55738	-0.49353	0.228388	0.5498	0.0575	3-(Uracil-1-yl)-L-alanine	19.995216	-	-	-	274.6035	200.175359
POS03631	1326373.043	1282442.405	-0.04859	0.889057	0.9493	0.00687	Butefine hydrochloride	13.257043	-	-	-	272.098	354.940069
POS03647	902787.3567	722547.295	-0.32129	0.251902	0.5702	0.26833	4-Bromophenylacetate	11.532733	-	-	-	266.0665	216.053757
POS03656	48988.68147	47375.57404	-0.04831	0.803526	0.9104	0.0086	3-Bromosulfolane	14.612512	-	-	-	266.009	200.076185
POS03663	31476873.29	23294122.38	-0.43433	0.190277	0.5172	1.78116	Cuprous oxide	21.025449	-	-	-	265.7525	144.101685
POS03671	2041042.636	1672942.047	-0.28692	0.47518	0.721	0.29382	3-Bromo-2Z-heptenoic acid	11.11712	-	-	-	265.5235	208.074579

POS03676	12540680.03	3369845.23	-1.89586	0.295515	0.6092	2.11094	2,5-Dichloro-2,5-cyclohexadiene-1,4-diol	9.0054989	-	-	-	265.372	182.025507
POS03688	2682662.664	2597811.962	-0.04637	0.879584	0.9466	0.06624	Salvarsan	-16.73689	-	-	-	264.767	440.000929
POS03690	67928.99055	58263.83918	-0.22143	0.665598	0.8354	0.02951	2-Oxoglutaramate	-22.80959	-	-	-	264.7515	146.117367
POS03705	333641.8856	356935.0927	0.097361	0.698181	0.8513	0.05577	4-Hydroxybutanoic acid	-15.402936	-	-	-	261.7585	105.110173
POS03706	8092941.622	8000396.476	-0.01659	0.935558	0.9711	0.05497	3-hydroxy-2-isobutyrate	14.450066	-	-	-	261.695	104.106766
POS03707	554046.4445	492910.5048	-0.16868	0.178949	0.5052	0.15254	Aldehydo-L-iduronate	-6.6775713	-	-	-	261.572	194.137987
POS03719	342566.2634	241647.1977	-0.50348	0.009773	0.194	0.28245	2,4-Dichlorophenol	-11.937161	-	-	-	258.3885	164.006731
POS03730	663677.4339	626890.3075	-0.08227	0.710866	0.8589	0.07664	4-Nitrocatechol	-15.873237	-	-	-	257.944	156.113015
POS03749	234780.8008	374483.7051	0.673589	0.1728	0.4988	0.21959	4-Oxoproline	9.336823	-	-	-	256.6875	130.122482
POS03754	171065.4172	128794.0271	-0.40948	0.040173	0.2969	0.16184	Didecyldimethylammonium chloride	8.1855776	-	-	-	256.17	363.08644
POS03758	835824.0759	1022236.922	0.290458	0.494553	0.7339	0.19794	Nitrosylsulfuric acid	-20.311341	-	-	-	255.998	128.081695
POS03763	453274.2695	372344.849	-0.28374	0.040892	0.2988	0.23093	Flucytosine	-18.191013	-	-	-	255.3015	130.097428
POS03773	511881.2019	460854.8769	-0.1515	0.475125	0.721	0.11872	Aminomalote	-23.329368	-	-	-	252.885	120.080599
POS03782	905680.011	447569.4534	-1.01689	0.08359	0.3803	0.50493	3,5-Diiodo-4-hydroxyphenylpyruvate	10.807858	-	-	-	251.795	432.962445
POS03786	3955004.006	2130330.616	-0.8926	0.091343	0.3917	1.07855	Isometamidium chloride	-17.750377	-	-	-	251.613	497.004372
POS03802	15467556.21	5948279.842	-1.3787	0.198286	0.524	1.92888	Cyazine	19.695158	-	-	-	250.541	241.704717
POS03804	845202.6075	665953.3238	-0.34388	0.133115	0.4534	0.31852	Butefine hydrochloride	13.118155	-	-	-	250.2525	354.940019
POS03812	52569562.2	40626163.31	-0.37182	0.163014	0.4904	2.41613	Bromobenzene	-18.686155	-	-	-	248.329	158.012243
POS03840	1984570.756	1580412.755	-0.32853	0.191359	0.5181	0.41002	Calcium oxalate	-17.710631	-	-	-	245.6625	129.102008
POS03844	2206806.672	1461598.535	-0.59441	0.243695	0.5621	0.53713	3-Iodo-4-hydroxyphenylpyruvate	0.2572816	-	-	-	245.397	307.061355
POS03876	4171382.895	3240896.388	-0.36413	0.507014	0.7431	0.45565	Salvarsan	-16.748248	-	-	-	244.666	440.000924
POS03883	1873435.573	2749419.174	0.553441	0.407004	0.6725	0.50816	3-Bromo-2Z-heptenoic acid	11.186783	-	-	-	244.335	208.074593
POS03893	7509564.832	6825135.829	-0.13787	0.839728	0.9271	0.33011	cis-4-Carboxymethylenebut-2-en-4-olide	7.1568406	-	-	-	244.003	141.101879

POS03902	4190385.03	6882105.679	0.715767	0.125824	0.4446	1.09668	2,5-Dichloro-2,5-cyclohexadiene-1,4-diol	9.1125916	-	-	-	243.962	182.025526
POS03909	787872.0162	1060540.403	0.428766	0.293081	0.6078	0.2958	Nornitrogen mustard	8.1641219	-	-	-	243.665	143.035436
POS03915	2889792.004	5274925.478	0.868185	0.184936	0.5109	1.11243	Bronopol	5.1262394	-	-	-	243.506	200.996302
POS03926	55191.94809	49776.32822	-0.149	0.473205	0.7201	0.03578	Pyruvate oxime	-23.219453	-	-	-	241.638	104.081583
POS03955	1026093.044	859025.8807	-0.25639	0.166975	0.4933	0.29627	4-Nitrocatechol	-15.810901	-	-	-	231.651	156.113024
POS03956	6476396.699	4910717.986	-0.39926	0.008334	0.1881	1.0723	Cyazine	19.575596	-	-	-	231.576	241.704688
POS03962	849143.0241	1337005.494	0.654926	0.054663	0.3261	0.55894	3,3',5,5'-Tetrachloro-4,4'-biphenyldiol	21.244512	-	-	-	230.681	325.00096
POS03970	229563.5955	186805.8506	-0.29735	0.705053	0.8558	0.06367	2-Aminomucote	-17.947183	-	-	-	227.962	158.128557
POS03981	1147599.886	830764.4353	-0.46611	0.051123	0.3203	0.43194	3-Iodo-4-hydroxyphenylpyruvate	-0.0540117	-	-	-	225.052	307.06126
POS03982	776629.1875	656823.2278	-0.24172	0.175056	0.501	0.22181	Sodium methallylsulfote	-9.9760424	-	-	-	224.578	159.156999
POS03991	1717237.231	1516699.955	-0.17915	0.393078	0.6624	0.19829	Salvarsan	-16.695619	-	-	-	223.632	440.000947
POS04007	263374.8627	216835.9757	-0.28051	0.014923	0.2218	0.18276	N-phosphocreatinate(2-)	15.547602	-	-	-	223.1335	210.109528
POS04011	23078.4534	22037.62206	-0.06658	0.79879	0.9083	0.00848	Bropirimine	-1.6160298	-	-	-	223.086	267.100847
POS04034	3190865.221	3062299.36	-0.05933	0.802784	0.91	0.06046	Bronopol	5.2090646	-	-	-	222.284	200.996318
POS04046	1326445.881	390929.8028	-1.76258	0.080031	0.3748	0.71185	Succinimide	-4.7769668	-	-	-	221.182	100.094803
POS04068	1025452.342	849331.6304	-0.27186	0.454324	0.7079	0.23945	3-Bromo-2Z-heptenoic acid	11.288422	-	-	-	218.589	208.074614
POS04076	23203379.38	15857348.14	-0.54918	0.43032	0.689	1.43931	2,5-Dichloro-2,5-cyclohexadiene-1,4-diol	9.3940365	-	-	-	217.941	182.025577
POS04082	208422.9312	171683.35	-0.27976	0.223714	0.5455	0.12041	5-FU	-23.433209	-	-	-	217.261	131.081428
POS04091	978585.1562	1144522.96	0.225977	0.437544	0.695	0.21395	Urate radical	19.766538	-	-	-	216.0635	168.11288
POS04094	36516534.74	30163510.27	-0.27575	0.068894	0.3541	2.00537	Bromobenzene	-18.55415	-	-	-	215.7005	158.012263
POS04100	874078.9332	932894.1855	0.09395	0.777038	0.8976	0.09113	Nornitrogen mustard	8.2983523	-	-	-	214.866	143.035455
POS04102	138632.9853	70784.89627	-0.96976	0.019619	0.2365	0.2291	Sulfur mustard	13.573599	-	-	-	214.5755	160.086736
POS04103	1083943.219	817221.6349	-0.40749	0.021143	0.2424	0.45434	Oxaloacetate	-23.422636	-	-	-	214.2445	133.075783

POS04122	1292912.609	1261827.731	-0.03511	0.904339	0.9559	0.00016	Salvarsan	-16.653903	-	-	-	210.8885	440.000966
POS04129	625765.2084	679007.0383	0.117805	0.622095	0.8124	0.04937	Calcium L-aspartate	-16.540444	-	-	-	210.0255	172.169245
POS04143	11914339.92	12293436.97	0.045189	0.871459	0.9428	0.35386	Cyazine	19.726806	-	-	-	206.8465	241.704725
POS04160	5696978.458	4914844.664	-0.21305	0.036	0.2855	0.70244	Cryptocyanin	14.40575	-	-	-	203.647	481.398097
POS04162	4673135.982	4101759.373	-0.18815	0.246649	0.5647	0.39959	Calcium oxalate	-17.476522	-	-	-	203.159	129.102038
POS04192	6146874.301	6660346.233	0.115744	0.562017	0.776	0.38566	Pyrazinoic acid	19.396135	-	-	-	199.098	125.107184
POS04202	81860.80421	40325.81939	-1.02147	0.051194	0.3203	0.15977	Pyrazolate	-17.866667	-	-	-	197.732	440.311728
POS04216	5801670.128	7100663.822	0.291486	0.601242	0.7983	0.60337	Bronopol	5.272321	-	-	-	196.071	200.996331
POS04219	101607.609	124145.9256	0.289028	0.192375	0.519	0.11801	Hydroxyzine	-18.040689	-	-	-	195.733	375.904813
POS04225	38338.1554	39737.85823	0.051733	0.774696	0.8961	0.01662	Aldotetraouronic acid	-20.170384	-	-	-	195.399	605.504883
POS04229	951409.6898	766221.1588	-0.31231	0.396863	0.6661	0.25935	(S)-Malate	-23.813768	-	-	-	195.0425	135.091483
POS04234	1397151.465	641007.3642	-1.12408	0.077821	0.3724	0.67413	Salvarsan	-16.652792	-	-	-	194.729	440.000966
POS04249	2148202.487	3161357.808	0.557414	0.194006	0.5197	0.72344	3-Bromo-2Z-heptenoic acid	11.402879	-	-	-	194.029	208.074638
POS04261	5457641.348	4131865.45	-0.40148	0.03338	0.2802	0.92437	Threote	-24.30564	-	-	-	193.377	137.107269
POS04275	99073059.29	98168981.9	-0.01323	0.961389	0.9825	0.30391	Bromobenzene	-18.553981	-	-	-	192.882	158.012263
POS04297	603170.0352	237359.2979	-1.34549	0.346073	0.6346	0.31396	Se-Methyl-L-selenocysteine	23.120073	-	-	-	191.6995	183.091286
POS04300	371294.2513	351862.876	-0.07755	0.77507	0.8962	0.02027	cis-Chlorobenzene dihydrodiol	1.6373604	-	-	-	191.6155	250.914486
POS04311	9336298.288	6176682.443	-0.59602	0.085454	0.3847	1.38643	2,5-Dichloro-2,5-cyclohexadiene-1,4-diol	9.540357	-	-	-	191.173	182.025604
POS04315	1121173.426	1148747.981	0.035053	0.954806	0.98	0.03206	Nornitrogen mustard	8.4528608	-	-	-	191.035	143.035477
POS04323	117335.6399	73702.992	-0.67085	0.071311	0.3591	0.15906	Butechlor	-13.359663	-	-	-	190.1875	310.834137
POS04325	1496961.782	969156.5796	-0.62724	0.045337	0.3118	0.54067	3-Iodo-4-hydroxyphenylpyruvate	0.3171337	-	-	-	190.028	307.061374
POS04328	395538.5661	427547.6038	0.112267	0.494354	0.7338	0.10655	Safranin	24.328394	-	-	-	190.022	351.860512
POS04334	42064.63627	57828.50289	0.459173	0.228226	0.5496	0.08809	Bowdichione	9.231148	-	-	-	189.6975	299.25703

POS04353	293017.6306	211848.2124	-0.46796	0.118505	0.4336	0.19298	Nitarsone	-0.3090056	-	-	-	188.19	248.0443
POS04357	13031662.43	3783215.206	-1.78434	0.159991	0.4875	2.22993	Potassium iodide	14.597682	-	-	-	188.02	167.0125
POS04364	243604.319	211758.8226	-0.20212	0.418111	0.6797	0.1059	Triasulfuron	-20.299409	-	-	-	187.6935	402.82442
POS04387	395029.4883	247308.535	-0.67565	0.003075	0.1183	0.35522	2-Oxoglutaramate	-22.762418	-	-	-	186.349	146.117373
POS04392	86317.1559	82616.78437	-0.06321	0.823354	0.9188	0.0103	Tepoxalin	-11.223612	-	-	-	185.692	386.847046
POS04406	1861735.644	1994268.783	0.099212	0.701586	0.8537	0.16066	Citrate	12.837591	-	-	-	185.047	193.133243
POS04407	3799881.9	2018150.234	-0.91292	0.000148	0.0721	1.29922	Isopentenyl phosphate	-10.928652	-	-	-	185.0275	167.117661
POS04413	10226941.74	7823855.041	-0.38642	0.013318	0.2135	1.37795	Trimethylselenonium	-0.5395521	-	-	-	184.7085	125.07081
POS04416	2576079.956	2278134.632	-0.17732	0.296129	0.6098	0.2633	Oxalosuccite	14.161119	-	-	-	184.7005	191.117569
POS04424	3874855.12	2625353.601	-0.56163	0.387259	0.6585	0.63933	cis-4-Carboxymethylenebut-2-en-4-olide	8.0723143	-	-	-	184.423	141.102007
POS04427	934098.2784	779239.7055	-0.26151	0.017147	0.2275	0.32705	Calcium glycerophosphate	3.5412678	-	-	-	184.38	211.143821
POS04432	1552414.63	905396.6576	-0.77789	0.04782	0.3143	0.6771	p-Aminobenzamidine dihydrochloride	18.788174	-	-	-	184.365	209.099486
POS04439	398011.2654	354977.8858	-0.16508	0.345322	0.6341	0.10847	3-Phospho-D-glyceroyl phosphate	-7.6096204	-	-	-	184.349	267.042352
POS04441	1371824.53	1015264.241	-0.43424	0.009081	0.1917	0.51663	4-Amino-2-methyl-5-(phosphooxymethyl)pyrimidine	6.6623756	-	-	-	184.3195	220.143837
POS04442	1997521.248	1869776.502	-0.09535	0.790171	0.9034	0.10617	Stipitatote	-17.945894	-	-	-	184.3085	209.128042
POS04446	71873.89584	69073.30418	-0.05734	0.83823	0.9265	0.0114	3-keto Fusidic acid	5.959136	-	-	-	184.259	531.703139
POS04466	1342060.645	1105616.56	-0.2796	0.010378	0.1964	0.41688	2,4-Dinitroaniline	22.385338	-	-	-	184.0335	184.132976
POS04474	1017605.919	1094866.334	0.105576	0.648489	0.8276	0.13177	5-Nitrofurfural	-19.556877	-	-	-	184.025	142.086117
POS04478	3677575.483	4042339.781	0.136436	0.638761	0.8217	0.32988	Trifluoromethanesulfonic acid	14.34389	-	-	-	184.019	151.086429
POS04482	1455906.279	1124257.872	-0.37294	0.015813	0.2226	0.50729	3-(Imidazol-4-yl)-2-oxopropyl phosphate	5.0794301	-	-	-	184	221.128195
POS04483	1395206.906	1231228.538	-0.18038	0.293817	0.6082	0.2221	Tetrahydroxypteridine	1.8047864	-	-	-	183.978	197.128031
POS04487	1448238.44	1425730.879	-0.0226	0.90621	0.9567	0.03199	D-Ribose 5-phosphate	-20.517095	-	-	-	183.894	231.112355
POS04489	1308012.74	1392313.429	0.090107	0.731424	0.8719	0.07782	5-Amino-4-imidazolecarboxyamide	-8.3754831	-	-	-	183.8905	127.12282

POS04497	152317.6398	87088.38493	-0.80653	0.021185	0.2424	0.22377		Phenylmercury acetate	24.841768	-	-	-	183.8525	337.753542
POS04503	946158.8472	1743301.347	0.881668	0.447532	0.7021	0.50245		2,5-Furandicarboxylate	-20.935939	-	-	-	183.7875	157.097009
POS04511	1105151.239	1089639.903	-0.02039	0.928239	0.9674	0.01744		D-Ribitol 5-phosphate	-21.636497	-	-	-	183.72	233.127954
POS04519	1968143.037	1549989.702	-0.34458	0.039206	0.2958	0.5408		Methyl 2-propenyl selenide	-12.533923	-	-	-	183.702	136.075584
POS04531	1089207.387	811560.552	-0.42451	0.052192	0.3219	0.40491		unsym-Bis(4'-chlorophenyl)ethylene	2.3161215	-	-	-	183.685	250.143054
POS04532	401247.9361	293015.7761	-0.45352	0.455338	0.7084	0.21142		5-FU	-23.771404	-	-	-	183.6825	131.081384
POS04533	2583942.079	2155475.584	-0.26157	0.211411	0.5346	0.35743		3-Oxalomalate	-9.1726574	-	-	-	183.68	207.112386
POS04535	1636100.709	1618999.695	-0.01516	0.937972	0.972	0.03097		O-Phospho-4-hydroxy-L-threonine	-19.889045	-	-	-	183.6485	216.101498
POS04536	3175179.677	2340844.727	-0.43981	0.054075	0.3242	0.69299		2,3,6-Trihydroxypyridine	11.415018	-	-	-	183.6475	128.106827
POS04548	3059751.134	2564302.108	-0.25485	0.378921	0.653	0.37416		[5-(Aminomethyl)furan-3-yl]methyl phosphate	21.66743	-	-	-	183.505	208.132864
POS04552	158948.5855	128414.0275	-0.30776	0.040752	0.2986	0.15394		3alpha,7alpha,12alpha,24(S)-tetrahydroxy-5beta-cholestan-27-al	13.052271	-	-	-	183.415	451.673159
POS04559	181057.0033	143742.4098	-0.33296	0.228827	0.5501	0.13082		1-Cyclohexyl-11-heneicosanone	16.183322	-	-	-	183.381	393.714832
POS04566	141202.303	113808.6273	-0.31115	0.255599	0.5732	0.12462		1,26-Hexacosanediol	-18.115511	-	-	-	183.378	399.705854
POS04573	445410.0146	392271.6409	-0.18328	0.122722	0.4403	0.16026		Pentacosanoylglycine	23.526785	-	-	-	183.3745	440.732222
POS04579	873507.1912	676619.6925	-0.36847	0.013832	0.2168	0.39389		Apraclonidine	-15.794345	-	-	-	183.371	246.111905
POS04584	213030.2382	202562.1185	-0.07269	0.65096	0.829	0.03348		Kolaflavanone	-19.693646	-	-	-	183.362	589.510787
POS04589	907073.9421	911806.6449	0.007508	0.988185	0.9937	0.05595		S-(1,2-Dichlorovinyl)-L-cysteine	17.922703	-	-	-	183.357	217.096749
POS04617	1065741.151	1034543.71	-0.04286	0.88008	0.9466	0.04629		4-Nitrophenyl phosphate	2.5749694	-	-	-	183.1255	220.096541
POS04621	81747.7403	90354.91338	0.144424	0.557913	0.7733	0.0314		2-Methylhexacosane	-24.151714	-	-	-	183.0535	381.731581
POS04633	610347.667	466776.3646	-0.3869	0.006704	0.1711	0.35336		5alpha-Cyprinol	-8.7195209	-	-	-	183.044	453.67033
POS04634	1257346.998	722584.5381	-0.79914	0.010467	0.1964	0.64456		2-Chloroethanol	-4.6651054	-	-	-	183.0425	81.520401
POS04638	58585.43976	50107.02044	-0.22553	0.166041	0.493	0.06564		Demethylphylloquinol	-5.1919106	-	-	-	183.041	439.689999
POS04640	665557.4368	437909.119	-0.60393	0.039893	0.2968	0.39029		Barban	-3.6457894	-	-	-	183.0385	259.107036

POS04648	97894.06961	67892.50419	-0.52797	0.010585	0.1971	0.15906	20-COOH-leukotriene E4	6.3262146	-	-	-	183.0255	470.602247
POS04651	74295.99955	46910.97422	-0.66336	0.026352	0.2576	0.13686	Indapamide	11.565164	-	-	-	183.012	366.846508
POS04653	298627.6697	248183.335	-0.26694	0.130178	0.4485	0.15497	Callystatin A	-0.8447214	-	-	-	183.001	457.664191
POS04660	1055131.82	954302.5982	-0.1449	0.290165	0.6045	0.16065	Dichlormid	-3.3754879	-	-	-	182.879	209.091574
POS04665	699180.843	663407.9557	-0.07577	0.666637	0.8359	0.06613	Se-Propenylselenocysteine Se-oxide	-3.8517779	-	-	-	182.725	225.122813
POS04669	3464552.238	2649438.378	-0.38698	0.050639	0.3192	0.68556	Thenylchlor	-9.8537491	-	-	-	182.718	324.841686
POS04674	315507.2357	270783.7004	-0.22053	0.075409	0.3679	0.13977	S-(4-Bromophenyl)-mercaptopyruvate	-15.624998	-	-	-	182.714	276.122078
POS04675	748398.8218	631292.1123	-0.2455	0.175021	0.501	0.20747	Glyphosate	3.4921699	-	-	-	182.7125	170.080967
POS04686	616616.6205	605747.7027	-0.02566	0.882737	0.9474	0.02469	Benoxacor	-3.9878068	-	-	-	182.703	261.122739
POS04687	6411718.605	5188587.897	-0.30537	0.093387	0.3962	0.75628	Cyanidin	3.0315167	-	-	-	182.7015	288.252347
POS04688	479871.6927	396943.017	-0.27372	0.178296	0.5043	0.18754	13-chloro-5,14-dihydroxy-7,12-dimethyl-6-(3-methylbut-2-en-1-yl)-10	-20.231132	-	-	-	182.699	403.829127
POS04693	909999.3878	850015.2797	-0.09838	0.450239	0.7041	0.10605	Dihydrokaempferol	-12.98462	-	-	-	182.696	289.255734
POS04695	856996.2955	783305.7777	-0.12971	0.55008	0.7693	0.12663	3-(Phosphoacetylamido)-L-alanine	8.220319	-	-	-	182.6955	243.133067
POS04696	113664.6905	95922.4175	-0.24484	0.477763	0.7223	0.06807	F5231	16.216039	-	-	-	182.695	382.799568
POS04700	62855.37839	52639.60209	-0.25589	0.397152	0.6662	0.05282	7-Chloro-3,4',5,6,8-pentamethoxyflavone	4.2258167	-	-	-	182.601	407.822996
POS04707	222425.8851	175031.5964	-0.34571	0.063089	0.3422	0.17375	Chlorcyclizine	-24.91315	-	-	-	182.482	301.825782
POS04713	721330.574	619078.0483	-0.22054	0.669229	0.837	0.09503	(1S,3R)-3-(2,2-Dichloroethenyl)-2,2-dimethylcyclopropanecarb	-7.3821019	-	-	-	182.393	210.075533
POS04714	655865.8442	696414.6347	0.086546	0.831005	0.9228	0.10353	Alendronic acid	14.133109	-	-	-	182.379	250.106797
POS04719	546220.3711	434858.1846	-0.32894	0.242668	0.5618	0.25477	Stigmastane	-3.7442225	-	-	-	182.37	401.728976
POS04724	56830.12603	40759.4413	-0.47952	0.084297	0.3821	0.0921	4-Chloroprogesterone	0.9822212	-	-	-	182.363	349.914419
POS04735	816942.253	632139.4039	-0.36999	0.00924	0.1917	0.37959	Butachlor	13.715195	-	-	-	182.261	312.858354
POS04738	374368.2178	344652.5314	-0.11932	0.669666	0.837	0.07607	BMS 204352	-20.599629	-	-	-	182.1935	360.702567
POS04742	317357.1495	226824.4854	-0.48453	0.00718	0.1779	0.26598	Cyazofamid	9.5658222	-	-	-	182.125	325.796283

POS04783	360412.3041	199800.6802	-0.85109	0.059461	0.3364	0.31293	Haloxyfop	-22.254818	-	-	-	181.8525	362.699627
POS04794	1506520.208	1468155.265	-0.03722	0.822046	0.9184	0.05671	1,7-diphospho-1-epi-valienol	20.717771	-	-	-	181.716	337.14124
POS04796	4320840.501	4950834.053	0.19636	0.669781	0.837	0.3742	2,6-Dibromohydroquinone	23.508577	-	-	-	181.7125	268.916375
POS04801	70641034.81	57771659.51	-0.29014	0.022149	0.2454	2.86763	Cyazine	19.318607	-	-	-	181.673	241.704626
POS04808	10126837.73	8454058.213	-0.26047	0.067083	0.3515	0.9559	N-(2,3-Dihydroxybenzoyl)-L-serine	6.2886937	-	-	-	181.6235	242.206193
POS04821	589344.0964	546709.9681	-0.10833	0.489586	0.7306	0.0743	12,13,14-trihydroxy-9-oxo-8,17-dioxatetracyclo[8.7.0.0	-0.6995524	-	-	-	181.372	343.266037
POS04833	207945.5002	196686.1637	-0.08031	0.754895	0.8858	0.00659	Chondroitin sulfate	-10.801753	-	-	-	181.057	464.371271
POS04835	7960298.105	3628347.674	-1.13351	0.000355	0.0721	2.04822	Iodate	16.641784	-	-	-	181.047	175.912887
POS04866	934313.9795	726346.9753	-0.36325	0.256712	0.5746	0.26334	6-[2,3-dihydroxy-5-(3,5,7-trihydroxy-3,4-dihydro-2H-1-benzopyran-2	6.8419402	-	-	-	180.376	483.404577
POS04875	26833204.37	14615582.71	-0.87651	0.18995	0.5169	2.15616	Cryptocyanin	14.509507	-	-	-	180.309	481.398147
POS04881	529479.1065	481082.294	-0.13829	0.459059	0.7112	0.10978	4-Bromophenol	-24.486489	-	-	-	180.068	174.01034
POS04892	2817420.152	2424771.285	-0.21653	0.469921	0.718	0.31469	Bismuth	-4.2524258	-	-	-	179.91	209.986788
POS04895	38041.0567	38014.77302	-0.001	0.992341	0.996	0.0056	C.I. Acid Yellow 17	10.495548	-	-	-	179.716	508.337601
POS04907	1789936.53	1280827.789	-0.48283	0.014272	0.218	0.6006	3-Fluoro-cis,cis-mucote	-0.378353	-	-	-	179.033	161.107116
POS04909	187620.2687	174189.4568	-0.10716	0.439745	0.6968	0.06569	2-O-[2-O-(alpha-D-Mannopyranosyl)-alpha-D-glucopyranosyl]-3	21.89586	-	-	-	178.726	511.356851
POS04917	68498.3318	51479.47828	-0.41207	0.117035	0.432	0.08847	3'-(2',3'-Digalloylglucosyl)-phloroacetophenone	9.6839019	-	-	-	177.323	635.509221
POS04918	537084.8386	594881.5543	0.147452	0.681973	0.8435	0.07539	Atheroline	20.784629	-	-	-	177.314	338.340388
POS04919	63344.2338	68636.15761	0.115755	0.558587	0.7736	0.04195	alpha-D-Galactosyl-N-acetyllactosamine	-19.509005	-	-	-	177.147	546.485635
POS04920	6866391.18	4815635.047	-0.51183	0.057375	0.3315	1.16151	Dinitrosopentamethylenetetramine	5.6192866	-	-	-	177.044	187.180223
POS04929	3630817.115	3447020.407	-0.07494	0.724167	0.8676	0.14369	Calcium oxalate	-17.215802	-	-	-	176.314	129.102071
POS04930	122494.5579	108950.5874	-0.16904	0.433651	0.6919	0.06001	23-Hydroxyphysalolactone	-22.512328	-	-	-	176.278	556.051781
POS04934	80479.49345	103389.3275	0.361394	0.460134	0.7119	0.08802	1-Methyladenosine	11.073249	-	-	-	176.0405	282.278291
POS04936	1398645.079	1082467.636	-0.36971	0.363016	0.6446	0.37114	Dihydrobiopterin	18.489288	-	-	-	175.94	240.2429

POS04943	154091.2329	196925.4594	0.353865	0.049924	0.3189	0.17958	DG(22:5(4Z,7Z,10Z,13Z,1 6Z)/22:6(4Z,7Z,10Z,13Z,1 6Z,19Z)/0:0)	-6.2163722	-	-	-	175.354	716.058532
POS04947	300530.9352	358017.068	0.252515	0.343889	0.6332	0.1174	DG(22:6(4Z,7Z,10Z,13Z,1 6Z,19Z)/22:6(4Z,7Z,10Z,1 3Z,16Z,19Z)/0:0)	20.615054	-	-	-	175.0845	714.061776
POS04949	5495930.804	3712547.503	-0.56595	0.020635	0.2393	1.08178	Pyrazinoic acid	19.398815	-	-	-	174.939	125.107184
POS04950	161924.6636	149773.3862	-0.11254	0.700492	0.853	0.01636	Koryoginsenoside R1	-22.071234	-	-	-	174.908	870.074695
POS04951	1368558.63	1752672.699	0.356899	0.180998	0.5068	0.39819	3-Hydroxy-2-methylpyridine-4,5-dicarboxylate	-18.698579	-	-	-	174.898	198.14849
POS04956	6206009.92	5119871.579	-0.27756	0.449403	0.7037	0.61887	2,4-Dihydroxypteridine	-9.9676991	-	-	-	174.591	165.127241
POS04957	854026.1996	722296.5267	-0.24169	0.053126	0.3235	0.27473	N-Acetyldemethylphosphinothricin	20.175518	-	-	-	174.591	210.148396
POS04960	947534.9829	765045.1752	-0.30863	0.023636	0.2494	0.34687	2-Methyl-3-hydroxy-5-formylpyridine-4-carboxylate	5.3589604	-	-	-	174.564	182.153747
POS04966	208731.1602	257095.2814	0.300657	0.093681	0.3967	0.17004	Pectenotoxin 1	10.583661	-	-	-	174.252	876.066238
POS04971	3716298.004	3794110.066	0.029895	0.863008	0.9376	0.03565	Threote	-24.596734	-	-	-	173.906	137.107229
POS04980	350173.011	338608.6771	-0.04845	0.872225	0.9428	0.02227	Trichloro(docosyl)silane	10.918837	-	-	-	173.552	445.052125
POS04982	317652.0446	414096.3596	0.382519	0.149813	0.4753	0.21208	Avermectin A1b	-16.219453	-	-	-	173.3565	874.070016
POS04984	192977.206	211134.0488	0.129729	0.613652	0.8077	0.05803	SM(d18:0/16:0)	18.145178	-	-	-	173.217	707.071988
POS04997	1155273.065	990209.2678	-0.22243	0.411363	0.6761	0.17297	SM C16:1	5.7034485	-	-	-	172.177	717.058161
POS04998	1459514.852	1205085.906	-0.27635	0.047375	0.3143	0.42427	5-Amino-4-imidazolecarboxyamide	-8.073721	-	-	-	172.137	127.122858
POS05008	432739.439	409398.0115	-0.07999	0.760857	0.8884	0.03264	PC(14:0/P-18:0)	11.326037	-	-	-	171.487	719.054909
POS05033	272450.3788	254581.558	-0.09787	0.757964	0.8874	0.0452	Victorin C	-19.627485	-	-	-	170.58	817.071659
POS05038	940532.8789	762667.1295	-0.30242	0.26879	0.5858	0.25372	2-Hydroxymucote semialdehyde	7.6480077	-	-	-	170.3375	143.117763
POS05041	742643.6434	746276.2553	0.00704	0.977476	0.9893	0.04757	Isocil	-14.531699	-	-	-	169.871	248.092786
POS05046	254747.071	276366.5561	0.117517	0.732549	0.8729	0.07818	Potassium bromide	12.650318	-	-	-	169.4035	120.011082
POS05050	415691.135	423227.2899	0.025921	0.852026	0.9327	0.03908	Nitrosylsulfuric acid	-20.842274	-	-	-	168.164	128.081628
POS05053	5035530.533	3879867.417	-0.37614	0.059736	0.3366	0.82485	Oxaloacetate	-22.519889	-	-	-	167.905	133.075902
POS05060	49965.89934	60515.15032	0.276353	0.612539	0.8073	0.03633	2-[({3,4-dihydroxy-5-[2,4,5-trihydroxy-3-(3,4,5-trihydroxybenzoyloxy)ben	7.0139296	-	-	-	167.347	548.392116

POS05071	308302.5428	234699.2874	-0.39353	0.071607	0.36	0.21153	2,6-Dichloroindophenol	-9.8858688	-	-	-	166.353	269.100126
POS05073	3005011.479	2220400.286	-0.43655	0.030684	0.2697	0.76186	2,6-Dichloroindophenol	14.389882	-	-	-	166.142	269.106634
POS05081	16150647.28	18008710.56	0.157103	0.737675	0.8757	0.19397	Cryptocyanin	14.617442	-	-	-	165.792	481.398199
POS05091	779960.4542	1152060.361	0.562743	0.283086	0.5991	0.34556	6-[2,3-dihydroxy-5-(3,5,7-trihydroxy-3,4-dihydro-2H-1-benzopyran-2	6.9688365	-	-	-	165.68	483.404638
POS05104	347289.8824	214895.4348	-0.69251	0.025819	0.2571	0.31001	4-Bromophenol	-24.476738	-	-	-	165.264	174.010342
POS05105	4443615.955	3347531.594	-0.40864	0.020273	0.2381	0.89758	Iodate	16.658966	-	-	-	165.264	175.91289
POS05119	1418128.387	1135180.844	-0.32107	0.019453	0.2351	0.44266	Bismuth	-4.2731319	-	-	-	164.766	209.986784
POS05125	762892.7188	644852.7458	-0.24251	0.059147	0.3361	0.25884	N,N'-Dimethylurea	-4.4622979	-	-	-	164.672	89.1152834
POS05143	964277.2939	544785.5138	-0.82376	0.070215	0.357	0.48469	Thenylchlor	-9.7114292	-	-	-	164.067	324.841732
POS05153	1715203.95	1361252.248	-0.33345	0.115439	0.43	0.39863	Dichlormid	-3.0112379	-	-	-	163.999	209.09165
POS05161	1258781.475	1343979.196	0.094483	0.758953	0.8875	0.14869	Creatinine	22.006177	-	-	-	163.7095	114.127666
POS05170	122767.6622	94033.76122	-0.38468	0.133774	0.4541	0.12349	Stearoylcarnitine	8.8241267	-	-	-	163.6	428.67195
POS05177	5122445.327	3857661.743	-0.40911	0.316811	0.6253	0.74337	Isopentenyl phosphate	-10.589861	-	-	-	163.156	167.117717
POS05180	100981.7373	99635.54698	-0.01936	0.92804	0.9674	0.00825	({6-[5,7-dihydroxy-4-oxo-8-(3,4,5-trihydroxyoxan-2-yl)-2-(2,4,5	-1.7323514	-	-	-	163.079	677.556105
POS05192	72050.23462	57601.62917	-0.32289	0.198038	0.5236	0.08102	Octacosanal	-19.381366	-	-	-	162.983	409.742955
POS05193	226837.5995	230964.5672	0.026012	0.899965	0.9537	0.01679	Bis(4-nitrophenyl)phosphate	-23.394965	-	-	-	162.979	341.181518
POS05202	138967.4115	25202.85486	-2.46309	0.307649	0.6189	0.17851	DIBOA-glucoside	12.457276	-	-	-	162.665	344.297653
POS05203	72206.52784	68636.63165	-0.07315	0.758369	0.8874	0.01478	({6-[2-(3,4-dihydroxyphenyl)-5,7-dihydroxy-4-oxo-3-[3,4,5	-23.276903	-	-	-	162.665	691.571202
POS05209	694201.765	379211.9001	-0.87235	0.034856	0.2838	0.45149	Dianthalexin	-6.6781041	-	-	-	162.655	240.231879
POS05211	900386.3202	577139.9922	-0.64162	0.006204	0.1646	0.52413	Fenoldopam	2.9620437	-	-	-	162.652	306.764282
POS05214	148311.7162	136128.8265	-0.12366	0.533063	0.7588	0.04782	Hydroxypyruvate	8.7247426	-	-	-	162.529	105.069685
POS05218	396436.6289	423453.9038	0.095115	0.887725	0.9486	0.01234	3,5-Dinitrosalicylic acid	-24.809656	-	-	-	162.412	229.117517
POS05219	449550.2294	225543.8557	-0.99507	0.001956	0.0991	0.44859	SQ 26180	-4.9482589	-	-	-	162.396	239.224498

POS05222	137053.2095	103905.6315	-0.39946	0.052361	0.3227	0.14401	2-Bromomaleylacetate	24.957083	-	-	-	162.325	238.018092
POS05232	222754.7689	172059.7396	-0.37255	0.495303	0.7341	0.10932	Nebularine	3.709287	-	-	-	162.309	253.234912
POS05234	992943.5283	960499.3751	-0.04793	0.78147	0.8994	0.02975	(S)-Malate	-23.109282	-	-	-	162.1525	135.091578
POS05237	2527202.573	2349114.612	-0.10542	0.687056	0.8457	0.11879	1,7-diphospho-1-epi-valienol	20.847112	-	-	-	161.994	337.141284
POS05241	81550.94451	85119.30055	0.061785	0.832781	0.9237	0.01922	Bupranolol	13.562851	-	-	-	161.986	272.793963
POS05247	89807.18262	70364.81543	-0.35198	0.123601	0.4411	0.10309	N1-(2-Hydroxyethyl)flurazepam	1.1205885	-	-	-	161.974	347.790665
POS05255	135123.7587	108286.3158	-0.31943	0.311765	0.6218	0.09325	3-oxobrimonidine	13.841913	-	-	-	161.6575	311.16157
POS05265	1275633.858	1228633.305	-0.05416	0.756692	0.8869	0.03022	3-(Phosphoacetylamido)-L-alanine	8.6029025	-	-	-	161.3125	243.13316
POS05268	4890903.187	4440274.892	-0.13945	0.219207	0.5401	0.39532	Pyrazinoic acid	19.584622	-	-	-	161.3095	125.107207
POS05269	1065712.622	417855.9604	-1.35074	0.075697	0.3687	0.60915	5,6-Dihydrothymine	15.569654	-	-	-	161.309	129.138472
POS05270	59501.88683	52616.86287	-0.17741	0.461156	0.7119	0.05078	Vecuronium	-0.7996784	-	-	-	161.308	558.834231
POS05279	137516.5774	114492.7677	-0.26435	0.348591	0.636	0.09185	UDP-N-acetylmuramoyl-L-alanyl-D-glutamate	-23.594229	-	-	-	161.016	880.594723
POS05280	32825.24274	19552.54641	-0.74745	0.054343	0.3251	0.08655	Pencycuron	-9.1343088	-	-	-	161.014	329.840073
POS05294	417756.5891	324656.2961	-0.36375	0.02013	0.2381	0.2561	3-Phospho-D-glyceroyl phosphate	-7.4246424	-	-	-	160.6505	267.042401
POS05295	3715940.278	3726959.843	0.004272	0.991052	0.9956	0.10854	3-Oxalomalate	-8.7747023	-	-	-	160.6385	207.112468
POS05296	3695618.144	4239463.782	0.198066	0.641487	0.8239	0.21635	Stipitatote	-17.73039	-	-	-	160.637	209.128086
POS05301	3453895.082	3385887.351	-0.02869	0.924641	0.9659	0.01013	Oxalosuccite	14.415585	-	-	-	160.391	191.117617
POS05306	1157778.121	987333.5463	-0.22975	0.02957	0.2674	0.33844	Dopaquinone	6.0520068	-	-	-	160.302	196.180558
POS05313	2179494.963	2189934.699	0.006894	0.972882	0.9873	0.07121	Citrate	13.257476	-	-	-	160.1375	193.133324
POS05316	8142911.775	7029857.514	-0.21205	0.329801	0.6326	0.60384	Cyanidin	3.3242361	-	-	-	159.702	288.252431
POS05321	59546.33986	55613.16174	-0.09859	0.856408	0.9349	0.0076	alpha-1,5-L-Arabinotetraose	-20.970587	-	-	-	159.6345	547.469617
POS05328	378246.7115	329382.5768	-0.19956	0.54109	0.7628	0.08527	Metocurine Iodide	-22.267045	-	-	-	159.618	907.614989
POS05331	7250184.013	4170809.92	-0.79769	0.034345	0.2826	1.38692	2,3,6-Trihydroxypyridine	11.661818	-	-	-	159.4625	128.106859

POS05336	5885590.994	8307115.461	0.49716	0.632314	0.8181	0.72745	Cytosine	23.096179	-	-	-	159.283	112.111843
POS05340	217848.1689	183890.7718	-0.24447	0.519425	0.7502	0.07892	({3,4,5-trihydroxy-6-[3,5,7-trihydroxy-2-(2,4,5-trihydroxyphenyl)-3,4	15.710163	-	-	-	158.9755	549.485893
POS05344	761214.5115	563470.6866	-0.43396	0.189188	0.5161	0.28107	Heterophylliin E	-13.027996	-	-	-	158.9655	909.630739
POS05346	55998.80998	43260.43829	-0.37235	0.514429	0.7469	0.05257	Pradimicinone I	-1.8289009	-	-	-	158.959	550.488572
POS05348	2832222.44	2880404.147	0.024337	0.924726	0.9659	0.03631	3-Sulfopyruvate	4.5779559	-	-	-	158.947	169.133346
POS05355	3539143.574	3036327.398	-0.22107	0.734053	0.8739	0.27411	6-Imino-5-oxocyclohexa-1,3-dienecarboxylate	-4.716798	-	-	-	158.624	152.126064
POS05356	6656442.446	5996260.724	-0.15069	0.319965	0.6276	0.5827	Threote	-24.296656	-	-	-	158.6235	137.10727
POS05357	10966893.89	12096535.35	0.141439	0.711563	0.8592	0.49064	N-(2,3-Dihydroxybenzoyl)-L-serine	6.3532298	-	-	-	158.614	242.206209
POS05359	55625.82318	60281.07095	0.11595	0.801296	0.9096	0.01802	TG(18:0/22:4(7Z,10Z,13Z,16Z)/o-18:0)	9.6616888	-	-	-	158.453	926.578219
POS05361	740094.5739	676448.5583	-0.12973	0.532842	0.7588	0.10919	12,13,14-trihydroxy-9-oxo-8,17-dioxatetracyclo[8.7.0.0	-0.9519292	-	-	-	158.2905	343.265951
POS05362	1283215.484	1035213.562	-0.30984	0.128422	0.4471	0.36063	Sagerinic acid	8.0334156	-	-	-	158.288	721.642766
POS05364	169340.1627	134712.3581	-0.33004	0.299041	0.6119	0.11747	5,7-dihydroxy-3-[5-hydroxy-2-(4-hydroxy-3-methoxyphenyl)-4-oxo-7	-18.056418	-	-	-	158.287	735.657011
POS05368	1343122.901	1431868.845	0.092308	0.814777	0.9158	0.11935	(3-aminopropoxy)sulfonic acid	-17.152972	-	-	-	158.2785	156.174615
POS05369	1030640.891	1209276.413	0.230602	0.814986	0.9159	0.03325	5,6-Indolequinone-2-carboxylic acid	9.0566033	-	-	-	158.2755	192.149308
POS05386	4720760.385	3336907.146	-0.50051	0.090118	0.3896	0.85225	3-Fluoro-cis,cis-mucote	-0.1156489	-	-	-	157.9515	161.107158
POS05388	75581.57904	93352.03895	0.304647	0.544691	0.7653	0.05709	TG(18:1(11Z)/22:2(13Z,16Z)/o-18:0)	9.1253813	-	-	-	157.948	928.593741
POS05390	587204.4037	467846.7679	-0.32783	0.122554	0.4403	0.2519	hydroxyitraconazole	8.1874971	-	-	-	157.9455	722.646185
POS05394	15131365.29	11281975.02	-0.42352	0.083219	0.3796	1.50961	Bromobenzene	-18.226427	-	-	-	157.787	158.012315
POS05410	307473.6064	288144.9061	-0.09367	0.74943	0.8825	0.04247	Casuarictin	7.2981152	-	-	-	157.4675	937.659512
POS05412	45027.38963	34012.16257	-0.40475	0.300066	0.6129	0.06126	Proanthocyanidin A2	7.0292822	-	-	-	157.311	577.515729
POS05421	79178.49132	138890.9087	0.810772	0.39129	0.661	0.14413	Manniflavanone	0.4087422	-	-	-	157.287	591.495418
POS05422	211097.1183	331200.9236	0.6498	0.445276	0.7005	0.19298	TG(16:1(9Z)/24:1(15Z)/o-18:0)	9.7237395	-	-	-	157.2865	930.610316
POS05434	894345.372	994236.0301	0.152756	0.761673	0.8887	0.13306	TG(16:0/24:1(15Z)/o-18:0)	10.640678	-	-	-	156.9595	932.62719

POS05435	947438.6242	987028.7065	0.05906	0.962053	0.9827	0.09629	Potassium iodide	15.413803	-	-	-	156.9575	167.012635
POS05449	1506477.903	1438951.541	-0.06616	0.833091	0.9237	0.05491	TG(16:0/24:0/o-18:0)	10.072463	-	-	-	156.7995	934.642681
POS05450	778916.604	748990.9601	-0.05652	0.854602	0.9339	0.02909	Alnusiin	10.098375	-	-	-	156.7895	935.646215
POS05460	168924.7701	166270.6805	-0.02285	0.958127	0.981	#######	Luteoskyrin	8.8769833	-	-	-	156.618	575.500876
POS05468	110611.8867	117565.1383	0.087954	0.804984	0.9107	0.02907	D-Erythroascorbic acid 1'-a-D-glucoside	-14.991904	-	-	-	156.605	309.241356
POS05470	137356.4285	104805.4929	-0.39021	0.390493	0.6605	0.0951	4,6-Dichloro-3-methyl-cis-1,2-dihydroxycyclohexa-3,5-dione	-19.791095	-	-	-	156.58	196.046616
POS05478	128200.7586	4382.880058	-4.87038	0.334374	0.6326	0.18333	11,15-dihydroxy-17,18-dimethoxy-6,8,20-trioxapentacyclo[10.8.0.0i	14.057692	-	-	-	156.283	371.325482
POS05483	92243.703	123669.2058	0.422964	0.587469	0.7902	0.07001	TG(18:4(6Z,9Z,12Z,15Z)/24:1(15Z)/o-18:0)	-14.152339	-	-	-	156.2745	952.593809
POS05487	1262748.642	948080.8385	-0.41349	0.127936	0.4468	0.39792	3,4,5-trihydroxy-6-{[3-hydroxy-2-(hydroxymethyl)-2	-17.178097	-	-	-	156.258	311.256947
POS05489	181497.5244	126340.3955	-0.52263	0.063011	0.3422	0.18987	4-Bromo-3,5-cyclohexadiene-1,2-dione	-13.382908	-	-	-	156.11	187.995574
POS05490	4347181.638	2624798.703	-0.72787	0.161114	0.4886	0.8894	Urate radical	19.834236	-	-	-	156.1085	168.112891
POS05493	204772.0096	255676.9442	0.320304	0.288337	0.603	0.12766	TG(18:1(11Z)/24:1(15Z)/o-18:0)	-14.93286	-	-	-	155.951	958.640976
POS05496	181181.1931	222399.833	0.295722	0.402113	0.6697	0.10324	TG(18:2(9Z,12Z)/24:1(15Z)/o-18:0)	-15.372442	-	-	-	155.948	956.624586
POS05499	170586.5759	220842.8191	0.372516	0.492906	0.7328	0.10229	TG(18:3(6Z,9Z,12Z)/24:1(15Z)/o-18:0)	-14.812272	-	-	-	155.947	954.609151
POS05500	230338.9466	235253.7736	0.030459	0.919341	0.9634	0.01164	TG(18:0/24:1(15Z)/o-18:0)	-15.661885	-	-	-	155.947	960.656246
POS05505	935813.001	814081.4698	-0.20105	0.148917	0.4745	0.24411	Monomethyl phenylphosphonate	15.966252	-	-	-	155.942	173.128325
POS05511	451492.2471	405861.7388	-0.15371	0.593041	0.7922	0.1011	3,5-Dichloro-2-methylmucote	-4.5407944	-	-	-	155.7855	226.032355
POS05517	318636.2177	259625.9086	-0.29548	0.290017	0.6044	0.14731	TG(18:0/24:0/o-18:0)	-15.359129	-	-	-	155.616	962.672506
POS05519	6159110.174	6386777.376	0.052366	0.87504	0.9448	0.11198	2-Amino-3,4-dihydroxypentanedioic acid	15.31402	-	-	-	155.611	180.13812
POS05520	201021.8925	146804.0157	-0.45346	0.227901	0.5492	0.15002	Cyanophos	-1.0370994	-	-	-	155.605	244.226424
POS05522	3628116.654	2714173.839	-0.41871	0.368834	0.6479	0.50162	N-Dimethyl-2-aminoethylphosphote	-9.7952731	-	-	-	155.4625	154.122477
POS05529	2712875.402	2363150.78	-0.19911	0.212532	0.5357	0.38119	[5-(Aminomethyl)furan-3-yl]methyl phosphate	21.83979	-	-	-	155.282	208.1329
POS05537	201106.3107	207824.91	0.04741	0.789191	0.9032	0.03937	3,4,5-trihydroxy-6-({5-hydroxy-12,12-dimethyl-6-oxo-4-phenyl-3,11,14	10.376438	-	-	-	155.26	529.47876

POS05539	653110.8069	647940.3397	-0.01147	0.937584	0.9719	0.00497	4-O-(beta-L-Arabinofuranosyl-(1->2) beta-L	-10.165815	-	-	-	155.2555	528.475614
POS05540	149323.8666	67925.49492	-1.13642	0.063731	0.3438	0.20558	Vinclozolin	8.358673	-	-	-	155.248	287.120468
POS05542	322684.9	226018.5739	-0.51368	0.40782	0.673	0.1434	3,4-Dihydroxy-L-phenylalanine	5.085546	-	-	-	154.949	198.196179
POS05548	212963.0928	123042.6961	-0.79144	0.017349	0.2275	0.25557	4-Chloromethandienone	-23.684354	-	-	-	154.77	335.879345
POS05556	2533104.153	1900902.99	-0.41422	0.204541	0.5283	0.51349	N-Acetyl-L-aspartate	-19.94128	-	-	-	154.28	176.143184
POS05562	710944.207	517571.7673	-0.45798	0.0372	0.2891	0.36328	Bromoxynil	4.1157978	-	-	-	154.25	277.921216
POS05578	36121282.09	34644353.17	-0.06023	0.734904	0.8744	0.15725	Trimethylselenonium	-0.1421474	-	-	-	152.944	125.070859
POS05581	917370.9603	859852.811	-0.09342	0.858042	0.9353	0.05122	Vicianose	-16.737501	-	-	-	152.9225	313.27255
POS05586	348837.2397	286914.1656	-0.28193	0.015584	0.2226	0.21094	3'-Hydroxydiclofenac	-6.1702723	-	-	-	152.871	313.153351
POS05588	1288484.48	1060183.438	-0.28136	0.65823	0.8318	0.16248	Aminosalicylate sodium anhydrous	10.112082	-	-	-	152.723	176.126247
POS05603	96647.10964	90646.08587	-0.09248	0.650968	0.829	0.03136	Bowdichione	9.3457206	-	-	-	150.919	299.257064
POS05611	751098.993	959303.4845	0.352984	0.286664	0.6021	0.26172	Quinoxaline	16.878558	-	-	-	150.596	131.156073
POS05613	987948.8835	956520.1481	-0.04664	0.810519	0.9135	0.04068	Melizame	-7.2967562	-	-	-	150.569	179.154077
POS05618	702572.6916	507328.3819	-0.46973	0.070075	0.3569	0.33554	Lamotrigine	1.5061229	-	-	-	150.234	257.098662
POS05619	4388865.239	2367578.812	-0.89044	0.044238	0.3084	1.23647	Pterin	-4.8806751	-	-	-	150.222	164.14328
POS05621	108061.3869	88882.19696	-0.28188	0.199645	0.5255	0.08548	Spirolaurenone	-5.0261199	-	-	-	150.0405	300.252273
POS05622	185382.0738	128067.7891	-0.53359	0.065948	0.3485	0.17991	Scopoletin	11.795427	-	-	-	149.925	193.177643
POS05625	917517.1385	818293.1761	-0.16512	0.766839	0.8917	0.11554	sn-Glycero-3-phosphoethanolamine	2.0543269	-	-	-	149.8665	216.149219
POS05633	137668.4817	130885.3539	-0.07289	0.778962	0.8985	0.01036	Pyrifenox	12.450806	-	-	-	149.133	296.174852
POS05635	140616.2482	147723.7915	0.071139	0.742139	0.8785	0.02274	Nocardicin E	14.024381	-	-	-	148.884	400.367077
POS05636	58530.38517	39803.67388	-0.55628	0.199035	0.525	0.09589	Dinocton 6	12.56724	-	-	-	148.873	355.36683
POS05638	742793.4716	666438.0267	-0.15649	0.048542	0.3158	0.216	5,6-Dihydrothymine	15.482424	-	-	-	148.8375	129.13846
POS05651	245840.6243	271146.797	0.141351	0.724341	0.8677	0.06638	Metamitron	-1.719331	-	-	-	147.533	203.219529

POS05656	2464148.639	2081331.207	-0.24358	0.488676	0.73	0.27705	Allantoate	2.6390687	-	-	-	147.192	177.138441
POS05663	380246.8309	308355.9462	-0.30234	0.607103	0.8029	0.10895	Pyrimidodiazepine	-9.1944834	-	-	-	146.8	222.221143
POS05674	185285.9104	149806.9751	-0.30665	0.367271	0.6468	0.09374	3,5-Dinitrosalicylic acid	-7.4276624	-	-	-	146.207	229.121482
POS05675	329937.1254	121156.7058	-1.44532	0.315299	0.6246	0.2524	6-Acetamido-3-oxohexanoate	1.2740679	-	-	-	146.206	188.200615
POS05676	138848.0068	119109.872	-0.22121	0.527168	0.7557	0.07063	M1	11.297345	-	-	-	146.199	305.136712
POS05678	214820.9206	180059.7615	-0.25466	0.213527	0.5364	0.11796	Selenodiglutathione	-10.379585	-	-	-	146.195	692.591198
POS05679	296944.7112	345089.3337	0.216776	0.513435	0.7463	0.08703	3-Phospho-D-glycerate	-10.219837	-	-	-	146.1605	187.062575
POS05683	290373.0606	237726.7227	-0.2886	0.521619	0.7515	0.09546	NTP	-8.8128912	-	-	-	146.1345	389.100656
POS05686	283324.3281	214207.8761	-0.40344	0.03953	0.296	0.21318	Dimethylenetriurea	1.4220198	-	-	-	146.1125	205.194767
POS05689	764606.1291	1082953.214	0.502182	0.425933	0.6859	0.22108	erythro-3-Hydroxy-Ls-aspartate	20.151432	-	-	-	146.069	150.112381
POS05693	1632427.935	1337543.096	-0.28743	0.235671	0.5559	0.32297	3-hydroxy-2-isobutyrate	14.997505	-	-	-	145.8695	104.106823
POS05696	1081940.358	551409.8514	-0.97242	0.304178	0.6167	0.48159	Calcium propiote	-9.6499651	-	-	-	145.849	187.22468
POS05699	83990.07828	73714.51556	-0.18827	0.495318	0.7341	0.06129	Triphenyl phosphate	-7.029944	-	-	-	145.65	327.288083
POS05705	116648.9056	76568.42549	-0.60735	0.026275	0.2576	0.16484	Bisnorbadioquinone A	9.7080529	-	-	-	145.2795	651.511892
POS05709	125847.9098	99512.38991	-0.33873	0.124389	0.4427	0.11857	Norbadione A	-13.251692	-	-	-	145.1435	679.506685
POS05718	1754439.227	1171256.984	-0.58295	0.008338	0.1881	0.68205	3-Dehydroquite	-21.14247	-	-	-	144.84	191.153956
POS05722	711772.5469	765890.1262	0.105721	0.730679	0.8715	0.04054	Piperazine phosphate	2.8914279	-	-	-	144.502	203.153961
POS05724	423964.0391	287357.6857	-0.56109	0.265474	0.5818	0.21131	Lathyrine	-9.9424532	-	-	-	144.437	183.185365
POS05729	220114.2862	186476.4779	-0.23926	0.177592	0.5035	0.13372	Vitisin A	-18.0472	-	-	-	143.8285	562.465444
POS05737	170302.1899	196111.05	0.203574	0.738213	0.8761	0.03952	3-[3-hydroxy-5-methoxy-4-(sulfooxy)phenyl]prop-2-enoic acid	-17.954086	-	-	-	143.467	291.242066
POS05742	181194.3867	191554.5584	0.080217	0.625132	0.8134	0.04338	Domoic acid	-4.6247792	-	-	-	143.1525	312.336137
POS05743	136398.3412	142394.4577	0.062067	0.621389	0.8122	0.03637	Dichlofluanid	-3.7762346	-	-	-	143.147	334.236218
POS05749	6045172.651	4822675.917	-0.32595	0.23491	0.5558	0.63992	2,3,6-Trihydroxypyridine	11.725598	-	-	-	142.804	128.106867

POS05750	112148.1461	112623.7159	0.006105	0.971171	0.9872	0.00371	Agrocin 84	-0.2454643	-	-	-	142.803	703.506504
POS05755	84286.29058	79698.23888	-0.08075	0.766084	0.8916	0.02073	Wyerone	20.206167	-	-	-	142.4095	259.281795
POS05757	74884.41812	95385.36992	0.349102	0.431223	0.6899	0.08722	Famphur	9.0985582	-	-	-	142.3375	326.351737
POS05760	216093.512	145142.6784	-0.57418	0.089882	0.3895	0.19011	L-Arginine phosphate	-13.278427	-	-	-	142.148	255.184801
POS05761	1010736.471	789182.503	-0.35698	0.027242	0.2602	0.38781	Formothion	14.380137	-	-	-	142.1465	258.278476
POS05764	217397.9502	207055.1518	-0.07032	0.709777	0.8583	0.0516	[2,6-dihydroxy-4-({[3-hydroxy-6-(hydroxymethyl)-2,5-	-20.156205	-	-	-	142.1345	717.522834
POS05765	101276.4983	91118.86791	-0.15248	0.416602	0.6793	0.06026	3,4,5-trihydroxy-6-({15-hydroxy-11,17,18-trimethoxy-13-oxo-6,8,20-	24.159795	-	-	-	142.132	577.484204
POS05766	604947.2604	545173.6164	-0.15009	0.311892	0.6219	0.16863	N(omega)-(ADP-D-ribosyl)-L-arginine	15.686595	-	-	-	142.0905	716.5199
POS05769	228996.7032	185306.3554	-0.30541	0.28863	0.603	0.13392	Ditalimfos	-3.7575154	-	-	-	141.753	300.288752
POS05770	1006307.557	796400.896	-0.3375	0.084312	0.3821	0.34344	Theophylline	-8.9911783	-	-	-	141.694	181.169657
POS05775	21527.13141	59563.87005	1.468281	0.321793	0.629	0.13013	Triaziquone	-16.326578	-	-	-	141.451	232.254001
POS05776	1114379.347	1479329.169	0.408703	0.561997	0.776	0.33959	Asulam	10.250443	-	-	-	141.44	231.250637
POS05778	8518658.232	11225897.08	0.398133	0.567773	0.7785	0.91813	N-Hydroxy-MeIQx	9.6035192	-	-	-	141.371	230.247378
POS05783	93902.0682	196303.1423	1.063854	0.462275	0.7127	0.15657	DHAP(10:0)	17.486698	-	-	-	141.113	325.320148
POS05787	529468.2711	409770.7519	-0.36973	0.093252	0.396	0.28125	5-Hydroxysulfamethoxazole	-22.097512	-	-	-	141.0685	270.278326
POS05789	56109.28751	48188.72445	-0.21954	0.265503	0.5818	0.06497	Manniflavanone	7.1701618	-	-	-	140.7895	591.49941
POS05790	136821.7282	148099.758	0.114272	0.673407	0.8391	0.03051	fluvoxamino acid	11.388119	-	-	-	140.778	319.302501
POS05792	1088336.645	1064672.136	-0.03172	0.838749	0.9266	0.06708	Lettowianthine	-9.4110223	-	-	-	140.767	318.299191
POS05793	531053.4818	354777.3367	-0.58194	0.200048	0.5255	0.27848	sn-Glycerol 3-phosphate	-1.2883282	-	-	-	140.75	173.080755
POS05795	691422.8906	614414.5968	-0.17036	0.130179	0.4485	0.19683	2,7-Anhydro-alpha-N-acetylneuraminic acid	2.1044147	-	-	-	140.663	292.26249
POS05801	374405.3337	412370.8931	0.139342	0.38865	0.6595	0.11359	N-Acetoxy-4-aminobiphenyl	10.008953	-	-	-	140.4045	228.268051
POS05812	363831.669	321298.105	-0.17936	0.053148	0.3235	0.16475	Glutaminylhistidine	-5.0333887	-	-	-	139.7675	284.293851
POS05815	592244.5444	767093.8309	0.37321	0.128782	0.4471	0.27825	2-(3-Carboxy-3-(methylammonio)propyl)-L-histidine	-23.486727	-	-	-	139.756	272.293905

POS05825	187045.2614	155534.5202	-0.26615	0.153335	0.4811	0.11808	Reduced coenzyme F420	-8.4456432	-	-	-	139.082	776.609226
POS05827	133061.6184	70294.62483	-0.92061	0.354376	0.6384	0.15413	(2-benzyl-3-oxopropoxy)sulfonic acid	-4.3730042	-	-	-	139.029	245.266208
POS05828	1245776.489	746173.222	-0.73946	0.2136	0.5364	0.48666	Prolyl-Gamma-glutamate	-16.858745	-	-	-	138.9855	244.262876
POS05830	876527.3863	724711.9378	-0.27439	0.037334	0.2898	0.31633	Coenzyme F420	-2.3952772	-	-	-	138.7545	774.598024
POS05835	239057.6283	235805.8267	-0.01976	0.955422	0.9803	0.0091	Pencozole	12.770548	-	-	-	138.393	285.195206
POS05837	514251.2497	406672.2879	-0.33861	0.021871	0.2452	0.27684	Luteolin 7-O-[beta-D-glucuronosyl-(1->2)-beta-D-glucuronide] 4'-O	20.477095	-	-	-	138.3755	815.632657
POS05840	63216.58621	58114.87931	-0.1214	0.448888	0.7031	0.03284	Clofentezine	-23.496673	-	-	-	138.055	304.146254
POS05843	561166.3287	761349.2254	0.44013	0.154218	0.4823	0.27112	Letrozole	-1.6554753	-	-	-	138.047	286.309504
POS05845	135466.3577	121901.0037	-0.15222	0.719256	0.8647	0.04019	{[(4E)-5-(4-methoxyphenyl)-3-oxopent-4-en-1-	19.590074	-	-	-	137.815	287.312885
POS05852	374828.2703	283284.8096	-0.40398	0.000297	0.0721	0.29234	Theogallinin	-21.285374	-	-	-	137.702	801.616935
POS05853	1415055.066	1247537.634	-0.18177	0.094456	0.3979	0.29981	Phosfolan	-11.044724	-	-	-	137.699	256.299157
POS05854	284753.9552	253294.8981	-0.1689	0.406114	0.6721	0.10266	Longifolonine	-10.499012	-	-	-	137.6875	298.309455
POS05856	73521.70203	44947.47436	-0.70993	0.280553	0.5965	0.12088	Phloretin	2.3253621	-	-	-	137.614	275.276614
POS05860	168198.9706	128560.0045	-0.38773	0.216847	0.5382	0.13784	Tyrosyl-Glutamate	24.0902	-	-	-	137.381	310.309328
POS05861	634568.2648	481243.3096	-0.39901	0.022293	0.246	0.32468	5-Sulfoxymethylfurfural	24.102152	-	-	-	137.3755	207.185246
POS05862	274141.5554	289072.1354	0.076509	0.590948	0.7918	0.03565	2,3,9,10-Tetrahydroxyberbine	-11.375975	-	-	-	137.372	300.325072
POS05864	412398.1704	125308.7693	-1.71855	0.046158	0.3134	0.4479	N(beta)-Epoxysuccimoyl-DAP	15.054764	-	-	-	137.357	218.189846
POS05870	1043770.504	874770.0172	-0.25483	0.023114	0.2478	0.34476	2',3',6'-Tris-O-(3,4,5-trihydroxybenzoyl)-3'-Glucosyl-2',4',6'-	-7.6607346	-	-	-	137.03	787.601351
POS05873	144777.5714	116775.3215	-0.3101	0.085569	0.3848	0.13656	Avenanthramide C	-4.0112387	-	-	-	136.7235	316.283512
POS05874	153960.0145	126949.3057	-0.2783	0.01538	0.2226	0.13875	Xenognosin A	-4.4596204	-	-	-	136.604	257.302534
POS05880	74422.9836	64735.81983	-0.20118	0.414829	0.6784	0.04745	5-Hydroxy-1-(4-sulfophenyl)-4-[(E)-(4-sulfophenyl)diazenyl]-1H	15.866316	-	-	-	136.0245	469.432709
POS05885	700862.7635	425080.4145	-0.7214	0.189363	0.5161	0.34214	1-Carboxyvinyl carboxyphosphote	24.411825	-	-	-	135.998	197.064163
POS05891	96812.72198	110440.6688	0.190003	0.780709	0.8992	0.0313	Cyanotriphenylborate	-16.321397	-	-	-	135.66	292.132425

POS05893	65961.72989	57985.55904	-0.18594	0.698057	0.8513	0.03969	1,2-Bis(4-hydroxy-3-methoxyphenyl)ethylene	-21.334843	-	-	-	135.636	273.297267
POS05897	130856.813	187051.282	0.515445	0.205021	0.5287	0.16487	Sulfamethoxazole	-5.4828221	-	-	-	135.549	254.283488
POS05901	205828.8591	232277.0204	0.174401	0.745934	0.8803	0.01969	5'-Methylthioadenosine	17.036067	-	-	-	134.7525	298.345842
POS05904	241445.9939	138116.7882	-0.80581	0.109922	0.4215	0.23745	Fluometuron	-4.1333143	-	-	-	134.634	233.208717
POS05905	627773.1111	517098.6738	-0.2798	0.015154	0.2226	0.28034	N-Acetyl-L-2-amino-6-oxopimelate	-19.12183	-	-	-	134.5995	232.205456
POS05910	76533.45174	99881.47601	0.384127	0.241466	0.5615	0.08873	Chicoric acid	4.0645376	-	-	-	133.652	475.380305
POS05913	61963.32287	59892.2888	-0.04904	0.713388	0.8606	0.02285	Rhododendrin	-16.155142	-	-	-	133.466	329.359572
POS05914	35569.88353	53514.37996	0.58927	0.399759	0.6679	0.07538	Indol-3-ylacetyl-myo-inositol L-arabinoside	-3.0177859	-	-	-	133.458	470.44506
POS05916	221661.81	221089.8909	-0.00373	0.986555	0.9932	0.00387	2-Hydroxy-6-oxono-2,4-diene-1,9-dioate	-20.425571	-	-	-	133.377	215.175002
POS05917	442274.797	426136.3326	-0.05363	0.655596	0.8306	0.04692	Protodeoxyviolaceinic acid	5.4248979	-	-	-	133.356	342.371828
POS05921	533532.2612	489497.291	-0.12427	0.794411	0.9055	0.08734	1-phthaleneacetic acid sodium salt	24.751366	-	-	-	132.941	209.20083
POS05922	738549.2315	445704.455	-0.72861	0.001023	0.0749	0.52342	Calcium L-aspartate	-15.928154	-	-	-	132.9395	172.16935
POS05923	619389.577	511470.8184	-0.2762	0.577594	0.7844	0.15573	Albendazole-beta-hydroxysulphone	11.823362	-	-	-	132.9185	314.340581
POS05924	75154.94988	132517.1068	0.818239	0.307821	0.619	0.10776	dTDP-4-amino-2,3,4,6-tetradeoxy-D-glucose	-4.6048418	-	-	-	132.459	516.350904
POS05925	2278844.339	1930471.537	-0.23935	0.038231	0.2927	0.47952	[5-(Aminomethyl)furan-3-yl]methyl phosphate	21.894545	-	-	-	132.431	208.132911
POS05926	119500.1906	111819.0589	-0.09585	0.691938	0.8473	0.0426	2-(3,4-dihydroxy-5-methoxyphenyl)-3,5,7-trihydroxy-1chromen-1	1.748573	-	-	-	131.9195	318.278031
POS05929	204200.5589	201666.3193	-0.01802	0.908045	0.9578	0.01329	Taxiphyllin	-19.330071	-	-	-	131.566	312.288559
POS05931	168930.4643	179044.6519	0.08389	0.642053	0.824	0.03264	Buntanine	-12.432298	-	-	-	131.3935	356.387258
POS05933	236032.779	371178.8125	0.653127	0.127482	0.4463	0.25488	N(omega)-Nitro-L-arginine	-1.226755	-	-	-	131.073	220.205508
POS05936	833096.0078	606664.1703	-0.45758	0.294144	0.6083	0.26339	Zonisamide	-4.2266714	-	-	-	130.7075	213.23208
POS05937	152704.9652	148757.3461	-0.03779	0.883231	0.9474	0.02208	5-Carboxy-2-oxohept-3-enedioate	8.8643847	-	-	-	130.545	217.154093
POS05938	98505.90833	77971.62489	-0.33726	0.362477	0.6443	0.0753	Dapdiamide B	-2.6632598	-	-	-	130.5435	315.344039
POS05939	204052.9073	159530.9557	-0.35511	0.242668	0.5618	0.15127	1-(Methylnitrosoamino)-4-(3-pyridinyl)-1,4-butanediol	3.1257633	-	-	-	130.541	226.252381

POS05940	44050.68131	33372.75499	-0.40049	0.190719	0.5176	0.05826	dTDP-4-dehydro-beta-L-rhamnose	14.610532	-	-	-	130.178	547.328959
POS05950	983456.6554	1098113.141	0.159093	0.68454	0.845	0.19091	Selenomethionine se-oxide	22.507166	-	-	-	129.2	213.122051
POS05951	3788270.046	2667697.817	-0.50594	0.034123	0.2814	0.83578	Phosphocreatine	-12.093165	-	-	-	129.181	212.117824
POS05952	404352.1924	314999.8944	-0.36026	0.085311	0.3847	0.21148	D-Glucosamine	-21.957267	-	-	-	129.1765	180.174442
POS05953	850896.4209	732291.7721	-0.21656	0.046045	0.3134	0.28048	Fraxetin	-11.134959	-	-	-	129.174	209.172459
POS05967	140422.418	126816.7202	-0.14703	0.662525	0.8336	0.05337	5'-Deoxy-5-fluorocytidine	24.637713	-	-	-	128.5475	246.221018
POS05968	2529499.774	1911316.258	-0.40429	0.14238	0.4668	0.56173	Lichenin	0.0233036	-	-	-	128.503	163.14788
POS05972	730163.2153	412890.7576	-0.82246	0.19002	0.5169	0.36003	Flurochloridone	7.9398721	-	-	-	128.338	313.124855
POS05974	963875.8527	907443.9217	-0.08704	0.585544	0.789	0.11696	Morphinone	4.1329466	-	-	-	128.166	284.330248
POS05976	11787155.07	10667139.73	-0.14404	0.295383	0.6091	0.64902	Cyazine	19.651585	-	-	-	128.1635	241.704707
POS05977	706558.9526	416464.3027	-0.76262	0.162321	0.4895	0.35796	Lormetazepam	24.263503	-	-	-	128.161	336.200409
POS05981	2092855.561	1748407.242	-0.25943	0.035531	0.2845	0.49279	3-Fluoro-cis,cis-mucote	-0.1443548	-	-	-	127.8115	161.107153
POS05984	4319886.417	4064621.373	-0.08787	0.518911	0.7498	0.1813	2,3,6-Trihydroxypyridine	11.653615	-	-	-	127.306	128.106858
POS05986	97601.87795	71563.05966	-0.44769	0.012569	0.2092	0.13357	Furaneol 4-(6-malonylglucoside)	-14.046339	-	-	-	126.782	377.314691
POS05987	2257240.77	1774680.202	-0.347	0.278212	0.5939	0.40452	Citalopram alcohol	12.658043	-	-	-	126.781	312.361318
POS05989	276579.8317	187659.9792	-0.55957	0.061957	0.3409	0.21988	Neosaxitoxin	17.771679	-	-	-	126.436	316.29878
POS05992	545492.1722	368768.2224	-0.56484	0.093846	0.397	0.31407	Flumazenil	10.294373	-	-	-	126.021	304.298799
POS05994	76524.56434	45684.73869	-0.74421	0.256502	0.5745	0.10825	beta-Cotonefuran	1.2948462	-	-	-	125.781	305.302271
POS06003	1899583.201	1276074.453	-0.57397	0.447527	0.7021	0.3852	3-carboxy-2,3-dihydroxypropanoate	-13.593249	-	-	-	124.7495	150.08425
POS06004	12286314.37	10028547.74	-0.29294	0.599848	0.7973	0.55249	Cytosine	23.314814	-	-	-	124.731	112.111867
POS06007	3052561.638	2530545.432	-0.27057	0.192779	0.519	0.45869	Oxalosuccite	14.298919	-	-	-	124.27	191.117595
POS06008	168421.9864	128975.5749	-0.38498	0.010187	0.1952	0.182	Maculosine	8.0316024	-	-	-	124.235	332.329938
POS06009	6924058.955	4661723.217	-0.57075	0.096041	0.4006	1.00598	cis-4-Carboxymethylenebut-2-en-4-olide	8.7865487	-	-	-	124.076	141.102108

POS06013	32804.43506	10870.6971	-1.59345	0.411455	0.6762	0.07474	4,4-Bis[4-(acetyloxy)phenyl]3-hexanone	-9.2304089	-	-	-	123.7595	369.426876
POS06014	638135.6771	477115.5482	-0.41952	0.059268	0.3362	0.31316	Diphenylcarbazide	12.888615	-	-	-	123.725	243.286899
POS06019	51694.96138	25977.99969	-0.99273	0.242134	0.5616	0.09989	Quinoline-3-carboxamides	1.097959	-	-	-	123.056	360.360871
POS06026	149168.4528	121287.2143	-0.29851	0.350509	0.6364	0.10345	2,3,4,7,8,9,15,22,23,28-decahydroxy-14-(hydroxymethyl)	15.707268	-	-	-	122.717	757.55316
POS06027	190929.2184	84583.32015	-1.17459	0.27546	0.5921	0.21557	(2-benzyl-3-oxopropoxy)sulfonic acid	-4.4148089	-	-	-	122.374	245.266198
POS06028	1664704.441	868065.182	-0.93939	0.213631	0.5364	0.62337	Prolyl-Gamma-glutamate	-16.901523	-	-	-	122.373	244.262865
POS06035	2549241.089	1248142.001	-1.03029	0.102488	0.4087	0.78707	2-Aminomucote	-6.0696801	-	-	-	122.041	158.130423
POS06041	535498.0142	382858.627	-0.48407	0.027418	0.2606	0.34712	Melicopine	-24.803774	-	-	-	121.9305	314.304205
POS06044	70152.70726	30851.99315	-1.18514	0.20421	0.5278	0.12307	Thifensulfuron-methyl	-17.119611	-	-	-	121.848	388.392145
POS06049	501043.4793	494363.8526	-0.01936	0.963256	0.983	0.03878	Glutaminylhistidine	-4.8909166	-	-	-	121.5305	284.293891
POS06051	109421.8081	103703.4492	-0.07744	0.629459	0.8159	0.03385	Ditalimfos	-4.0179793	-	-	-	121.365	300.288674
POS06052	7117771.716	8017275.153	0.171686	0.874513	0.9445	0.05084	Serylglutamic acid	-0.2456511	-	-	-	121.351	234.205819
POS06053	144873.6087	126325.605	-0.19765	0.470397	0.7182	0.07151	Cyanophos	-0.9651085	-	-	-	121.3485	244.226442
POS06055	884207.2318	954438.086	0.110267	0.911067	0.9581	0.00696	(R)-(Homo)3-citrate	-5.9564066	-	-	-	121.343	235.209182
POS06058	1283033.631	1083079.383	-0.24442	0.503499	0.7398	0.19103	6-Succinoaminopurine	19.952147	-	-	-	121.001	236.211469
POS06066	74851.69007	68026.63314	-0.13794	0.594577	0.7936	0.03723	Urothion	8.0144572	-	-	-	119.9935	326.376884
POS06067	1344190.914	1101118.828	-0.28777	0.060857	0.3392	0.36359	Phosfolan	-11.173509	-	-	-	119.717	256.299124
POS06068	579238.1855	289022.0801	-1.00298	0.143338	0.4679	0.39858	2-(3-Carboxy-3-(methylammonio)propyl)-L-histidine	-23.388511	-	-	-	119.6625	272.293931
POS06069	38961.10397	17861.70583	-1.12516	0.373324	0.6502	0.08914	1,2-Bis(4-hydroxy-3-methoxyphenyl)ethylene	-21.317634	-	-	-	119.6115	273.297272
POS06072	160747.2035	162139.7161	0.012444	0.967023	0.9849	0.00745	Samaderin A	-1.0856511	-	-	-	118.632	331.338818
POS06073	54132.02653	62274.63228	0.202162	0.588184	0.7904	0.03612	Betagarin	-0.2879632	-	-	-	118.607	329.323182
POS06086	952711.909	934328.7827	-0.02811	0.907573	0.9575	0.03078	Violacein	24.065445	-	-	-	117.213	344.351039
POS06088	4152646.987	3489452.492	-0.25103	0.298157	0.6115	0.45431	5-Nitrofurfural	-19.147011	-	-	-	116.594	142.086175

POS06089	161069.5185	280635.0794	0.801012	0.210128	0.5338	0.21881	Pelargonidin	19.997139	-	-	-	116.2695	272.257501
POS06094	124237.1432	134806.4573	0.117793	0.597689	0.7957	0.04495	Miraxanthin-I	-11.877601	-	-	-	115.904	359.36992
POS06095	901984.5988	768133.9431	-0.23174	0.037853	0.2906	0.29927	Deoxyviolaceinic acid	-7.8494222	-	-	-	115.8995	358.366572
POS06098	385931.3433	267527.7084	-0.52866	0.007199	0.1779	0.31208	Formothion	14.058384	-	-	-	115.399	258.278393
POS06100	222982.6745	227781.1783	0.030717	0.900722	0.9541	0.00912	6-Hydroxyprotopine	-23.811014	-	-	-	115.25	370.366482
POS06105	1740125.205	1406766.882	-0.30681	0.682322	0.8437	0.22288	Maleimide	-1.6760015	-	-	-	115.0365	98.0792139
POS06111	420900.5107	356407.6682	-0.23995	0.399615	0.6679	0.12135	Citalopram alcohol	12.542171	-	-	-	114.54	312.361282
POS06112	44912.94269	54502.50566	0.279191	0.567332	0.7784	0.05026	MK826	6.1018925	-	-	-	114.536	494.563488
POS06113	23407.83855	1503295.771	6.004993	0.342282	0.6327	0.53067	ATP	-17.598771	-	-	-	114.473	508.179351
POS06118	109581.1055	123930.9558	0.177538	0.361565	0.6438	0.06471	Clavamycin F	12.080977	-	-	-	114.065	373.385475
POS06120	725956.8221	700355.6632	-0.0518	0.562642	0.776	0.08085	N-Methyl-14-O-demethylepiporphyroxine	-23.876332	-	-	-	113.886	372.382209
POS06121	61214.70342	75396.38767	0.300617	0.537093	0.7602	0.05854	1-Oleoylglycerophosphoserine	12.466102	-	-	-	113.8605	522.594979
POS06122	6406371.407	3679376.873	-0.80005	0.208311	0.5321	1.2124	Cyazine	19.535972	-	-	-	113.859	241.704679
POS06127	2287442.052	2116362.63	-0.11215	0.738658	0.8763	0.14279	[5-(Aminomethyl)furan-3-yl]methyl phosphate	21.525377	-	-	-	113.533	208.132835
POS06130	160803.9705	152072.5374	-0.08054	0.702807	0.8544	0.03936	Silbin	-23.368821	-	-	-	113.173	426.428735
POS06134	681622.7103	551640.2018	-0.30525	0.052091	0.3217	0.27781	9-Carboxymethoxymethylguanine	-1.1635949	-	-	-	112.182	240.195198
POS06138	2378530.865	1769267.388	-0.42692	0.102587	0.4087	0.51018	3-Fluoro-cis,cis-mucote	-0.3340245	-	-	-	111.472	161.107123
POS06139	174541.4477	152767.3317	-0.19223	0.51191	0.7453	0.08065	Oolongtheanin	-9.3630353	-	-	-	110.962	733.597717
POS06152	1064613.097	1059575.013	-0.00684	0.947197	0.9764	0.01143	Anthocyanin 3'-O-beta-D-glucoside	8.4063776	-	-	-	110.108	386.397716
POS06153	270942.7136	235451.5556	-0.20256	0.228875	0.5501	0.12455	Epigallocatechin-(4beta->8)-epicatechin-3-O-gallate ester	-23.26515	-	-	-	110.104	747.613806
POS06156	123350.8152	94361.42733	-0.3865	0.126933	0.4452	0.11276	Prodelphinidin A2 3'-gallate	17.999358	-	-	-	109.766	761.628367
POS06161	624825.5412	197609.876	-1.6608	0.440843	0.6978	0.32773	Rabeprazole sodium	19.447722	-	-	-	109.057	382.439194
POS06165	84136.66505	71086.52121	-0.24316	0.126335	0.445	0.08059	Ceftizoxime	21.784171	-	-	-	108.404	384.418429

POS06168	825416.2294	1075314.742	0.381565	0.311114	0.6218	0.26034	2-Pyrone-4,6-dicarboxylate	-18.032625	-	-	-	108.072	185.107057
POS06171	5002940.879	2950009.436	-0.76206	0.112232	0.4244	0.99277	2-Amino-5-phosphopentanoic acid	5.9489749	-	-	-	107.7225	198.134649
POS06173	541043.4937	176788.3941	-1.61372	0.013983	0.2172	0.49802	3,4-Dihydroxyphthalate	5.6814845	-	-	-	107.7175	199.138002
POS06174	3509223.173	2772954.972	-0.33973	0.255033	0.5727	0.58824	Methyl 2-propenyl selenide	-12.143245	-	-	-	107.717	136.075636
POS06177	105529.8942	82785.6579	-0.3502	0.286841	0.6022	0.08188	Quercetin 3-O-beta-D-glucosyl-(1->2)-beta-D-glucosyl-(1->2)	-6.7180885	-	-	-	107.1	789.659478
POS06179	2219566.581	2139632.757	-0.05291	0.643215	0.8247	0.08754	3-Oxalomalate	-8.9750468	-	-	-	107.051	207.112427
POS06180	1889735.567	1653907.13	-0.19231	0.091839	0.393	0.34736	Citrate	13.123615	-	-	-	107.037	193.133298
POS06182	377839.2169	287921.3837	-0.3921	0.008748	0.1905	0.25756	{6-[(2-{3-[(6-carboxy-3,4,5-trihydroxyoxan-2-yl)oxy]-4-hydroxyphenyl}	-6.6591685	-	-	-	107.033	788.657031
POS06185	37323817.21	29282300.82	-0.35007	0.226824	0.5477	1.86095	Trimethylselenonium	-0.4403269	-	-	-	106.687	125.070822
POS06189	455127.3748	474332.2126	0.059627	0.715014	0.8614	0.0525	N-Pyruvoyl-5-methoxy-3-hydroxyanthranilate	-18.254983	-	-	-	106.015	254.210754
POS06191	611152.9363	306254.641	-0.9968	0.275576	0.5922	0.36993	Sevoflurane	17.035642	-	-	-	105.84	201.065485
POS06193	21629122.28	19586639.44	-0.14311	0.794147	0.9053	0.55818	Calcium hydroxide	-4.2637086	-	-	-	105.674	75.0996607
POS06194	328213.8654	212588.5851	-0.62657	0.015394	0.2226	0.27699	Delphinidin 3-lathyroside 5-(6-acetylglucoside)	-13.570297	-	-	-	105.6675	802.672498
POS06196	284755.1185	264810.3741	-0.10476	0.676979	0.8402	0.04164	2-(Formamido)-N1-(5'-phosphoribosyl)acetamidine	3.9987847	-	-	-	105.194	314.210229
POS06197	2810181.708	2331888.767	-0.26916	0.814631	0.9158	0.20675	Oxolinic acid	-1.7165804	-	-	-	105.18	262.236928
POS06203	1200418.692	927690.0594	-0.37182	0.030392	0.2685	0.40676	2-hydroxy-3-[4-(sulfooxy)phenyl]propanoic acid	12.059595	-	-	-	104.6725	263.240439
POS06204	295685.1114	291745.9087	-0.01935	0.906484	0.9569	0.00524	4,8-dihydroxy-2H-furo[2,3-h]chromen-2-one	11.600057	-	-	-	104.662	219.173807
POS06207	7481271.204	5856588.248	-0.35322	0.35071	0.6364	0.68289	2,3,6-Trihydroxypyridine	11.485509	-	-	-	104.641	128.106836
POS06210	1048603.053	782289.2704	-0.42269	0.086519	0.3863	0.37795	Oxalosuccite	14.063589	-	-	-	103.982	191.11755
POS06214	781132.8576	709245.4855	-0.13928	0.225208	0.5468	0.15778	2-hydroxy-3-[4-hydroxy-3-(sulfooxy)phenyl]propanoic acid	-23.253353	-	-	-	103.6335	279.230807
POS06216	4680107.671	4411045.896	-0.08542	0.734269	0.874	0.12942	N-Dimethyl-2-aminoethylphosphote	-9.9326465	-	-	-	102.2974	154.122456
POS06220	762362.8987	538228.4391	-0.50226	0.430244	0.6889	0.26696	Meconic acid	9.5806122	-	-	-	102.2645	201.111694
POS06224	1598733.052	1191128.233	-0.4246	0.446423	0.7014	0.33935	GMP	10.163657	-	-	-	102.236	364.231568

POS06227	2354745.568	1541474.985	-0.61126	0.272765	0.5901	0.56847	3-Deoxy-D-manno-octulosote 8-phosphate	-16.611685	-	-	-	102.046	319.173891
POS06228	696585.6507	538833.5802	-0.37046	0.545165	0.7658	0.19369	Lormetazepam	24.281987	-	-	-	101.978	336.200416
POS06233	1088905.836	10463.29193	-6.7014	0.343283	0.6329	0.57126	4,4-Bis[4-(acetyloxy)phenyl]3-hexanone	-9.0166804	-	-	-	100.963	369.426955
POS06240	92025.31522	18996.2536	-2.27632	0.238856	0.5588	0.18308	{[(2E)-2-methyl-3-phenylprop-2-en-1-yl]oxy}sulfonic acid	18.020203	-	-	-	99.8853	229.27139
POS06248	75805.78579	193399.4996	1.351204	0.537903	0.7608	0.10706	Oxidized Renilla luciferin	-13.944558	-	-	-	98.6859	396.454862
POS06253	2707764.198	2311223.442	-0.22845	0.061248	0.3403	0.46282	S-(1,2-Dichlorovinyl)-L-cysteine	17.846396	-	-	-	97.569	217.096733
POS06259	243048.474	259895.6386	0.096688	0.766729	0.8917	0.03883	CGH 2466	2.6263646	-	-	-	95.4598	323.220523
POS06260	5728081.464	5096894.573	-0.16843	0.555331	0.7717	0.26033	Maleimide	-1.6670766	-	-	-	95.4471	98.0792148
POS06263	319351.4368	244909.8528	-0.38289	0.209067	0.5326	0.1821	Olsalazine	18.405067	-	-	-	95.0929	303.251839
POS06271	407732.9394	414325.2742	0.023139	0.952825	0.9795	0.00116	2-Protocatechoylphloroglucinolcarboxylate	-19.595982	-	-	-	94.7352	307.225676
POS06277	302184.1917	300207.555	-0.00947	0.984637	0.9923	0.01061	dTDP-3-amino-3,4,6-trideoxy-D-glucose	-12.815605	-	-	-	94.0749	532.345867
POS06279	1749167.288	1491305.785	-0.23009	0.495207	0.7341	0.24269	Phosfolan	-11.160734	-	-	-	93.88705	256.299127
POS06280	457700.6753	248012.1901	-0.88399	0.06166	0.3409	0.34651	Se-Methylselenomethionine	10.453753	-	-	-	93.7181	212.150384
POS06282	483938.2842	448110.7401	-0.11097	0.705646	0.8562	0.0652	Riluzole	-2.3352381	-	-	-	93.3764	235.20503
POS06283	94859.94727	106985.2906	0.173541	0.709899	0.8584	0.04584	cis-(Homo)3-aconitate	-3.190615	-	-	-	93.2197	217.194587
POS06289	164080.4499	184435.2353	0.168711	0.717922	0.8638	0.07263	D-Erythroascorbic acid 1'-a-D-glucoside	-14.89795	-	-	-	92.702	309.241384
POS06290	298244.7456	182991.022	-0.70472	0.701757	0.8538	0.11113	cis-3,4-Leucopelargonidin	-13.895954	-	-	-	92.5352	291.271343
POS06291	201529.413	171049.5341	-0.23658	0.350894	0.6364	0.10298	Xenognosin A	-4.5519607	-	-	-	92.4923	257.30251
POS06299	464971.1564	405687.0114	-0.19677	0.622925	0.8124	0.09931	3,4,5-trihydroxy-6-{[3-hydroxy-2-(hydroxymethyl)-2	-17.176741	-	-	-	91.51175	311.256947
POS06307	267293.8735	139376.6231	-0.93944	0.32396	0.6305	0.22759	Formothion	14.072163	-	-	-	90.3141	258.278397
POS06308	321157.8254	288128.0672	-0.15657	0.81606	0.9163	0.04109	dTDP-4-oxo-2,3,6-trideoxy-D-glucose	-9.729636	-	-	-	90.29425	515.317173
POS06311	796484.6575	865640.134	0.120121	0.822513	0.9184	0.08624	Vicianose	-16.725099	-	-	-	89.96755	313.272554
POS06312	99593.38124	165998.2551	0.737046	0.334884	0.6326	0.15567	Thelephoric acid	24.671723	-	-	-	89.9574	353.267267

POS06316	137300.4655	106540.3104	-0.36594	0.144546	0.4686	0.13322	Ditalimfos	-4.0618567	-	-	-	89.2843	300.288661
POS06317	78773.4887	135335.6365	0.78076	0.483762	0.7265	0.12751	N'-Hydroxyneosaxitoxin	-18.851104	-	-	-	89.03	332.286332
POS06319	320810.5604	267434.6722	-0.26254	0.260821	0.5788	0.11742	5'-Methylthioadenosine	17.023277	-	-	-	88.94095	298.345838
POS06329	248694.7653	265846.4131	0.096217	0.735434	0.8745	0.04264	Prusin	-4.5944579	-	-	-	87.20955	296.29382
POS06330	223063.395	200996.1633	-0.15029	0.594911	0.7937	0.06883	Melicopine	-24.973975	-	-	-	86.88685	314.304152
POS06332	563825.6245	506786.9747	-0.15387	0.688203	0.8461	0.12052	N-Methyl-N'-nitro-N-nitrosoguanidine	-22.002949	-	-	-	86.5516	148.09684
POS06337	344040.036	314102.016	-0.13134	0.147944	0.4735	0.10908	Psicofuranine	-4.9155953	-	-	-	85.5163	298.273115
POS06338	469946.1127	374658.2149	-0.32692	0.588127	0.7904	0.1372	GMP	10.392407	-	-	-	85.4694	364.231651
POS06341	145576.098	126864.3258	-0.19849	0.806977	0.9118	0.06344	C.I. Pigment Blue 63	-2.9795561	-	-	-	84.5086	450.376938
POS06345	246445.9498	151991.285	-0.69728	0.243919	0.5622	0.183	(Z)-But-1-ene-1,2,4-tricarboxylate	-0.9327733	-	-	-	83.98145	189.141901
POS06347	127486.0824	118549.3301	-0.10485	0.774842	0.8961	0.0318	Dephospho-CoA	10.45039	-	-	-	83.6368	688.568662
POS06348	695546.7935	588521.8705	-0.24105	0.590918	0.7918	0.14339	3-Deoxy-D-manno-octulosote 8-phosphate	-16.327835	-	-	-	83.50745	319.173982
POS06357	136856.8282	111006.1849	-0.30203	0.138668	0.4616	0.1104	Myxochelin C	-15.82188	-	-	-	82.18605	540.53264
POS06359	244556.9024	222407.793	-0.13696	0.31721	0.6257	0.07437	Bluensidine 6-phosphate	20.206594	-	-	-	81.7103	345.229932
POS06360	291970.735	249697.5549	-0.22564	0.610441	0.8056	0.07647	2,4-Dioxotetrahydropyrimidine D-ribonucleotide	-14.788501	-	-	-	81.6851	327.199653
POS06362	107298.2624	143004.1949	0.414431	0.622358	0.8124	0.09847	Aurothioglucose	4.7820493	-	-	-	81.58175	393.189252
POS06365	631298.7676	340389.6978	-0.89114	0.138521	0.4615	0.40408	4-Hydroxylamino-2,6-dinitrotoluene	-2.1111323	-	-	-	81.4138	214.154427
POS06367	270322.8591	260784.7964	-0.05182	0.854304	0.9337	0.01049	Longifolonine	-10.58719	-	-	-	81.3774	298.309429
POS06370	149476.7943	119657.8474	-0.32101	0.308862	0.62	0.09418	Lycoricidine	-2.4393153	-	-	-	80.9943	292.262666
POS06373	338996.8192	261579.4252	-0.37402	0.025296	0.2562	0.23258	Delphinidin 3-O-(6 -O-malonyl)-beta-glucoside-3'-O-beta-glucoside	8.8644991	-	-	-	80.5171	714.584602
POS06378	30480925.43	24848796.59	-0.29473	0.274436	0.5919	1.3907	Trimethylselenonium	-0.2840471	-	-	-	79.59395	125.070841
POS06381	858539.3977	461725.792	-0.89485	0.002543	0.1099	0.59689	5,6-Dihydro-5-fluorouracil	5.3109024	-	-	-	79.4121	133.101078
POS06386	237588.9093	185356.7117	-0.35816	0.141382	0.4651	0.16518	Isoneotheaflavin 3-O-gallate	-2.8121647	-	-	-	78.95635	717.603161

POS06392	41898081.34	25178547.01	-0.73469	0.009051	0.1917	3.56369	Calcium hydroxide	-4.2452026	-	-	-	78.5446	75.0996621
POS06405	293180.6355	207899.8728	-0.4959	0.011724	0.2033	0.25589	ent-Epicatechin-(4alpha->8)-ent-epicatechin 3'-gallate	-17.926872	-	-	-	77.5076	731.618679
POS06407	217167.3374	78473.316	-1.46853	0.002943	0.117	0.34346	Capensinidin	-1.2863839	-	-	-	77.325	346.330232
POS06408	304489.0978	151632.5038	-1.00581	0.389437	0.66	0.18904	5-Hydroxymethyldeoxycytidylate	2.396229	-	-	-	77.3042	338.231185
POS06412	993172.4311	1021716.344	0.040879	0.925604	0.9662	0.0144	Oxolinic acid	-1.8449547	-	-	-	77.13525	262.236895
POS06416	533541.7342	382169.9341	-0.48139	0.025028	0.2549	0.31597	Malvidin 3-(6-malonylglucoside) 5-glucoside	-20.720225	-	-	-	76.74555	742.61611
POS06419	551054.9403	244341.3604	-1.1733	0.211624	0.5346	0.3796	Alginic acid	-21.0401	-	-	-	76.6145	419.225977
POS06431	5197270.564	5396641.376	0.054308	0.771383	0.8941	0.12227	2,3,6-Trihydroxypyridine	11.550727	-	-	-	74.9895	128.106845
POS06432	144942.6856	127783.1503	-0.18178	0.775168	0.8962	0.06649	Lancerin	16.711038	-	-	-	74.9545	407.354267
POS06434	512079.9465	365930.914	-0.4848	0.003993	0.136	0.34332	Cyanidin 3-O-(6''-glucosyl-2''-xylosylgalactoside)	-21.463092	-	-	-	74.9177	744.631416
POS06444	115612.3145	96639.41809	-0.25861	0.338972	0.6326	0.06827	Vicine	6.8243216	-	-	-	74.2648	305.265953
POS06447	1291016.085	1639371.572	0.344636	0.742995	0.8788	0.05772	gamma-L-Glutamyl-D-alanine	-0.6908464	-	-	-	74.233	219.214326
POS06450	1137253.537	923451.0177	-0.30045	0.478722	0.7225	0.20769	2-Amino-1,2-bis(p-chlorophenyl)ethanol	5.7961227	-	-	-	74.2248	283.174012
POS06455	313046.3759	238679.575	-0.3913	0.112708	0.4252	0.22027	N2-Acetyl-L-aminoadipyl-delta-phosphate	-9.1304843	-	-	-	73.883	284.177091
POS06459	8924679.974	15831740.47	0.826948	0.467249	0.7158	1.0282	Alanyl-Glutamic acid	20.851211	-	-	-	73.4378	218.211005
POS06472	356840.4763	265678.4315	-0.4256	0.276673	0.5932	0.16572	Fluo-3	-1.4639172	-	-	-	72.15115	770.54115
POS06480	5400986.331	3960212.379	-0.44765	0.082336	0.3788	0.89423	N-Dimethyl-2-aminoethylphosphote	-9.8216268	-	-	-	71.1527	154.122473
POS06491	707811.3804	667012.448	-0.08565	0.820174	0.9178	0.03689	3-Oxopropionyl-CoA	-24.193856	-	-	-	70.1134	838.567912
POS06492	450828.2062	236127.2118	-0.93301	0.175402	0.5016	0.29198	L-Selenomethionine	2.525737	-	-	-	69.60995	197.114072
POS06495	290606.8625	192251.8823	-0.59607	0.03769	0.2904	0.25173	(6-carboxy-3,4,5-trihydroxyoxan-2-yl)({4-[11-hydroxy-7-(4-hydroxy	-16.181447	-	-	-	69.0795	786.677563
POS06502	4388816.253	2816596.647	-0.63988	0.253381	0.572	0.75088	D-erythro-1-(Imidazol-4-yl)glycerol 3-phosphate	24.666644	-	-	-	68.05535	239.148251
POS06506	383836.4547	228861.2021	-0.74602	0.135827	0.458	0.2507	4,4'-Diaminostilbene dihydrochloride	8.8028141	-	-	-	67.8469	284.20597
POS06511	34361.82015	39279.76273	0.19298	0.657378	0.8316	0.02761	{2-[3,5-dihydroxy-4-(sulfooxy)phenyl]-3-hydroxy-5-oxo-5H	-20.891629	-	-	-	67.3098	384.299269

POS06512	21620899.5	17158422.39	-0.33351	0.35521	0.6391	1.2058	Cyanidin	3.2797008	-	-	-	67.19735	288.252419
POS06514	258824.1825	174931.4491	-0.56518	0.116666	0.4319	0.23074	Letrozole	-1.6267739	-	-	-	67.17105	286.309512
POS06516	3750777.632	3983452.547	0.08683	0.595948	0.7946	0.15082	Methyl 2-propenyl selenide	-12.193912	-	-	-	67.01395	136.07563
POS06520	20932.95969	3800.637153	-2.46146	0.386884	0.6585	0.07961	{[(2E)-2-methyl-3-phenylprop-2-en-1-yl]oxy}sulfonic acid	18.143556	-	-	-	66.50295	229.271418
POS06521	425067.857	193729.11	-1.13365	0.112447	0.4247	0.36245	N-Acetoxy-4-aminobiphenyl	9.9398484	-	-	-	66.4656	228.268036
POS06527	118366.8764	173986.5518	0.55571	0.317286	0.6257	0.15317	IAA-94	18.503026	-	-	-	66.1155	358.242286
POS06529	683191.8267	870790.8712	0.350036	0.436393	0.6941	0.20001	Bis(4-nitrophenyl)phosphate	-14.034524	-	-	-	65.648	341.184702
POS06532	6293485.741	6715083.934	0.093546	0.914451	0.9601	0.04341	Debromohymenialdisine	-9.9120936	-	-	-	65.30435	246.242146
POS06541	1945625.374	1794072.914	-0.117	0.85808	0.9353	0.09365	Quinolite	-19.316176	-	-	-	64.257	168.122949
POS06546	4391394.393	2091108.988	-1.07041	0.006612	0.1696	1.32603	2,4-Dinitroaniline	22.383967	-	-	-	63.9161	184.132976
POS06547	20157748.05	17044374.2	-0.24204	0.081139	0.3777	1.29187	Trans-urocanate	10.153952	-	-	-	63.9161	138.127669
POS06548	1717844.568	1267248.587	-0.4389	0.014016	0.2172	0.57143	sn-Glycerol 3-phosphate	-16.11988	-	-	-	63.6947	173.078203
POS06550	275773.7109	221411.8961	-0.31675	0.076895	0.3704	0.17206	PMEG	9.3325538	-	-	-	63.2525	290.195075
POS06554	20111.48484	56855.32457	1.499276	0.006051	0.1639	0.16951	Prunin 4",6"-di-O-gallate	19.662333	-	-	-	63.07365	739.623699
POS06555	116507.9918	82825.0877	-0.49229	0.031729	0.2743	0.15563	Xenognosin A	-4.0995117	-	-	-	63.0262	257.302626
POS06557	1501499.608	1129270.477	-0.41101	0.072855	0.3626	0.50897	Antirrhinoside	-22.313856	-	-	-	62.89765	363.328392
POS06558	128705.6694	105552.8808	-0.28611	0.449919	0.704	0.09248	7,8-Didemethyl-8-hydroxy-5-deazariboflavin	5.0662927	-	-	-	62.8968	364.331217
POS06559	484486.5281	456544.8645	-0.0857	0.521403	0.7515	0.09318	Violacein	24.09244	-	-	-	62.8902	344.351048
POS06563	1231874.388	1328728.956	0.109192	0.762141	0.889	0.07862	Dehydroascorbide(1-)	22.910757	-	-	-	62.8547	174.112242
POS06573	30939.29128	39257.26358	0.343519	0.7585	0.8874	0.02898	Proacacipetalin	-20.9961	-	-	-	61.9271	260.257633
POS06574	6141981.415	4613051.801	-0.41298	0.091781	0.393	1.00273	fluvoxamino acid	11.593407	-	-	-	61.8675	319.302567
POS06576	35215115.99	26362235.8	-0.41772	0.074622	0.3661	2.46436	Lettowianthine	-9.1013355	-	-	-	61.85835	318.299289
POS06584	156339.384	140123.0719	-0.15799	0.623194	0.8125	0.05155	Frutinone A	-17.951793	-	-	-	61.2264	265.234833

POS06585	214183.2718	226278.7874	0.079256	0.847043	0.931	0.02106	4-Nitrophenyl sulfate	-9.7307418	-	-	-	61.1931	220.177144
POS06586	1491890.667	1427447.815	-0.0637	0.81833	0.9171	0.10989	4,8-dihydroxy-2H-furo[2,3-h]chromen-2-one	11.862563	-	-	-	61.18855	219.173865
POS06590	45852.52986	49134.49771	0.099735	0.71007	0.8585	0.0235	Anthocyanin 3'-O-beta-D-glucoside	8.3695606	-	-	-	60.8478	386.397702
POS06591	183004.3089	203111.0772	0.150391	0.418008	0.6797	0.08799	3',5'-Cyclic dAMP	8.6412729	-	-	-	60.84775	314.216483
POS06594	221194.6006	189029.0253	-0.22671	0.076485	0.3703	0.13577	Gonyautoxin 1	-0.2699225	-	-	-	60.8127	412.355666
POS06595	141580.336	160890.4743	0.184458	0.231859	0.553	0.0973	N-Methyl-14-O-demethylepiporphyroxine	-23.931916	-	-	-	60.5351	372.382189
POS06596	126189.8861	151094.9914	0.25986	0.624272	0.8129	0.06389	N-Hydroxy-MeIQx	9.5556577	-	-	-	60.4927	230.247367
POS06598	36470391.78	20971496.75	-0.7983	0.477703	0.7223	1.91887	Nitrogen mustard	-9.0093093	-	-	-	60.16365	157.059371
POS06599	875681.0643	750720.9799	-0.22213	0.332296	0.6326	0.22054	L-Tyrosine methyl ester 4-sulfate	-21.092611	-	-	-	60.1336	276.27977
POS06600	11502598.32	9832376.366	-0.22635	0.326758	0.6319	0.78836	Phloretin	2.5461462	-	-	-	60.11105	275.276675
POS06602	262077.4268	202001.7124	-0.37563	0.407687	0.673	0.13554	3,5,7-trihydroxy-2-(4-hydroxy-3,5-dimethoxyphenyl)-5H	22.430248	-	-	-	59.9214	332.314708
POS06604	2076772.23	1655858.121	-0.32676	0.039239	0.2958	0.52444	Pyruvate	16.054258	-	-	-	59.8398	89.0707904
POS06605	16660.79356	11266.74154	-0.56439	0.511228	0.7452	0.03702	Sulfadimethoxine sodium	-0.2085762	-	-	-	59.8291	333.318007
POS06612	5378125.281	4735147.817	-0.18369	0.877624	0.9458	0.30339	Dihydroxyaluminium	-5.3624442	-	-	-	59.65955	138.077342
POS06614	3976574.859	3722950.712	-0.09508	0.599688	0.7973	0.18732	Cyazine	19.856982	-	-	-	59.4768	241.704756
POS06616	46317.76448	55483.7638	0.2605	0.278348	0.594	0.06158	GDP-4-acetamido-4,6-dideoxy-alpha-D-mannose	-17.722138	-	-	-	59.4728	631.389705
POS06621	97525.18498	73440.70596	-0.40919	0.261979	0.5794	0.10886	Aucubin	-9.8378528	-	-	-	59.13825	347.333669
POS06622	693882.3698	511125.5323	-0.44101	0.063476	0.3435	0.36397	Capensinidin	-0.9953097	-	-	-	59.11385	346.330333
POS06624	67599.57333	62163.96157	-0.12094	0.487982	0.7295	0.03752	PG(a-13:0/18:2(9Z,11Z))	7.1679795	-	-	-	59.0847	705.935329
POS06638	488194.1515	325933.7506	-0.58288	0.30581	0.6178	0.25097	L-Asp(4-L-Arg)	-0.657288	-	-	-	58.65195	290.295486
POS06640	772658.5746	1021411.021	0.402661	0.201951	0.5264	0.35298	1,7-diphospho-1-epi-valienol	20.915194	-	-	-	58.5072	337.141307
POS06657	216863.2371	212795.9053	-0.02732	0.877687	0.9458	0.01293	Se-Adenosylselenomethionine	-5.6496318	-	-	-	58.1939	447.345055
POS06659	31425.9901	43628.03735	0.473297	0.217788	0.5389	0.07403	dTDP-L-epivancosamine	-13.095258	-	-	-	58.1401	546.372135

POS06663	6845.237026	15223.15794	1.153095	0.044069	0.3081	0.07272	Aminobacteriohopanetriol	-24.411406	-	-	-	58.1298	546.873551
POS06672	50830262.79	42024775.25	-0.27445	0.521762	0.7515	1.80104	2,5-Furandicarboxylate	-20.505301	-	-	-	57.9512	157.097076
POS06673	1989834.793	1733372.335	-0.19907	0.217264	0.5384	0.31155	Tetrahydroxypteridine	2.3881233	-	-	-	57.9343	197.128145
POS06674	182779.0276	75308.45492	-1.27922	0.3301	0.6326	0.20185	UDP-L-Ara4N	11.449826	-	-	-	57.8775	536.304406
POS06682	36073.95985	22100.53677	-0.70688	0.237367	0.5572	0.08073	Diacetylspermine	9.9987408	-	-	-	57.8251	360.345869
POS06684	1182803.609	1338861.424	0.178796	0.565865	0.7776	0.18262	Aminomalote	-22.462666	-	-	-	57.8141	120.080702
POS06687	107342.3437	164220.2567	0.613413	0.328197	0.6322	0.14969	Carboplatin	-9.1479963	-	-	-	57.78815	372.25788
POS06688	2147838.444	1593362.554	-0.43081	0.079002	0.3738	0.62495	Flutriafol	21.243106	-	-	-	57.7545	302.304377
POS06689	2856493.423	1671060.701	-0.77348	0.140584	0.464	0.70941	2-Aminoethyl diphenylborite	24.92323	-	-	-	57.6583	226.106787
POS06690	295714.5419	176403.129	-0.74533	0.03277	0.2781	0.2983	Violaceinic acid	-19.510721	-	-	-	57.6391	374.361492
POS06691	367129.5779	260200.2779	-0.49667	0.090615	0.39	0.27131	{2-methoxy-4-[(5-oxooxolan-2-yl)methyl]phenyl}oxidane	1.5980599	-	-	-	57.6368	303.30776
POS06692	252019.7102	238106.7949	-0.08193	0.698471	0.8515	0.02795	Chlorobenside	-23.616337	-	-	-	57.61765	270.190419
POS06702	2166004.668	1927214.693	-0.16852	0.262714	0.58	0.26514	3-(Imidazol-4-yl)-2-oxopropyl phosphate	4.2566471	-	-	-	57.4958	221.128014
POS06704	51652.95394	81288.53123	0.654201	0.271902	0.5889	0.09028	Coformycin	7.5878217	-	-	-	57.4882	285.277934
POS06705	5782094.207	4673698.126	-0.30703	0.675814	0.8399	0.42103	Stipitatote	-17.28896	-	-	-	57.4882	209.128178
POS06715	162206.5871	583592.4415	1.847129	0.060558	0.3387	0.5092	5-(3'-Carboxy-3'-oxopropenyl)-4,6-dihydroxypicolite	-20.473663	-	-	-	57.4571	254.167193
POS06718	5948376.275	5321043.731	-0.16079	0.772846	0.8949	0.18303	Isopentenyl phosphate	-10.51529	-	-	-	57.4439	167.11773
POS06720	137034.9709	126234.3649	-0.11844	0.549884	0.7693	0.06028	PA(15:0/20:4(5Z,8Z,11Z,14Z))	-6.3872752	-	-	-	57.43885	683.922915
POS06722	2858857.275	2784700.028	-0.03792	0.866552	0.9399	0.02062	3-Oxalomalate	-8.7714255	-	-	-	57.372	207.112469
POS06723	6154997.172	4739591.348	-0.377	0.094162	0.3973	0.88423	2,3,6-Trihydroxypyridine	11.65651	-	-	-	57.3255	128.106858
POS06728	594798.6756	435523.6883	-0.44965	0.557651	0.7732	0.17415	5'-Phosphoribosyl-N-formylglycimide	2.4070223	-	-	-	57.3055	315.194533
POS06735	189608.3895	178496.0614	-0.08713	0.766629	0.8917	0.03665	Methyl nigakinone	-8.1509507	-	-	-	57.13185	281.283092
POS06736	196738.1153	198023.1139	0.009392	0.973139	0.9874	0.00129	Picein	-7.6826049	-	-	-	57.13185	299.293485

POS06745	802344.4183	681160.7132	-0.23623	0.404877	0.6713	0.17614	Hydroxysanguirine	-9.4338962	-	-	-	56.92475	348.3249
POS06747	8240.22838	20974.86582	1.347905	0.113939	0.4271	0.09051	3,4-dihydroxy-5-[2,4,5-trihydroxy-3-(3,4,5-trihydroxybenzoyloxy)ben	-2.829116	-	-	-	56.8277	491.334889
POS06754	2250782.182	2128119.544	-0.08085	0.734969	0.8744	0.07933	Citrate	13.265131	-	-	-	56.7725	193.133325
POS06756	128689.2669	98201.24916	-0.39008	0.093009	0.3958	0.13042	N-[(7,8-Dihydropterin-6-yl)methyl]-4-(beta-D-ribofuranosyl)aniline 5'	-21.485419	-	-	-	56.6946	483.381312
POS06758	72730.24196	54179.3446	-0.42481	0.050821	0.3197	0.11078	Cartormin	0.6759994	-	-	-	56.58745	576.525766
POS06762	104091.4108	30594.48364	-1.76651	0.034509	0.283	0.22389	Halomon	-19.253978	-	-	-	56.4554	402.392648
POS06770	31152.7794	32679.97111	0.069046	0.926438	0.9665	0.0145	L-Threonylcarbamoyladenylate	17.623835	-	-	-	56.3875	493.350553
POS06771	118641.2758	101718.606	-0.22202	0.548641	0.7686	0.0603	Ciprofloxacin	-20.326454	-	-	-	56.3569	332.342042
POS06775	1545236.353	733895.0121	-1.07418	0.018215	0.2285	0.76341	Samaderin A	-0.7635757	-	-	-	56.2681	331.338924
POS06780	36997.8532	43120.72156	0.22094	0.752107	0.8844	0.0311	m-Trigallic acid	10.153976	-	-	-	56.18905	475.340193
POS06785	247737.9536	229114.6137	-0.11275	0.031139	0.2714	0.12042	PA(18:1(11Z)/15:0)	-16.934236	-	-	-	56.128	661.910085
POS06786	102018.3442	77440.27091	-0.39767	0.108824	0.4191	0.12157	2-{[hydroxy(6-hydroxy-7-methoxy-2H-1,3-benzodioxol-5	1.674885	-	-	-	56.128	270.216727
POS06791	12798.0554	16284.49921	0.347575	0.673628	0.8392	0.03102	HR1917	-13.865192	-	-	-	56.0964	571.426568
POS06792	301164.3895	318395.9188	0.080271	0.622131	0.8124	0.07557	DHAP(6:0)	7.9919413	-	-	-	55.9772	269.21022
POS06794	54090.84563	44887.25992	-0.26908	0.536401	0.7595	0.0551	(6-carboxy-3,4,5-trihydroxyoxan-2-yl)[4-(2H-chromen-2	-10.76308	-	-	-	55.94925	400.376978
POS06795	1369.66827	1168.107154	-0.22965	0.876031	0.9451	0.0093	2,4-Dihydroxy-7,8-dimethoxy-2H-1,4-benzoxazin-3(4H)-one 2	-24.299437	-	-	-	55.94795	404.335476
POS06796	4055262.154	2336170.931	-0.79565	0.030725	0.2698	1.07336	cis-4-Carboxymethylenebut-2-en-4-olide	8.9382564	-	-	-	55.9328	141.102129
POS06799	36562087.02	26256096.07	-0.4777	0.002323	0.1065	3.02138	Calcium hydroxide	-4.0567302	-	-	-	55.8071	75.099676
POS06800	719829.104	600199.2334	-0.26221	0.031355	0.2724	0.27165	Boc-Asn-OPhNO2	2.1300332	-	-	-	55.7798	354.335329
POS06808	486648.3983	471730.3259	-0.04492	0.924229	0.9658	0.03775	Diflunisal	-19.686645	-	-	-	55.5236	251.199951
POS06817	479054.0432	192745.8167	-1.31349	0.187436	0.5135	0.35465	Deoxy-5-methylcytidylate	17.537197	-	-	-	55.4095	322.23661
POS06820	203367.678	169636.0492	-0.26165	0.000653	0.0721	0.17553	Estramustine phosphate sodium	-4.7392547	-	-	-	55.1755	565.351302
POS06826	1743196.844	700515.956	-1.31525	0.002918	0.117	0.9257	Miraxanthin-I	-11.559809	-	-	-	55.14635	359.370034

POS06827	497770.8085	386620.934	-0.36456	0.175538	0.5017	0.23929	Tricrozarin A	16.887582	-	-	-	55.14055	295.225945
POS06828	136959.0474	105101.21	-0.38197	0.000249	0.0721	0.17519	Canthaxanthin	10.233359	-	-	-	55.13165	565.852757
POS06832	8737672.346	3991349.597	-1.13037	0.034526	0.283	1.73244	Deoxyviolaceinic acid	-7.5326497	-	-	-	55.1107	358.366685
POS06833	160269.6593	54957.40657	-1.54412	0.007096	0.1777	0.28495	Sinigrin	-20.440043	-	-	-	55.1104	360.373231
POS06834	25529.76984	33943.99151	0.410976	0.354634	0.6386	0.03887	({3-[(7-methoxy-2-oxo-2H-chromen-8-yl)methyl]-2-methyloxiran-2	-8.2524106	-	-	-	55.1083	357.354336
POS06843	255931.4443	132409.4382	-0.95075	0.090618	0.39	0.251	Se-Methylselenomethionine	10.751345	-	-	-	55.0336	212.150447
POS06845	31520.40142	12185.55928	-1.37111	0.324751	0.631	0.08744	UDP-N-acetyl-2-amino-2-deoxy-D-glucurote	-6.2778891	-	-	-	54.9411	622.340576
POS06846	170116.1944	139162.7045	-0.28975	0.301084	0.6141	0.10979	8-Oxoguanine	-9.1402564	-	-	-	54.9275	166.115367
POS06849	4868884.801	3013306.657	-0.69224	0.21711	0.5384	0.91172	Trifluoromethanesulfonic acid	-22.621355	-	-	-	54.8801	151.080882
POS06851	216176.0474	270880.2606	0.325449	0.535441	0.7591	0.06769	O-Phospho-L-serine	-21.380171	-	-	-	54.7996	186.07582
POS06852	53177.95342	20032.25936	-1.4085	0.380392	0.6542	0.10946	Formothion	14.477598	-	-	-	54.77115	258.278501
POS06853	365579.0082	352496.1816	-0.05258	0.830265	0.9227	0.02365	norsertraline	-0.3042237	-	-	-	54.7657	293.210188
POS06856	2914380.623	1851359.414	-0.6546	0.016869	0.2275	0.92005	D-Ribose 5-phosphate	12.144416	-	-	-	54.759	231.119871
POS06862	705822.9284	525797.0534	-0.4248	0.23925	0.559	0.23331	Flutamide	-13.58975	-	-	-	54.51345	277.215423
POS06863	185974.0796	345598.3327	0.893995	0.339458	0.6326	0.1779	4-Bromophenylthiourea	13.273281	-	-	-	54.47985	232.123244
POS06865	123314.2223	132347.7569	0.101995	0.64836	0.8276	0.03842	Benzoyl meso-tartaric acid	-4.6666348	-	-	-	54.4502	255.19899
POS06871	1059210.406	697066.7697	-0.60362	0.037653	0.2904	0.49129	N-Acetyldemethylphosphinothricin	-13.20267	-	-	-	54.42755	210.141415
POS06873	325421.2833	281515.6498	-0.20909	0.492401	0.7324	0.07779	Bis(2-chloro-1-methylethyl)ether	19.290734	-	-	-	54.3667	172.075477
POS06874	1857723.669	2524015.175	0.442185	0.579388	0.7851	0.36281	meso-Tartaric acid	16.466855	-	-	-	54.3142	151.096548
POS06875	1351235.26	850930.1505	-0.66717	0.230508	0.552	0.43213	Oxalosuccite	14.705192	-	-	-	54.2576	191.117672
POS06879	483361.0538	418601.6415	-0.20752	0.003635	0.129	0.23742	(3S,3'S,5R,5'R,6R)-3,6-Epoxy-5,6-dihydro-3',5,8'-trihydroxy-beta,kappa	11.617546	-	-	-	54.10325	617.884043
POS06882	121821.3454	109293.9574	-0.15655	0.292676	0.6073	0.06698	GW 4064	-9.6535188	-	-	-	54.1003	543.839636
POS06890	9455.595723	19875.09913	1.071722	0.023182	0.2482	0.08336	3,4-Dihydro-3-hydroxy-4-S-glutathionyl bromobenzene	-3.0764709	-	-	-	54.0909	481.336599

POS06894	4041715.747	3072227.802	-0.39568	0.128315	0.4471	0.74449	Methyl 2-propenyl selenide	-11.994081	-	-	-	53.8061	136.075657
POS06897	5164.3343	33809.15363	2.71076	0.041163	0.2989	0.14086	3-Hydroxy-L-tyrosyl-AMP	-0.1797351	-	-	-	53.7541	527.401082
POS06908	238063.3547	200398.5637	-0.24847	0.473924	0.7203	0.08104	Sevoflurane	17.436271	-	-	-	53.12635	201.065565
POS06917	536998.6562	435354.4826	-0.30273	0.001412	0.0864	0.29757	6-Hydroxydesacetyluvaricin (incorr.)	1.9816355	-	-	-	52.9245	595.871055
POS06921	287869.2543	237249.8088	-0.27901	0.265216	0.5818	0.12894	2-Hydroxy-6-oxono-2,4-diene-1,9-dioate	-20.6847	-	-	-	52.7688	215.174947
POS06924	11203.01627	32361.36518	1.530385	0.113956	0.4271	0.11304	N,N'-Diacetylchitobiose 6'-phosphate	0.1748273	-	-	-	52.7464	505.387565
POS06934	1144025.441	987338.2215	-0.2125	0.533949	0.7588	0.15566	Calcium oxalate	-16.791936	-	-	-	52.4568	129.102126
POS06935	1890329.895	1294103.634	-0.54668	0.096896	0.4028	0.64649	Calcium L-aspartate	-15.800839	-	-	-	52.4465	172.169372
POS06938	139384.9714	106226.7408	-0.39193	0.097661	0.4036	0.1471	Pteroyltriglutamic acid	12.985998	-	-	-	52.4107	700.641762
POS06940	47493.28556	44861.17531	-0.08226	0.860125	0.9365	0.01086	11,15-dihydroxy-17,18-dimethoxy-6,8,20-trioxapentacyclo[10.8.0.0i	13.870692	-	-	-	52.4027	371.325413
POS06941	170852.946	311704.0482	0.867422	0.408632	0.6736	0.23285	3-(2-Carboxyethenyl)-cis,cis-mucote	18.635855	-	-	-	52.4014	213.16743
POS06942	211564.784	164492.1698	-0.36308	0.300428	0.6132	0.1306	4,4'-Diaminostilbene dihydrochloride	9.0343398	-	-	-	52.3991	284.206035
POS06948	209908.9289	179008.4215	-0.22974	0.426216	0.6861	0.07324	6-Deoxy-6-sulfo-D-glucono-1,5-lactone	-20.501921	-	-	-	52.0661	243.206111
POS06954	1582074.534	2029533.887	0.359331	0.562004	0.776	0.33917	Cytosine	24.133684	-	-	-	52.0631	112.111958
POS06958	352768.4977	228459.3327	-0.62678	0.110004	0.4215	0.23204	Anthragallol	15.418057	-	-	-	52.0477	257.221527
POS06963	91111.73411	77295.36461	-0.23725	0.041366	0.2993	0.09673	Glycerophosphocholine	-13.212857	-	-	-	51.7196	258.225178
POS06969	487759.5493	407989.4036	-0.25764	0.016294	0.2246	0.23244	Pancuronium	-19.529597	-	-	-	51.6527	573.857989
POS06973	293288.914	166870.7241	-0.81359	0.095266	0.3994	0.26021	4-hydroxy-3-nitrophenylacetate	20.698331	-	-	-	51.3858	197.150336
POS06977	159548.9279	143991.4489	-0.14802	0.57158	0.7805	0.06172	GDP-3,6-dideoxy-D-galactose	16.87469	-	-	-	51.38355	574.359252
POS06978	68046.01729	60030.23679	-0.18082	0.440197	0.6972	0.04736	Malonyl-CoA	-9.6497455	-	-	-	51.381	854.57934
POS06979	3918320.79	2834247.453	-0.46727	0.104537	0.4116	0.69271	N-Dimethyl-2-aminoethylphosphote	-9.561488	-	-	-	51.3779	154.122513
POS06984	1636920.027	955636.1773	-0.77645	0.467561	0.7159	0.31202	Quisqualic acid	0.7546214	-	-	-	51.1345	190.133619
POS06987	131492.8181	119914.2133	-0.13298	0.65134	0.829	0.05302	Pseudohypericin	8.8480233	-	-	-	51.0778	521.454481

POS06991	1092045.101	847747.478	-0.36533	0.043572	0.3066	0.39831	Phosphotyrosine	14.440094	-	-	-	51.0464	262.179448
POS06992	53647.55025	44268.3199	-0.27724	0.259631	0.5782	0.05589	Selenodiglutathione	2.3763647	-	-	-	51.0464	692.60002
POS06994	56044.61225	41207.6158	-0.44366	0.28667	0.6021	0.06985	Cyhexatin	-16.960904	-	-	-	51.0445	386.172744
POS06995	515539.2939	575763.256	0.159393	0.634988	0.8188	0.09918	Dihydrostreptomycin 3'alpha,6-bisphosphate	3.3068407	-	-	-	51.0402	744.559435
POS06998	1525325.743	1010473.368	-0.59409	0.014034	0.2172	0.61229	Oxaloacetate	-22.10027	-	-	-	51.0245	133.075958
POS07000	68635.13302	50292.34232	-0.44861	0.059814	0.3369	0.11274	Pumiloside	12.49	-	-	-	50.92575	513.522178
POS07004	868204.1975	854591.4917	-0.0228	0.899286	0.9537	0.05081	Coenzyme F420	7.9775833	-	-	-	50.8643	774.606048
POS07006	140218.1434	87328.20582	-0.68315	0.214597	0.5366	0.13828	Selenohomocystine	-19.804714	-	-	-	50.7584	363.143704
POS07010	1400927.21	1411804.334	0.011158	0.967787	0.9854	0.00966	3,4,5-trihydroxy-6-{[3-hydroxy-2-(hydroxymethyl)-2	-17.016907	-	-	-	50.712	311.256997
POS07012	122556.182	111143.9425	-0.14101	0.572379	0.7811	0.04602	Reduced coenzyme F420	-0.0835688	-	-	-	50.712	776.615712
POS07014	67424.29719	74145.30228	0.137087	0.644734	0.8254	0.03807	Proanthocyanidin A2	8.0551024	-	-	-	50.7041	577.51632
POS07015	1177794.899	1593894.545	0.436468	0.426739	0.6863	0.35388	Vicianose	-16.45586	-	-	-	50.7039	313.272638
POS07017	454183.6956	431632.4053	-0.07347	0.785016	0.9013	0.03392	2-Protocatechoylphloroglucinolcarboxylate	-19.205238	-	-	-	50.7033	307.225795
POS07021	307843.1025	324256.7404	0.074941	0.858304	0.9354	0.04561	Bromodiphenhydramine	-4.0059855	-	-	-	50.62445	335.256938
POS07035	151714.3665	279865.9973	0.883379	0.356852	0.6404	0.21321	Manniflavanone	1.3468258	-	-	-	50.36945	591.495972
POS07040	41500.13877	56279.4158	0.439491	0.359624	0.6425	0.07051	19-Bromoaplysiatoxin	15.822716	-	-	-	50.36315	751.528752
POS07044	38303.29824	60091.5142	0.649693	0.424174	0.6846	0.07552	Protoleucomelone	-0.5165696	-	-	-	50.3472	593.510771
POS07055	70340.22011	88607.17112	0.333074	0.63303	0.8185	0.06108	Apigenin 7-[glucuronyl-(1->2)-glucuronide] 4'-glucuronide	-10.736245	-	-	-	50.064	799.608003
POS07056	1098011.435	727304.024	-0.59426	0.026851	0.2585	0.51762	2-Amino-4-oxo-6-(1',2'-dioxoprolyl)-7,8-dihydroxypteridine	20.597567	-	-	-	50.0543	268.21108
POS07059	165031.6327	146298.8905	-0.17382	0.608418	0.8042	0.05654	Theogallinin	-12.114517	-	-	-	50.0543	801.624277
POS07064	455842.0089	358659.4663	-0.34592	0.396825	0.6661	0.17933	Delphinidin 3,5-di(6-O-malonylglucoside)	-3.9631487	-	-	-	50.026	800.621308
POS07068	42079.74208	70325.10536	0.740914	0.336751	0.6326	0.09609	3,4,5-trihydroxy-6-({6,13,14-trihydroxy-3,10-dioxo-12-[5,6,7	-13.213005	-	-	-	50.00165	791.544831
POS07070	196277.8839	204488.2066	0.05912	0.856843	0.9351	0.02805	{11-methoxy-16,18-dioxo-6,8,19-trioxapentacyclo[10.7.0.0	10.687445	-	-	-	49.95735	409.341641

POS07073	855746.9195	830758.6493	-0.04275	0.930889	0.9685	0.01053	Oxaloglutarate	7.8607284	-	-	-	49.8092	205.143081
POS07075	157565.7034	208098.6577	0.401314	0.578035	0.7845	0.09633	Luteoskyrin	9.2601287	-	-	-	49.752	575.501096
POS07078	267047.1508	187481.2627	-0.51035	0.111658	0.4241	0.19797	2-Amino-3,4-dihydroxypentanedioic acid	-23.931904	-	-	-	49.7019	180.13109
POS07080	63249.6897	66252.38275	0.066914	0.867307	0.9403	0.01637	3,4,5-trihydroxy-6-(1-oxo-1H-isochromene-3-carbonyloxy)oxane-2	24.038744	-	-	-	49.7004	367.294081
POS07082	93487.46051	171987.0298	0.879455	0.331932	0.6326	0.12638	Arbutin 6-phosphate	-21.315806	-	-	-	49.60165	353.230869
POS07086	173734.4234	187115.5874	0.107046	0.846906	0.9309	0.04545	Tetracenomycin D3	-5.4294536	-	-	-	49.34385	381.309712
POS07087	1488601.469	1260315.897	-0.24017	0.341287	0.6326	0.25452	Dihydrokaempferol	-12.508768	-	-	-	49.20125	289.255871
POS07088	11047854.46	7297549.609	-0.59828	0.036823	0.2883	1.62192	3-Deoxy-lyxo-heptulosaric acid	-14.625841	-	-	-	49.1912	223.153527
POS07089	9729886.497	7832622.839	-0.31293	0.77725	0.8978	0.41318	Trimethylselenonium	-0.5469698	-	-	-	49.1668	125.070809
POS07094	36208.09622	27926.89716	-0.37466	0.201885	0.5264	0.0566	TG(22:0/22:5(7Z,10Z,13Z,16Z,19Z)/22:5(4Z,7Z,10Z,13Z,16Z))	-20.877731	-	-	-	48.9846	1040.62547
POS07099	8767410.124	6796137.038	-0.36744	0.067908	0.3531	1.07799	Cyanidin	3.8674613	-	-	-	48.6694	288.252588
POS07103	811241.2289	1033950.005	0.349964	0.610389	0.8056	0.23557	Sulfur mustard	13.76025	-	-	-	48.3092	160.086766
POS07130	2124860.069	1372655.78	-0.6304	0.352383	0.6365	0.44999	Glyphosate	3.7527646	-	-	-	46.4669	170.081011
POS07131	790619.6867	650880.5006	-0.28059	0.153805	0.4815	0.25426	4,8-dihydroxy-2H-furo[2,3-h]chromen-2-one	11.994423	-	-	-	46.4499	219.173893
POS07137	3708350.196	2770433.845	-0.42067	0.458084	0.7106	0.59481	5-Nitrofurfural	-18.896064	-	-	-	45.33685	142.086211
POS07142	570189.1791	361093.2832	-0.65907	0.128677	0.4471	0.3366	Pydanon	15.515156	-	-	-	44.6951	189.148296
POS07143	1882453.678	848950.9073	-1.14886	0.009908	0.194	0.91038	2-(6-carboxy-3,4,5-trihydroxyoxan-2-yl)propanedioic acid	9.8363645	-	-	-	44.68085	281.195033
POS07150	9011924.809	7811983.773	-0.20615	0.383831	0.6571	0.71959	Lettowianthine	-9.1242072	-	-	-	44.2699	318.299282
POS07151	2822358.318	2433101.84	-0.2141	0.375438	0.6505	0.37593	Phloretin	2.6808558	-	-	-	44.2402	275.276712
POS07154	3365215.91	759992.3726	-2.14664	0.289958	0.6044	0.91254	Dihydroxyaluminium	-5.2610871	-	-	-	44.12395	138.077355
POS07155	563212.3285	486479.8528	-0.2113	0.496048	0.7344	0.1301	Hydroxysanguirine	-9.4619776	-	-	-	44.1162	348.32489
POS07158	1691030.929	1347054.23	-0.3281	0.10734	0.4167	0.45602	fluvoxamino acid	11.941825	-	-	-	43.98305	319.302678
POS07167	1282120.207	1046401.654	-0.29309	0.298279	0.6115	0.31735	3-(Phosphoacetylamido)-L-alanine	8.627422	-	-	-	43.0188	243.133166

POS07174	805920.3116	592175.164	-0.44461	0.289952	0.6044	0.27462	4-Nitrophenol	13.195009	-	-	-	42.6505	140.117912
POS07185	263045.641	166389.0074	-0.66075	0.035306	0.2845	0.26843	Capensinidin	-0.8122775	-	-	-	42.0664	346.330396
POS07196	727793.7388	571045.0643	-0.34993	0.219224	0.5401	0.29423	Flutriafol	21.238804	-	-	-	41.7496	302.304376
POS07198	242584.8488	176348.3111	-0.46006	0.562504	0.776	0.12361	3,5,7-trihydroxy-2-(4-hydroxy-3,5-dimethoxyphenyl)-5H	22.475006	-	-	-	41.72845	332.314723
POS07203	30844.56162	36845.12778	0.256458	0.471139	0.719	0.04178	ent-Epicatechin-(4alpha->8)-ent-epicatechin 3'-gallate	-17.785622	-	-	-	41.6448	731.618782
POS07204	1120578.042	866756.2619	-0.37054	0.023569	0.2493	0.41144	Meconic acid	-1.8462165	-	-	-	41.6446	201.109407
POS07208	603801.7586	451152.3665	-0.42046	0.145716	0.4704	0.26123	Lofexidine	-1.6720798	-	-	-	41.6229	260.138843
POS07221	340686.7466	344439.1527	0.015803	0.971342	0.9872	0.02619	L-Selenocystathionine	-18.159405	-	-	-	41.46565	270.159489
POS07225	115157.2218	92051.81096	-0.32309	0.417189	0.6796	0.07392	Malvidin 3-(6-malonylglucoside) 5-glucoside	-21.239681	-	-	-	41.4024	742.615725
POS07231	971858.0104	855263.5919	-0.18438	0.331581	0.6326	0.21522	Se-Propenylselenocysteine Se-oxide	-3.592456	-	-	-	41.3189	225.122871
POS07239	541332.8784	533931.6629	-0.01986	0.916658	0.9615	0.03713	1,7-diphospho-1-epi-valienol	21.329226	-	-	-	41.1324	337.141446
POS07241	1239179.422	933175.1797	-0.40917	0.2551	0.5727	0.35421	5-Phosphoribosylamine	-17.104254	-	-	-	41.13035	230.128358
POS07245	176134.3191	295798.3594	0.747938	0.349851	0.6362	0.20251	Cyanidin 3-O-(6''-glucosyl-2''-xylosylgalactoside)	-22.432349	-	-	-	41.11075	744.630695
POS07248	988728.043	584046.9383	-0.75949	0.154473	0.4825	0.5032	Phosphocreatine	-11.58137	-	-	-	41.0575	212.117932
POS07250	467235.7565	1125584.214	1.268451	0.354272	0.6383	0.3466	Cobalt sulfate	-19.180195	-	-	-	40.9957	282.104685
POS07253	973913.5526	642193.8141	-0.60078	0.239403	0.559	0.37196	4-Nitro-6-oxohepta-2,4-dienedioate	18.977687	-	-	-	40.9807	216.128459
POS07255	2464577.481	2488953.501	0.014199	0.942025	0.9742	0.07161	cis-4-Carboxymethylenebut-2-en-4-olide	8.7085705	-	-	-	40.96935	141.102097
POS07265	1792834.565	1616223.925	-0.14962	0.483015	0.7262	0.20635	Tetrahydroxypteridine	2.2697385	-	-	-	40.9505	197.128122
POS07267	846250.3673	708423.8434	-0.25647	0.197676	0.5233	0.22253	Benoxacor	-3.8992084	-	-	-	40.8529	261.122762
POS07269	2230241.006	2355340.803	0.078736	0.790203	0.9034	0.05706	4-Oxoproline	9.5355221	-	-	-	40.8481	130.122508
POS07287	2073666.679	717702.116	-1.53073	0.005758	0.1587	1.11896	Sedoheptulose 7-phosphate	1.2115986	-	-	-	40.70895	291.169428
POS07292	884520.0325	668152.3277	-0.40472	0.117734	0.4323	0.29984	Mevinphos	16.085631	-	-	-	40.6459	225.159182
POS07293	5386596.135	4725415.118	-0.18893	0.351513	0.6364	0.40839	Trifluoromethanesulfonic acid	14.277489	-	-	-	40.64455	151.086419

POS07295	567133.7248	296925.3282	-0.93359	0.075236	0.3677	0.39079	Samaderin A	-0.7787774	-	-	-	40.64395	331.338919
POS07310	1419027.343	987821.6207	-0.52258	0.416391	0.6793	0.33689	4-Nitrophenyl phosphate	2.1993587	-	-	-	40.6259	220.096458
POS07311	724742.2954	495454.919	-0.54871	0.126435	0.445	0.31827	Barban	-3.0933138	-	-	-	40.6234	259.107178
POS07312	565998.1104	576930.2176	0.0276	0.904607	0.956	0.00922	2-Dehydro-3-deoxy-D-arabino-heptote 7-phosphate	1.7918476	-	-	-	40.6229	289.153693
POS07317	890643.4725	767252.1006	-0.21515	0.20058	0.5255	0.19385	DCI	12.048893	-	-	-	40.6225	249.159167
POS07337	117517.2596	546897.0181	2.218397	0.311222	0.6218	0.30164	2,6-Dichlorophenolindophenol sodium salt	-4.2497976	-	-	-	40.4453	291.083344
POS07338	488637.5018	303652.0531	-0.68635	0.100015	0.4061	0.2819	Picein	-7.3922743	-	-	-	40.4453	299.293572
POS07341	3436016.299	3177546.394	-0.11282	0.635209	0.8189	0.23713	Cyazine	19.967902	-	-	-	40.4204	241.704783
POS07344	86959.04128	74735.66556	-0.21854	0.511741	0.7452	0.03694	(6-carboxy-3,4,5-trihydroxyoxan-2-yl)({4-[11-hydroxy-7-(4-hydroxy [illegible]	-16.374662	-	-	-	40.4167	786.677411
POS07354	991978.5324	956236.7447	-0.05294	0.909799	0.9581	0.04341	Dichlormid	-2.6695911	-	-	-	40.3231	209.091721
POS07359	2743.229143	13888.9695	2.339993	0.327326	0.632	0.06053	({3-[(7-methoxy-2-oxo-2H-chromen-8-yl)methyl]-2-methyloxiran-2 [illegible]	-7.3627616	-	-	-	40.3119	357.354653
POS07362	480205.7774	354462.1418	-0.43802	0.162221	0.4895	0.26431	Azacozole	19.159244	-	-	-	40.3066	301.153627
POS07364	1288611.056	974746.5759	-0.40272	0.13016	0.4485	0.40521	3-Sulfopyruvate	4.5283145	-	-	-	40.30405	169.133338
POS07365	587232.7823	993989.0146	0.759297	0.35114	0.6364	0.28244	S-(4-Bromophenyl)-L-cysteine	-13.139882	-	-	-	40.3017	277.153848
POS07366	776286.687	295178.6962	-1.395	0.0066	0.1696	0.61315	Miraxanthin-I	-11.363118	-	-	-	40.3017	359.370104
POS07367	106118.3003	189224.9673	0.834429	0.097705	0.4036	0.22703	alpha-1,5-L-Arabinotetraose	-20.431817	-	-	-	40.3007	547.469911
POS07376	143287.7978	289749.2029	1.015889	0.051008	0.3203	0.31821	({3,4,5-trihydroxy-6-[3,5,7-trihydroxy-2-(2,4,5-trihydroxyphenyl)-3,4 [illegible]	15.61896	-	-	-	40.2419	549.485843
POS07378	137134.0538	241445.1419	0.816109	0.208653	0.5322	0.19309	Methylselenocysteine Se-oxide	0.028301	-	-	-	40.1964	199.086482
POS07379	2258961.733	1812017.469	-0.31806	0.106064	0.4145	0.4742	Citrate	13.715985	-	-	-	40.1928	193.133412
POS07380	2797895.366	2215153.801	-0.33694	0.225527	0.5472	0.41821	3-Oxalomalate	-8.8142464	-	-	-	40.1885	207.11246
POS07385	73737.14578	130691.571	0.825703	0.030903	0.2705	0.21546	PA(18:1(11Z)/15:0)	-17.780656	-	-	-	40.1603	661.909525
POS07386	1535454.786	680637.6071	-1.17371	0.013038	0.211	0.83699	3-(3,4-Dihydroxyphenyl)pyruvate	2.5908433	-	-	-	40.1527	197.164585
POS07387	1100567.508	1118203.825	0.022936	0.955331	0.9803	0.04991	CoA	23.893501	-	-	-	40.1445	768.559716

POS07391	1506597.976	1203437.327	-0.32413	0.141519	0.4653	0.36438	S-(1,2-Dichlorovinyl)-L-cysteine	17.82872	-	-	-	40.1261	217.096729
POS07394	880638.286	2115959.354	1.26469	0.370813	0.6495	0.45123	3-(2-Carboxyethenyl)-cis,cis-mucote	-19.885414	-	-	-	40.11	213.159258
POS07397	1792184.674	761870.1563	-1.2341	0.007189	0.1779	0.96196	1,1-Dichloro-2,2-diphenylethane	1.8299773	-	-	-	40.11	252.158836
POS07402	1895620.719	1779387.551	-0.09129	0.646375	0.8266	0.09622	Stipitatote	-17.492564	-	-	-	40.0632	209.128136
POS07407	3500788.567	1513192.868	-1.21008	0.006994	0.1764	1.23416	Deoxyviolaceinic acid	-7.6194246	-	-	-	40.0562	358.366654
POS07408	1174789.576	896333.4771	-0.39029	0.147109	0.4726	0.32674	O-Phospho-4-hydroxy-L-threonine	-19.191399	-	-	-	40.0554	216.101649
POS07409	2377056.003	1907795.255	-0.31727	0.062817	0.3421	0.50882	Oxalosuccite	14.689933	-	-	-	40.0504	191.117669
POS07420	979794.6724	1412906.572	0.528115	0.408846	0.6736	0.27826	unsym-Bis(4'-chlorophenyl)ethylene	2.9301446	-	-	-	40.0132	250.143207
POS07421	1092923.914	743745.4519	-0.55531	0.444017	0.6995	0.37451	2-epi-5-epi-Valiolone 7-phosphate	18.99314	-	-	-	40.0132	273.158946
POS07424	105418.2562	154485.5432	0.551347	0.235327	0.5559	0.14987	Proanthocyanidin A2	7.6725097	-	-	-	40.0062	577.5161
POS07428	804829.5015	691040.0146	-0.21991	0.385579	0.6583	0.19343	2-Phosphinomethylmalate	0.4876563	-	-	-	40.0006	213.10188
POS07433	565058.3619	698935.9673	0.30676	0.538582	0.761	0.12547	Tris(2-methyl-1-aziridinyl)phosphine oxide	15.303276	-	-	-	39.9908	216.24307
POS07436	122433.8475	117236.1976	-0.06258	0.865369	0.9391	0.00379	N2-Acetyl-L-aminoadipyl-delta-phosphate	17.739191	-	-	-	39.9874	284.1847
POS07438	2407438.014	1092902.811	-1.13933	0.327626	0.632	0.86468	CNQX	20.076419	-	-	-	39.9874	233.164437
POS07441	4434054.868	3280654.223	-0.43464	0.037702	0.2904	0.79149	Diethylphosphoric acid	-14.606883	-	-	-	39.9795	155.106526
POS07443	938700.4043	876096.557	-0.09957	0.659736	0.8327	0.10099	D-Glucurote	11.792498	-	-	-	39.9772	195.148966
POS07444	984912.7956	736834.6531	-0.41866	0.05348	0.3235	0.37265	Apraclonidine	-15.471317	-	-	-	39.97685	246.111984
POS07448	930867.0411	303510.512	-1.61683	0.00094	0.0736	0.71102	Orellanine	10.448495	-	-	-	39.9764	253.190212
POS07449	1115375.906	864053.2153	-0.36834	0.0555	0.3276	0.37815	Oxaloacetate	-22.50949	-	-	-	39.97515	133.075904
POS07450	2906056.727	2413480.879	-0.26795	0.104944	0.412	0.47848	Isopentenyl phosphate	-10.51934	-	-	-	39.973	167.117729
POS07453	743521.997	483088.9793	-0.62209	0.013853	0.2168	0.45768	3-Dehydroquite	-20.841012	-	-	-	39.9412	191.154014
POS07463	5333661.577	3713468.025	-0.52236	0.417206	0.6796	0.84752	Maleimide	-1.4303521	-	-	-	39.8222	98.0792378
POS07466	541270.44	536475.6217	-0.01284	0.91034	0.9581	0.00199	Se-Methyl-L-selenocysteine	24.060877	-	-	-	39.80595	183.091458

POS07474	6491751.616	6072349.138	-0.09635	0.553675	0.7708	0.2241	2,3,6-Trihydroxypyridine	11.994956	-	-	-	39.7646	128.106901
POS07475	165057.4153	145001.6473	-0.1869	0.710319	0.8585	0.04282	L-Histidine	0.6063503	-	-	-	39.7646	156.161971
POS07478	2005766.028	1355588.049	-0.56523	0.059044	0.336	0.66887	(E)-2-(2-Furyl)-3-(5-nitro-2-furyl)acrylamide	-13.172741	-	-	-	39.75735	249.195607
POS07479	52177.52487	3356732.373	6.007485	0.345853	0.6345	0.78925	Sulfentrazone	-5.1373559	-	-	-	39.7464	388.195187
POS07480	105440.8164	153485.2619	0.541667	0.128565	0.4471	0.16668	2,2',3,3',4',5,6'-Heptabromodiphenyl ether	22.831371	-	-	-	39.7461	723.503372
POS07484	54681.7831	70185.81791	0.360119	0.373934	0.6502	0.07565	Tri-N-acetylchitotriose	-24.522178	-	-	-	39.72335	628.584687
POS07485	723843.3093	461331.3058	-0.64987	0.120485	0.4373	0.37233	5-(3'-Carboxy-3'-oxopropenyl)-4,6-dihydroxypicolite	8.2276334	-	-	-	39.71725	254.17446
POS07488	917924.6361	610530.8488	-0.58831	0.006055	0.1639	0.47671	D-Glucosamite-6-phosphate	3.9481426	-	-	-	39.6949	276.158763
POS07492	703351.5531	499097.9954	-0.49492	0.323207	0.6304	0.22021	D-Glucosamine 6-phosphate	21.296533	-	-	-	39.6731	260.163796
POS07493	422615.1831	436767.5516	0.047521	0.865696	0.9394	0.04967	Diclofec	10.407906	-	-	-	39.6699	297.158959
POS07496	402818.7408	152354.4119	-1.4027	2.40E-05	0.0721	0.50531	Coumermic acid	-20.759667	-	-	-	39.6686	548.465611
POS07509	784957.4906	640313.1937	-0.29384	0.079686	0.374	0.27781	Aminopyrrolnitrin	18.763277	-	-	-	39.6603	228.101438
POS07512	1103834.624	883618.7419	-0.32103	0.29849	0.6115	0.25412	Bowdichione	9.7716598	-	-	-	39.6603	299.257191
POS07516	276119.7508	208365.1678	-0.40618	0.100268	0.4065	0.18931	(3-aminopropoxy)sulfonic acid	-17.45749	-	-	-	39.6585	156.174568
POS07522	2683585.851	2334800.968	-0.20086	0.082187	0.3786	0.51298	Methyl (2-propenylthio) selenide	5.069845	-	-	-	39.65395	168.138124
POS07535	1094582.312	1059492.22	-0.04701	0.963849	0.983	0.03891	S-Sulfo-L-cysteine	4.9867699	-	-	-	39.5633	202.22968
POS07539	1027529.492	791934.4537	-0.37573	0.544232	0.7649	0.22362	N-Formylmaleamic acid	-20.506296	-	-	-	39.545	144.101842
POS07543	5696038.477	3969983.002	-0.52083	0.051386	0.3203	1.09858	3-[3,5-dihydroxy-4-(sulfooxy)phenyl]prop-2-enoic acid	-3.6604024	-	-	-	39.5052	277.226266
POS07548	3075357.721	2869629.153	-0.09989	0.708577	0.8578	0.15927	Threote	-23.995909	-	-	-	39.5031	137.107311
POS07549	400136.5327	422398.8104	0.078113	0.808522	0.9121	0.0725	2,6-Dichloro-4'-biphenylol	-13.046292	-	-	-	39.5031	240.101457
POS07550	1280417.139	912758.0152	-0.48831	0.198935	0.5248	0.36756	2-Deoxy-D-ribose 1-phosphate	-0.2113288	-	-	-	39.5031	215.117631
POS07552	107271.5612	1346134.149	3.649483	0.343031	0.6329	0.48649	Nitisinone	7.7494615	-	-	-	39.48925	330.237928
POS07560	474675.3949	596806.1976	0.330321	0.363562	0.6446	0.23172	D-Erythroascorbic acid 1'-a-D-glucoside	-14.694979	-	-	-	39.45545	309.241447

POS07561	2945500.039	2524799.734	-0.22234	0.305278	0.6174	0.36791	Methyl 2-propenyl selenide	-12.001651	-	-	-	39.44685	136.075656
POS07569	454923.2597	403259.2894	-0.17392	0.288181	0.603	0.12864	(3S,3'S,5R,5'R,6R)-3,6-Epoxy-5,6-dihydro-3',5,8'-trihydroxy-beta,kappa [illegible]	10.989238	-	-	-	39.42575	617.883656
POS07570	384173.8467	129763.2908	-1.56588	0.000258	0.0721	0.50376	Pentamide	16.651907	-	-	-	39.4234	102.155861
POS07571	2545456.55	1982358.939	-0.36071	0.005471	0.1556	0.67309	3-Fluoro-cis,cis-mucote	-0.1372238	-	-	-	39.4234	161.107155
POS07572	451449.4488	155801.4661	-1.53486	0.005508	0.1559	0.50095	2-Amino-4-hydroxy-6-hydroxymethyl-7,8-dihydropteridine	13.627355	-	-	-	39.4234	196.188636
POS07573	6501.420779	14581841.93	11.13113	0.340125	0.6326	1.67146	Bifenox	-2.0718034	-	-	-	39.4234	343.137568
POS07575	1505842.775	724025.4423	-1.05646	0.000965	0.0736	0.87703	Potassium bicarbote	-24.278243	-	-	-	39.4221	101.119946
POS07581	34104.84799	659203.0632	4.272674	0.316603	0.6253	0.35984	4-(N-Maleimido)phenyltrimethylammonium iodide	16.124945	-	-	-	39.4208	359.187952
POS07586	641424.06	458094.4516	-0.48563	0.033911	0.2807	0.33963	Calcium peroxide	-0.3754775	-	-	-	39.3992	73.0840495
POS07587	434199.5315	337397.0591	-0.36391	0.061234	0.3403	0.22757	Theophylline	-8.733225	-	-	-	39.3992	181.169703
POS07589	519690.2238	630993.7064	0.279974	0.44176	0.6978	0.18653	Dihydrostreptomycin 3'alpha,6-bisphosphate	2.4340769	-	-	-	39.3992	744.558786
POS07590	1003633.29	855943.6131	-0.22964	0.345837	0.6345	0.21237	Coenzyme F420	7.9441069	-	-	-	39.3992	774.606022
POS07591	575137.1531	465100.8439	-0.30636	0.039515	0.296	0.26226	6-Hydroxydesacetyluvaricin (incorr.)	1.3666819	-	-	-	39.39755	595.87069
POS07593	310489.8792	204015.0144	-0.60587	0.086931	0.3863	0.27118	{[(2E)-2-methyl-3-phenylprop-2-en-1-yl]oxy}sulfonic acid	-17.205959	-	-	-	39.38825	229.263349
POS07600	4452068.275	2156668.594	-1.04567	0.000772	0.0721	1.49038	(alpha-keto-dimethyl-delta-N,N-Dimethylguanidyno[illegible]	11.032849	-	-	-	39.38825	202.235497
POS07604	228446.0134	150032.0123	-0.60658	0.0128	0.21	0.26202	L-Caline	10.789887	-	-	-	39.3773	135.142524
POS07611	3094659.015	2319303.958	-0.41609	0.057241	0.3313	0.65351	[5-(Aminomethyl)furan-3-yl]methyl phosphate	22.234449	-	-	-	39.3773	208.132982
POS07612	547577.9343	216237.389	-1.34045	0.000178	0.0721	0.5718	[(2Z)-3-oxo-2-(phenylmethylidene)propoxy]sulfonic acid	18.905059	-	-	-	39.3773	243.261856
POS07618	1438653.875	1157356.528	-0.31389	0.005215	0.1523	0.48071	L-Hexahydro-3-imino-1,2,4-oxadiazepine-3-carboxylic acid	11.37457	-	-	-	39.3745	160.152387
POS07620	1877669.682	849759.3668	-1.14382	0.001164	0.0803	0.99882	N-Hydroxy-IQ	0.2627589	-	-	-	39.3745	215.230633
POS07621	4025651.244	1881720.658	-1.09717	0.000724	0.0721	1.45096	Tetrahydrobiopterin	16.809708	-	-	-	39.3745	242.258432
POS07622	1102461.7	809907.3216	-0.4449	0.016911	0.2275	0.43723	Phosphotyrosine	14.596177	-	-	-	39.3745	262.179489
POS07623	1572725.881	751039.368	-1.06631	0.002069	0.1021	0.87552	5-Hydroxysulfamethoxazole	19.532089	-	-	-	39.3745	270.289536

POS07624	386027.9105	175254.1449	-1.13926	0.000929	0.0736	0.44961	3,5,7-trihydroxy-2-(3-methoxyphenyl)-5H-chromen-5-yl	7.4629336	-	-	-	39.3745	286.284406
POS07636	1885380.257	1653929.844	-0.18896	0.081747	0.378	0.36398	2,4-Dinitroaniline	22.961248	-	-	-	39.36285	184.133081
POS07637	357939.1539	180055.6572	-0.99127	0.001421	0.0864	0.41224	7-Methylinosine	3.3117973	-	-	-	39.3627	284.268815
POS07638	615097.2985	366659.398	-0.74637	0.002418	0.1081	0.47417	2-Amino-4-hydroxy-6-(D-erythro-1,2,3-trihydroxypropyl)-7,8	-1.0273206	-	-	-	39.36005	256.237614
POS07641	20205217.48	11160763.15	-0.85629	0.000294	0.0721	3.00792	3-Fluoro-1-(4-hydroxyphenyl)-1-propanone	-15.279641	-	-	-	39.36005	169.169707
POS07642	2358398.804	1502252.758	-0.65068	0.001737	0.0951	0.89985	6-Thiourate	3.6038671	-	-	-	39.36005	185.18384
POS07645	2169201.407	1067441.947	-1.02301	0.00095	0.0736	1.04434	Niridazole	10.871255	-	-	-	39.36005	215.211405
POS07651	59076126.79	27742215.09	-1.09049	0.000413	0.0721	5.6032	Dinitrosopentamethylenetetramine	5.524782	-	-	-	39.35935	187.180205
POS07653	402661.6679	270148.7665	-0.57581	0.019878	0.238	0.31463	Nebularine	15.932702	-	-	-	39.35935	253.237995
POS07658	1551867.227	2083354.364	0.424903	0.379629	0.6536	0.42117	Vicianose	-16.597893	-	-	-	39.34855	313.272594
POS07659	1376496.532	1174270.12	-0.22924	0.046314	0.3134	0.33344	2-C-Methyl-D-erythritol 4-phosphate	-2.0632657	-	-	-	39.34855	217.133131
POS07663	2071487.131	1358363.05	-0.6088	0.485273	0.728	0.4405	Pyrazinoic acid	20.157342	-	-	-	39.3484	125.107278
POS07665	633834.7898	349133.7609	-0.86033	0.004147	0.1373	0.51584	Danielone	11.159251	-	-	-	39.3484	213.208945
POS07668	145092.4453	87845.8271	-0.72393	0.012963	0.2101	0.22093	(3S)-3-Hydroxy-L-enduracididine	21.180376	-	-	-	39.3481	189.195762
POS07671	1699847.116	1832625.173	0.108507	0.695944	0.8501	0.14969	3,4,5-trihydroxy-6-{[3-hydroxy-2-(hydroxymethyl)-2	-17.183805	-	-	-	39.3477	311.256945
POS07672	349145.0074	196450.3413	-0.82966	0.006	0.1639	0.34881	Creatinine	21.515013	-	-	-	39.34755	114.12761
POS07674	6062619.584	3620271.85	-0.74384	0.000872	0.0734	1.53254	Sepiapterin	19.566937	-	-	-	39.347	238.227218
POS07679	3168361.513	1865733.17	-0.76399	0.000767	0.0721	1.12406	5-O-Methyl-myo-inositol	-22.906262	-	-	-	39.347	195.185329
POS07681	196788.2375	154483.5533	-0.34919	0.484534	0.7272	0.09621	Mecarphon	14.227728	-	-	-	39.347	272.305236
POS07693	6893126.528	3328897.677	-1.05011	0.000538	0.0721	1.87258	2-Amino-5-(5-nitro-2-furyl)-1,3,4-thiadiazole	11.602853	-	-	-	39.3449	213.195739
POS07694	10986698.57	5386473.522	-1.02834	0.00052	0.0721	2.35697	2-Aminophenoxazin-3-one	16.907073	-	-	-	39.3449	213.214964
POS07695	39534922.7	16198577.03	-1.28726	0.000403	0.0721	4.82701	Dihydrobiopterin	18.264955	-	-	-	39.3449	240.242846
POS07698	141317.1747	64508.12189	-1.13138	0.005896	0.1617	0.24951	Disperse Blue 1	-2.670246	-	-	-	39.3434	269.27726

POS07700	340182.5079	185243.7445	-0.87688	0.005682	0.1571	0.36403	5-Methyl-3-isoxazolyl sulfate	18.847195	-	-	-	39.3429	180.161853
POS07701	1424317.856	1242682.321	-0.19681	0.047117	0.3141	0.30271	3-Hydroxy-2-methylpyridine-4,5-dicarboxylate	-18.134162	-	-	-	39.34285	198.148602
POS07703	716454.0439	438447.6436	-0.70847	0.005092	0.1518	0.49832	Vicine	16.511881	-	-	-	39.3428	305.2689
POS07706	317481.2686	267995.0801	-0.24446	0.303292	0.6157	0.13044	Neopterin	0.0898745	-	-	-	39.3428	254.221999
POS07707	558213.5551	489877.6185	-0.1884	0.155246	0.4838	0.18221	Pancuronium	-19.600812	-	-	-	39.3428	573.857948
POS07709	1365373.059	1028205.422	-0.40917	0.001803	0.0964	0.54586	Phosphoagmatine	6.2973251	-	-	-	39.34275	211.18
POS07710	1087461.218	681564.1706	-0.67404	0.001982	0.0995	0.62168	6-Succinoaminopurine	20.383199	-	-	-	39.34275	236.211571
POS07713	514240.2649	369036.7503	-0.47868	0.250864	0.5689	0.27132	SQ 26180	20.236075	-	-	-	39.3427	239.230497
POS07725	404345.3529	276330.3037	-0.54919	0.026299	0.2576	0.30521	Fluconazole	21.147839	-	-	-	39.341	307.284554
POS07726	416004.9403	411726.3039	-0.01492	0.962146	0.9827	0.0449	Glyphosate-isopropylammonium	-0.4450839	-	-	-	39.3404	229.190475
POS07727	293451.7566	731125.9052	1.316997	0.454036	0.7077	0.36247	Guanine	-17.882805	-	-	-	39.3404	152.130674
POS07728	790100.4778	348070.1616	-1.18266	0.000278	0.0721	0.66833	Omethoate	-0.2749548	-	-	-	39.3402	214.199018
POS07729	855799.6237	477473.5341	-0.84185	0.002479	0.1088	0.58021	Indicaxanthin	20.531811	-	-	-	39.3402	309.300206
POS07738	582113.9503	300793.0034	-0.95253	0.013649	0.2159	0.46668	2-{[5,7-dihydroxy-2-(3-hydroxyphenyl)-5H-chromen-3-yl]oxy}oxane	7.0827265	-	-	-	39.337	404.373134
POS07742	211132.1058	142331.2907	-0.56889	0.316331	0.6253	0.15066	Ethalfluralin	7.1223264	-	-	-	39.3215	334.27285
POS07744	148461.2191	128268.8388	-0.21092	0.501324	0.7379	0.06369	SN38 glucuronide carboxylate form	-8.5597276	-	-	-	39.3209	586.538365
POS07746	764996.113	483970.4169	-0.66053	0.006114	0.1646	0.49594	Taxifolin	-2.7716704	-	-	-	39.3094	305.258033
POS07750	795271.1685	624598.531	-0.34852	0.133828	0.4541	0.2586	Lormetazepam	6.5609377	-	-	-	39.28135	336.194476
POS07753	821318.6712	652120.2114	-0.3328	0.398356	0.6673	0.25425	L-Asp(4-L-Arg)	-1.0036333	-	-	-	39.28135	290.295386
POS07754	189499.55	98856.01843	-0.93879	0.357072	0.6405	0.17945	UDP-L-Ara4N	11.418431	-	-	-	39.2745	536.304389
POS07758	1252144.301	1215537.184	-0.04281	0.968161	0.9856	0.15954	Phosmet	-5.0931431	-	-	-	39.264	318.32666
POS07759	165024.4641	59385.77662	-1.47449	0.007974	0.1842	0.28848	Dihydroxycarbazepine	8.6222119	-	-	-	39.2617	271.292907
POS07760	379274.3238	590471.2244	0.638625	0.42502	0.6851	0.28788	Robustine	12.485287	-	-	-	39.2454	216.214763

POS07761	1777983.849	195601.9963	-3.18425	0.312782	0.6229	0.68461	Methacrifos	23.711912	-	-	-	39.22725	241.226873
POS07762	1671798.559	1306719.943	-0.35545	0.043392	0.3066	0.4538	N-Acetyldemethylphosphinothricin	20.881524	-	-	-	39.2265	210.148544
POS07765	3010491.806	2431866.5	-0.30794	0.049402	0.3185	0.59194	N-Dimethyl-2-aminoethylphosphote	-9.7509363	-	-	-	39.2095	154.122484
POS07768	211071.0259	196157.9207	-0.10571	0.813208	0.9151	0.01572	Lumichrome	7.7319524	-	-	-	39.1926	243.24255
POS07771	24276037.6	17636418.45	-0.46098	0.005354	0.1543	2.30734	Calcium hydroxide	-3.9031645	-	-	-	39.1853	75.0996874
POS07774	116225.3103	381053.313	1.713069	0.234384	0.5554	0.31981	Auranofin	-12.632597	-	-	-	39.1853	679.478706
POS07775	164626.2324	74429.16578	-1.14525	0.003723	0.1314	0.27721	Dyphylline	14.752613	-	-	-	39.1807	255.253627
POS07778	975435.0888	626690.3169	-0.63829	0.014997	0.2222	0.51662	N,N'-dinitrosopiperazine	16.385488	-	-	-	39.16755	145.141538
POS07780	80284.66815	70959.2598	-0.17813	0.691693	0.8473	0.02098	Amaroswerin	10.82919	-	-	-	39.1499	603.553902
POS07781	795305.4013	386949.7185	-1.03936	0.495012	0.7341	0.27065	N-Benzoylanthranilate	1.3924523	-	-	-	39.1498	242.249613
POS07787	4348793.995	3034336.977	-0.51923	0.204316	0.5278	0.79448	N(beta)-Epoxysuccimoyl-DAP-Val	18.039739	-	-	-	39.1329	317.323383
POS07790	22609744.38	15977010.36	-0.50095	0.233983	0.555	1.73841	Hippeastrine	-24.779856	-	-	-	39.1329	316.320063
POS07792	290764.838	127572.0835	-1.18854	0.114439	0.4282	0.27704	{2-[3,5-dihydroxy-4-(sulfooxy)phenyl]-3-hydroxy-5-oxo-5H	5.6813723	-	-	-	39.12815	384.309454
POS07794	83802.47329	52416.63073	-0.67697	0.024409	0.2524	0.14568	Olsalazine	23.358902	-	-	-	39.12355	303.253337
POS07795	115137.2939	88816.03399	-0.37446	0.338303	0.6326	0.10439	Leucodelphinidin	15.70676	-	-	-	39.116	323.279238
POS07796	1132289.897	950242.0806	-0.25288	0.770764	0.8941	0.09168	2-Amino-3,4-dihydroxypentanedioic acid	14.986017	-	-	-	39.116	180.138061
POS07800	1304950.803	972449.3617	-0.4243	0.013924	0.2168	0.51665	Quite	-22.174694	-	-	-	39.1125	193.169615
POS07801	512301.8907	518621.0923	0.017687	0.969362	0.9861	0.00604	Triphenyl phosphate	-6.3124341	-	-	-	39.1125	327.288317
POS07802	439292.7871	236194.1546	-0.89521	0.16892	0.4949	0.28404	Nedocromil	-5.9518692	-	-	-	39.1125	372.345866
POS07806	48482.7034	5570990.591	6.84432	0.340012	0.6326	1.02908	Flurochloridone	15.173404	-	-	-	39.109	313.127112
POS07809	1687355.881	1181607.979	-0.51401	0.035031	0.2838	0.59059	2-Amino-4-oxo-6-(1',2'-dioxoprolyl)-7,8-dihydroxypteridine	20.927988	-	-	-	39.109	268.211169
POS07812	3175824.672	1798992.812	-0.81994	0.009921	0.194	1.07143	1,3,7-Trimethyluric acid	10.34661	-	-	-	39.1061	211.199451
POS07813	189809.3483	95908.99683	-0.98481	0.011608	0.2019	0.2672	4a-Hydroxytetrahydrobiopterin	-2.4804014	-	-	-	39.1061	258.253139

POS07815	454296.1642	298804.3895	-0.60443	0.107637	0.4173	0.26858	Nedaplatin	6.8435614	-	-	-	39.1022	304.189851
POS07820	247664.5419	179818.0887	-0.46185	0.098689	0.4049	0.20345	TG(15:0/20:4(5Z,8Z,11Z,14Z)/24:1(15Z))	10.633342	-	-	-	39.09285	952.573395
POS07824	197846.9388	329324.5133	0.735125	0.477601	0.7223	0.11915	PQQ	-14.564882	-	-	-	39.0835	331.208467
POS07828	947751.5016	852499.0808	-0.15281	0.439789	0.6968	0.17493	dTDP-4-dimethylamino-4,6-dideoxy-5-C-methyl-L-mannose	-12.825351	-	-	-	39.0726	590.424217
POS07833	1211737.319	2061651.777	0.766724	0.483809	0.7265	0.3172	Trans-urocanate	10.260679	-	-	-	39.0617	138.127684
POS07835	1503166.075	1550727.397	0.044941	0.842202	0.9279	0.10892	Calcium glycerophosphate	3.3054875	-	-	-	39.061	211.143771
POS07836	3722652.915	2275198.583	-0.71034	0.008897	0.1917	1.02447	p-Benzoquinone	-8.4582987	-	-	-	39.0566	109.101162
POS07844	194429.3466	61515.34129	-1.66023	0.282259	0.5981	0.19809	ringenin 7-O-beta-D-glucoside	-17.368071	-	-	-	39.0315	435.393132
POS07847	1663205.276	809585.4486	-1.03871	0.000588	0.0721	0.921	Desacetyl-nitazoxanide	22.383639	-	-	-	39.03105	266.258214
POS07849	1434518.474	1048948.463	-0.45162	0.013482	0.2149	0.56238	Cytosine	23.822552	-	-	-	39.0309	112.111923
POS07851	1542483.743	1328390.57	-0.21558	0.445622	0.7007	0.21499	Clavulanic acid	-19.211351	-	-	-	39.03005	200.16425
POS07854	457355.0987	512717.2767	0.164849	0.676783	0.8402	0.11946	Bromodiphenhydramine	-3.720037	-	-	-	39.0286	335.257033
POS07857	350500.7892	293305.8699	-0.25701	0.76122	0.8886	0.09862	Xanthopterin-B2	16.294541	-	-	-	39.0231	239.211258
POS07858	546611.6398	381358.5112	-0.51937	0.207987	0.532	0.27526	Propyzamide	1.7010988	-	-	-	39.0228	257.135512
POS07862	95301.70845	327835.949	1.7824	0.271231	0.5886	0.24055	Trifluralin	6.821754	-	-	-	39.02265	336.288664
POS07863	967141.4052	374895.6616	-1.36724	0.197593	0.5233	0.48577	3,4,5-trihydroxy-6-(4-hydroxy-1H-indole-3-carbonyloxy)oxane-2	24.673743	-	-	-	39.02195	354.298993
POS07864	468278.8607	603725.5337	0.366525	0.713443	0.8606	0.14707	Pyrimidine nucleoside	3.3205677	-	-	-	39.01955	214.218485
POS07866	538128.9933	531573.1332	-0.01768	0.964346	0.9834	0.03777	Irisxanthone	-2.3361949	-	-	-	39.0147	437.372457
POS07870	617721.6473	237042.0767	-1.38181	0.104715	0.4117	0.43759	2-Pyrone-4,6-dicarboxylate	-17.881531	-	-	-	38.9962	185.107085
POS07871	748361.4151	301920.9902	-1.30956	0.277236	0.5932	0.36845	(1E,3E)-4-Hydroxybuta-1,3-diene-1,2,4-tricarboxylate	8.8913166	-	-	-	38.9866	203.127374
POS07873	9960443.524	7288363.943	-0.45061	0.064744	0.3459	1.31697	2,4-dihydroxy-3-(sulfooxy)benzoic acid	-10.353244	-	-	-	38.96175	251.184686
POS07874	236394.0834	261228.7271	0.14412	0.521489	0.7515	0.04605	Selenomethionine Se-oxide	-15.859338	-	-	-	38.95765	213.109613
POS07875	1634676.406	6997720.151	2.09788	0.376312	0.6512	0.95202	3,5-Dichloro-L-tyrosine	19.265339	-	-	-	38.94575	251.090794

POS07879	46487.09498	49001.23969	0.075988	0.858324	0.9354	0.02113	Bay-K-8644	17.437264	-	-	-	38.9137	357.309989
POS07880	49191124.62	22443126.22	-1.13212	0.000525	0.0721	5.14773	Deoxyvasicinone	-3.0381704	-	-	-	38.9133	187.216611
POS07889	9973.420899	23584.15042	1.241657	0.214568	0.5366	0.0875	N,N'-Diacetylchitobiose 6'-phosphate	0.1365533	-	-	-	38.82415	505.387545
POS07891	178034.8072	170548.8204	-0.06197	0.932723	0.9693	0.00092	[1-(6,7-dimethoxy-2H-1,3-benzodioxol-5-yl)-3-oxopropoxy]sulfonic acid	24.398484	-	-	-	38.7992	335.315433
POS07894	65057.29894	77795.91188	0.257983	0.792702	0.9047	0.05454	Phloroacetophenone 6'-[xylosyl-(1->6)-glucoside]	-13.923939	-	-	-	38.7886	491.455548
POS07898	16175036.56	5534510.451	-1.54724	0.358272	0.6416	2.12749	9-Carboxymethoxymethylguanine	-0.6581917	-	-	-	38.7717	240.195319
POS07902	137897.1811	80829.63318	-0.77064	0.015862	0.2226	0.22127	8-Oxodeoxycoformycin	3.908286	-	-	-	38.7535	267.261617
POS07906	956120.0382	873952.8473	-0.12964	0.447446	0.7021	0.1325	Stipitatate	2.173374	-	-	-	38.7176	183.137872
POS07909	390517.7685	276089.1659	-0.50025	0.421073	0.682	0.20975	3-[3-hydroxy-5-methoxy-4-(sulfooxy)phenyl]prop-2-enoic acid	-17.334778	-	-	-	38.7171	291.242245
POS07914	237039.6175	320887.0817	0.436938	0.16142	0.4889	0.19006	Corilagin	-12.459172	-	-	-	38.7072	635.452172
POS07915	216737.0578	119429.9817	-0.85978	0.089523	0.3895	0.22069	Prodiamine	24.494556	-	-	-	38.7058	351.309557
POS07916	752471.174	579167.4919	-0.37766	0.152049	0.4792	0.28251	(4-{7-[3,5-dimethoxy-4-(sulfooxy)phenyl]-6,11-dihydroxy-2,8-	18.386279	-	-	-	38.70485	528.496975
POS07919	196371.7287	136151.3334	-0.52838	0.310408	0.6217	0.13312	[2-methoxy-4-(3,5,7-trihydroxy-5H-chromen-2-yl)phenyl]oxidanesulfonic	-18.826215	-	-	-	38.6998	382.330098
POS07920	418773.051	298943.3362	-0.4863	0.024778	0.2538	0.28816	Melizame	-7.1737807	-	-	-	38.68265	179.154099
POS07921	218761.444	154369.2114	-0.50297	0.318449	0.6268	0.12229	Dichlofluanid	-3.9243114	-	-	-	38.6795	334.236169
POS07924	1274470.472	1052769.001	-0.27571	0.284702	0.6008	0.29561	3-Fluorocyclohexadiene-cis,cis-1,2-diol-1-carboxylate	-5.886435	-	-	-	38.63585	175.132752
POS07925	1081204.701	741837.5199	-0.54346	0.087213	0.3863	0.46129	Aminomalote	-22.294786	-	-	-	38.6141	120.080722
POS07930	2114519.136	3014941.832	0.511801	0.472494	0.7195	0.32007	3-Bromo-2Z-heptenoic acid	-19.807702	-	-	-	38.5443	208.068175
POS07933	58404.65998	38423.54295	-0.60409	0.529016	0.7568	0.05368	6-(5-{[(2E)-3-(3,4-dihydroxyphenyl)prop-2-enoyl]oxy}-3,4-	-20.239833	-	-	-	38.5368	513.416905
POS07936	593621.3838	448202.3094	-0.40539	0.46148	0.712	0.19992	2-hydroxy-3-(sulfooxy)butanedioic acid	7.0348671	-	-	-	38.5293	231.148896
POS07948	968418.824	804828.4843	-0.26695	0.047273	0.3142	0.31363	D-Glucono-1,5-lactone	-24.883484	-	-	-	38.4134	179.142844
POS07949	200815.5004	220303.8443	0.133624	0.756836	0.887	0.03518	rciclasine	-17.222253	-	-	-	38.40935	308.257485
POS07957	875280.7543	239520.5637	-1.8696	0.173866	0.5001	0.47999	2-Hydroxy-4-trifluoromethyl benzoic acid	-18.084662	-	-	-	38.39225	207.122249

POS07960	2405465.541	1947942.558	-0.30436	0.197142	0.5228	0.4579	9-Riburonosyladenine	-18.629506	-	-	-	38.3907	282.226838
POS07961	872188.5092	770765.2862	-0.17835	0.307079	0.6189	0.17383	Aurasperone D	13.569995	-	-	-	38.3907	557.531129
POS07964	129240.9039	112178.009	-0.20427	0.4177	0.6797	0.06173	Deoxyloganic acid tetraacetate	-19.524608	-	-	-	38.3896	529.500058
POS07965	565603.7933	547057.9767	-0.0481	0.879026	0.9463	0.0164	Tricrozarin A	17.077073	-	-	-	38.3893	295.226001
POS07966	214482.5816	238011.9158	0.150173	0.830981	0.9228	0.06359	Kolaflavanone	23.771711	-	-	-	38.3893	589.536367
POS07968	2338308.789	1745532.626	-0.4218	0.00257	0.1099	0.67236	L-dopachromate	1.2619503	-	-	-	38.3804	193.158519
POS07969	192968.3021	197951.7435	0.036785	0.875806	0.945	0.01613	alpha-D-Galactopyranuronosyl-(1->4)-alpha-D	11.985465	-	-	-	38.3752	547.401425
POS07970	1057038.934	610151.9086	-0.79279	0.045625	0.3126	0.55744	4-Amino-2,6-dinitrotoluene	5.4105933	-	-	-	38.3736	198.156543
POS07973	1300582.122	666655.1898	-0.96414	0.124608	0.4428	0.53698	D-Ribitol 5-phosphate	-21.725331	-	-	-	38.3224	233.127934
POS07979	199995.0051	127670.7185	-0.64754	0.200067	0.5255	0.175	Flumioxazin	-24.055459	-	-	-	38.2045	355.330553
POS07986	734590.711	543107.6654	-0.4357	0.298497	0.6115	0.22647	Fenclorim	19.732739	-	-	-	38.1601	226.085718
POS07990	1364546.644	1053209.434	-0.37363	0.060455	0.3385	0.42841	Decuroside III	-0.0837425	-	-	-	38.14965	571.547029
POS07991	518417.9639	361391.0558	-0.52055	0.143026	0.4676	0.26184	Chlorogete	10.523943	-	-	-	38.1458	355.319705
POS07996	4649288.006	2344240.448	-0.98789	0.023815	0.2497	1.25844	3-Nitroacrylate	-20.724228	-	-	-	38.12345	118.065051
POS07997	225485.8378	230521.227	0.031863	0.898746	0.9533	0.02312	Dimethylaminoethyl reserpilite dihydrochloride	24.299631	-	-	-	38.1153	543.515559
POS07998	129670.6829	92509.83629	-0.48717	0.160517	0.4881	0.11912	2,3,4-trihydroxy-5-(3,4,5-trihydroxybenzoyloxy)benzoic acid	2.6928386	-	-	-	38.11465	339.232187
POS08001	4698435.688	3920136.987	-0.26128	0.068825	0.3541	0.65502	Flaviolin	-0.1790693	-	-	-	38.09725	207.15884
POS08004	311114.3625	243634.7067	-0.35273	0.563501	0.7763	0.13457	Cyanophos	-0.1843153	-	-	-	38.0782	244.226632
POS08005	104168.1089	236833.9572	1.184962	0.317484	0.6257	0.16721	L-Dopachrome	-9.167487	-	-	-	38.07735	194.161706
POS08006	379096.5618	283510.0067	-0.41917	0.354575	0.6386	0.18535	2,2-Bis(4-hydroxyphenyl)hexafluoropropane	-0.3379012	-	-	-	38.0772	337.236263
POS08007	222729.5674	192157.8355	-0.213	0.61701	0.8097	0.06002	2-Oxoglutaramate	-22.039337	-	-	-	38.0751	146.117478
POS08008	721298.2375	591699.3293	-0.28573	0.235904	0.5559	0.21825	(Z)-But-1-ene-1,2,4-tricarboxylate	-19.395949	-	-	-	38.0751	189.138428
POS08009	470383.7223	400872.1828	-0.2307	0.389805	0.6601	0.12355	2-C-Methyl-D-erythritol 2,4-cyclodiphosphate	-19.503259	-	-	-	38.0741	279.092753

POS08014	505131.7706	482044.4712	-0.06749	0.794862	0.9059	0.02109	Pelargonidin 3-O-rutinoside	-9.2481946	-	-	-	38.0604	580.528517
POS08015	1289197.249	922454.1587	-0.48292	0.189897	0.5169	0.37242	Chlorfenprop-methyl	-17.445492	-	-	-	38.0604	234.09441
POS08016	1512518.432	1107290.571	-0.44992	0.178836	0.5052	0.41135	Isosorbide dinitrate	-22.870793	-	-	-	38.0359	237.138176
POS08017	1	480219.1833	18.87333	0.340893	0.6326	0.303	ATP	-17.119447	-	-	-	38.0236	508.179594
POS08020	366655.2039	225843.3841	-0.6991	0.216229	0.5379	0.22341	{[4-(7-methoxy-2-oxo-2H-chromen-6-yl)-2-oxobut-3-en-1-yl]oxy}sulfonic acid	20.46107	-	-	-	37.9925	341.31424
POS08021	801487.3092	700677.0672	-0.19393	0.310772	0.6218	0.17047	(R)-5-Phosphomevalote	-14.33316	-	-	-	37.9629	229.141007
POS08026	192799.2926	156284.5521	-0.30292	0.3501	0.6362	0.10474	6,11-dihydroxy-3-methyl-7-(3,4,5-trihydroxyphenyl)-28	-6.411271	-	-	-	37.9461	342.299088
POS08032	1378169.821	1746018.048	0.341315	0.490368	0.7313	0.31957	Bromobutide	10.796125	-	-	-	37.8912	313.255948
POS08034	41157.76244	53950.16608	0.390463	0.378833	0.653	0.07287	(2-hydroxy-2-{9-hydroxy-7-oxo-2H,3H,7H-furo[3,2-g]chromen-2-	-5.6520273	-	-	-	37.8912	359.325251
POS08040	408643.6292	310434.3095	-0.39656	0.366365	0.646	0.15172	N-Acetyl-D-glucosaminyldiphosphodolichol	-15.453052	-	-	-	37.8648	588.532697
POS08047	338804.3359	1088879.887	1.684321	0.331569	0.6326	0.38473	2,6-Dichloroindophenol	-4.8286336	-	-	-	37.8424	269.101482
POS08049	349893.535	287287.0574	-0.28442	0.488627	0.73	0.09477	2-{[(2,4-dihydroxy-3-methoxyphenyl)(hydroxy)methylidene]amino}acetic	20.7994	-	-	-	37.8384	242.211293
POS08050	308455.2723	336737.5903	0.126563	0.883507	0.9474	0.06655	C.I. Acid red 33	-6.7255105	-	-	-	37.8384	468.388133
POS08053	378413.9797	243038.9768	-0.63878	0.454265	0.7079	0.21299	Pyridoxal phosphate	-24.597551	-	-	-	37.81075	248.143098
POS08055	657938.8623	448550.4099	-0.55268	0.549499	0.769	0.20701	Morfamquat	-7.5399461	-	-	-	37.7987	540.497609
POS08058	3582161.212	1714402.412	-1.06312	0.203133	0.5274	0.93812	3-Sulfomucote	0.8609298	-	-	-	37.7881	223.180068
POS08066	283578.4875	227803.8846	-0.31596	0.418022	0.6797	0.12417	Endalin	-19.494605	-	-	-	37.7624	230.211108
POS08068	17383051.71	9993941.594	-0.79856	0.224396	0.5461	1.55908	Thioguanosine 5'-diphosphate	-11.763759	-	-	-	37.7618	460.267874
POS08071	2011024.785	1580456.743	-0.34759	0.054904	0.3269	0.48399	5'-Hydroxystreptomycin	-9.7465483	-	-	-	37.7612	598.574952
POS08072	475126.292	296558.8284	-0.67999	0.105025	0.412	0.32673	5'-Phosphoribosylglycimide	-4.7171667	-	-	-	37.7612	287.182327
POS08073	513435.1362	211488.6125	-1.2796	0.10187	0.4083	0.37579	2,3-Diketo-5-methylthiopentyl-1-phosphate	3.6205802	-	-	-	37.7608	243.194853
POS08074	1	276698.1042	18.07795	0.340893	0.6326	0.23	Silver sulfadiazine	-24.333479	-	-	-	37.7608	358.135586
POS08075	731261.4249	648639.0355	-0.17297	0.465303	0.7147	0.12927	10-Acetoxyligustroside	-19.134293	-	-	-	37.7607	583.54663

POS08078	555170.5569	522376.5566	-0.08784	0.680636	0.8427	0.05978	SN38 glucuronide	-8.3299133	-	-	-	37.7601	569.531241
POS08079	134647.3179	129260.3522	-0.05891	0.891566	0.9503	0.0158	N-Amidino-L-aspartate	7.933216	-	-	-	37.759	176.151366
POS08080	4681853.911	2726302.081	-0.78013	0.230059	0.5513	0.79477	CDP-N-methylethanolamine	-14.93909	-	-	-	37.759	461.271201
POS08081	1352265.311	1086900.506	-0.31516	0.119523	0.4348	0.32837	2-Methyl-3-hydroxy-5-formylpyridine-4-carboxylate	6.3992039	-	-	-	37.7586	182.153936
POS08083	529509.7258	472835.0038	-0.16332	0.373374	0.6502	0.12025	sn-Glycero-3-phospho-1-inositol	-2.4703795	-	-	-	37.7586	335.220751
POS08084	1033552.124	667268.874	-0.63127	0.07462	0.3661	0.45416	3-Carboxy-cis,cis-mucote	-18.761588	-	-	-	37.7574	187.122685
POS08087	445069.5836	385271.0687	-0.20816	0.352166	0.6364	0.11643	Sulbactam sodium	-16.178246	-	-	-	37.741	256.226648
POS08092	53903.87302	70132.87496	0.379702	0.321352	0.6288	0.08769	2-Decarboxybetanin	-6.978683	-	-	-	37.68805	508.471035
POS08094	990506.8796	772251.9896	-0.3591	0.053171	0.3235	0.36969	4-Carboxy-4-hydroxy-2-oxoadipate	-13.512222	-	-	-	37.672	221.137902
POS08095	324975.3696	263904.804	-0.30031	0.237803	0.5574	0.14261	Deoxy-5-methylcytidylate	18.185846	-	-	-	37.6668	322.236818
POS08098	339693.5384	200277.7426	-0.76223	0.107833	0.4177	0.24306	Decarbamoylgonyautoxin 1	11.536131	-	-	-	37.6491	369.335226
POS08100	318135.5609	229142.2682	-0.4734	0.077226	0.371	0.24682	Se-Methylselenomethionine	-4.3407129	-	-	-	37.6171	212.14726
POS08104	909240.9772	275338.2582	-1.72346	0.269187	0.5861	0.5386	2,4'-Dichlorobiphenyl	6.6149222	-	-	-	37.5981	224.106652
POS08108	381393.1882	208195.1811	-0.87334	0.157112	0.4855	0.26012	U50488	-14.404065	-	-	-	37.5653	370.330457
POS08110	2745888.637	1693652.344	-0.69714	0.030155	0.2685	0.82783	Panfuran S	-2.5543006	-	-	-	37.545	294.242028
POS08113	40616.88374	28573.37654	-0.50741	0.342451	0.6327	0.05877	12alpha-Bromo-11beta-hydroxypregn-4-ene-3,20-dione	-7.2399644	-	-	-	37.53555	410.361513
POS08114	139779.4359	143591.6949	0.03882	0.910575	0.9581	0.02768	6-({2-[4-ethyl-3-(sulfooxy)phenyl]-3,5-dihydroxy-3,4-dihydro	-0.0145098	-	-	-	37.5247	559.517268
POS08117	1	1369822.997	20.38556	0.340893	0.6326	0.51175	TG(14:0/20:3(5Z,8Z,11Z)/14:0)	10.653251	-	-	-	37.5247	802.288413
POS08121	1297122.714	985059.4982	-0.39703	0.034066	0.2814	0.419	[(6-{[5,7-dihydroxy-2-(4-oxocyclohexa-2,5-dien-1-ylidene)-2H-chromen-3	14.004956	-	-	-	37.51305	610.574813
POS08127	878987.6815	460008.1304	-0.93418	0.02259	0.2473	0.52266	Lamivudine-monophosphate	-20.676554	-	-	-	37.5014	310.236883
POS08129	5446483.671	3659839.766	-0.57354	0.027943	0.2623	1.14941	Tulobuterol hydrochloride	6.447721	-	-	-	37.49975	265.20038
POS08130	1704239.478	1488663.605	-0.19511	0.209841	0.5333	0.30051	Oxaloglutarate	8.4765583	-	-	-	37.49975	205.143207
POS08133	158629.9336	145946.9827	-0.12022	0.652965	0.8291	0.03683	Bluensomycin	-1.842714	-	-	-	37.4981	586.565698

POS08137	487133.5768	470590.9282	-0.04984	0.753232	0.8848	0.04575	7,8-Dihydroxykynurete	20.190492	-	-	-	37.473	222.178042
POS08140	250395.0331	144416.0774	-0.79397	0.188421	0.5153	0.19186	NA	-3.2223043	-	-	-	37.469	364.28345
POS08142	55638.07937	38411.77891	-0.53452	0.146184	0.4716	0.08423	Puerarin xyloside	9.5732865	-	-	-	37.4552	549.505227
POS08144	187453.4285	199646.3869	0.090915	0.879933	0.9466	0.05084	Sodium picosulfate	-23.481094	-	-	-	37.44645	482.403273
POS08145	131783.2459	133173.8219	0.015144	0.956032	0.9806	0.00082	Lippioside II	19.362956	-	-	-	37.4459	555.515313
POS08149	546677.9336	396487.4585	-0.46342	0.098164	0.4046	0.28177	Trinitrotoluene	-1.5315801	-	-	-	37.4402	228.138029
POS08150	439226.987	1157262.551	1.397678	0.168362	0.4947	0.5795	Phenyl 5-phospho-alpha-D-ribofuranoside	-8.4308822	-	-	-	37.4402	307.210495
POS08151	100484.0204	210791.049	1.068848	0.318962	0.6272	0.15242	2-(Formamido)-N1-(5'-phosphoribosyl)acetamidi[illegible]	5.663066	-	-	-	37.4382	314.21075
POS08156	77021.63672	49968.87504	-0.62423	0.578144	0.7846	0.06173	Sodium folite	-18.973782	-	-	-	37.4377	496.418977
POS08158	1313498.56	938859.1073	-0.48443	0.01688	0.2275	0.53322	Barbiturate	-19.870926	-	-	-	37.4366	129.090931
POS08161	173883.7628	163027.0928	-0.09301	0.874915	0.9448	0.06472	dUMP	2.3978334	-	-	-	37.43635	309.189916
POS08167	153642.209	226964.8775	0.562894	0.296761	0.6103	0.12318	Pelargonidin 3-O-beta-D-sambubioside	10.094153	-	-	-	37.4345	566.513085
POS08169	415697.5639	337321.2881	-0.30141	0.121855	0.4395	0.17359	Neohesperidin dihydrochalcone	16.660485	-	-	-	37.434	613.593882
POS08171	1294402.38	1171381.719	-0.14407	0.414693	0.6784	0.15145	Sulfite	-5.3258053	-	-	-	37.4314	81.0698502
POS08172	34545.65759	30785.70292	-0.16624	0.79017	0.9034	0.00384	6-[2,3-dihydroxy-5-(3,5,7-trihydroxy-3,4-dihydro-2H-1-benzopyran-2-[illegible]	9.6290975	-	-	-	37.4314	483.405922
POS08173	133083.4598	143622.3664	0.109949	0.838834	0.9266	0.02753	3,4,5-trihydroxy-6-{2-[9-hydroxy-2-oxo-2H,8H,9H-furo[2,3-h]chromen-8-yl]-[illegible]	22.374234	-	-	-	37.4314	537.50528
POS08175	462384.1207	339762.5127	-0.44457	0.304844	0.6169	0.23252	N-Pyruvoyl-5-methoxy-3-hydroxyanthranilate	-17.311718	-	-	-	37.41635	254.210993
POS08177	900154.0955	723469.4917	-0.31524	0.389223	0.6599	0.28153	Pelargonidin	20.983866	-	-	-	37.4147	272.257768
POS08179	403710.4664	365712.5899	-0.14261	0.533589	0.7588	0.05732	Hesperidin	17.001989	-	-	-	37.4132	611.578257
POS08181	468418.3797	342739.4329	-0.45069	0.348229	0.6355	0.18959	Furcozole-cis	2.0218726	-	-	-	37.3987	397.199878
POS08185	959671.7492	800398.8825	-0.26182	0.363918	0.6446	0.20363	N-Phosphohypotaurocyamine	-23.478722	-	-	-	37.3919	232.169249
POS08188	260118.9726	187860.2384	-0.46951	0.351103	0.6364	0.1496	Lead nitrate	11.288213	-	-	-	37.3404	332.220815
POS08194	1742098.552	987389.4353	-0.81914	0.011182	0.1994	0.73012	trans-2-[(Dimethylamino)methylimino]-5-[2-(5-nitro-2-[illegible]	14.040666	-	-	-	37.2832	278.247269

POS08196	1918114.675	15568930.56	3.020909	0.352687	0.6367	1.57651	Chlorthiamid	9.0096909	-	-	-	37.2664	207.101433
POS08200	106161.2706	57783.05711	-0.87754	0.214017	0.5365	0.12883	Thiadiazolidinethione	-5.176799	-	-	-	37.2602	343.283305
POS08205	674046.8164	393469.2166	-0.7766	0.04211	0.3014	0.43542	5-Acetylamino-6-formylamino-3-methyluracil	15.632569	-	-	-	37.2596	227.200213
POS08207	1106526.486	1296131.284	0.228174	0.715187	0.8615	0.06441	O-Phospho-L-homoserine	2.1912532	-	-	-	37.25265	200.106813
POS08208	424793.2633	322406.5757	-0.39788	0.585836	0.7891	0.14899	Bretylium	-6.4583489	-	-	-	37.2429	244.169106
POS08212	284555.1512	211795.4394	-0.42604	0.388275	0.6594	0.14013	Bis(4'-chlorophenyl)acetate	-0.8565736	-	-	-	37.2256	282.141036
POS08224	1153653.99	978615.4071	-0.2374	0.570927	0.7802	0.13863	Musk xylene	-22.709255	-	-	-	37.1988	298.264526
POS08225	162943.9122	118648.7198	-0.45768	0.33821	0.6326	0.1132	2'-Deoxy-5-hydroxymethylcytidine-5'-diphosphate	21.205723	-	-	-	37.1988	418.219124
POS08229	448941.2065	414492.5456	-0.11518	0.630542	0.8165	0.06947	Cartormin	13.384667	-	-	-	37.1988	576.53308
POS08235	63638.4817	61049.14924	-0.05993	0.911057	0.9581	0.00642	Ethiprole	-17.66524	-	-	-	37.18585	398.20326
POS08237	256043.1985	213330.7921	-0.2633	0.244324	0.5624	0.11145	Diosmin	17.616891	-	-	-	37.17535	609.562697
POS08245	538272.2406	465123.909	-0.21072	0.313524	0.6237	0.13053	Fosaprepitant	-20.363253	-	-	-	37.1642	615.401365
POS08251	383104.6439	222115.8699	-0.78643	0.042265	0.302	0.31042	Velloquercetin	6.9429498	-	-	-	37.1478	369.346634
POS08254	352338.5754	147992.247	-1.25144	0.199195	0.5251	0.31669	Taxiphyllin	-18.343287	-	-	-	37.13625	312.288867
POS08255	318109.9038	239942.2627	-0.40684	0.045942	0.3134	0.21419	2-amino-4-({1-[(carboxymethyl)-C-hydroxycarbonimidoyl]-2	-3.985703	-	-	-	37.13625	622.574799
POS08258	952130.5406	883631.8525	-0.10771	0.741747	0.8784	0.04515	4-Pyridoxate	4.7818296	-	-	-	37.1227	184.169552
POS08261	391028.9237	222276.7294	-0.81492	0.075649	0.3687	0.31463	Profluralin	-24.67217	-	-	-	37.11315	348.288508
POS08267	435796.4103	362424.6922	-0.26597	0.271541	0.5887	0.15381	5'-Deoxy-5'-fluoroadenosine	9.2630567	-	-	-	37.1087	270.242171
POS08268	229604.1775	199488.3529	-0.20284	0.618982	0.8104	0.04821	2-(alpha-D-Mannosyl)-3-phosphoglycerate	-14.457086	-	-	-	37.1087	349.200043
POS08269	292968.5695	143586.3156	-1.02883	0.004589	0.1436	0.35072	Dinitramine	-18.938224	-	-	-	37.1077	323.241674
POS08273	286257.7658	201126.4206	-0.50921	0.010154	0.1952	0.24197	Forsythiaside	-1.447952	-	-	-	37.1064	625.593472
POS08275	472065.2231	419544.2943	-0.17016	0.510204	0.7445	0.09004	CGH 2466	3.0022979	-	-	-	37.10625	323.220644
POS08277	238904.2824	192279.4943	-0.31323	0.088514	0.3878	0.14035	Kuwanon L	-6.1301032	-	-	-	37.1051	627.609535

POS08280	324797.3372	260747.9215	-0.31688	0.528784	0.7568	0.09103	Phosphoguanidinoacetate	-12.998013	-	-	-	37.105	198.091215
POS08282	123578.2522	105215.8584	-0.23207	0.378649	0.6529	0.06959	6-{[5,7-dihydroxy-2-(4-hydroxyphenyl)-4-oxo-4H-chromen-3-yl]oxy}-4,5	24.455906	-	-	-	37.1038	595.546816
POS08286	667007.3076	335061.52	-0.99328	0.072184	0.3613	0.4293	Xanthurenate-8-O-beta-D-glucoside	-0.3126016	-	-	-	37.1013	368.314662
POS08287	91323.41752	88056.98451	-0.05255	0.865349	0.9391	0.00322	Genipin 1-beta-gentiobioside	11.602899	-	-	-	37.096	551.520764
POS08288	71216.17032	63803.43529	-0.15857	0.700317	0.8529	0.05071	dIDP	2.8383489	-	-	-	37.09165	413.194947
POS08289	446107.14	365754.0544	-0.28652	0.246643	0.5647	0.16289	N-[4-(5-Nitro-2-furyl)-2-thiazolyl]acetamide	21.696688	-	-	-	37.0857	254.247371
POS08297	247623.7022	209615.7325	-0.2404	0.35744	0.6409	0.09219	Mulberrofuran M	20.757074	-	-	-	37.06575	591.552034
POS08299	281634.0626	310289.4783	0.139793	0.557871	0.7733	0.04783	7,8-Dihydro-7,8-dihydroxykynurete	-18.536122	-	-	-	37.0614	224.18534
POS08304	984036.201	578334.2141	-0.76681	0.019094	0.2336	0.5115	S-Nitrosoglutathione	-0.9331791	-	-	-	37.04425	337.328963
POS08313	199114.7264	156844.7694	-0.34426	0.730892	0.8715	0.12536	6-Deoxy-6-sulfo-D-glucono-1,5-lactone	-3.349188	-	-	-	36.94075	243.210265
POS08320	268322.2004	155433.4995	-0.78767	0.036892	0.2883	0.2791	1-(4-Amino-2-methylpyrimid-5-ylmethyl)-3-(beta	-17.573585	-	-	-	36.9068	340.232215
POS08322	85454.92505	65600.80077	-0.38145	0.373179	0.6502	0.0718	2-{[(2E)-3-(3,4-dihydroxyphenyl)prop-2-enoyl]oxy}-3-[4-hydroxy	10.713142	-	-	-	36.90625	441.391994
POS08325	1	163606.4053	17.31987	0.340893	0.6326	0.17686	Cryptophycin 1	15.47061	-	-	-	36.8993	656.194913
POS08327	123886.3696	107282.8814	-0.2076	0.255969	0.5738	0.07579	Biotinyl-5'-AMP	-11.372245	-	-	-	36.8866	574.517354
POS08328	184112.606	144781.1182	-0.34671	0.204056	0.5278	0.1137	Aflatoxin B1exo-8,9-epoxide-GSH	-20.905546	-	-	-	36.886	636.590489
POS08329	211494.067	168663.3063	-0.32647	0.152082	0.4792	0.12493	Cefoperazone sodium	-5.8408706	-	-	-	36.886	668.652577
POS08333	1	1844163.122	20.81453	0.340893	0.6326	0.59378	dATP	-8.274898	-	-	-	36.8739	492.184812
POS08338	1	718975.4885	19.45558	0.340893	0.6326	0.37075	DG(24:0/0:0/22:4n6)	22.41408	-	-	-	36.8739	758.262249
POS08340	208001.2272	196874.9859	-0.07931	0.704209	0.8556	0.01838	Fluorocitric acid	7.012874	-	-	-	36.8733	211.12275
POS08347	454690.492	456477.9131	0.00566	0.979632	0.99	0.02263	UDP-2,4-bis(acetamido)-2,4,6-trideoxy-beta-L-altropyranose	-1.4441101	-	-	-	36.8564	633.412563
POS08351	27751.36819	20210.18363	-0.45748	0.371809	0.6502	0.0434	Pyranocyanin A	-9.9201383	-	-	-	36.8467	634.574991
POS08353	298759.0504	193470.4936	-0.62687	0.412503	0.6769	0.15766	S-methyl-5-thio-D-ribulose 1-phosphate(2-)	9.4099855	-	-	-	36.8438	259.189706
POS08354	92949.6806	71728.47096	-0.3739	0.16098	0.4884	0.0854	Orobanchoside	-1.0903347	-	-	-	36.8438	623.577898

POS08357	140084.3697	101087.5377	-0.47069	0.077156	0.371	0.13752	Kanokoside C	-15.958131	-	-	-	36.83735	639.609286
POS08359	44606.3203	44052.47344	-0.01803	0.957171	0.981	0.00438	3,4,5-trihydroxy-6-({7-methoxy-4-oxo-2-phenyl-8-[3,4,5-trihydroxy-6	7.6059582	-	-	-	36.8309	607.54489
POS08360	104519.2459	105999.764	0.020292	0.92867	0.9674	0.0098	Ditalimfos	-3.0384516	-	-	-	36.8309	300.288967
POS08364	1563433.145	1160500.733	-0.42997	0.04772	0.3143	0.46318	2-hydroxy-3-[4-hydroxy-3-(sulfooxy)phenyl]propanoic acid	-22.67764	-	-	-	36.81005	279.230967
POS08374	7849444.459	4234825.755	-0.89029	0.036553	0.2867	1.51414	Caffeoyl aspartic acid	19.02758	-	-	-	36.78485	296.257694
POS08377	466630.2503	436441.071	-0.09649	0.887254	0.9486	0.10532	3,4,5-trihydroxy-6-(4-hydroxy-1H-indole-3-carbonyloxy)oxane-2	-18.259055	-	-	-	36.7788	354.283826
POS08391	488054.9335	366816.5633	-0.41198	0.040749	0.2986	0.28895	D-Galactose 6-sulfate	-21.71451	-	-	-	36.7722	261.220726
POS08392	1158263.296	1029256.937	-0.17036	0.345387	0.6341	0.16286	2,4-Dihydroxypteridine	-9.4007718	-	-	-	36.7661	165.127334
POS08395	1291699.825	681596.4977	-0.92228	0.040381	0.2976	0.61691	DHAP(8:0)	-0.6705078	-	-	-	36.7629	297.261078
POS08397	4401472.729	540698.7754	-3.02509	0.004966	0.1496	1.70078	3,6,8-Trimethylallantoin	7.9020408	-	-	-	36.7586	201.204059
POS08400	415740.794	457689.1543	0.138684	0.562347	0.776	0.11732	Isosorbide dinitrate	18.567977	-	-	-	36.74625	237.147961
POS08402	645221.1359	699526.8208	0.116586	0.916418	0.9615	0.01001	D-Ribose 5-phosphate	-20.682822	-	-	-	36.7442	231.112317
POS08410	323983.8678	279307.392	-0.21407	0.344849	0.634	0.11258	4-Maleylacetoacetate	-22.23918	-	-	-	36.7202	201.148326
POS08411	151356.7377	168989.5015	0.158981	0.597313	0.7955	0.07991	Benzoylprop-ethyl	3.0645617	-	-	-	36.70105	367.246899
POS08413	236397.3919	233708.2673	-0.01651	0.924277	0.9658	0.00113	GDP-4-acetamido-4,6-dideoxy-alpha-D-mannose	-5.7331125	-	-	-	36.69115	631.397262
POS08418	5961393.726	3451039.802	-0.78862	0.708872	0.8579	0.39978	Convicine	-22.078876	-	-	-	36.65825	306.241937
POS08419	179464.9759	150309.3443	-0.25577	0.329531	0.6326	0.08987	5-Hydroxy-1-(4-sulfophenyl)-4-[(E)-(4-sulfophenyl)diazenyl]-1H	-4.0075499	-	-	-	36.65795	469.423399
POS08422	635776.246	481487.8013	-0.40102	0.175805	0.502	0.23615	thymidine 3'-monophosphate	9.4342646	-	-	-	36.64155	321.205297
POS08425	973599.5117	811116.1416	-0.26342	0.154744	0.4831	0.25361	norsertraline	0.1905628	-	-	-	36.6332	293.210332
POS08430	536087.4541	387441.1768	-0.46849	0.016687	0.2268	0.30845	1-Methylhypoxanthine	18.852708	-	-	-	36.5931	151.148107
POS08431	270485.7611	9995898.373	5.207712	0.340645	0.6326	1.36373	Uracil	18.465872	-	-	-	36.5872	113.096146
POS08440	181245.8209	129392.701	-0.48619	0.332401	0.6326	0.12088	Validamine 7-phosphate	-2.5830115	-	-	-	36.5632	258.184812
POS08455	67957.16997	22756.30106	-1.57836	0.003937	0.1352	0.18879	Coenzyme B	-19.386299	-	-	-	36.5465	344.334221

POS08457	6641.510355	1392390.98	7.711837	0.343098	0.6329	0.51289	Deltamethrin	-11.885816	-	-	-	36.5465	506.200472
POS08461	883225.7598	588588.0485	-0.58552	0.00036	0.0721	0.50103	D-Glycerate	8.6055732	-	-	-	36.5437	107.085489
POS08467	89958.56012	65757.60027	-0.4521	0.054876	0.3269	0.12218	Aventhramide A	-18.321382	-	-	-	36.52665	300.279893
POS08469	158735.6921	246718.9225	0.636242	0.404473	0.671	0.19603	Nitrofurantoin	-2.9288418	-	-	-	36.5164	239.163579
POS08470	534325.3723	453050.7282	-0.23805	0.426146	0.6861	0.11657	Halobetasol Propionate	8.2169427	-	-	-	36.512	485.971261
POS08472	3384020.218	2187509.137	-0.62945	0.084359	0.3821	0.77598	Psicofuranine	-4.1938113	-	-	-	36.512	298.27333
POS08473	197390.5806	135256.2563	-0.54536	0.159243	0.4871	0.16962	Estra-1,3,5(10)-triene-3,17beta-diol 3-phosphate	21.636217	-	-	-	36.512	353.3768
POS08476	105469.7725	100573.2837	-0.06858	0.628083	0.8149	0.02824	7,8-Dihydroxykynurete	-17.998256	-	-	-	36.50545	222.169596
POS08481	115607.0741	138496.8683	0.260624	0.659469	0.8327	0.0646	Fleroxacin	11.488898	-	-	-	36.4956	370.34982
POS08482	121504.0239	93793.65091	-0.37344	0.271062	0.5885	0.09414	BL V	-0.0326369	-	-	-	36.4827	425.364263
POS08483	455524.1054	383164.607	-0.24956	0.033494	0.2804	0.21119	L-Oxalylalbizziine	12.956428	-	-	-	36.47825	220.162316
POS08485	509180.5589	322918.9178	-0.65701	0.129632	0.4481	0.28087	N7-Methylguanosine	-19.02143	-	-	-	36.4741	299.276803
POS08486	390238.1311	288432.1369	-0.43612	0.060257	0.3376	0.25298	cis-(Homo)2-aconitate	-22.919218	-	-	-	36.47405	203.164043
POS08488	2810965.45	2447663.466	-0.19966	0.800717	0.9093	0.29493	Pyrido[3,4-c]psoralen	-5.6110891	-	-	-	36.4646	238.216246
POS08490	247896.9115	131541.8239	-0.91422	0.20408	0.5278	0.20832	[1-(7-methoxy-2-oxo-2H-chromen-6-yl)-3-oxobutoxy]sulfonic acid	10.404935	-	-	-	36.45775	343.330838
POS08491	152739.4326	240448.5434	0.654656	0.383573	0.6571	0.15602	1-(2,3,4,6-tetrahydroxyphenyl)-3-(2,3,4-	-6.5463776	-	-	-	36.4577	337.257075
POS08493	68370.67402	56898.64901	-0.26498	0.297998	0.6114	0.06151	6-(alpha-D-Glucosaminyl)-1D-myo-inositol	-1.5479316	-	-	-	36.45125	342.318448
POS08495	528094.4544	336841.8009	-0.64872	0.095723	0.4004	0.34718	Pyrimidine 5'-deoxynucleotide	19.472584	-	-	-	36.4503	278.203674
POS08496	5305854.343	3712608.124	-0.51515	0.025324	0.2562	1.07447	D-Erythroascorbic acid 1'-a-D-xylopyranoside	-15.88048	-	-	-	36.4496	279.215658
POS08500	123969.0106	38912.64631	-1.67167	0.098538	0.4049	0.20822	Pyrazolate	4.5943041	-	-	-	36.44495	440.321595
POS08505	79978.23462	74553.6854	-0.10133	0.821031	0.9179	0.00991	4,4'-Diaminostilbene dihydrochloride	-11.240885	-	-	-	36.4444	284.200293
POS08508	97236.79747	38910.90837	-1.32133	0.012839	0.21	0.20346	Fraxin	-1.8343998	-	-	-	36.4444	371.314697
POS08509	174653.2261	137017.9677	-0.35013	0.573388	0.7814	0.0875	Melicopine	-23.980809	-	-	-	36.44285	314.304463

POS08519	597574.0325	411307.0775	-0.5389	0.345077	0.6341	0.24592	a-Methyldopa mono-O-sulfate	-6.6907271	-	-	-	36.4269	324.325113
POS08521	113215.0164	99186.54044	-0.19085	0.319396	0.6273	0.05775	Diamidafos	13.597868	-	-	-	36.42355	201.184799
POS08524	585706.0725	453102.0111	-0.37034	0.603175	0.7994	0.19303	Acyclovir	19.324695	-	-	-	36.40885	226.216229
POS08526	433906.2813	334137.3116	-0.37694	0.010205	0.1952	0.27761	Calcium carbote	18.774677	-	-	-	36.4083	101.096056
POS08529	473313.8009	463293.6151	-0.03087	0.885304	0.9482	0.00644	Flumetsulam	9.9337243	-	-	-	36.4083	326.304508
POS08531	355247.443	310055.0962	-0.1963	0.320918	0.6286	0.10845	L-Rhamnose	-0.2408674	-	-	-	36.3925	165.163737
POS08532	458449.0303	394610.333	-0.21633	0.294757	0.6086	0.14738	Sulfite	-11.630251	-	-	-	36.3925	83.085422
POS08539	258114.8544	153455.6636	-0.75019	0.522996	0.7529	0.13485	[2-hydroxy-5-(3,5,7-trihydroxy-5H-chromen-2-yl)phenyl]oxidanesulfonic	-19.983218	-	-	-	36.31705	368.299937
POS08542	280826.9565	223613.8829	-0.32867	0.101938	0.4083	0.16933	D-Mannitol 1-phosphate	18.396338	-	-	-	36.3133	263.163799
POS08548	126714.79	82424.32512	-0.62044	0.618279	0.8102	0.07015	Bis(3-azidopyridinium)-1,10-decane perchlorate	-22.345891	-	-	-	36.3009	452.39309
POS08549	396000.3886	303473.6104	-0.38393	0.011014	0.1993	0.26159	N-Acetylgalactosamite	-21.335376	-	-	-	36.2919	236.193559
POS08551	31606.02752	25169.04302	-0.32855	0.782222	0.8996	0.02238	Styraxin	6.1867333	-	-	-	36.28685	371.362268
POS08552	93827.17818	63828.43634	-0.55581	0.110488	0.4223	0.12708	[2-(5,6-dihydroxy-7-methoxy-4-oxo-2-phenyl-4H-chromen-8-yl)-3,5	15.009111	-	-	-	36.28645	497.454728
POS08553	505305.6454	394676.5718	-0.35649	0.059587	0.3364	0.22576	Ketamine hydrochloride	23.888715	-	-	-	36.28165	275.200027
POS08555	108914.2789	114356.8185	0.070349	0.748711	0.8819	0.01291	S-Methyl-3-phospho-1-thio-D-glycerate	8.7502706	-	-	-	36.2673	217.158568
POS08557	1019429.257	865119.9423	-0.23679	0.239072	0.559	0.24822	Dicrotophos	14.655043	-	-	-	36.2617	238.200853
POS08559	485339.8498	230579.8961	-1.07373	0.12707	0.4454	0.33501	D-Glucono-1,5-lactone 6-phosphate	19.284002	-	-	-	36.255	259.132154
POS08566	441511.3757	343315.9585	-0.36291	0.380146	0.6541	0.14677	Bialaphos	-3.692723	-	-	-	36.23425	324.288683
POS08569	158086.7507	93552.70655	-0.75687	0.06881	0.3541	0.19485	Pachyrrhizone	8.7927234	-	-	-	36.2261	367.331498
POS08570	132350.4353	125360.9454	-0.07827	0.814763	0.9158	0.03633	Loquatoside	-20.605845	-	-	-	36.2261	439.380343
POS08574	1215038.933	892500.3257	-0.44508	0.12561	0.4442	0.41337	N-Acetyl-L-aspartate	-19.405843	-	-	-	36.21985	176.143278
POS08577	95313.77457	394285.6165	2.048484	0.358836	0.6422	0.22906	Nicotite D-ribonucleotide	5.264899	-	-	-	36.2184	337.220947
POS08579	13386465.09	201652.4264	-6.05276	0.000669	0.0721	3.32977	3,4-Dihydroxymandelate	1.1209926	-	-	-	36.208	185.153583

POS08584	95025.51727	213063.7062	1.164898	0.197517	0.5233	0.23506	Dihydroferulic acid 4-O-glucuronide	-2.8258941	-	-	-	36.2023	373.330224
POS08587	859095.2862	726941.3868	-0.24098	0.048883	0.3167	0.27669	Parathion-methyl	6.4646706	-	-	-	36.18505	264.216478
POS08588	281537.1569	136351.8839	-1.04599	0.004048	0.137	0.35181	3,3',4',5,6,7,8-Heptahydroxyflavone	0.2501496	-	-	-	36.18505	335.24186
POS08592	257959.2719	182017.0648	-0.50307	0.381164	0.6548	0.14348	3,4,5-trihydroxy-6-{[3-(3,4,5-trihydroxyphenyl)propano	-3.76388	-	-	-	36.1683	375.303868
POS08593	694635.795	690762.784	-0.00807	0.995616	0.9978	0.01749	3,4-Dihydroxyphenylglycol O-sulfate	13.140379	-	-	-	36.1596	251.236565
POS08595	291166.7993	234948.5158	-0.3095	0.250096	0.568	0.13991	Bortezomib	17.082282	-	-	-	36.1533	385.25084
POS08596	530696.6466	412744.9351	-0.36264	0.085768	0.3851	0.25298	Endothion	16.924055	-	-	-	36.15245	281.246719
POS08599	205706.9206	186632.5821	-0.14039	0.438483	0.6955	0.07059	Eflornithine	-8.2225048	-	-	-	36.1412	183.174279
POS08601	235300.8941	249691.9709	0.085643	0.80851	0.9121	0.04001	2-Amino-1,2-bis(p-chlorophenyl)ethanol	-13.33533	-	-	-	36.129	283.168614
POS08602	290575.6401	261084.7857	-0.1544	0.70907	0.8579	0.03593	Nitazoxanide	15.095868	-	-	-	36.1259	308.293915
POS08608	248078.4513	227208.7899	-0.12678	0.726222	0.8685	0.0572	3-(Imidazol-4-yl)-2-oxopropyl phosphate	4.1110796	-	-	-	36.1228	221.127982
POS08610	1309196.959	1098417.249	-0.25326	0.035476	0.2845	0.36443	5'-Dehydroadenosine	-2.37355	-	-	-	36.121	266.232047
POS08611	1768226.448	1633069.034	-0.11472	0.627891	0.8149	0.14422	Formyl phosphate	22.89798	-	-	-	36.1207	127.015462
POS08618	6717657.209	6458561.532	-0.05675	0.808226	0.9121	0.13066	2-Oxo-4-phosphonobutanoate	12.385882	-	-	-	36.1184	183.078032
POS08622	262498.8504	186669.6984	-0.49182	0.675499	0.8398	0.1049	Nitrofurazone	1.5485438	-	-	-	36.1149	199.143783
POS08624	3063526.62	1924544.321	-0.67068	0.089717	0.3895	0.7213	Mitomycin B	17.395178	-	-	-	36.11315	336.325109
POS08628	80473.11357	72331.61309	-0.15388	0.623659	0.8127	0.04174	alpha,beta-Dihydroxyethyl-TPP	7.5634972	-	-	-	36.10945	486.377248
POS08629	547733.5741	286161.882	-0.93664	0.017996	0.2285	0.43918	Musca-aurin-I	-20.220952	-	-	-	36.10605	352.268474
POS08631	119611.494	76300.48889	-0.64859	0.09932	0.4055	0.16308	Sterigmatocystin	1.7618409	-	-	-	36.1042	325.292148
POS08632	230187.0611	122711.5947	-0.90754	0.024279	0.2519	0.2755	{4-[(2,4-dihydroxy-3-oxo-6-{[3,4,5-trihydroxy-6-(hydroxymethyl)oxan-2	4.231617	-	-	-	36.1007	531.459521
POS08643	73638.80039	67912.48634	-0.11679	0.717591	0.8635	0.02747	3-oxobrimonidine	-17.378539	-	-	-	36.0643	311.151887
POS08647	200966.302	621805.0652	1.629509	0.035404	0.2845	0.5576	Nifurthiazole	6.0135055	-	-	-	36.0535	255.231405
POS08648	213979.9841	76105.66175	-1.4914	0.007278	0.1779	0.34349	Jacareubin	1.0719244	-	-	-	36.0512	327.307826

POS08653	1851210.481	980906.5304	-0.91628	0.032453	0.2773	0.72779	2-hydroxy-3-[4-(sulfooxy)phenyl]propanoi c acid	-3.0136942	-	-	-	36.0347	263.236486
POS08658	569116.778	447962.3329	-0.34535	0.036136	0.2856	0.29431	5,6-Dihydrouracil	16.324802	-	-	-	35.9862	115.111739
POS08659	244134.2638	259556.4614	0.088374	0.824282	0.9196	0.05506	p-Nitrophenyl-O-ethyl ethylphosphote	-8.6125665	-	-	-	35.9858	260.200644
POS08663	664431.7477	591979.2638	-0.16657	0.870702	0.9423	0.00074	N-Acetylneuramite	-13.069084	-	-	-	35.9767	310.273135
POS08672	3750771.438	2956405.957	-0.34334	0.020362	0.2383	0.75008	2-hydroxy-3-(sulfooxy)benzoic acid	11.788424	-	-	-	35.9377	235.190037
POS08674	535227.1554	453216.4894	-0.23995	0.375984	0.651	0.13183	Acetyl citrate	7.8375072	-	-	-	35.9353	233.153396
POS08676	294843.5089	206090.9177	-0.51667	0.26091	0.5788	0.16558	1-Deoxy-D-altro-heptulose 7-phosphate	-21.129994	-	-	-	35.9239	275.163884
POS08679	242074.7533	195457.2637	-0.3086	0.533221	0.7588	0.06575	(5,6,7-trihydroxy-4-oxo-2-phenyl-3,4-dihydro-2H-1-benzopyran-3	0.5833229	-	-	-	35.9182	369.317491
POS08681	1267859.983	1047408.351	-0.27557	0.050308	0.3192	0.35764	Mono-(3-carboxypropyl) phthalate	-22.908705	-	-	-	35.90065	252.216522
POS08684	105750.176	290030.2247	1.455543	0.027251	0.2602	0.37107	2-Methylaminoadenosine	-1.2485198	-	-	-	35.89455	297.289407
POS08688	156725.8797	122164.2277	-0.35942	0.106675	0.4153	0.14766	Bortezomib	-20.610788	-	-	-	35.8904	385.236357
POS08689	352535.7651	302594.897	-0.22038	0.287042	0.6024	0.11721	Thiamine(1+) Diphosphate(1-)	-23.063726	-	-	-	35.8904	425.303491
POS08691	88412.47443	129231.2384	0.547633	0.406688	0.6724	0.10744	Coenzyme F420-1	-24.57518	-	-	-	35.8904	645.470038
POS08699	269370.1165	206320.1088	-0.38471	0.565971	0.7776	0.12823	Diallat	-1.8429133	-	-	-	35.8644	271.225879
POS08701	850503.8929	739020.0603	-0.2027	0.088437	0.3878	0.24703	Lycomarasmine B	-23.893973	-	-	-	35.86215	278.231952
POS08704	132767.1034	184467.8988	0.474472	0.408077	0.6731	0.13528	Geranyl diphosphate	-7.217288	-	-	-	35.8437	315.214109
POS08709	97195.4909	84142.76839	-0.20805	0.519188	0.75	0.04879	Hydroflumethiazide	-15.471298	-	-	-	35.84075	332.294151
POS08710	328819.1142	287272.4339	-0.19487	0.498561	0.7356	0.08069	fluvoxamino acid	-1.0663004	-	-	-	35.835	319.298537
POS08712	109038.4085	83483.3993	-0.38528	0.293521	0.6081	0.09052	(4-{6,11-dihydroxy-7-[3-hydroxy-5-methoxy-4-(sulfooxy)phenyl]-2,8	6.6180906	-	-	-	35.8303	514.460675
POS08714	266046.2757	308564.1843	0.213893	0.383722	0.6571	0.0844	2,5-Furandicarboxylate	-8.8959386	-	-	-	35.8226	157.098888
POS08715	1020797.067	797614.3916	-0.35593	0.672981	0.8388	0.19299	N-Trimethyl-2-aminoethylphosphote	0.5921998	-	-	-	35.81755	169.158676
POS08722	697651.529	803550.0043	0.203881	0.767834	0.8921	0.13288	Aurothioglucose	5.9762215	-	-	-	35.8045	393.18972
POS08729	945827.6346	434457.6001	-1.12236	0.032061	0.2761	0.57324	N-Acetoxy-IQ	-3.8241926	-	-	-	35.7911	257.266297

POS08730	39315.74791	12877.69871	-1.61023	0.032763	0.2781	0.13018	Formothion	-19.152867	-	-	-	35.7911	258.269849
POS08733	171728.519	130166.5337	-0.39977	0.449657	0.7039	0.12863	Aripiprazole	9.9041669	-	-	-	35.79085	449.397117
POS08736	1109946.7	849311.9408	-0.38612	0.110232	0.422	0.3714	Pterin	-4.3070218	-	-	-	35.7883	164.143374
POS08743	264094.9798	235569.8237	-0.1649	0.470243	0.7182	0.07302	4-Hydroxy-2,2',3,4',5,5',6-heptachlorobiphenyl	-4.4448099	-	-	-	35.785	412.328048
POS08746	462987.8452	385341.4856	-0.26484	0.055435	0.3276	0.20618	Atoxin a(s)	0.4745845	-	-	-	35.77885	253.215396
POS08749	302680.1016	258338.4356	-0.22853	0.729102	0.8706	0.05281	6-Thioguanosine monophosphate	16.600713	-	-	-	35.7722	380.299773
POS08750	161241.2026	105034.9993	-0.61835	0.172035	0.4983	0.16241	Ethoxysulfuron	-11.829585	-	-	-	35.7722	399.393564
POS08753	560163.5538	461687.7834	-0.27893	0.050793	0.3197	0.25277	UDP-N-acetyl-D-galactosamine 4-sulfate	-8.4270499	-	-	-	35.77185	688.418384
POS08757	545343.9579	324215.6704	-0.75021	0.026055	0.2576	0.37938	Sulfoquinovose	-4.1443639	-	-	-	35.7566	245.225964
POS08762	489381.6626	380434.6915	-0.36331	0.053905	0.3242	0.24553	3-Deoxy-D-manno-octulosote	3.3680496	-	-	-	35.7364	239.200079
POS08765	215780.0655	156515.139	-0.46326	0.390262	0.6604	0.10367	Mannopine	16.099064	-	-	-	35.7344	311.313272
POS08768	443094.8742	425962.4032	-0.05689	0.826647	0.9204	0.00203	Fosfomycin calcium	-6.6305994	-	-	-	35.7311	177.127309
POS08769	1046659.777	508177.2244	-1.04239	0.066264	0.3498	0.54375	(5,7-dihydroxy-4-oxo-2-phenyl-3,4-dihydro-2H-1-benzopyran-3	17.411158	-	-	-	35.7306	353.323411
POS08772	7192267.524	6299826.918	-0.19113	0.229326	0.5504	0.58507	Calcium L-aspartate	-15.115399	-	-	-	35.7278	172.169489
POS08774	514952.0872	296956.5822	-0.79419	0.109013	0.4192	0.34063	2,3-Dioxo-6-nitro-7-sulfamoylbenzo[f]quinoxaline	-9.5291238	-	-	-	35.7248	337.284172
POS08775	927300.6678	564705.4255	-0.71554	0.282709	0.5986	0.433	5-Acetylamino-6-amino-3-methyluracil	9.2705289	-	-	-	35.7248	199.188414
POS08778	89208.25666	50689.26178	-0.8155	0.100978	0.4074	0.15382	O-Carbamoyladenylate	-15.520848	-	-	-	35.71475	391.24722
POS08779	5031056.816	2503034.272	-1.00718	0.055831	0.3276	1.19491	Pretetramid	8.9569823	-	-	-	35.71325	352.320023
POS08783	443355.602	384246.6568	-0.20643	0.388718	0.6595	0.11215	Nifuradene	16.330031	-	-	-	35.68655	225.184437
POS08784	139542.3521	128943.3283	-0.11397	0.634995	0.8188	0.02522	Gallagic acid	-18.254712	-	-	-	35.6764	605.381544
POS08786	215852.2738	181441.4695	-0.25054	0.379326	0.6535	0.11811	4-(L-Alanin-3-yl)-2-hydroxy-cis,cis-mucote 6-semialdehyde	-16.952678	-	-	-	35.6666	230.190091
POS08790	118458.1754	103240.5974	-0.19837	0.487066	0.7291	0.0508	Sanguisorbic acid dilactone	11.247725	-	-	-	35.64895	471.308866
POS08795	10728350.81	3230154.082	-1.73175	0.158519	0.4869	1.89735	Avenanthramide C	-2.8613323	-	-	-	35.62635	316.283874

POS08797	1932319.946	2070654.477	0.099753	0.893553	0.9511	0.03233	Riluzole	-0.8105355	-	-	-	35.61095	235.205387
POS08802	155341.5294	89730.40673	-0.79177	0.016424	0.2247	0.20712	2-epi-5-epi-Valiolone 7-phosphate	-22.614969	-	-	-	35.59475	273.147622
POS08807	412257.6575	298727.1375	-0.46472	0.2538	0.572	0.19706	1-epi-Valienol 7-phosphate	-22.045488	-	-	-	35.5777	257.14873
POS08808	1619218.512	234996.3207	-2.78459	0.342189	0.6327	0.62754	5-(methylsulfanyl)-2,3-dioxopentyl Phosphate(2-)	8.9465317	-	-	-	35.5739	241.179425
POS08812	570169.0235	448394.555	-0.34662	0.264618	0.581	0.20792	Ellagic acid	-16.309384	-	-	-	35.5633	303.194948
POS08815	216811.3522	215527.5108	-0.00857	0.974319	0.9877	0.01457	Ethidium bromide	-13.600091	-	-	-	35.56285	395.309514
POS08816	3634.79813	1	-11.8277	0.114589	0.4284	0.04216	Candesartan N2-glucuronide	16.462992	-	-	-	35.5622	617.595527
POS08822	193746.1264	287484.2439	0.569315	0.309901	0.6211	0.16122	Aspartyl-Glutamate	0.6416098	-	-	-	35.5424	262.216144
POS08824	48648.60714	39800.95285	-0.2896	0.275868	0.5926	0.05437	Diosbulbinoside D	-22.64784	-	-	-	35.5424	507.494905
POS08825	1465241.67	687218.2263	-1.0923	0.043166	0.3057	0.67635	Sodium caffeine benzoate	-3.8646089	-	-	-	35.5412	339.299769
POS08827	278196.2441	186889.2443	-0.57392	0.106517	0.415	0.23602	2-formamido-N(1)-(5-O-phosphonato-D-ribosyl)acetamidine	-13.623549	-	-	-	35.52585	313.198023
POS08828	186693.5869	90928.61035	-1.03787	0.421863	0.6829	0.16517	Lulicozole	-2.5846158	-	-	-	35.52585	355.283761
POS08832	138764.6135	130631.1688	-0.08714	0.821428	0.9182	0.00799	Methyclothiazide	-23.041674	-	-	-	35.52255	361.236276
POS08835	415887.062	371519.0303	-0.16276	0.434306	0.6923	0.09697	Gravacridonetriol glucoside	4.0605732	-	-	-	35.51475	520.507286
POS08836	259540.0814	239012.0109	-0.11887	0.542689	0.7639	0.05702	Rheidin C	23.727546	-	-	-	35.51475	539.521054
POS08837	376993.1889	261668.2575	-0.5268	0.037509	0.2904	0.27643	Cycloguanil hydrochloride	-15.07166	-	-	-	35.5093	289.179233
POS08839	584236.1295	490500.606	-0.2523	0.157967	0.4864	0.17826	TEPP	-0.4857149	-	-	-	35.5093	291.194836
POS08840	134472.6935	203699.5633	0.59913	0.587917	0.7904	0.06758	Diclofop	-13.718574	-	-	-	35.5093	328.162188
POS08841	591926.8524	532979.3071	-0.15134	0.266259	0.5827	0.13577	Cefamandole fate	7.0072121	-	-	-	35.49945	513.505468
POS08842	2492091.333	1902199.184	-0.38969	0.189185	0.5161	0.46233	Boc-Asn-OPhNO2	3.1177129	-	-	-	35.49695	354.335678
POS08844	444999.4102	344988.3876	-0.36726	0.148164	0.4736	0.22936	4-Hydroxylamino-2,6-dinitrotoluene	18.463572	-	-	-	35.49695	214.158812
POS08845	199776.4483	120247.2816	-0.73238	0.22082	0.5419	0.16192	Sulfazecin	-5.468682	-	-	-	35.49695	397.378709
POS08846	824795.9321	748769.491	-0.13952	0.38917	0.6599	0.13691	3-Demethylsimmondsin 2'-(Z)-ferulate	-4.8174081	-	-	-	35.49695	538.517787

POS08852	47040.94824	29689.23285	-0.66398	0.51673	0.7483	0.05483	dTDP-3-N,N-dimethylamino-2,3,6-trideoxy-4-keto-D-glucose	-21.498301	-	-	-	35.4799	558.377994
POS08855	1748949.099	1470892.079	-0.2498	0.053987	0.3242	0.39226	5,6-Dihydro-5-fluorouracil	6.3705135	-	-	-	35.47225	133.101218
POS08856	309509.0055	283959.4287	-0.1243	0.280822	0.5966	0.08125	Cyometrinil	-22.851606	-	-	-	35.47225	186.185145
POS08859	397952.3524	231224.5192	-0.7833	0.024549	0.2524	0.33276	2-(2,4-dihydroxy-5-methoxyphenyl)-3,5,7-trihydroxy-3,4-dihydro	18.732729	-	-	-	35.47165	335.293539
POS08864	286781.955	291849.2924	0.025269	0.983334	0.9918	0.01587	IACI	2.8136733	-	-	-	35.4599	599.44436
POS08865	197300.5012	357350.0915	0.856944	0.327423	0.632	0.2365	2-(3,5-Dichlorophenylcarbamoyl)-1,2	-7.3694116	-	-	-	35.4599	303.15825
POS08867	67767.56695	56911.8981	-0.25186	0.291142	0.6054	0.05661	Carminomycin	15.760525	-	-	-	35.4599	514.50867
POS08872	48364024.33	20797460.76	-1.21753	0.039867	0.2968	4.0679	1-Methyladenosine	12.255506	-	-	-	35.4574	282.278624
POS08873	640551.1023	274581.1014	-1.22208	0.057627	0.3326	0.45352	(E)-Avenanthramide D	-2.134878	-	-	-	35.4574	284.285372
POS08874	444257.8015	345452.6745	-0.36291	0.291763	0.6058	0.16295	Flumioxazin	0.841678	-	-	-	35.4574	355.339375
POS08880	386459.914	3867072.315	3.322851	0.352224	0.6364	1.1284	4-nitrophenolate	12.782926	-	-	-	35.4549	139.112042
POS08882	250097.1095	767400.301	1.617491	0.406767	0.6724	0.27528	2,8-Dihydroxyquinoline-beta-D-glucuronide	0.694521	-	-	-	35.4549	338.289011
POS08884	33390.01151	17618.89883	-0.92229	0.053755	0.3239	0.09085	3-O-Methylquercetin	-5.605128	-	-	-	35.4549	317.267804
POS08885	266333.3829	192977.0365	-0.4648	0.394691	0.6637	0.12619	N,6-O-Disulfo-D-glucosamine	-4.9081808	-	-	-	35.4549	340.303111
POS08886	152412.5998	86643.19515	-0.81482	0.047427	0.3143	0.20159	Molybdopterin precursor Z	1.6977368	-	-	-	35.4549	345.205261
POS08887	2604682.945	2256530.722	-0.207	0.104303	0.4115	0.41582	Myxochelin C	-14.087884	-	-	-	35.4543	540.533576
POS08888	223505.609	223768.5847	0.001696	0.995494	0.9977	0.01214	Sodium methallylsulfote	-9.0491666	-	-	-	35.4543	159.157145
POS08893	112956.72	91075.30597	-0.31064	0.422006	0.6829	0.09776	3-[3,5-dihydroxy-4-(sulfooxy)benzoyloxy]-4,5-dihydroxybenzoic acid	-10.400182	-	-	-	35.4511	403.283093
POS08899	205608.071	204681.2873	-0.00652	0.978738	0.9898	0.02499	Endothal-disodium	20.671935	-	-	-	35.44665	231.137734
POS08906	51937.67647	45544.3964	-0.18951	0.434957	0.6931	0.03382	Flavonol 3-O-[alpha-L-rhamnosyl-(1->6)-beta-D-glucoside]	-4.2669899	-	-	-	35.4396	547.524845
POS08908	27115.61597	45983.73254	0.762	0.132063	0.4516	0.10733	Americanin B	-4.6631647	-	-	-	35.43675	493.47908
POS08912	1578969.32	996573.3619	-0.66394	0.171679	0.4979	0.5027	Pyrimidine 5'-nucleotide	-23.840859	-	-	-	35.4339	294.190687
POS08914	75779.5607	69135.41191	-0.13238	0.710791	0.8589	0.01931	Lycoricidine	-1.5118246	-	-	-	35.4339	292.262936

POS08918	204359.0951	181085.8882	-0.17443	0.455172	0.7083	0.07613	L-Ascorbate 6-phosphate	-4.3391204	-	-	-	35.41845	257.110165
POS08919	903188.1112	816897.1126	-0.14487	0.508992	0.7444	0.12964	3-Sulfocatechol	-9.3689279	-	-	-	35.41845	191.179295
POS08922	160255.6363	142550.179	-0.16891	0.521785	0.7515	0.06506	5-Hydroxyisourate	0.8049117	-	-	-	35.4159	185.117125
POS08923	195291.8185	91800.45303	-1.08906	0.000516	0.0721	0.31207	2-Methyl-1,4-naphthalenediol bis(dihydrogen phosphate)	8.4672653	-	-	-	35.41395	335.165806
POS08926	351916.1107	247202.1083	-0.50954	0.225459	0.5471	0.22154	(4-ethenyl-2-hydroxy-6-methoxyphenyl)oxidanesulfonic acid	17.832597	-	-	-	35.403	247.241668
POS08927	75007.32784	56971.93924	-0.39678	0.315084	0.6245	0.08339	Daunorubicin	-5.4506058	-	-	-	35.403	528.524301
POS08929	244472.1661	292763.5065	0.260066	0.706968	0.8568	0.0872	D-Fructose 6-phosphate	19.308733	-	-	-	35.3998	261.148099
POS08943	935774.1484	642998.2328	-0.54135	0.015469	0.2226	0.45578	4-Hydroxy-4-methylglutamate	-19.91581	-	-	-	35.3547	178.158948
POS08944	663845.0229	523952.3885	-0.34141	0.287885	0.603	0.18677	dTDP-3-amino-2,3,6-trideoxy-4-keto-D-glucose	17.926872	-	-	-	35.3547	530.346266
POS08946	2504971.987	1291749.895	-0.95547	0.039062	0.2955	0.86679	Glutaminylhistidine	-3.6264974	-	-	-	35.33645	284.294249
POS08947	446109.5155	363061.6765	-0.29718	0.109461	0.4204	0.20159	(Z)-Resveratrol 3,4'-diglucoside	9.0135601	-	-	-	35.33645	553.536757
POS08949	193206.7386	186795.8488	-0.04868	0.92773	0.9672	0.02629	Dianthramine	-0.5677252	-	-	-	35.3182	290.247312
POS08951	64269.86095	45520.98974	-0.49761	0.078371	0.3727	0.09905	Pyranocyanin B	9.719127	-	-	-	35.3162	488.444814
POS08953	15803116.43	12903069.19	-0.29249	0.022724	0.2473	1.38042	Pyrazimide	1.5106098	-	-	-	35.31315	124.120163
POS08954	150924.2716	97190.1104	-0.63494	0.019923	0.2381	0.19375	DHAP(10:0)	18.014018	-	-	-	35.31315	325.320319
POS08955	1432698.183	748190.9871	-0.93726	0.096521	0.4017	0.58643	c-DNP-Cys	19.147796	-	-	-	35.31285	330.299482
POS08957	504745.1161	431341.3729	-0.22672	0.124069	0.442	0.18039	Pinocembrin 7-rhamnosylglucoside	-11.525513	-	-	-	35.29635	565.53597
POS08958	412643.5033	202943.1301	-1.02382	0.058728	0.3352	0.3454	6-Thioinosinic acid	-5.9300292	-	-	-	35.2909	285.297591
POS08959	123602.3896	86395.07899	-0.51669	0.208787	0.5323	0.11519	Hydroxypyruvate	9.4265525	-	-	-	35.2799	105.069758
POS08960	197940.6872	843983.8512	2.092147	0.394446	0.6634	0.31786	3',5'-Cyclic AMP	-23.820926	-	-	-	35.2738	330.205335
POS08962	861103.507	462206.1448	-0.89765	0.316344	0.6253	0.39796	4-Hydroxy-3-nitrosobenzamide	9.9310351	-	-	-	35.2636	167.143026
POS08963	151563.7377	114589.0138	-0.40346	0.088214	0.3878	0.14602	D-Lombricine	1.3288911	-	-	-	35.2636	271.187936
POS08965	1718213.676	1578531.03	-0.12233	0.83102	0.9228	0.03806	Fraxetin	-9.4826296	-	-	-	35.25915	209.172803

POS08967	140357.3725	81818.23546	-0.77861	0.01599	0.2226	0.19512	Furaneol 4-glucoside	-21.096063	-	-	-	35.2557	291.267653
POS08968	223800.8579	201584.6035	-0.15083	0.553876	0.7709	0.05879	5-hydroxy-7-methoxy-2-phenyl-6,8-bis[3,4,5-trihydroxy-6	17.993266	-	-	-	35.2508	593.567939
POS08980	1329866.146	1124085.124	-0.24253	0.184788	0.5109	0.29657	Lipoyl-GMP	-8.8811817	-	-	-	35.2377	552.533378
POS08984	11213.39361	9132.79579	-0.29609	0.833408	0.9238	0.00724	6-[1-(3,4-dimethoxyphenyl)-2-{[3-(3,4-dimethoxyphenyl)-1	-17.836258	-	-	-	35.2374	564.553225
POS08991	458480.2847	372340.488	-0.30024	0.107438	0.4169	0.2104	3,4,5-trihydroxy-6-{[3-hydroxy-10-(3-hydroxybutanoyl)-2,2	-19.288051	-	-	-	35.2129	567.552349
POS08993	50188.19017	48791.30579	-0.04072	0.89997	0.9537	0.00308	Neoacrimarine J	1.7456355	-	-	-	35.1952	518.49168
POS08996	625069.3688	504739.6547	-0.30848	0.05993	0.3373	0.25456	Osthenol-7-O-beta-D-gentiobioside	8.44367	-	-	-	35.1884	555.552359
POS08999	391463.3194	292062.6838	-0.4226	0.433154	0.6917	0.19271	Se-Methylselenomethionine	-23.298831	-	-	-	35.1842	212.143257
POS09006	418544.5722	266332.8954	-0.65215	0.575424	0.783	0.1412	Lecanoric acid	-6.7449527	-	-	-	35.18	319.28333
POS09007	227194.9163	773518.2669	1.767505	0.393282	0.6624	0.29526	(3E)-4-(2-Carboxyphenyl)-2-oxobut-3-enoate	19.265411	-	-	-	35.18	221.189718
POS09008	865292.6547	949491.8821	0.133968	0.743447	0.8788	0.11191	Cyclic di-3',5'-adenylate	14.280265	-	-	-	35.17755	659.428579
POS09012	208910.8836	118717.7463	-0.81535	0.042039	0.3013	0.23194	Leucodelphinidin	-16.785798	-	-	-	35.1668	323.268767
POS09016	546898.4301	335428.9117	-0.70527	0.070658	0.3578	0.36019	5'-Phosphoribosyl-N-formylglycimide	2.6663009	-	-	-	35.1626	315.194614
POS09022	29235.39776	19536.14178	-0.58157	0.267915	0.5849	0.05776	Isoglobotriaose	-16.033047	-	-	-	35.154	503.463521
POS09024	651337.9585	603431.9712	-0.11022	0.588053	0.7904	0.05457	2-Maleylacetate	4.596041	-	-	-	35.1452	159.116803
POS09026	160599.5871	134411.9131	-0.25681	0.369796	0.6489	0.08449	Baicalein 5,6,7-trimethyl ether	15.082282	-	-	-	35.1452	313.328587
POS09027	551550.3999	316200.7953	-0.80265	0.480231	0.7237	0.257	Dopachrome o-semiquinone	14.817151	-	-	-	35.1452	195.174254
POS09028	114362.5266	140478.5543	0.296736	0.168433	0.4948	0.10697	1-(5-Phosphoribosyl)-4-(N-succinocarboxamide)-5-aminoimidazole	8.8465899	-	-	-	35.1452	410.299997
POS09029	70280.69378	91115.11828	0.374562	0.305638	0.6178	0.09491	3-[3,5-dihydroxy-4-(sulfooxy)benzoyloxy]-2,4,5-trihydroxybenzoic	-22.824344	-	-	-	35.1452	419.27773
POS09030	1531347.769	1191264.371	-0.36231	0.046867	0.314	0.4405	2-amino-4-({1-[(carboxymethyl)-C-hydroxycarbonimidoyl]-2	-22.400862	-	-	-	35.1376	568.564563
POS09034	191990.1177	525962.204	1.453927	0.281336	0.5968	0.28152	Dianthalexin	-6.480773	-	-	-	35.1376	240.231926
POS09037	573704.879	447553.8019	-0.35825	0.046249	0.3134	0.27044	Citrusin B	-10.853349	-	-	-	35.1376	569.568006
POS09038	172055.3341	116589.7933	-0.56143	0.206284	0.5307	0.1618	Lorazepam	-24.156504	-	-	-	35.13625	322.157519

POS09039	199480.5296	116237.8426	-0.77917	0.18262	0.5082	0.19299	3,4-Dihydroxyphthalate	-21.957477	-	-	-	35.13405	199.132526
POS09043	83545.90352	39855.0306	-1.06781	0.049256	0.3185	0.15309	Gonyautoxin 5	-15.459706	-	-	-	35.13	380.351112
POS09045	104125.9904	94763.57858	-0.13593	0.709032	0.8579	0.02381	4-Deoxy-beta-D-gluc-4-enuronosyl-(1,3)-N-acetyl-D-galactosamine	-23.830862	-	-	-	35.1296	380.314837
POS09050	225098.3617	136260.863	-0.72418	0.070489	0.3575	0.22008	3,4,5-trihydroxy-6-(3-{3,5,7-trihydroxy-6-[3,4,5-trihydroxy-6	20.521781	-	-	-	35.1282	613.556847
POS09052	124461.2526	95471.47452	-0.38256	0.17373	0.5	0.12524	Etobenzanid	5.2296142	-	-	-	35.1273	341.210256
POS09053	252447.1937	225049.3126	-0.16574	0.378575	0.6529	0.08666	N(beta)-Epoxysuccimoyl-DAP	16.48049	-	-	-	35.12645	218.190156
POS09058	47792.81386	45802.65194	-0.06136	0.855291	0.9345	0.00445	Alkaloid RC	-15.751363	-	-	-	35.12555	532.507505
POS09063	714318.6202	239438.7522	-1.57691	0.06118	0.3403	0.54364	Voricozole	22.010417	-	-	-	35.12125	350.325465
POS09064	736038.1763	723274.0654	-0.02524	0.9221	0.9648	0.02144	N-[4'-hydroxy-(E)-cinnamoyl]-L-aspartic acid	-17.71143	-	-	-	35.1196	280.247731
POS09068	145997.5942	138691.7307	-0.07406	0.845209	0.9297	0.03319	Reduced 2,6-Dichlorophenolindophenol	-0.166867	-	-	-	35.1196	271.118632
POS09070	272957.486	107981.5827	-1.33789	0.001761	0.0956	0.37296	3,4,5-trihydroxy-6-(1-oxo-1H-isochromene-3-carbonyloxy)oxane-2	-4.7336646	-	-	-	35.1196	367.283543
POS09075	259242.6321	259201.4317	-0.00023	0.999014	0.9997	0.00526	6-({2-[(acetyloxy)methyl]-3,5,6-trihydroxyoxan-4-yl}oxy)-3,4,5	1.3247448	-	-	-	35.11145	399.324804
POS09077	460953.3857	481094.456	0.061699	0.95212	0.9791	0.02456	Potassium dichromate	-4.4765351	-	-	-	35.1095	295.19056
POS09078	151628.684	80153.21117	-0.91971	0.007203	0.1779	0.22583	N-benzoylglycinate	22.806181	-	-	-	35.1095	179.17934
POS09080	228570.0997	145691.5509	-0.64972	0.45483	0.7083	0.14158	3-Epihydroxymugineic acid	21.272067	-	-	-	35.1077	337.30963
POS09081	101139.6647	102261.7223	0.015917	0.966224	0.9843	0.01632	Cefuroxime axetil	15.343765	-	-	-	35.1077	511.489609
POS09083	66402.48334	1126776.406	4.08482	0.337396	0.6326	0.452	2-Deoxy-D-ribose 1,5-bisphosphate	13.234813	-	-	-	35.1059	295.101469
POS09090	232118.8946	206324.0341	-0.16995	0.613988	0.8079	0.06748	Chlorhexidine acetate	19.191702	-	-	-	35.1	626.569782
POS09103	205582.6882	189780.2178	-0.11539	0.788678	0.9028	0.02716	alpha,alpha'-Trehalose 6-phosphate	10.721026	-	-	-	35.0562	423.288204
POS09108	84327.34167	83376.83174	-0.01635	0.943591	0.9747	0.00414	2-Hydroxy-6-oxono-2,4-diene-1,9-dioate	0.6901933	-	-	-	35.053	215.179524
POS09110	138778.3593	52226.28782	-1.40993	0.005114	0.1518	0.26645	Arborinine	-2.7702814	-	-	-	35.0524	286.301086
POS09111	156652.6422	121898.1909	-0.36189	0.155853	0.4841	0.12737	2-Bromo-5-hydroxy-N-[2-(4-hydroxyphenyl)ethyl]-4-methoxy-N	3.0198202	-	-	-	35.0524	381.241525
POS09113	115454.7223	93755.25796	-0.30036	0.257095	0.5751	0.08928	2-amino-4-({1-[(carboxymethyl)-C-hydroxycarbonimidoyl]-2	-15.772415	-	-	-	35.03615	560.538451

POS09115	175619.904	143635.1686	-0.29005	0.304414	0.6169	0.10727	N-debutylhalofantrine	12.836564	-	-	-	35.03395	445.32998
POS09119	452415.4032	379329.644	-0.2542	0.218994	0.54	0.15222	2-amino-4-({1-[(carboxymethyl)-C-hydroxycarbonimidoyl]-2	21.308865	-	-	-	35.02535	594.579925
POS09124	388206.6529	365893.921	-0.0854	0.73462	0.8742	0.05149	1-Methylseleno-N-acetyl-D-galactosamine	-7.4097129	-	-	-	35.00615	299.200067
POS09126	217343.9858	199613.0693	-0.12277	0.725795	0.8684	0.07419	D-Erythritol 4-phosphate	-2.8069464	-	-	-	35.0022	203.106409
POS09128	68874.64434	60081.19492	-0.19706	0.577518	0.7844	0.02793	Metsulfuron-methyl	-10.605865	-	-	-	34.9983	382.367032
POS09129	223031.9862	185336.6669	-0.2671	0.390304	0.6604	0.08378	Primisulfuron	-7.3792332	-	-	-	34.9983	455.314324
POS09130	351563.7261	247938.3153	-0.5038	0.025883	0.2573	0.25942	Flavonol 3-O-beta-D-glucosyl-(1->2)-beta-D-glucoside	-13.049018	-	-	-	34.9983	563.519236
POS09131	165228.279	136254.584	-0.27816	0.427066	0.6863	0.0726	Clitocine	-16.778258	-	-	-	34.99545	288.231857
POS09133	87034.16728	53679.86512	-0.6972	0.093118	0.3958	0.13821	5-Amino-6-(5'-phosphoribosylamino)uracil	22.524278	-	-	-	34.9921	355.225855
POS09134	634937.4663	569447.1058	-0.15705	0.336597	0.6326	0.11382	5-FU	8.3015626	-	-	-	34.9921	131.085556
POS09135	121300.6532	60553.87325	-1.0023	0.073411	0.3635	0.17845	2-{[hydroxy({3,4,8,9,10-pentahydroxy-6-oxo-6H-benzo[c]chromen-1	-12.511555	-	-	-	34.9921	378.263556
POS09139	281988.547	273381.2265	-0.04472	0.834549	0.9243	0.00685	3,4,5-trihydroxy-6-[(2,3,5-trihydroxy-6-{[(3-methoxy-3	12.830449	-	-	-	34.95475	457.366132
POS09140	43774.28067	44638.84666	0.028216	0.95438	0.98	0.00651	UDP-3-ketoglucose	-16.179668	-	-	-	34.9329	565.284047
POS09142	717119.9044	577189.9264	-0.31317	0.099368	0.4055	0.25852	Streptomycin	-2.4220384	-	-	-	34.9226	582.579968
POS09143	265449.7566	145262.78	-0.86977	0.018349	0.2285	0.30735	CGP 28-392	0.7845981	-	-	-	34.9187	366.335663
POS09144	194996.1192	172649.9339	-0.1756	0.571205	0.7803	0.06205	3,4,5-trihydroxy-6-{[3-(6-hydroxy-7-methoxy-2H-1,3-benzodioxol-5	-13.820064	-	-	-	34.9123	415.320551
POS09145	419776.1129	436846.2077	0.057505	0.928524	0.9674	0.02127	3-oxo-3-[(3,4,5,6-tetrahydroxyoxan-2-yl)methoxy]propanoic	-12.034324	-	-	-	34.9123	267.206073
POS09146	140887.1635	139447.0115	-0.01482	0.957926	0.981	0.00405	2-O-Galloylgalactaric acid	4.3348076	-	-	-	34.9123	363.251947
POS09150	141615.1462	127327.7439	-0.15343	0.591426	0.7918	0.04001	Kuwanon Z	20.061582	-	-	-	34.9078	595.583404
POS09152	1092726.36	88740.23756	-3.6222	0.184387	0.5108	0.66127	(+)-Luguine	-23.129278	-	-	-	34.9033	336.309721
POS09154	220591.3904	185510.1884	-0.24988	0.376345	0.6512	0.09449	UK-47265	11.395053	-	-	-	34.9033	340.191142
POS09155	973537.3169	715235.5064	-0.44482	0.035478	0.2845	0.38572	Lichenin	1.034007	-	-	-	34.9033	163.148044
POS09161	107170.8923	44653.10447	-1.26308	0.022408	0.2466	0.21763	hesperetin 3'-O-sulfate	3.812064	-	-	-	34.8891	383.350734

POS09162	315740.4739	257253.1357	-0.29555	0.0904	0.3896	0.16639	Orotidine	-9.1916864	-	-	-	34.88835	289.215527
POS09165	107229.5581	122543.1172	0.192587	0.518702	0.7497	0.05119	1,2,3,4,6,7,8-Heptachlorodibenzofuran	-4.130157	-	-	-	34.8743	410.312286
POS09170	2486735.622	1928421.459	-0.36683	0.11082	0.4228	0.53265	Isosorbide mononitrate	0.3994756	-	-	-	34.8546	192.146153
POS09173	155032.6437	115625.1784	-0.42312	0.292595	0.6073	0.10809	Propicozole	-5.2021517	-	-	-	34.84455	343.225896
POS09175	713645.24	614314.4692	-0.21623	0.335825	0.6326	0.17118	1D-1-Guanidino-3-amino-1,3-dideoxy-scyllo-inositol 4-phosphate	6.6021501	-	-	-	34.8438	301.215459
POS09176	177084.5117	103800.2433	-0.77063	0.143323	0.4679	0.17389	[2,6-dihydroxy-3-(7-hydroxy-4-oxo-4H-chromen-2	-14.88231	-	-	-	34.8438	367.301825
POS09177	144236.3257	77835.57941	-0.88993	0.065127	0.3462	0.1807	Molybdopterin	16.798092	-	-	-	34.8438	396.365718
POS09180	223927.2777	171890.1721	-0.38154	0.022066	0.2454	0.17489	O-Carbamoyladenylate	22.813685	-	-	-	34.84185	391.26218
POS09181	354521.6044	205277.3509	-0.7883	0.078869	0.3736	0.25962	5-Hydroxytryptophol glucuronide	-0.5208792	-	-	-	34.84185	340.3475
POS09182	168770.2164	134766.8124	-0.3246	0.474399	0.7206	0.09977	5-(2'-Carboxyethyl)-4,6-dihydroxypicolite	-16.207134	-	-	-	34.8399	228.174495
POS09185	198573.7508	142120.0692	-0.48256	0.129819	0.4485	0.16603	2-(2,5-dihydroxy-4-methoxyphenyl)-3,5,6,7-tetrahydroxy-4H-chromen	6.7353356	-	-	-	34.83835	349.272622
POS09186	3876834.162	2135007.665	-0.86064	0.045018	0.3112	0.94288	[2,6-dihydroxy-3-(3-phenylpropanoyl)phenyl]oxidanesulfonic acid	20.880515	-	-	-	34.83765	339.344341
POS09187	17928339.45	9894325.314	-0.85757	0.046322	0.3134	2.02114	Atheroline	22.344038	-	-	-	34.83765	338.340914
POS09189	121947.3547	90968.43202	-0.42282	0.096762	0.4024	0.14273	2-(2,4-dihydroxy-5-methoxyphenyl)-3,5,7-trihydroxy-4H-chromen-4	20.607056	-	-	-	34.831	333.278124
POS09190	552587.52	484577.5451	-0.18948	0.668176	0.8368	0.08463	3-Deoxy-D-manno-octulosote 8-phosphate	-15.102772	-	-	-	34.83025	319.174371
POS09191	305012.281	296450.9114	-0.04107	0.802641	0.91	0.00239	Pyridoxamine phosphate	17.026441	-	-	-	34.83025	249.184502
POS09193	234161.6726	434177.8854	0.890781	0.38347	0.6571	0.18399	5-Fluorouridine	4.222714	-	-	-	34.82605	263.200184
POS09202	94945.19654	66393.06509	-0.51606	0.12247	0.4401	0.1192	5-Methylerlodictyol 7-[glucosyl-(1->4)-galactoside]	9.1986238	-	-	-	34.8113	627.57304
POS09203	115788.2282	69583.81059	-0.73466	0.098608	0.4049	0.15408	D-Glucosamine	-20.717923	-	-	-	34.80975	180.174665
POS09205	235244.0951	92821.01178	-1.34164	0.090544	0.39	0.27054	6-(2H-1,3-benzodioxole-5-carbonyloxy)-3,4,5-trihydroxyoxane-2	-2.0163086	-	-	-	34.80665	343.262587
POS09207	73295.71471	55333.22443	-0.40558	0.321831	0.629	0.07353	THF-L-glutamate	11.099264	-	-	-	34.80555	575.556854
POS09208	221314.6611	271979.736	0.2974	0.511806	0.7452	0.10944	Chlorophyll b	19.277099	-	-	-	34.80485	909.52379
POS09211	223841.6806	146721.8781	-0.60939	0.18943	0.5161	0.17827	Bis(4'-chlorophenyl)methane	-23.785944	-	-	-	34.7944	238.126136

POS09213	237640.0702	130426.9822	-0.86554	0.079813	0.3742	0.21955	(5,6-dihydroxy-7-methoxy-2,2-dimethyl-3,4-dihydro-2H-1-benzopyran	-8.435671	-	-	-	34.793	321.314575
POS09215	209923.0739	8908879.915	5.407311	0.34386	0.6332	1.79501	Catechol	-10.151815	-	-	-	34.79215	111.116759
POS09217	546995.9906	374000.6751	-0.54849	0.040901	0.2988	0.31513	Procymidone	-13.708876	-	-	-	34.7852	285.141281
POS09220	560539.839	433727.0288	-0.37003	0.108908	0.4191	0.24405	2-Carboxy-2-hydroxy-8-carboxychromene	-1.9452336	-	-	-	34.7793	237.184417
POS09224	152000.5867	128232.8458	-0.24531	0.195317	0.5208	0.10042	Diflufenican	-22.991594	-	-	-	34.77585	395.293111
POS09230	124480.7907	91895.83578	-0.43785	0.476107	0.7213	0.06884	N-(6-Oxo-6H-dibenzo[b,d]pyran-3-yl)-2,2,2-trifluoroacetamide	-5.8466578	-	-	-	34.7671	308.22958
POS09232	117228.3115	99235.49236	-0.24039	0.200755	0.5255	0.0818	Pyriprole	-18.795221	-	-	-	34.7665	495.266487
POS09235	434909.4716	227799.9853	-0.93295	0.237527	0.5573	0.28643	Daphnoretin	6.4116468	-	-	-	34.74395	353.303935
POS09237	693864.6989	654480.3844	-0.0843	0.643424	0.8248	0.07888	L-Galacturonic acid calcium salt	18.144735	-	-	-	34.7302	427.355912
POS09239	228272.3111	193088.0549	-0.2415	0.257585	0.5759	0.10693	2-amino-4-({1-[(carboxymethyl)-C-hydroxycarbonimidoyl]-2	6.6067939	-	-	-	34.7187	610.611304
POS09241	106755.3398	101778.5113	-0.06888	0.900534	0.9541	0.00455	Dinicozole	7.5906862	-	-	-	34.7152	327.230753
POS09242	156721.2153	108444.8748	-0.53124	0.128018	0.4468	0.15114	2-O-Galloyl-1,4-galactarolactone	18.517057	-	-	-	34.7152	345.241451
POS09244	120838.9355	131297.1405	0.11975	0.6566	0.8314	0.04485	Mometasone	-21.33257	-	-	-	34.7112	428.35946
POS09246	1033665.816	425175.8066	-1.28164	0.088042	0.3875	0.57014	Prusin	-3.3895165	-	-	-	34.7072	296.294176
POS09253	549596.286	441563.0374	-0.31575	0.455082	0.7083	0.1682	D-Ribitol 5-phosphate	21.025024	-	-	-	34.6697	233.137857
POS09254	181003.1477	129409.9665	-0.48407	0.310901	0.6218	0.16321	Fluorodifen	7.9698677	-	-	-	34.668	329.210192
POS09256	69343.18557	2019304.236	4.86396	0.324499	0.6309	0.62909	2,4-Dinitro-1-(3-nitrophenoxy)benzene	-5.0663361	-	-	-	34.6593	306.20563
POS09264	131185.8376	110256.1796	-0.25075	0.67176	0.8378	0.03749	Furaneol 4-(6-malonylglucoside)	1.5263981	-	-	-	34.60435	377.320551
POS09265	230613.3594	194181.4752	-0.24807	0.436759	0.6943	0.10148	Termilin	-16.771024	-	-	-	34.60375	603.366574
POS09266	1125650.598	888812.89	-0.34081	0.085537	0.3848	0.33684	2-Protocatechoylphloroglucinolcarboxylate	-18.551732	-	-	-	34.6018	307.225996
POS09267	76548.07483	35987.98606	-1.08885	0.031586	0.2737	0.15155	1-Hydroxyrutacridone epoxide	22.849363	-	-	-	34.6018	340.35703
POS09270	58655.81217	85287.95149	0.540068	0.515303	0.7477	0.08301	TG(18:0/22:5(4Z,7Z,10Z,13Z,16Z)/18:0)	10.835839	-	-	-	34.59625	938.524035
POS09273	230751.7883	129411.7295	-0.83437	0.044195	0.3084	0.22554	2-S-Glutathionyl acetate	14.483199	-	-	-	34.5853	366.372168

POS09276	157720.604	88843.76999	-0.82803	0.272683	0.5901	0.15379	N2-Citryl-N6-acetyl-N6-hydroxy-L-lysine	-20.300126	-	-	-	34.5748	379.331496
POS09277	8714.332662	2256.766779	-1.94913	0.114502	0.4283	0.05445	Asparenomycin A	3.4149377	-	-	-	34.57275	341.360039
POS09282	811819.8493	393081.4167	-1.04633	0.05348	0.3235	0.48308	Longifolonine	-9.1078939	-	-	-	34.5688	298.309869
POS09285	127846.1412	100581.7562	-0.34604	0.36034	0.6431	0.09659	C.I. Pigment Blue 63	-22.844653	-	-	-	34.5688	450.368011
POS09295	131541.1828	197702.2655	0.587815	0.562242	0.776	0.13129	3,3'-Biflaviolin	-15.999356	-	-	-	34.5439	411.288112
POS09296	133414.8443	76515.75201	-0.80209	0.201377	0.5258	0.15321	2-{[hydroxy(6-hydroxy-7-methoxy-2H-1,3-benzodioxol-5-	18.462956	-	-	-	34.5439	270.221247
POS09298	678160.3206	546227.5647	-0.31212	0.074354	0.3656	0.27011	3,4,5-trihydroxy-6-[(2-hydroxyacetyl)oxy]oxane-2-carboxylic acid	-11.696276	-	-	-	34.54015	253.179327
POS09299	76936.10072	70247.72254	-0.13121	0.571829	0.7807	0.04251	Atalanine	-4.3763764	-	-	-	34.54015	611.614604
POS09302	25388.0431	13577.5562	-0.90293	0.202189	0.5265	0.06006	Glycyrol	11.514226	-	-	-	34.5252	367.375495
POS09303	687768.3132	474931.0299	-0.5342	0.663222	0.834	0.17412	2-[4-hydroxy-3-(sulfooxy)phenyl]acetic acid	14.390437	-	-	-	34.5115	249.220848
POS09306	229383.4285	216510.3581	-0.08333	0.803799	0.9105	0.00282	Diethylstilbestrol diphosphate	-0.8017696	-	-	-	34.5115	429.316933
POS09311	66440.18473	59400.90367	-0.16157	0.584317	0.7882	0.0341	Flavanone 7-O-[alpha-L-rhamnosyl-(1->2)-beta-D-glucoside]	-10.531746	-	-	-	34.5025	549.5373
POS09312	64060.96889	49640.37459	-0.36793	0.249354	0.5669	0.06455	Cefotetan disodium	8.399372	-	-	-	34.5025	620.595081
POS09313	425752.9309	401685.3848	-0.08395	0.529227	0.7568	0.05431	L-Selenomethionine	18.669239	-	-	-	34.5024	197.117238
POS09321	120210.2	79776.86169	-0.59152	0.056316	0.3284	0.15556	Cyfluthrin	-15.02439	-	-	-	34.4854	435.288352
POS09328	76312.4642	63500.38246	-0.26515	0.387596	0.6585	0.06078	Amisulbrom	2.529007	-	-	-	34.47945	467.314256
POS09329	68993.53216	45375.82207	-0.60454	0.118093	0.433	0.11242	Kanokoside D	-9.0630042	-	-	-	34.47855	625.630316
POS09330	161274.5328	124584.0156	-0.3724	0.310528	0.6217	0.11076	Aluminum acetoacetate	-15.294557	-	-	-	34.4773	331.225826
POS09332	138976.5032	233445.7434	0.748246	0.413164	0.6774	0.11962	5-Hydroxysulfamethoxazole	-20.954906	-	-	-	34.4773	270.278634
POS09334	135725.4674	44428.75813	-1.61113	0.000719	0.0721	0.27645	[1-(3-hydroxy-5-methoxyphenyl)-2-(2-hydroxyphenyl)ethoxy]sul	-20.32433	-	-	-	34.47545	341.350359
POS09336	505032.3246	431716.5691	-0.22629	0.275298	0.5921	0.16072	Se-Adenosylselenomethionine	-4.7277917	-	-	-	34.4636	447.345466
POS09337	119438.5537	123353.7735	0.046533	0.87189	0.9428	0.02073	Myricatin	9.6728654	-	-	-	34.4636	553.43062
POS09338	304857.8116	227530.1936	-0.42208	0.410051	0.6751	0.17599	Fospropofol	-18.656385	-	-	-	34.46185	333.241378

POS09339	241726.1633	205026.445	-0.23756	0.122668	0.4403	0.13161	Benzoyl meso-tartaric acid	-20.198105	-	-	-	34.4534	255.195042
POS09343	176559.0199	137630.0475	-0.35936	0.123046	0.441	0.13449	N-Acetyl-L-2-amino-6-oxopimelate	-17.715237	-	-	-	34.4518	232.205781
POS09344	551762.2749	171188.4308	-1.68846	0.116162	0.4311	0.41274	EDTA	-9.995976	-	-	-	34.4504	293.246955
POS09346	312060.7522	364188.4692	0.222858	0.635042	0.8188	0.0475	Streptamine phosphate	-17.780454	-	-	-	34.44915	259.168986
POS09352	291810.5147	229718.3984	-0.34517	0.102275	0.4084	0.16994	cis-(Homo)3-aconitate	-1.538614	-	-	-	34.4431	217.194944
POS09360	122300.6731	83867.44178	-0.54425	0.134014	0.4544	0.13732	5-Amino-6-(5'-phospho-D-ribitylamino)uracil	21.657675	-	-	-	34.4213	357.241392
POS09361	136059.8812	121119.9585	-0.16781	0.644622	0.8254	0.04726	4-Demethylpremithramycin[illegible]	-5.9883317	-	-	-	34.42115	401.340479
POS09362	65170.24708	61880.27876	-0.07473	0.84368	0.9289	0.02619	DTDP-alpha-D-glucose(2-)	-19.096913	-	-	-	34.4206	563.309638
POS09365	149613.0217	93645.92581	-0.67595	0.061843	0.3409	0.19778	Mefloquine	-0.0293684	-	-	-	34.4079	379.319465
POS09367	156124.0673	127766.6034	-0.28918	0.464538	0.7141	0.08548	3,4,5-trihydroxy-6-{[3-(6-hydroxy-7-methoxy-2H-1,2-benzodioxol-5[illegible]	-17.461845	-	-	-	34.3957	417.335007
POS09370	18010.40358	15961.89736	-0.1742	0.515491	0.7477	0.02343	{2-methoxy-4-[(5-oxooxolan-2-yl)methyl]phenyl}oxidane[illegible]	-11.783411	-	-	-	34.3747	303.303714
POS09372	315404.1395	226336.2657	-0.47873	0.012834	0.21	0.25041	5'-(4-Hydroxy-(E)-cinnamoyl) alpha-L-arabinofuranosyl-(1[illegible]	-12.868458	-	-	-	34.369	561.501964
POS09374	355891.6149	307182.1854	-0.21234	0.363618	0.6446	0.13697	Rhein	-24.406359	-	-	-	34.3608	285.22074
POS09377	84123.2624	51622.89114	-0.70449	0.026771	0.2585	0.14784	4-(4-Deoxy-beta-D-gluc-4-enuronosyl)-D-galacturote	-24.255448	-	-	-	34.3556	353.246933
POS09378	262447.3336	232843.9737	-0.17266	0.525129	0.7541	0.05893	3,4-Hexahydroxydiphenoylarabinose	11.675751	-	-	-	34.3548	453.335058
POS09381	1399426.154	677111.2764	-1.04737	0.219405	0.5402	0.5106	4,8-dihydroxy-2H-furo[2,3-h]chromen-2-one	12.516037	-	-	-	34.3389	219.174007
POS09383	171572.8732	157897.4795	-0.11983	0.475697	0.721	0.05175	3,4,5-trihydroxy-6-{[2-(4-methoxy-1-benzofuran-5-yl)-2[illegible]	-5.5987886	-	-	-	34.3389	397.309058
POS09386	200154.138	145988.9282	-0.45525	0.279957	0.5958	0.14506	2-(2,5-dihydroxy-4-methoxyphenyl)-3,5,6,7-tetrahydroxy-3,4-dihydro[illegible]	6.4382203	-	-	-	34.3345	351.288532
POS09388	294837.1506	152892.1284	-0.9474	0.387444	0.6585	0.24233	5-Fluorodeoxyuridine	23.163141	-	-	-	34.3301	247.205379
POS09390	138107.2494	128866.9522	-0.09991	0.550963	0.7699	0.04195	Tuliposide B	0.7649863	-	-	-	34.33	295.262702
POS09391	443780.3536	268477.1106	-0.72505	0.26653	0.5831	0.2946	Metoxadiazone	3.3027746	-	-	-	34.3269	223.20541
POS09392	131109.72	81631.95787	-0.68357	0.04745	0.3143	0.17304	Ibandronate	-19.210211	-	-	-	34.3269	320.230044
POS09394	235210.9249	223803.7106	-0.07172	0.898415	0.953	0.02547	Ascorbate 2-sulfate	-18.744675	-	-	-	34.32115	257.189774

POS09397	73198.99622	24290.21171	-1.59145	0.172215	0.4986	0.14259	Molybdoenzyme molybdenum cofactor	13.68175	-	-	-	34.30825	522.289109
POS09399	210495.095	162287.5034	-0.37523	0.370618	0.6495	0.13145	2-O-(alpha-D-Mannosyl)-D-glycerate	2.967231	-	-	-	34.2932	269.225972
POS09400	179665.1066	136820.6288	-0.39302	0.157723	0.4863	0.13264	3,4,5-trihydroxy-6-(2,4,6-trihydroxybenzoyloxy)oxane-2-carboxylic acid	16.451763	-	-	-	34.2932	347.256973
POS09401	151402.4383	68634.77605	-1.14138	0.126821	0.4452	0.19452	Salicin 6-phosphate	-7.0182581	-	-	-	34.2932	367.262406
POS09403	50840.00619	54522.62277	0.100891	0.773067	0.8949	0.02417	HR1917	1.4406714	-	-	-	34.26135	571.435298
POS09411	375048.6303	363643.0977	-0.04455	0.781317	0.8994	0.02218	Oseltamivir phosphate	-24.481593	-	-	-	34.2516	411.396929
POS09414	89077.94467	35045.90818	-1.34582	0.285675	0.6011	0.1342	SB 228357	-11.191555	-	-	-	34.2437	432.385349
POS09415	192453.8818	145846.6432	-0.40006	0.298947	0.6119	0.12729	2-O-Caffeoyltartronic acid	-17.940916	-	-	-	34.2366	283.205214
POS09416	129476.2877	195729.6053	0.596174	0.293217	0.608	0.16131	Tectorigenin 4'-sulfate	5.726912	-	-	-	34.2331	381.335455
POS09418	265806.1894	239914.804	-0.14785	0.365826	0.6455	0.07823	Flecainide	14.350845	-	-	-	34.21035	415.355923
POS09419	454650.449	354450.707	-0.35917	0.09038	0.3896	0.24377	3'-Methoxyfukiic acid	-20.078245	-	-	-	34.21035	287.23633
POS09420	160329.1633	196969.8641	0.296938	0.739534	0.8769	0.09602	Felodipine	23.922739	-	-	-	34.2068	385.270469
POS09424	194057.3475	159001.1307	-0.28745	0.075301	0.3677	0.1368	Diethyl phenyl phosphate	24.687079	-	-	-	34.177	231.21046
POS09425	269375.4327	207839.4221	-0.37415	0.562526	0.776	0.10098	beta-Chloro-alpha-phenylbenzeneethamine hydrochloride	3.6784699	-	-	-	34.177	269.189863
POS09428	341633.718	285034.0402	-0.26132	0.030278	0.2685	0.20615	Dimethylenetriurea	2.4528523	-	-	-	34.1756	205.194977
POS09430	125325.7918	70889.88979	-0.82203	0.438115	0.6952	0.13238	Alginic acid	-20.568239	-	-	-	34.1756	419.226174
POS09433	113808.6292	106270.1712	-0.09887	0.646549	0.8266	0.03664	6-({13,14-dihydroxy-9-oxo-8,17-dioxatetracyclo[8.7.0.0	14.607414	-	-	-	34.1625	461.361001
POS09437	1251.418232	417859.5914	8.38331	0.34102	0.6326	0.28222	Carbovir Triphosphate	-20.786018	-	-	-	34.148	488.19005
POS09439	134152.3323	74240.85112	-0.85359	0.039025	0.2955	0.19698	5-Phosphoribosyl-4-carboxy-5-aminoimidazole	-11.729236	-	-	-	34.146	337.177334
POS09440	151321.0337	132595.0155	-0.19059	0.321236	0.6288	0.0606	5-Hydroxydantrolene	8.8947908	-	-	-	34.146	331.262614
POS09444	231586.6046	214342.8755	-0.11163	0.634306	0.8188	0.03851	6-O-Methylarmillaridin	-8.674349	-	-	-	34.14265	463.966261
POS09449	137302.4761	115715.7659	-0.24677	0.338397	0.6326	0.08004	Diclobutrazol	8.2430605	-	-	-	34.1393	329.246882
POS09450	172124.8723	100105.0944	-0.78194	0.026225	0.2576	0.21792	2-Galloyl-1,4-galactarolactone methyl ester	-13.458696	-	-	-	34.1393	359.256855

POS09452	154056.9377	75604.62773	-1.02692	0.053048	0.3235	0.21599	Glutathioselenol	-7.2625389	-	-	-	34.1393	387.287971
POS09457	50787.09387	30509.44642	-0.73521	0.400284	0.6679	0.07494	3'-Phosphoadenylyl sulfate	4.6721017	-	-	-	34.13355	508.273947
POS09458	233662.4218	226794.7186	-0.04304	0.75178	0.8841	0.01748	N(omega)-Nitro-L-arginine	-0.0458391	-	-	-	34.13255	220.205767
POS09459	485210.9716	362515.2123	-0.42057	0.009615	0.1933	0.31136	Maclurin	-3.3544481	-	-	-	34.13145	263.221297
POS09464	2345923.556	727724.7554	-1.68869	0.198586	0.5243	0.94848	2-Hydroxy-6-oxo-6-phenylhexa-2,4-dienoate	-9.5781545	-	-	-	34.12255	219.210587
POS09465	420228.5648	343195.4787	-0.29214	0.099147	0.4054	0.19209	3,5-Dihydroxyphenyl 1-O-(6-O-galloyl-beta-D-glucopyranoside)	21.341506	-	-	-	34.12135	441.371574
POS09466	158399.6328	198656.3936	0.326706	0.391557	0.6612	0.11543	Anagrelide	-15.206571	-	-	-	34.1192	257.091382
POS09468	59583.98005	163352.322	1.454991	0.010518	0.1967	0.27464	Clomeprop	14.393874	-	-	-	34.1192	325.213743
POS09477	92254.02878	145470.9203	0.657047	0.148748	0.4744	0.14527	Demethylbellidifolin	-2.7819964	-	-	-	34.1078	261.205553
POS09478	193639.5474	132101.2997	-0.55173	0.251671	0.5699	0.15052	3,4,5-trihydroxy-6-({3-oxo-3-[(3,4,5,6-tetrahydroxyoxan-2	-0.5165918	-	-	-	34.1078	443.333048
POS09480	62479.25634	34072.06567	-0.87479	0.129067	0.4476	0.11443	3,4-Dihydro-3-hydroxy-4-S-glutathionyl bromobenzene	-17.954263	-	-	-	34.1062	481.329453
POS09491	2085881.644	1843122.029	-0.17851	0.272707	0.5901	0.30121	Quercetin 3-O-(6-O-malonyl-beta-D-glucoside)	-12.413793	-	-	-	34.05775	551.422944
POS09492	63020.41283	56973.21602	-0.14554	0.516481	0.7481	0.02903	1-O,6-O-Digalloyl-beta-D-glucose	-22.41779	-	-	-	34.0458	485.360818
POS09493	1071680.753	118123.2307	-3.18151	0.31676	0.6253	0.53841	N-Acetyldemethylphosphinothricin tripeptide	-3.1960472	-	-	-	34.0458	352.298854
POS09506	240844.3375	71093.5778	-1.76031	0.068894	0.3541	0.30978	Shoyuflavone B	8.7586692	-	-	-	34.0283	403.3193
POS09508	324566.5975	494623.6856	0.607817	0.263185	0.58	0.26078	Gallamine Triethiodide	-5.4390675	-	-	-	34.0253	892.531528
POS09509	168872.1235	105185.339	-0.683	0.260558	0.5788	0.14787	3,4,5-trihydroxy-6-(6-hydroxy-7-methoxy-2H-1,3-benzodioxole-5	-10.270977	-	-	-	34.02325	389.284289
POS09512	315565.5776	180468.2498	-0.80619	0.288099	0.603	0.21263	Talc	14.3128	-	-	-	34.0077	380.278405
POS09517	150991.8119	122316.1183	-0.30386	0.360707	0.6433	0.08547	Fosphenytoin	19.389175	-	-	-	33.9986	363.288201
POS09518	207370.7982	107141.7202	-0.95269	0.11168	0.4241	0.25186	Brevifolincarboxylic acid 9-sulfate	11.42804	-	-	-	33.9986	373.272531
POS09519	213554.7704	199575.7701	-0.09767	0.658094	0.8318	0.02368	Methylgallic acid-O-sulphate	-2.3702598	-	-	-	33.9954	265.21565
POS09521	346468.272	261587.4781	-0.40543	0.220062	0.5412	0.19522	Tetracenomycin D3	7.3890338	-	-	-	33.9831	381.314587
POS09527	173508.7165	123066.5329	-0.49557	0.020429	0.2388	0.18135	Epigallocatechin 3-gallate	7.6924348	-	-	-	33.9598	459.382503

POS09529	113703.5222	98961.17805	-0.20034	0.412144	0.6765	0.07425	Arbutin	-4.1515542	-	-	-	33.9591	273.257346
POS09531	118813.0599	93743.9705	-0.3419	0.133177	0.4535	0.10155	Theogallin	0.3025178	-	-	-	33.95255	345.278181
POS09533	249907.027	231506.9549	-0.11034	0.577667	0.7844	0.04677	Cryptocyanin	20.466008	-	-	-	33.952	481.401008
POS09535	179231.6649	76500.89832	-1.22828	0.017597	0.2275	0.26472	FMN	-5.7985261	-	-	-	33.9263	457.34843
POS09536	39642.01775	9883.618526	-2.00392	0.295975	0.6096	0.11191	5-(5-Phospho-D-ribosylaminoformimino)-1-(5-phosphoribosyl)	23.174639	-	-	-	33.9263	578.351656
POS09541	278641.1175	260200.8157	-0.09878	0.907614	0.9575	0.01631	3-oxobrimonidine	19.508476	-	-	-	33.9086	311.163327
POS09546	209842.0205	68667.71461	-1.6116	0.237345	0.5572	0.23525	Cliomide	-5.4172916	-	-	-	33.88535	433.272835
POS09547	85477.55048	74183.37675	-0.20445	0.577024	0.7841	0.04773	Tizoxanide glucuronide	-2.2672882	-	-	-	33.88345	442.375276
POS09549	213914.9224	85623.33797	-1.32096	0.081695	0.378	0.27132	6-Methylthiopurine 5'-monophosphate ribonucleotide	-14.39501	-	-	-	33.86035	379.300031
POS09550	140828.0954	52080.1218	-1.43513	0.00097	0.0736	0.27493	Homatropine Methylbromide	17.554807	-	-	-	33.8589	371.294777
POS09551	2898922.674	1691665.094	-0.77707	0.332846	0.6326	0.66441	5-Sulfosalicylate	17.076912	-	-	-	33.8433	219.194903
POS09553	124912.8501	104319.6032	-0.25991	0.15542	0.4838	0.10609	Myricetin 3-arabinoside	-2.9083276	-	-	-	33.8433	451.355667
POS09554	906636.1625	330488.5266	-1.45592	0.336101	0.6326	0.40829	Violanthin	-21.285648	-	-	-	33.8433	579.513662
POS09559	223798.298	209661.2125	-0.09414	0.853299	0.9334	0.01394	Imazalil nitrate	-0.5680057	-	-	-	33.8324	361.199672
POS09561	284955.7171	198761.0455	-0.5197	0.234325	0.5554	0.19036	Dimethyl 2-galloylgalactarate	-11.155029	-	-	-	33.83185	391.299123
POS09563	509915.8258	452162.0727	-0.17342	0.374777	0.6504	0.12024	8-Hydroxypinoresinol 4-glucoside	4.1033308	-	-	-	33.8289	537.534578
POS09566	295444.259	81825.70011	-1.85226	0.14753	0.4732	0.32576	Baicalin	-9.7529117	-	-	-	33.821	447.363923
POS09567	122433.6939	102698.9165	-0.25358	0.210597	0.5339	0.07308	Flucarbazone-sodium	12.271057	-	-	-	33.81805	419.293409
POS09568	158483.8507	107073.2471	-0.56574	0.036969	0.2884	0.17777	Xanthotoxol glucoside	-18.475085	-	-	-	33.8058	365.304046
POS09569	426047.9809	346786.8416	-0.29697	0.105292	0.4125	0.19488	{4-[2,3-dioxo-3-(2,4,6-trihydroxy-3-methoxyphenyl)propyl]-2	-1.4156373	-	-	-	33.8058	445.366648
POS09570	193368.5289	67612.66204	-1.51599	0.050646	0.3192	0.27884	Tenitramine	24.380453	-	-	-	33.8057	417.316226
POS09571	158612.4067	117357.4949	-0.4346	0.152712	0.4804	0.1479	3,4,5-trihydroxy-6-{[2-(2,3,4-trihydroxyphenyl)acetyl]o	-16.036915	-	-	-	33.8044	361.272499
POS09573	368163.8405	358779.21	-0.03725	0.927177	0.9669	0.03063	16alpha-Bromo-17beta-estradiol	-4.6217089	-	-	-	33.80335	352.283653

POS09578	107336.7263	98316.95893	-0.12663	0.599559	0.7973	0.04127	N-Sulfo-D-glucosamine	-17.529549	-	-	-	33.79205	260.237032
POS09582	188121.6991	197793.7883	0.072331	0.621983	0.8124	0.04521	Threoninyl-Glutamate	18.540466	-	-	-	33.7911	248.23706
POS09584	121556.7223	56650.53911	-1.10147	0.053056	0.3235	0.18932	Cellobiono-1,5-lactone	-12.34708	-	-	-	33.7904	341.283675
POS09587	167949.0625	143230.3688	-0.22969	0.261379	0.5789	0.10426	5-Carboxy-2'-deoxyuridine	7.9651469	-	-	-	33.7857	273.220945
POS09588	218028.348	88523.84024	-1.30038	0.002734	0.1138	0.33986	1,2,3,4-Tetrahydro-alpha,7-dihydroxy-beta-(hydroxymethyl)-9	-6.1999469	-	-	-	33.7857	349.309217
POS09591	519343.4867	765551.3716	0.55981	0.23786	0.5574	0.31369	Chlorophyll a	22.518113	-	-	-	33.7835	895.543419
POS09598	222266.5142	170046.0251	-0.38637	0.015826	0.2226	0.1834	(-)-Epicatechin 3-O-gallate	8.8132851	-	-	-	33.77775	443.387175
POS09599	128272.6787	113512.6885	-0.17636	0.340865	0.6326	0.06315	Cellulose triacetate	13.607121	-	-	-	33.77515	538.537591
POS09600	324510.1916	401416.9815	0.306838	0.187219	0.513	0.21238	Phaseolic acid	16.88046	-	-	-	33.7742	297.241877
POS09601	211781.3	139884.6619	-0.59834	0.419543	0.681	0.15184	3-O-Ethyl-L-ascorbic acid	-24.103547	-	-	-	33.7742	205.179655
POS09602	627328.0432	490870.5443	-0.35388	0.068698	0.3541	0.28475	Prostalidin A	-20.094921	-	-	-	33.7742	395.330453
POS09603	96585.39849	97902.59779	0.019542	0.935102	0.971	0.01055	2,3,3',4,4',5,5'-Heptachlorobiphenyl	7.8075932	-	-	-	33.7742	396.333563
POS09609	4401417.901	3243393.76	-0.44046	0.091925	0.3933	0.76195	Cobalt sulfate	19.108601	-	-	-	33.74195	282.115448
POS09610	217734.661	202802.6539	-0.10249	0.551348	0.7699	0.04588	trans-Zeatin riboside diphosphate	-17.571029	-	-	-	33.74195	512.315792
POS09611	109696.0359	62774.11213	-0.80527	0.000686	0.0721	0.20793	Miraxanthin-II	9.5197289	-	-	-	33.74195	327.269282
POS09614	124738.3249	90558.14653	-0.46199	0.193741	0.5196	0.12407	3,4,5-trihydroxy-6-{[2-oxo-3-(2,3,4,5-tetrahydroxyphenyl)propa	-23.280221	-	-	-	33.7358	405.277865
POS09621	234368.3662	185453.2189	-0.33772	0.566412	0.7778	0.07244	6-Thioxanthine 5'-monophosphate	0.1195289	-	-	-	33.7026	381.278322
POS09622	130523.5125	58462.38586	-1.15873	0.083484	0.3801	0.20184	Carmine	-13.689546	-	-	-	33.7026	493.386936
POS09628	326042.1749	266174.4482	-0.29269	0.085379	0.3847	0.17233	6-Deoxy-6-sulfo-D-fructose 1-phosphate	-16.725932	-	-	-	33.6881	325.201454
POS09638	72068.23013	37068.41909	-0.95917	0.082629	0.3793	0.13486	Kalbreclasine	-11.828424	-	-	-	33.64965	470.397824
POS09639	395613.8337	247078.8885	-0.67912	0.336952	0.6326	0.19999	4,4',6,6'-Tetranitro-2,2'-azoxytoluene	18.572018	-	-	-	33.646	407.278822
POS09640	332838.8076	136167.1188	-1.28945	0.483312	0.7262	0.23504	(2-{[5,7-dihydroxy-2-(3-hydroxyphenyl)-4-oxo-4H-chromen-3-yl]oxy}-4	-7.3037279	-	-	-	33.63845	499.413636
POS09641	76196.88655	57990.95068	-0.3939	0.30476	0.6169	0.06739	N1-(5-Phospho-alpha-D-ribosyl)-5,6-dimethylbenzimidazole	7.7515692	-	-	-	33.6287	359.293654

POS09645	328497.2454	371959.9942	0.179266	0.452724	0.7066	0.13147	DHAP(6:0)	9.2405746	-	-	-	33.6145	269.210555
POS09646	247098.4275	175883.3657	-0.49047	0.060201	0.3376	0.19999	3-Hydroxy-L-glutamate	-9.505806	-	-	-	33.6142	164.134426
POS09649	456955.5106	410209.8683	-0.15569	0.403797	0.6703	0.10439	Diflunisal	-20.05326	-	-	-	33.5967	251.199859
POS09651	6439040.447	4641758.66	-0.47217	0.055391	0.3276	1.02479	Erythro-4-hydroxy-L-glutamate(1-)	10.554978	-	-	-	33.588	163.130988
POS09652	366637.2194	545394.3415	0.572946	0.036405	0.2865	0.31972	6-Methylthioguanosine monophosphate	-5.7685561	-	-	-	33.588	394.317808
POS09654	87903.67113	62555.51085	-0.49079	0.100436	0.407	0.11865	Buchanine	-17.243975	-	-	-	33.58325	286.252358
POS09655	216124.8017	171168.594	-0.33645	0.20815	0.5321	0.13362	Trifloxystrobin	8.5986944	-	-	-	33.5793	409.381888
POS09656	104302.7484	88136.4278	-0.24297	0.3606	0.6432	0.07269	Tetracenomycin C	-15.797129	-	-	-	33.5793	473.398114
POS09657	108836.2974	73202.29623	-0.5722	0.052922	0.3235	0.14461	3,4,5,11,14,20,21,22-octahydroxy-13-(hydroxymethyl)-9,12,16- [illegible]	-21.298305	-	-	-	33.5793	483.345503
POS09659	276566.2857	265503.0494	-0.0589	0.82252	0.9184	0.03165	Bikaverin	6.2519743	-	-	-	33.579	383.330067
POS09661	714424.469	491434.7121	-0.53978	0.211348	0.5346	0.27896	Sodium ortho-phenylphete	-6.4635437	-	-	-	33.5787	193.195034
POS09665	177639.1156	108638.8765	-0.70941	0.02969	0.2674	0.21839	Pachyrrhizone	-23.573346	-	-	-	33.55245	367.319641
POS09666	439963.381	312702.896	-0.49259	0.000971	0.0736	0.33954	Dihydro-ME	-21.836553	-	-	-	33.54345	399.361378
POS09667	143381.8611	96294.47853	-0.57434	0.015769	0.2226	0.17644	Fluacrypyrim	-1.8168194	-	-	-	33.54345	427.392902
POS09668	171245.1301	171835.8237	0.004968	0.9841	0.9921	0.00037	Eucommin A	-16.218642	-	-	-	33.5425	551.550047
POS09672	656505.9679	518286.1719	-0.34106	0.036941	0.2884	0.29918	{2,6-dimethoxy-4-[3-oxo-3-(2,4,6-trihydroxyphenyl)prop-1- [illegible]	-0.9179462	-	-	-	33.50725	413.376898
POS09676	190034.0784	55542.94048	-1.77458	0.11981	0.4353	0.26238	6-[3-(6,7-dimethoxy-2H-1,3-benzodioxol-5-yl)oxiran-2- [illegible]	-9.0612473	-	-	-	33.50095	445.34825
POS09678	487993.5541	359585.8731	-0.44053	0.067277	0.352	0.27151	2-Sulfotrehalose	-13.555167	-	-	-	33.4916	423.361251
POS09679	126097.7072	122392.4008	-0.04303	0.843771	0.9289	0.00384	Acadesine	11.964312	-	-	-	33.48795	259.241666
POS09680	248638.0316	167001.5323	-0.57419	0.185674	0.5117	0.18151	Oxaziclomefone	-0.7854549	-	-	-	33.48795	377.283381
POS09682	147477.3858	68012.66186	-1.11662	0.015255	0.2226	0.23758	Tomentin	-5.4830856	-	-	-	33.4864	347.293678
POS09683	100572.4608	109278.7091	0.119777	0.822423	0.9184	0.04631	Kimycin D	-13.831136	-	-	-	33.4812	455.387292
POS09686	87644.42658	75075.25964	-0.22332	0.419485	0.681	0.05376	Pantoyllactone glucoside	-22.031611	-	-	-	33.4696	293.283237

POS09691	1080417	978757.0434	-0.14257	0.186935	0.5126	0.22276	{11-methoxy-16,18-dioxo-6,8,19-trioxapentacyclo[10.7.0.0	19.812732	-	-	-	33.46385	409.345367
POS09692	69665.91246	55358.95328	-0.33164	0.257707	0.576	0.06378	Halofantrine	-2.8190353	-	-	-	33.46275	501.429566
POS09694	357121.7435	297782.008	-0.26216	0.080293	0.3749	0.17794	Chamaemeloside	-23.13039	-	-	-	33.4614	577.496742
POS09695	102783.9672	71886.45167	-0.51582	0.045864	0.3134	0.13709	3,4,5-trihydroxy-2-[hydroxy(3,4,5-trihydroxyoxan-2	-24.981622	-	-	-	33.4588	467.387625
POS09696	111703.9284	128923.3681	0.206834	0.332856	0.6326	0.08165	Niflumic acid	2.8099604	-	-	-	33.4581	283.22607
POS09698	110626.4652	47212.0136	-1.22847	0.219425	0.5402	0.15874	Spectinomycin dihydrochloride	2.4994729	-	-	-	33.4581	406.27969
POS09699	637774.8155	537308.7623	-0.2473	0.097242	0.4032	0.22401	Methyl nogalote	-21.271662	-	-	-	33.4555	397.345746
POS09700	69923.52442	90138.95308	0.366373	0.318122	0.6265	0.0909	(5-L-Glutamyl)-L-glutamine	8.757113	-	-	-	33.4529	276.268187
POS09701	53554.0391	52482.06556	-0.02917	0.93911	0.9725	0.00666	Tolcapone	16.625186	-	-	-	33.4526	274.252619
POS09702	183503.4722	137315.3702	-0.41831	0.070288	0.357	0.15795	Cromolyn	-16.100228	-	-	-	33.4526	469.366236
POS09703	191937.796	127904.5914	-0.58557	0.122435	0.4401	0.18511	Apigenin	-9.4317337	-	-	-	33.45165	271.241628
POS09704	362190.5923	344975.017	-0.07026	0.613219	0.8075	0.05945	Gonyautoxin 1	21.741547	-	-	-	33.45055	412.36472
POS09705	155914.7779	169112.7744	0.117228	0.681564	0.8432	0.03422	Macrozamin	4.9777029	-	-	-	33.4504	385.34569
POS09707	232035.2601	66471.69504	-1.80353	0.097531	0.4036	0.30017	Quercitrin	-10.763428	-	-	-	33.4482	449.379351
POS09708	134948.8829	228390.9882	0.759093	0.549344	0.769	0.15491	Piceatannol 4'-galloylglucoside	-13.835606	-	-	-	33.4467	559.48705
POS09709	1367806.524	1091978.426	-0.32492	0.107916	0.4178	0.36635	CMP	-16.660856	-	-	-	33.44635	324.198392
POS09710	657039.7144	543043.7506	-0.27491	0.04821	0.3148	0.2553	1,4-Bis(chloromethoxymethyl)benzene	-17.975422	-	-	-	33.44635	236.11015
POS09713	615102.0832	892545.2266	0.537099	0.385729	0.6583	0.29924	Methyl aklanote	4.2694037	-	-	-	33.4452	411.382529
POS09715	301328.3618	255020.0566	-0.24073	0.888741	0.9492	0.119	Amritoside	23.937861	-	-	-	33.4452	627.496073
POS09717	155709.4593	160129.93	0.040386	0.85251	0.933	0.01255	Flazasulfuron	3.3607857	-	-	-	33.44295	408.333746
POS09719	87705.00077	84933.19966	-0.04633	0.933966	0.9703	0.00064	Pancratistatin	17.097876	-	-	-	33.4414	326.283638
POS09721	225962.2671	193196.4347	-0.22601	0.078143	0.3724	0.12113	CI Orange G	-11.35337	-	-	-	33.43855	453.371441
POS09722	39942.28587	49538.29666	0.310627	0.307897	0.619	0.06033	{2-[(3-methylbut-2-enoyl)oxy]-2-{9-[(3-methylbut-2-enoyl)oxy]-2	-15.239695	-	-	-	33.4357	523.519314

POS09724	213556.1938	234244.3776	0.133399	0.533838	0.7588	0.06888	Cefuroxime sodium	16.633643	-	-	-	33.4066	447.381801
POS09725	201998.6602	181604.6765	-0.15354	0.47486	0.7207	0.0607	O-Acetyl-ADP-ribose	15.678103	-	-	-	33.4066	602.369105
POS09727	47619.87601	51934.18438	0.125121	0.615592	0.8087	0.01587	Flunitrazepam	-22.078746	-	-	-	33.4021	314.28356
POS09728	123692.3808	107846.5437	-0.19778	0.477294	0.7223	0.06092	N-Adenylylanthranilate	3.6329986	-	-	-	33.401	467.350871
POS09731	111858.2125	45327.9153	-1.3032	0.007172	0.1779	0.23162	6-{3,5-dihydroxy-2-[3,4,5-trihydroxy-6-(hydroxymethyl)oxan-2	-24.520289	-	-	-	33.3687	465.37189
POS09734	193649.672	149262.7873	-0.37559	0.643674	0.825	0.07275	Lactitol dihydrate	1.0796037	-	-	-	33.3561	381.350587
POS09737	92989.10485	100581.2649	0.113228	0.67912	0.8418	0.01939	CGS 7181	8.49987	-	-	-	33.338	407.365931
POS09738	101778.7146	69362.69769	-0.5532	0.060268	0.3376	0.13289	6-{3,5-dihydroxy-2-[3-(3,4,5-trimethoxyphenyl)propano	7.828673	-	-	-	33.3238	525.486383
POS09739	200054.1478	155916.1184	-0.35962	0.121968	0.4395	0.14699	Kaempferol 3-O-beta-D-xyloside	-18.178067	-	-	-	33.3148	419.350572
POS09744	99303.33118	100904.2956	0.023074	0.902119	0.9547	0.01434	4-Keto-anhydrotetracycline	18.026395	-	-	-	33.2849	398.349439
POS09745	160199.8887	67464.2743	-1.24768	0.073851	0.3652	0.23471	alpha-L-Rhamnopyranosyl-(1->2)-beta-D	-3.8755348	-	-	-	33.2849	503.426529
POS09747	206741.6946	232806.0619	0.171299	0.835608	0.9247	0.0362	Lancerin	7.9426707	-	-	-	33.2572	407.350704
POS09748	58228.73649	45928.49332	-0.34234	0.344923	0.6341	0.06767	7-Methylxanthosine	2.9588125	-	-	-	33.244	300.268162
POS09751	4004511.338	3551201.459	-0.17332	0.469282	0.7174	0.39577	Isoimide	14.273953	-	-	-	33.24215	307.166247
POS09752	129741.2551	80485.75333	-0.68883	0.075273	0.3677	0.17925	Aluminoparaaminosalicylate calcium	16.203314	-	-	-	33.24145	405.335028
POS09753	25586811.65	22342267.55	-0.19562	0.410087	0.6751	1.11603	Dichlorphemide	-10.507569	-	-	-	33.2381	306.16287
POS09755	1035655.096	901519.6995	-0.20011	0.221185	0.5422	0.22275	Chloramphenicol 3-acetate	-2.6271181	-	-	-	33.2097	366.172417
POS09760	39795.19647	35074.20647	-0.18218	0.325932	0.6316	0.04584	Acetaminophen glucuronide	15.700858	-	-	-	33.1754	328.299115
POS09765	183205.5534	70124.77082	-1.38547	0.190997	0.5179	0.22578	Neoastilbin	-11.741226	-	-	-	33.1713	451.394788
POS09766	134133.0634	128840.5312	-0.05808	0.867176	0.9403	0.00123	Quercetin 3-O-[beta-D-xylosyl-(1->2)-beta-D-glucoside]	-20.199669	-	-	-	33.1713	597.486128
POS09767	120323.7027	44560.02093	-1.4331	0.07068	0.3578	0.21097	3,4,5-trihydroxy-6-({11-methoxy-13-oxo-6,8,20-trioxapentacyclo[10.8.0.0i	-17.222757	-	-	-	33.1669	501.410658
POS09768	147360.762	60605.54602	-1.28183	0.124198	0.4422	0.21124	3,4,5-trihydroxy-6-({11-hydroxy-13-oxo-6,8,20-trioxapentacyclo[10.8.0.0i	4.8061068	-	-	-	33.16485	489.410624
POS09770	81298.62581	57520.46303	-0.49916	0.131009	0.4499	0.11602	Nicergoline	9.2705462	-	-	-	33.1584	485.397767

POS09774	67518.22688	62745.18365	-0.10577	0.622027	0.8124	0.01854	Dikegulac	-5.1130109	-	-	-	33.138	275.272974
POS09776	90850.70458	51087.06014	-0.83054	0.016086	0.2228	0.16494	Ephedrannin A	-14.566298	-	-	-	33.1378	557.472371
POS09777	265702.4362	96909.83669	-1.4551	0.11865	0.4338	0.29894	6-Hydroxyluteolin 6-xyloside	10.314983	-	-	-	33.13485	435.362057
POS09778	313736.6223	214660.3091	-0.5475	0.663829	0.8345	0.16733	Nitarsone	23.811469	-	-	-	33.1317	248.050259
POS09779	132135.0295	105031.3706	-0.33119	0.347286	0.6349	0.10653	Biochanin A-beta-D-glucoside	-21.915085	-	-	-	33.1317	447.401594
POS09782	160425.7089	36235.22776	-2.14644	0.094261	0.3975	0.2623	3',5'-Cyclic AMP	23.274332	-	-	-	33.1293	330.220839
POS09783	124397.1377	103743.3281	-0.26193	0.163935	0.4916	0.08815	[3,5-dihydroxy-2-(5,6,7-trihydroxy-4-oxo-2-phenyl-4H-chromen-8	3.0347605	-	-	-	33.1292	483.418741
POS09784	1262251.196	1181817.508	-0.09499	0.58826	0.7904	0.12417	Delphinidin 3-O-(6''-O-malonyl)-beta-D-glucoside	-16.64424	-	-	-	33.12685	552.428498
POS09786	88263.17787	50307.97475	-0.81102	0.03503	0.2838	0.15468	Nocardicin E	9.3707675	-	-	-	33.1182	400.365219
POS09787	212833.7214	156071.2358	-0.44752	0.188892	0.516	0.14971	Bicalutamide	5.8604708	-	-	-	33.1167	431.383199
POS09788	166073.3669	147358.4853	-0.17249	0.386174	0.6583	0.08084	Quercetin 3,3'-bissulfate	16.059074	-	-	-	33.1167	463.376802
POS09789	32114.6452	26437.12638	-0.28067	0.471329	0.719	0.03578	6-{[1-(2-{[2-(3,4-dimethoxyphenyl)ethyl]-C	0.8565764	-	-	-	33.11595	552.552749
POS09791	33917.31577	28942.78001	-0.22882	0.564321	0.7766	0.02665	Dopaxanthin	11.827526	-	-	-	33.1105	391.355993
POS09794	1157578.29	935452.679	-0.30737	0.219738	0.5406	0.28084	6-({2-[4-ethyl-3-(sulfooxy)phenyl]-3,5-dihydroxy-3,4-dihydro	-13.339102	-	-	-	33.1058	559.509827
POS09799	234935.2034	50766.11315	-2.21033	0.233153	0.554	0.25394	7-Methoxypradimicinone II	-0.5185055	-	-	-	33.09775	580.515276
POS09809	119165.0489	39716.8126	-1.58514	0.095983	0.4006	0.20843	(2-{[2-(3,4-dihydroxyphenyl)-5,7-dihydroxy-4-oxo-3,4	9.0032158	-	-	-	33.0525	517.441926
POS09813	49342.4723	36335.36835	-0.44146	0.104642	0.4116	0.075	Gardenoside	-3.5073289	-	-	-	33.0306	405.371758
POS09816	553015.0783	500791.067	-0.14311	0.360577	0.6432	0.10816	ringin	-23.773822	-	-	-	33.0149	581.528075
POS09820	156167.8003	36614.28466	-2.09262	0.13783	0.4613	0.24187	Tolylfluanid	11.056574	-	-	-	33.012	348.267916
POS09822	123306.6386	130478.071	0.081557	0.701819	0.8538	0.03009	Muscapurpurin	-9.891907	-	-	-	33.012	419.314339
POS09824	137328.2515	97697.72881	-0.49123	0.049782	0.3188	0.15041	N-[(7,8-Dihydropterin-6-yl)methyl]-4-(beta-D-ribofuranosyl)aniline 5'	-19.427401	-	-	-	33.012	483.382305
POS09828	166083.6764	26135.3524	-2.66784	0.182402	0.5082	0.25019	Carboplatin	17.989448	-	-	-	32.98635	372.267955
POS09829	95141.43323	59015.89767	-0.68897	0.068063	0.3534	0.13658	3,4,5-trihydroxy-6-[(5-hydroxy-6-methoxy-4-oxo-2-phenyl-4H	-2.6057953	-	-	-	32.98605	461.397077

POS09832	173496.4202	95725.77538	-0.85793	0.231666	0.553	0.18936		3,4,5-trihydroxy-6-[1-(7-methoxy-2-oxo-2H-chromen-6-yl)-3	3.2908178	-	-	-	32.9734	439.393719
POS09834	115612.6231	160379.3904	0.47219	0.662494	0.8336	0.07662		9-Bromo-16alpha-methyl-pregn-4-ene-3,11,20-trione	4.4835504	-	-	-	32.9607	422.377066
POS09836	52948.43419	46689.81142	-0.18148	0.50936	0.7445	0.03413		Mahuannin D	7.1049743	-	-	-	32.9598	529.517232
POS09845	155586.2922	67029.67355	-1.21484	0.130196	0.4485	0.20754		[2-(5,7-dihydroxy-4-oxo-2-phenyl-4H-chromen-6-yl)-3-hydroxy-6-methyl-5	-2.1150026	-	-	-	32.92925	479.426265
POS09846	83656.32249	43016.3925	-0.95959	0.027878	0.2623	0.16032		Tetraphyllin B sulfate	-15.972919	-	-	-	32.92595	368.330409
POS09847	179810.0787	81466.58913	-1.14219	0.147022	0.4726	0.20281		Lamiide	-16.381765	-	-	-	32.9255	423.381457
POS09848	151485.9485	28946.93878	-2.3877	0.163962	0.4916	0.24018		Prothiofos	-1.3036138	-	-	-	32.9107	346.252227
POS09849	130456.1887	60507.61189	-1.10838	0.165544	0.4924	0.18129		2''-O-Acetylisoorientin	10.4085	-	-	-	32.9071	491.425981
POS09850	98918.25274	62157.89064	-0.6703	0.177364	0.5035	0.11683		Neolinustatin	-13.883317	-	-	-	32.9071	424.413698
POS09856	127948.8921	51916.38821	-1.30131	0.067496	0.3525	0.21403		Nilutamide	-22.069487	-	-	-	32.88765	318.220976
POS09858	402016.7296	523052.625	0.379701	0.64677	0.8266	0.17775		N-Acetyl-N6,N6,O-tridemethylpuromycin-5'-phosphate	3.6184407	-	-	-	32.8673	552.455672
POS09859	169280.3303	29099.70359	-2.54034	0.212316	0.5354	0.24184		Apholate	-22.358294	-	-	-	32.841	388.299117
POS09862	99282.52211	60422.39189	-0.71646	0.018043	0.2285	0.16324		N'-Phosphoguanidinoethyl methyl phosphate	13.638626	-	-	-	32.8298	278.120456
POS09865	85533.32197	101605.003	0.248413	0.515427	0.7477	0.05582		Tetracenomycin E	4.8754574	-	-	-	32.81365	409.366868
POS09866	1457224.666	1266014.712	-0.20293	0.174428	0.5007	0.30938		{4-[2,3-dioxo-3-(2,4,6-trihydroxy-3-methoxyphenyl)propyl]-2	10.087912	-	-	-	32.7907	429.371598
POS09868	84936.66205	63533.0268	-0.41888	0.315463	0.6247	0.08518		Secogalioside	-15.972392	-	-	-	32.7885	421.365862
POS09869	169745.7669	139478.5224	-0.28333	0.535744	0.7591	0.08264		Cinchoin 1a	-15.902501	-	-	-	32.7885	453.410282
POS09870	101192.8968	86662.2746	-0.22363	0.529619	0.757	0.04552		Lippioside I	-6.2865549	-	-	-	32.7853	539.501791
POS09871	198712.9627	187524.5217	-0.08361	0.908394	0.9578	0.00487		Mefluidide	-19.96574	-	-	-	32.78375	311.293781
POS09872	172721.026	103339.4415	-0.74105	0.179688	0.5058	0.17184		Permethrin	-20.309378	-	-	-	32.78375	392.28703
POS09875	191613.7734	144875.0629	-0.40339	0.374087	0.6502	0.13297		6-{[3,5-dihydroxy-2-(1-hydroxy-3-methoxy-4-oxocyclohexyl)-6	6.9789515	-	-	-	32.7832	543.457062
POS09876	108662.2909	47080.07084	-1.20666	0.130985	0.4499	0.17592		Triflusulfuron-methyl	9.7631489	-	-	-	32.7814	493.441684
POS09879	109274.2964	51731.39277	-1.07884	0.142341	0.4668	0.16014		4-Deoxy-beta-D-gluc-4-enuronosyl-(1,3)-N-acetyl-D-galactosamine	16.597942	-	-	-	32.7739	380.330172

POS09881	106547.5929	65202.47754	-0.7085	0.086651	0.3863	0.14674	Isoscoparine	4.2226292	-	-	-	32.7739	463.412729
POS09882	136259.0979	63792.93644	-1.09488	0.14637	0.4718	0.17915	[2-(5,7-dihydroxy-4-oxo-2-phenyl-4H-chromen-8-yl)-3,5-dihydroxyoxan-4	18.558565	-	-	-	32.77015	467.425933
POS09884	179404.1001	202424.9739	0.174174	0.755884	0.8865	0.06039	Epitheaflagallin 3-O-gallate	23.837075	-	-	-	32.76885	553.460345
POS09887	148145.022	34307.01303	-2.11043	0.194728	0.5203	0.22566	Validoxylamine A 7'-phosphate	-17.43423	-	-	-	32.7633	416.330136
POS09892	406935.2713	244152.2879	-0.73702	0.065825	0.3485	0.31666	Salviaflaside methyl ester	-6.1744512	-	-	-	32.7409	537.485964
POS09897	119127.5834	57591.42261	-1.04858	0.161898	0.4894	0.16567	Raffinose	-5.2019661	-	-	-	32.6896	505.441753
POS09898	164554.7147	153019.0623	-0.10486	0.540021	0.7619	0.04362	3-Hydroxykynurenine	1.9690688	-	-	-	32.6868	225.221018
POS09907	273454.2173	111574.5109	-1.29329	0.015874	0.2226	0.32905	Isorhamnetin 3-(6"-malonylglucoside)	9.5297496	-	-	-	32.65505	565.461756
POS09909	51909.74667	66343.60748	0.353952	0.351277	0.6364	0.07324	Sulfazecin	2.8280456	-	-	-	32.64445	397.381998
POS09910	113984.9096	40381.11105	-1.49709	0.277887	0.5938	0.16564	N-Acetylaspartylglutamylglutamate	5.3873635	-	-	-	32.638	434.376911
POS09912	80826.63694	46778.5414	-0.78898	0.164129	0.4916	0.12622	Tercozole	5.5292837	-	-	-	32.6049	533.472221
POS09914	113524.1508	188997.2763	0.735366	0.028598	0.2639	0.24256	2-Aminoadenosine	-2.0976388	-	-	-	32.593	283.262685
POS09919	80534.38141	54201.0962	-0.57128	0.271618	0.5888	0.09238	Sodium folite	13.105973	-	-	-	32.58255	496.43487
POS09920	129623.1122	133236.5741	0.039667	0.951226	0.9785	0.02629	Pteroic acid	-8.4629845	-	-	-	32.5713	313.288134
POS09921	75581.34338	56366.1182	-0.4232	0.372648	0.6502	0.08132	Violaceinic acid	2.504928	-	-	-	32.5713	374.369712
POS09922	163431.1592	79412.776	-1.04124	0.231197	0.553	0.18195	Sakuranin	14.865897	-	-	-	32.5602	449.433943
POS09923	108238.7862	75905.65359	-0.51194	0.297968	0.6114	0.10535	Cassiamin C	-17.657069	-	-	-	32.5602	507.457434
POS09924	65005.65995	75902.75174	0.223587	0.750355	0.8829	0.03709	3'-Phosphoadenylylselete	15.19398	-	-	-	32.5602	555.174996
POS09925	123006.3157	175984.5951	0.516717	0.187773	0.5138	0.14452	TG(14:0/24:1(15Z)/o-18:0)	0.3040461	-	-	-	32.5602	904.563551
POS09926	189763.5938	72019.87979	-1.39774	0.148855	0.4745	0.27184	(x)-1,2-Propanediol 1-O-b-D-glucopyranoside	-23.406418	-	-	-	32.5552	239.2367
POS09928	111070.4721	40006.04292	-1.47319	0.280659	0.5965	0.16108	Cyanidin 3-arabinoside	-11.526547	-	-	-	32.53505	420.361343
POS09933	152393.4791	82353.3218	-0.8879	0.065098	0.3462	0.19659	{11,15-dimethoxy-13-oxo-6,8,20-trioxapentacyclo[10.8.0.0i	-0.0192196	-	-	-	32.4994	437.397268
POS09934	423108.3705	380938.9242	-0.15147	0.234559	0.5554	0.12197	Syringin	-20.858033	-	-	-	32.4991	373.36661

POS09937	81372.81791	65942.52146	-0.30334	0.229644	0.5507	0.07292	6-beta-D-Glucopyranosyl-4',5-dihydroxy-3',7-dimethoxyflavone	-18.627427	-	-	-	32.4988	477.428502
POS09949	365257.3518	165816.1883	-1.13933	0.278487	0.5941	0.24122	Diloxanide furoate	12.409635	-	-	-	32.45005	329.158749
POS09958	137296.76	133511.768	-0.04033	0.861972	0.9375	0.01546	Inumakilactone A glycoside	13.927121	-	-	-	32.42965	527.501809
POS09960	108799.8598	81937.56225	-0.40908	0.205858	0.5304	0.09412	Betanin	9.8854037	-	-	-	32.4211	551.481518
POS09964	74942.38695	51050.14833	-0.55387	0.19834	0.524	0.0947	6-{[6-(3,4-dihydroxy-6-methyl-5-oxooxan-2-yl)-2-(3,4-dihydroxyphenyl)-5	-8.2074153	-	-	-	32.39655	607.491299
POS09965	185908.8373	114565.6974	-0.69842	0.121589	0.4392	0.16698	3,4,5-trihydroxy-6-({15-hydroxy-11,17,18-trimethoxy-13-oxo-6,8,20	-7.5393773	-	-	-	32.37445	577.46593
POS09966	197199.7869	134131.6889	-0.55601	0.291571	0.6056	0.11679	Clofazimine glucuronide	0.0704452	-	-	-	32.37445	666.527323
POS09970	155332.3891	154925.1087	-0.00379	0.986892	0.9932	0.00048	4'-Methylisoscutellarein 8-(2''-sulfoglucoside)	6.0480258	-	-	-	32.3526	543.477557
POS09974	94153.34706	68768.60034	-0.45326	0.193285	0.5192	0.10352	Genipin 1-beta-gentiobioside	-23.192225	-	-	-	32.3492	551.501609
POS09976	102516.3528	74275.86076	-0.46489	0.073525	0.3639	0.12187	Hesperetin 7-O-glucoside	4.2282192	-	-	-	32.3444	465.42864
POS09977	107860.1036	82240.55767	-0.39124	0.301611	0.6145	0.09505	[2,2-dimethyl-6-(3,5,7-trihydroxy-4-oxo-4H-chromen-2-yl)-3,4	-10.074426	-	-	-	32.3396	451.412739
POS09979	72797.35751	79581.22157	0.128542	0.647055	0.8267	0.03498	Iridin	14.99659	-	-	-	32.31	523.470512
POS09981	21997.53782	46790.02562	1.088859	0.051218	0.3203	0.12548	TG(14:1(9Z)/24:1(15Z)/o-18:0)	0.9921491	-	-	-	32.31	902.548171
POS09982	50876.85199	56838.80649	0.159867	0.798559	0.9083	0.00025	3-Iodo-L-tyrosine	6.4614057	-	-	-	32.2551	308.094361
POS09985	927486.208	241370.8577	-1.94207	0.260827	0.5788	0.47465	3,4,5-trihydroxy-6-{[5-hydroxy-2-(3-hydroxyphenyl)-4-oxo-8	15.117545	-	-	-	32.2544	579.495022
POS09991	93229.24285	59730.00387	-0.64233	0.132938	0.4532	0.138	Carmoisine	18.331309	-	-	-	32.22585	503.444487
POS09995	213216.7225	164272.1943	-0.37623	0.447419	0.7021	0.09062	3',5'-Diiodo-L-thyronine-beta-D-glucuronoside	4.0572035	-	-	-	32.2113	702.211222
POS10000	468939.1524	360457.2199	-0.37957	0.833974	0.9241	0.176	Amritoside	-3.4872416	-	-	-	32.1643	627.478892
POS10001	78804.38921	99240.90057	0.332659	0.090262	0.3896	0.10136	11-[(6-carboxy-3,4,5-trihydroxyoxan-2-yl)oxy]-6-hydroxy-3	-19.51764	-	-	-	32.15595	534.413866
POS10002	107886.3203	113717.0756	0.075937	0.69483	0.8493	0.02665	Streptonigrin	8.1838684	-	-	-	32.14355	507.475621
POS10003	83319.57758	68851.01639	-0.27518	0.150737	0.477	0.07265	Gentiobiosyl 2-methyl-6-oxo-2E,4E-heptadienoate	-15.240446	-	-	-	32.1355	479.444385
POS10004	327793.6127	391084.0043	0.254691	0.288693	0.603	0.14751	Myricatin	17.375772	-	-	-	32.13355	553.434875
POS10012	68433.94602	47953.51667	-0.51308	0.082487	0.3788	0.11181	1-Hydroxy-3-methoxy-7-primeverosyloxyxanthone	10.084444	-	-	-	32.1031	553.497848

POS10015	70379.62539	73250.19946	0.057675	0.75786	0.8874	0.01626	Dehypoxanthine futalosine	-5.5846853	-	-	-	32.0875	297.278222
POS10024	163251.2557	107041.918	-0.60892	0.243227	0.5621	0.12883	TG(14:1(9Z)/18:0/14:1(9Z))	-16.203467	-	-	-	32.0494	776.230015
POS10027	77537.14882	67918.5698	-0.19108	0.527737	0.756	0.05026	{2-[5,7-dihydroxy-2-(4-hydroxy-3-methoxyphenyl)-4-oxo	20.103274	-	-	-	32.0142	525.46782
POS10028	82497.43707	85902.17811	0.058345	0.877806	0.9458	0.00796	Sclerocitrin	8.3938259	-	-	-	32.0142	727.563075
POS10029	93073.26738	70572.94349	-0.39925	0.341279	0.6326	0.07818	Rheidin B	17.802735	-	-	-	32.0139	509.491329
POS10032	128817.0115	106288.7129	-0.27733	0.208379	0.5321	0.08642	Chryso-obtusin glucoside	3.1381184	-	-	-	32.006	521.49151
POS10035	433452.5281	323605.4064	-0.42164	0.087873	0.3872	0.22636	Justicidin A	14.599756	-	-	-	31.9337	395.387134
POS10037	277525.5223	348486.2927	0.328481	0.695175	0.8495	0.07887	Actinorhodin	-8.8725728	-	-	-	31.9249	635.542047
POS10042	58445.13203	57533.0193	-0.02269	0.941004	0.9734	0.00548	3,4,5-trihydroxy-6-{3-[(E)-2-(4-hydroxyphenyl)ethenyl]-5	24.624509	-	-	-	31.90395	567.533227
POS10043	64055.23259	49538.81373	-0.37076	0.118388	0.4333	0.08644	Flavonol 3-O-D-xylosylglucoside	-17.472144	-	-	-	31.89765	533.491273
POS10044	165076.9982	161410.7443	-0.0324	0.850466	0.9322	0.01604	[4-({[5,7-dihydroxy-2-(3,4,5-trihydroxyphenyl)-3,4-dihydro-2H-1	18.865863	-	-	-	31.89765	539.447435
POS10046	101785.4138	120732.7077	0.246286	0.316742	0.6253	0.0865	Luteolin 7-O-(6''-malonylglucoside)	-0.953953	-	-	-	31.8767	535.429867
POS10047	232912.7787	248698.4681	0.094608	0.89807	0.9529	0.02196	Caloxetate trisodium	-21.78691	-	-	-	31.8767	632.522718
POS10052	51222.93972	57283.78966	0.161337	0.473905	0.7203	0.03723	1,3-Dicaffeoylquinic acid	4.1159842	-	-	-	31.875	517.460302
POS10055	497182.3443	469195.762	-0.08359	0.548391	0.7686	0.08671	6-beta-Hydroxy-mometasone furoate	16.024865	-	-	-	31.8519	538.444889
POS10056	180793.3308	129676.4134	-0.47943	0.747989	0.8814	0.12433	2'-C-Methylmyricetin 3-rhamnoside 5'-gallate	-4.9743066	-	-	-	31.8476	631.51124
POS10057	119602.394	107030.9622	-0.16022	0.378632	0.6529	0.06114	N-Acetyl-N6,O-didemethylpuromycin-5'-phosphate	-6.8834567	-	-	-	31.8288	566.476384
POS10075	101498.142	57706.71948	-0.81464	0.231465	0.553	0.11572	PC(o-22:1(13Z)/20:4(8Z,11Z,14Z,17Z))	0.3949989	-	-	-	31.76565	851.249312
POS10076	38575.737	40345.95334	0.06473	0.892573	0.9506	0.00458	Sceptrin	2.7983539	-	-	-	31.7622	621.308812
POS10078	274855.9942	210606.8217	-0.38412	0.104844	0.4118	0.18674	DH	19.178641	-	-	-	31.75875	666.461039
POS10080	96798.16344	99627.51409	0.041565	0.877297	0.9458	0.01139	Dalpanin	-19.159072	-	-	-	31.7524	535.506236
POS10082	92322.95278	75670.71218	-0.28695	0.223826	0.5455	0.09144	2-(2,5-dihydroxy-4-methoxyphenyl)-5,7-dihydroxy-6-[3,4,5	22.035472	-	-	-	31.7524	611.54173
POS10083	152955.4141	131208.0782	-0.22125	0.392556	0.662	0.07066	Calcium Gluceptate	24.479849	-	-	-	31.726	491.444282

POS10087	385285.3563	466267.2108	0.27523	0.218093	0.5394	0.18546	3,3',5,5'-Tetrahydroxy-6,7-methyleneoxy-4'-methoxyflavone 3-	-18.683685	-	-	-	31.71415	537.393155
POS10089	64820.58939	76958.80015	0.247634	0.742307	0.8786	0.03901	Kaempferol 3-(6''-acetylgalactoside) 7-rhamnoside	-7.4556043	-	-	-	31.6993	637.557331
POS10092	60342.76647	42995.92576	-0.48898	0.079087	0.3738	0.09335	TG(16:0/24:0/o-18:0)	3.9864089	-	-	-	31.6817	934.636998
POS10096	50825.9574	36872.16839	-0.46303	0.026402	0.2576	0.09748	Ferrocytochrome	21.42462	-	-	-	31.6759	605.497227
POS10097	86574.26742	107372.7245	0.310617	0.30621	0.6184	0.0924	(2-{[2-(3,4-dihydroxyphenyl)-5,7-dihydroxy-4-oxo-4H-	-12.760092	-	-	-	31.67525	515.410713
POS10098	245221.0766	118370.7495	-1.05077	0.179533	0.5056	0.21161	Dynemicin A	0.6838205	-	-	-	31.6724	538.480844
POS10099	1106326.52	1279120.22	0.209375	0.303343	0.6157	0.24722	Cyanidin 3-O-(6-O-malonyl-beta-D-glucoside)	-5.0598627	-	-	-	31.67175	536.435567
POS10103	41592.89885	55432.80185	0.414403	0.574468	0.7822	0.04312	Luteolin 7-O-[beta-D-glucuronosyl-(1->2)-beta-D-glucuronide]	4.524944	-	-	-	31.627	639.494666
POS10106	22457.47391	11141.75104	-1.01122	0.285049	0.6008	0.05333	PE-NMe2(18:3(6Z,9Z,12Z)/24:1(15Z))	8.6550708	-	-	-	31.60485	853.246653
POS10107	29963.65347	28504.46245	-0.07203	0.872079	0.9428	0.00388	Amaroswerin	-24.833435	-	-	-	31.59615	603.532413
POS10108	20638.20597	15826.7751	-0.38295	0.436454	0.6941	0.0311	Alnusiin	4.1083281	-	-	-	31.5945	935.640616
POS10109	156923.4234	113704.9564	-0.46477	0.136129	0.458	0.14269	Ketoconazole	-1.1554214	-	-	-	31.588	532.437663
POS10112	36749.74668	42206.89487	0.199744	0.627624	0.8149	0.02905	3''-Deoxydihydrostreptomycin 6,3''-bis-phosphate	12.949237	-	-	-	31.5621	728.566998
POS10115	393249.724	482325.3249	0.294561	0.217652	0.5387	0.19522	1,4-beta-D-Glucan	-7.7806826	-	-	-	31.5472	537.439003
POS10132	114410.5263	50847.41393	-1.16997	0.121861	0.4395	0.1615	PC(DiMe(11,3)/DiMe(13,5))	-10.680492	-	-	-	31.4401	924.266716
POS10136	143084.6149	140306.9612	-0.02828	0.936157	0.9714	0.00429	Medicarpin 3-O-glucoside-6'-malote	3.2224304	-	-	-	31.43465	519.475647
POS10142	62469.87626	40431.21517	-0.62769	0.009858	0.194	0.13284	2,3,4,7,8,9,15,22,23,28-decahydroxy-14-(hydroxymethyl)-	4.6661101	-	-	-	31.41805	711.519592
POS10144	9227.059235	21776.30607	1.238816	0.094591	0.3981	0.08633	Rhamnazin 3-rutinoside	-7.8926346	-	-	-	31.3936	639.572937
POS10154	43046.76962	28651.36502	-0.5873	0.001285	0.084	0.11694	Molybdopterin guanine dinucleotide	2.5927489	-	-	-	31.2388	739.550491
POS10160	296782.9288	236219.2662	-0.32928	0.177286	0.5035	0.1522	Celecoxib glucuronide	-12.791582	-	-	-	31.09205	560.46912
POS10162	61158.56954	55698.66563	-0.13491	0.815872	0.9163	0.01504	DG(22:4(7Z,10Z,13Z,16Z)/24:0/0:0)	-10.861184	-	-	-	31.08755	758.219052
POS10165	50769.05654	51103.02665	0.009459	0.990685	0.9953	0.00584	6-[(2-{4-[(3-{[3,4-dihydroxy-4-({[(2E)-3-(4-hydroxyphenyl)prop-2-	-22.17899	-	-	-	31.0837	873.769919
POS10169	182693.0966	222510.6075	0.284452	0.740936	0.8779	0.05737	Chlorhexidine glucote	18.630788	-	-	-	31.07815	898.781203

POS10171	181446.4161	243012.5657	0.421487	0.676294	0.8402	0.07051	6-({3-[2-(3,4- dihydroxyphenyl)-5- hydroxy-4-oxo-7-{[3,4,5	3.8411383	-	-	-	31.0144	897.770721
POS10172	17588.28174	26953.77081	0.615873	0.540639	0.7625	0.04816	3,4,5-trihydroxy-6-(2- hydroxy-4-{3,5,7- trihydroxy-4-[3,5,7	2.1481136	-	-	-	31.0144	901.801212
POS10173	84998.32095	113374.7863	0.415594	0.630312	0.8164	0.06502	[Gallocatechin(4alpha- >8)]2catechin	7.7732788	-	-	-	30.9441	899.785463
POS10174	136915.9529	160193.7341	0.226527	0.783693	0.9006	0.00462	Octanoyl-CoA	14.837877	-	-	-	30.8923	894.750838
POS10179	162099.814	144931.1515	-0.16151	0.90704	0.9573	0.0751	2-trans,4-trans- Octadienoyl-CoA	14.157743	-	-	-	30.8034	890.718873
POS10185	70090.02853	61908.75043	-0.17907	0.900725	0.9541	0.05077	2,3-diene-Valproic acid- CoA	10.761785	-	-	-	30.7778	891.722862
POS10190	7038.658046	9846.758114	0.484348	0.568339	0.7786	0.0258	({6-[2-(3,4- dihydroxyphenyl)-4-[2- (3,4-dihydroxyphenyl)	13.899456	-	-	-	30.75885	821.738684
POS10192	35262.15891	38076.15613	0.110767	0.771187	0.8941	0.01466	Epiafzelechin-(4b->8)- epicatechin 3,3'-digallate	-16.268226	-	-	-	30.7389	867.722576
POS10194	47102.29125	68425.56543	0.538738	0.047194	0.3142	0.11961	2-Decarboxyphyllocactin	-21.569187	-	-	-	30.4068	594.507975
POS10198	329226.0732	225096.2919	-0.54854	0.339211	0.6326	0.17155	Hypericin	18.509853	-	-	-	29.9328	505.459814
POS10203	467631.5306	399678.2144	-0.22653	0.211117	0.5344	0.16491	Isoimide	-18.050406	-	-	-	29.5579	307.15635
POS10209	183986.066	193599.1877	0.073476	0.890556	0.9499	0.01067	Reduced FMN	-15.521674	-	-	-	29.0927	459.359862
POS10210	165210.0366	47005.9846	-1.81338	0.271098	0.5885	0.2105	IAA-94	10.940665	-	-	-	29.07695	358.239585
POS10223	106364.7131	125858.8479	0.242787	0.611637	0.8067	0.05077	3,4,5-trihydroxy-6-({11- hydroxy-13-oxo-6,8,20- trioxapentacyclo[10.8.0.0i cosa- 1(12),2(9),4,10,14(19),15, 17-heptaen-15- yl}oxy)oxane-2-carboxylic acid	-1.873796	-	-	-	28.6681	487.391365
POS10224	183074.1157	141351.1287	-0.37314	0.019874	0.238	0.17411	Validamycin A	-23.640045	-	-	-	28.64545	498.486316
POS10225	22328.29884	55810.63259	1.321667	0.03601	0.2855	0.15444	Carboplatin	-16.892089	-	-	-	28.6402	372.255005
POS10236	112006.9242	71342.52531	-0.65075	0.023936	0.2505	0.17981	Oleuropein	-8.260505	-	-	-	28.3777	541.516612
POS10238	44704.83468	28267.20434	-0.6613	0.04705	0.314	0.10123	N2-Maltulosylarginine	-0.1836339	-	-	-	28.3621	499.489385

POS10241	40582.29283	16096.22027	-1.33413	0.562305	0.776	0.0706	(2-{[2-(3,4-dihydroxyphenyl)-5,7-dihydroxy-4-oxo-4H-chromen-3-yl]oxy}-4-hydroxy-5-(hydroxymethyl)oxolan-3-yl)oxidanesulfonic acid	9.9763007	-	-	-	28.3459	515.422409
POS10245	955515.2442	644977.526	-0.56703	0.304866	0.6169	0.34368	L-3,4-Dihydroxybutan-2-one 4-phosphate	0.5419161	-	-	-	28.1143	185.091776
POS10247	75975.27132	71095.03375	-0.09578	0.671001	0.8378	0.02593	Melampodinin	1.2870171	-	-	-	28.0881	523.506449
POS10248	48913.96043	26615.93183	-0.87796	0.552595	0.7706	0.05724	meta-O-Dealkylated flecainide lactam	15.551175	-	-	-	27.9219	347.314362
POS10249	115771.2737	64693.32696	-0.83959	0.519826	0.7504	0.08869	Flufecet	8.1835233	-	-	-	27.8174	364.34075
POS10253	93812.97087	86695.30096	-0.11383	0.66655	0.8359	0.03519	8-Hydroxypinoresinol 4-glucoside	-19.64015	-	-	-	27.71005	537.521839
POS10259	21301.64307	5242.292369	-2.02269	0.355855	0.6395	0.06434	Proacaciberin	-14.943815	-	-	-	27.35345	392.371828
POS10264	46770.3642	34754.69192	-0.42839	0.028104	0.2629	0.08791	2-[({3,4-dihydroxy-5-[2,4,5-trihydroxy-3-(3,4,5-trihydroxybenzoyloxy)benzoyloxy]phenyl}(hydroxy)	-7.5227157	-	-	-	26.9981	548.384159
POS10267	27100572.77	20327373.96	-0.4149	0.036037	0.2855	2.2224	Calcium hydroxide	-4.2311796	-	-	-	26.6967	75.0996631
POS10268	1782838.901	1789125.567	0.005078	0.987783	0.9937	0.07207	S-(1,2-Dichlorovinyl)-L-cysteine	17.862287	-	-	-	26.6967	217.096736
POS10275	1914110.076	1650210.139	-0.21402	0.357978	0.6412	0.31039	Tetrahydroxypteridine	2.2153943	-	-	-	26.0253	197.128111
POS10280	4336385.836	3485470.186	-0.31514	0.547845	0.768	0.37039	Urate radical	19.634985	-	-	-	25.6525	168.112858
POS10300	62975.96264	98495.98899	0.645264	0.477327	0.7223	0.1169	TG(10:0/10:0/20:0)	11.835468	-	-	-	24.94065	696.138504
POS10301	8029.842837	22785.64694	1.504682	0.311532	0.6218	0.08383	DG(22:0/0:0/18:2n6)	-3.4383979	-	-	-	24.9337	692.1399
POS10309	2642084.56	4538941.495	0.780679	0.380483	0.6542	0.59284	cis-4-Carboxymethylenebut-2-en-4-olide	8.8889524	-	-	-	24.5583	141.102122
POS10310	1849848.333	1990682.232	0.105856	0.632762	0.8182	0.12943	O-Phospho-4-hydroxy-L-threonine	-19.719061	-	-	-	24.4499	216.101535
POS10317	3315839.511	2870979.033	-0.20783	0.655122	0.8304	0.27973	5-Nitrofurfural	-19.17389	-	-	-	23.98105	142.086172
POS10319	3278895.151	2072045.975	-0.66215	0.138183	0.4613	0.79377	Oxalosuccite	14.023034	-	-	-	23.9302	191.117542
POS10321	4473187.098	3219508.554	-0.47446	0.180937	0.5068	0.74224	N-Dimethyl-2-aminoethylphosphote	-9.8445841	-	-	-	23.911	154.122469
POS10330	2305034.506	2135840.367	-0.10998	0.55488	0.7716	0.19321	Citrate	13.0663	-	-	-	23.4882	193.133287

POS10334	4242.331777	45634.84197	3.427206	0.262683	0.58	0.14307		2-Octaprenyl-6-methoxyphenol	-8.0323645		-	-	-	23.2402	670.075302
POS10336	144818.6951	422272.5886	1.543927	0.326826	0.6319	0.35781		TG(10:0/10:0/18:0)	-6.839617		-	-	-	23.2356	668.071714
POS10339	77676.5094	64460.48211	-0.26906	0.269941	0.5872	0.06008		Ubiquinone-8	-3.1406503		-	-	-	23.209	728.114493
POS10341	2168794.583	2282200.165	0.073532	0.839275	0.9268	0.04213		[5-(Aminomethyl)furan-3-yl]methyl phosphate	21.452081		-	-	-	23.1623	208.13282
POS10345	11337835.25	9183953.249	-0.30396	0.403675	0.6702	0.68079		Trifluoromethanesulfonic acid	14.610323		-	-	-	22.8929	151.086469
POS10347	4879692.044	4819217.052	-0.01799	0.961893	0.9827	0.01468		Methyl 2-propenyl selenide	-12.123909		-	-	-	22.6044	136.075639
POS10352	4859728.696	4910897.731	0.015111	0.952959	0.9795	0.08572		2,3,6-Trihydroxypyridine	11.567089		-	-	-	22.0574	128.106847
POS10355	48579165.23	109917798.6	1.178015	0.131085	0.4499	6.24014		Fosetyl	-6.9645208		-	-	-	21.71785	111.05541
POS10358	3849849.052	3240775.536	-0.24846	0.432644	0.6912	0.41531		3-Oxalomalate	-8.8297326		-	-	-	21.5374	207.112457
POS10360	2045221.006	2075294.05	0.021059	0.918081	0.9625	0.09113		3-(Phosphoacetylamido)-L-alanine	8.5076785		-	-	-	21.5068	243.133137
POS10367	14610226.83	15261060.43	0.062877	0.817229	0.9166	0.20009		Cyanidin	3.2143693		-	-	-	21.2584	288.2524
POS10369	995320.2997	1091837.195	0.133525	0.725953	0.8684	0.11668		Benoxacor	-4.0698616		-	-	-	21.1565	261.122718
POS10401	2752418.515	2026948.54	-0.44139	0.381938	0.6555	0.46182		2,4-Dinitroaniline	22.330337		-	-	-	19.1812	184.132966
POS10403	2576007.123	2354682.051	-0.1296	0.649089	0.8278	0.21545		4-Nitrophenyl phosphate	1.87655		-	-	-	19.1558	220.096388
POS10407	226214.2047	234310.1156	0.05073	0.728906	0.8705	0.033		6-Oxo-2-hydroxy-7-(4'-chlorophenyl)-3,8,8-trichloroocta-2E,4E,7E-trienoate	-7.2220394		-	-	-	18.8988	383.027418
POS10410	4543312.408	3874166.967	-0.22986	0.395119	0.664	0.51743		Dichlormid	-2.6065881		-	-	-	18.6627	209.091734
POS10413	8969916.855	7700286.968	-0.22018	0.585834	0.7891	0.38336		Dihydroxyaluminium	-5.3064926		-	-	-	18.57765	138.077349
POS10418	516959.2443	429713.6311	-0.26668	0.069804	0.3563	0.23194		1,1-Dichloro-2,2-diphenylethane	1.2288856		-	-	-	18.1392	252.158685
POS10430	49802091.93	35055493.37	-0.50657	0.159648	0.4872	2.57458		Trimethylselenonium	-0.3039686		-	-	-	17.1202	125.070839
POS10437	922335.3121	803392.4882	-0.19919	0.183329	0.5093	0.19559		Se-Propenylselenocysteine Se-oxide	-3.985015		-	-	-	16.76795	225.122783
POS10443	1115666.877	1203396.412	0.109206	0.744098	0.8791	0.09819		Apraclonidine	-15.65922		-	-	-	16.1028	246.111938
POS10446	33022207.4	42926502.25	0.378432	0.569227	0.7791	1.47782		5-Diazouracil	22.115378		-	-	-	15.7555	139.09463

POS10448	349114.0846	214131.6286	-0.7052	0.088966	0.3888	0.2638	Calcium L-aspartate	-16.057771	-	-	-	15.58765	172.169328
POS10460	630200.1709	548408.617	-0.20056	0.098039	0.4044	0.20259	Mitomycin B	16.472059	-	-	-	14.1935	336.3248
POS10464	473414.5663	398558.8831	-0.24831	0.332594	0.6326	0.17136	Clavulanic acid	-19.500757	-	-	-	13.0089	200.164193
POS10469	3175519.999	3680090.613	0.212748	0.55077	0.7698	0.33717	Stipitatote	-17.878662	-	-	-	11.2219	209.128056
POS10477	2605606.695	1845920.929	-0.49728	0.56998	0.7794	0.35333	Urate radical	19.736112	-	-	-	9.9934	168.112875
POS10479	3606606.015	2821097.965	-0.35439	0.397479	0.6665	0.41656	Tetrahydroxypteridine	2.2400043	-	-	-	9.94264	197.128116
POS10484	20607203.98	9890783.421	-1.05899	0.01173	0.2033	2.97921	cis-4-Carboxymethylenebut-2-en-4-olide	8.4439709	-	-	-	9.520135	141.10206
POS10490	6045635.799	5280613.404	-0.19519	0.662379	0.8336	0.40262	5-Nitrofurfural	-19.239116	-	-	-	9.01517	142.086162
POS10493	7239646.566	5678927.796	-0.3503	0.344057	0.6332	0.82398	N-Dimethyl-2-aminoethylphosphote	-9.8827164	-	-	-	9.00287	154.122463
POS10494	78623574	33774808.77	-1.21901	0.191827	0.5185	5.19894	2,5-Furandicarboxylate	-20.140153	-	-	-	8.97005	157.097133
POS10509	3965990.576	3056172.574	-0.37596	0.020593	0.2393	0.78211	3-Fluoro-cis,cis-mucote	-0.29733	-	-	-	8.29217	161.107129
POS10511	3191022.846	2356398.314	-0.43744	0.373509	0.6502	0.40702	O-Phospho-4-hydroxy-L-threonine	-19.765299	-	-	-	8.161765	216.101525
POS10515	1318999.365	2274299.004	0.785978	0.138285	0.4613	0.64849	D-Glucurote	11.432719	-	-	-	7.9499	195.148896
POS10524	3303764.884	3002452.226	-0.13797	0.636685	0.8198	0.23771	N-Acetyldemethylphosphinothricin	20.356543	-	-	-	7.639215	210.148434
POS10525	2249062.118	1927392.177	-0.22267	0.649736	0.8284	0.19909	3-Hydroxy-2-methylpyridine-4,5-dicarboxylate	-18.676968	-	-	-	7.62119	198.148495
POS10526	4607259.066	2185962.83	-1.07564	0.011798	0.2035	1.34362	3-(Imidazol-4-yl)-2-oxopropyl phosphate	4.1477348	-	-	-	7.54582	221.12799
POS10528	3948068.272	2871237.837	-0.45947	0.113736	0.427	0.73164	3-Oxalomalate	-8.881729	-	-	-	7.505475	207.112446
POS10534	2422292.177	2027886.632	-0.2564	0.44111	0.6978	0.22859	S-(1,2-Dichlorovinyl)-L-cysteine	17.794984	-	-	-	7.34489	217.096722
POS10537	28705256.99	62920358.4	1.132212	0.138181	0.4613	4.63348	Fosetyl	-7.1332672	-	-	-	7.28604	111.055392
POS10546	3049027.724	3869863.596	0.343933	0.588594	0.7905	0.41619	meso-Tartaric acid	1.9328006	-	-	-	7.23927	151.094367
POS10548	2530535.4	1754083.883	-0.52872	0.105392	0.4127	0.6489	Calcium glycerophosphate	2.8112167	-	-	-	7.048895	211.143667
POS10549	2418664.156	2512881.969	0.055132	0.90175	0.9544	0.05187	2-C-Methyl-D-erythritol 4-phosphate	-2.3224271	-	-	-	7.00728	217.133075
POS10553	3410972.313	2367095.427	-0.52707	0.096177	0.4009	0.77274	D-Ribitol 5-phosphate	-21.781136	-	-	-	6.94283	233.127921

POS10554	1019852.278	873287.1692	-0.22383	0.326691	0.6319	0.23505	DCI	12.013088	-	-	-	6.802495	249.159158
POS10560	3118381.251	2596802.659	-0.26406	0.442655	0.6984	0.32167	D-Ribose 5-phosphate	-20.784505	-	-	-	6.66071	231.112294
POS10564	6351615.273	4384985.095	-0.53455	0.395111	0.664	0.71756	Methyl (2-propenylthio) selenide	4.6589672	-	-	-	6.60834	168.138055
POS10565	573242.1277	474741.1514	-0.272	0.025496	0.2569	0.25367	unsym-Bis(4'-chlorophenyl)ethylene	3.2436639	-	-	-	6.60377	250.143285
POS10568	1983182.09	1965422.079	-0.01298	0.974209	0.9877	0.01907	3-Sulfopyruvate	4.4226984	-	-	-	6.45244	169.13332
POS10577	2891834.04	3197920.741	0.145149	0.660189	0.8327	0.20168	2-Amino-3,4-dihydroxypentanedioic acid	14.600865	-	-	-	6.286	180.137992
POS10584	4585739.899	2992636.458	-0.61574	0.294972	0.6086	0.86185	Methyl 2-propenyl selenide	-12.145996	-	-	-	6.252305	136.075636
POS10586	3421285.672	2615531.319	-0.38743	0.323469	0.6304	0.47983	Oxalosuccite	14.090439	-	-	-	6.12628	191.117555
POS10611	730329.9042	605411.2134	-0.27063	0.039937	0.2968	0.28586	1,1-Dichloro-2,2-diphenylethane	1.2444556	-	-	-	5.252115	252.158689
POS10625	32567769.87	26546445.91	-0.29493	0.272792	0.5901	1.62906	Calcium hydroxide	-4.2441918	-	-	-	4.31168	75.0996621
POS10627	887152.6414	752903.1007	-0.23672	0.300311	0.6132	0.23859	2-Aminophenoxazin-3-one	16.613616	-	-	-	4.263305	213.214902
POS10629	547915.4971	430537.2165	-0.34782	0.20058	0.5255	0.20103	Glyphosate-monoammonium	-18.792051	-	-	-	4.25238	187.107379
POS10645	4924067.794	5197243.703	0.077896	0.874667	0.9446	0.06328	N-Formylmaleamic acid	-20.96718	-	-	-	3.58254	144.101776
POS10647	1068333.9	862898.0822	-0.3081	0.460242	0.7119	0.24933	L-3,4-Dihydroxybutan-2-one 4-phosphate	0.6190405	-	-	-	3.581505	185.091791
POS10650	4183688.069	4851233.451	0.213576	0.803859	0.9105	0.07473	6-Thiourate	3.4349839	-	-	-	3.57309	185.183809
POS10652	5024427.509	4345253.362	-0.20952	0.231489	0.553	0.45994	Cyanidin	3.1435284	-	-	-	3.57039	288.25238
POS10664	1383145.001	842264.7991	-0.71561	0.07796	0.3724	0.53705	Sepiapterin	19.330065	-	-	-	2.90197	238.227162
POS10666	1077227.193	1338051.887	0.312812	0.454679	0.7083	0.26175	1,3,7-Trimethyluric acid	9.7542221	-	-	-	2.89699	211.199327
POS10667	790033.7351	684581.7452	-0.20669	0.655983	0.8308	0.13815	Quite	-22.191808	-	-	-	2.89695	193.169612
POS10670	410929.7796	338887.9963	-0.27808	0.081883	0.3781	0.19928	5-FU	-23.631207	-	-	-	2.576725	131.081403
POS10673	1228637.593	678044.4906	-0.85761	0.007568	0.1804	0.66591	5-O-Methyl-myo-inositol	-23.282228	-	-	-	2.56663	195.185256
POS10685	224885.199	247320.1589	0.137191	0.583105	0.7875	0.06883	Dimefox	-8.5710671	-	-	-	2.22312	155.128956
POS10687	1395639.091	1860788.581	0.414988	0.053378	0.3235	0.58122	Trifluoromethyl-bismethyl ketone	16.530091	-	-	-	2.21922	141.113293

POS10689	285640.589	279292.3194	-0.03243	0.905292	0.9562	0.01812		Dopaquinone	5.8898431		-	-	-	2.218445	196.180526
POS10690	1659625.622	1870987.941	0.172942	0.600795	0.798	0.2403		5-Amino-4-imidazolecarboxyamide	-8.1380252		-	-	-	2.20621	127.12285
POS10692	3236444.42	7973347.917	1.300776	0.133627	0.454	1.63625		Pyrazinoic acid	19.592095		-	-	-	1.88285	125.107208
