# Supplementary material for: Rice SST Variation Shapes the Rhizosphere Bacterial Community, Conferring Tolerance to Salt Stress through Regulating Soil Metabolites
Source: mSystems. 2020 Nov 24;5(6):e00721-20. doi: 10.1128/mSystems.00721-20 (PMC7687028; doi:10.1128/mSystems.00721-20)
Supplement: TABLE S4 [file mSystems.00721-20-st004.pdf]

TableS4 Differentially expressed metabolites betweenNa-HHZ and Na-HHZ*cas*

index	Na-HHZ	Na-HHZ*cas*	log2_FC	Pvalue	fdr	vip	MS2 name	MS1 name	SuperClass
NEG00124	2400674.483	1	-21.19500837	0.014054446	0.305283932	2.016659573		Glyoxylate	
POS08579	13386465.09	201652.4264	-6.052760469	0.000669391	0.072093715	3.329773113		3,4-Dihydroxymandelate	
NEG06859	45093071.34	791868.872	-5.831500417	0.021103008	0.349501435	8.613515666		Urate-3-ribonucleoside	
NEG06714	38347747.97	1090104.252	-5.136603837	0.019905457	0.342903714	7.936815596		1D-1-Guanidino-3-amino-1,3-dideoxy-scyllo-inositol 4-phosphate	
NEG05758	21813999.43	697326.6584	-4.967275855	0.014696934	0.308688618	6.033726332		5'-Phosphoribosyl-N-formylglycimide	
NEG05744	1314105.215	42729.18584	-4.942715152	0.012846807	0.293169162	1.491014448		Furcozole-cis	
NEG05174	1399701.756	108351.8176	-3.691324195	0.014383459	0.308198598	1.539135855		3',5'-Cyclic IMP	
NEG06536	1643172.513	140253.1534	-3.55037884	0.028574699	0.377967259	1.541746365		Furcozole-cis	
NEG06009	1447744.023	124045.0011	-3.544871039	0.018205443	0.333199597	1.470455528		3',5'-Cyclic IMP	
NEG05452	1396435.27	153703.0363	-3.183531131	0.014112766	0.305283932	1.486150821		3',5'-Cyclic IMP	
POS08397	4401472.729	540698.7754	-3.025089335	0.004966098	0.14961244	1.700783548		3,6,8-Trimethylallantoin	
NEG05045	876532.5527	119288.7911	-2.877349183	0.011783478	0.283126866	1.143664426		3',5'-Cyclic IMP	
NEG07167	1320158.455	273073.4362	-2.273350218	0.023728695	0.359299145	1.297608348		3',5'-Cyclic IMP	
NEG05241	1185721.178	301131.7908	-1.977297873	0.035150284	0.415784531	1.184658651		2,2-Bis(4-hydroxyphenyl)hexafluoropropane	
NEG05250	898606.0444	251436.4643	-1.837494876	0.028115418	0.376102621	1.080561959		Dichlofenthion	
NEG09044	2051885.559	582227.1606	-1.817296222	0.043313521	0.446660825	1.374469508		3,4-Bis(7-chloroindol-3-yl)pyrrole-2,5-dicarboxylate	
POS00077	7205990.251	2263785.316	-1.670459552	0.027011978	0.259329536	1.995203327	2-Ethyl-5-methyloxazole		Organoheterocyclic compounds
POS00052	2627324.274	834028.4366	-1.655425795	0.005665415	0.157143689	1.216113067	Oleamide		Lipids and lipid-like molecules
POS03550	163177720.6	55161160.62	-1.564719376	0.019167247	0.234011078	8.208893111		Bromobenzene	

POS07287	2073666.679	717702.116	-1.530726935	0.005757959	0.158714878	1.118963203	Sedoheptulose 7-phosphate
NEG05379	1762097.21	621008.9816	-1.504607477	0.023265849	0.359299145	1.431834374	sn-Glycero-3-phospho-1-inositol
NEG01016	2245419.984	904517.2426	-1.311765403	0.011684671	0.281903717	1.440791682	Iodide
POS07695	39534922.7	16198577.03	-1.28726052	0.000403393	0.072093715	4.827011841	Dihydrobiopterin
NEG00793	40205697.83	16563554.6	-1.279387656	0.00118597	0.099333335	6.200354156	Dihydrogen phosphate
NEG02663	4657733.142	1973494.939	-1.238875165	0.021006207	0.348864382	1.897356946	6-Oxo-2-hydroxy-7-(4'-chlorophenyl)-3,8,8-trichloroocta-2E,4E,7E-trienoate
POS01566	2718972.526	1166547.417	-1.220816624	0.008577854	0.1899382	1.12407678	Calcium formate
POS08872	48364024.33	20797460.76	-1.2175269	0.039866595	0.296823582	4.067903918	1-Methyladenosine
POS07407	3500788.567	1513192.868	-1.21008405	0.006994102	0.176375633	1.234158248	Deoxyviolaceinic acid
POS03157	4380982.167	1900339.087	-1.204997473	0.048668171	0.31603284	1.331090573	Urate radical
POS00897	2606100.955	1139811.522	-1.19309769	0.025601099	0.256908871	1.070533686	5-O-Methyl-myo-inositol
NEG03459	2031096.533	910042.7936	-1.158252516	0.041895201	0.440482445	1.207775345	L-Selenocysteine
NEG03838	1543279.916	695378.4165	-1.150129564	0.01986807	0.342903714	1.181158113	Dehydrogenated ticlopidine
NEG00550	3210551.791	1455239.305	-1.141564857	0.004116126	0.182519682	1.792294863	(1S,3R)-3-(2,2-Dichloroethenyl)-2,2-dimethylcyclopropanecarboxylate
POS04835	7960298.105	3628347.674	-1.133509755	0.000354599	0.072093715	2.048224052	Iodate
POS07880	49191124.62	22443126.22	-1.132124388	0.000524698	0.072093715	5.14773457	Deoxyvasicinone
POS06832	8737672.346	3991349.597	-1.130372359	0.03452599	0.28295438	1.732441068	Deoxyviolaceinic acid
POS07621	4025651.244	1881720.658	-1.097169716	0.00072375	0.072093715	1.45096254	Tetrahydrobiopterin
POS07651	59076126.79	27742215.09	-1.090492257	0.000413466	0.072093715	5.603196987	Dinitrosopentamethylenetetramine
POS10526	4607259.066	2185962.83	-1.075639853	0.01179832	0.203460029	1.343615638	3-(Imidazol-4-yl)-2-oxopropyl phosphate
POS06546	4391394.393	2091108.988	-1.070410853	0.006611907	0.169578765	1.326031741	2,4-Dinitroaniline

POS10484	20607203.98	9890783.421	-1.058992069	0.011729955	0.203257003	2.979209352		cis-4-Carboxymethylenebut-2-en-4-olide	
POS07693	6893126.528	3328897.677	-1.050113971	0.000537766	0.072093715	1.872584529		2-Amino-5-(5-nitro-2-furyl)-1,3,4-thiadiazole	
POS07600	4452068.275	2156668.594	-1.045671216	0.000772286	0.072093715	1.490381798		(alpha-keto-dimethyl-delta-N,N-Dimethylguanidynol) valeric acid	
NEG02516	5330941.452	2582734.647	-1.045490911	0.020897838	0.348707276	1.885026551		6-Oxo-2-hydroxy-7-(4'-chlorophenyl)-3,8,8-trichloroocta-2E,4E,7E-trienoate	
POS01503	2039702.243	988900.9411	-1.044460644	0.000491649	0.072093715	1.002688778		Calcium formate	
POS07694	10986698.57	5386473.522	-1.028344964	0.000520172	0.072093715	2.356967592		2-Aminophenoxazin-3-one	
POS07645	2169201.407	1067441.947	-1.023006401	0.000949679	0.073627187	1.044336506		Niridazole	
POS02079	6105992.842	3026476.041	-1.012586968	0.00437771	0.140178456	1.651763758		Calcium formate	
NEG06253	3685355.249	1829700.18	-1.010196431	0.018506669	0.334446937	1.852532501		Fosetyl	
POS07996	4649288.006	2344240.448	-0.987889244	0.023815184	0.24970921	1.258437357		3-Nitroacrylate	
POS00909	8236402.461	4178650.806	-0.978977124	0.045535206	0.312252296	1.641496734		Calcium peroxide	
NEG06349	2692893.484	1384122.146	-0.960185902	0.013633444	0.301208218	1.542391919		2,2-Bis(4-hydroxyphenyl)hexafluoropropane	
POS00869	4552425.59	2343208.961	-0.958149821	0.036570901	0.286720761	1.231508071		3-Nitroacrylate	
NEG02823	2099045.144	1089199.494	-0.946464977	0.021575794	0.350245329	1.249234217		6-Oxo-2-hydroxy-7-(4'-chlorophenyl)-3,8,8-trichloroocta-2E,4E,7E-trienoate	
POS00093	14152109.2	7458545.147	-0.924050932	0.005853045	0.160921135	2.416829754	piperazine		Organoheterocyclic compounds
POS04407	3799881.9	2018150.234	-0.912921006	0.000148472	0.072093715	1.299215458		Isopentenyl phosphate	
POS00095	288193621.2	153600702.6	-0.907853588	0.03983753	0.296823582	9.076384809	Tranexamic Acid		Organic acids and derivatives
POS05619	4388865.239	2367578.812	-0.890435522	0.044237765	0.308424313	1.236467008		Pterin	
POS08374	7849444.459	4234825.755	-0.890287941	0.036552944	0.286720761	1.514142587		Caffeoyl aspartic acid	
POS00013	102559831	55778693.51	-0.878679741	0.021447563	0.243763753	5.978383523	4-Hydroxybenzylamin[illegible]		Benzenoids

POS03102	3810370.393	2083245.134	-0.871098633	0.029575156	0.267376414	1.101914763		Calcium formate	
POS00959	5205296.624	2848271.763	-0.869893573	0.045881587	0.313354961	1.192403199		L-Hexahydro-3-imino-1,2,4-oxadiazepine-3-carboxylic acid	
POS09187	17928339.45	9894325.314	-0.857568631	0.046322038	0.313354961	2.021136182		Atheroline	
POS07641	20205217.48	11160763.15	-0.856292202	0.000293785	0.072093715	3.00792197		3-Fluoro-1-(4-hydroxyphenyl)-1-propanone	
POS07812	3175824.672	1798992.812	-0.819941846	0.00992098	0.194020584	1.071433652		1,3,7-Trimethyluric acid	
POS05331	7250184.013	4170809.92	-0.797690047	0.034345131	0.282554754	1.386915106		2,3,6-Trihydroxypyridine	
POS06796	4055262.154	2336170.931	-0.795649345	0.030725041	0.269790077	1.073356022		cis-4-Carboxymethylenebut-2-en-4-olide	
POS07679	3168361.513	1865733.17	-0.763994285	0.000767463	0.072093715	1.124056944		5-O-Methyl-myo-inositol	
POS00057	8409429.499	4955991.37	-0.762834255	0.034256748	0.282044584	1.413434707	5-aminopentanoic acid		Organic acids and derivatives
POS07674	6062619.584	3620271.85	-0.743843265	0.000872095	0.073441401	1.532542106		Sepiapterin	
POS00707	4440965.275	2657453.505	-0.74082884	0.03885227	0.295117207	1.085832827		3-Nitroacrylate	
POS06392	41898081.34	25178547.01	-0.734689149	0.009051021	0.191741123	3.563688023		Calcium hydroxide	
POS07836	3722652.915	2275198.583	-0.71033864	0.008896579	0.191741123	1.024470679		p-Benzoquinone	
POS03222	13097967.79	8232708.833	-0.66990388	0.014406622	0.218215654	1.8668604		Trifluoromethyl-bismethyl ketone	
NEG00031	3253867.43	2061257.197	-0.658630942	0.019710986	0.342903714	1.473053624	(3R,4R)-3,4-dihydroxyoxolan-2-one		Organoheterocyclic compounds
NEG09490	5578482.881	3643373.461	-0.614597937	0.007169098	0.237597298	1.748089951		(1S,3R)-3-(2,2-Dichloroethenyl)-2,2-dimethylcyclopropanecarboxylate	
POS07088	11047854.46	7297549.609	-0.598282201	0.036822551	0.288299551	1.62191683		3-Deoxy-lyxo-heptulosaric acid	
POS02076	14643988.51	9778792.614	-0.582580295	0.022756729	0.247269242	1.941319102		Cyazine	
POS08129	5446483.671	3659839.766	-0.573544619	0.027942624	0.262251643	1.149414258		Tulobuterol hydrochloride	
POS04949	5495930.804	3712547.503	-0.565954355	0.020634683	0.239332829	1.081778564		Pyrazinoic acid	

NEG02269	16026636.84	11020990.4	-0.540217834	0.030830837	0.395331452	3.00835747		6-Oxo-2-hydroxy-7-(4'-chlorophenyl)-3,8,8-trichloroocta-2E,4E,7E-trienoate	
POS03315	8159010.352	5643986.411	-0.531679656	0.046494988	0.313604117	1.329232492		Nitrosylsulfuric acid	
POS08496	5305854.343	3712608.124	-0.515152028	0.025323618	0.256230931	1.074469924		D-Erythroascorbic acid 1'-a-D-xylopyranoside	
POS00073	5971477.086	4216424.797	-0.502067613	0.000635091	0.072093715	1.274855982	Ketoconazole		Organoheterocyclic compounds
POS06799	36562087.02	26256096.07	-0.477696001	0.002322685	0.106520599	3.021382474		Calcium hydroxide	
POS07771	24276037.6	17636418.45	-0.460975348	0.00535424	0.154294804	2.307344891		Calcium hydroxide	
NEG04183	8851981.532	6431807.786	-0.460776149	0.024246309	0.359490941	1.99640669		N-(3,5-Dichlorophenyl)succinimide	
POS01670	5800336.829	4252696.793	-0.447758683	0.002768694	0.114328902	1.163474616		Paramethadione	
POS00004	32970728.87	24533805.07	-0.426414774	0.032432729	0.277321862	2.474100926	(2R,3R,4S,5R)-2-(6-aminopurin-9-yl)-5		Nucleosides, nucleotides, and analogues
POS10267	27100572.77	20327373.96	-0.414899494	0.036037099	0.28549391	2.222399457		Calcium hydroxide	
NEG00016	30393216.95	22888215.35	-0.409144407	0.037998378	0.427671521	3.01950651	heptadecanoic acid		Lipids and lipid-like molecules
POS03956	6476396.699	4910717.986	-0.399257384	0.008333515	0.188101157	1.072298786		Cyazine	
NEG00067	5352784.434	4079433.36	-0.39192078	0.047831261	0.463201126	1.218132516	16-Hydroxy hexadecanoic acid		Lipids and lipid-like molecules
POS04413	10226941.74	7823855.041	-0.386423241	0.013317643	0.213541521	1.377947462		Trimethylselenonium	
NEG06275	62565109.73	48830516.05	-0.357575318	0.024893436	0.359621919	4.295807347		Nifurthiazole	
NEG06031	9458616.874	7619404.963	-0.3119509	0.001153854	0.099333335	1.827240256		Angelicin	
NEG06109	5311670.658	4314993.658	-0.299807259	0.01411994	0.305283932	1.233635314		3-hydroxy-2-methyl-2-[(sulfooxy)methyl]propanoic acid	
POS08953	15803116.43	12903069.19	-0.292494817	0.02272383	0.247269242	1.380424117		Pyrazimide	
POS04801	70641034.81	57771659.51	-0.29014454	0.022149497	0.245398822	2.867629245		Cyazine	
POS01158	8446921.389	6935637.158	-0.2843972	0.015505979	0.222611825	1.018232905		Cyazine	

NEG03785	9761883.089	8113609.789	-0.266815554	0.003041579	0.15064018	1.812468438		Ticlopidine	
NEG02965	27072440.93	23394670.24	-0.210645078	0.040957389	0.43999784	2.577398767		Radon-222	
NEG06831	7941628.166	6971706.913	-0.187922894	0.041423625	0.440339605	1.082815362		Angelicin	
NEG00018	7748543.946	6873229.283	-0.172937147	0.047974057	0.463201126	1.253973555	phthalic acid		Benzenoids
NEG06729	2662778.072	3233136.181	0.280002083	0.009142895	0.25888362	1.035657361		Bowdichione	
NEG06865	6159406.997	8717847.42	0.501180493	0.004312227	0.186047324	2.156903273		2,3,4-trihydroxy-5-(3,4,5-trihydroxybenzoyloxy)benzoic acid	
NEG06591	5792459.938	8385816.807	0.533775153	0.012539338	0.292275182	2.180862299		Arbutin 6-phosphate	
NEG06372	110621844.8	191365108.1	0.790691498	0.007651413	0.242747668	12.11408505		Coformycin	
NEG06374	21041892.86	36858955.2	0.808750687	0.009665771	0.261464066	5.32282442		3,5,7-trihydroxy-2-(3-methoxyphenyl)-5H-chromen-5-yl	
NEG06375	1850143.155	3326628.343	0.846423791	0.00971823	0.261464066	1.625766283		Salicin	
NEG09348	871273.1375	1791335.521	1.039838614	0.013573115	0.300933556	1.27795349		Arbutin 6-phosphate	
NEG04613	1973880.481	4131060.98	1.065477719	0.038821193	0.432359756	1.757012032		sn-Glycerol 3-phosphate	
NEG06239	678117.8831	1458935.249	1.105307858	0.004745103	0.192716609	1.233328723		5''-Phosphoribostamycin	
NEG08509	1409898.05	3431765.186	1.283359996	0.003303615	0.157423006	1.937830031		Procymidone	
NEG09462	1896675.723	5260165.476	1.471635145	0.00864269	0.255127434	2.375560304		2-Phosphoglycolate	
NEG08516	2651835.704	7621567.264	1.523096302	0.000683972	0.094678188	3.167868022		5-O-(1-Carboxyvinyl)-3-phosphoshikimate	
NEG08510	497880.5767	1454441.65	1.54659378	0.000643234	0.094678188	1.38435328		Chlorobenzilate	
NEG08470	1088488.619	3431107.04	1.656347812	0.000759708	0.094678188	2.172919594		Diclofec	
NEG08560	1895793.333	6165263.165	1.701360778	0.001081911	0.096846181	2.911356338		Azafenidin	
POS00033	1334822.543	4463774.318	1.741616132	0.040329121	0.297462031	1.365413309	Gentiatibetine		Organoheterocyclic compounds
NEG04724	1568132.372	5269196.865	1.748535734	0.049374635	0.471063574	1.885753473		Dihydrogen phosphate	

NEG08559	325408.723	1211059.246	1.895944614	0.000572499	0.094678188	1.339056525	1-(5-Phospho-D-ribosyl)-5-amino-4-imidazolecarboxylate
NEG08662	101038275.6	391403817.7	1.953755906	0.001588109	0.112740328	23.84965055	Etobenzanid
NEG08626	141986983.8	561944944.5	1.984670111	0.000814945	0.094678188	29.08321505	2,4-Dioxotetrahydropyrimidine D-ribonucleotide
NEG08660	2105879.633	8334610.528	1.984691807	0.001831536	0.116949658	3.483824416	Propicozole
NEG08618	2799449.052	11094864.78	1.986677255	0.000874735	0.094678188	4.089329683	Fluorodifen
NEG08665	818604.6189	3254264.674	1.99109288	0.001566504	0.112740328	2.186335036	Boscalid
NEG08628	5746092.945	23158626.25	2.010896442	0.000887737	0.094678188	5.914777729	Etacozole
NEG08584	2431169.497	10568999.76	2.120116464	0.000605775	0.094678188	4.07203107	Eudistomin G
NEG08613	175858.3844	838499.6539	2.253396068	0.000754075	0.094678188	1.150620632	3',5'-Cyclic AMP
NEG07178	2600285.883	15305188.88	2.557278698	0.000952295	0.094678188	5.055815512	Bis(4-nitrophenyl)phosphate
NEG06812	1246070.162	16439048.05	3.721669549	0.000136532	0.094678188	5.810733424	1-Methylseleno-N-acetyl-D-galactosamine
NEG08014	616726.3264	37510166.4	5.92650742	0.013389441	0.298908181	8.351596574	5-Fluorodeoxyuridine monophosphate
NEG08430	137305.6285	35672615.21	8.021282409	0.000275084	0.094678188	8.792625094	Diclofop
